# Supplementary material for: Identification of Key Residues Required for RNA Silencing Suppressor Activity of p23 Protein from a Mild Strain of Citrus Tristeza Virus
Source: Viruses. 2019 Aug 25;11(9):782. doi: 10.3390/v11090782 (PMC6784005; doi:10.3390/v11090782)
Supplement: Supplementary file 1 [file viruses-11-00782-s001.zip › Supplemental Datas.pdf]

Supplemental Data

Supplemental Data 1. Related sequences of CTV-N4VSR expression vector

Sequence of pH7LIC3.0 (35S-EV)

GGGTACCCTCGAATTATCATACATGAGAATTAAGGGAGTCACGTTATGACCCCGCCGATGACGCGGGACA
AGCCGTTTTACGTTTGGAACTGACAGAACCGCAACGTTGAAGGAGCCACTGAGCCGCGGGTTTCTGGAGT
TTAATGAGCTAAGCACATACGTCAGAAACCATTATTGCGCGTTCAAAAGTCGCCTAAGGTCACTATCAGC
TAGCAAATATTTCTTGTCAAAAATGCTCCACTGACGTTCCATAAATTCCCCTCGGTATCCAATTAGAGTC
TCATATTCACTCTCAACTCGAATCCCCCCTATGAAAAAGCCTGAACTCACCGCGACGTCTGTCGAGAAGT
TTCTGATCGAAAAGTTCGACAGCGTCTCCGACCTGATGCAGCTCTCGGAGGGCGAAGAATCTCGTGCTTT
CAGCTTCGATGTAGGAGGGCGTGGATATGTCCTGCGGGTAAATAGCTGCGCCGATGGTTTCTACAAAGAT
CGTTATGTTTATCGGCACTTTGCATCGGCCGCGCTCCCGATTCCGGAAGTGCTTGACATTGGGGAGTTCA
GCGAGAGCCTGACCTATTGCATCTCCCGCCGTGCACAGGGTGTCACGTTGCAAGACCTGCCTGAAACCGA
ACTGCCCGCTGTTCTTCAGCCGGTCGCGGAGGCTATGGATGCTATCGCTGCGGCCGATCTTAGCCAGACG
AGCGGGTTCGGCCCATTCGGACCGCAAGGAATCGGTCAATACACTACATGGCGTGATTTCATATGCGCGA
TTGCTGATCCCCATGTGTATCACTGGCAAACTGTGATGGACGACACCGTCAGTGCGTCCGTCGCGCAGGC
TCTCGATGAGCTGATGCTTTGGGCGAGGACTGCCCCGAAGTCCGGCACTCGTGCACGCGGATTCGGCTCC
AACAATGTCCTGACGGACAATGGCCGCATAACAGCGGTCATTGACTGGAGCGAGGCGATGTTCGGGGATT
CCCAATACGAGGTCGCCAACATCTTCTTCTGGAGGCCGTGGTTGGCTTGTATGGAGCAGCAGACGCGCTA
CTTCGAGCGGAGGCATCCGGAGCTTGCAGGATCGCCAAGGCTCCGGGCGTATATGCTCCGCATTGGTCTT
GACCAACTCTATCAGAGCTTGGTTGACGGCAATTTCGATGATGCAGCTTGGGCGCAGGGTCGATGCGACG
CAATCGTCCGATCCGGAGCCGGGACTGTCGGGCGTACACAAATCGCCCGCAGAAGCGCGGCCGTCTGGAC
CGATGGCTGTGTAGAAGTACTCGCCGATAGTGGAAACCGACGCCCCAGCACTCGTCCGAGGGCAAAGGAA
TAGAGTAGATGCCGACCGAACAAGAGCTGATTTCGAGAACGCCTCAGCCAGCAACTCGCGCGAGCCTAGC
AAGGCAAATGCGAGAGAACGGCCTTACGCTTGGTGGCACAGTTCTCGTCCACAGTTCGCTAAGCTCGCTC
GGCTGGGTCGCGGGAGGGCCGGTCGCAGTGATTCAGGAATTAATTCCCTAGAGTCAAGCAGATCGTTCAA
ACATTTGGCAATAAAGTTTCTTAAGATTGAATCCTGTTGCCGGTCTTGCGATGATTATCATATAATTTCT
GTTGAATTACGTTAAGCATGTAATAATTAACATGTAATGCATGACGTTATTTATGAGATGGGTTTTTATG
ATTAGAGTCCCGCAATTATACATTTAATACGCGATAGAAAACAAAATATAGCGCGCAAACTAGGATAAAT
TATCGCGCGCGGTGTCATCTATGTTACTAGATCGACCGGCATGCAAGCTGATAATTCAATTCGGCGTTAA
TTCAGTACATTAAAAACGTCCGCAATGTGTTATTAAGTTGTCTAAGCGTCAATTTGTTTACACCACAATA
TATCCTGCCACCAGCCAGCCAACAGCTCCCCGACCGGCAGCTCGGCACAAAATCACCACTCGATACAGGC
AGCCCATCAGTCCGGGACGGCGTCAGCGGGAGAGCCGTTGTAAGGCGGCAGACTTTGCTCATGTTACCGA
TGCTATTCGGAAGAACGGCAACTAAGCTGCCGGGTTTGAAACACGGATGATCTCGCGGAGGGTAGCATGT
TGATTGTAACGATGACAGAGCGTTGCTGCCTGTGATCAATTCGGGCACGAACCCAGTGGACATAAGCCTC
GTTCGGTTCGTAAGCTGTAATGCAAGTAGCGTAACTGCCGTCACGCAACTGGTCCAGAACCTTGACCGAA
CGCAGCGGTGGTAACGGCGCAGTGGCGGTTTTCATGGCTTCTTGTTATGACATGTTTTTTTGGGGTACAG
TCTATGCCTCGGGCATCCAAGCAGCAAGCGCGTTACGCCGTGGGTCGATGTTTGATGTTATGGAGCAGCA
ACGATGTTACGCAGCAGGGCAGTCGCCCTAAAACAAAGTTAAACATCATGGGGGAAGCGGTGATCGCCGA
AGTATCGACTCAACTATCAGAGGTAGTTGGCGTCATCGAGCGCCATCTCGAACCGACGTTGCTGGCCGTA
CATTTGTACGGCTCCGCAGTGGATGGCGGCCTGAAGCCACACAGTGATATTGATTTGCTGGTTACGGTGA
CCGTAAGGCTTGATGAAACAACGCGGCGAGCTTTGATCAACGACCTTTTGGAAACTTCGGCTTCCCCTGG

AGAGAGCGAGATTCTCCGCGCTGTAGAAGTCACCATTGTTGTGCACGACGACATCATTCCGTGGCGTTAT
CCAGCTAAGCGCGAACTGCAATTTGGAGAATGGCAGCGCAATGACATTCTTGCAGGTATCTTCGAGCCAG
CCACGATCGACATTGATCTGGCTATCTTGCTGACAAAAGCAAGAGAACATAGCGTTGCCTTGGTAGGTCC
AGCGGCGGAGGAACTCTTTGATCCGGTTCCTGAACAGGATCTATTTGAGGCGCTAAATGAAACCTTAACG
CTATGGAACTCGCCGCCCGACTGGGCTGGCGATGAGCGAAATGTAGTGCTTACGTTGTCCCGCATTTGGT
ACAGCGCAGTAACCGGCAAAATCGCGCCGAAGGATGTCGCTGCCGACTGGGCAATGGAGCGCCTGCCGGC
CCAGTATCAGCCCGTCATACTTGAAGCTAGACAGGCTTATCTTGGACAAGAAGAAGATCGCTTGGCCTCG
CGCGCAGATCAGTTGGAAGAATTTGTCCACTACGTGAAAGGCGAGATCACCAAGGTAGTCGGCAAATAAT
GTCTAGCTAGAAATTCGTTCAAGCCGACGCCGCTTCGCCGGCGTTAACTCAAGCGATTAGATGCACTAAG
CACATAATTGCTCACAGCCAAACTATCAGGTCAAGTCTGCTTTTATTATTTTTAAGCGTGCATAATAAGC
CCTACACAAATTGGGAGATATATCATGCATGACCAAAATCCCTTAACGTGAGTTTTCGTTCCACTGAGCG
TCAGACCCCGTAGAAAAGATCAAAGGATCTTCTTGAGATCCTTTTTTTCTGCGCGTAATCTGCTGCTTGC
AAACAAAAAAACCACCGCTACCAGCGGTGGTTTGTTTGCCGGATCAAGAGCTACCAACTCTTTTTCCGAA
GGTAACTGGCTTCAGCAGAGCGCAGATACCAAATACTGTCCTTCTAGTGTAGCCGTAGTTAGGCCACCAC
TTCAAGAACTCTGTAGCACCGCCTACATACCTCGCTCTGCTAATCCTGTTACCAGTGGCTGCTGCCAGTG
GCGATAAGTCGTGTCTTACCGGGTTGGACTCAAGACGATAGTTACCGGATAAGGCGCAGCGGTCGGGCTG
AACGGGGGGTTCGTGCACACAGCCCAGCTTGGAGCGAACGACCTACACCGAACTGAGATACCTACAGCGT
GAGCTATGAGAAAGCGCCACGCTTCCCGAAGGGAGAAAGGCGGACAGGTATCCGGTAAGCGGCAGGGTCG
GAACAGGAGAGCGCACGAGGGAGCTTCCAGGGGGAAACGCCTGGTATCTTTATAGTCCTGTCGGGTTTCG
CCACCTCTGACTTGAGCGTCGATTTTTGTGATGCTCGTCAGGGGGGCGGAGCCTATGGAAAAACGCCAGC
AACGCGGCCTTTTTACGGTTCCTGGCCTTTTGCTGGCCTTTTGCTCACATGTTCTTTCCTGCGTTATCCC
CTGATTCTGTGGATAACCGTATTACCGCCTTTGAGTGAGCTGATACCGCTCGCCGCAGCCGAACGACCGA
GCGCAGCGAGTCAGTGAGCGAGGAAGCGGAAGAGCGCCTGATGCGGTATTTTCTCCTTACGCATCTGTGC
GGTATTTCACACCGCATATGGTGCACTCTCAGTACAATCTGCTCTGATGCCGCATAGTTAAGCCAGTATA
CACTCCGCTATCGCTACGTGACTGGGTCATGGCTGCGCCCCGACACCCGCCAACACCCGCTGACGCGCCC
TGACGGGCTTGTCTGCTCCCGGCATCCGCTTACAGACAAGCTGTGACCGTCTCCGGGAGCTGCATGTGTC
AGAGGTTTTCACCGTCATCACCGAAACGCGCGAGGCAGGGTGCCTTGATGTGGGCGCCGGCGGTCGAGTG
GCGACGGCGCGGCTTGTCCGCGCCCTGGTAGATTGCCTGGCCGTAGGCCAGCCATTTTTGAGCGGCCAGC
GGCCGCGATAGGCCGACGCGAAGCGGCGGGGCGTAGGGAGCGCAGCGACCGAAGGGTAGGCGCTTTTTGC
AGCTCTTCGGCTGTGCGCTGGCCAGACAGTTATGCACAGGCCAGGCGGGTTTTAAGAGTTTTAATAAGTT
TTAAAGAGTTTTAGGCGGAAAAATCGCCTTTTTTCTCTTTTATATCAGTCACTTACATGTGTGACCGGTT
CCCAATGTACGGCTTTGGGTTCCCAATGTACGGGTTCCGGTTCCCAATGTACGGCTTTGGGTTCCCAATG
TACGTGCTATCCACAGGAAAGAGACCTTTTCGACCTTTTTCCCCTGCTAGGGCAATTTGCCCTAGCATCT
GCTCCGTACATTAGGAACCGGCGGATGCTTCGCCCTCGATCAGGTTGCGGTAGCGCATGACTAGGATCGG
GCCAGCCTGCCCCGCCTCCTCCTTCAAATCGTACTCCGGCAGGTCATTTGACCCGATCAGCTTGCGCACG
GTGAAACAGAACTTCTTGAACTCTCCGGCGCTGCCACTGCGTTCGTAGATCGTCTTGAACAACCATCTGG
CTTCTGCCTTGCCTGCGGCGCGGCGTGCCAGGCGGTAGAGAAAACGGCCGATGCCGGGATCGATCAAAAA
GTAATCGGGGTGAACCGTCAGCACGTCCGGGTTCTTGCCTTCTGTGATCTCGCGGTACATCCAATCAGCT
AGCTCGATCTCGATGTACTCCGGCCGCCCGGTTTCGCTCTTTACGATCTTGTAGCGGCTAATCAAGGCTT
CACCCTCGGATACCGTCACCAGGCGGCCGTTCTTGGCCTTCTTCGTACGCTGCATGGCAACGTGCGTGGT
GTTTAACCGAATGCAGGTTTCTACCAGGTCGTCTTTCTGCTTTCCGCCATCGGCTCGCCGGCAGAACTTG
AGTACGTCCGCAACGTGTGGACGGAACACGCGGCCGGGCTTGTCTCCCTTCCCTTCCCGGTATCGGTTCA
TGGATTCGGTTAGATGGGAAACCGCCATCAGTACCAGGTCGTAATCCCACACACTGGCCATGCCGGCCGG
CCCTGCGGAAACCTCTACGTGCCCGTCTGGAAGCTCGTAGCGGATCACCTCGCCAGCTCGTCGGTCACGC

TTCGACAGACGGAAAACGGCCACGTCCATGATGCTGCGACTATCGCGGGTGCCCACGTCATAGAGCATCG
GAACGAAAAAATCTGGTTGCTCGTCGCCCTTGGGCGGCTTCCTAATCGACGGCGCACCGGCTGCCGGCGG
TTGCCGGGATTCTTTGCGGATTCGATCAGCGGCCGCTTGCCACGATTCACCGGGGCGTGCTTCTGCCTCG
ATGCGTTGCCGCTGGGCGGCCTGCGCGGCCTTCAACTTCTCCACCAGGTCATCACCCAGCGCCGCGCCGA
TTTGTACCGGGCCGGATGGTTTGCGACCGTCACGCCGATTCCTCGGGCTTGGGGGTTCCAGTGCCATTGC
AGGGCCGGCAGACAACCCAGCCGCTTACGCCTGGCCAACCGCCCGTTCCTCCACACATGGGGCATTCCAC
GGCGTCGGTGCCTGGTTGTTCTTGATTTTCCATGCCGCCTCCTTTAGCCGCTAAAATTCATCTACTCATT
TATTCATTTGCTCATTTACTCTGGTAGCTGCGCGATGTATTCAGATAGCAGCTCGGTAATGGTCTTGCCT
TGGCGTACCGCGTACATCTTCAGCTTGGTGTGATCCTCCGCCGGCAACTGAAAGTTGACCCGCTTCATGG
CTGGCGTGTCTGCCAGGCTGGCCAACGTTGCAGCCTTGCTGCTGCGTGCGCTCGGACGGCCGGCACTTAG
CGTGTTTGTGCTTTTGCTCATTTTCTCTTTACCTCATTAACTCAAATGAGTTTTGATTTAATTTCAGCGG
CCAGCGCCTGGACCTCGCGGGCAGCGTCGCCCTCGGGTTCTGATTCAAGAACGGTTGTGCCGGCGGCGGC
AGTGCCTGGGTAGCTCACGCGCTGCGTGATACGGGACTCAAGAATGGGCAGCTCGTACCCGGCCAGCGCC
TCGGCAACCTCACCGCCGATGCGCGTGCCTTTGATCGCCCGCGACACGACAAAGGCCGCTTGTAGCCTTC
CATCCGTGACCTCAATGCGCTGCTTAACCAGCTCCACCAGGTCGGCGGTGGCCCATATGTCGTAAGGGCT
TGGCTGCACCGGAATCAGCACGAAGTCGGCTGCCTTGATCGCGGACACAGCCAAGTCCGCCGCCTGGGGC
GCTCCGTCGATCACTACGAAGTCGCGCCGGCCGATGGCCTTCACGTCGCGGTCAATCGTCGGGCGGTCGA
TGCCGACAACGGTTAGCGGTTGATCTTCCCGCACGGCCGCCCAATCGCGGGCACTGCCCTGGGGATCGGA
ATCGACTAACAGAACATCGGCCCCGGCGAGTTGCAGGGCGCGGGCTAGATGGGTTGCGATGGTCGTCTTG
CCTGACCCGCCTTTCTGGTTAAGTACAGCGATAACCTTCATGCGTTCCCCTTGCGTATTTGTTTATTTAC
TCATCGCATCATATACGCAGCGACCGCATGACGCAAGCTGTTTTACTCAAATACACATCACCTTTTTAGA
CGGCGGCGCTCGGTTTCTTCAGCGGCCAAGCTGGCCGGCCAGGCCGCCAGCTTGGCATCAGACAAACCGG
CCAGGATTTCATGCAGCCGCACGGTTGAGACGTGCGCGGGCGGCTCGAACACGTACCCGGCCGCGATCAT
CTCCGCCTCGATCTCTTCGGTAATGAAAAACGGTTCGTCCTGGCCGTCCTGGTGCGGTTTCATGCTTGTT
CCTCTTGGCGTTCATTCTCGGCGGCCGCCAGGGCGTCGGCCTCGGTCAATGCGTCCTCACGGAAGGCACC
GCGCCGCCTGGCCTCGGTGGGCGTCACTTCCTCGCTGCGCTCAAGTGCGCGGTACAGGGTCGAGCGATGC
ACGCCAAGCAGTGCAGCCGCCTCTTTCACGGTGCGGCCTTCCTGGTCGATCAGCTCGCGGGCGTGCGCGA
TCTGTGCCGGGGTGAGGGTAGGGCGGGGGCCAAACTTCACGCCTCGGGCCTTGGCGGCCTCGCGCCCGCT
CCGGGTGCGGTCGATGATTAGGGAACGCTCGAACTCGGCAATGCCGGCGAACACGGTCAACACCATGCGG
CCGGCCGGCGTGGTGGTGTCGGCCCACGGCTCTGCCAGGCTACGCAGGCCCGCGCCGGCCTCCTGGATGC
GCTCGGCAATGTCCAGTAGGTCGCGGGTGCTGCGGGCCAGGCGGTCTAGCCTGGTCACTGTCACAACGTC
GCCAGGGCGTAGGTGGTCAAGCATCCTGGCCAGCTCCGGGCGGTCGCGCCTGGTGCCGGTGATCTTCTCG
GAAAACAGCTTGGTGCAGCCGGCCGCGTGCAGTTCGGCCCGTTGGTTGGTCAAGTCCTGGTCGTCGGTGC
TGACGCGGGCATAGCCCAGCAGGCCAGCGGCGGCGCTCTTGTTCATGGCGTAATGTCTCCGGTTCTAGTC
GCAAGTATTCTACTTTATGCGACTAAAACACGCGACAAGAAAACGCCAGGAAAAGGGCAGGGCGGCAGCC
TGTCGCGTAACTTAGGACTTGTGCGACATGTCGTTTTCAGAAGACGGCTGCACTGAACGTCAGAAGCCGA
CTGCACTATAGCAGCGGAGGGGTTGGATCAAAGTACTTTGATCCCGAGGGGAACCCTGTGGTTGGCATGC
ACATACAAATGGACGAACGGATAAACCTTTTCACGCCCTTTTAAATATCCGTTATTCTAATAAACGCTCT
TTTCTCTTAGGTTTACCCGCCAATATATCCTGTCAAACACTGATAGTTTAAACTGAAGGCGGGAAACGAC
AATCTGATCCAAGCTCAAGCTAAGCTTGAGCTCTGCATGCCTGCAGGTCCCCAGATTAGCCTTTTCAATT
TCAGAAAGAATGCTAACCCACAGATGGTTAGAGAGGCTTACGCAGCAGGTCTCATCAAGACGATCTACCC
GAGCAATAATCTCCAGGAAATCAAATACCTTCCCAAGAAGGTTAAAGATGCAGTCAAAAGATTCAGGACT
AACTGCATCAAGAACACAGAGAAAGATATATTTCTCAAGATCAGAAGTACTATTCCAGTATGGACGATTC
AAGGCTTGCTTCACAAACCAAGGCAAGTAATAGAGATTGGAGTCTCTAAAAAGGTAGTTCCCACTGAATC

AAAGGCCATGGAGTCAAAGATTCAAATAGAGGACCTAACAGAACTCGCCGTAAAGACTGGCGAACAGTTC
ATACAGAGTCTCTTACGACTCAATGACAAGAAGAAAATCTTCGTCAACATGGTGGAGCACGACACACTTG
TCTACTCCAAAAATATCAAAGATACAGTCTCAGAAGACCAAAGGGCAATTGAGACTTTTCAACAAAGGGT
AATATCCGGAAACCTCCTCGGATTCCATTGCCCAGCTATCTGTCACTTTATTGTGAAGATAGTGGAAAAG
GAAGGTGGCTCCTACAAATGCCATCATTGCGATAAAGGAAAGGCCATCGTTGAAGATGCCTCTGCCGACA
GTGGTCCCAAAGATGGACCCCCACCCACGAGGAGCATCGTGGAAAAAGAAGACGTTCCAACCACGTCTTC
AAAGCAAGTGGATTGATGTGATATCTCCACTGACGTAAGGGATGACGCACAATCCCACTATCCTTCGCAA
GACCCTTCCTCTATATAAGGAAGTTCATTTCATTTGGAGAGAACACGGGGGACTCTAGAGGATCCAAGGA
GATATAACATGCCACCATGGATTACAAGGACGATGATGACAAGGACTACAAGGACGATGACGATAAGGAC
TATAAGGATGACGATGACAAGGATTACGCCGAGGCCTCTTCCCTAATGATATCCCGCGGCCATGCTAGAG
TCCGCAAAAATCACCAGTCTCTCTCTACAAATCTATCTCTCTCTATTTTTCTCCAGAATAATGTGTGAGT
AGTTCCCAGATAAGGGAATTAGGGTTCTTATAGGGTTTCGCTCATGTGTTGAGCATATAAGAAACCCTTA
GTATGTATTTGTATTTGTAAAATACTTCTATCAATAAAATTTCTAATTCCTAAAACCAAAATCCAGTGAC
CTGCAGGCATGCGACGTCG

Sequence of pH7LIC3.1.1-N4CP (35S-N4CP)

GGGTACCCTCGAATTATCATACATGAGAATTAAGGGAGTCACGTTATGACCCCCGCCGATGACGCGGGACA
AGCCGTTTTACGTTTGGAACTGACAGAACCGCAACGTTGAAGGAGCCACTGAGCCGCGGGTTTCTGGAGT
TTAATGAGCTAAGCACATACGTCAGAAACCATTATTGCGCGTTCAAAAGTCGCCTAAGGTCACTATCAGC
TAGCAAATATTTCTTGTCAAAAATGCTCCACTGACGTTCCATAAATTCCCCTCGGTATCCAATTAGAGTC
TCATATTCACTCTCAACTCGAATCCCCCCTATGAAAAAGCCTGAACTCACCGCGACGTCTGTCGAGAAGT
TTCTGATCGAAAAGTTCGACAGCGTCTCCGACCTGATGCAGCTCTCGGAGGGCGAAGAATCTCGTGCTTT
CAGCTTCGATGTAGGAGGGCGTGGATATGTCCTGCGGGTAAATAGCTGCGCCGATGGTTTCTACAAAGAT
CGTTATGTTTATCGGCACTTTGCATCGGCCGCGCTCCCGATTCCGGAAGTGCTTGACATTGGGGAGTTCA
GCGAGAGCCTGACCTATTGCATCTCCCGCCGTGCACAGGGTGTCACGTTGCAAGACCTGCCTGAAACCGA
ACTGCCCGCTGTTCTTCAGCCGGTCGCGGAGGCTATGGATGCTATCGCTGCGGCCGATCTTAGCCAGACG
AGCGGGTTCGGCCCATTCGGACCGCAAGGAATCGGTCAATACACTACATGGCGTGATTTCATATGCGCGA
TTGCTGATCCCCATGTGTATCACTGGCAAACTGTGATGGACGACACCGTCAGTGCGTCCGTCGCGCAGGC
TCTCGATGAGCTGATGCTTTGGGCGAGGACTGCCCCGAAGTCCGGCACTCGTGCACGCGGATTCGGCTCC
AACAATGTCCTGACGGACAATGGCCGCATAACAGCGGTCATTGACTGGAGCGAGGCGATGTTCGGGGATT
CCCAATACGAGGTCGCCAACATCTTCTTCTGGAGGCCGTGGTTGGCTTGTATGGAGCAGCAGACGCGCTA
CTTCGAGCGGAGGCATCCGGAGCTTGCAGGATCGCCAAGGCTCCGGGCGTATATGCTCCGCATTGGTCTT
GACCAACTCTATCAGAGCTTGGTTGACGGCAATTTCGATGATGCAGCTTGGGCGCAGGGTCGATGCGACG
CAATCGTCCGATCCGGAGCCGGGACTGTCGGGCGTACACAAATCGCCCGCAGAAGCGCGGCCGTCTGGAC
CGATGGCTGTGTAGAAGTACTCGCCGATAGTGGAAACCGACGCCCCAGCACTCGTCCGAGGGCAAAGGAA
TAGAGTAGATGCCGACCGAACAAGAGCTGATTTCGAGAACGCCTCAGCCAGCAACTCGCGCGAGCCTAGC
AAGGCAAATGCGAGAGAACGGCCTTACGCTTGGTGGCACAGTTCTCGTCCACAGTTCGCTAAGCTCGCTC
GGCTGGGTCGCGGGAGGGCCGGTCGCAGTGATTCAGGAATTAATTCCCTAGAGTCAAGCAGATCGTTCAA
ACATTTGGCAATAAAGTTTCTTAAGATTGAATCCTGTTGCCGGTCTTGCGATGATTATCATATAATTTCT
GTTGAATTACGTTAAGCATGTAATAATTAACATGTAATGCATGACGTTATTTATGAGATGGGTTTTTATG
ATTAGAGTCCCGCAATTATACATTTAATACGCGATAGAAAACAAAATATAGCGCGCAAACTAGGATAAAT
TATCGCGCGCGGTGTCATCTATGTTACTAGATCGACCGGCATGCAAGCTGATAATTCAATTCGGCGTTAA
TTCAGTACATTAAAAACGTCCGCAATGTGTTATTAAGTTGTCTAAGCGTCAATTTGTTTACACCACAATA
TATCCTGCCACCAGCCAGCCAACAGCTCCCCGACCGGCAGCTCGGCACAAAATCACCACTCGATACAGGC

AGCCCATCAGTCCGGGACGGCGTCAGCGGGAGAGCCGTTGTAAGGCGGCAGACTTTGCTCATGTTACCGA
TGCTATTCGGAAGAACGGCAACTAAGCTGCCGGGTTTGAAACACGGATGATCTCGCGGAGGGTAGCATGT
TGATTGTAACGATGACAGAGCGTTGCTGCCTGTGATCAATTCGGGCACGAACCCAGTGGACATAAGCCTC
GTTCGGTTCGTAAGCTGTAATGCAAGTAGCGTAACTGCCGTCACGCAACTGGTCCAGAACCTTGACCGAA
CGCAGCGGTGGTAACGGCGCAGTGGCGGTTTTCATGGCTTCTTGTTATGACATGTTTTTTTGGGGTACAG
TCTATGCCTCGGGCATCCAAGCAGCAAGCGCGTTACGCCGTGGGTCGATGTTTGATGTTATGGAGCAGCA
ACGATGTTACGCAGCAGGGCAGTCGCCCTAAAACAAAGTTAAACATCATGGGGGAAGCGGTGATCGCCGA
AGTATCGACTCAACTATCAGAGGTAGTTGGCGTCATCGAGCGCCATCTCGAACCGACGTTGCTGGCCGTA
CATTTGTACGGCTCCGCAGTGGATGGCGGCCTGAAGCCACACAGTGATATTGATTTGCTGGTTACGGTGA
CCGTAAGGCTTGATGAAACAACGCGGCGAGCTTTGATCAACGACCTTTTGGAAACTTCGGCTTCCCCTGG
AGAGAGCGAGATTCTCCGCGCTGTAGAAGTCACCATTGTTGTGCACGACGACATCATTCCGTGGCGTTAT
CCAGCTAAGCGCGAACTGCAATTTGGAGAATGGCAGCGCAATGACATTCTTGCAGGTATCTTCGAGCCAG
CCACGATCGACATTGATCTGGCTATCTTGCTGACAAAAGCAAGAGAACATAGCGTTGCCTTGGTAGGTCC
AGCGGCGGAGGAACTCTTTGATCCGGTTCCTGAACAGGATCTATTTGAGGCGCTAAATGAAACCTTAACG
CTATGGAACTCGCCGCCCGACTGGGCTGGCGATGAGCGAAATGTAGTGCTTACGTTGTCCCGCATTTGGT
ACAGCGCAGTAACCGGCAAAATCGCGCCGAAGGATGTCGCTGCCGACTGGGCAATGGAGCGCCTGCCGGC
CCAGTATCAGCCCGTCATACTTGAAGCTAGACAGGCTTATCTTGGACAAGAAGAAGATCGCTTGGCCTCG
CGCGCAGATCAGTTGGAAGAATTTGTCCACTACGTGAAAGGCGAGATCACCAAGGTAGTCGGCAAATAAT
GTCTAGCTAGAAATTCGTTCAAGCCGACGCCGCTTCGCCGGCGTTAACTCAAGCGATTAGATGCACTAAG
CACATAATTGCTCACAGCCAAACTATCAGGTCAAGTCTGCTTTTATTATTTTTAAGCGTGCATAATAAGC
CCTACACAAATTGGGAGATATATCATGCATGACCAAAATCCCTTAACGTGAGTTTTCGTTCCACTGAGCG
TCAGACCCCGTAGAAAAGATCAAAGGATCTTCTTGAGATCCTTTTTTTCTGCGCGTAATCTGCTGCTTGC
AAACAAAAAAACCACCGCTACCAGCGGTGGTTTGTTTGCCGGATCAAGAGCTACCAACTCTTTTTCCGAA
GGTAACTGGCTTCAGCAGAGCGCAGATACCAAATACTGTCCTTCTAGTGTAGCCGTAGTTAGGCCACCAC
TTCAAGAACTCTGTAGCACCGCCTACATACCTCGCTCTGCTAATCCTGTTACCAGTGGCTGCTGCCAGTG
GCGATAAGTCGTGTCTTACCGGGTTGGACTCAAGACGATAGTTACCGGATAAGGCGCAGCGGTCGGGCTG
AACGGGGGGTTCGTGCACACAGCCCAGCTTGGAGCGAACGACCTACACCGAACTGAGATACCTACAGCGT
GAGCTATGAGAAAGCGCCACGCTTCCCGAAGGGAGAAAGGCGGACAGGTATCCGGTAAGCGGCAGGGTCG
GAACAGGAGAGCGCACGAGGGAGCTTCCAGGGGGAAACGCCTGGTATCTTTATAGTCCTGTCGGGTTTCG
CCACCTCTGACTTGAGCGTCGATTTTTGTGATGCTCGTCAGGGGGGCGGAGCCTATGGAAAAACGCCAGC
AACGCGGCCTTTTTACGGTTCCTGGCCTTTTGCTGGCCTTTTGCTCACATGTTCTTTCCTGCGTTATCCC
CTGATTCTGTGGATAACCGTATTACCGCCTTTGAGTGAGCTGATACCGCTCGCCGCAGCCGAACGACCGA
GCGCAGCGAGTCAGTGAGCGAGGAAGCGGAAGAGCGCCTGATGCGGTATTTTCTCCTTACGCATCTGTGC
GGTATTTCACACCGCATATGGTGCACTCTCAGTACAATCTGCTCTGATGCCGCATAGTTAAGCCAGTATA
CACTCCGCTATCGCTACGTGACTGGGTCATGGCTGCGCCCCGACACCCGCCAACACCCGCTGACGCGCCC
TGACGGGCTTGTCTGCTCCCGGCATCCGCTTACAGACAAGCTGTGACCGTCTCCGGGAGCTGCATGTGTC
AGAGGTTTTCACCGTCATCACCGAAACGCGCGAGGCAGGGTGCCTTGATGTGGGCGCCGGCGGTCGAGTG
GCGACGGCGCGGCTTGTCCGCGCCCTGGTAGATTGCCTGGCCGTAGGCCAGCCATTTTTGAGCGGCCAGC
GGCCGCGATAGGCCGACGCGAAGCGGCGGGGCGTAGGGAGCGCAGCGACCGAAGGGTAGGCGCTTTTTGC
AGCTCTTCGGCTGTGCGCTGGCCAGACAGTTATGCACAGGCCAGGCGGGTTTTAAGAGTTTTAATAAGTT
TTAAAGAGTTTTAGGCGGAAAAATCGCCTTTTTTCTCTTTTATATCAGTCACTTACATGTGTGACCGGTT
CCCAATGTACGGCTTTGGGTTCCCAATGTACGGGTTCCGGTTCCCAATGTACGGCTTTGGGTTCCCAATG
TACGTGCTATCCACAGGAAAGAGACCTTTTCGACCTTTTTCCCCTGCTAGGGCAATTTGCCCTAGCATCT
GCTCCGTACATTAGGAACCGGCGGATGCTTCGCCCTCGATCAGGTTGCGGTAGCGCATGACTAGGATCGG

GCCAGCCTGCCCCGCCTCCTCCTTCAAATCGTACTCCGGCAGGTCATTTGACCCGATCAGCTTGCGCACG
GTGAAACAGAACTTCTTGAACTCTCCGGCGCTGCCACTGCGTTCGTAGATCGTCTTGAACAACCATCTGG
CTTCTGCCTTGCCTGCGGCGCGGCGTGCCAGGCGGTAGAGAAAACGGCCGATGCCGGGATCGATCAAAAA
GTAATCGGGGTGAACCGTCAGCACGTCCGGGTTCTTGCCTTCTGTGATCTCGCGGTACATCCAATCAGCT
AGCTCGATCTCGATGTACTCCGGCCGCCCGGTTTCGCTCTTTACGATCTTGTAGCGGCTAATCAAGGCTT
CACCCTCGGATACCGTCACCAGGCGGCCGTTCTTGGCCTTCTTCGTACGCTGCATGGCAACGTGCGTGGT
GTTTAACCGAATGCAGGTTTCTACCAGGTCGTCTTTCTGCTTTCCGCCATCGGCTCGCCGGCAGAACTTG
AGTACGTCCGCAACGTGTGGACGGAACACGCGGCCGGGCTTGTCTCCCTTCCCTTCCCGGTATCGGTTCA
TGGATTCGGTTAGATGGGAAACCGCCATCAGTACCAGGTCGTAATCCCACACACTGGCCATGCCGGCCGG
CCCTGCGGAAACCTCTACGTGCCCGTCTGGAAGCTCGTAGCGGATCACCTCGCCAGCTCGTCGGTCACGC
TTCGACAGACGGAAAACGGCCACGTCCATGATGCTGCGACTATCGCGGGTGCCCACGTCATAGAGCATCG
GAACGAAAAAATCTGGTTGCTCGTCGCCCTTGGGCGGCTTCCTAATCGACGGCGCACCGGCTGCCGGCGG
TTGCCGGGATTCTTTGCGGATTCGATCAGCGGCCGCTTGCCACGATTCACCGGGGCGTGCTTCTGCCTCG
ATGCGTTGCCGCTGGGCGGCCTGCGCGGCCTTCAACTTCTCCACCAGGTCATCACCCAGCGCCGCGCCGA
TTTGTACCGGGCCGGATGGTTTGCGACCGTCACGCCGATTCCTCGGGCTTGGGGGTTCCAGTGCCATTGC
AGGGCCGGCAGACAACCCAGCCGCTTACGCCTGGCCAACCGCCCGTTCCTCCACACATGGGGCATTCCAC
GGCGTCGGTGCCTGGTTGTTCTTGATTTTCCATGCCGCCTCCTTTAGCCGCTAAAATTCATCTACTCATT
TATTCATTTGCTCATTTACTCTGGTAGCTGCGCGATGTATTCAGATAGCAGCTCGGTAATGGTCTTGCCT
TGGCGTACCGCGTACATCTTCAGCTTGGTGTGATCCTCCGCCGGCAACTGAAAGTTGACCCGCTTCATGG
CTGGCGTGTCTGCCAGGCTGGCCAACGTTGCAGCCTTGCTGCTGCGTGCGCTCGGACGGCCGGCACTTAG
CGTGTTTGTGCTTTTGCTCATTTTCTCTTTACCTCATTAACTCAAATGAGTTTTGATTTAATTTCAGCGG
CCAGCGCCTGGACCTCGCGGGCAGCGTCGCCCTCGGGTTCTGATTCAAGAACGGTTGTGCCGGCGGCGGC
AGTGCCTGGGTAGCTCACGCGCTGCGTGATACGGGACTCAAGAATGGGCAGCTCGTACCCGGCCAGCGCC
TCGGCAACCTCACCGCCGATGCGCGTGCCTTTGATCGCCCGCGACACGACAAAGGCCGCTTGTAGCCTTC
CATCCGTGACCTCAATGCGCTGCTTAACCAGCTCCACCAGGTCGGCGGTGGCCCATATGTCGTAAGGGCT
TGGCTGCACCGGAATCAGCACGAAGTCGGCTGCCTTGATCGCGGACACAGCCAAGTCCGCCGCCTGGGGC
GCTCCGTCGATCACTACGAAGTCGCGCCGGCCGATGGCCTTCACGTCGCGGTCAATCGTCGGGCGGTCGA
TGCCGACAACGGTTAGCGGTTGATCTTCCCGCACGGCCGCCCAATCGCGGGCACTGCCCTGGGGATCGGA
ATCGACTAACAGAACATCGGCCCCGGCGAGTTGCAGGGCGCGGGCTAGATGGGTTGCGATGGTCGTCTTG
CCTGACCCGCCTTTCTGGTTAAGTACAGCGATAACCTTCATGCGTTCCCCTTGCGTATTTGTTTATTTAC
TCATCGCATCATATACGCAGCGACCGCATGACGCAAGCTGTTTTACTCAAATACACATCACCTTTTTAGA
CGGCGGCGCTCGGTTTCTTCAGCGGCCAAGCTGGCCGGCCAGGCCGCCAGCTTGGCATCAGACAAACCGG
CCAGGATTTCATGCAGCCGCACGGTTGAGACGTGCGCGGGCGGCTCGAACACGTACCCGGCCGCGATCAT
CTCCGCCTCGATCTCTTCGGTAATGAAAAACGGTTCGTCCTGGCCGTCCTGGTGCGGTTTCATGCTTGTT
CCTCTTGGCGTTCATTCTCGGCGGCCGCCAGGGCGTCGGCCTCGGTCAATGCGTCCTCACGGAAGGCACC
GCGCCGCCTGGCCTCGGTGGGCGTCACTTCCTCGCTGCGCTCAAGTGCGCGGTACAGGGTCGAGCGATGC
ACGCCAAGCAGTGCAGCCGCCTCTTTCACGGTGCGGCCTTCCTGGTCGATCAGCTCGCGGGCGTGCGCGA
TCTGTGCCGGGGTGAGGGTAGGGCGGGGGCCAAACTTCACGCCTCGGGCCTTGGCGGCCTCGCGCCCGCT
CCGGGTGCGGTCGATGATTAGGGAACGCTCGAACTCGGCAATGCCGGCGAACACGGTCAACACCATGCGG
CCGGCCGGCGTGGTGGTGTCGGCCCACGGCTCTGCCAGGCTACGCAGGCCCGCGCCGGCCTCCTGGATGC
GCTCGGCAATGTCCAGTAGGTCGCGGGTGCTGCGGGCCAGGCGGTCTAGCCTGGTCACTGTCACAACGTC
GCCAGGGCGTAGGTGGTCAAGCATCCTGGCCAGCTCCGGGCGGTCGCGCCTGGTGCCGGTGATCTTCTCG
GAAAACAGCTTGGTGCAGCCGGCCGCGTGCAGTTCGGCCCGTTGGTTGGTCAAGTCCTGGTCGTCGGTGC
TGACGCGGGCATAGCCCAGCAGGCCAGCGGCGGCGCTCTTGTTCATGGCGTAATGTCTCCGGTTCTAGTC

GCAAGTATTCTACTTTATGCGACTAAAACACGCGACAAGAAAACGCCAGGAAAAGGGCAGGGCGGCAGCC
TGTCGCGTAACTTAGGACTTGTGCGACATGTCGTTTTCAGAAGACGGCTGCACTGAACGTCAGAAGCCGA
CTGCACTATAGCAGCGGAGGGGTTGGATCAAAGTACTTTGATCCCGAGGGGAACCCTGTGGTTGGCATGC
ACATACAAATGGACGAACGGATAAACCTTTTCACGCCCTTTTAAATATCCGTTATTCTAATAAACGCTCT
TTTCTCTTAGGTTTACCCGCCAATATATCCTGTCAAACACTGATAGTTTAAACTGAAGGCGGGAAACGAC
AATCTGATCCAAGCTCAAGCTAAGCTTGAGCTCTGCATGCCTGCAGGTCCCCAGATTAGCCTTTTCAATT
TCAGAAAGAATGCTAACCCACAGATGGTTAGAGAGGCTTACGCAGCAGGTCTCATCAAGACGATCTACCC
GAGCAATAATCTCCAGGAAATCAAATACCTTCCCAAGAAGGTTAAAGATGCAGTCAAAAGATTCAGGACT
AACTGCATCAAGAACACAGAGAAAGATATATTTCTCAAGATCAGAAGTACTATTCCAGTATGGACGATTC
AAGGCTTGCTTCACAAACCAAGGCAAGTAATAGAGATTGGAGTCTCTAAAAAGGTAGTTCCCACTGAATC
AAAGGCCATGGAGTCAAAGATTCAAATAGAGGACCTAACAGAACTCGCCGTAAAGACTGGCGAACAGTTC
ATACAGAGTCTCTTACGACTCAATGACAAGAAGAAAATCTTCGTCAACATGGTGGAGCACGACACACTTG
TCTACTCCAAAAATATCAAAGATACAGTCTCAGAAGACCAAAGGGCAATTGAGACTTTTCAACAAAGGGT
AATATCCGGAAACCTCCTCGGATTCCATTGCCCAGCTATCTGTCACTTTATTGTGAAGATAGTGGAAAAG
GAAGGTGGCTCCTACAAATGCCATCATTGCGATAAAGGAAAGGCCATCGTTGAAGATGCCTCTGCCGACA
GTGGTCCCAAAGATGGACCCCCACCCACGAGGAGCATCGTGGAAAAAGAAGACGTTCCAACCACGTCTTC
AAAGCAAGTGGATTGATGTGATATCTCCACTGACGTAAGGGATGACGCACAATCCCACTATCCTTCGCAA
GACCCTTCCTCTATATAAGGAAGTTCATTTCATTTGGAGAGAACACGGGGGACTCTAGAGGATCCAAGGA
GATATAAGCCGCCACCATGGATTACAAGGACGATGATGACAAGGACTACAAGGACGATGACGATAAGGAC
TATAAGGATGACGATGACAAGGATTACGCCGAGGTCATGGACGACGAAACAAAGAAATTGAAGAACAAAA
ACAAGGAAACGAAAGAAGGCGACGAAGTTGTTGCTGCTGAGTCTTCTTTCGGTTCCGTAAACTTACACAT
CGATCCGACTCTGATAACGATGAACGATGTGCGTCAGTTGAGTACTCAACAGAATGCTGCTTTGAACAGG
GACTTATTTCTTGCTCTGAAAGGGAAGTATCCTAACTTGCCTGACAAAGATAAGGACTTTCACATAGCTA
TGATGTTATACCGATTAGCGGTTAAGAGTTCATCATTGCAAAGTGATGATGACACCACGGGCATAACGTA
CACTCGGGAGGGTGTTGAAGTAGATTTGTCTGACAAACTTTGGACCGACATCGTGTATAATTCTAAGGGT
ATTGGTAACCGAACTAACGCCCTTCGAGTCTGGGGTAGAACTAACGATGCTCTTTACTTAGCCTTTTGTA
GACAGAACCGCAATTTGAGTTATGGCGGACGTCCGCTAGATGCAGGGATTCCGGCTGGGTATCATTATTT
GTGTGCAGATTTCTTGACCGGAGCTGGCTTGACTGATTTAGAATGTGCTGTGTACATACAAGCTAAAGAA
CAATTGTTGAAAAAGCGAGGGGCTGATGAGGTTGTAGTTACTAATGTCAGGCAGCTTGGGAAATTTAACA
CACGTTAACCTCTTCCCTAATGATATCCCGCGGCCATGCTAGAGTCCGCAAAAATCACCAGTCTCTCTCT
ACAAATCTATCTCTCTCTATTTTTCTCCAGAATAATGTGTGAGTAGTTCCCAGATAAGGGAATTAGGGTT
CTTATAGGGTTTCGCTCATGTGTTGAGCATATAAGAAACCCTTAGTATGTATTTGTATTTGTAAAATACT
TCTATCAATAAAATTTCTAATTCCTAAAACCAAAATCCAGTGACCTGCAGGCATGCGACGTCG

Sequence of pH7LIC3.1.1-N4p20 (35S-N4p20)

GGGTACCCTCGAATTATCATACATGAGAATTAAGGGAGTCACGTTATGACCCCCGCCGATGACGCGGGACA
AGCCGTTTTACGTTTGGAACTGACAGAACCGCAACGTTGAAGGAGCCACTGAGCCGCGGGTTTCTGGAGT
TTAATGAGCTAAGCACATACGTCAGAAACCATTATTGCGCGTTCAAAAGTCGCCTAAGGTCACTATCAGC
TAGCAAATATTTCTTGTCAAAAATGCTCCACTGACGTTCCATAAATTCCCCTCGGTATCCAATTAGAGTC
TCATATTCACTCTCAACTCGAATCCCCCCTATGAAAAAGCCTGAACTCACCGCGACGTCTGTCGAGAAGT
TTCTGATCGAAAAGTTCGACAGCGTCTCCGACCTGATGCAGCTCTCGGAGGGCGAAGAATCTCGTGCTTT
CAGCTTCGATGTAGGAGGGCGTGGATATGTCCTGCGGGTAAATAGCTGCGCCGATGGTTTCTACAAAGAT
CGTTATGTTTATCGGCACTTTGCATCGGCCGCGCTCCCGATTCCGGAAGTGCTTGACATTGGGGAGTTCA
GCGAGAGCCTGACCTATTGCATCTCCCGCCGTGCACAGGGTGTCACGTTGCAAGACCTGCCTGAAACCGA

ACTGCCCGCTGTTCTTCAGCCGGTCGCGGAGGCTATGGATGCTATCGCTGCGGCCGATCTTAGCCAGACG
AGCGGGTTCGGCCCATTCGGACCGCAAGGAATCGGTCAATACACTACATGGCGTGATTTCATATGCGCGA
TTGCTGATCCCCATGTGTATCACTGGCAAACTGTGATGGACGACACCGTCAGTGCGTCCGTCGCGCAGGC
TCTCGATGAGCTGATGCTTTGGGCGAGGACTGCCCCGAAGTCCGGCACTCGTGCACGCGGATTCGGCTCC
AACAATGTCCTGACGGACAATGGCCGCATAACAGCGGTCATTGACTGGAGCGAGGCGATGTTCGGGGATT
CCCAATACGAGGTCGCCAACATCTTCTTCTGGAGGCCGTGGTTGGCTTGTATGGAGCAGCAGACGCGCTA
CTTCGAGCGGAGGCATCCGGAGCTTGCAGGATCGCCAAGGCTCCGGGCGTATATGCTCCGCATTGGTCTT
GACCAACTCTATCAGAGCTTGGTTGACGGCAATTTCGATGATGCAGCTTGGGCGCAGGGTCGATGCGACG
CAATCGTCCGATCCGGAGCCGGGACTGTCGGGCGTACACAAATCGCCCGCAGAAGCGCGGCCGTCTGGAC
CGATGGCTGTGTAGAAGTACTCGCCGATAGTGGAAACCGACGCCCCAGCACTCGTCCGAGGGCAAAGGAA
TAGAGTAGATGCCGACCGAACAAGAGCTGATTTCGAGAACGCCTCAGCCAGCAACTCGCGCGAGCCTAGC
AAGGCAAATGCGAGAGAACGGCCTTACGCTTGGTGGCACAGTTCTCGTCCACAGTTCGCTAAGCTCGCTC
GGCTGGGTCGCGGGAGGGCCGGTCGCAGTGATTCAGGAATTAATTCCCTAGAGTCAAGCAGATCGTTCAA
ACATTTGGCAATAAAGTTTCTTAAGATTGAATCCTGTTGCCGGTCTTGCGATGATTATCATATAATTTCT
GTTGAATTACGTTAAGCATGTAATAATTAACATGTAATGCATGACGTTATTTATGAGATGGGTTTTTATG
ATTAGAGTCCCGCAATTATACATTTAATACGCGATAGAAAACAAAATATAGCGCGCAAACTAGGATAAAT
TATCGCGCGCGGTGTCATCTATGTTACTAGATCGACCGGCATGCAAGCTGATAATTCAATTCGGCGTTAA
TTCAGTACATTAAAAACGTCCGCAATGTGTTATTAAGTTGTCTAAGCGTCAATTTGTTTACACCACAATA
TATCCTGCCACCAGCCAGCCAACAGCTCCCCGACCGGCAGCTCGGCACAAAATCACCACTCGATACAGGC
AGCCCATCAGTCCGGGACGGCGTCAGCGGGAGAGCCGTTGTAAGGCGGCAGACTTTGCTCATGTTACCGA
TGCTATTCGGAAGAACGGCAACTAAGCTGCCGGGTTTGAAACACGGATGATCTCGCGGAGGGTAGCATGT
TGATTGTAACGATGACAGAGCGTTGCTGCCTGTGATCAATTCGGGCACGAACCCAGTGGACATAAGCCTC
GTTCGGTTCGTAAGCTGTAATGCAAGTAGCGTAACTGCCGTCACGCAACTGGTCCAGAACCTTGACCGAA
CGCAGCGGTGGTAACGGCGCAGTGGCGGTTTTCATGGCTTCTTGTTATGACATGTTTTTTTGGGGTACAG
TCTATGCCTCGGGCATCCAAGCAGCAAGCGCGTTACGCCGTGGGTCGATGTTTGATGTTATGGAGCAGCA
ACGATGTTACGCAGCAGGGCAGTCGCCCTAAAACAAAGTTAAACATCATGGGGGAAGCGGTGATCGCCGA
AGTATCGACTCAACTATCAGAGGTAGTTGGCGTCATCGAGCGCCATCTCGAACCGACGTTGCTGGCCGTA
CATTTGTACGGCTCCGCAGTGGATGGCGGCCTGAAGCCACACAGTGATATTGATTTGCTGGTTACGGTGA
CCGTAAGGCTTGATGAAACAACGCGGCGAGCTTTGATCAACGACCTTTTGGAAACTTCGGCTTCCCCTGG
AGAGAGCGAGATTCTCCGCGCTGTAGAAGTCACCATTGTTGTGCACGACGACATCATTCCGTGGCGTTAT
CCAGCTAAGCGCGAACTGCAATTTGGAGAATGGCAGCGCAATGACATTCTTGCAGGTATCTTCGAGCCAG
CCACGATCGACATTGATCTGGCTATCTTGCTGACAAAAGCAAGAGAACATAGCGTTGCCTTGGTAGGTCC
AGCGGCGGAGGAACTCTTTGATCCGGTTCCTGAACAGGATCTATTTGAGGCGCTAAATGAAACCTTAACG
CTATGGAACTCGCCGCCCGACTGGGCTGGCGATGAGCGAAATGTAGTGCTTACGTTGTCCCGCATTTGGT
ACAGCGCAGTAACCGGCAAAATCGCGCCGAAGGATGTCGCTGCCGACTGGGCAATGGAGCGCCTGCCGGC
CCAGTATCAGCCCGTCATACTTGAAGCTAGACAGGCTTATCTTGGACAAGAAGAAGATCGCTTGGCCTCG
CGCGCAGATCAGTTGGAAGAATTTGTCCACTACGTGAAAGGCGAGATCACCAAGGTAGTCGGCAAATAAT
GTCTAGCTAGAAATTCGTTCAAGCCGACGCCGCTTCGCCGGCGTTAACTCAAGCGATTAGATGCACTAAG
CACATAATTGCTCACAGCCAAACTATCAGGTCAAGTCTGCTTTTATTATTTTTAAGCGTGCATAATAAGC
CCTACACAAATTGGGAGATATATCATGCATGACCAAAATCCCTTAACGTGAGTTTTCGTTCCACTGAGCG
TCAGACCCCGTAGAAAAGATCAAAGGATCTTCTTGAGATCCTTTTTTTCTGCGCGTAATCTGCTGCTTGC
AAACAAAAAAACCACCGCTACCAGCGGTGGTTTGTTTGCCGGATCAAGAGCTACCAACTCTTTTTCCGAA
GGTAACTGGCTTCAGCAGAGCGCAGATACCAAATACTGTCCTTCTAGTGTAGCCGTAGTTAGGCCACCAC
TTCAAGAACTCTGTAGCACCGCCTACATACCTCGCTCTGCTAATCCTGTTACCAGTGGCTGCTGCCAGTG

GCGATAAGTCGTGTCTTACCGGGTTGGACTCAAGACGATAGTTACCGGATAAGGCGCAGCGGTCGGGCTG
AACGGGGGGTTCGTGCACACAGCCCAGCTTGGAGCGAACGACCTACACCGAACTGAGATACCTACAGCGT
GAGCTATGAGAAAGCGCCACGCTTCCCGAAGGGAGAAAGGCGGACAGGTATCCGGTAAGCGGCAGGGTCG
GAACAGGAGAGCGCACGAGGGAGCTTCCAGGGGGAAACGCCTGGTATCTTTATAGTCCTGTCGGGTTTCG
CCACCTCTGACTTGAGCGTCGATTTTTGTGATGCTCGTCAGGGGGGCGGAGCCTATGGAAAAACGCCAGC
AACGCGGCCTTTTTACGGTTCCTGGCCTTTTGCTGGCCTTTTGCTCACATGTTCTTTCCTGCGTTATCCC
CTGATTCTGTGGATAACCGTATTACCGCCTTTGAGTGAGCTGATACCGCTCGCCGCAGCCGAACGACCGA
GCGCAGCGAGTCAGTGAGCGAGGAAGCGGAAGAGCGCCTGATGCGGTATTTTCTCCTTACGCATCTGTGC
GGTATTTCACACCGCATATGGTGCACTCTCAGTACAATCTGCTCTGATGCCGCATAGTTAAGCCAGTATA
CACTCCGCTATCGCTACGTGACTGGGTCATGGCTGCGCCCCGACACCCGCCAACACCCGCTGACGCGCCC
TGACGGGCTTGTCTGCTCCCGGCATCCGCTTACAGACAAGCTGTGACCGTCTCCGGGAGCTGCATGTGTC
AGAGGTTTTCACCGTCATCACCGAAACGCGCGAGGCAGGGTGCCTTGATGTGGGCGCCGGCGGTCGAGTG
GCGACGGCGCGGCTTGTCCGCGCCCTGGTAGATTGCCTGGCCGTAGGCCAGCCATTTTTGAGCGGCCAGC
GGCCGCGATAGGCCGACGCGAAGCGGCGGGGCGTAGGGAGCGCAGCGACCGAAGGGTAGGCGCTTTTTGC
AGCTCTTCGGCTGTGCGCTGGCCAGACAGTTATGCACAGGCCAGGCGGGTTTTAAGAGTTTTAATAAGTT
TTAAAGAGTTTTAGGCGGAAAAATCGCCTTTTTTCTCTTTTATATCAGTCACTTACATGTGTGACCGGTT
CCCAATGTACGGCTTTGGGTTCCCAATGTACGGGTTCCGGTTCCCAATGTACGGCTTTGGGTTCCCAATG
TACGTGCTATCCACAGGAAAGAGACCTTTTCGACCTTTTTCCCCTGCTAGGGCAATTTGCCCTAGCATCT
GCTCCGTACATTAGGAACCGGCGGATGCTTCGCCCTCGATCAGGTTGCGGTAGCGCATGACTAGGATCGG
GCCAGCCTGCCCCGCCTCCTCCTTCAAATCGTACTCCGGCAGGTCATTTGACCCGATCAGCTTGCGCACG
GTGAAACAGAACTTCTTGAACTCTCCGGCGCTGCCACTGCGTTCGTAGATCGTCTTGAACAACCATCTGG
CTTCTGCCTTGCCTGCGGCGCGGCGTGCCAGGCGGTAGAGAAAACGGCCGATGCCGGGATCGATCAAAAA
GTAATCGGGGTGAACCGTCAGCACGTCCGGGTTCTTGCCTTCTGTGATCTCGCGGTACATCCAATCAGCT
AGCTCGATCTCGATGTACTCCGGCCGCCCGGTTTCGCTCTTTACGATCTTGTAGCGGCTAATCAAGGCTT
CACCCTCGGATACCGTCACCAGGCGGCCGTTCTTGGCCTTCTTCGTACGCTGCATGGCAACGTGCGTGGT
GTTTAACCGAATGCAGGTTTCTACCAGGTCGTCTTTCTGCTTTCCGCCATCGGCTCGCCGGCAGAACTTG
AGTACGTCCGCAACGTGTGGACGGAACACGCGGCCGGGCTTGTCTCCCTTCCCTTCCCGGTATCGGTTCA
TGGATTCGGTTAGATGGGAAACCGCCATCAGTACCAGGTCGTAATCCCACACACTGGCCATGCCGGCCGG
CCCTGCGGAAACCTCTACGTGCCCGTCTGGAAGCTCGTAGCGGATCACCTCGCCAGCTCGTCGGTCACGC
TTCGACAGACGGAAAACGGCCACGTCCATGATGCTGCGACTATCGCGGGTGCCCACGTCATAGAGCATCG
GAACGAAAAAATCTGGTTGCTCGTCGCCCTTGGGCGGCTTCCTAATCGACGGCGCACCGGCTGCCGGCGG
TTGCCGGGATTCTTTGCGGATTCGATCAGCGGCCGCTTGCCACGATTCACCGGGGCGTGCTTCTGCCTCG
ATGCGTTGCCGCTGGGCGGCCTGCGCGGCCTTCAACTTCTCCACCAGGTCATCACCCAGCGCCGCGCCGA
TTTGTACCGGGCCGGATGGTTTGCGACCGTCACGCCGATTCCTCGGGCTTGGGGGTTCCAGTGCCATTGC
AGGGCCGGCAGACAACCCAGCCGCTTACGCCTGGCCAACCGCCCGTTCCTCCACACATGGGGCATTCCAC
GGCGTCGGTGCCTGGTTGTTCTTGATTTTCCATGCCGCCTCCTTTAGCCGCTAAAATTCATCTACTCATT
TATTCATTTGCTCATTTACTCTGGTAGCTGCGCGATGTATTCAGATAGCAGCTCGGTAATGGTCTTGCCT
TGGCGTACCGCGTACATCTTCAGCTTGGTGTGATCCTCCGCCGGCAACTGAAAGTTGACCCGCTTCATGG
CTGGCGTGTCTGCCAGGCTGGCCAACGTTGCAGCCTTGCTGCTGCGTGCGCTCGGACGGCCGGCACTTAG
CGTGTTTGTGCTTTTGCTCATTTTCTCTTTACCTCATTAACTCAAATGAGTTTTGATTTAATTTCAGCGG
CCAGCGCCTGGACCTCGCGGGCAGCGTCGCCCTCGGGTTCTGATTCAAGAACGGTTGTGCCGGCGGCGGC
AGTGCCTGGGTAGCTCACGCGCTGCGTGATACGGGACTCAAGAATGGGCAGCTCGTACCCGGCCAGCGCC
TCGGCAACCTCACCGCCGATGCGCGTGCCTTTGATCGCCCGCGACACGACAAAGGCCGCTTGTAGCCTTC
CATCCGTGACCTCAATGCGCTGCTTAACCAGCTCCACCAGGTCGGCGGTGGCCCATATGTCGTAAGGGCT

TGGCTGCACCGGAATCAGCACGAAGTCGGCTGCCTTGATCGCGGACACAGCCAAGTCCGCCGCCTGGGGC
GCTCCGTCGATCACTACGAAGTCGCGCCGGCCGATGGCCTTCACGTCGCGGTCAATCGTCGGGCGGTCGA
TGCCGACAACGGTTAGCGGTTGATCTTCCCGCACGGCCGCCCAATCGCGGGCACTGCCCTGGGGATCGGA
ATCGACTAACAGAACATCGGCCCCGGCGAGTTGCAGGGCGCGGGCTAGATGGGTTGCGATGGTCGTCTTG
CCTGACCCGCCTTTCTGGTTAAGTACAGCGATAACCTTCATGCGTTCCCCTTGCGTATTTGTTTATTTAC
TCATCGCATCATATACGCAGCGACCGCATGACGCAAGCTGTTTTACTCAAATACACATCACCTTTTTAGA
CGGCGGCGCTCGGTTTCTTCAGCGGCCAAGCTGGCCGGCCAGGCCGCCAGCTTGGCATCAGACAAACCGG
CCAGGATTTCATGCAGCCGCACGGTTGAGACGTGCGCGGGCGGCTCGAACACGTACCCGGCCGCGATCAT
CTCCGCCTCGATCTCTTCGGTAATGAAAAACGGTTCGTCCTGGCCGTCCTGGTGCGGTTTCATGCTTGTT
CCTCTTGGCGTTCATTCTCGGCGGCCGCCAGGGCGTCGGCCTCGGTCAATGCGTCCTCACGGAAGGCACC
GCGCCGCCTGGCCTCGGTGGGCGTCACTTCCTCGCTGCGCTCAAGTGCGCGGTACAGGGTCGAGCGATGC
ACGCCAAGCAGTGCAGCCGCCTCTTTCACGGTGCGGCCTTCCTGGTCGATCAGCTCGCGGGCGTGCGCGA
TCTGTGCCGGGGTGAGGGTAGGGCGGGGGCCAAACTTCACGCCTCGGGCCTTGGCGGCCTCGCGCCCGCT
CCGGGTGCGGTCGATGATTAGGGAACGCTCGAACTCGGCAATGCCGGCGAACACGGTCAACACCATGCGG
CCGGCCGGCGTGGTGGTGTCGGCCCACGGCTCTGCCAGGCTACGCAGGCCCGCGCCGGCCTCCTGGATGC
GCTCGGCAATGTCCAGTAGGTCGCGGGTGCTGCGGGCCAGGCGGTCTAGCCTGGTCACTGTCACAACGTC
GCCAGGGCGTAGGTGGTCAAGCATCCTGGCCAGCTCCGGGCGGTCGCGCCTGGTGCCGGTGATCTTCTCG
GAAAACAGCTTGGTGCAGCCGGCCGCGTGCAGTTCGGCCCGTTGGTTGGTCAAGTCCTGGTCGTCGGTGC
TGACGCGGGCATAGCCCAGCAGGCCAGCGGCGGCGCTCTTGTTCATGGCGTAATGTCTCCGGTTCTAGTC
GCAAGTATTCTACTTTATGCGACTAAAACACGCGACAAGAAAACGCCAGGAAAAGGGCAGGGCGGCAGCC
TGTCGCGTAACTTAGGACTTGTGCGACATGTCGTTTTCAGAAGACGGCTGCACTGAACGTCAGAAGCCGA
CTGCACTATAGCAGCGGAGGGGTTGGATCAAAGTACTTTGATCCCGAGGGGAACCCTGTGGTTGGCATGC
ACATACAAATGGACGAACGGATAAACCTTTTCACGCCCTTTTAAATATCCGTTATTCTAATAAACGCTCT
TTTCTCTTAGGTTTACCCGCCAATATATCCTGTCAAACACTGATAGTTTAAACTGAAGGCGGGAAACGAC
AATCTGATCCAAGCTCAAGCTAAGCTTGAGCTCTGCATGCCTGCAGGTCCCCAGATTAGCCTTTTCAATT
TCAGAAAGAATGCTAACCCACAGATGGTTAGAGAGGCTTACGCAGCAGGTCTCATCAAGACGATCTACCC
GAGCAATAATCTCCAGGAAATCAAATACCTTCCCAAGAAGGTTAAAGATGCAGTCAAAAGATTCAGGACT
AACTGCATCAAGAACACAGAGAAAGATATATTTCTCAAGATCAGAAGTACTATTCCAGTATGGACGATTC
AAGGCTTGCTTCACAAACCAAGGCAAGTAATAGAGATTGGAGTCTCTAAAAAGGTAGTTCCCACTGAATC
AAAGGCCATGGAGTCAAAGATTCAAATAGAGGACCTAACAGAACTCGCCGTAAAGACTGGCGAACAGTTC
ATACAGAGTCTCTTACGACTCAATGACAAGAAGAAAATCTTCGTCAACATGGTGGAGCACGACACACTTG
TCTACTCCAAAAATATCAAAGATACAGTCTCAGAAGACCAAAGGGCAATTGAGACTTTTCAACAAAGGGT
AATATCCGGAAACCTCCTCGGATTCCATTGCCCAGCTATCTGTCACTTTATTGTGAAGATAGTGGAAAAG
GAAGGTGGCTCCTACAAATGCCATCATTGCGATAAAGGAAAGGCCATCGTTGAAGATGCCTCTGCCGACA
GTGGTCCCAAAGATGGACCCCCACCCACGAGGAGCATCGTGGAAAAAGAAGACGTTCCAACCACGTCTTC
AAAGCAAGTGGATTGATGTGATATCTCCACTGACGTAAGGGATGACGCACAATCCCACTATCCTTCGCAA
GACCCTTCCTCTATATAAGGAAGTTCATTTCATTTGGAGAGAACACGGGGGACTCTAGAGGATCCAAGGA
GATATAAGCCGCCACCATGGATTACAAGGACGATGATGACAAGGACTACAAGGACGATGACGATAAGGAC
TATAAGGATGACGATGACAAGGATTACGCCGAGGTCATGCGAGCTTACTTTAGTGTTAACGATTACATAA
GCCTTTTGGCTAAGGTTGGTTCAGTTGTGGAACGTTTGTGCGATCCCAGCGTAACTCTCACGGAAGTGAT
GGACGAAATTAATGATTTTAACTCGTTTCTCGCTTTGGTGCACTCTATGAAGTCGGACATGAACGGCGAT
CATCAGGATGGTCACCATGAGATGGGTGAACATAAATCTCGGTTGTTGTGCAATATAGAGGCGAAACTGC
GAGTGCTTCTCGACATCATAAGACGTCGGTTTACTCGCGAAAAACTGCTCTGTACCAGTGCTACAGATGT
TGTGGGCTTCTTCGTCGTGAGGTACATGAGTTCTAGTCACACTAGTTTCGAAACCGTAATGCGAACGGAA

CTGAAGTTGGTGGTTAAAGCTGTGCTGTCGGAATTGTCCCGCACGCATAAACTAGATTTTAGCGAGCGAG
CCTTTACGGCTTACGGTATCCTTTTGCAGAAGGGCACCGTGGCAACCGTTTGCGGTCAGTTTGACATTAA
TTTAGTTTCTCCGTCTTGCGTGTGACCTCTTCCCTAATGATATCCCGCGGCCATGCTAGAGTCCGCAAAA
ATCACCAGTCTCTCTCTACAAATCTATCTCTCTCTATTTTTCTCCAGAATAATGTGTGAGTAGTTCCCAG
ATAAGGGAATTAGGGTTCTTATAGGGTTTCGCTCATGTGTTGAGCATATAAGAAACCCTTAGTATGTATT
TGTATTTGTAAAATACTTCTATCAATAAAATTTCTAATTCCTAAAACCAAAATCCAGTGACCTGCAGGCA
TGCGACGTCG

Sequence of pH7LIC3.1.1-N4p23 (35S-N4p23)

GGGTACCCTCGAATTATCATACATGAGAATTAAGGGAGTCACGTTATGACCCCCGCCGATGACGCGGGACA
AGCCGTTTTACGTTTGGAACTGACAGAACCGCAACGTTGAAGGAGCCACTGAGCCGCGGGTTTCTGGAGT
TTAATGAGCTAAGCACATACGTCAGAAACCATTATTGCGCGTTCAAAAGTCGCCTAAGGTCACTATCAGC
TAGCAAATATTTCTTGTCAAAAATGCTCCACTGACGTTCCATAAATTCCCCTCGGTATCCAATTAGAGTC
TCATATTCACTCTCAACTCGAATCCCCCCTATGAAAAAGCCTGAACTCACCGCGACGTCTGTCGAGAAGT
TTCTGATCGAAAAGTTCGACAGCGTCTCCGACCTGATGCAGCTCTCGGAGGGCGAAGAATCTCGTGCTTT
CAGCTTCGATGTAGGAGGGCGTGGATATGTCCTGCGGGTAAATAGCTGCGCCGATGGTTTCTACAAAGAT
CGTTATGTTTATCGGCACTTTGCATCGGCCGCGCTCCCGATTCCGGAAGTGCTTGACATTGGGGAGTTCA
GCGAGAGCCTGACCTATTGCATCTCCCGCCGTGCACAGGGTGTCACGTTGCAAGACCTGCCTGAAACCGA
ACTGCCCGCTGTTCTTCAGCCGGTCGCGGAGGCTATGGATGCTATCGCTGCGGCCGATCTTAGCCAGACG
AGCGGGTTCGGCCCATTCGGACCGCAAGGAATCGGTCAATACACTACATGGCGTGATTTCATATGCGCGA
TTGCTGATCCCCATGTGTATCACTGGCAAACTGTGATGGACGACACCGTCAGTGCGTCCGTCGCGCAGGC
TCTCGATGAGCTGATGCTTTGGGCGAGGACTGCCCCGAAGTCCGGCACTCGTGCACGCGGATTCGGCTCC
AACAATGTCCTGACGGACAATGGCCGCATAACAGCGGTCATTGACTGGAGCGAGGCGATGTTCGGGGATT
CCCAATACGAGGTCGCCAACATCTTCTTCTGGAGGCCGTGGTTGGCTTGTATGGAGCAGCAGACGCGCTA
CTTCGAGCGGAGGCATCCGGAGCTTGCAGGATCGCCAAGGCTCCGGGCGTATATGCTCCGCATTGGTCTT
GACCAACTCTATCAGAGCTTGGTTGACGGCAATTTCGATGATGCAGCTTGGGCGCAGGGTCGATGCGACG
CAATCGTCCGATCCGGAGCCGGGACTGTCGGGCGTACACAAATCGCCCGCAGAAGCGCGGCCGTCTGGAC
CGATGGCTGTGTAGAAGTACTCGCCGATAGTGGAAACCGACGCCCCAGCACTCGTCCGAGGGCAAAGGAA
TAGAGTAGATGCCGACCGAACAAGAGCTGATTTCGAGAACGCCTCAGCCAGCAACTCGCGCGAGCCTAGC
AAGGCAAATGCGAGAGAACGGCCTTACGCTTGGTGGCACAGTTCTCGTCCACAGTTCGCTAAGCTCGCTC
GGCTGGGTCGCGGGAGGGCCGGTCGCAGTGATTCAGGAATTAATTCCCTAGAGTCAAGCAGATCGTTCAA
ACATTTGGCAATAAAGTTTCTTAAGATTGAATCCTGTTGCCGGTCTTGCGATGATTATCATATAATTTCT
GTTGAATTACGTTAAGCATGTAATAATTAACATGTAATGCATGACGTTATTTATGAGATGGGTTTTTATG
ATTAGAGTCCCGCAATTATACATTTAATACGCGATAGAAAACAAAATATAGCGCGCAAACTAGGATAAAT
TATCGCGCGCGGTGTCATCTATGTTACTAGATCGACCGGCATGCAAGCTGATAATTCAATTCGGCGTTAA
TTCAGTACATTAAAAACGTCCGCAATGTGTTATTAAGTTGTCTAAGCGTCAATTTGTTTACACCACAATA
TATCCTGCCACCAGCCAGCCAACAGCTCCCCGACCGGCAGCTCGGCACAAAATCACCACTCGATACAGGC
AGCCCATCAGTCCGGGACGGCGTCAGCGGGAGAGCCGTTGTAAGGCGGCAGACTTTGCTCATGTTACCGA
TGCTATTCGGAAGAACGGCAACTAAGCTGCCGGGTTTGAAACACGGATGATCTCGCGGAGGGTAGCATGT
TGATTGTAACGATGACAGAGCGTTGCTGCCTGTGATCAATTCGGGCACGAACCCAGTGGACATAAGCCTC
GTTCGGTTCGTAAGCTGTAATGCAAGTAGCGTAACTGCCGTCACGCAACTGGTCCAGAACCTTGACCGAA
CGCAGCGGTGGTAACGGCGCAGTGGCGGTTTTCATGGCTTCTTGTTATGACATGTTTTTTTGGGGTACAG
TCTATGCCTCGGGCATCCAAGCAGCAAGCGCGTTACGCCGTGGGTCGATGTTTGATGTTATGGAGCAGCA
ACGATGTTACGCAGCAGGGCAGTCGCCCTAAAACAAAGTTAAACATCATGGGGGAAGCGGTGATCGCCGA

AGTATCGACTCAACTATCAGAGGTAGTTGGCGTCATCGAGCGCCATCTCGAACCGACGTTGCTGGCCGTA
CATTTGTACGGCTCCGCAGTGGATGGCGGCCTGAAGCCACACAGTGATATTGATTTGCTGGTTACGGTGA
CCGTAAGGCTTGATGAAACAACGCGGCGAGCTTTGATCAACGACCTTTTGGAAACTTCGGCTTCCCCTGG
AGAGAGCGAGATTCTCCGCGCTGTAGAAGTCACCATTGTTGTGCACGACGACATCATTCCGTGGCGTTAT
CCAGCTAAGCGCGAACTGCAATTTGGAGAATGGCAGCGCAATGACATTCTTGCAGGTATCTTCGAGCCAG
CCACGATCGACATTGATCTGGCTATCTTGCTGACAAAAGCAAGAGAACATAGCGTTGCCTTGGTAGGTCC
AGCGGCGGAGGAACTCTTTGATCCGGTTCCTGAACAGGATCTATTTGAGGCGCTAAATGAAACCTTAACG
CTATGGAACTCGCCGCCCGACTGGGCTGGCGATGAGCGAAATGTAGTGCTTACGTTGTCCCGCATTTGGT
ACAGCGCAGTAACCGGCAAAATCGCGCCGAAGGATGTCGCTGCCGACTGGGCAATGGAGCGCCTGCCGGC
CCAGTATCAGCCCGTCATACTTGAAGCTAGACAGGCTTATCTTGGACAAGAAGAAGATCGCTTGGCCTCG
CGCGCAGATCAGTTGGAAGAATTTGTCCACTACGTGAAAGGCGAGATCACCAAGGTAGTCGGCAAATAAT
GTCTAGCTAGAAATTCGTTCAAGCCGACGCCGCTTCGCCGGCGTTAACTCAAGCGATTAGATGCACTAAG
CACATAATTGCTCACAGCCAAACTATCAGGTCAAGTCTGCTTTTATTATTTTTAAGCGTGCATAATAAGC
CCTACACAAATTGGGAGATATATCATGCATGACCAAAATCCCTTAACGTGAGTTTTCGTTCCACTGAGCG
TCAGACCCCGTAGAAAAGATCAAAGGATCTTCTTGAGATCCTTTTTTTCTGCGCGTAATCTGCTGCTTGC
AAACAAAAAAACCACCGCTACCAGCGGTGGTTTGTTTGCCGGATCAAGAGCTACCAACTCTTTTTCCGAA
GGTAACTGGCTTCAGCAGAGCGCAGATACCAAATACTGTCCTTCTAGTGTAGCCGTAGTTAGGCCACCAC
TTCAAGAACTCTGTAGCACCGCCTACATACCTCGCTCTGCTAATCCTGTTACCAGTGGCTGCTGCCAGTG
GCGATAAGTCGTGTCTTACCGGGTTGGACTCAAGACGATAGTTACCGGATAAGGCGCAGCGGTCGGGCTG
AACGGGGGGTTCGTGCACACAGCCCAGCTTGGAGCGAACGACCTACACCGAACTGAGATACCTACAGCGT
GAGCTATGAGAAAGCGCCACGCTTCCCGAAGGGAGAAAGGCGGACAGGTATCCGGTAAGCGGCAGGGTCG
GAACAGGAGAGCGCACGAGGGAGCTTCCAGGGGGAAACGCCTGGTATCTTTATAGTCCTGTCGGGTTTCG
CCACCTCTGACTTGAGCGTCGATTTTTGTGATGCTCGTCAGGGGGGCGGAGCCTATGGAAAAACGCCAGC
AACGCGGCCTTTTTACGGTTCCTGGCCTTTTGCTGGCCTTTTGCTCACATGTTCTTTCCTGCGTTATCCC
CTGATTCTGTGGATAACCGTATTACCGCCTTTGAGTGAGCTGATACCGCTCGCCGCAGCCGAACGACCGA
GCGCAGCGAGTCAGTGAGCGAGGAAGCGGAAGAGCGCCTGATGCGGTATTTTCTCCTTACGCATCTGTGC
GGTATTTCACACCGCATATGGTGCACTCTCAGTACAATCTGCTCTGATGCCGCATAGTTAAGCCAGTATA
CACTCCGCTATCGCTACGTGACTGGGTCATGGCTGCGCCCCGACACCCGCCAACACCCGCTGACGCGCCC
TGACGGGCTTGTCTGCTCCCGGCATCCGCTTACAGACAAGCTGTGACCGTCTCCGGGAGCTGCATGTGTC
AGAGGTTTTCACCGTCATCACCGAAACGCGCGAGGCAGGGTGCCTTGATGTGGGCGCCGGCGGTCGAGTG
GCGACGGCGCGGCTTGTCCGCGCCCTGGTAGATTGCCTGGCCGTAGGCCAGCCATTTTTGAGCGGCCAGC
GGCCGCGATAGGCCGACGCGAAGCGGCGGGGCGTAGGGAGCGCAGCGACCGAAGGGTAGGCGCTTTTTGC
AGCTCTTCGGCTGTGCGCTGGCCAGACAGTTATGCACAGGCCAGGCGGGTTTTAAGAGTTTTAATAAGTT
TTAAAGAGTTTTAGGCGGAAAAATCGCCTTTTTTCTCTTTTATATCAGTCACTTACATGTGTGACCGGTT
CCCAATGTACGGCTTTGGGTTCCCAATGTACGGGTTCCGGTTCCCAATGTACGGCTTTGGGTTCCCAATG
TACGTGCTATCCACAGGAAAGAGACCTTTTCGACCTTTTTCCCCTGCTAGGGCAATTTGCCCTAGCATCT
GCTCCGTACATTAGGAACCGGCGGATGCTTCGCCCTCGATCAGGTTGCGGTAGCGCATGACTAGGATCGG
GCCAGCCTGCCCCGCCTCCTCCTTCAAATCGTACTCCGGCAGGTCATTTGACCCGATCAGCTTGCGCACG
GTGAAACAGAACTTCTTGAACTCTCCGGCGCTGCCACTGCGTTCGTAGATCGTCTTGAACAACCATCTGG
CTTCTGCCTTGCCTGCGGCGCGGCGTGCCAGGCGGTAGAGAAAACGGCCGATGCCGGGATCGATCAAAAA
GTAATCGGGGTGAACCGTCAGCACGTCCGGGTTCTTGCCTTCTGTGATCTCGCGGTACATCCAATCAGCT
AGCTCGATCTCGATGTACTCCGGCCGCCCGGTTTCGCTCTTTACGATCTTGTAGCGGCTAATCAAGGCTT
CACCCTCGGATACCGTCACCAGGCGGCCGTTCTTGGCCTTCTTCGTACGCTGCATGGCAACGTGCGTGGT
GTTTAACCGAATGCAGGTTTCTACCAGGTCGTCTTTCTGCTTTCCGCCATCGGCTCGCCGGCAGAACTTG

AGTACGTCCGCAACGTGTGGACGGAACACGCGGCCGGGCTTGTCTCCCTTCCCTTCCCGGTATCGGTTCA
TGGATTCGGTTAGATGGGAAACCGCCATCAGTACCAGGTCGTAATCCCACACACTGGCCATGCCGGCCGG
CCCTGCGGAAACCTCTACGTGCCCGTCTGGAAGCTCGTAGCGGATCACCTCGCCAGCTCGTCGGTCACGC
TTCGACAGACGGAAAACGGCCACGTCCATGATGCTGCGACTATCGCGGGTGCCCACGTCATAGAGCATCG
GAACGAAAAAATCTGGTTGCTCGTCGCCCTTGGGCGGCTTCCTAATCGACGGCGCACCGGCTGCCGGCGG
TTGCCGGGATTCTTTGCGGATTCGATCAGCGGCCGCTTGCCACGATTCACCGGGGCGTGCTTCTGCCTCG
ATGCGTTGCCGCTGGGCGGCCTGCGCGGCCTTCAACTTCTCCACCAGGTCATCACCCAGCGCCGCGCCGA
TTTGTACCGGGCCGGATGGTTTGCGACCGTCACGCCGATTCCTCGGGCTTGGGGGTTCCAGTGCCATTGC
AGGGCCGGCAGACAACCCAGCCGCTTACGCCTGGCCAACCGCCCGTTCCTCCACACATGGGGCATTCCAC
GGCGTCGGTGCCTGGTTGTTCTTGATTTTCCATGCCGCCTCCTTTAGCCGCTAAAATTCATCTACTCATT
TATTCATTTGCTCATTTACTCTGGTAGCTGCGCGATGTATTCAGATAGCAGCTCGGTAATGGTCTTGCCT
TGGCGTACCGCGTACATCTTCAGCTTGGTGTGATCCTCCGCCGGCAACTGAAAGTTGACCCGCTTCATGG
CTGGCGTGTCTGCCAGGCTGGCCAACGTTGCAGCCTTGCTGCTGCGTGCGCTCGGACGGCCGGCACTTAG
CGTGTTTGTGCTTTTGCTCATTTTCTCTTTACCTCATTAACTCAAATGAGTTTTGATTTAATTTCAGCGG
CCAGCGCCTGGACCTCGCGGGCAGCGTCGCCCTCGGGTTCTGATTCAAGAACGGTTGTGCCGGCGGCGGC
AGTGCCTGGGTAGCTCACGCGCTGCGTGATACGGGACTCAAGAATGGGCAGCTCGTACCCGGCCAGCGCC
TCGGCAACCTCACCGCCGATGCGCGTGCCTTTGATCGCCCGCGACACGACAAAGGCCGCTTGTAGCCTTC
CATCCGTGACCTCAATGCGCTGCTTAACCAGCTCCACCAGGTCGGCGGTGGCCCATATGTCGTAAGGGCT
TGGCTGCACCGGAATCAGCACGAAGTCGGCTGCCTTGATCGCGGACACAGCCAAGTCCGCCGCCTGGGGC
GCTCCGTCGATCACTACGAAGTCGCGCCGGCCGATGGCCTTCACGTCGCGGTCAATCGTCGGGCGGTCGA
TGCCGACAACGGTTAGCGGTTGATCTTCCCGCACGGCCGCCCAATCGCGGGCACTGCCCTGGGGATCGGA
ATCGACTAACAGAACATCGGCCCCGGCGAGTTGCAGGGCGCGGGCTAGATGGGTTGCGATGGTCGTCTTG
CCTGACCCGCCTTTCTGGTTAAGTACAGCGATAACCTTCATGCGTTCCCCTTGCGTATTTGTTTATTTAC
TCATCGCATCATATACGCAGCGACCGCATGACGCAAGCTGTTTTACTCAAATACACATCACCTTTTTAGA
CGGCGGCGCTCGGTTTCTTCAGCGGCCAAGCTGGCCGGCCAGGCCGCCAGCTTGGCATCAGACAAACCGG
CCAGGATTTCATGCAGCCGCACGGTTGAGACGTGCGCGGGCGGCTCGAACACGTACCCGGCCGCGATCAT
CTCCGCCTCGATCTCTTCGGTAATGAAAAACGGTTCGTCCTGGCCGTCCTGGTGCGGTTTCATGCTTGTT
CCTCTTGGCGTTCATTCTCGGCGGCCGCCAGGGCGTCGGCCTCGGTCAATGCGTCCTCACGGAAGGCACC
GCGCCGCCTGGCCTCGGTGGGCGTCACTTCCTCGCTGCGCTCAAGTGCGCGGTACAGGGTCGAGCGATGC
ACGCCAAGCAGTGCAGCCGCCTCTTTCACGGTGCGGCCTTCCTGGTCGATCAGCTCGCGGGCGTGCGCGA
TCTGTGCCGGGGTGAGGGTAGGGCGGGGGCCAAACTTCACGCCTCGGGCCTTGGCGGCCTCGCGCCCGCT
CCGGGTGCGGTCGATGATTAGGGAACGCTCGAACTCGGCAATGCCGGCGAACACGGTCAACACCATGCGG
CCGGCCGGCGTGGTGGTGTCGGCCCACGGCTCTGCCAGGCTACGCAGGCCCGCGCCGGCCTCCTGGATGC
GCTCGGCAATGTCCAGTAGGTCGCGGGTGCTGCGGGCCAGGCGGTCTAGCCTGGTCACTGTCACAACGTC
GCCAGGGCGTAGGTGGTCAAGCATCCTGGCCAGCTCCGGGCGGTCGCGCCTGGTGCCGGTGATCTTCTCG
GAAAACAGCTTGGTGCAGCCGGCCGCGTGCAGTTCGGCCCGTTGGTTGGTCAAGTCCTGGTCGTCGGTGC
TGACGCGGGCATAGCCCAGCAGGCCAGCGGCGGCGCTCTTGTTCATGGCGTAATGTCTCCGGTTCTAGTC
GCAAGTATTCTACTTTATGCGACTAAAACACGCGACAAGAAAACGCCAGGAAAAGGGCAGGGCGGCAGCC
TGTCGCGTAACTTAGGACTTGTGCGACATGTCGTTTTCAGAAGACGGCTGCACTGAACGTCAGAAGCCGA
CTGCACTATAGCAGCGGAGGGGTTGGATCAAAGTACTTTGATCCCGAGGGGAACCCTGTGGTTGGCATGC
ACATACAAATGGACGAACGGATAAACCTTTTCACGCCCTTTTAAATATCCGTTATTCTAATAAACGCTCT
TTTCTCTTAGGTTTACCCGCCAATATATCCTGTCAAACACTGATAGTTTAAACTGAAGGCGGGAAACGAC
AATCTGATCCAAGCTCAAGCTAAGCTTGAGCTCTGCATGCCTGCAGGTCCCCAGATTAGCCTTTTCAATT
TCAGAAAGAATGCTAACCCACAGATGGTTAGAGAGGCTTACGCAGCAGGTCTCATCAAGACGATCTACCC

GAGCAATAATCTCCAGGAAATCAAATACCTTCCCAAGAAGGTTAAAGATGCAGTCAAAAGATTCAGGACT
AACTGCATCAAGAACACAGAGAAAGATATATTTCTCAAGATCAGAAGTACTATTCCAGTATGGACGATTC
AAGGCTTGCTTCACAAACCAAGGCAAGTAATAGAGATTGGAGTCTCTAAAAAGGTAGTTCCCACTGAATC
AAAGGCCATGGAGTCAAAGATTCAAATAGAGGACCTAACAGAACTCGCCGTAAAGACTGGCGAACAGTTC
ATACAGAGTCTCTTACGACTCAATGACAAGAAGAAAATCTTCGTCAACATGGTGGAGCACGACACACTTG
TCTACTCCAAAAATATCAAAGATACAGTCTCAGAAGACCAAAGGGCAATTGAGACTTTTCAACAAAGGGT
AATATCCGGAAACCTCCTCGGATTCCATTGCCCAGCTATCTGTCACTTTATTGTGAAGATAGTGGAAAAG
GAAGGTGGCTCCTACAAATGCCATCATTGCGATAAAGGAAAGGCCATCGTTGAAGATGCCTCTGCCGACA
GTGGTCCCAAAGATGGACCCCCACCCACGAGGAGCATCGTGGAAAAAGAAGACGTTCCAACCACGTCTTC
AAAGCAAGTGGATTGATGTGATATCTCCACTGACGTAAGGGATGACGCACAATCCCACTATCCTTCGCAA
GACCCTTCCTCTATATAAGGAAGTTCATTTCATTTGGAGAGAACACGGGGGACTCTAGAGGATCCAAGGA
GATATAAGCCGCCACCATGGATTACAAGGACGATGATGACAAGGACTACAAGGACGATGACGATAAGGAC
TATAAGGATGACGATGACAAGGATTACGCCGAGGTCATGGATGATACTAGCGGACAAACTTTCGTTTCTG
TGAACCTTTCTGACGAAAGCAACACGGCAAGCACTAAAGTTGAAAACGTAAAATCGGAAGCGGATCGCTT
GGAGTTTTTACGTAAAATGAATCCCTTTATTGTTGACGCTCTGGTGCGGAAAACCAATTATCAGGGTGCT
CGCTTTCGCGCAAGAATAATAGGAGTGTGCGTGGATTGTGGTAGAAAACACGACAAGGCGCTCAAGACTG
AACGTAAGTGTAAGGTCAACAACACGCAATCTCAGAACGAGGTGGCGCATATGTTGATGCACGATCCCGT
TAAGTATTTGAACAAAAGAAAGGCTAGAGCCTTTTCTAACGCAGAGATGTTTGCGATTGAACTGGTTTCG
TACACCAAGGAAAGGCAATTGGCGGTCGATTTAGCCGCTGAAAGGGAGAAGACGAGACTGGCTCGTAGAC
ACCCAATACGTTCTCCGGAAGAAACTCCGGAACATTATAAATTCGGTATGACTGCTAAGGCAATGTTACC
GGACATCAACGCCGTAGACGTTGGTGATAACGAGGAAACTTCGTCGGAGTACCCAGTGAGTCTGAGTGTT
TCTGGCGGAGTTCTCCGTGAACACCACTTCATCTGACCTCTTCCCTAATGATATCCCGCGGCCATGCTAG
AGTCCGCAAAAATCACCAGTCTCTCTCTACAAATCTATCTCTCTCTATTTTTCTCCAGAATAATGTGTGA
GTAGTTCCCAGATAAGGGAATTAGGGTTCTTATAGGGTTTCGCTCATGTGTTGAGCATATAAGAAACCCT
TAGTATGTATTTGTATTTGTAAAATACTTCTATCAATAAAATTTCTAATTCCTAAAACCAAAATCCAGTG
ACCTGCAGGCATGCGACGTCG

Sequence of pH7LIC3.1.1-p19 (35S-p19)

GGGTACCCTCGAATTATCATACATGAGAATTAAGGGAGTCACGTTATGACCCCCGCCGATGACGCGGACA
AGCCGTTTTACGTTTGGAACTGACAGAACCGCAACGTTGAAGGAGCCACTGAGCCGCGGGTTTCTGGAGT
TTAATGAGCTAAGCACATACGTCAGAAACCATTATTGCGCGTTCAAAAGTCGCCTAAGGTCACTATCAGC
TAGCAAATATTTCTTGTCAAAAATGCTCCACTGACGTTCCATAAATTCCCCTCGGTATCCAATTAGAGTC
TCATATTCACTCTCAACTCGAATCCCCCCTATGAAAAAGCCTGAACTCACCGCGACGTCTGTCGAGAAGT
TTCTGATCGAAAAGTTCGACAGCGTCTCCGACCTGATGCAGCTCTCGGAGGGCGAAGAATCTCGTGCTTT
CAGCTTCGATGTAGGAGGGCGTGGATATGTCCTGCGGGTAAATAGCTGCGCCGATGGTTTCTACAAAGAT
CGTTATGTTTATCGGCACTTTGCATCGGCCGCGCTCCCGATTCCGGAAGTGCTTGACATTGGGGAGTTCA
GCGAGAGCCTGACCTATTGCATCTCCCGCCGTGCACAGGGTGTCACGTTGCAAGACCTGCCTGAAACCGA
ACTGCCCGCTGTTCTTCAGCCGGTCGCGGAGGCTATGGATGCTATCGCTGCGGCCGATCTTAGCCAGACG
AGCGGGTTCGGCCCATTCGGACCGCAAGGAATCGGTCAATACACTACATGGCGTGATTTCATATGCGCGA
TTGCTGATCCCCATGTGTATCACTGGCAAACTGTGATGGACGACACCGTCAGTGCGTCCGTCGCGCAGGC
TCTCGATGAGCTGATGCTTTGGGCGAGGACTGCCCCGAAGTCCGGCACTCGTGCACGCGGATTCGGCTCC
AACAATGTCCTGACGGACAATGGCCGCATAACAGCGGTCATTGACTGGAGCGAGGCGATGTTCGGGGATT
CCCAATACGAGGTCGCCAACATCTTCTTCTGGAGGCCGTGGTTGGCTTGTATGGAGCAGCAGACGCGCTA
CTTCGAGCGGAGGCATCCGGAGCTTGCAGGATCGCCAAGGCTCCGGGCGTATATGCTCCGCATTGGTCTT

GACCAACTCTATCAGAGCTTGGTTGACGGCAATTTCGATGATGCAGCTTGGGCGCAGGGTCGATGCGACG
CAATCGTCCGATCCGGAGCCGGGACTGTCGGGCGTACACAAATCGCCCGCAGAAGCGCGGCCGTCTGGAC
CGATGGCTGTGTAGAAGTACTCGCCGATAGTGGAAACCGACGCCCCAGCACTCGTCCGAGGGCAAAGGAA
TAGAGTAGATGCCGACCGAACAAGAGCTGATTTCGAGAACGCCTCAGCCAGCAACTCGCGCGAGCCTAGC
AAGGCAAATGCGAGAGAACGGCCTTACGCTTGGTGGCACAGTTCTCGTCCACAGTTCGCTAAGCTCGCTC
GGCTGGGTCGCGGGAGGGCCGGTCGCAGTGATTCAGGAATTAATTCCCTAGAGTCAAGCAGATCGTTCAA
ACATTTGGCAATAAAGTTTCTTAAGATTGAATCCTGTTGCCGGTCTTGCGATGATTATCATATAATTTCT
GTTGAATTACGTTAAGCATGTAATAATTAACATGTAATGCATGACGTTATTTATGAGATGGGTTTTTATG
ATTAGAGTCCCGCAATTATACATTTAATACGCGATAGAAAACAAAATATAGCGCGCAAACTAGGATAAAT
TATCGCGCGCGGTGTCATCTATGTTACTAGATCGACCGGCATGCAAGCTGATAATTCAATTCGGCGTTAA
TTCAGTACATTAAAAACGTCCGCAATGTGTTATTAAGTTGTCTAAGCGTCAATTTGTTTACACCACAATA
TATCCTGCCACCAGCCAGCCAACAGCTCCCCGACCGGCAGCTCGGCACAAAATCACCACTCGATACAGGC
AGCCCATCAGTCCGGGACGGCGTCAGCGGGAGAGCCGTTGTAAGGCGGCAGACTTTGCTCATGTTACCGA
TGCTATTCGGAAGAACGGCAACTAAGCTGCCGGGTTTGAAACACGGATGATCTCGCGGAGGGTAGCATGT
TGATTGTAACGATGACAGAGCGTTGCTGCCTGTGATCAATTCGGGCACGAACCCAGTGGACATAAGCCTC
GTTCGGTTCGTAAGCTGTAATGCAAGTAGCGTAACTGCCGTCACGCAACTGGTCCAGAACCTTGACCGAA
CGCAGCGGTGGTAACGGCGCAGTGGCGGTTTTCATGGCTTCTTGTTATGACATGTTTTTTTGGGGTACAG
TCTATGCCTCGGGCATCCAAGCAGCAAGCGCGTTACGCCGTGGGTCGATGTTTGATGTTATGGAGCAGCA
ACGATGTTACGCAGCAGGGCAGTCGCCCTAAAACAAAGTTAAACATCATGGGGGAAGCGGTGATCGCCGA
AGTATCGACTCAACTATCAGAGGTAGTTGGCGTCATCGAGCGCCATCTCGAACCGACGTTGCTGGCCGTA
CATTTGTACGGCTCCGCAGTGGATGGCGGCCTGAAGCCACACAGTGATATTGATTTGCTGGTTACGGTGA
CCGTAAGGCTTGATGAAACAACGCGGCGAGCTTTGATCAACGACCTTTTGGAAACTTCGGCTTCCCCTGG
AGAGAGCGAGATTCTCCGCGCTGTAGAAGTCACCATTGTTGTGCACGACGACATCATTCCGTGGCGTTAT
CCAGCTAAGCGCGAACTGCAATTTGGAGAATGGCAGCGCAATGACATTCTTGCAGGTATCTTCGAGCCAG
CCACGATCGACATTGATCTGGCTATCTTGCTGACAAAAGCAAGAGAACATAGCGTTGCCTTGGTAGGTCC
AGCGGCGGAGGAACTCTTTGATCCGGTTCCTGAACAGGATCTATTTGAGGCGCTAAATGAAACCTTAACG
CTATGGAACTCGCCGCCCGACTGGGCTGGCGATGAGCGAAATGTAGTGCTTACGTTGTCCCGCATTTGGT
ACAGCGCAGTAACCGGCAAAATCGCGCCGAAGGATGTCGCTGCCGACTGGGCAATGGAGCGCCTGCCGGC
CCAGTATCAGCCCGTCATACTTGAAGCTAGACAGGCTTATCTTGGACAAGAAGAAGATCGCTTGGCCTCG
CGCGCAGATCAGTTGGAAGAATTTGTCCACTACGTGAAAGGCGAGATCACCAAGGTAGTCGGCAAATAAT
GTCTAGCTAGAAATTCGTTCAAGCCGACGCCGCTTCGCCGGCGTTAACTCAAGCGATTAGATGCACTAAG
CACATAATTGCTCACAGCCAAACTATCAGGTCAAGTCTGCTTTTATTATTTTTAAGCGTGCATAATAAGC
CCTACACAAATTGGGAGATATATCATGCATGACCAAAATCCCTTAACGTGAGTTTTCGTTCCACTGAGCG
TCAGACCCCGTAGAAAAGATCAAAGGATCTTCTTGAGATCCTTTTTTTCTGCGCGTAATCTGCTGCTTGC
AAACAAAAAAACCACCGCTACCAGCGGTGGTTTGTTTGCCGGATCAAGAGCTACCAACTCTTTTTCCGAA
GGTAACTGGCTTCAGCAGAGCGCAGATACCAAATACTGTCCTTCTAGTGTAGCCGTAGTTAGGCCACCAC
TTCAAGAACTCTGTAGCACCGCCTACATACCTCGCTCTGCTAATCCTGTTACCAGTGGCTGCTGCCAGTG
GCGATAAGTCGTGTCTTACCGGGTTGGACTCAAGACGATAGTTACCGGATAAGGCGCAGCGGTCGGGCTG
AACGGGGGGTTCGTGCACACAGCCCAGCTTGGAGCGAACGACCTACACCGAACTGAGATACCTACAGCGT
GAGCTATGAGAAAGCGCCACGCTTCCCGAAGGGAGAAAGGCGGACAGGTATCCGGTAAGCGGCAGGGTCG
GAACAGGAGAGCGCACGAGGGAGCTTCCAGGGGGAAACGCCTGGTATCTTTATAGTCCTGTCGGGTTTCG
CCACCTCTGACTTGAGCGTCGATTTTTGTGATGCTCGTCAGGGGGGCGGAGCCTATGGAAAAACGCCAGC
AACGCGGCCTTTTTACGGTTCCTGGCCTTTTGCTGGCCTTTTGCTCACATGTTCTTTCCTGCGTTATCCC
CTGATTCTGTGGATAACCGTATTACCGCCTTTGAGTGAGCTGATACCGCTCGCCGCAGCCGAACGACCGA

GCGCAGCGAGTCAGTGAGCGAGGAAGCGGAAGAGCGCCTGATGCGGTATTTTCTCCTTACGCATCTGTGC
GGTATTTCACACCGCATATGGTGCACTCTCAGTACAATCTGCTCTGATGCCGCATAGTTAAGCCAGTATA
CACTCCGCTATCGCTACGTGACTGGGTCATGGCTGCGCCCCGACACCCGCCAACACCCGCTGACGCGCCC
TGACGGGCTTGTCTGCTCCCGGCATCCGCTTACAGACAAGCTGTGACCGTCTCCGGGAGCTGCATGTGTC
AGAGGTTTTCACCGTCATCACCGAAACGCGCGAGGCAGGGTGCCTTGATGTGGGCGCCGGCGGTCGAGTG
GCGACGGCGCGGCTTGTCCGCGCCCTGGTAGATTGCCTGGCCGTAGGCCAGCCATTTTTGAGCGGCCAGC
GGCCGCGATAGGCCGACGCGAAGCGGCGGGGCGTAGGGAGCGCAGCGACCGAAGGGTAGGCGCTTTTTGC
AGCTCTTCGGCTGTGCGCTGGCCAGACAGTTATGCACAGGCCAGGCGGGTTTTAAGAGTTTTAATAAGTT
TTAAAGAGTTTTAGGCGGAAAAATCGCCTTTTTTCTCTTTTATATCAGTCACTTACATGTGTGACCGGTT
CCCAATGTACGGCTTTGGGTTCCCAATGTACGGGTTCCGGTTCCCAATGTACGGCTTTGGGTTCCCAATG
TACGTGCTATCCACAGGAAAGAGACCTTTTCGACCTTTTTCCCCTGCTAGGGCAATTTGCCCTAGCATCT
GCTCCGTACATTAGGAACCGGCGGATGCTTCGCCCTCGATCAGGTTGCGGTAGCGCATGACTAGGATCGG
GCCAGCCTGCCCCGCCTCCTCCTTCAAATCGTACTCCGGCAGGTCATTTGACCCGATCAGCTTGCGCACG
GTGAAACAGAACTTCTTGAACTCTCCGGCGCTGCCACTGCGTTCGTAGATCGTCTTGAACAACCATCTGG
CTTCTGCCTTGCCTGCGGCGCGGCGTGCCAGGCGGTAGAGAAAACGGCCGATGCCGGGATCGATCAAAAA
GTAATCGGGGTGAACCGTCAGCACGTCCGGGTTCTTGCCTTCTGTGATCTCGCGGTACATCCAATCAGCT
AGCTCGATCTCGATGTACTCCGGCCGCCCGGTTTCGCTCTTTACGATCTTGTAGCGGCTAATCAAGGCTT
CACCCTCGGATACCGTCACCAGGCGGCCGTTCTTGGCCTTCTTCGTACGCTGCATGGCAACGTGCGTGGT
GTTTAACCGAATGCAGGTTTCTACCAGGTCGTCTTTCTGCTTTCCGCCATCGGCTCGCCGGCAGAACTTG
AGTACGTCCGCAACGTGTGGACGGAACACGCGGCCGGGCTTGTCTCCCTTCCCTTCCCGGTATCGGTTCA
TGGATTCGGTTAGATGGGAAACCGCCATCAGTACCAGGTCGTAATCCCACACACTGGCCATGCCGGCCGG
CCCTGCGGAAACCTCTACGTGCCCGTCTGGAAGCTCGTAGCGGATCACCTCGCCAGCTCGTCGGTCACGC
TTCGACAGACGGAAAACGGCCACGTCCATGATGCTGCGACTATCGCGGGTGCCCACGTCATAGAGCATCG
GAACGAAAAAATCTGGTTGCTCGTCGCCCTTGGGCGGCTTCCTAATCGACGGCGCACCGGCTGCCGGCGG
TTGCCGGGATTCTTTGCGGATTCGATCAGCGGCCGCTTGCCACGATTCACCGGGGCGTGCTTCTGCCTCG
ATGCGTTGCCGCTGGGCGGCCTGCGCGGCCTTCAACTTCTCCACCAGGTCATCACCCAGCGCCGCGCCGA
TTTGTACCGGGCCGGATGGTTTGCGACCGTCACGCCGATTCCTCGGGCTTGGGGGTTCCAGTGCCATTGC
AGGGCCGGCAGACAACCCAGCCGCTTACGCCTGGCCAACCGCCCGTTCCTCCACACATGGGGCATTCCAC
GGCGTCGGTGCCTGGTTGTTCTTGATTTTCCATGCCGCCTCCTTTAGCCGCTAAAATTCATCTACTCATT
TATTCATTTGCTCATTTACTCTGGTAGCTGCGCGATGTATTCAGATAGCAGCTCGGTAATGGTCTTGCCT
TGGCGTACCGCGTACATCTTCAGCTTGGTGTGATCCTCCGCCGGCAACTGAAAGTTGACCCGCTTCATGG
CTGGCGTGTCTGCCAGGCTGGCCAACGTTGCAGCCTTGCTGCTGCGTGCGCTCGGACGGCCGGCACTTAG
CGTGTTTGTGCTTTTGCTCATTTTCTCTTTACCTCATTAACTCAAATGAGTTTTGATTTAATTTCAGCGG
CCAGCGCCTGGACCTCGCGGGCAGCGTCGCCCTCGGGTTCTGATTCAAGAACGGTTGTGCCGGCGGCGGC
AGTGCCTGGGTAGCTCACGCGCTGCGTGATACGGGACTCAAGAATGGGCAGCTCGTACCCGGCCAGCGCC
TCGGCAACCTCACCGCCGATGCGCGTGCCTTTGATCGCCCGCGACACGACAAAGGCCGCTTGTAGCCTTC
CATCCGTGACCTCAATGCGCTGCTTAACCAGCTCCACCAGGTCGGCGGTGGCCCATATGTCGTAAGGGCT
TGGCTGCACCGGAATCAGCACGAAGTCGGCTGCCTTGATCGCGGACACAGCCAAGTCCGCCGCCTGGGGC
GCTCCGTCGATCACTACGAAGTCGCGCCGGCCGATGGCCTTCACGTCGCGGTCAATCGTCGGGCGGTCGA
TGCCGACAACGGTTAGCGGTTGATCTTCCCGCACGGCCGCCCAATCGCGGGCACTGCCCTGGGGATCGGA
ATCGACTAACAGAACATCGGCCCCGGCGAGTTGCAGGGCGCGGGCTAGATGGGTTGCGATGGTCGTCTTG
CCTGACCCGCCTTTCTGGTTAAGTACAGCGATAACCTTCATGCGTTCCCCTTGCGTATTTGTTTATTTAC
TCATCGCATCATATACGCAGCGACCGCATGACGCAAGCTGTTTTACTCAAATACACATCACCTTTTTAGA
CGGCGGCGCTCGGTTTCTTCAGCGGCCAAGCTGGCCGGCCAGGCCGCCAGCTTGGCATCAGACAAACCGG

CCAGGATTTCATGCAGCCGCACGGTTGAGACGTGCGCGGGCGGCTCGAACACGTACCCGGCCGCGATCAT
CTCCGCCTCGATCTCTTCGGTAATGAAAAACGGTTCGTCCTGGCCGTCCTGGTGCGGTTTCATGCTTGTT
CCTCTTGGCGTTCATTCTCGGCGGCCGCCAGGGCGTCGGCCTCGGTCAATGCGTCCTCACGGAAGGCACC
GCGCCGCCTGGCCTCGGTGGGCGTCACTTCCTCGCTGCGCTCAAGTGCGCGGTACAGGGTCGAGCGATGC
ACGCCAAGCAGTGCAGCCGCCTCTTTCACGGTGCGGCCTTCCTGGTCGATCAGCTCGCGGGCGTGCGCGA
TCTGTGCCGGGGTGAGGGTAGGGCGGGGGCCAAACTTCACGCCTCGGGCCTTGGCGGCCTCGCGCCCGCT
CCGGGTGCGGTCGATGATTAGGGAACGCTCGAACTCGGCAATGCCGGCGAACACGGTCAACACCATGCGG
CCGGCCGGCGTGGTGGTGTCGGCCCACGGCTCTGCCAGGCTACGCAGGCCCGCGCCGGCCTCCTGGATGC
GCTCGGCAATGTCCAGTAGGTCGCGGGTGCTGCGGGCCAGGCGGTCTAGCCTGGTCACTGTCACAACGTC
GCCAGGGCGTAGGTGGTCAAGCATCCTGGCCAGCTCCGGGCGGTCGCGCCTGGTGCCGGTGATCTTCTCG
GAAAACAGCTTGGTGCAGCCGGCCGCGTGCAGTTCGGCCCGTTGGTTGGTCAAGTCCTGGTCGTCGGTGC
TGACGCGGGCATAGCCCAGCAGGCCAGCGGCGGCGCTCTTGTTCATGGCGTAATGTCTCCGGTTCTAGTC
GCAAGTATTCTACTTTATGCGACTAAAACACGCGACAAGAAAACGCCAGGAAAAGGGCAGGGCGGCAGCC
TGTCGCGTAACTTAGGACTTGTGCGACATGTCGTTTTCAGAAGACGGCTGCACTGAACGTCAGAAGCCGA
CTGCACTATAGCAGCGGAGGGGTTGGATCAAAGTACTTTGATCCCGAGGGGAACCCTGTGGTTGGCATGC
ACATACAAATGGACGAACGGATAAACCTTTTCACGCCCTTTTAAATATCCGTTATTCTAATAAACGCTCT
TTTCTCTTAGGTTTACCCGCCAATATATCCTGTCAAACACTGATAGTTTAAACTGAAGGCGGGAAACGAC
AATCTGATCCAAGCTCAAGCTAAGCTTGAGCTCTGCATGCCTGCAGGTCCCCAGATTAGCCTTTTCAATT
TCAGAAAGAATGCTAACCCACAGATGGTTAGAGAGGCTTACGCAGCAGGTCTCATCAAGACGATCTACCC
GAGCAATAATCTCCAGGAAATCAAATACCTTCCCAAGAAGGTTAAAGATGCAGTCAAAAGATTCAGGACT
AACTGCATCAAGAACACAGAGAAAGATATATTTCTCAAGATCAGAAGTACTATTCCAGTATGGACGATTC
AAGGCTTGCTTCACAAACCAAGGCAAGTAATAGAGATTGGAGTCTCTAAAAAGGTAGTTCCCACTGAATC
AAAGGCCATGGAGTCAAAGATTCAAATAGAGGACCTAACAGAACTCGCCGTAAAGACTGGCGAACAGTTC
ATACAGAGTCTCTTACGACTCAATGACAAGAAGAAAATCTTCGTCAACATGGTGGAGCACGACACACTTG
TCTACTCCAAAAATATCAAAGATACAGTCTCAGAAGACCAAAGGGCAATTGAGACTTTTCAACAAAGGGT
AATATCCGGAAACCTCCTCGGATTCCATTGCCCAGCTATCTGTCACTTTATTGTGAAGATAGTGGAAAAG
GAAGGTGGCTCCTACAAATGCCATCATTGCGATAAAGGAAAGGCCATCGTTGAAGATGCCTCTGCCGACA
GTGGTCCCAAAGATGGACCCCCACCCACGAGGAGCATCGTGGAAAAAGAAGACGTTCCAACCACGTCTTC
AAAGCAAGTGGATTGATGTGATATCTCCACTGACGTAAGGGATGACGCACAATCCCACTATCCTTCGCAA
GACCCTTCCTCTATATAAGGAAGTTCATTTCATTTGGAGAGAACACGGGGGACTCTAGAGGATCCAAGGA
GATATAAGCCGCCACCATGGATTACAAGGACGATGATGACAAGGACTACAAGGACGATGACGATAAGGAC
TATAAGGATGACGATGACAAGGATTACGCCGAGGTCATGGAACGTGCGATCCAGGGCAATGATGCGCGTG
AACAGGCGAACAGCGAACGTTGGGATGGCGGCTCTGGCGGCACGACTAGCCCGTTCAAACTGCCGGATGA
AAGCCCGTCCTGGACCGAATGGCGTCTGCACAACGACGAGACGAACAGCAACCAGGATAACCCGCTGGGT
TTCAAAGAAAGCTGGGGCTTTGGCAAAGTGGTGTTCAAACGTTACCTGCGTTATGACCGCACCGAAGCGT
CACTGCACCGTGTGCTGGGTTCTTGGACGGGCGACAGCGTGAACTACGCGGCGAGCCGTTTCTTCGGCTT
CGACCAGATCGGTTGCACCTACAGCATCCGTTTCCGTGGTGTGAGCATCACCGTGAGCGGTGGTAGCCGT
ACCCTGCAGCACCTGTGCGAAATGGCGATCCGTAGCAAACAGGAACTCCTTCAACTGGCGCCGATCGAAG
TGGAATCTAACGTGTCGCGTGGCTGTCCGGAAGGTACCGAAACCTTCGAAAAAGAAAGCGAACCTCTTCC
CTAATGATATCCCGCGGCCATGCTAGAGTCCGCAAAAATCACCAGTCTCTCTCTACAAATCTATCTCTCT
CTATTTTTCTCCAGAATAATGTGTGAGTAGTTCCCAGATAAGGGAATTAGGGTTCTTATAGGGTTTCGCT
CATGTGTTGAGCATATAAGAAACCCTTAGTATGTATTTGTATTTGTAAAATACTTCTATCAATAAAATTT
CTAATTCCTAAAACCAAAATCCAGTGACCTGCAGGCATGCGACGTCG

Sequence of pMS4 (35S-GFP)

GGCCGCACTAGTGATATCCCGCGGCCATGGCGGCCGGGAGCATGCGACGTCGGGCCCAATTCGCCCTATA
GTGAGTCGTATTACAATTCACTGGCCGTCGTTTTACAACGTCGTGACTGGGAAAACCCTGGCGTTACCCA
ACTTAATCGCCTTGCAGCACATCCCCCTTTCGCCAGCTGGCGTAATAGCGAAGAGGCCCGCACCGATCGC
CCTTCCCAACAGTTGCGCAGCCTGAATGGCGAATGGAAATTGTAAACGTTAATGGGTTTCTGGAGTTTAA
TGAGCTAAGCACATACGTCAGAAACCATTATTGCGCGTTCAAAAGTCGCCTAAGGTCACTATCAGCTAGC
AAATATTTCTTGTCAAAAATGCTCCACTGACGTTCCATAAATTCCCCTCGGTATCCAATTAGAGTCTCAT
ATTCACTCTCAATCCAAATAATCTGCAATGGCAATTACCTTATCCGCAACTTCTTTACCTATTTCCGCCC
GGATCCGGGCAGGTTCTCCGGCCGCTTGGGTGGAGAGGCTATTCGGCTATGACTGGGCACAACAGACAAT
CGGCTGCTCTGATGCCGCCGTGTTCCGGCTGTCAGCGCAGGGGCGCCCGGTTCTTTTTGTCAAGACCGAC
CTGTCCGGTGCCCTGAATGAACTGCAGGACGAGGCAGCGCGGCTATCGTGGCTGGCCACGACGGGCGTTC
CTTGCGCAGCTGTGCTCGACGTTGTCACTGAAGCGGGAAGGGACTGGCTGCTATTGGGCGAAGTGCCGGG
GCAGGATCTCCTGTCATCTCACCTTGCTCCTGCCGAGAAAGTATCCATCATGGCTGATGCAATGCGGCGG
CTGCATACGCTTGATCCGGCTACCTGCCCATTCGACCACCAAGCGAAACATCGCATCGAGCGAGCACGTA
CTCGGATGGAAGCCGGTCTTGTCGATCAGGATGATCTGGACGAAGAGCATCAGGGGCTCGCGCCAGCCGA
ACTGTTCGCCAGGCTCAAGGCGCGCATGCCCGACGGCGAGGATCTCGTCGTGACCCATGGCGATGCCTGC
TTGCCGAATATCATGGTGGAAAATGGCCGCTTTTCTGGATTCATCGACTGTGGCCGGCTGGGTGTGGCGG
ACCGCTATCAGGACATAGCGTTGGCTACCCGTGATATTGCTGAAGAGCTTGGCGGCGAATGGGCTGACCG
CTTCCTCGTGCTTTACGGTATCGCCGCTCCCGATTCGCAGCGCATCGCCTTCTATCGCCTTCTTGACGAG
TTCTTCTGAGCGGGACTCTGGGGTTCGAAATGACCGACCAAGCGACGCCCAACCTGCCATCACGAGATTT
CGATTCCACCGCCGCCTTCTATGAAAGGTTGGGCTTCGGAATCGTTTTCCGGGACGCCGGCTGGATGATC
CTCCAGCGCGGGGATCTCATGCTGGAGTTCTTCGCCCACCCCGATCCAACACTTACGTTTGCAACGTCCA
AGAGCAAATAGACCACGAACGCCGGAAGGTTGCCGCAGCGTGTGGATTGCGTCTCAATTCTCTCTTGCAG
GAATGCAATGATGAATATGATACTGACTATGAAACTTTGAGGGAATACTGCCTAGCACCGTCACCTCATA
ACGTGCATCATGCATGCCCTGACAACATGGAACATCGCTATTTTTCTGAAGAATTATGCTCGTTGGAGGA
TGTCGCGGCAATTGCAGCTATTGCCAACATCGAACTACCCCTCACGCATGCATTCATCAATATTATTCAT
GCGGGGAAAGGCAAGATTAATCCAACTGGCAAATCATCCAGCGTGATTGGTAACTTCAGTTCCAGCGACT
TGATTCGTTTTGGTGCTACCCACGTTTTCAATAAGGACGAGATGGTGGAGTAAAGAAGGAGTGCGTCGAA
GCAGATCGTTCAAACATTTGGCAATAAAGTTTCTTAAGATTGAATCCTGTTGCCGGTCTTGCGATGATTA
TCATATAATTTCTGTTGAATTACGTTAAGCATGTAATAATTAACATGTAATGCATGACGTTATTTATGAG
ATGGGTTTTTATGATTAGAGTCCCGCAATTATACATTTAATACGCGATAGAAAACAAAATATAGCGCGCA
AACTAGGATAAATTATCGCGCGCGGTGTCATCTATGTTACTAGATCGAATTAATTCCAGGCGGTGAAGGG
CAATCAGCTGTTGCCCGTCTCACTGGTGAAAAGAAAAACCACCCCAGTACATTAAAAACGTCCGCAATGT
GTTATTAAGTTGTCTAAGCGTCAATTTGTTTACACCACAATATATCCTGCCACCAGCCAGCCAACAGCTC
CCCGACCGGCAGCTCGGCACAAAATCACCACTCGATACAGGCAGCCCATCAGTCCGGGACGGCGTCAGCG
GGAGAGCCGTTGTAAGGCGGCAGACTTTGCTCATGTTACCGATGCTATTCGGAAGAACGGCAACTAAGCT
GCCGGGTTTGAAACACGGATGATCTCGCGGAGGGTAGCATGTTGATTGTAACGATGACAGAGCGTTGCTG
CCTGTGATCAAATATCATCTCCCTCGCAGAGATCCGAATTATCAGCCTTCTTATTCATTTCTCGCTTAAC
CGTGACAGGCTGTCGATCTTGAGAACTATGCCGACATAATAGGAAATCGCTGGATAAAGCCGCTGAGGAA
GCTGAGTGGCGCTATTTCTTTAGAAGTGAACGTTGACGATGTCGACGGATCTTTTCCGCTGCATAACCCT
GCTTCGGGGTCATTATAGCGATTTTTTCGGTATATCCATCCTTTTTCGCACGATATACAGGATTTTGCCA
AAGGGTTCGTGTAGACTTTCCTTGGTGTATCCAACGGCGTCAGCCGGGCAGGATAGGTGAAGTAGGCCCA
CCCGCGAGCGGGTGTTCCTTCTTCACTGTCCCTTATTCGCACCTGGCGGTGCTCAACGGGAATCCTGCTC
TGCGAGGCTGGCCGGCTACCGCCGGCGTAACAGATGAGGGCAAGCGGATGGCTGATGAAACCAAGCCAAC

CAGGGGTGATGCTGCCAACTTACTGATTTAGTGTATGATGGTGTTTTTGAGGTGCTCCAGTGGCTTCTGT
TTCTATCAGCTGTCCCTCCTGTTCAGCTACTGACGGGGTGGTGCGTAACGGCAAAAGCACCGCCGGACAT
CAGCGCTATCTCTGCTCTCACTGCCGTAAAACATGGCAACTGCAGTTCACTTACACCGCTTCTCAACCCG
GTACGCACCAGAAAATCATTGATATGGCCATGAATGGCGTTGGATGCCGGGCAACAGCCCGCATTATGGG
CGTTGGCCTCAACACGATTTTACGTCACTTAAAAAACTCAGGCCGCAGTCGGTAACCTCGCGCATACAGC
CGGGCAGTGACGTCATCGTCTGCGCGGAAATGGACGAACAGTGGGGCTATGTCGGGGCTAAATCGCGCCA
GCGCTGGCTGTTTTACGCGTATGACAGTCTCCGGAAGACGGTTGTTGCGCACGTATTCGGTGAACGCACT
ATGGCGACGCTGGGGCGTCTTATGAGCCTGCTGTCACCCTTTGACGTGGTGATATGGATGACGGATGGCT
GGCCGCTGTATGAATCCCGCCTGAAGGGAAAGCTGCACGTAATCAGCAAGCGATATACGCAGCGAATTGA
GCGGCATAACCTGAATCTGAGGCAGCACCTGGCACGGCTGGGACGGAAGTCGCTGTCGTTCTCAAAATCG
GTGGAGCTGCATGACAAAGTCATCGGGCATTATCTGAACATAAAACACTATCAATAAGTTGGAGTCATTA
CCCAACCAGGAAGGGCAGCCCACCTATCAAGGTGTACTGCCTTCCAGACGAACGAAGAGCGATTGAGGAA
AAGGCGGCGGCGGCCGGCATGAGCCTGTCGGCCTACCTGCTGGCCGTCGGCCAGGGCTACAAAATCACGG
GCGTCGTGGACTATGAGCACGTCCGCGAGCTGGCCCGCATCAATGGCGACCTGGGCCGCCTGGGCGGCCT
GCTGAAACTCTGGCTCACCGACGACCCGCGCACGGCGCGGTTCGGTGATGCCACGATCCTCGCCCTGCTG
GCGAAGATCGAAGAGAAGCAGGACGAGCTTGGCAAGGTCATGATGGGCGTGGTCCGCCCGAGGGCAGAGC
CATGACTTTTTTAGCCGCTAAAACGGCCGGGGGGTGCGCGTGATTGCCAAGCACGTCCCCATGCGCTCCA
TCAAGAAGAGCGACTTCGCGGAGCTGGTATTCGTGCAGGGCAAGATTCGGAATACCAAGTACGAGAAGGA
CGGCCAGACGGTCTACGGGACCGACTTCATTGCCGATAAGGTGGATTATCTGGACACCAAGGCACCAGGC
GGGTCAAATCAGGAATAAGGGCACATTGCCCCGGCGTGAGTCGGGGCAATCCCGCAAGGAGGGTGAATGA
ATCGGACGTTTGACCGGAAGGCATACAGGCAAGAACTGATCGACGCGGGGTTTTCCGCCGAGGATGCCGA
AACCATCGCAAGCCGCACCGTCATGCGTGCGCCCCGCGAAACCTTCCAGTCCGTCGGCTCGATGGTCCAG
CAAGCTACGGCCAAGATCGAGCGCGACAGCGTGCAACTGGCTCCCCCTGCCCTGCCCGCGCCATCGGCCG
CCGTGGAGCGTTCGCGTCGTCTCGAACAGGAGGCGGCAGGTTTGGCGAAGTCGATGACCATCGACACGCG
AGGAACTATGACGACCAAGAAGCGAAAAACCGCCGGCGAGGACCTGGCAAAACAGGTCAGCGAGGCCAAG
CAGGCCGCGTTGCTGAAACACACGAAGCAGCAGATCAAGGAAATGCAGCTTTCCTTGTTCGATATTGCGC
CGTGGCCGGACACGATGCGAGCGATGCCAAACGACACGGCCCGCTCTGCCCTGTTCACCACGCGCAACAA
GAAAATCCCGCGCGAGGCGCTGCAAAACAAGGTCATTTTCCACGTCAACAAGGACGTGAAGATCACCTAC
ACCGGCGTCGAGCTGCGGGCCGACGATGACGAACTGGTGTGGCAGCAGGTGTTGGAGTACGCGAAGCGCA
CCCCTATCGGCGAGCCGATCACCTTCACGTTCTACGAGCTTTGCCAGGACCTGGGCTGGTCGATCAATGG
CCGGTATTACACGAAGGCCGAGGAATGCCTGTCGCGCCTACAGGCGACGGCGATGGGCTTCACGTCCGAC
CGCGTTGGGCACCTGGAATCGGTGTCGCTGCTGCACCGCTTCCGCGTCCTGGACCGTGGCAAGAAAACGT
CCCGTTGCCAGGTCCTGATCGACGAGGAAATCGTCGTGCTGTTTGCTGGCGACCACTACACGAAATTCAT
ATGGGAGAAGTACCGCAAGCTGTCGCCGACGGCCCGACGGATGTTCGACTATTTCAGCTCGCACCGGGAG
CCGTACCCGCTCAAGCTGGAAACCTTCCGCCTCATGTGCGGATCGGATTCCACCCGCGTGAAGAAGTGGC
GCGAGCAGGTCGGCGAAGCCTGCGAAGAGTTGCGAGGCAGCGGCCTGGTGGAACACGCCTGGGTCAATGA
TGACCTGGTGCATTGCAAACGCTAGGGCCTTGTGGGGTCAGTTCCGGCTGGGGGTTCAGCAGCCAGCGCT
TTACTGGCATTTCAGGAACAAGCGGGCACTGCTCGACGCACTTGCTTCGCTCAGTATCGCTCGGGACGCA
CGGCGCGCTCTACGAACTGCCGATAAACAGAGGATTAAAATTGACAATTGTGATTAAGGCTCAGATTCGA
CGGCTTGGAGCGGCCGACGTGCAGGATTTCCGCGAGATCCGATTGTCGGCCCTGAAGAAAGCTCCAGAGA
TGTTCGGGTCCGTTTACGAGCACGAGGAGAAAAAGCCCATGGAGGCGTTCGCTGAACGGTTGCGAGATGC
CGTGGCATTCGGCGCCTACATCGACGGCGAGATCATTGGGCTGTCGGTCTTCAAACAGGAGGACGGCCCC
AAGGACGCTCACAAGGCGCATCTGTCCGGCGTTTTCGTGGAGCCCGAACAGCGAGGCCGAGGGGTCGCCG
GTATGCTGCTGCGGGCGTTGCCGGCGGGTTTATTGCTCGTGATGATCGTCCGACAGATTCCAACGGGAAT

CTGGTGGATGCGCATCTTCATCCTCGGCGCACTTAATATTTCGCTATTCTGGAGCTTGTTGTTTATTTCG
GTCTACCGCCTGCCGGGCGGGGTCGCGGCGACGGTAGGCGCTGTGCAGCCGCTGATGGTCGTGTTCATCT
CTGCCGCTCTGCTAGGTAGCCCGATACGATTGATGGCGGTCCTGGGGGCTATTTGCGGAACTGCGGGCGT
GGCGCTGTTGGTGTTGACACCAAACGCAGCGCTAGATCCTGTCGGCGTCGCAGCGGGCCTGGCGGGGGCG
GTTTCCATGGCGTTCGGAACCGTGCTGACCCGCAAGTGGCAACCTCCCGTGCCTCTGCTCACCTTTACCG
CCTGGCAACTGGCGGCCGGAGGACTTCTGCTCGTTCCAGTAGCTTTAGTGTTTGATCCGCCAATCCCGAT
GCCTACAGGAACCAATGTTCTCGGCCTGGCGTGGCTCGGCCTGATCGGAGCGGGTTTAACCTACTTCCTT
TGGTTCCGGGGGATCTCGCGACTCGAACCTACAGTTGTTTCCTTACTGGGCTTTCTCAGCCGGGATGGCG
CTAAGAAGCTATTGCCGCCGATCTTCATATGCGGTGTGAAATACCGCACAGATGCGTAAGGAGAAAATAC
CGCATCAGGCGCTCTTCCGCTTCCTCGCTCACTGACTCGCTGCGCTCGGTCGTTCGGCTGCGGCGAGCGG
TATCAGCTCACTCAAAGGCGGTAATACGGTTATCCACAGAATCAGGGGATAACGCAGGAAAGAACATGTG
AGCAAAAGGCCAGCAAAAGGCCAGGAACCGTAAAAAGGCCGCGTTGCTGGCGTTTTTCCATAGGCTCCGC
CCCCCTGACGAGCATCACAAAAATCGACGCTCAAGTCAGAGGTGGCGAAACCCGACAGGACTATAAAGAT
ACCAGGCGTTTCCCCCTGGAAGCTCCCTCGTGCGCTCTCCTGTTCCGACCCTGCCGCTTACCGGATACCT
GTCCGCCTTTCTCCCTTCGGGAAGCGTGGCGCTTTCTCAATGCTCACGCTGTAGGTATCTCAGTTCGGTG
TAGGTCGTTCGCTCCAAGCTGGGCTGTGTGCACGAACCCCCCGTTCAGCCCGACCGCTGCGCCTTATCCG
GTAACTATCGTCTTGAGTCCAACCCGGTAAGACACGACTTATCGCCACTGGCAGCAGCCACTGGTAACAG
GATTAGCAGAGCGAGGTATGTAGGCGGTGCTACAGAGTTCTTGAAGTGGTGGCCTAACTACGGCTACACT
AGAAGGACAGTATTTGGTATCTGCGCTCTGCTGAAGCCAGTTACCTTCGGAAAAAGAGTTGGTAGCTCTT
GATCCGGCAAACAAACCACCGCTGGTAGCGGTGGTTTTTTTGTTTGCAAGCAGCAGATTACGCGCAGAAA
AAAAGGATATCAAGAAGATCCTTTGATCTTTTCTACGGGGTCTGACGCTCAGTGGAACGAAAACTCACGT
TAAGGGATTTTGGTCATGAGATTATCAAAAAGGATCTTCACCTAGATCCTTTTAAATTAAAAATGAAGTT
TTAAATCAATCTAAAGTATATATGAGTAAACTTGGTCTGACAGTTACCAATGCTTAATCAGTGAGGCACC
TATCTCAGCGATCTGTCTATTTCGTTCATCCATAGTTGCCTGACTCCCCGTCGTGTAGATAACTACGATA
CGGGAGGGCTTACCATCTGGCCCCAGTGCTGCAATGATACCGCGAGACCCACGCTCACCGGCTCCAGATT
TATCAGCAATAAACCAGCCAGCCGGAAGGGCCGAGCGCAGAAGTGGTCCTGCAACTTTATCCGCCTCCAT
CCAGTCTATTAAACAAGTGGCAGCAACGGATTCGCAAACCTGTCACGCCTTTTGTGCCAAAAGCCGCGCC
AGGTTTGCGATCCGCTGTGCCAGGCGTTAGGCGTCATATGAAGATTTCGGTGATCCCTGAGCAGGTGGCG
GAAACATTGGATGCTGAGAACCATTTCATTGTTCGTGAAGTGTTCGATGTGCACCTATCCGACCAAGGCT
TTGAACTATCTACCAGAAGTGTGAGCCCCTACCGGAAGGATTACATCTCGGATGATGACTCTGATGAAGA
CTCTGCTTGCTATGGCGCATTCATCGACCAAGAGCTTGTCGGGAAGATTGAACTCAACTCAACATGGAAC
GATCTAGCCTCTATCGAACACATTGTTGTGTCGCACACGCACCGAGGCAAAGGAGTCGCGCACAGTCTCA
TCGAATTTGCGAAAAAGTGGGCACTAAGCAGACAGCTCCTTGGCATACGATTAGAGACACAAACGAACAA
TGTACCTGCCTGCAATTTGTACGCAAAATGTGGCTTTACTCTCGGCGGCATTGACCTGTTCACGTATAAA
ACTAGACCTCAAGTCTCGAACGAAACAGCGATGTACTGGTACTGGTTCTCGGGAGCACAGGATGACGCCT
AACAATTCATTCAAGCCGACACCGCTTCGCGGCGCGGCTTAATTCAGGAGTTAAACATCATGAGGGAAGC
GGTGATCGCCGAAGTATCGACTCAACTATCAGAGGTAGTTGGCGTCATCGAGCGCCATCTCGAACCGACG
TTGCTGGCCGTACATTTGTACGGCTCCGCAGTGGATGGCGGCCTGAAGCCACACAGTGATATTGATTTGC
TGGTTACGGTGACCGTAAGGCTTGATGAAACAACGCGGCGAGCTTTGATCAACGACCTTTTGGAAACTTC
GGCTTCCCCTGGAGAGAGCGAGATTCTCCGCGCTGTAGAAGTCACCATTGTTGTGCACGACGACATCATT
CCGTGGCGTTATCCAGCTAAGCGCGAACTGCAATTTGGAGAATGGCAGCGCAATGACATTCTTGCAGGTA
TCTTCGAGCCAGCCACGATCGACATTGATCTGGCTATCTTGCTGACAAAAGCAAGAGAACATAGCGTTGC
CTTGGTAGGTCCAGCGGCGGAGGAACTCTTTGATCCGGTTCCTGAACAGGATCTATTTGAGGCGCTAAAT
GAAACCTTAACGCTATGGAACTCGCCGCCCGACTGGGCTGGCGATGAGCGAAATGTAGTGCTTACGTTGT

CCCGCATTTGGTACAGCGCAGTAACCGGCAAAATCGCGCCGAAGGATGTCGCTGCCGACTGGGCAATGGA
GCGCCTGCCGGCCCAGTATCAGCCCGTCATACTTGAAGCTAGGCAGGCTTATCTTGGACAAGAAGATCGC
TTGGCCTCGCGCGCAGATCAGTTGGAAGAATTTGTTCACTACGTGAAAGGCGAGATCACCAAGGTAGTCG
GCAAATAATGTCTAACAATTCGTTCAAGCCGACGCCGCTTCGCGGCGCGGCTTAACTCAAGCGTTAGAGA
GCTGGGGAAGACTATGCGCGATCTGTTGAAGGTGGTTCTAAGCCTCGTACTTGCGATGGCATCGGGGCAG
GCACTTGCTGACCTGCCAATTGTTTTAGTGGATGAAGCTCGTCTTCCCTATGACTACTCCCCATCCAACT
ACGACATTTCTCCAAGCAACTACGACAACTCCATAAGCAATTACGACAATAGTCCATCAAATTACGACAA
CTCTGAGAGCAACTACGATAATAGTTCATCCAATTACGACAATAGTCGCAACGGAAATCGTAGGCTTATA
TATAGCGCAAATGGGTCTCGCACTTTCGCCGGCTACTACGTCATTGCCAACAATGGGACAACGAACTTCT
TTTCCACATCTGGCAAAAGGATGTTCTACACCCCAAAAGGGGGGCGCGGCGTCTATGGCGGCAAAGATGG
GAGCTTCTGCGGGGCATTGGTCGTCATAAATGGCCAATTTTCGCTTGCCCTGACAGATAACGGCCTGAAG
ATCATGTATCTAAGCAACTAGCCTGCTCTCTAATAAAATGTTAGGAGCTTGGCTGCCATTTTTGGGGTGA
GGCCGTTCGCGGCCGAGGGGCGCAGCCCCTGGGGGGATGGGAGGCCCGCGTTAGCGGGCCGGGAGGGTTC
GAGAAGGGGGGGCACCCCCCTTCGGCGTGCGCGGTCACGCGCCAGGGCGCAGCCCTGGTTAAAAACAAGG
TTTATAAATATTGGTTTAAAAGCAGGTTAAAAGACAGGTTAGCGGTGGCCGAAAAACGGGCGGAAACCCT
TGCAAATGCTGGATTTTCTGCCTGTGGACAGCCCCTCAAATGTCAATAGGTGCGCCCCTCATCTGTCAGC
ACTCTGCCCCTCAAGTGTCAAGGATCGCGCCCCTCATCTGTCAGTAGTCGCGCCCCTCAAGTGTCAATAC
CGCAGGGCACTTATCCCCAGGCTTGTCCACATCATCTGTGGGAAACTCGCGTAAAATCAGGCGTTTTCGC
CGATTTGCGAGGCTGGCCAGCTCCACGTCGCCGGCCGAAATCGAGCCTGCCCCTCATCTGTCAACGCCGC
GCCGGGTGAGTCGGCCCCTCAAGTGTCAACGTCCGCCCCTCATCTGTCAGTGAGGGCCAAGTTTTCCGCG
AGGTATCCACAACGCCGGCGGCCGGCCGCGGTGTCTCGCACACGGCTTCGACGGCGTTTCTGGCGCGTTT
GCAGGGCCATAGACGGCCGCCAGCCCAGCGGCGAGGGCAACCAGCCCGGTGAGCGTCGGAAAGGGTCGAC
ATCTTGCTGCGTTCGGATATTTTCGTGGAGTTCCCGCCACAGACCCGGATTGAAGGCGAGATCCAGCAAC
TCGCGCCAGATCATCCTGTGACGGAACTTTGGCGCGTGATGACTGGCCAGGACGTCGGCCGAAAGAGCGA
CAAGCAGATCACGATTTTCGACAGCGTCGGATTTGCGATCGAGGATTTTTCGGCGCTGCGCTACGTCCGC
GACCGCGTTGAGGGATCAAGCCACAGCAGCCCACTCGACCTTCTAGCCGACCCAGACGAGCCAAGGGATC
TTTTTGGAATGCTGCTCCGTCGTCAGGCTTTCCGACGTTTGGGTGGTTGAACAGAAGTCATTATCGTACG
GAATGCCAGCACTCCCGAGGGGAACCCTGTGGTTGGCATGCACATACAAATGGACGAACGGATAAACCTT
TTCACGCCCTTTTAAATATCCGTTATTCTAATAAACGCTCTTTTCTCTTAGGTTTACCCGCCAATATATC
CTGTCAAACACTGATAGTTTAAACTGAAGGCGGGAAACGACAATCTGATCATGAGCGGAGAATTAAGGGA
GTCACGTTATGACCCCCGCCGATGACGCGGGACAAGCCGTTTTACGTTTGGAACTGACAGAACCGCAACG
ATTGAAGGAGCCACTCAGCCCCAATACGCAAACCGCCTCTCCCCGCGCGTTGGCCGATTCATTAATGCAG
CTGGCACGACAGGTTTCCCGACTGGAAAGCGGGCAGTGAGCGCAACGCAATTAATGTGAGTTAGCTCACT
CATTAGGCACCCCAGGCTTTACACTTTATGCTTCCGGCTCGTATGTTGTGTGGAATTGTGAGCGGATAAC
AATTTCACACAGGAAACAGCTATGACCATGATTACGCCAAGCTATTTAGGTGACACTATAGAATACTCAA
GCTATGCATCCAACGCGTTGGGAGCTCTCCCATATCGACCTGCAGGCGGCCGCTCGACGAATTAATTCCA
ATCCCACAAAAATCTGAGCTTAACAGCACAGTTGCTCCTCTCAGAGCAGAATCGGGTATTCAACACCCTC
ATATCAACTACTACGTTGTGTATAACGGTCCACATGCCGGTATATACGATGACTGGGGTTGTACAAAGGC
GGCAACAAACGGCGTTCCCGGAGTTGCACACAAGAAATTTGCCACTATTACAGAGGCAAGAGCAGCAGCT
GACGCGTACACAACAAGTCAGCAAACAGACAGGTTGAACTTCATCCCCAAAGGAGAAGCTCAACTCAAGC
CCAAGAGCTTTGCTAAGGCCCTAACAAGCCCACCAAAGCAAAAAGCCCACTGGCTCACGCTAGGAACCAA
AAGGCCCAGCAGTGATCCAGCCCCAAAAGAGATCTCCTTTGCCCCGGAGATTACAATGGACGATTTCCTC
TATCTTTACGATCTAGGAAGGAAGTTCGAAGGTGAAGGTGACGACACTATGTTCACCACTGATAATGAGA
AGGTTAGCCTCTTCAATTTCAGAAAGAATGCTGACCCACAGATGGTTAGAGAGGCCTACGCAGCAGGTCT

CATCAAGACGATCTACCCGAGTAACAATCTCCAGGAGATCAAATACCTTCCCAAGAAGGTTAAAGATGCA
GTCAAAAGATTCAGGACTAATTGCATCAAGAACACAGAGAAAGACATATTTCTCAAGATCAGAAGTACTA
TTCCAGTATGGACGATTCAAGGCTTGCTTCATAAACCAAGGCAAGTAATAGAGATTGGAGTCTCTAAAAA
GGTAGTTCCTACTGAATCTAAGGCCATGCATGGAGTCTAAGATTCAAATCGAGGATCTAACAGAACTCGC
CGTGAAGACTGGCGAACAGTTCATACAGAGTCTTTTACGACTCAATGACAAGAAGAAAATCTTCGTCAAC
ATGGTGGAGCACGACACTCTGGTCTACTCCAAAAATGTCAAAGATACAGTCTCAGAAGACCAAAGGGCTA
TTGAGACTTTTCAACAAAGGATAATTTCGGGAAACCTCCTCGGATTCCATTGCCCAGCTATCTGTCACTT
CATCGAAAGGACAGTAGAAAAGGAAGGTGGCTCCTACAAATGCCATCATTGCGATAAAGGAAAGGCTATC
ATTCAAGATCTCTCTGCCGACAGTGGTCCCAAAGATGGACCCCCACCCACGAGGAGCATCGTGGAAAAAG
AAGACGTTCCAACCACGTCTTCAAAGCAAGTGGATTGATGTGACATCTCCACTGACGTAAGGGATGACGC
ACAATCCCACTATCCTTCGCAAGACCCTTCCTCTATATAAGGAAGTTCATTTCATTTGGAGAGGACACGC
TCGACTATTTTTACAACAATTACCATGAAGACTAATCTTTTTCTCTTTCTCATCTTTTCACTTCTCCTAT
CATTATCCTCGGCCGAATTCCTCGAGTCGGGAAAACAACTTTGGCTACAATCTAGAAGTAAAGGAGAAGA
ACTTTTCACTGGAGTTGTCCCAATTCTTGTTGAATTAGATGGTGATGTTAATGGGTACAAATTTTCTGTC
AGTGGAGAGGGTGAAGGTGATGCAACATACGGAAAACTTACCCTTAAATTTATTTGCACTACTGGAAAAC
TACCTGTTCCATGGCCAACACTTGTCACTACTTTCTCTTATGGTGTTCAATGCTTTTCAAGATACCCAGA
TCATATGAAGCGGCACGACTTCTTCAAGAGCGCCATGCCTGAGGGATACGTGCAGGAGAGGACCATCTTC
TTCAAGGACGACGGGAACTACAAGACACGTGCTGAAGTCAAGTTTGAGGGAGACACCCTCGTCAACAGGA
TCGAGCTTAAGGGAATCGATTTCAAGGAGGACGGAAACATCCTCGGCCACAAGTTGGAATACAACTACAA
CTCCCACAACGTATACATCATGGCCGACAAGCAAAAGAACGGCATCAAAGCCAACTTCAAGACCCGCCAC
AACATCGAAGACGGCGGCGTGCAACTCGCTGATCATTATCAACAAAATACTCCAATTGGCGATGGCCCTG
TCCTTTTACCAGACAACCATTACCTGTCCACACAATCTGCCCTTTCGAAAGATCCCAACGAAAAGAGAGA
CCACATGGTCCTTCTTGAGTTTGTAACAGCTGCTGGGATTACACATGGCATGGATGAACTATACAAACAT
GATGAGCTTTGAACTAGAGTCCTGCTTTAATGAGATATGCGAGACGCCTATGATCGCATGATATTTGCTT
TCAATTCTGTTGTGCACGTTGTAAAAAACCTGAGCATGTGTAGCTCAGATCCTTACCGCCGGTTTCGGTT
CATTCTAATGAATATATCACCCGTTACTATCGTATTTTTATGAATAATATTCTCCGTTCAATTTACTGAT
TGTACCCTACTACTTATATGTACAATATTAAAATGAAAACAATATATTGTGCTGAATAGGTTTATAGCGA
CATCTATGATAGAGCGCCACAATAACAAACAATTGCGTTTTATTATTACAAATCCAATTTTAAAAAAAGC
GGCAGAACCGGTCAAACCTAAAAGACTGATTACATAAATCTTATTCAAATTTCAAAAGGCCCCAGGGGCT
AGTATCTACGACACACCGAGCGGCGAACTAATAACGTTCACTGAAGGGAACTCCGGTTCCCCGCCGGCGC
GCATGGGTGAGATTCCTTGAAGTTGAGTATTGGCCGTCCGCTCTACCGAAAGTTACGGGCACCATTCAAC
CCGGTCCAGCACGGCGGCCGGGTAACCGACTTGCTGCCCCGAGAATTATGCAGCATTTTTTTGGTGTATG
TGGGCCCCAAATGAAGTGCAGGTCAAACCTTGACAGTGACGACAAATCGTTGGGCGGGTCCAGGGCGAAT
TTTGCGACAACATGTCGAGGCTCAGCAGGACCTGCAGGCATGCAAGCTAGCTTACTAGTGATGCATATTC
TATAGTGTCACCTAAATCTGC

Supplemental Data 2. Related sequences of CTV-N4p23 mutants with point mutation

Sequence of pH7LIC3.1.1(35S-CCDB)

GGGTACCCTCGAATTATCATACATGAGAATTAAGGGAGTCACGTTATGACCCCGCCGATGACGCGGGACA
AGCCGTTTTACGTTTGGAACTGACAGAACCGCAACGTTGAAGGAGCCACTGAGCCGCGGGTTTCTGGAGT
TTAATGAGCTAAGCACATACGTCAGAAACCATTATTGCGCGTTCAAAAGTCGCCTAAGGTCACTATCAGC
TAGCAAATATTTCTTGTCAAAAATGCTCCACTGACGTTCCATAAATTCCCCTCGGTATCCAATTAGAGTC
TCATATTCACTCTCAACTCGAATCCCCCCTATGAAAAAGCCTGAACTCACCGCGACGTCTGTCGAGAAGT
TTCTGATCGAAAAGTTCGACAGCGTCTCCGACCTGATGCAGCTCTCGGAGGGCGAAGAATCTCGTGCTTT
CAGCTTCGATGTAGGAGGGCGTGGATATGTCCTGCGGGTAAATAGCTGCGCCGATGGTTTCTACAAAGAT
CGTTATGTTTATCGGCACTTTGCATCGGCCGCGCTCCCGATTCCGGAAGTGCTTGACATTGGGGAGTTCA
GCGAGAGCCTGACCTATTGCATCTCCCGCCGTGCACAGGGTGTCACGTTGCAAGACCTGCCTGAAACCGA
ACTGCCCGCTGTTCTTCAGCCGGTCGCGGAGGCTATGGATGCTATCGCTGCGGCCGATCTTAGCCAGACG
AGCGGGTTCGGCCCATTCGGACCGCAAGGAATCGGTCAATACACTACATGGCGTGATTTCATATGCGCGA
TTGCTGATCCCCATGTGTATCACTGGCAAACTGTGATGGACGACACCGTCAGTGCGTCCGTCGCGCAGGC
TCTCGATGAGCTGATGCTTTGGGCGAGGACTGCCCCGAAGTCCGGCACTCGTGCACGCGGATTCGGCTCC
AACAATGTCCTGACGGACAATGGCCGCATAACAGCGGTCATTGACTGGAGCGAGGCGATGTTCGGGGATT
CCCAATACGAGGTCGCCAACATCTTCTTCTGGAGGCCGTGGTTGGCTTGTATGGAGCAGCAGACGCGCTA
CTTCGAGCGGAGGCATCCGGAGCTTGCAGGATCGCCAAGGCTCCGGGCGTATATGCTCCGCATTGGTCTT
GACCAACTCTATCAGAGCTTGGTTGACGGCAATTTCGATGATGCAGCTTGGGCGCAGGGTCGATGCGACG
CAATCGTCCGATCCGGAGCCGGGACTGTCGGGCGTACACAAATCGCCCGCAGAAGCGCGGCCGTCTGGAC
CGATGGCTGTGTAGAAGTACTCGCCGATAGTGGAAACCGACGCCCCAGCACTCGTCCGAGGGCAAAGGAA
TAGAGTAGATGCCGACCGAACAAGAGCTGATTTCGAGAACGCCTCAGCCAGCAACTCGCGCGAGCCTAGC
AAGGCAAATGCGAGAGAACGGCCTTACGCTTGGTGGCACAGTTCTCGTCCACAGTTCGCTAAGCTCGCTC
GGCTGGGTCGCGGGAGGGCCGGTCGCAGTGATTCAGGAATTAATTCCCTAGAGTCAAGCAGATCGTTCAA
ACATTTGGCAATAAAGTTTCTTAAGATTGAATCCTGTTGCCGGTCTTGCGATGATTATCATATAATTTCT
GTTGAATTACGTTAAGCATGTAATAATTAACATGTAATGCATGACGTTATTTATGAGATGGGTTTTTATG
ATTAGAGTCCCGCAATTATACATTTAATACGCGATAGAAAACAAAATATAGCGCGCAAACTAGGATAAAT
TATCGCGCGCGGTGTCATCTATGTTACTAGATCGACCGGCATGCAAGCTGATAATTCAATTCGGCGTTAA
TTCAGTACATTAAAAACGTCCGCAATGTGTTATTAAGTTGTCTAAGCGTCAATTTGTTTACACCACAATA
TATCCTGCCACCAGCCAGCCAACAGCTCCCCGACCGGCAGCTCGGCACAAAATCACCACTCGATACAGGC
AGCCCATCAGTCCGGGACGGCGTCAGCGGGAGAGCCGTTGTAAGGCGGCAGACTTTGCTCATGTTACCGA
TGCTATTCGGAAGAACGGCAACTAAGCTGCCGGGTTTGAAACACGGATGATCTCGCGGAGGGTAGCATGT
TGATTGTAACGATGACAGAGCGTTGCTGCCTGTGATCAATTCGGGCACGAACCCAGTGGACATAAGCCTC
GTTCGGTTCGTAAGCTGTAATGCAAGTAGCGTAACTGCCGTCACGCAACTGGTCCAGAACCTTGACCGAA
CGCAGCGGTGGTAACGGCGCAGTGGCGGTTTTCATGGCTTCTTGTTATGACATGTTTTTTTGGGGTACAG
TCTATGCCTCGGGCATCCAAGCAGCAAGCGCGTTACGCCGTGGGTCGATGTTTGATGTTATGGAGCAGCA
ACGATGTTACGCAGCAGGGCAGTCGCCCTAAAACAAAGTTAAACATCATGGGGGAAGCGGTGATCGCCGA
AGTATCGACTCAACTATCAGAGGTAGTTGGCGTCATCGAGCGCCATCTCGAACCGACGTTGCTGGCCGTA
CATTTGTACGGCTCCGCAGTGGATGGCGGCCTGAAGCCACACAGTGATATTGATTTGCTGGTTACGGTGA
CCGTAAGGCTTGATGAAACAACGCGGCGAGCTTTGATCAACGACCTTTTGGAAACTTCGGCTTCCCCTGG
AGAGAGCGAGATTCTCCGCGCTGTAGAAGTCACCATTGTTGTGCACGACGACATCATTCCGTGGCGTTAT
CCAGCTAAGCGCGAACTGCAATTTGGAGAATGGCAGCGCAATGACATTCTTGCAGGTATCTTCGAGCCAG

CCACGATCGACATTGATCTGGCTATCTTGCTGACAAAAGCAAGAGAACATAGCGTTGCCTTGGTAGGTCC
AGCGGCGGAGGAACTCTTTGATCCGGTTCCTGAACAGGATCTATTTGAGGCGCTAAATGAAACCTTAACG
CTATGGAACTCGCCGCCCGACTGGGCTGGCGATGAGCGAAATGTAGTGCTTACGTTGTCCCGCATTTGGT
ACAGCGCAGTAACCGGCAAAATCGCGCCGAAGGATGTCGCTGCCGACTGGGCAATGGAGCGCCTGCCGGC
CCAGTATCAGCCCGTCATACTTGAAGCTAGACAGGCTTATCTTGGACAAGAAGAAGATCGCTTGGCCTCG
CGCGCAGATCAGTTGGAAGAATTTGTCCACTACGTGAAAGGCGAGATCACCAAGGTAGTCGGCAAATAAT
GTCTAGCTAGAAATTCGTTCAAGCCGACGCCGCTTCGCCGGCGTTAACTCAAGCGATTAGATGCACTAAG
CACATAATTGCTCACAGCCAAACTATCAGGTCAAGTCTGCTTTTATTATTTTTAAGCGTGCATAATAAGC
CCTACACAAATTGGGAGATATATCATGCATGACCAAAATCCCTTAACGTGAGTTTTCGTTCCACTGAGCG
TCAGACCCCGTAGAAAAGATCAAAGGATCTTCTTGAGATCCTTTTTTTCTGCGCGTAATCTGCTGCTTGC
AAACAAAAAAACCACCGCTACCAGCGGTGGTTTGTTTGCCGGATCAAGAGCTACCAACTCTTTTTCCGAA
GGTAACTGGCTTCAGCAGAGCGCAGATACCAAATACTGTCCTTCTAGTGTAGCCGTAGTTAGGCCACCAC
TTCAAGAACTCTGTAGCACCGCCTACATACCTCGCTCTGCTAATCCTGTTACCAGTGGCTGCTGCCAGTG
GCGATAAGTCGTGTCTTACCGGGTTGGACTCAAGACGATAGTTACCGGATAAGGCGCAGCGGTCGGGCTG
AACGGGGGGTTCGTGCACACAGCCCAGCTTGGAGCGAACGACCTACACCGAACTGAGATACCTACAGCGT
GAGCTATGAGAAAGCGCCACGCTTCCCGAAGGGAGAAAGGCGGACAGGTATCCGGTAAGCGGCAGGGTCG
GAACAGGAGAGCGCACGAGGGAGCTTCCAGGGGGAAACGCCTGGTATCTTTATAGTCCTGTCGGGTTTCG
CCACCTCTGACTTGAGCGTCGATTTTTGTGATGCTCGTCAGGGGGGCGGAGCCTATGGAAAAACGCCAGC
AACGCGGCCTTTTTACGGTTCCTGGCCTTTTGCTGGCCTTTTGCTCACATGTTCTTTCCTGCGTTATCCC
CTGATTCTGTGGATAACCGTATTACCGCCTTTGAGTGAGCTGATACCGCTCGCCGCAGCCGAACGACCGA
GCGCAGCGAGTCAGTGAGCGAGGAAGCGGAAGAGCGCCTGATGCGGTATTTTCTCCTTACGCATCTGTGC
GGTATTTCACACCGCATATGGTGCACTCTCAGTACAATCTGCTCTGATGCCGCATAGTTAAGCCAGTATA
CACTCCGCTATCGCTACGTGACTGGGTCATGGCTGCGCCCCGACACCCGCCAACACCCGCTGACGCGCCC
TGACGGGCTTGTCTGCTCCCGGCATCCGCTTACAGACAAGCTGTGACCGTCTCCGGGAGCTGCATGTGTC
AGAGGTTTTCACCGTCATCACCGAAACGCGCGAGGCAGGGTGCCTTGATGTGGGCGCCGGCGGTCGAGTG
GCGACGGCGCGGCTTGTCCGCGCCCTGGTAGATTGCCTGGCCGTAGGCCAGCCATTTTTGAGCGGCCAGC
GGCCGCGATAGGCCGACGCGAAGCGGCGGGGCGTAGGGAGCGCAGCGACCGAAGGGTAGGCGCTTTTTGC
AGCTCTTCGGCTGTGCGCTGGCCAGACAGTTATGCACAGGCCAGGCGGGTTTTAAGAGTTTTAATAAGTT
TTAAAGAGTTTTAGGCGGAAAAATCGCCTTTTTTCTCTTTTATATCAGTCACTTACATGTGTGACCGGTT
CCCAATGTACGGCTTTGGGTTCCCAATGTACGGGTTCCGGTTCCCAATGTACGGCTTTGGGTTCCCAATG
TACGTGCTATCCACAGGAAAGAGACCTTTTCGACCTTTTTCCCCTGCTAGGGCAATTTGCCCTAGCATCT
GCTCCGTACATTAGGAACCGGCGGATGCTTCGCCCTCGATCAGGTTGCGGTAGCGCATGACTAGGATCGG
GCCAGCCTGCCCCGCCTCCTCCTTCAAATCGTACTCCGGCAGGTCATTTGACCCGATCAGCTTGCGCACG
GTGAAACAGAACTTCTTGAACTCTCCGGCGCTGCCACTGCGTTCGTAGATCGTCTTGAACAACCATCTGG
CTTCTGCCTTGCCTGCGGCGCGGCGTGCCAGGCGGTAGAGAAAACGGCCGATGCCGGGATCGATCAAAAA
GTAATCGGGGTGAACCGTCAGCACGTCCGGGTTCTTGCCTTCTGTGATCTCGCGGTACATCCAATCAGCT
AGCTCGATCTCGATGTACTCCGGCCGCCCGGTTTCGCTCTTTACGATCTTGTAGCGGCTAATCAAGGCTT
CACCCTCGGATACCGTCACCAGGCGGCCGTTCTTGGCCTTCTTCGTACGCTGCATGGCAACGTGCGTGGT
GTTTAACCGAATGCAGGTTTCTACCAGGTCGTCTTTCTGCTTTCCGCCATCGGCTCGCCGGCAGAACTTG
AGTACGTCCGCAACGTGTGGACGGAACACGCGGCCGGGCTTGTCTCCCTTCCCTTCCCGGTATCGGTTCA
TGGATTCGGTTAGATGGGAAACCGCCATCAGTACCAGGTCGTAATCCCACACACTGGCCATGCCGGCCGG
CCCTGCGGAAACCTCTACGTGCCCGTCTGGAAGCTCGTAGCGGATCACCTCGCCAGCTCGTCGGTCACGC
TTCGACAGACGGAAAACGGCCACGTCCATGATGCTGCGACTATCGCGGGTGCCCACGTCATAGAGCATCG
GAACGAAAAAATCTGGTTGCTCGTCGCCCTTGGGCGGCTTCCTAATCGACGGCGCACCGGCTGCCGGCGG

TTGCCGGGATTCTTTGCGGATTCGATCAGCGGCCGCTTGCCACGATTCACCGGGGCGTGCTTCTGCCTCG
ATGCGTTGCCGCTGGGCGGCCTGCGCGGCCTTCAACTTCTCCACCAGGTCATCACCCAGCGCCGCGCCGA
TTTGTACCGGGCCGGATGGTTTGCGACCGTCACGCCGATTCCTCGGGCTTGGGGGTTCCAGTGCCATTGC
AGGGCCGGCAGACAACCCAGCCGCTTACGCCTGGCCAACCGCCCGTTCCTCCACACATGGGGCATTCCAC
GGCGTCGGTGCCTGGTTGTTCTTGATTTTCCATGCCGCCTCCTTTAGCCGCTAAAATTCATCTACTCATT
TATTCATTTGCTCATTTACTCTGGTAGCTGCGCGATGTATTCAGATAGCAGCTCGGTAATGGTCTTGCCT
TGGCGTACCGCGTACATCTTCAGCTTGGTGTGATCCTCCGCCGGCAACTGAAAGTTGACCCGCTTCATGG
CTGGCGTGTCTGCCAGGCTGGCCAACGTTGCAGCCTTGCTGCTGCGTGCGCTCGGACGGCCGGCACTTAG
CGTGTTTGTGCTTTTGCTCATTTTCTCTTTACCTCATTAACTCAAATGAGTTTTGATTTAATTTCAGCGG
CCAGCGCCTGGACCTCGCGGGCAGCGTCGCCCTCGGGTTCTGATTCAAGAACGGTTGTGCCGGCGGCGGC
AGTGCCTGGGTAGCTCACGCGCTGCGTGATACGGGACTCAAGAATGGGCAGCTCGTACCCGGCCAGCGCC
TCGGCAACCTCACCGCCGATGCGCGTGCCTTTGATCGCCCGCGACACGACAAAGGCCGCTTGTAGCCTTC
CATCCGTGACCTCAATGCGCTGCTTAACCAGCTCCACCAGGTCGGCGGTGGCCCATATGTCGTAAGGGCT
TGGCTGCACCGGAATCAGCACGAAGTCGGCTGCCTTGATCGCGGACACAGCCAAGTCCGCCGCCTGGGGC
GCTCCGTCGATCACTACGAAGTCGCGCCGGCCGATGGCCTTCACGTCGCGGTCAATCGTCGGGCGGTCGA
TGCCGACAACGGTTAGCGGTTGATCTTCCCGCACGGCCGCCCAATCGCGGGCACTGCCCTGGGGATCGGA
ATCGACTAACAGAACATCGGCCCCGGCGAGTTGCAGGGCGCGGGCTAGATGGGTTGCGATGGTCGTCTTG
CCTGACCCGCCTTTCTGGTTAAGTACAGCGATAACCTTCATGCGTTCCCCTTGCGTATTTGTTTATTTAC
TCATCGCATCATATACGCAGCGACCGCATGACGCAAGCTGTTTTACTCAAATACACATCACCTTTTTAGA
CGGCGGCGCTCGGTTTCTTCAGCGGCCAAGCTGGCCGGCCAGGCCGCCAGCTTGGCATCAGACAAACCGG
CCAGGATTTCATGCAGCCGCACGGTTGAGACGTGCGCGGGCGGCTCGAACACGTACCCGGCCGCGATCAT
CTCCGCCTCGATCTCTTCGGTAATGAAAAACGGTTCGTCCTGGCCGTCCTGGTGCGGTTTCATGCTTGTT
CCTCTTGGCGTTCATTCTCGGCGGCCGCCAGGGCGTCGGCCTCGGTCAATGCGTCCTCACGGAAGGCACC
GCGCCGCCTGGCCTCGGTGGGCGTCACTTCCTCGCTGCGCTCAAGTGCGCGGTACAGGGTCGAGCGATGC
ACGCCAAGCAGTGCAGCCGCCTCTTTCACGGTGCGGCCTTCCTGGTCGATCAGCTCGCGGGCGTGCGCGA
TCTGTGCCGGGGTGAGGGTAGGGCGGGGGCCAAACTTCACGCCTCGGGCCTTGGCGGCCTCGCGCCCGCT
CCGGGTGCGGTCGATGATTAGGGAACGCTCGAACTCGGCAATGCCGGCGAACACGGTCAACACCATGCGG
CCGGCCGGCGTGGTGGTGTCGGCCCACGGCTCTGCCAGGCTACGCAGGCCCGCGCCGGCCTCCTGGATGC
GCTCGGCAATGTCCAGTAGGTCGCGGGTGCTGCGGGCCAGGCGGTCTAGCCTGGTCACTGTCACAACGTC
GCCAGGGCGTAGGTGGTCAAGCATCCTGGCCAGCTCCGGGCGGTCGCGCCTGGTGCCGGTGATCTTCTCG
GAAAACAGCTTGGTGCAGCCGGCCGCGTGCAGTTCGGCCCGTTGGTTGGTCAAGTCCTGGTCGTCGGTGC
TGACGCGGGCATAGCCCAGCAGGCCAGCGGCGGCGCTCTTGTTCATGGCGTAATGTCTCCGGTTCTAGTC
GCAAGTATTCTACTTTATGCGACTAAAACACGCGACAAGAAAACGCCAGGAAAAGGGCAGGGCGGCAGCC
TGTCGCGTAACTTAGGACTTGTGCGACATGTCGTTTTCAGAAGACGGCTGCACTGAACGTCAGAAGCCGA
CTGCACTATAGCAGCGGAGGGGTTGGATCAAAGTACTTTGATCCCGAGGGGAACCCTGTGGTTGGCATGC
ACATACAAATGGACGAACGGATAAACCTTTTCACGCCCTTTTAAATATCCGTTATTCTAATAAACGCTCT
TTTCTCTTAGGTTTACCCGCCAATATATCCTGTCAAACACTGATAGTTTAAACTGAAGGCGGGAAACGAC
AATCTGATCCAAGCTCAAGCTAAGCTTGAGCTCTGCATGCCTGCAGGTCCCCAGATTAGCCTTTTCAATT
TCAGAAAGAATGCTAACCCACAGATGGTTAGAGAGGCTTACGCAGCAGGTCTCATCAAGACGATCTACCC
GAGCAATAATCTCCAGGAAATCAAATACCTTCCCAAGAAGGTTAAAGATGCAGTCAAAAGATTCAGGACT
AACTGCATCAAGAACACAGAGAAAGATATATTTCTCAAGATCAGAAGTACTATTCCAGTATGGACGATTC
AAGGCTTGCTTCACAAACCAAGGCAAGTAATAGAGATTGGAGTCTCTAAAAAGGTAGTTCCCACTGAATC
AAAGGCCATGGAGTCAAAGATTCAAATAGAGGACCTAACAGAACTCGCCGTAAAGACTGGCGAACAGTTC
ATACAGAGTCTCTTACGACTCAATGACAAGAAGAAAATCTTCGTCAACATGGTGGAGCACGACACACTTG

TCTACTCCAAAAATATCAAAGATACAGTCTCAGAAGACCAAAGGGCAATTGAGACTTTTCAACAAAGGGT
AATATCCGGAAACCTCCTCGGATTCCATTGCCCAGCTATCTGTCACTTTATTGTGAAGATAGTGGAAAAG
GAAGGTGGCTCCTACAAATGCCATCATTGCGATAAAGGAAAGGCCATCGTTGAAGATGCCTCTGCCGACA
GTGGTCCCAAAGATGGACCCCCACCCACGAGGAGCATCGTGGAAAAAGAAGACGTTCCAACCACGTCTTC
AAAGCAAGTGGATTGATGTGATATCTCCACTGACGTAAGGGATGACGCACAATCCCACTATCCTTCGCAA
GACCCTTCCTCTATATAAGGAAGTTCATTTCATTTGGAGAGAACACGGGGGACTCTAGAGGATCCAAGGA
GATATAAGCCGCCACCATGGATTACAAGGACGATGATGACAAGGACTACAAGGACGATGACGATAAGGAC
TATAAGGATGACGATGACAAGGATTACGCCGAGGCCTGTCGACCTGCAGACTGGCTGTGTATAAGGGAGC
CTGACATTTATATTCCCCAGAACATCAGGTTAATGGCGTTTTTGATGTCATTTTCGCGGTGGCTGAGATC
AGCCACTTCTTCCCCGATAACGGAGACCGGCACACTGGCCATATCGGTGGTCATCATGCGCCAGCTTTCA
TCCCCGATATGCACCACCGGGTAAAGTTCACGGGAGACTTTATCTGACAGCAGACGTGCACTGGCCAGGG
GGATCACCATCCGTCGCCCGGGCGTGTCAATAATATCACTCTGTACATCCACAAACAGACGATAACGGCT
CTCTCTTTTATAGGTGTAAACCTTAAACTGCATTTCACCAGCCCCTGTTCTCGTCAGCAAAAGAGCCGTT
CATTTCAATAAACCGGGCGACCTCAGCCATCCCTTCCTGATTTTCCGCTTTCCAGCGTTCGGCACGCAGA
CGACGGGCTTCATTCTGCATGGTTGTGCTTACCAGACCGGAGATATTGACATCATATATGCCTTGAGCAA
CTGATAGCTGTCGCTGTCAACTGTCACTGTAATACGCTGCTTCATAGCATACCTCTTTTTGACATACTTC
GGGTATACATATCAGTATATATTCTTATACCGCAAAAATCAGCGCGCAAATACGCATACTGTTATCTGGC
TTTTAGTAAGCCGGATCCACGCGTTTACGCCCCGCCCTGCCACTCATCGCAGTACTGTTGTAATTCATTA
AGCATTCTGCCGACATGGAAGCCATCACAGACGGCATGATGAACCTGAATCGCCAGCGGCATCAGCACCT
TGTCGCCTTGCGTATAATATTTGCCCATGGTGAAAACGGGGGCGAAGAAGTTGTCCATATTGGCCACGTT
TAAATCAAAACTGGTGAAACTCACCCAGGGATTGGCTGAGACGAAAAACATATTCTCAATAAACCCTTTA
GGGAAATAGGCCAGGTTTTCACCGTAACACGCCACATCTTGCGAATATATGTGTAGAAACTGCCGGAAAT
CGTCGTGGTATTCACTCCAGAGCGATGAAAACGTTTCAGTTTGCTCATGGAAAACGGTGTAACAAGGGTG
AACACTATCCCATATCACCAGCTCACCGTCTTTCATTGCCATACGGAATTCCGGATGAGCATTCATCAGG
CGGGCAAGAATGTGAATAAAGGCCGGATAAAACTTGTGCTTATTTTTCTTTACGGTCTTTAAAAAGGCCG
TAATATCCAGCTGAACGGTCTGGTTATAGGTACATTGAGCAACTGACTGAAATGCCTCAAAATGTTCTTT
ACGATGCCATTGGGATATATCAACGGTGGTATATCCAGTGATTTTTTTCTCCATTTTAGCTTCCTTAGCT
CCTGAAAATCTCGCCGGATCCTAACTCAAAATCCACACATTATACGAGCCGGAAGCATAAAGTGTAAAGC
CTGGGGTGCCTAATGCGGCCGCAGGCCTCTTCCCTAATGATATCCCGCGGCCATGCTAGAGTCCGCAAAA
ATCACCAGTCTCTCTCTACAAATCTATCTCTCTCTATTTTTCTCCAGAATAATGTGTGAGTAGTTCCCAG
ATAAGGGAATTAGGGTTCTTATAGGGTTTCGCTCATGTGTTGAGCATATAAGAAACCCTTAGTATGTATT
TGTATTTGTAAAATACTTCTATCAATAAAATTTCTAATTCCTAAAACCAAAATCCAGTGACCTGCAGGCA
TGCGACGTCG

Sequence of pH7LIC3.1.1-p23M1 (35S- p23M1)

GGGTACCCTCGAATTATCATACATGAGAATTAAGGGAGTCACGTTATGACCCCGCCGATGACGCGGGACA
AGCCGTTTTACGTTTGGAACTGACAGAACCGCAACGTTGAAGGAGCCACTGAGCCGCGGGTTTCTGGAGT
TTAATGAGCTAAGCACATACGTCAGAAACCATTATTGCGCGTTCAAAAGTCGCCTAAGGTCACTATCAGC
TAGCAAATATTTCTTGTCAAAAATGCTCCACTGACGTTCCATAAATTCCCCTCGGTATCCAATTAGAGTC
TCATATTCACTCTCAACTCGAATCCCCCCTATGAAAAAGCCTGAACTCACCGCGACGTCTGTCGAGAAGT
TTCTGATCGAAAAGTTCGACAGCGTCTCCGACCTGATGCAGCTCTCGGAGGGCGAAGAATCTCGTGCTTT
CAGCTTCGATGTAGGAGGGCGTGGATATGTCCTGCGGGTAAATAGCTGCGCCGATGGTTTCTACAAAGAT
CGTTATGTTTATCGGCACTTTGCATCGGCCGCGCTCCCGATTCCGGAAGTGCTTGACATTGGGGAGTTCA
GCGAGAGCCTGACCTATTGCATCTCCCGCCGTGCACAGGGTGTCACGTTGCAAGACCTGCCTGAAACCGA

ACTGCCCGCTGTTCTTCAGCCGGTCGCGGAGGCTATGGATGCTATCGCTGCGGCCGATCTTAGCCAGACG
AGCGGGTTCGGCCCATTCGGACCGCAAGGAATCGGTCAATACACTACATGGCGTGATTTCATATGCGCGA
TTGCTGATCCCCATGTGTATCACTGGCAAACTGTGATGGACGACACCGTCAGTGCGTCCGTCGCGCAGGC
TCTCGATGAGCTGATGCTTTGGGCGAGGACTGCCCCGAAGTCCGGCACTCGTGCACGCGGATTCGGCTCC
AACAATGTCCTGACGGACAATGGCCGCATAACAGCGGTCATTGACTGGAGCGAGGCGATGTTCGGGGATT
CCCAATACGAGGTCGCCAACATCTTCTTCTGGAGGCCGTGGTTGGCTTGTATGGAGCAGCAGACGCGCTA
CTTCGAGCGGAGGCATCCGGAGCTTGCAGGATCGCCAAGGCTCCGGGCGTATATGCTCCGCATTGGTCTT
GACCAACTCTATCAGAGCTTGGTTGACGGCAATTTCGATGATGCAGCTTGGGCGCAGGGTCGATGCGACG
CAATCGTCCGATCCGGAGCCGGGACTGTCGGGCGTACACAAATCGCCCGCAGAAGCGCGGCCGTCTGGAC
CGATGGCTGTGTAGAAGTACTCGCCGATAGTGGAAACCGACGCCCCAGCACTCGTCCGAGGGCAAAGGAA
TAGAGTAGATGCCGACCGAACAAGAGCTGATTTCGAGAACGCCTCAGCCAGCAACTCGCGCGAGCCTAGC
AAGGCAAATGCGAGAGAACGGCCTTACGCTTGGTGGCACAGTTCTCGTCCACAGTTCGCTAAGCTCGCTC
GGCTGGGTCGCGGGAGGGCCGGTCGCAGTGATTCAGGAATTAATTCCCTAGAGTCAAGCAGATCGTTCAA
ACATTTGGCAATAAAGTTTCTTAAGATTGAATCCTGTTGCCGGTCTTGCGATGATTATCATATAATTTCT
GTTGAATTACGTTAAGCATGTAATAATTAACATGTAATGCATGACGTTATTTATGAGATGGGTTTTTATG
ATTAGAGTCCCGCAATTATACATTTAATACGCGATAGAAAACAAAATATAGCGCGCAAACTAGGATAAAT
TATCGCGCGCGGTGTCATCTATGTTACTAGATCGACCGGCATGCAAGCTGATAATTCAATTCGGCGTTAA
TTCAGTACATTAAAAACGTCCGCAATGTGTTATTAAGTTGTCTAAGCGTCAATTTGTTTACACCACAATA
TATCCTGCCACCAGCCAGCCAACAGCTCCCCGACCGGCAGCTCGGCACAAAATCACCACTCGATACAGGC
AGCCCATCAGTCCGGGACGGCGTCAGCGGGAGAGCCGTTGTAAGGCGGCAGACTTTGCTCATGTTACCGA
TGCTATTCGGAAGAACGGCAACTAAGCTGCCGGGTTTGAAACACGGATGATCTCGCGGAGGGTAGCATGT
TGATTGTAACGATGACAGAGCGTTGCTGCCTGTGATCAATTCGGGCACGAACCCAGTGGACATAAGCCTC
GTTCGGTTCGTAAGCTGTAATGCAAGTAGCGTAACTGCCGTCACGCAACTGGTCCAGAACCTTGACCGAA
CGCAGCGGTGGTAACGGCGCAGTGGCGGTTTTCATGGCTTCTTGTTATGACATGTTTTTTTGGGGTACAG
TCTATGCCTCGGGCATCCAAGCAGCAAGCGCGTTACGCCGTGGGTCGATGTTTGATGTTATGGAGCAGCA
ACGATGTTACGCAGCAGGGCAGTCGCCCTAAAACAAAGTTAAACATCATGGGGGAAGCGGTGATCGCCGA
AGTATCGACTCAACTATCAGAGGTAGTTGGCGTCATCGAGCGCCATCTCGAACCGACGTTGCTGGCCGTA
CATTTGTACGGCTCCGCAGTGGATGGCGGCCTGAAGCCACACAGTGATATTGATTTGCTGGTTACGGTGA
CCGTAAGGCTTGATGAAACAACGCGGCGAGCTTTGATCAACGACCTTTTGGAAACTTCGGCTTCCCCTGG
AGAGAGCGAGATTCTCCGCGCTGTAGAAGTCACCATTGTTGTGCACGACGACATCATTCCGTGGCGTTAT
CCAGCTAAGCGCGAACTGCAATTTGGAGAATGGCAGCGCAATGACATTCTTGCAGGTATCTTCGAGCCAG
CCACGATCGACATTGATCTGGCTATCTTGCTGACAAAAGCAAGAGAACATAGCGTTGCCTTGGTAGGTCC
AGCGGCGGAGGAACTCTTTGATCCGGTTCCTGAACAGGATCTATTTGAGGCGCTAAATGAAACCTTAACG
CTATGGAACTCGCCGCCCGACTGGGCTGGCGATGAGCGAAATGTAGTGCTTACGTTGTCCCGCATTTGGT
ACAGCGCAGTAACCGGCAAAATCGCGCCGAAGGATGTCGCTGCCGACTGGGCAATGGAGCGCCTGCCGGC
CCAGTATCAGCCCGTCATACTTGAAGCTAGACAGGCTTATCTTGGACAAGAAGAAGATCGCTTGGCCTCG
CGCGCAGATCAGTTGGAAGAATTTGTCCACTACGTGAAAGGCGAGATCACCAAGGTAGTCGGCAAATAAT
GTCTAGCTAGAAATTCGTTCAAGCCGACGCCGCTTCGCCGGCGTTAACTCAAGCGATTAGATGCACTAAG
CACATAATTGCTCACAGCCAAACTATCAGGTCAAGTCTGCTTTTATTATTTTTAAGCGTGCATAATAAGC
CCTACACAAATTGGGAGATATATCATGCATGACCAAAATCCCTTAACGTGAGTTTTCGTTCCACTGAGCG
TCAGACCCCGTAGAAAAGATCAAAGGATCTTCTTGAGATCCTTTTTTTCTGCGCGTAATCTGCTGCTTGC
AAACAAAAAAACCACCGCTACCAGCGGTGGTTTGTTTGCCGGATCAAGAGCTACCAACTCTTTTTCCGAA
GGTAACTGGCTTCAGCAGAGCGCAGATACCAAATACTGTCCTTCTAGTGTAGCCGTAGTTAGGCCACCAC
TTCAAGAACTCTGTAGCACCGCCTACATACCTCGCTCTGCTAATCCTGTTACCAGTGGCTGCTGCCAGTG

GCGATAAGTCGTGTCTTACCGGGTTGGACTCAAGACGATAGTTACCGGATAAGGCGCAGCGGTCGGGCTG
AACGGGGGGTTCGTGCACACAGCCCAGCTTGGAGCGAACGACCTACACCGAACTGAGATACCTACAGCGT
GAGCTATGAGAAAGCGCCACGCTTCCCGAAGGGAGAAAGGCGGACAGGTATCCGGTAAGCGGCAGGGTCG
GAACAGGAGAGCGCACGAGGGAGCTTCCAGGGGGAAACGCCTGGTATCTTTATAGTCCTGTCGGGTTTCG
CCACCTCTGACTTGAGCGTCGATTTTTGTGATGCTCGTCAGGGGGGCGGAGCCTATGGAAAAACGCCAGC
AACGCGGCCTTTTTACGGTTCCTGGCCTTTTGCTGGCCTTTTGCTCACATGTTCTTTCCTGCGTTATCCC
CTGATTCTGTGGATAACCGTATTACCGCCTTTGAGTGAGCTGATACCGCTCGCCGCAGCCGAACGACCGA
GCGCAGCGAGTCAGTGAGCGAGGAAGCGGAAGAGCGCCTGATGCGGTATTTTCTCCTTACGCATCTGTGC
GGTATTTCACACCGCATATGGTGCACTCTCAGTACAATCTGCTCTGATGCCGCATAGTTAAGCCAGTATA
CACTCCGCTATCGCTACGTGACTGGGTCATGGCTGCGCCCCGACACCCGCCAACACCCGCTGACGCGCCC
TGACGGGCTTGTCTGCTCCCGGCATCCGCTTACAGACAAGCTGTGACCGTCTCCGGGAGCTGCATGTGTC
AGAGGTTTTCACCGTCATCACCGAAACGCGCGAGGCAGGGTGCCTTGATGTGGGCGCCGGCGGTCGAGTG
GCGACGGCGCGGCTTGTCCGCGCCCTGGTAGATTGCCTGGCCGTAGGCCAGCCATTTTTGAGCGGCCAGC
GGCCGCGATAGGCCGACGCGAAGCGGCGGGGCGTAGGGAGCGCAGCGACCGAAGGGTAGGCGCTTTTTGC
AGCTCTTCGGCTGTGCGCTGGCCAGACAGTTATGCACAGGCCAGGCGGGTTTTAAGAGTTTTAATAAGTT
TTAAAGAGTTTTAGGCGGAAAAATCGCCTTTTTTCTCTTTTATATCAGTCACTTACATGTGTGACCGGTT
CCCAATGTACGGCTTTGGGTTCCCAATGTACGGGTTCCGGTTCCCAATGTACGGCTTTGGGTTCCCAATG
TACGTGCTATCCACAGGAAAGAGACCTTTTCGACCTTTTTCCCCTGCTAGGGCAATTTGCCCTAGCATCT
GCTCCGTACATTAGGAACCGGCGGATGCTTCGCCCTCGATCAGGTTGCGGTAGCGCATGACTAGGATCGG
GCCAGCCTGCCCCGCCTCCTCCTTCAAATCGTACTCCGGCAGGTCATTTGACCCGATCAGCTTGCGCACG
GTGAAACAGAACTTCTTGAACTCTCCGGCGCTGCCACTGCGTTCGTAGATCGTCTTGAACAACCATCTGG
CTTCTGCCTTGCCTGCGGCGCGGCGTGCCAGGCGGTAGAGAAAACGGCCGATGCCGGGATCGATCAAAAA
GTAATCGGGGTGAACCGTCAGCACGTCCGGGTTCTTGCCTTCTGTGATCTCGCGGTACATCCAATCAGCT
AGCTCGATCTCGATGTACTCCGGCCGCCCGGTTTCGCTCTTTACGATCTTGTAGCGGCTAATCAAGGCTT
CACCCTCGGATACCGTCACCAGGCGGCCGTTCTTGGCCTTCTTCGTACGCTGCATGGCAACGTGCGTGGT
GTTTAACCGAATGCAGGTTTCTACCAGGTCGTCTTTCTGCTTTCCGCCATCGGCTCGCCGGCAGAACTTG
AGTACGTCCGCAACGTGTGGACGGAACACGCGGCCGGGCTTGTCTCCCTTCCCTTCCCGGTATCGGTTCA
TGGATTCGGTTAGATGGGAAACCGCCATCAGTACCAGGTCGTAATCCCACACACTGGCCATGCCGGCCGG
CCCTGCGGAAACCTCTACGTGCCCGTCTGGAAGCTCGTAGCGGATCACCTCGCCAGCTCGTCGGTCACGC
TTCGACAGACGGAAAACGGCCACGTCCATGATGCTGCGACTATCGCGGGTGCCCACGTCATAGAGCATCG
GAACGAAAAAATCTGGTTGCTCGTCGCCCTTGGGCGGCTTCCTAATCGACGGCGCACCGGCTGCCGGCGG
TTGCCGGGATTCTTTGCGGATTCGATCAGCGGCCGCTTGCCACGATTCACCGGGGCGTGCTTCTGCCTCG
ATGCGTTGCCGCTGGGCGGCCTGCGCGGCCTTCAACTTCTCCACCAGGTCATCACCCAGCGCCGCGCCGA
TTTGTACCGGGCCGGATGGTTTGCGACCGTCACGCCGATTCCTCGGGCTTGGGGGTTCCAGTGCCATTGC
AGGGCCGGCAGACAACCCAGCCGCTTACGCCTGGCCAACCGCCCGTTCCTCCACACATGGGGCATTCCAC
GGCGTCGGTGCCTGGTTGTTCTTGATTTTCCATGCCGCCTCCTTTAGCCGCTAAAATTCATCTACTCATT
TATTCATTTGCTCATTTACTCTGGTAGCTGCGCGATGTATTCAGATAGCAGCTCGGTAATGGTCTTGCCT
TGGCGTACCGCGTACATCTTCAGCTTGGTGTGATCCTCCGCCGGCAACTGAAAGTTGACCCGCTTCATGG
CTGGCGTGTCTGCCAGGCTGGCCAACGTTGCAGCCTTGCTGCTGCGTGCGCTCGGACGGCCGGCACTTAG
CGTGTTTGTGCTTTTGCTCATTTTCTCTTTACCTCATTAACTCAAATGAGTTTTGATTTAATTTCAGCGG
CCAGCGCCTGGACCTCGCGGGCAGCGTCGCCCTCGGGTTCTGATTCAAGAACGGTTGTGCCGGCGGCGGC
AGTGCCTGGGTAGCTCACGCGCTGCGTGATACGGGACTCAAGAATGGGCAGCTCGTACCCGGCCAGCGCC
TCGGCAACCTCACCGCCGATGCGCGTGCCTTTGATCGCCCGCGACACGACAAAGGCCGCTTGTAGCCTTC
CATCCGTGACCTCAATGCGCTGCTTAACCAGCTCCACCAGGTCGGCGGTGGCCCATATGTCGTAAGGGCT

TGGCTGCACCGGAATCAGCACGAAGTCGGCTGCCTTGATCGCGGACACAGCCAAGTCCGCCGCCTGGGGC
GCTCCGTCGATCACTACGAAGTCGCGCCGGCCGATGGCCTTCACGTCGCGGTCAATCGTCGGGCGGTCGA
TGCCGACAACGGTTAGCGGTTGATCTTCCCGCACGGCCGCCCAATCGCGGGCACTGCCCTGGGGATCGGA
ATCGACTAACAGAACATCGGCCCCGGCGAGTTGCAGGGCGCGGGCTAGATGGGTTGCGATGGTCGTCTTG
CCTGACCCGCCTTTCTGGTTAAGTACAGCGATAACCTTCATGCGTTCCCCTTGCGTATTTGTTTATTTAC
TCATCGCATCATATACGCAGCGACCGCATGACGCAAGCTGTTTTACTCAAATACACATCACCTTTTTAGA
CGGCGGCGCTCGGTTTCTTCAGCGGCCAAGCTGGCCGGCCAGGCCGCCAGCTTGGCATCAGACAAACCGG
CCAGGATTTCATGCAGCCGCACGGTTGAGACGTGCGCGGGCGGCTCGAACACGTACCCGGCCGCGATCAT
CTCCGCCTCGATCTCTTCGGTAATGAAAAACGGTTCGTCCTGGCCGTCCTGGTGCGGTTTCATGCTTGTT
CCTCTTGGCGTTCATTCTCGGCGGCCGCCAGGGCGTCGGCCTCGGTCAATGCGTCCTCACGGAAGGCACC
GCGCCGCCTGGCCTCGGTGGGCGTCACTTCCTCGCTGCGCTCAAGTGCGCGGTACAGGGTCGAGCGATGC
ACGCCAAGCAGTGCAGCCGCCTCTTTCACGGTGCGGCCTTCCTGGTCGATCAGCTCGCGGGCGTGCGCGA
TCTGTGCCGGGGTGAGGGTAGGGCGGGGGCCAAACTTCACGCCTCGGGCCTTGGCGGCCTCGCGCCCGCT
CCGGGTGCGGTCGATGATTAGGGAACGCTCGAACTCGGCAATGCCGGCGAACACGGTCAACACCATGCGG
CCGGCCGGCGTGGTGGTGTCGGCCCACGGCTCTGCCAGGCTACGCAGGCCCGCGCCGGCCTCCTGGATGC
GCTCGGCAATGTCCAGTAGGTCGCGGGTGCTGCGGGCCAGGCGGTCTAGCCTGGTCACTGTCACAACGTC
GCCAGGGCGTAGGTGGTCAAGCATCCTGGCCAGCTCCGGGCGGTCGCGCCTGGTGCCGGTGATCTTCTCG
GAAAACAGCTTGGTGCAGCCGGCCGCGTGCAGTTCGGCCCGTTGGTTGGTCAAGTCCTGGTCGTCGGTGC
TGACGCGGGCATAGCCCAGCAGGCCAGCGGCGGCGCTCTTGTTCATGGCGTAATGTCTCCGGTTCTAGTC
GCAAGTATTCTACTTTATGCGACTAAAACACGCGACAAGAAAACGCCAGGAAAAGGGCAGGGCGGCAGCC
TGTCGCGTAACTTAGGACTTGTGCGACATGTCGTTTTCAGAAGACGGCTGCACTGAACGTCAGAAGCCGA
CTGCACTATAGCAGCGGAGGGGTTGGATCAAAGTACTTTGATCCCGAGGGGAACCCTGTGGTTGGCATGC
ACATACAAATGGACGAACGGATAAACCTTTTCACGCCCTTTTAAATATCCGTTATTCTAATAAACGCTCT
TTTCTCTTAGGTTTACCCGCCAATATATCCTGTCAAACACTGATAGTTTAAACTGAAGGCGGGAAACGAC
AATCTGATCCAAGCTCAAGCTAAGCTTGAGCTCTGCATGCCTGCAGGTCCCCAGATTAGCCTTTTCAATT
TCAGAAAGAATGCTAACCCACAGATGGTTAGAGAGGCTTACGCAGCAGGTCTCATCAAGACGATCTACCC
GAGCAATAATCTCCAGGAAATCAAATACCTTCCCAAGAAGGTTAAAGATGCAGTCAAAAGATTCAGGACT
AACTGCATCAAGAACACAGAGAAAGATATATTTCTCAAGATCAGAAGTACTATTCCAGTATGGACGATTC
AAGGCTTGCTTCACAAACCAAGGCAAGTAATAGAGATTGGAGTCTCTAAAAAGGTAGTTCCCACTGAATC
AAAGGCCATGGAGTCAAAGATTCAAATAGAGGACCTAACAGAACTCGCCGTAAAGACTGGCGAACAGTTC
ATACAGAGTCTCTTACGACTCAATGACAAGAAGAAAATCTTCGTCAACATGGTGGAGCACGACACACTTG
TCTACTCCAAAAATATCAAAGATACAGTCTCAGAAGACCAAAGGGCAATTGAGACTTTTCAACAAAGGGT
AATATCCGGAAACCTCCTCGGATTCCATTGCCCAGCTATCTGTCACTTTATTGTGAAGATAGTGGAAAAG
GAAGGTGGCTCCTACAAATGCCATCATTGCGATAAAGGAAAGGCCATCGTTGAAGATGCCTCTGCCGACA
GTGGTCCCAAAGATGGACCCCCACCCACGAGGAGCATCGTGGAAAAAGAAGACGTTCCAACCACGTCTTC
AAAGCAAGTGGATTGATGTGATATCTCCACTGACGTAAGGGATGACGCACAATCCCACTATCCTTCGCAA
GACCCTTCCTCTATATAAGGAAGTTCATTTCATTTGGAGAGAACACGGGGGACTCTAGAGGATCCAAGGA
GATATAAGCCGCCACCATGGATTACAAGGACGATGATGACAAGGACTACAAGGACGATGACGATAAGGAC
TATAAGGATGACGATGACAAGGATTACGCCGAGGATGGATGATACTAGCGGACAAACTTTCGTTTCTGTG
AACCTTTCTGACGAAAGCAACACGGCAAGCACTAAAGTTGAAAACGTAAAATCGGAAGCGGATCGCTTGG
AGTTTTTACGTAAAATGAATCCCTTTATTGTTGACGCTCTGGTGCGGAAAACCAATTATCAGGGTGCTCG
CTTTCGCGCAAGAATAATAGGAGTGTGCGTGGATTGTGGTAGAGCGGCGGACAAGGCGCTCAAGACTGAA
CGTAAGTGTAAGGTCAACAACACGCAATCTCAGAACGAGGTGGCGCATATGTTGATGCACGATCCCGTTA
AGTATTTGAACAAAAGAAAGGCTAGAGCCTTTTCTAACGCAGAGATGTTTGCGATTGAACTGGTTTCGTA

CACCAAGGAAAGGCAATTGGCGGTCGATTTAGCCGCTGAAAGGGAGAAGACGAGACTGGCTCGTAGACAC
CCAATACGTTCTCCGGAAGAAACTCCGGAACATTATAAATTCGGTATGACTGCTAAGGCAATGTTACCGG
ACATCAACGCCGTAGACGTTGGTGATAACGAGGAAACTTCGTCGGAGTACCCAGTGAGTCTGAGTGTTTC
TGGCGGAGTTCTCCGTGAACACCACTTCATCTGACCTCTTCCCTAATGATATCCCGCGGCCATGCTAGAG
TCCGCAAAAATCACCAGTCTCTCTCTACAAATCTATCTCTCTCTATTTTTCTCCAGAATAATGTGTGAGT
AGTTCCCAGATAAGGGAATTAGGGTTCTTATAGGGTTTCGCTCATGTGTTGAGCATATAAGAAACCCTTA
GTATGTATTTGTATTTGTAAAATACTTCTATCAATAAAATTTCTAATTCCTAAAACCAAAATCCAGTGAC
CTGCAGGCATGCGACGTCG

Sequence of pH7LIC3.1.1-p23M2 (35S- p23M2)

GGGTACCCTCGAATTATCATACATGAGAATTAAGGGAGTCACGTTATGACCCCGCCGATGACGCGGGACA
AGCCGTTTTACGTTTGGAACTGACAGAACCGCAACGTTGAAGGAGCCACTGAGCCGCGGGTTTCTGGAGT
TTAATGAGCTAAGCACATACGTCAGAAACCATTATTGCGCGTTCAAAAGTCGCCTAAGGTCACTATCAGC
TAGCAAATATTTCTTGTCAAAAATGCTCCACTGACGTTCCATAAATTCCCCTCGGTATCCAATTAGAGTC
TCATATTCACTCTCAACTCGAATCCCCCCTATGAAAAAGCCTGAACTCACCGCGACGTCTGTCGAGAAGT
TTCTGATCGAAAAGTTCGACAGCGTCTCCGACCTGATGCAGCTCTCGGAGGGCGAAGAATCTCGTGCTTT
CAGCTTCGATGTAGGAGGGCGTGGATATGTCCTGCGGGTAAATAGCTGCGCCGATGGTTTCTACAAAGAT
CGTTATGTTTATCGGCACTTTGCATCGGCCGCGCTCCCGATTCCGGAAGTGCTTGACATTGGGGAGTTCA
GCGAGAGCCTGACCTATTGCATCTCCCGCCGTGCACAGGGTGTCACGTTGCAAGACCTGCCTGAAACCGA
ACTGCCCGCTGTTCTTCAGCCGGTCGCGGAGGCTATGGATGCTATCGCTGCGGCCGATCTTAGCCAGACG
AGCGGGTTCGGCCCATTCGGACCGCAAGGAATCGGTCAATACACTACATGGCGTGATTTCATATGCGCGA
TTGCTGATCCCCATGTGTATCACTGGCAAACTGTGATGGACGACACCGTCAGTGCGTCCGTCGCGCAGGC
TCTCGATGAGCTGATGCTTTGGGCGAGGACTGCCCCGAAGTCCGGCACTCGTGCACGCGGATTCGGCTCC
AACAATGTCCTGACGGACAATGGCCGCATAACAGCGGTCATTGACTGGAGCGAGGCGATGTTCGGGGATT
CCCAATACGAGGTCGCCAACATCTTCTTCTGGAGGCCGTGGTTGGCTTGTATGGAGCAGCAGACGCGCTA
CTTCGAGCGGAGGCATCCGGAGCTTGCAGGATCGCCAAGGCTCCGGGCGTATATGCTCCGCATTGGTCTT
GACCAACTCTATCAGAGCTTGGTTGACGGCAATTTCGATGATGCAGCTTGGGCGCAGGGTCGATGCGACG
CAATCGTCCGATCCGGAGCCGGGACTGTCGGGCGTACACAAATCGCCCGCAGAAGCGCGGCCGTCTGGAC
CGATGGCTGTGTAGAAGTACTCGCCGATAGTGGAAACCGACGCCCCAGCACTCGTCCGAGGGCAAAGGAA
TAGAGTAGATGCCGACCGAACAAGAGCTGATTTCGAGAACGCCTCAGCCAGCAACTCGCGCGAGCCTAGC
AAGGCAAATGCGAGAGAACGGCCTTACGCTTGGTGGCACAGTTCTCGTCCACAGTTCGCTAAGCTCGCTC
GGCTGGGTCGCGGGAGGGCCGGTCGCAGTGATTCAGGAATTAATTCCCTAGAGTCAAGCAGATCGTTCAA
ACATTTGGCAATAAAGTTTCTTAAGATTGAATCCTGTTGCCGGTCTTGCGATGATTATCATATAATTTCT
GTTGAATTACGTTAAGCATGTAATAATTAACATGTAATGCATGACGTTATTTATGAGATGGGTTTTTATG
ATTAGAGTCCCGCAATTATACATTTAATACGCGATAGAAAACAAAATATAGCGCGCAAACTAGGATAAAT
TATCGCGCGCGGTGTCATCTATGTTACTAGATCGACCGGCATGCAAGCTGATAATTCAATTCGGCGTTAA
TTCAGTACATTAAAAACGTCCGCAATGTGTTATTAAGTTGTCTAAGCGTCAATTTGTTTACACCACAATA
TATCCTGCCACCAGCCAGCCAACAGCTCCCCGACCGGCAGCTCGGCACAAAATCACCACTCGATACAGGC
AGCCCATCAGTCCGGGACGGCGTCAGCGGGAGAGCCGTTGTAAGGCGGCAGACTTTGCTCATGTTACCGA
TGCTATTCGGAAGAACGGCAACTAAGCTGCCGGGTTTGAAACACGGATGATCTCGCGGAGGGTAGCATGT
TGATTGTAACGATGACAGAGCGTTGCTGCCTGTGATCAATTCGGGCACGAACCCAGTGGACATAAGCCTC
GTTCGGTTCGTAAGCTGTAATGCAAGTAGCGTAACTGCCGTCACGCAACTGGTCCAGAACCTTGACCGAA
CGCAGCGGTGGTAACGGCGCAGTGGCGGTTTTCATGGCTTCTTGTTATGACATGTTTTTTTGGGGTACAG
TCTATGCCTCGGGCATCCAAGCAGCAAGCGCGTTACGCCGTGGGTCGATGTTTGATGTTATGGAGCAGCA

ACGATGTTACGCAGCAGGGCAGTCGCCCTAAAACAAAGTTAAACATCATGGGGGAAGCGGTGATCGCCGA
AGTATCGACTCAACTATCAGAGGTAGTTGGCGTCATCGAGCGCCATCTCGAACCGACGTTGCTGGCCGTA
CATTTGTACGGCTCCGCAGTGGATGGCGGCCTGAAGCCACACAGTGATATTGATTTGCTGGTTACGGTGA
CCGTAAGGCTTGATGAAACAACGCGGCGAGCTTTGATCAACGACCTTTTGGAAACTTCGGCTTCCCCTGG
AGAGAGCGAGATTCTCCGCGCTGTAGAAGTCACCATTGTTGTGCACGACGACATCATTCCGTGGCGTTAT
CCAGCTAAGCGCGAACTGCAATTTGGAGAATGGCAGCGCAATGACATTCTTGCAGGTATCTTCGAGCCAG
CCACGATCGACATTGATCTGGCTATCTTGCTGACAAAAGCAAGAGAACATAGCGTTGCCTTGGTAGGTCC
AGCGGCGGAGGAACTCTTTGATCCGGTTCCTGAACAGGATCTATTTGAGGCGCTAAATGAAACCTTAACG
CTATGGAACTCGCCGCCCGACTGGGCTGGCGATGAGCGAAATGTAGTGCTTACGTTGTCCCGCATTTGGT
ACAGCGCAGTAACCGGCAAAATCGCGCCGAAGGATGTCGCTGCCGACTGGGCAATGGAGCGCCTGCCGGC
CCAGTATCAGCCCGTCATACTTGAAGCTAGACAGGCTTATCTTGGACAAGAAGAAGATCGCTTGGCCTCG
CGCGCAGATCAGTTGGAAGAATTTGTCCACTACGTGAAAGGCGAGATCACCAAGGTAGTCGGCAAATAAT
GTCTAGCTAGAAATTCGTTCAAGCCGACGCCGCTTCGCCGGCGTTAACTCAAGCGATTAGATGCACTAAG
CACATAATTGCTCACAGCCAAACTATCAGGTCAAGTCTGCTTTTATTATTTTTAAGCGTGCATAATAAGC
CCTACACAAATTGGGAGATATATCATGCATGACCAAAATCCCTTAACGTGAGTTTTCGTTCCACTGAGCG
TCAGACCCCGTAGAAAAGATCAAAGGATCTTCTTGAGATCCTTTTTTTCTGCGCGTAATCTGCTGCTTGC
AAACAAAAAAACCACCGCTACCAGCGGTGGTTTGTTTGCCGGATCAAGAGCTACCAACTCTTTTTCCGAA
GGTAACTGGCTTCAGCAGAGCGCAGATACCAAATACTGTCCTTCTAGTGTAGCCGTAGTTAGGCCACCAC
TTCAAGAACTCTGTAGCACCGCCTACATACCTCGCTCTGCTAATCCTGTTACCAGTGGCTGCTGCCAGTG
GCGATAAGTCGTGTCTTACCGGGTTGGACTCAAGACGATAGTTACCGGATAAGGCGCAGCGGTCGGGCTG
AACGGGGGGTTCGTGCACACAGCCCAGCTTGGAGCGAACGACCTACACCGAACTGAGATACCTACAGCGT
GAGCTATGAGAAAGCGCCACGCTTCCCGAAGGGAGAAAGGCGGACAGGTATCCGGTAAGCGGCAGGGTCG
GAACAGGAGAGCGCACGAGGGAGCTTCCAGGGGGAAACGCCTGGTATCTTTATAGTCCTGTCGGGTTTCG
CCACCTCTGACTTGAGCGTCGATTTTTGTGATGCTCGTCAGGGGGGCGGAGCCTATGGAAAAACGCCAGC
AACGCGGCCTTTTTACGGTTCCTGGCCTTTTGCTGGCCTTTTGCTCACATGTTCTTTCCTGCGTTATCCC
CTGATTCTGTGGATAACCGTATTACCGCCTTTGAGTGAGCTGATACCGCTCGCCGCAGCCGAACGACCGA
GCGCAGCGAGTCAGTGAGCGAGGAAGCGGAAGAGCGCCTGATGCGGTATTTTCTCCTTACGCATCTGTGC
GGTATTTCACACCGCATATGGTGCACTCTCAGTACAATCTGCTCTGATGCCGCATAGTTAAGCCAGTATA
CACTCCGCTATCGCTACGTGACTGGGTCATGGCTGCGCCCCGACACCCGCCAACACCCGCTGACGCGCCC
TGACGGGCTTGTCTGCTCCCGGCATCCGCTTACAGACAAGCTGTGACCGTCTCCGGGAGCTGCATGTGTC
AGAGGTTTTCACCGTCATCACCGAAACGCGCGAGGCAGGGTGCCTTGATGTGGGCGCCGGCGGTCGAGTG
GCGACGGCGCGGCTTGTCCGCGCCCTGGTAGATTGCCTGGCCGTAGGCCAGCCATTTTTGAGCGGCCAGC
GGCCGCGATAGGCCGACGCGAAGCGGCGGGGCGTAGGGAGCGCAGCGACCGAAGGGTAGGCGCTTTTTGC
AGCTCTTCGGCTGTGCGCTGGCCAGACAGTTATGCACAGGCCAGGCGGGTTTTAAGAGTTTTAATAAGTT
TTAAAGAGTTTTAGGCGGAAAAATCGCCTTTTTTCTCTTTTATATCAGTCACTTACATGTGTGACCGGTT
CCCAATGTACGGCTTTGGGTTCCCAATGTACGGGTTCCGGTTCCCAATGTACGGCTTTGGGTTCCCAATG
TACGTGCTATCCACAGGAAAGAGACCTTTTCGACCTTTTTCCCCTGCTAGGGCAATTTGCCCTAGCATCT
GCTCCGTACATTAGGAACCGGCGGATGCTTCGCCCTCGATCAGGTTGCGGTAGCGCATGACTAGGATCGG
GCCAGCCTGCCCCGCCTCCTCCTTCAAATCGTACTCCGGCAGGTCATTTGACCCGATCAGCTTGCGCACG
GTGAAACAGAACTTCTTGAACTCTCCGGCGCTGCCACTGCGTTCGTAGATCGTCTTGAACAACCATCTGG
CTTCTGCCTTGCCTGCGGCGCGGCGTGCCAGGCGGTAGAGAAAACGGCCGATGCCGGGATCGATCAAAAA
GTAATCGGGGTGAACCGTCAGCACGTCCGGGTTCTTGCCTTCTGTGATCTCGCGGTACATCCAATCAGCT
AGCTCGATCTCGATGTACTCCGGCCGCCCGGTTTCGCTCTTTACGATCTTGTAGCGGCTAATCAAGGCTT
CACCCTCGGATACCGTCACCAGGCGGCCGTTCTTGGCCTTCTTCGTACGCTGCATGGCAACGTGCGTGGT

GTTTAACCGAATGCAGGTTTCTACCAGGTCGTCTTTCTGCTTTCCGCCATCGGCTCGCCGGCAGAACTTG
AGTACGTCCGCAACGTGTGGACGGAACACGCGGCCGGGCTTGTCTCCCTTCCCTTCCCGGTATCGGTTCA
TGGATTCGGTTAGATGGGAAACCGCCATCAGTACCAGGTCGTAATCCCACACACTGGCCATGCCGGCCGG
CCCTGCGGAAACCTCTACGTGCCCGTCTGGAAGCTCGTAGCGGATCACCTCGCCAGCTCGTCGGTCACGC
TTCGACAGACGGAAAACGGCCACGTCCATGATGCTGCGACTATCGCGGGTGCCCACGTCATAGAGCATCG
GAACGAAAAAATCTGGTTGCTCGTCGCCCTTGGGCGGCTTCCTAATCGACGGCGCACCGGCTGCCGGCGG
TTGCCGGGATTCTTTGCGGATTCGATCAGCGGCCGCTTGCCACGATTCACCGGGGCGTGCTTCTGCCTCG
ATGCGTTGCCGCTGGGCGGCCTGCGCGGCCTTCAACTTCTCCACCAGGTCATCACCCAGCGCCGCGCCGA
TTTGTACCGGGCCGGATGGTTTGCGACCGTCACGCCGATTCCTCGGGCTTGGGGGTTCCAGTGCCATTGC
AGGGCCGGCAGACAACCCAGCCGCTTACGCCTGGCCAACCGCCCGTTCCTCCACACATGGGGCATTCCAC
GGCGTCGGTGCCTGGTTGTTCTTGATTTTCCATGCCGCCTCCTTTAGCCGCTAAAATTCATCTACTCATT
TATTCATTTGCTCATTTACTCTGGTAGCTGCGCGATGTATTCAGATAGCAGCTCGGTAATGGTCTTGCCT
TGGCGTACCGCGTACATCTTCAGCTTGGTGTGATCCTCCGCCGGCAACTGAAAGTTGACCCGCTTCATGG
CTGGCGTGTCTGCCAGGCTGGCCAACGTTGCAGCCTTGCTGCTGCGTGCGCTCGGACGGCCGGCACTTAG
CGTGTTTGTGCTTTTGCTCATTTTCTCTTTACCTCATTAACTCAAATGAGTTTTGATTTAATTTCAGCGG
CCAGCGCCTGGACCTCGCGGGCAGCGTCGCCCTCGGGTTCTGATTCAAGAACGGTTGTGCCGGCGGCGGC
AGTGCCTGGGTAGCTCACGCGCTGCGTGATACGGGACTCAAGAATGGGCAGCTCGTACCCGGCCAGCGCC
TCGGCAACCTCACCGCCGATGCGCGTGCCTTTGATCGCCCGCGACACGACAAAGGCCGCTTGTAGCCTTC
CATCCGTGACCTCAATGCGCTGCTTAACCAGCTCCACCAGGTCGGCGGTGGCCCATATGTCGTAAGGGCT
TGGCTGCACCGGAATCAGCACGAAGTCGGCTGCCTTGATCGCGGACACAGCCAAGTCCGCCGCCTGGGGC
GCTCCGTCGATCACTACGAAGTCGCGCCGGCCGATGGCCTTCACGTCGCGGTCAATCGTCGGGCGGTCGA
TGCCGACAACGGTTAGCGGTTGATCTTCCCGCACGGCCGCCCAATCGCGGGCACTGCCCTGGGGATCGGA
ATCGACTAACAGAACATCGGCCCCGGCGAGTTGCAGGGCGCGGGCTAGATGGGTTGCGATGGTCGTCTTG
CCTGACCCGCCTTTCTGGTTAAGTACAGCGATAACCTTCATGCGTTCCCCTTGCGTATTTGTTTATTTAC
TCATCGCATCATATACGCAGCGACCGCATGACGCAAGCTGTTTTACTCAAATACACATCACCTTTTTAGA
CGGCGGCGCTCGGTTTCTTCAGCGGCCAAGCTGGCCGGCCAGGCCGCCAGCTTGGCATCAGACAAACCGG
CCAGGATTTCATGCAGCCGCACGGTTGAGACGTGCGCGGGCGGCTCGAACACGTACCCGGCCGCGATCAT
CTCCGCCTCGATCTCTTCGGTAATGAAAAACGGTTCGTCCTGGCCGTCCTGGTGCGGTTTCATGCTTGTT
CCTCTTGGCGTTCATTCTCGGCGGCCGCCAGGGCGTCGGCCTCGGTCAATGCGTCCTCACGGAAGGCACC
GCGCCGCCTGGCCTCGGTGGGCGTCACTTCCTCGCTGCGCTCAAGTGCGCGGTACAGGGTCGAGCGATGC
ACGCCAAGCAGTGCAGCCGCCTCTTTCACGGTGCGGCCTTCCTGGTCGATCAGCTCGCGGGCGTGCGCGA
TCTGTGCCGGGGTGAGGGTAGGGCGGGGGCCAAACTTCACGCCTCGGGCCTTGGCGGCCTCGCGCCCGCT
CCGGGTGCGGTCGATGATTAGGGAACGCTCGAACTCGGCAATGCCGGCGAACACGGTCAACACCATGCGG
CCGGCCGGCGTGGTGGTGTCGGCCCACGGCTCTGCCAGGCTACGCAGGCCCGCGCCGGCCTCCTGGATGC
GCTCGGCAATGTCCAGTAGGTCGCGGGTGCTGCGGGCCAGGCGGTCTAGCCTGGTCACTGTCACAACGTC
GCCAGGGCGTAGGTGGTCAAGCATCCTGGCCAGCTCCGGGCGGTCGCGCCTGGTGCCGGTGATCTTCTCG
GAAAACAGCTTGGTGCAGCCGGCCGCGTGCAGTTCGGCCCGTTGGTTGGTCAAGTCCTGGTCGTCGGTGC
TGACGCGGGCATAGCCCAGCAGGCCAGCGGCGGCGCTCTTGTTCATGGCGTAATGTCTCCGGTTCTAGTC
GCAAGTATTCTACTTTATGCGACTAAAACACGCGACAAGAAAACGCCAGGAAAAGGGCAGGGCGGCAGCC
TGTCGCGTAACTTAGGACTTGTGCGACATGTCGTTTTCAGAAGACGGCTGCACTGAACGTCAGAAGCCGA
CTGCACTATAGCAGCGGAGGGGTTGGATCAAAGTACTTTGATCCCGAGGGGAACCCTGTGGTTGGCATGC
ACATACAAATGGACGAACGGATAAACCTTTTCACGCCCTTTTAAATATCCGTTATTCTAATAAACGCTCT
TTTCTCTTAGGTTTACCCGCCAATATATCCTGTCAAACACTGATAGTTTAAACTGAAGGCGGGAAACGAC
AATCTGATCCAAGCTCAAGCTAAGCTTGAGCTCTGCATGCCTGCAGGTCCCCAGATTAGCCTTTTCAATT

TCAGAAAGAATGCTAACCCACAGATGGTTAGAGAGGCTTACGCAGCAGGTCTCATCAAGACGATCTACCC
GAGCAATAATCTCCAGGAAATCAAATACCTTCCCAAGAAGGTTAAAGATGCAGTCAAAAGATTCAGGACT
AACTGCATCAAGAACACAGAGAAAGATATATTTCTCAAGATCAGAAGTACTATTCCAGTATGGACGATTC
AAGGCTTGCTTCACAAACCAAGGCAAGTAATAGAGATTGGAGTCTCTAAAAAGGTAGTTCCCACTGAATC
AAAGGCCATGGAGTCAAAGATTCAAATAGAGGACCTAACAGAACTCGCCGTAAAGACTGGCGAACAGTTC
ATACAGAGTCTCTTACGACTCAATGACAAGAAGAAAATCTTCGTCAACATGGTGGAGCACGACACACTTG
TCTACTCCAAAAATATCAAAGATACAGTCTCAGAAGACCAAAGGGCAATTGAGACTTTTCAACAAAGGGT
AATATCCGGAAACCTCCTCGGATTCCATTGCCCAGCTATCTGTCACTTTATTGTGAAGATAGTGGAAAAG
GAAGGTGGCTCCTACAAATGCCATCATTGCGATAAAGGAAAGGCCATCGTTGAAGATGCCTCTGCCGACA
GTGGTCCCAAAGATGGACCCCCACCCACGAGGAGCATCGTGGAAAAAGAAGACGTTCCAACCACGTCTTC
AAAGCAAGTGGATTGATGTGATATCTCCACTGACGTAAGGGATGACGCACAATCCCACTATCCTTCGCAA
GACCCTTCCTCTATATAAGGAAGTTCATTTCATTTGGAGAGAACACGGGGGACTCTAGAGGATCCAAGGA
GATATAAGCCGCCACCATGGATTACAAGGACGATGATGACAAGGACTACAAGGACGATGACGATAAGGAC
TATAAGGATGACGATGACAAGGATTACGCCGAGGATGGATGATACTAGCGGACAAACTTTCGTTTCTGTG
AACCTTTCTGACGAAAGCAACACGGCAAGCACTAAAGTTGAAAACGTAAAATCGGAAGCGGATCGCTTGG
AGTTTTTACGTAAAATGAATCCCTTTATTGTTGACGCTCTGGTGCGGAAAACCAATTATCAGGGTGCTCG
CTTTCGCGCAAGAATAATAGGAGTGTGCGTGGATTGTGGTAGAAAACACGACAAGGCGCTCAAGACTGCG
CGTAAGTGTAAGGTCAACAACACGCAATCTCAGAACGAGGTGGCGCATATGTTGATGCACGATCCCGTTA
AGTATTTGAACAAAAGAAAGGCTAGAGCCTTTTCTAACGCAGAGATGTTTGCGATTGAACTGGTTTCGTA
CACCAAGGAAAGGCAATTGGCGGTCGATTTAGCCGCTGAAAGGGAGAAGACGAGACTGGCTCGTAGACAC
CCAATACGTTCTCCGGAAGAAACTCCGGAACATTATAAATTCGGTATGACTGCTAAGGCAATGTTACCGG
ACATCAACGCCGTAGACGTTGGTGATAACGAGGAAACTTCGTCGGAGTACCCAGTGAGTCTGAGTGTTTC
TGGCGGAGTTCTCCGTGAACACCACTTCATCTGACCTCTTCCCTAATGATATCCCGCGGCCATGCTAGAG
TCCGCAAAAATCACCAGTCTCTCTCTACAAATCTATCTCTCTCTATTTTTCTCCAGAATAATGTGTGAGT
AGTTCCCAGATAAGGGAATTAGGGTTCTTATAGGGTTTCGCTCATGTGTTGAGCATATAAGAAACCCTTA
GTATGTATTTGTATTTGTAAAATACTTCTATCAATAAAATTTCTAATTCCTAAAACCAAAATCCAGTGAC
CTGCAGGCATGCGACGTCG

Sequence of pH7LIC3.1.1-p23M3 (35S- p23M3)

GGGTACCCTCGAATTATCATACATGAGAATTAAGGGAGTCACGTTATGACCCCGCCGATGACGCGGGACA
AGCCGTTTTACGTTTGGAACTGACAGAACCGCAACGTTGAAGGAGCCACTGAGCCGCGGGTTTCTGGAGT
TTAATGAGCTAAGCACATACGTCAGAAACCATTATTGCGCGTTCAAAAGTCGCCTAAGGTCACTATCAGC
TAGCAAATATTTCTTGTCAAAAATGCTCCACTGACGTTCCATAAATTCCCCTCGGTATCCAATTAGAGTC
TCATATTCACTCTCAACTCGAATCCCCCCTATGAAAAAGCCTGAACTCACCGCGACGTCTGTCGAGAAGT
TTCTGATCGAAAAGTTCGACAGCGTCTCCGACCTGATGCAGCTCTCGGAGGGCGAAGAATCTCGTGCTTT
CAGCTTCGATGTAGGAGGGCGTGGATATGTCCTGCGGGTAAATAGCTGCGCCGATGGTTTCTACAAAGAT
CGTTATGTTTATCGGCACTTTGCATCGGCCGCGCTCCCGATTCCGGAAGTGCTTGACATTGGGGAGTTCA
GCGAGAGCCTGACCTATTGCATCTCCCGCCGTGCACAGGGTGTCACGTTGCAAGACCTGCCTGAAACCGA
ACTGCCCGCTGTTCTTCAGCCGGTCGCGGAGGCTATGGATGCTATCGCTGCGGCCGATCTTAGCCAGACG
AGCGGGTTCGGCCCATTCGGACCGCAAGGAATCGGTCAATACACTACATGGCGTGATTTCATATGCGCGA
TTGCTGATCCCCATGTGTATCACTGGCAAACTGTGATGGACGACACCGTCAGTGCGTCCGTCGCGCAGGC
TCTCGATGAGCTGATGCTTTGGGCGAGGACTGCCCCGAAGTCCGGCACTCGTGCACGCGGATTCGGCTCC
AACAATGTCCTGACGGACAATGGCCGCATAACAGCGGTCATTGACTGGAGCGAGGCGATGTTCGGGGATT
CCCAATACGAGGTCGCCAACATCTTCTTCTGGAGGCCGTGGTTGGCTTGTATGGAGCAGCAGACGCGCTA

CTTCGAGCGGAGGCATCCGGAGCTTGCAGGATCGCCAAGGCTCCGGGCGTATATGCTCCGCATTGGTCTT
GACCAACTCTATCAGAGCTTGGTTGACGGCAATTTCGATGATGCAGCTTGGGCGCAGGGTCGATGCGACG
CAATCGTCCGATCCGGAGCCGGGACTGTCGGGCGTACACAAATCGCCCGCAGAAGCGCGGCCGTCTGGAC
CGATGGCTGTGTAGAAGTACTCGCCGATAGTGGAAACCGACGCCCCAGCACTCGTCCGAGGGCAAAGGAA
TAGAGTAGATGCCGACCGAACAAGAGCTGATTTCGAGAACGCCTCAGCCAGCAACTCGCGCGAGCCTAGC
AAGGCAAATGCGAGAGAACGGCCTTACGCTTGGTGGCACAGTTCTCGTCCACAGTTCGCTAAGCTCGCTC
GGCTGGGTCGCGGGAGGGCCGGTCGCAGTGATTCAGGAATTAATTCCCTAGAGTCAAGCAGATCGTTCAA
ACATTTGGCAATAAAGTTTCTTAAGATTGAATCCTGTTGCCGGTCTTGCGATGATTATCATATAATTTCT
GTTGAATTACGTTAAGCATGTAATAATTAACATGTAATGCATGACGTTATTTATGAGATGGGTTTTTATG
ATTAGAGTCCCGCAATTATACATTTAATACGCGATAGAAAACAAAATATAGCGCGCAAACTAGGATAAAT
TATCGCGCGCGGTGTCATCTATGTTACTAGATCGACCGGCATGCAAGCTGATAATTCAATTCGGCGTTAA
TTCAGTACATTAAAAACGTCCGCAATGTGTTATTAAGTTGTCTAAGCGTCAATTTGTTTACACCACAATA
TATCCTGCCACCAGCCAGCCAACAGCTCCCCGACCGGCAGCTCGGCACAAAATCACCACTCGATACAGGC
AGCCCATCAGTCCGGGACGGCGTCAGCGGGAGAGCCGTTGTAAGGCGGCAGACTTTGCTCATGTTACCGA
TGCTATTCGGAAGAACGGCAACTAAGCTGCCGGGTTTGAAACACGGATGATCTCGCGGAGGGTAGCATGT
TGATTGTAACGATGACAGAGCGTTGCTGCCTGTGATCAATTCGGGCACGAACCCAGTGGACATAAGCCTC
GTTCGGTTCGTAAGCTGTAATGCAAGTAGCGTAACTGCCGTCACGCAACTGGTCCAGAACCTTGACCGAA
CGCAGCGGTGGTAACGGCGCAGTGGCGGTTTTCATGGCTTCTTGTTATGACATGTTTTTTTGGGGTACAG
TCTATGCCTCGGGCATCCAAGCAGCAAGCGCGTTACGCCGTGGGTCGATGTTTGATGTTATGGAGCAGCA
ACGATGTTACGCAGCAGGGCAGTCGCCCTAAAACAAAGTTAAACATCATGGGGGAAGCGGTGATCGCCGA
AGTATCGACTCAACTATCAGAGGTAGTTGGCGTCATCGAGCGCCATCTCGAACCGACGTTGCTGGCCGTA
CATTTGTACGGCTCCGCAGTGGATGGCGGCCTGAAGCCACACAGTGATATTGATTTGCTGGTTACGGTGA
CCGTAAGGCTTGATGAAACAACGCGGCGAGCTTTGATCAACGACCTTTTGGAAACTTCGGCTTCCCCTGG
AGAGAGCGAGATTCTCCGCGCTGTAGAAGTCACCATTGTTGTGCACGACGACATCATTCCGTGGCGTTAT
CCAGCTAAGCGCGAACTGCAATTTGGAGAATGGCAGCGCAATGACATTCTTGCAGGTATCTTCGAGCCAG
CCACGATCGACATTGATCTGGCTATCTTGCTGACAAAAGCAAGAGAACATAGCGTTGCCTTGGTAGGTCC
AGCGGCGGAGGAACTCTTTGATCCGGTTCCTGAACAGGATCTATTTGAGGCGCTAAATGAAACCTTAACG
CTATGGAACTCGCCGCCCGACTGGGCTGGCGATGAGCGAAATGTAGTGCTTACGTTGTCCCGCATTTGGT
ACAGCGCAGTAACCGGCAAAATCGCGCCGAAGGATGTCGCTGCCGACTGGGCAATGGAGCGCCTGCCGGC
CCAGTATCAGCCCGTCATACTTGAAGCTAGACAGGCTTATCTTGGACAAGAAGAAGATCGCTTGGCCTCG
CGCGCAGATCAGTTGGAAGAATTTGTCCACTACGTGAAAGGCGAGATCACCAAGGTAGTCGGCAAATAAT
GTCTAGCTAGAAATTCGTTCAAGCCGACGCCGCTTCGCCGGCGTTAACTCAAGCGATTAGATGCACTAAG
CACATAATTGCTCACAGCCAAACTATCAGGTCAAGTCTGCTTTTATTATTTTTAAGCGTGCATAATAAGC
CCTACACAAATTGGGAGATATATCATGCATGACCAAAATCCCTTAACGTGAGTTTTCGTTCCACTGAGCG
TCAGACCCCGTAGAAAAGATCAAAGGATCTTCTTGAGATCCTTTTTTTCTGCGCGTAATCTGCTGCTTGC
AAACAAAAAAACCACCGCTACCAGCGGTGGTTTGTTTGCCGGATCAAGAGCTACCAACTCTTTTTCCGAA
GGTAACTGGCTTCAGCAGAGCGCAGATACCAAATACTGTCCTTCTAGTGTAGCCGTAGTTAGGCCACCAC
TTCAAGAACTCTGTAGCACCGCCTACATACCTCGCTCTGCTAATCCTGTTACCAGTGGCTGCTGCCAGTG
GCGATAAGTCGTGTCTTACCGGGTTGGACTCAAGACGATAGTTACCGGATAAGGCGCAGCGGTCGGGCTG
AACGGGGGGTTCGTGCACACAGCCCAGCTTGGAGCGAACGACCTACACCGAACTGAGATACCTACAGCGT
GAGCTATGAGAAAGCGCCACGCTTCCCGAAGGGAGAAAGGCGGACAGGTATCCGGTAAGCGGCAGGGTCG
GAACAGGAGAGCGCACGAGGGAGCTTCCAGGGGGAAACGCCTGGTATCTTTATAGTCCTGTCGGGTTTCG
CCACCTCTGACTTGAGCGTCGATTTTTGTGATGCTCGTCAGGGGGGCGGAGCCTATGGAAAAACGCCAGC
AACGCGGCCTTTTTACGGTTCCTGGCCTTTTGCTGGCCTTTTGCTCACATGTTCTTTCCTGCGTTATCCC

CTGATTCTGTGGATAACCGTATTACCGCCTTTGAGTGAGCTGATACCGCTCGCCGCAGCCGAACGACCGA
GCGCAGCGAGTCAGTGAGCGAGGAAGCGGAAGAGCGCCTGATGCGGTATTTTCTCCTTACGCATCTGTGC
GGTATTTCACACCGCATATGGTGCACTCTCAGTACAATCTGCTCTGATGCCGCATAGTTAAGCCAGTATA
CACTCCGCTATCGCTACGTGACTGGGTCATGGCTGCGCCCCGACACCCGCCAACACCCGCTGACGCGCCC
TGACGGGCTTGTCTGCTCCCGGCATCCGCTTACAGACAAGCTGTGACCGTCTCCGGGAGCTGCATGTGTC
AGAGGTTTTCACCGTCATCACCGAAACGCGCGAGGCAGGGTGCCTTGATGTGGGCGCCGGCGGTCGAGTG
GCGACGGCGCGGCTTGTCCGCGCCCTGGTAGATTGCCTGGCCGTAGGCCAGCCATTTTTGAGCGGCCAGC
GGCCGCGATAGGCCGACGCGAAGCGGCGGGGCGTAGGGAGCGCAGCGACCGAAGGGTAGGCGCTTTTTGC
AGCTCTTCGGCTGTGCGCTGGCCAGACAGTTATGCACAGGCCAGGCGGGTTTTAAGAGTTTTAATAAGTT
TTAAAGAGTTTTAGGCGGAAAAATCGCCTTTTTTCTCTTTTATATCAGTCACTTACATGTGTGACCGGTT
CCCAATGTACGGCTTTGGGTTCCCAATGTACGGGTTCCGGTTCCCAATGTACGGCTTTGGGTTCCCAATG
TACGTGCTATCCACAGGAAAGAGACCTTTTCGACCTTTTTCCCCTGCTAGGGCAATTTGCCCTAGCATCT
GCTCCGTACATTAGGAACCGGCGGATGCTTCGCCCTCGATCAGGTTGCGGTAGCGCATGACTAGGATCGG
GCCAGCCTGCCCCGCCTCCTCCTTCAAATCGTACTCCGGCAGGTCATTTGACCCGATCAGCTTGCGCACG
GTGAAACAGAACTTCTTGAACTCTCCGGCGCTGCCACTGCGTTCGTAGATCGTCTTGAACAACCATCTGG
CTTCTGCCTTGCCTGCGGCGCGGCGTGCCAGGCGGTAGAGAAAACGGCCGATGCCGGGATCGATCAAAAA
GTAATCGGGGTGAACCGTCAGCACGTCCGGGTTCTTGCCTTCTGTGATCTCGCGGTACATCCAATCAGCT
AGCTCGATCTCGATGTACTCCGGCCGCCCGGTTTCGCTCTTTACGATCTTGTAGCGGCTAATCAAGGCTT
CACCCTCGGATACCGTCACCAGGCGGCCGTTCTTGGCCTTCTTCGTACGCTGCATGGCAACGTGCGTGGT
GTTTAACCGAATGCAGGTTTCTACCAGGTCGTCTTTCTGCTTTCCGCCATCGGCTCGCCGGCAGAACTTG
AGTACGTCCGCAACGTGTGGACGGAACACGCGGCCGGGCTTGTCTCCCTTCCCTTCCCGGTATCGGTTCA
TGGATTCGGTTAGATGGGAAACCGCCATCAGTACCAGGTCGTAATCCCACACACTGGCCATGCCGGCCGG
CCCTGCGGAAACCTCTACGTGCCCGTCTGGAAGCTCGTAGCGGATCACCTCGCCAGCTCGTCGGTCACGC
TTCGACAGACGGAAAACGGCCACGTCCATGATGCTGCGACTATCGCGGGTGCCCACGTCATAGAGCATCG
GAACGAAAAAATCTGGTTGCTCGTCGCCCTTGGGCGGCTTCCTAATCGACGGCGCACCGGCTGCCGGCGG
TTGCCGGGATTCTTTGCGGATTCGATCAGCGGCCGCTTGCCACGATTCACCGGGGCGTGCTTCTGCCTCG
ATGCGTTGCCGCTGGGCGGCCTGCGCGGCCTTCAACTTCTCCACCAGGTCATCACCCAGCGCCGCGCCGA
TTTGTACCGGGCCGGATGGTTTGCGACCGTCACGCCGATTCCTCGGGCTTGGGGGTTCCAGTGCCATTGC
AGGGCCGGCAGACAACCCAGCCGCTTACGCCTGGCCAACCGCCCGTTCCTCCACACATGGGGCATTCCAC
GGCGTCGGTGCCTGGTTGTTCTTGATTTTCCATGCCGCCTCCTTTAGCCGCTAAAATTCATCTACTCATT
TATTCATTTGCTCATTTACTCTGGTAGCTGCGCGATGTATTCAGATAGCAGCTCGGTAATGGTCTTGCCT
TGGCGTACCGCGTACATCTTCAGCTTGGTGTGATCCTCCGCCGGCAACTGAAAGTTGACCCGCTTCATGG
CTGGCGTGTCTGCCAGGCTGGCCAACGTTGCAGCCTTGCTGCTGCGTGCGCTCGGACGGCCGGCACTTAG
CGTGTTTGTGCTTTTGCTCATTTTCTCTTTACCTCATTAACTCAAATGAGTTTTGATTTAATTTCAGCGG
CCAGCGCCTGGACCTCGCGGGCAGCGTCGCCCTCGGGTTCTGATTCAAGAACGGTTGTGCCGGCGGCGGC
AGTGCCTGGGTAGCTCACGCGCTGCGTGATACGGGACTCAAGAATGGGCAGCTCGTACCCGGCCAGCGCC
TCGGCAACCTCACCGCCGATGCGCGTGCCTTTGATCGCCCGCGACACGACAAAGGCCGCTTGTAGCCTTC
CATCCGTGACCTCAATGCGCTGCTTAACCAGCTCCACCAGGTCGGCGGTGGCCCATATGTCGTAAGGGCT
TGGCTGCACCGGAATCAGCACGAAGTCGGCTGCCTTGATCGCGGACACAGCCAAGTCCGCCGCCTGGGGC
GCTCCGTCGATCACTACGAAGTCGCGCCGGCCGATGGCCTTCACGTCGCGGTCAATCGTCGGGCGGTCGA
TGCCGACAACGGTTAGCGGTTGATCTTCCCGCACGGCCGCCCAATCGCGGGCACTGCCCTGGGGATCGGA
ATCGACTAACAGAACATCGGCCCCGGCGAGTTGCAGGGCGCGGGCTAGATGGGTTGCGATGGTCGTCTTG
CCTGACCCGCCTTTCTGGTTAAGTACAGCGATAACCTTCATGCGTTCCCCTTGCGTATTTGTTTATTTAC
TCATCGCATCATATACGCAGCGACCGCATGACGCAAGCTGTTTTACTCAAATACACATCACCTTTTTAGA

CGGCGGCGCTCGGTTTCTTCAGCGGCCAAGCTGGCCGGCCAGGCCGCCAGCTTGGCATCAGACAAACCGG
CCAGGATTTCATGCAGCCGCACGGTTGAGACGTGCGCGGGCGGCTCGAACACGTACCCGGCCGCGATCAT
CTCCGCCTCGATCTCTTCGGTAATGAAAAACGGTTCGTCCTGGCCGTCCTGGTGCGGTTTCATGCTTGTT
CCTCTTGGCGTTCATTCTCGGCGGCCGCCAGGGCGTCGGCCTCGGTCAATGCGTCCTCACGGAAGGCACC
GCGCCGCCTGGCCTCGGTGGGCGTCACTTCCTCGCTGCGCTCAAGTGCGCGGTACAGGGTCGAGCGATGC
ACGCCAAGCAGTGCAGCCGCCTCTTTCACGGTGCGGCCTTCCTGGTCGATCAGCTCGCGGGCGTGCGCGA
TCTGTGCCGGGGTGAGGGTAGGGCGGGGGCCAAACTTCACGCCTCGGGCCTTGGCGGCCTCGCGCCCGCT
CCGGGTGCGGTCGATGATTAGGGAACGCTCGAACTCGGCAATGCCGGCGAACACGGTCAACACCATGCGG
CCGGCCGGCGTGGTGGTGTCGGCCCACGGCTCTGCCAGGCTACGCAGGCCCGCGCCGGCCTCCTGGATGC
GCTCGGCAATGTCCAGTAGGTCGCGGGTGCTGCGGGCCAGGCGGTCTAGCCTGGTCACTGTCACAACGTC
GCCAGGGCGTAGGTGGTCAAGCATCCTGGCCAGCTCCGGGCGGTCGCGCCTGGTGCCGGTGATCTTCTCG
GAAAACAGCTTGGTGCAGCCGGCCGCGTGCAGTTCGGCCCGTTGGTTGGTCAAGTCCTGGTCGTCGGTGC
TGACGCGGGCATAGCCCAGCAGGCCAGCGGCGGCGCTCTTGTTCATGGCGTAATGTCTCCGGTTCTAGTC
GCAAGTATTCTACTTTATGCGACTAAAACACGCGACAAGAAAACGCCAGGAAAAGGGCAGGGCGGCAGCC
TGTCGCGTAACTTAGGACTTGTGCGACATGTCGTTTTCAGAAGACGGCTGCACTGAACGTCAGAAGCCGA
CTGCACTATAGCAGCGGAGGGGTTGGATCAAAGTACTTTGATCCCGAGGGGAACCCTGTGGTTGGCATGC
ACATACAAATGGACGAACGGATAAACCTTTTCACGCCCTTTTAAATATCCGTTATTCTAATAAACGCTCT
TTTCTCTTAGGTTTACCCGCCAATATATCCTGTCAAACACTGATAGTTTAAACTGAAGGCGGGAAACGAC
AATCTGATCCAAGCTCAAGCTAAGCTTGAGCTCTGCATGCCTGCAGGTCCCCAGATTAGCCTTTTCAATT
TCAGAAAGAATGCTAACCCACAGATGGTTAGAGAGGCTTACGCAGCAGGTCTCATCAAGACGATCTACCC
GAGCAATAATCTCCAGGAAATCAAATACCTTCCCAAGAAGGTTAAAGATGCAGTCAAAAGATTCAGGACT
AACTGCATCAAGAACACAGAGAAAGATATATTTCTCAAGATCAGAAGTACTATTCCAGTATGGACGATTC
AAGGCTTGCTTCACAAACCAAGGCAAGTAATAGAGATTGGAGTCTCTAAAAAGGTAGTTCCCACTGAATC
AAAGGCCATGGAGTCAAAGATTCAAATAGAGGACCTAACAGAACTCGCCGTAAAGACTGGCGAACAGTTC
ATACAGAGTCTCTTACGACTCAATGACAAGAAGAAAATCTTCGTCAACATGGTGGAGCACGACACACTTG
TCTACTCCAAAAATATCAAAGATACAGTCTCAGAAGACCAAAGGGCAATTGAGACTTTTCAACAAAGGGT
AATATCCGGAAACCTCCTCGGATTCCATTGCCCAGCTATCTGTCACTTTATTGTGAAGATAGTGGAAAAG
GAAGGTGGCTCCTACAAATGCCATCATTGCGATAAAGGAAAGGCCATCGTTGAAGATGCCTCTGCCGACA
GTGGTCCCAAAGATGGACCCCCACCCACGAGGAGCATCGTGGAAAAAGAAGACGTTCCAACCACGTCTTC
AAAGCAAGTGGATTGATGTGATATCTCCACTGACGTAAGGGATGACGCACAATCCCACTATCCTTCGCAA
GACCCTTCCTCTATATAAGGAAGTTCATTTCATTTGGAGAGAACACGGGGGACTCTAGAGGATCCAAGGA
GATATAAGCCGCCACCATGGATTACAAGGACGATGATGACAAGGACTACAAGGACGATGACGATAAGGAC
TATAAGGATGACGATGACAAGGATTACGCCGAGGATGGATGATACTAGCGGACAAACTTTCGTTTCTGTG
AACCTTTCTGACGAAAGCAACACGGCAAGCACTAAAGTTGAAAACGTAAAATCGGAAGCGGATCGCTTGG
AGTTTTTACGTAAAATGAATCCCTTTATTGTTGACGCTCTGGTGCGGAAAACCAATTATCAGGGTGCTCG
CTTTCGCGCAAGAATAATAGGAGTGTGCGTGGATTGTGGTAGAAAACACGACAAGGCGCTCAAGACTGAA
CGTAAGTGTAAGGTCAACGCGGCGCAATCTCAGAACGAGGTGGCGCATATGTTGATGCACGATCCCGTTA
AGTATTTGAACAAAAGAAAGGCTAGAGCCTTTTCTAACGCAGAGATGTTTGCGATTGAACTGGTTTCGTA
CACCAAGGAAAGGCAATTGGCGGTCGATTTAGCCGCTGAAAGGGAGAAGACGAGACTGGCTCGTAGACAC
CCAATACGTTCTCCGGAAGAAACTCCGGAACATTATAAATTCGGTATGACTGCTAAGGCAATGTTACCGG
ACATCAACGCCGTAGACGTTGGTGATAACGAGGAAACTTCGTCGGAGTACCCAGTGAGTCTGAGTGTTTC
TGGCGGAGTTCTCCGTGAACACCACTTCATCTGACCTCTTCCCTAATGATATCCCGCGGCCATGCTAGAG
TCCGCAAAAATCACCAGTCTCTCTCTACAAATCTATCTCTCTCTATTTTTCTCCAGAATAATGTGTGAGT
AGTTCCCAGATAAGGGAATTAGGGTTCTTATAGGGTTTCGCTCATGTGTTGAGCATATAAGAAACCCTTA

GTATGTATTTGTATTTGTAAAATACTTCTATCAATAAAATTTCTAATTCCTAAAACCAAAATCCAGTGAC
CTGCAGGCATGCGACGTCG

Sequence of pH7LIC3.1.1-p23M4 (35S- p23M4)

GGGTACCCTCGAATTATCATACATGAGAATTAAGGGAGTCACGTTATGACCCCGCCGATGACGCGGGACA
AGCCGTTTTACGTTTGGAACTGACAGAACCGCAACGTTGAAGGAGCCACTGAGCCGCGGGTTTCTGGAGT
TTAATGAGCTAAGCACATACGTCAGAAACCATTATTGCGCGTTCAAAAGTCGCCTAAGGTCACTATCAGC
TAGCAAATATTTCTTGTCAAAAATGCTCCACTGACGTTCCATAAATTCCCCTCGGTATCCAATTAGAGTC
TCATATTCACTCTCAACTCGAATCCCCCTATGAAAAAGCCTGAACTCACCGCGACGTCTGTCGAGAAGT
TTCTGATCGAAAAGTTCGACAGCGTCTCCGACCTGATGCAGCTCTCGGAGGGCGAAGAATCTCGTGCTTT
CAGCTTCGATGTAGGAGGGCGTGGATATGTCCTGCGGGTAAATAGCTGCGCCGATGGTTTCTACAAAGAT
CGTTATGTTTATCGGCACTTTGCATCGGCCGCGCTCCCGATTCCGGAAGTGCTTGACATTGGGGAGTTCA
GCGAGAGCCTGACCTATTGCATCTCCCGCCGTGCACAGGGTGTCACGTTGCAAGACCTGCCTGAAACCGA
ACTGCCCGCTGTTCTTCAGCCGGTCGCGGAGGCTATGGATGCTATCGCTGCGGCCGATCTTAGCCAGACG
AGCGGGTTCGGCCCATTCGGACCGCAAGGAATCGGTCAATACACTACATGGCGTGATTTCATATGCGCGA
TTGCTGATCCCCATGTGTATCACTGGCAAACTGTGATGGACGACACCGTCAGTGCGTCCGTCGCGCAGGC
TCTCGATGAGCTGATGCTTTGGGCGAGGACTGCCCCGAAGTCCGGCACTCGTGCACGCGGATTCGGCTCC
AACAATGTCCTGACGGACAATGGCCGCATAACAGCGGTCATTGACTGGAGCGAGGCGATGTTCGGGGATT
CCCAATACGAGGTCGCCAACATCTTCTTCTGGAGGCCGTGGTTGGCTTGTATGGAGCAGCAGACGCGCTA
CTTCGAGCGGAGGCATCCGGAGCTTGCAGGATCGCCAAGGCTCCGGGCGTATATGCTCCGCATTGGTCTT
GACCAACTCTATCAGAGCTTGGTTGACGGCAATTTCGATGATGCAGCTTGGGCGCAGGGTCGATGCGACG
CAATCGTCCGATCCGGAGCCGGGACTGTCGGGCGTACACAAATCGCCCGCAGAAGCGCGGCCGTCTGGAC
CGATGGCTGTGTAGAAGTACTCGCCGATAGTGGAAACCGACGCCCCAGCACTCGTCCGAGGGCAAAGGAA
TAGAGTAGATGCCGACCGAACAAGAGCTGATTTCGAGAACGCCTCAGCCAGCAACTCGCGCGAGCCTAGC
AAGGCAAATGCGAGAGAACGGCCTTACGCTTGGTGGCACAGTTCTCGTCCACAGTTCGCTAAGCTCGCTC
GGCTGGGTCGCGGGAGGGCCGGTCGCAGTGATTCAGGAATTAATTCCCTAGAGTCAAGCAGATCGTTCAA
ACATTTGGCAATAAAGTTTCTTAAGATTGAATCCTGTTGCCGGTCTTGCGATGATTATCATATAATTTCT
GTTGAATTACGTTAAGCATGTAATAATTAACATGTAATGCATGACGTTATTTATGAGATGGGTTTTTATG
ATTAGAGTCCCGCAATTATACATTTAATACGCGATAGAAAACAAAATATAGCGCGCAAACTAGGATAAAT
TATCGCGCGCGGTGTCATCTATGTTACTAGATCGACCGGCATGCAAGCTGATAATTCAATTCGGCGTTAA
TTCAGTACATTAAAAACGTCCGCAATGTGTTATTAAGTTGTCTAAGCGTCAATTTGTTTACACCACAATA
TATCCTGCCACCAGCCAGCCAACAGCTCCCCGACCGGCAGCTCGGCACAAAATCACCACTCGATACAGGC
AGCCCATCAGTCCGGGACGGCGTCAGCGGGAGAGCCGTTGTAAGGCGGCAGACTTTGCTCATGTTACCGA
TGCTATTCGGAAGAACGGCAACTAAGCTGCCGGGTTTGAAACACGGATGATCTCGCGGAGGGTAGCATGT
TGATTGTAACGATGACAGAGCGTTGCTGCCTGTGATCAATTCGGGCACGAACCCAGTGGACATAAGCCTC
GTTCGGTTCGTAAGCTGTAATGCAAGTAGCGTAACTGCCGTCACGCAACTGGTCCAGAACCTTGACCGAA
CGCAGCGGTGGTAACGGCGCAGTGGCGGTTTTCATGGCTTCTTGTTATGACATGTTTTTTTGGGGTACAG
TCTATGCCTCGGGCATCCAAGCAGCAAGCGCGTTACGCCGTGGGTCGATGTTTGATGTTATGGAGCAGCA
ACGATGTTACGCAGCAGGGCAGTCGCCCTAAAACAAAGTTAAACATCATGGGGGAAGCGGTGATCGCCGA
AGTATCGACTCAACTATCAGAGGTAGTTGGCGTCATCGAGCGCCATCTCGAACCGACGTTGCTGGCCGTA
CATTTGTACGGCTCCGCAGTGGATGGCGGCCTGAAGCCACACAGTGATATTGATTTGCTGGTTACGGTGA
CCGTAAGGCTTGATGAAACAACGCGGCGAGCTTTGATCAACGACCTTTTGGAAACTTCGGCTTCCCCTGG
AGAGAGCGAGATTCTCCGCGCTGTAGAAGTCACCATTGTTGTGCACGACGACATCATTCCGTGGCGTTAT
CCAGCTAAGCGCGAACTGCAATTTGGAGAATGGCAGCGCAATGACATTCTTGCAGGTATCTTCGAGCCAG

CCACGATCGACATTGATCTGGCTATCTTGCTGACAAAAGCAAGAGAACATAGCGTTGCCTTGGTAGGTCC
AGCGGCGGAGGAACTCTTTGATCCGGTTCCTGAACAGGATCTATTTGAGGCGCTAAATGAAACCTTAACG
CTATGGAACTCGCCGCCCGACTGGGCTGGCGATGAGCGAAATGTAGTGCTTACGTTGTCCCGCATTTGGT
ACAGCGCAGTAACCGGCAAAATCGCGCCGAAGGATGTCGCTGCCGACTGGGCAATGGAGCGCCTGCCGGC
CCAGTATCAGCCCGTCATACTTGAAGCTAGACAGGCTTATCTTGGACAAGAAGAAGATCGCTTGGCCTCG
CGCGCAGATCAGTTGGAAGAATTTGTCCACTACGTGAAAGGCGAGATCACCAAGGTAGTCGGCAAATAAT
GTCTAGCTAGAAATTCGTTCAAGCCGACGCCGCTTCGCCGGCGTTAACTCAAGCGATTAGATGCACTAAG
CACATAATTGCTCACAGCCAAACTATCAGGTCAAGTCTGCTTTTATTATTTTTAAGCGTGCATAATAAGC
CCTACACAAATTGGGAGATATATCATGCATGACCAAAATCCCTTAACGTGAGTTTTCGTTCCACTGAGCG
TCAGACCCCGTAGAAAAGATCAAAGGATCTTCTTGAGATCCTTTTTTTCTGCGCGTAATCTGCTGCTTGC
AAACAAAAAAACCACCGCTACCAGCGGTGGTTTGTTTGCCGGATCAAGAGCTACCAACTCTTTTTCCGAA
GGTAACTGGCTTCAGCAGAGCGCAGATACCAAATACTGTCCTTCTAGTGTAGCCGTAGTTAGGCCACCAC
TTCAAGAACTCTGTAGCACCGCCTACATACCTCGCTCTGCTAATCCTGTTACCAGTGGCTGCTGCCAGTG
GCGATAAGTCGTGTCTTACCGGGTTGGACTCAAGACGATAGTTACCGGATAAGGCGCAGCGGTCGGGCTG
AACGGGGGGTTCGTGCACACAGCCCAGCTTGGAGCGAACGACCTACACCGAACTGAGATACCTACAGCGT
GAGCTATGAGAAAGCGCCACGCTTCCCGAAGGGAGAAAGGCGGACAGGTATCCGGTAAGCGGCAGGGTCG
GAACAGGAGAGCGCACGAGGGAGCTTCCAGGGGGAAACGCCTGGTATCTTTATAGTCCTGTCGGGTTTCG
CCACCTCTGACTTGAGCGTCGATTTTTGTGATGCTCGTCAGGGGGGCGGAGCCTATGGAAAAACGCCAGC
AACGCGGCCTTTTTACGGTTCCTGGCCTTTTGCTGGCCTTTTGCTCACATGTTCTTTCCTGCGTTATCCC
CTGATTCTGTGGATAACCGTATTACCGCCTTTGAGTGAGCTGATACCGCTCGCCGCAGCCGAACGACCGA
GCGCAGCGAGTCAGTGAGCGAGGAAGCGGAAGAGCGCCTGATGCGGTATTTTCTCCTTACGCATCTGTGC
GGTATTTCACACCGCATATGGTGCACTCTCAGTACAATCTGCTCTGATGCCGCATAGTTAAGCCAGTATA
CACTCCGCTATCGCTACGTGACTGGGTCATGGCTGCGCCCCGACACCCGCCAACACCCGCTGACGCGCCC
TGACGGGCTTGTCTGCTCCCGGCATCCGCTTACAGACAAGCTGTGACCGTCTCCGGGAGCTGCATGTGTC
AGAGGTTTTCACCGTCATCACCGAAACGCGCGAGGCAGGGTGCCTTGATGTGGGCGCCGGCGGTCGAGTG
GCGACGGCGCGGCTTGTCCGCGCCCTGGTAGATTGCCTGGCCGTAGGCCAGCCATTTTTGAGCGGCCAGC
GGCCGCGATAGGCCGACGCGAAGCGGCGGGGCGTAGGGAGCGCAGCGACCGAAGGGTAGGCGCTTTTTGC
AGCTCTTCGGCTGTGCGCTGGCCAGACAGTTATGCACAGGCCAGGCGGGTTTTAAGAGTTTTAATAAGTT
TTAAAGAGTTTTAGGCGGAAAAATCGCCTTTTTTCTCTTTTATATCAGTCACTTACATGTGTGACCGGTT
CCCAATGTACGGCTTTGGGTTCCCAATGTACGGGTTCCGGTTCCCAATGTACGGCTTTGGGTTCCCAATG
TACGTGCTATCCACAGGAAAGAGACCTTTTCGACCTTTTTCCCCTGCTAGGGCAATTTGCCCTAGCATCT
GCTCCGTACATTAGGAACCGGCGGATGCTTCGCCCTCGATCAGGTTGCGGTAGCGCATGACTAGGATCGG
GCCAGCCTGCCCCGCCTCCTCCTTCAAATCGTACTCCGGCAGGTCATTTGACCCGATCAGCTTGCGCACG
GTGAAACAGAACTTCTTGAACTCTCCGGCGCTGCCACTGCGTTCGTAGATCGTCTTGAACAACCATCTGG
CTTCTGCCTTGCCTGCGGCGCGGCGTGCCAGGCGGTAGAGAAAACGGCCGATGCCGGGATCGATCAAAAA
GTAATCGGGGTGAACCGTCAGCACGTCCGGGTTCTTGCCTTCTGTGATCTCGCGGTACATCCAATCAGCT
AGCTCGATCTCGATGTACTCCGGCCGCCCGGTTTCGCTCTTTACGATCTTGTAGCGGCTAATCAAGGCTT
CACCCTCGGATACCGTCACCAGGCGGCCGTTCTTGGCCTTCTTCGTACGCTGCATGGCAACGTGCGTGGT
GTTTAACCGAATGCAGGTTTCTACCAGGTCGTCTTTCTGCTTTCCGCCATCGGCTCGCCGGCAGAACTTG
AGTACGTCCGCAACGTGTGGACGGAACACGCGGCCGGGCTTGTCTCCCTTCCCTTCCCGGTATCGGTTCA
TGGATTCGGTTAGATGGGAAACCGCCATCAGTACCAGGTCGTAATCCCACACACTGGCCATGCCGGCCGG
CCCTGCGGAAACCTCTACGTGCCCGTCTGGAAGCTCGTAGCGGATCACCTCGCCAGCTCGTCGGTCACGC
TTCGACAGACGGAAAACGGCCACGTCCATGATGCTGCGACTATCGCGGGTGCCCACGTCATAGAGCATCG
GAACGAAAAAATCTGGTTGCTCGTCGCCCTTGGGCGGCTTCCTAATCGACGGCGCACCGGCTGCCGGCGG

TTGCCGGGATTCTTTGCGGATTCGATCAGCGGCCGCTTGCCACGATTCACCGGGGCGTGCTTCTGCCTCG
ATGCGTTGCCGCTGGGCGGCCTGCGCGGCCTTCAACTTCTCCACCAGGTCATCACCCAGCGCCGCGCCGA
TTTGTACCGGGCCGGATGGTTTGCGACCGTCACGCCGATTCCTCGGGCTTGGGGGTTCCAGTGCCATTGC
AGGGCCGGCAGACAACCCAGCCGCTTACGCCTGGCCAACCGCCCGTTCCTCCACACATGGGGCATTCCAC
GGCGTCGGTGCCTGGTTGTTCTTGATTTTCCATGCCGCCTCCTTTAGCCGCTAAAATTCATCTACTCATT
TATTCATTTGCTCATTTACTCTGGTAGCTGCGCGATGTATTCAGATAGCAGCTCGGTAATGGTCTTGCCT
TGGCGTACCGCGTACATCTTCAGCTTGGTGTGATCCTCCGCCGGCAACTGAAAGTTGACCCGCTTCATGG
CTGGCGTGTCTGCCAGGCTGGCCAACGTTGCAGCCTTGCTGCTGCGTGCGCTCGGACGGCCGGCACTTAG
CGTGTTTGTGCTTTTGCTCATTTTCTCTTTACCTCATTAACTCAAATGAGTTTTGATTTAATTTCAGCGG
CCAGCGCCTGGACCTCGCGGGCAGCGTCGCCCTCGGGTTCTGATTCAAGAACGGTTGTGCCGGCGGCGGC
AGTGCCTGGGTAGCTCACGCGCTGCGTGATACGGGACTCAAGAATGGGCAGCTCGTACCCGGCCAGCGCC
TCGGCAACCTCACCGCCGATGCGCGTGCCTTTGATCGCCCGCGACACGACAAAGGCCGCTTGTAGCCTTC
CATCCGTGACCTCAATGCGCTGCTTAACCAGCTCCACCAGGTCGGCGGTGGCCCATATGTCGTAAGGGCT
TGGCTGCACCGGAATCAGCACGAAGTCGGCTGCCTTGATCGCGGACACAGCCAAGTCCGCCGCCTGGGGC
GCTCCGTCGATCACTACGAAGTCGCGCCGGCCGATGGCCTTCACGTCGCGGTCAATCGTCGGGCGGTCGA
TGCCGACAACGGTTAGCGGTTGATCTTCCCGCACGGCCGCCCAATCGCGGGCACTGCCCTGGGGATCGGA
ATCGACTAACAGAACATCGGCCCCGGCGAGTTGCAGGGCGCGGGCTAGATGGGTTGCGATGGTCGTCTTG
CCTGACCCGCCTTTCTGGTTAAGTACAGCGATAACCTTCATGCGTTCCCCTTGCGTATTTGTTTATTTAC
TCATCGCATCATATACGCAGCGACCGCATGACGCAAGCTGTTTTACTCAAATACACATCACCTTTTTAGA
CGGCGGCGCTCGGTTTCTTCAGCGGCCAAGCTGGCCGGCCAGGCCGCCAGCTTGGCATCAGACAAACCGG
CCAGGATTTCATGCAGCCGCACGGTTGAGACGTGCGCGGGCGGCTCGAACACGTACCCGGCCGCGATCAT
CTCCGCCTCGATCTCTTCGGTAATGAAAAACGGTTCGTCCTGGCCGTCCTGGTGCGGTTTCATGCTTGTT
CCTCTTGGCGTTCATTCTCGGCGGCCGCCAGGGCGTCGGCCTCGGTCAATGCGTCCTCACGGAAGGCACC
GCGCCGCCTGGCCTCGGTGGGCGTCACTTCCTCGCTGCGCTCAAGTGCGCGGTACAGGGTCGAGCGATGC
ACGCCAAGCAGTGCAGCCGCCTCTTTCACGGTGCGGCCTTCCTGGTCGATCAGCTCGCGGGCGTGCGCGA
TCTGTGCCGGGGTGAGGGTAGGGCGGGGGCCAAACTTCACGCCTCGGGCCTTGGCGGCCTCGCGCCCGCT
CCGGGTGCGGTCGATGATTAGGGAACGCTCGAACTCGGCAATGCCGGCGAACACGGTCAACACCATGCGG
CCGGCCGGCGTGGTGGTGTCGGCCCACGGCTCTGCCAGGCTACGCAGGCCCGCGCCGGCCTCCTGGATGC
GCTCGGCAATGTCCAGTAGGTCGCGGGTGCTGCGGGCCAGGCGGTCTAGCCTGGTCACTGTCACAACGTC
GCCAGGGCGTAGGTGGTCAAGCATCCTGGCCAGCTCCGGGCGGTCGCGCCTGGTGCCGGTGATCTTCTCG
GAAAACAGCTTGGTGCAGCCGGCCGCGTGCAGTTCGGCCCGTTGGTTGGTCAAGTCCTGGTCGTCGGTGC
TGACGCGGGCATAGCCCAGCAGGCCAGCGGCGGCGCTCTTGTTCATGGCGTAATGTCTCCGGTTCTAGTC
GCAAGTATTCTACTTTATGCGACTAAAACACGCGACAAGAAAACGCCAGGAAAAGGGCAGGGCGGCAGCC
TGTCGCGTAACTTAGGACTTGTGCGACATGTCGTTTTCAGAAGACGGCTGCACTGAACGTCAGAAGCCGA
CTGCACTATAGCAGCGGAGGGGTTGGATCAAAGTACTTTGATCCCGAGGGGAACCCTGTGGTTGGCATGC
ACATACAAATGGACGAACGGATAAACCTTTTCACGCCCTTTTAAATATCCGTTATTCTAATAAACGCTCT
TTTCTCTTAGGTTTACCCGCCAATATATCCTGTCAAACACTGATAGTTTAAACTGAAGGCGGGAAACGAC
AATCTGATCCAAGCTCAAGCTAAGCTTGAGCTCTGCATGCCTGCAGGTCCCCAGATTAGCCTTTTCAATT
TCAGAAAGAATGCTAACCCACAGATGGTTAGAGAGGCTTACGCAGCAGGTCTCATCAAGACGATCTACCC
GAGCAATAATCTCCAGGAAATCAAATACCTTCCCAAGAAGGTTAAAGATGCAGTCAAAAGATTCAGGACT
AACTGCATCAAGAACACAGAGAAAGATATATTTCTCAAGATCAGAAGTACTATTCCAGTATGGACGATTC
AAGGCTTGCTTCACAAACCAAGGCAAGTAATAGAGATTGGAGTCTCTAAAAAGGTAGTTCCCACTGAATC
AAAGGCCATGGAGTCAAAGATTCAAATAGAGGACCTAACAGAACTCGCCGTAAAGACTGGCGAACAGTTC
ATACAGAGTCTCTTACGACTCAATGACAAGAAGAAAATCTTCGTCAACATGGTGGAGCACGACACACTTG

TCTACTCCAAAAATATCAAAGATACAGTCTCAGAAGACCAAAGGGCAATTGAGACTTTTCAACAAAGGGT
AATATCCGGAAACCTCCTCGGATTCCATTGCCCAGCTATCTGTCACTTTATTGTGAAGATAGTGGAAAAG
GAAGGTGGCTCCTACAAATGCCATCATTGCGATAAAGGAAAGGCCATCGTTGAAGATGCCTCTGCCGACA
GTGGTCCCAAAGATGGACCCCCACCCACGAGGAGCATCGTGGAAAAAGAAGACGTTCCAACCACGTCTTC
AAAGCAAGTGGATTGATGTGATATCTCCACTGACGTAAGGGATGACGCACAATCCCACTATCCTTCGCAA
GACCCTTCCTCTATATAAGGAAGTTCATTTCATTTGGAGAGAACACGGGGGACTCTAGAGGATCCAAGGA
GATATAAGCCGCCACCATGGATTACAAGGACGATGATGACAAGGACTACAAGGACGATGACGATAAGGAC
TATAAGGATGACGATGACAAGGATTACGCCGAGGATGGATGATACTAGCGGACAAACTTTCGTTTCTGTG
AACCTTTCTGACGAAAGCAACACGGCAAGCACTAAAGTTGAAAACGTAAAATCGGAAGCGGATCGCTTGG
AGTTTTTACGTAAAATGAATCCCTTTATTGTTGACGCTCTGGTGCGGAAAACCAATTATCAGGGTGCTCG
CTTTCGCGCAAGAATAATAGGAGTGTGCGTGGATTGTGGTAGAAAACACGACAAGGCGCTCAAGACTGAA
CGTAAGTGTAAGGTCAACAACACGCAATCTGCGAACGAGGTGGCGCATATGTTGATGCACGATCCCGTTA
AGTATTTGAACAAAAGAAAGGCTAGAGCCTTTTCTAACGCAGAGATGTTTGCGATTGAACTGGTTTCGTA
CACCAAGGAAAGGCAATTGGCGGTCGATTTAGCCGCTGAAAGGGAGAAGACGAGACTGGCTCGTAGACAC
CCAATACGTTCTCCGGAAGAAACTCCGGAACATTATAAATTCGGTATGACTGCTAAGGCAATGTTACCGG
ACATCAACGCCGTAGACGTTGGTGATAACGAGGAAACTTCGTCGGAGTACCCAGTGAGTCTGAGTGTTTC
TGGCGGAGTTCTCCGTGAACACCACTTCATCTGACCTCTTCCCTAATGATATCCCGCGGCCATGCTAGAG
TCCGCAAAAATCACCAGTCTCTCTCTACAAATCTATCTCTCTCTATTTTTCTCCAGAATAATGTGTGAGT
AGTTCCCAGATAAGGGAATTAGGGTTCTTATAGGGTTTCGCTCATGTGTTGAGCATATAAGAAACCCTTA
GTATGTATTTGTATTTGTAAAATACTTCTATCAATAAAATTTCTAATTCCTAAAACCAAAATCCAGTGAC
CTGCAGGCATGCGACGTCG

Sequence of pH7LIC3.1.1-p23M5 (35S- p23M5)

GGGTACCCTCGAATTATCATACATGAGAATTAAGGGAGTCACGTTATGACCCCGCCGATGACGCGGGACA
AGCCGTTTTACGTTTGGAACTGACAGAACCGCAACGTTGAAGGAGCCACTGAGCCGCGGGTTTCTGGAGT
TTAATGAGCTAAGCACATACGTCAGAAACCATTATTGCGCGTTCAAAAGTCGCCTAAGGTCACTATCAGC
TAGCAAATATTTCTTGTCAAAAATGCTCCACTGACGTTCCATAAATTCCCCTCGGTATCCAATTAGAGTC
TCATATTCACTCTCAACTCGAATCCCCCCTATGAAAAAGCCTGAACTCACCGCGACGTCTGTCGAGAAGT
TTCTGATCGAAAAGTTCGACAGCGTCTCCGACCTGATGCAGCTCTCGGAGGGCGAAGAATCTCGTGCTTT
CAGCTTCGATGTAGGAGGGCGTGGATATGTCCTGCGGGTAAATAGCTGCGCCGATGGTTTCTACAAAGAT
CGTTATGTTTATCGGCACTTTGCATCGGCCGCGCTCCCGATTCCGGAAGTGCTTGACATTGGGGAGTTCA
GCGAGAGCCTGACCTATTGCATCTCCCGCCGTGCACAGGGTGTCACGTTGCAAGACCTGCCTGAAACCGA
ACTGCCCGCTGTTCTTCAGCCGGTCGCGGAGGCTATGGATGCTATCGCTGCGGCCGATCTTAGCCAGACG
AGCGGGTTCGGCCCATTCGGACCGCAAGGAATCGGTCAATACACTACATGGCGTGATTTCATATGCGCGA
TTGCTGATCCCCATGTGTATCACTGGCAAACTGTGATGGACGACACCGTCAGTGCGTCCGTCGCGCAGGC
TCTCGATGAGCTGATGCTTTGGGCCGAGGACTGCCCCGAAGTCCGGCACCTCGTGCACGCGGATTCGGCTCC
AACAATGTCCTGACGGACAATGGCCGCATAACAGCGGTCATTGACTGGAGCGAGGCGATGTTCGGGGATT
CCCAATACGAGGTCGCCAACATCTTCTTCTGGAGGCCGTGGTTGGCTTGTATGGAGCAGCAGACGCGCTA
CTTCGAGCGGAGGCATCCGGAGCTTGCAGGATCGCCAAGGCTCCGGGCGTATATGCTCCGCATTGGTCTT
GACCAACTCTATCAGAGCTTGGTTGACGGCAATTTCGATGATGCAGCTTGGGCGCAGGGTCGATGCGACG
CAATCGTCCGATCCGGAGCCGGGACTGTCGGGCGTACACAAATCGCCCGCAGAAGCGCGGCCGTCTGGAC
CGATGGCTGTGTAGAAGTACTCGCCGATAGTGGAAACCGACGCCCCAGCACTCGTCCGAGGGCAAAGGAA
TAGAGTAGATGCCGACCGAACAAGAGCTGATTTCGAGAACGCCTCAGCCAGCAACTCGCGCGAGCCTAGC
AAGGCAAATGCGAGAGAACGGCCTTACGCTTGGTGGCACAGTTCTCGTCCACAGTTCGCTAAGCTCGCTC

GGCTGGGTCGCGGGAGGGCCGGTCGCAGTGATTCAGGAATTAATTCCCTAGAGTCAAGCAGATCGTTCAA
ACATTTGGCAATAAAGTTTCTTAAGATTGAATCCTGTTGCCGGTCTTGCGATGATTATCATATAATTTCT
GTTGAATTACGTTAAGCATGTAATAATTAACATGTAATGCATGACGTTATTTATGAGATGGGTTTTTATG
ATTAGAGTCCCGCAATTATACATTTAATACGCGATAGAAAACAAAATATAGCGCGCAAACTAGGATAAAT
TATCGCGCGCGGTGTCATCTATGTTACTAGATCGACCGGCATGCAAGCTGATAATTCAATTCGGCGTTAA
TTCAGTACATTAAAAACGTCCGCAATGTGTTATTAAGTTGTCTAAGCGTCAATTTGTTTACACCACAATA
TATCCTGCCACCAGCCAGCCAACAGCTCCCCGACCGGCAGCTCGGCACAAAATCACCACTCGATACAGGC
AGCCCATCAGTCCGGGACGGCGTCAGCGGGAGAGCCGTTGTAAGGCGGCAGACTTTGCTCATGTTACCGA
TGCTATTCGGAAGAACGGCAACTAAGCTGCCGGGTTTGAAACACGGATGATCTCGCGGAGGGTAGCATGT
TGATTGTAACGATGACAGAGCGTTGCTGCCTGTGATCAATTCGGGCACGAACCCAGTGGACATAAGCCTC
GTTCGGTTCGTAAGCTGTAATGCAAGTAGCGTAACTGCCGTCACGCAACTGGTCCAGAACCTTGACCGAA
CGCAGCGGTGGTAACGGCGCAGTGGCGGTTTTCATGGCTTCTTGTTATGACATGTTTTTTTGGGGTACAG
TCTATGCCTCGGGCATCCAAGCAGCAAGCGCGTTACGCCGTGGGTCGATGTTTGATGTTATGGAGCAGCA
ACGATGTTACGCAGCAGGGCAGTCGCCCTAAAACAAAGTTAAACATCATGGGGGAAGCGGTGATCGCCGA
AGTATCGACTCAACTATCAGAGGTAGTTGGCGTCATCGAGCGCCATCTCGAACCGACGTTGCTGGCCGTA
CATTTGTACGGCTCCGCAGTGGATGGCGGCCTGAAGCCACACAGTGATATTGATTTGCTGGTTACGGTGA
CCGTAAGGCTTGATGAAACAACGCGGCGAGCTTTGATCAACGACCTTTTGGAAACTTCGGCTTCCCCTGG
AGAGAGCGAGATTCTCCGCGCTGTAGAAGTCACCATTGTTGTGCACGACGACATCATTCCGTGGCGTTAT
CCAGCTAAGCGCGAACTGCAATTTGGAGAATGGCAGCGCAATGACATTCTTGCAGGTATCTTCGAGCCAG
CCACGATCGACATTGATCTGGCTATCTTGCTGACAAAAGCAAGAGAACATAGCGTTGCCTTGGTAGGTCC
AGCGGCGGAGGAACTCTTTGATCCGGTTCCTGAACAGGATCTATTTGAGGCGCTAAATGAAACCTTAACG
CTATGGAACTCGCCGCCCGACTGGGCTGGCGATGAGCGAAATGTAGTGCTTACGTTGTCCCGCATTTGGT
ACAGCGCAGTAACCGGCAAAATCGCGCCGAAGGATGTCGCTGCCGACTGGGCAATGGAGCGCCTGCCGGC
CCAGTATCAGCCCGTCATACTTGAAGCTAGACAGGCTTATCTTGGACAAGAAGAAGATCGCTTGGCCTCG
CGCGCAGATCAGTTGGAAGAATTTGTCCACTACGTGAAAGGCGAGATCACCAAGGTAGTCGGCAAATAAT
GTCTAGCTAGAAATTCGTTCAAGCCGACGCCGCTTCGCCGGCGTTAACTCAAGCGATTAGATGCACTAAG
CACATAATTGCTCACAGCCAAACTATCAGGTCAAGTCTGCTTTTATTATTTTTAAGCGTGCATAATAAGC
CCTACACAAATTGGGAGATATATCATGCATGACCAAAATCCCTTAACGTGAGTTTTCGTTCCACTGAGCG
TCAGACCCCGTAGAAAAGATCAAAGGATCTTCTTGAGATCCTTTTTTTCTGCGCGTAATCTGCTGCTTGC
AAACAAAAAAACCACCGCTACCAGCGGTGGTTTGTTTGCCGGATCAAGAGCTACCAACTCTTTTTCCGAA
GGTAACTGGCTTCAGCAGAGCGCAGATACCAAATACTGTCCTTCTAGTGTAGCCGTAGTTAGGCCACCAC
TTCAAGAACTCTGTAGCACCGCCTACATACCTCGCTCTGCTAATCCTGTTACCAGTGGCTGCTGCCAGTG
GCGATAAGTCGTGTCTTACCGGGTTGGACTCAAGACGATAGTTACCGGATAAGGCGCAGCGGTCGGGCTG
AACGGGGGGTTCGTGCACACAGCCCAGCTTGGAGCGAACGACCTACACCGAACTGAGATACCTACAGCGT
GAGCTATGAGAAAGCGCCACGCTTCCCGAAGGGAGAAAGGCGGACAGGTATCCGGTAAGCGGCAGGGTCG
GAACAGGAGAGCGCACGAGGGAGCTTCCAGGGGGAAACGCCTGGTATCTTTATAGTCCTGTCGGGTTTCG
CCACCTCTGACTTGAGCGTCGATTTTTGTGATGCTCGTCAGGGGGGCGGAGCCTATGGAAAAACGCCAGC
AACGCGGCCTTTTTACGGTTCCTGGCCTTTTGCTGGCCTTTTGCTCACATGTTCTTTCCTGCGTTATCCC
CTGATTCTGTGGATAACCGTATTACCGCCTTTGAGTGAGCTGATACCGCTCGCCGCAGCCGAACGACCGA
GCGCAGCGAGTCAGTGAGCGAGGAAGCGGAAGAGCGCCTGATGCGGTATTTTCTCCTTACGCATCTGTGC
GGTATTTCACACCGCATATGGTGCACTCTCAGTACAATCTGCTCTGATGCCGCATAGTTAAGCCAGTATA
CACTCCGCTATCGCTACGTGACTGGGTCATGGCTGCGCCCCGACACCCGCCAACACCCGCTGACGCGCCC
TGACGGGCTTGTCTGCTCCCGGCATCCGCTTACAGACAAGCTGTGACCGTCTCCGGGAGCTGCATGTGTC
AGAGGTTTTCACCGTCATCACCGAAACGCGCGAGGCAGGGTGCCTTGATGTGGGCGCCGGCGGTCGAGTG

GCGACGGCGCGGCTTGTCCGCGCCCTGGTAGATTGCCTGGCCGTAGGCCAGCCATTTTTGAGCGGCCAGC
GGCCGCGATAGGCCGACGCGAAGCGGCGGGGCGTAGGGAGCGCAGCGACCGAAGGGTAGGCGCTTTTTGC
AGCTCTTCGGCTGTGCGCTGGCCAGACAGTTATGCACAGGCCAGGCGGGTTTTAAGAGTTTTAATAAGTT
TTAAAGAGTTTTAGGCGGAAAAATCGCCTTTTTTCTCTTTTATATCAGTCACTTACATGTGTGACCGGTT
CCCAATGTACGGCTTTGGGTTCCCAATGTACGGGTTCCGGTTCCCAATGTACGGCTTTGGGTTCCCAATG
TACGTGCTATCCACAGGAAAGAGACCTTTTCGACCTTTTTCCCCTGCTAGGGCAATTTGCCCTAGCATCT
GCTCCGTACATTAGGAACCGGCGGATGCTTCGCCCTCGATCAGGTTGCGGTAGCGCATGACTAGGATCGG
GCCAGCCTGCCCCGCCTCCTCCTTCAAATCGTACTCCGGCAGGTCATTTGACCCGATCAGCTTGCGCACG
GTGAAACAGAACTTCTTGAACTCTCCGGCGCTGCCACTGCGTTCGTAGATCGTCTTGAACAACCATCTGG
CTTCTGCCTTGCCTGCGGCGCGGCGTGCCAGGCGGTAGAGAAAACGGCCGATGCCGGGATCGATCAAAAA
GTAATCGGGGTGAACCGTCAGCACGTCCGGGTTCTTGCCTTCTGTGATCTCGCGGTACATCCAATCAGCT
AGCTCGATCTCGATGTACTCCGGCCGCCCGGTTTCGCTCTTTACGATCTTGTAGCGGCTAATCAAGGCTT
CACCCTCGGATACCGTCACCAGGCGGCCGTTCTTGGCCTTCTTCGTACGCTGCATGGCAACGTGCGTGGT
GTTTAACCGAATGCAGGTTTCTACCAGGTCGTCTTTCTGCTTTCCGCCATCGGCTCGCCGGCAGAACTTG
AGTACGTCCGCAACGTGTGGACGGAACACGCGGCCGGGCTTGTCTCCCTTCCCTTCCCGGTATCGGTTCA
TGGATTCGGTTAGATGGGAAACCGCCATCAGTACCAGGTCGTAATCCCACACACTGGCCATGCCGGCCGG
CCCTGCGGAAACCTCTACGTGCCCGTCTGGAAGCTCGTAGCGGATCACCTCGCCAGCTCGTCGGTCACGC
TTCGACAGACGGAAAACGGCCACGTCCATGATGCTGCGACTATCGCGGGTGCCCACGTCATAGAGCATCG
GAACGAAAAAATCTGGTTGCTCGTCGCCCTTGGGCGGCTTCCTAATCGACGGCGCACCGGCTGCCGGCGG
TTGCCGGGATTCTTTGCGGATTCGATCAGCGGCCGCTTGCCACGATTCACCGGGGCGTGCTTCTGCCTCG
ATGCGTTGCCGCTGGGCGGCCTGCGCGGCCTTCAACTTCTCCACCAGGTCATCACCCAGCGCCGCGCCGA
TTTGTACCGGGCCGGATGGTTTGCGACCGTCACGCCGATTCCTCGGGCTTGGGGGTTCCAGTGCCATTGC
AGGGCCGGCAGACAACCCAGCCGCTTACGCCTGGCCAACCGCCCGTTCCTCCACACATGGGGCATTCCAC
GGCGTCGGTGCCTGGTTGTTCTTGATTTTCCATGCCGCCTCCTTTAGCCGCTAAAATTCATCTACTCATT
TATTCATTTGCTCATTTACTCTGGTAGCTGCGCGATGTATTCAGATAGCAGCTCGGTAATGGTCTTGCCT
TGGCGTACCGCGTACATCTTCAGCTTGGTGTGATCCTCCGCCGGCAACTGAAAGTTGACCCGCTTCATGG
CTGGCGTGTCTGCCAGGCTGGCCAACGTTGCAGCCTTGCTGCTGCGTGCGCTCGGACGGCCGGCACTTAG
CGTGTTTGTGCTTTTGCTCATTTTCTCTTTACCTCATTAACTCAAATGAGTTTTGATTTAATTTCAGCGG
CCAGCGCCTGGACCTCGCGGGCAGCGTCGCCCTCGGGTTCTGATTCAAGAACGGTTGTGCCGGCGGCGGC
AGTGCCTGGGTAGCTCACGCGCTGCGTGATACGGGACTCAAGAATGGGCAGCTCGTACCCGGCCAGCGCC
TCGGCAACCTCACCGCCGATGCGCGTGCCTTTGATCGCCCGCGACACGACAAAGGCCGCTTGTAGCCTTC
CATCCGTGACCTCAATGCGCTGCTTAACCAGCTCCACCAGGTCGGCGGTGGCCCATATGTCGTAAGGGCT
TGGCTGCACCGGAATCAGCACGAAGTCGGCTGCCTTGATCGCGGACACAGCCAAGTCCGCCGCCTGGGGC
GCTCCGTCGATCACTACGAAGTCGCGCCGGCCGATGGCCTTCACGTCGCGGTCAATCGTCGGGCGGTCGA
TGCCGACAACGGTTAGCGGTTGATCTTCCCGCACGGCCGCCCAATCGCGGGCACTGCCCTGGGGATCGGA
ATCGACTAACAGAACATCGGCCCCGGCGAGTTGCAGGGCGCGGGCTAGATGGGTTGCGATGGTCGTCTTG
CCTGACCCGCCTTTCTGGTTAAGTACAGCGATAACCTTCATGCGTTCCCCTTGCGTATTTGTTTATTTAC
TCATCGCATCATATACGCAGCGACCGCATGACGCAAGCTGTTTTACTCAAATACACATCACCTTTTTAGA
CGGCGGCGCTCGGTTTCTTCAGCGGCCAAGCTGGCCGGCCAGGCCGCCAGCTTGGCATCAGACAAACCGG
CCAGGATTTCATGCAGCCGCACGGTTGAGACGTGCGCGGGCGGCTCGAACACGTACCCGGCCGCGATCAT
CTCCGCCTCGATCTCTTCGGTAATGAAAAACGGTTCGTCCTGGCCGTCCTGGTGCGGTTTCATGCTTGTT
CCTCTTGGCGTTCATTCTCGGCGGCCGCCAGGGCGTCGGCCTCGGTCAATGCGTCCTCACGGAAGGCACC
GCGCCGCCTGGCCTCGGTGGGCGTCACTTCCTCGCTGCGCTCAAGTGCGCGGTACAGGGTCGAGCGATGC
ACGCCAAGCAGTGCAGCCGCCTCTTTCACGGTGCGGCCTTCCTGGTCGATCAGCTCGCGGGCGTGCGCGA

TCTGTGCCGGGGTGAGGGTAGGGCGGGGGCCAAACTTCACGCCTCGGGCCTTGGCGGCCTCGCGCCCGCT
CCGGGTGCGGTCGATGATTAGGGAACGCTCGAACTCGGCAATGCCGGCGAACACGGTCAACACCATGCGG
CCGGCCGGCGTGGTGGTGTCGGCCCACGGCTCTGCCAGGCTACGCAGGCCCGCGCCGGCCTCCTGGATGC
GCTCGGCAATGTCCAGTAGGTCGCGGGTGCTGCGGGCCAGGCGGTCTAGCCTGGTCACTGTCACAACGTC
GCCAGGGCGTAGGTGGTCAAGCATCCTGGCCAGCTCCGGGCGGTCGCGCCTGGTGCCGGTGATCTTCTCG
GAAAACAGCTTGGTGCAGCCGGCCGCGTGCAGTTCGGCCCGTTGGTTGGTCAAGTCCTGGTCGTCGGTGC
TGACGCGGGCATAGCCCAGCAGGCCAGCGGCGGCGCTCTTGTTCATGGCGTAATGTCTCCGGTTCTAGTC
GCAAGTATTCTACTTTATGCGACTAAAACACGCGACAAGAAAACGCCAGGAAAAGGGCAGGGCGGCAGCC
TGTCGCGTAACTTAGGACTTGTGCGACATGTCGTTTTCAGAAGACGGCTGCACTGAACGTCAGAAGCCGA
CTGCACTATAGCAGCGGAGGGGTTGGATCAAAGTACTTTGATCCCGAGGGGAACCCTGTGGTTGGCATGC
ACATACAAATGGACGAACGGATAAACCTTTTCACGCCCTTTTAAATATCCGTTATTCTAATAAACGCTCT
TTTCTCTTAGGTTTACCCGCCAATATATCCTGTCAAACACTGATAGTTTAAACTGAAGGCGGGAAACGAC
AATCTGATCCAAGCTCAAGCTAAGCTTGAGCTCTGCATGCCTGCAGGTCCCCAGATTAGCCTTTTCAATT
TCAGAAAGAATGCTAACCCACAGATGGTTAGAGAGGCTTACGCAGCAGGTCTCATCAAGACGATCTACCC
GAGCAATAATCTCCAGGAAATCAAATACCTTCCCAAGAAGGTTAAAGATGCAGTCAAAAGATTCAGGACT
AACTGCATCAAGAACACAGAGAAAGATATATTTCTCAAGATCAGAAGTACTATTCCAGTATGGACGATTC
AAGGCTTGCTTCACAAACCAAGGCAAGTAATAGAGATTGGAGTCTCTAAAAAGGTAGTTCCCACTGAATC
AAAGGCCATGGAGTCAAAGATTCAAATAGAGGACCTAACAGAACTCGCCGTAAAGACTGGCGAACAGTTC
ATACAGAGTCTCTTACGACTCAATGACAAGAAGAAAATCTTCGTCAACATGGTGGAGCACGACACACTTG
TCTACTCCAAAAATATCAAAGATACAGTCTCAGAAGACCAAAGGGCAATTGAGACTTTTCAACAAAGGGT
AATATCCGGAAACCTCCTCGGATTCCATTGCCCAGCTATCTGTCACTTTATTGTGAAGATAGTGGAAAAG
GAAGGTGGCTCCTACAAATGCCATCATTGCGATAAAGGAAAGGCCATCGTTGAAGATGCCTCTGCCGACA
GTGGTCCCAAAGATGGACCCCCACCCACGAGGAGCATCGTGGAAAAAGAAGACGTTCCAACCACGTCTTC
AAAGCAAGTGGATTGATGTGATATCTCCACTGACGTAAGGGATGACGCACAATCCCACTATCCTTCGCAA
GACCCTTCCTCTATATAAGGAAGTTCATTTCATTTGGAGAGAACACGGGGGACTCTAGAGGATCCAAGGA
GATATAAGCCGCCACCATGGATTACAAGGACGATGATGACAAGGACTACAAGGACGATGACGATAAGGAC
TATAAGGATGACGATGACAAGGATTACGCCGAGGATGGATGATACTAGCGGACAAACTTTCGTTTCTGTG
AACCTTTCTGACGAAAGCAACACGGCAAGCACTAAAGTTGAAAACGTAAAATCGGAAGCGGATCGCTTGG
AGTTTTTACGTAAAATGAATCCCTTTATTGTTGACGCTCTGGTGCGGAAAACCAATTATCAGGGTGCTCG
CTTTCGCGCAAGAATAATAGGAGTGTGCGTGGATTGTGGTAGAAAACACGACAAGGCGCTCAAGACTGAA
CGTAAGTGTAAGGTCAACAACACGCAATCTCAGAACGCGGCGGCGCATATGTTGATGCACGATCCCGTTA
AGTATTTGAACAAAAGAAAGGCTAGAGCCTTTTCTAACGCAGAGATGTTTGCGATTGAACTGGTTTCGTA
CACCAAGGAAAGGCAATTGGCGGTCGATTTAGCCGCTGAAAGGGAGAAGACGAGACTGGCTCGTAGACAC
CCAATACGTTCTCCGGAAGAAACTCCGGAACATTATAAATTCGGTATGACTGCTAAGGCAATGTTACCGG
ACATCAACGCCGTAGACGTTGGTGATAACGAGGAAACTTCGTCGGAGTACCCAGTGAGTCTGAGTGTTTC
TGGCGGAGTTCTCCGTGAACACCACTTCATCTGACCTAATGATATCCCGCGGCCATGCTAGAGTCCGCAA
AAATCACCAGTCTCTCTCTACAAATCTATCTCTCTCTATTTTTCTCCAGAATAATGTGTGAGTAGTTCCC
AGATAAGGGAATTAGGGTTCTTATAGGGTTTCGCTCATGTGTTGAGCATATAAGAAACCCTTAGTATGTA
TTTGTATTTGTAAAATACTTCTATCAATAAAATTTCTAATTCCTAAAACCAAAATCCAGTGACCTGCAGG
CATGCGACGTCG

Sequence of pH7LIC3.1.1-p23M6 (35S- p23M6)

GGGTACCCTCGAATTATCATACATGAGAATTAAGGGAGTCACGTTATGACCCCGCCGATGACGCGGGACA
AGCCGTTTTACGTTTGGAACTGACAGAACCGCAACGTTGAAGGAGCCACTGAGCCGCGGGTTTCTGGAGT

TTAATGAGCTAAGCACATACGTCAGAAACCATTATTGCGCGTTCAAAAGTCGCCTAAGGTCACTATCAGC
TAGCAAATATTTCTTGTCAAAAATGCTCCACTGACGTTCCATAAATTCCCCTCGGTATCCAATTAGAGTC
TCATATTCACTCTCAACTCGAATCCCCCCTATGAAAAAGCCTGAACTCACCGCGACGTCTGTCGAGAAGT
TTCTGATCGAAAAGTTCGACAGCGTCTCCGACCTGATGCAGCTCTCGGAGGGCGAAGAATCTCGTGCTTT
CAGCTTCGATGTAGGAGGGCGTGGATATGTCCTGCGGGTAAATAGCTGCGCCGATGGTTTCTACAAAGAT
CGTTATGTTTATCGGCACTTTGCATCGGCCGCGCTCCCGATTCCGGAAGTGCTTGACATTGGGGAGTTCA
GCGAGAGCCTGACCTATTGCATCTCCCGCCGTGCACAGGGTGTCACGTTGCAAGACCTGCCTGAAACCGA
ACTGCCCGCTGTTCTTCAGCCGGTCGCGGAGGCTATGGATGCTATCGCTGCGGCCGATCTTAGCCAGACG
AGCGGGTTCGGCCCATTCGGACCGCAAGGAATCGGTCAATACACTACATGGCGTGATTTCATATGCGCGA
TTGCTGATCCCCATGTGTATCACTGGCAAACTGTGATGGACGACACCGTCAGTGCGTCCGTCGCGCAGGC
TCTCGATGAGCTGATGCTTTGGGCGAGGACTGCCCCGAAGTCCGGCACTCGTGCACGCGGATTCGGCTCC
AACAATGTCCTGACGGACAATGGCCGCATAACAGCGGTCATTGACTGGAGCGAGGCGATGTTCGGGGATT
CCCAATACGAGGTCGCCAACATCTTCTTCTGGAGGCCGTGGTTGGCTTGTATGGAGCAGCAGACGCGCTA
CTTCGAGCGGAGGCATCCGGAGCTTGCAGGATCGCCAAGGCTCCGGGCGTATATGCTCCGCATTGGTCTT
GACCAACTCTATCAGAGCTTGGTTGACGGCAATTTCGATGATGCAGCTTGGGCGCAGGGTCGATGCGACG
CAATCGTCCGATCCGGAGCCGGGACTGTCGGGCGTACACAAATCGCCCGCAGAAGCGCGGCCGTCTGGAC
CGATGGCTGTGTAGAAGTACTCGCCGATAGTGGAAACCGACGCCCCAGCACTCGTCCGAGGGCAAAGGAA
TAGAGTAGATGCCGACCGAACAAGAGCTGATTTCGAGAACGCCTCAGCCAGCAACTCGCGCGAGCCTAGC
AAGGCAAATGCGAGAGAACGGCCTTACGCTTGGTGGCACAGTTCTCGTCCACAGTTCGCTAAGCTCGCTC
GGCTGGGTCGCGGGAGGGCCGGTCGCAGTGATTCAGGAATTAATTCCCTAGAGTCAAGCAGATCGTTCAA
ACATTTGGCAATAAAGTTTCTTAAGATTGAATCCTGTTGCCGGTCTTGCGATGATTATCATATAATTTCT
GTTGAATTACGTTAAGCATGTAATAATTAACATGTAATGCATGACGTTATTTATGAGATGGGTTTTTATG
ATTAGAGTCCCGCAATTATACATTTAATACGCGATAGAAAACAAAATATAGCGCGCAAACTAGGATAAAT
TATCGCGCGCGGTGTCATCTATGTTACTAGATCGACCGGCATGCAAGCTGATAATTCAATTCGGCGTTAA
TTCAGTACATTAAAAACGTCCGCAATGTGTTATTAAGTTGTCTAAGCGTCAATTTGTTTACACCACAATA
TATCCTGCCACCAGCCAGCCAACAGCTCCCCGACCGGCAGCTCGGCACAAAATCACCACTCGATACAGGC
AGCCCATCAGTCCGGGACGGCGTCAGCGGGAGAGCCGTTGTAAGGCGGCAGACTTTGCTCATGTTACCGA
TGCTATTCGGAAGAACGGCAACTAAGCTGCCGGGTTTGAAACACGGATGATCTCGCGGAGGGTAGCATGT
TGATTGTAACGATGACAGAGCGTTGCTGCCTGTGATCAATTCGGGCACGAACCCAGTGGACATAAGCCTC
GTTCGGTTCGTAAGCTGTAATGCAAGTAGCGTAACTGCCGTCACGCAACTGGTCCAGAACCTTGACCGAA
CGCAGCGGTGGTAACGGCGCAGTGGCGGTTTTCATGGCTTCTTGTTATGACATGTTTTTTTGGGGTACAG
TCTATGCCTCGGGCATCCAAGCAGCAAGCGCGTTACGCCGTGGGTCGATGTTTGATGTTATGGAGCAGCA
ACGATGTTACGCAGCAGGGCAGTCGCCCTAAAACAAAGTTAAACATCATGGGGGAAGCGGTGATCGCCGA
AGTATCGACTCAACTATCAGAGGTAGTTGGCGTCATCGAGCGCCATCTCGAACCGACGTTGCTGGCCGTA
CATTTGTACGGCTCCGCAGTGGATGGCGGCCTGAAGCCACACAGTGATATTGATTTGCTGGTTACGGTGA
CCGTAAGGCTTGATGAAACAACGCGGCGAGCTTTGATCAACGACCTTTTGGAAACTTCGGCTTCCCCTGG
AGAGAGCGAGATTCTCCGCGCTGTAGAAGTCACCATTGTTGTGCACGACGACATCATTCCGTGGCGTTAT
CCAGCTAAGCGCGAACTGCAATTTGGAGAATGGCAGCGCAATGACATTCTTGCAGGTATCTTCGAGCCAG
CCACGATCGACATTGATCTGGCTATCTTGCTGACAAAAGCAAGAGAACATAGCGTTGCCTTGGTAGGTCC
AGCGGCGGAGGAACTCTTTGATCCGGTTCCTGAACAGGATCTATTTGAGGCGCTAAATGAAACCTTAACG
CTATGGAACTCGCCGCCCGACTGGGCTGGCGATGAGCGAAATGTAGTGCTTACGTTGTCCCGCATTTGGT
ACAGCGCAGTAACCGGCAAAATCGCGCCGAAGGATGTCGCTGCCGACTGGGCAATGGAGCGCCTGCCGGC
CCAGTATCAGCCCGTCATACTTGAAGCTAGACAGGCTTATCTTGGACAAGAAGAAGATCGCTTGGCCTCG
CGCGCAGATCAGTTGGAAGAATTTGTCCACTACGTGAAAGGCGAGATCACCAAGGTAGTCGGCAAATAAT

GTCTAGCTAGAAATTCGTTCAAGCCGACGCCGCTTCGCCGGCGTTAACTCAAGCGATTAGATGCACTAAG
CACATAATTGCTCACAGCCAAACTATCAGGTCAAGTCTGCTTTTATTATTTTTAAGCGTGCATAATAAGC
CCTACACAAATTGGGAGATATATCATGCATGACCAAAATCCCTTAACGTGAGTTTTCGTTCCACTGAGCG
TCAGACCCCGTAGAAAAGATCAAAGGATCTTCTTGAGATCCTTTTTTTCTGCGCGTAATCTGCTGCTTGC
AAACAAAAAAACCACCGCTACCAGCGGTGGTTTGTTTGCCGGATCAAGAGCTACCAACTCTTTTTCCGAA
GGTAACTGGCTTCAGCAGAGCGCAGATACCAAATACTGTCCTTCTAGTGTAGCCGTAGTTAGGCCACCAC
TTCAAGAACTCTGTAGCACCGCCTACATACCTCGCTCTGCTAATCCTGTTACCAGTGGCTGCTGCCAGTG
GCGATAAGTCGTGTCTTACCGGGTTGGACTCAAGACGATAGTTACCGGATAAGGCGCAGCGGTCGGGCTG
AACGGGGGGTTCGTGCACACAGCCCAGCTTGGAGCGAACGACCTACACCGAACTGAGATACCTACAGCGT
GAGCTATGAGAAAGCGCCACGCTTCCCGAAGGGAGAAAGGCGGACAGGTATCCGGTAAGCGGCAGGGTCG
GAACAGGAGAGCGCACGAGGGAGCTTCCAGGGGGAAACGCCTGGTATCTTTATAGTCCTGTCGGGTTTCG
CCACCTCTGACTTGAGCGTCGATTTTTGTGATGCTCGTCAGGGGGGCGGAGCCTATGGAAAAACGCCAGC
AACGCGGCCTTTTTACGGTTCCTGGCCTTTTGCTGGCCTTTTGCTCACATGTTCTTTCCTGCGTTATCCC
CTGATTCTGTGGATAACCGTATTACCGCCTTTGAGTGAGCTGATACCGCTCGCCGCAGCCGAACGACCGA
GCGCAGCGAGTCAGTGAGCGAGGAAGCGGAAGAGCGCCTGATGCGGTATTTTCTCCTTACGCATCTGTGC
GGTATTTCACACCGCATATGGTGCACTCTCAGTACAATCTGCTCTGATGCCGCATAGTTAAGCCAGTATA
CACTCCGCTATCGCTACGTGACTGGGTCATGGCTGCGCCCCGACACCCGCCAACACCCGCTGACGCGCCC
TGACGGGCTTGTCTGCTCCCGGCATCCGCTTACAGACAAGCTGTGACCGTCTCCGGGAGCTGCATGTGTC
AGAGGTTTTCACCGTCATCACCGAAACGCGCGAGGCAGGGTGCCTTGATGTGGGCGCCGGCGGTCGAGTG
GCGACGGCGCGGCTTGTCCGCGCCCTGGTAGATTGCCTGGCCGTAGGCCAGCCATTTTTGAGCGGCCAGC
GGCCGCGATAGGCCGACGCGAAGCGGCGGGGCGTAGGGAGCGCAGCGACCGAAGGGTAGGCGCTTTTTGC
AGCTCTTCGGCTGTGCGCTGGCCAGACAGTTATGCACAGGCCAGGCGGGTTTTAAGAGTTTTAATAAGTT
TTAAAGAGTTTTAGGCGGAAAAATCGCCTTTTTTCTCTTTTATATCAGTCACTTACATGTGTGACCGGTT
CCCAATGTACGGCTTTGGGTTCCCAATGTACGGGTTCCGGTTCCCAATGTACGGCTTTGGGTTCCCAATG
TACGTGCTATCCACAGGAAAGAGACCTTTTCGACCTTTTTCCCCTGCTAGGGCAATTTGCCCTAGCATCT
GCTCCGTACATTAGGAACCGGCGGATGCTTCGCCCTCGATCAGGTTGCGGTAGCGCATGACTAGGATCGG
GCCAGCCTGCCCCGCCTCCTCCTTCAAATCGTACTCCGGCAGGTCATTTGACCCGATCAGCTTGCGCACG
GTGAAACAGAACTTCTTGAACTCTCCGGCGCTGCCACTGCGTTCGTAGATCGTCTTGAACAACCATCTGG
CTTCTGCCTTGCCTGCGGCGCGGCGTGCCAGGCGGTAGAGAAAACGGCCGATGCCGGGATCGATCAAAAA
GTAATCGGGGTGAACCGTCAGCACGTCCGGGTTCTTGCCTTCTGTGATCTCGCGGTACATCCAATCAGCT
AGCTCGATCTCGATGTACTCCGGCCGCCCGGTTTCGCTCTTTACGATCTTGTAGCGGCTAATCAAGGCTT
CACCCTCGGATACCGTCACCAGGCGGCCGTTCTTGGCCTTCTTCGTACGCTGCATGGCAACGTGCGTGGT
GTTTAACCGAATGCAGGTTTCTACCAGGTCGTCTTTCTGCTTTCCGCCATCGGCTCGCCGGCAGAACTTG
AGTACGTCCGCAACGTGTGGACGGAACACGCGGCCGGGCTTGTCTCCCTTCCCTTCCCGGTATCGGTTCA
TGGATTCGGTTAGATGGGAAACCGCCATCAGTACCAGGTCGTAATCCCACACACTGGCCATGCCGGCCGG
CCCTGCGGAAACCTCTACGTGCCCGTCTGGAAGCTCGTAGCGGATCACCTCGCCAGCTCGTCGGTCACGC
TTCGACAGACGGAAAACGGCCACGTCCATGATGCTGCGACTATCGCGGGTGCCCACGTCATAGAGCATCG
GAACGAAAAAATCTGGTTGCTCGTCGCCCTTGGGCGGCTTCCTAATCGACGGCGCACCGGCTGCCGGCGG
TTGCCGGGATTCTTTGCGGATTCGATCAGCGGCCGCTTGCCACGATTCACCGGGGCGTGCTTCTGCCTCG
ATGCGTTGCCGCTGGGCGGCCTGCGCGGCCTTCAACTTCTCCACCAGGTCATCACCCAGCGCCGCGCCGA
TTTGTACCGGGCCGGATGGTTTGCGACCGTCACGCCGATTCCTCGGGCTTGGGGGTTCCAGTGCCATTGC
AGGGCCGGCAGACAACCCAGCCGCTTACGCCTGGCCAACCGCCCGTTCCTCCACACATGGGGCATTCCAC
GGCGTCGGTGCCTGGTTGTTCTTGATTTTCCATGCCGCCTCCTTTAGCCGCTAAAATTCATCTACTCATT
TATTCATTTGCTCATTTACTCTGGTAGCTGCGCGATGTATTCAGATAGCAGCTCGGTAATGGTCTTGCCT

TGGCGTACCGCGTACATCTTCAGCTTGGTGTGATCCTCCGCCGGCAACTGAAAGTTGACCCGCTTCATGG
CTGGCGTGTCTGCCAGGCTGGCCAACGTTGCAGCCTTGCTGCTGCGTGCGCTCGGACGGCCGGCACTTAG
CGTGTTTGTGCTTTTGCTCATTTTCTCTTTACCTCATTAACTCAAATGAGTTTTGATTTAATTTCAGCGG
CCAGCGCCTGGACCTCGCGGGCAGCGTCGCCCTCGGGTTCTGATTCAAGAACGGTTGTGCCGGCGGCGGC
AGTGCCTGGGTAGCTCACGCGCTGCGTGATACGGGACTCAAGAATGGGCAGCTCGTACCCGGCCAGCGCC
TCGGCAACCTCACCGCCGATGCGCGTGCCTTTGATCGCCCGCGACACGACAAAGGCCGCTTGTAGCCTTC
CATCCGTGACCTCAATGCGCTGCTTAACCAGCTCCACCAGGTCGGCGGTGGCCCATATGTCGTAAGGGCT
TGGCTGCACCGGAATCAGCACGAAGTCGGCTGCCTTGATCGCGGACACAGCCAAGTCCGCCGCCTGGGGC
GCTCCGTCGATCACTACGAAGTCGCGCCGGCCGATGGCCTTCACGTCGCGGTCAATCGTCGGGCGGTCGA
TGCCGACAACGGTTAGCGGTTGATCTTCCCGCACGGCCGCCCAATCGCGGGCACTGCCCTGGGGATCGGA
ATCGACTAACAGAACATCGGCCCCGGCGAGTTGCAGGGCGCGGGCTAGATGGGTTGCGATGGTCGTCTTG
CCTGACCCGCCTTTCTGGTTAAGTACAGCGATAACCTTCATGCGTTCCCCTTGCGTATTTGTTTATTTAC
TCATCGCATCATATACGCAGCGACCGCATGACGCAAGCTGTTTTACTCAAATACACATCACCTTTTTAGA
CGGCGGCGCTCGGTTTCTTCAGCGGCCAAGCTGGCCGGCCAGGCCGCCAGCTTGGCATCAGACAAACCGG
CCAGGATTTCATGCAGCCGCACGGTTGAGACGTGCGCGGGCGGCTCGAACACGTACCCGGCCGCGATCAT
CTCCGCCTCGATCTCTTCGGTAATGAAAAACGGTTCGTCCTGGCCGTCCTGGTGCGGTTTCATGCTTGTT
CCTCTTGGCGTTCATTCTCGGCGGCCGCCAGGGCGTCGGCCTCGGTCAATGCGTCCTCACGGAAGGCACC
GCGCCGCCTGGCCTCGGTGGGCGTCACTTCCTCGCTGCGCTCAAGTGCGCGGTACAGGGTCGAGCGATGC
ACGCCAAGCAGTGCAGCCGCCTCTTTCACGGTGCGGCCTTCCTGGTCGATCAGCTCGCGGGCGTGCGCGA
TCTGTGCCGGGGTGAGGGTAGGGCGGGGGCCAAACTTCACGCCTCGGGCCTTGGCGGCCTCGCGCCCGCT
CCGGGTGCGGTCGATGATTAGGGAACGCTCGAACTCGGCAATGCCGGCGAACACGGTCAACACCATGCGG
CCGGCCGGCGTGGTGGTGTCGGCCCACGGCTCTGCCAGGCTACGCAGGCCCGCGCCGGCCTCCTGGATGC
GCTCGGCAATGTCCAGTAGGTCGCGGGTGCTGCGGGCCAGGCGGTCTAGCCTGGTCACTGTCACAACGTC
GCCAGGGCGTAGGTGGTCAAGCATCCTGGCCAGCTCCGGGCGGTCGCGCCTGGTGCCGGTGATCTTCTCG
GAAAACAGCTTGGTGCAGCCGGCCGCGTGCAGTTCGGCCCGTTGGTTGGTCAAGTCCTGGTCGTCGGTGC
TGACGCGGGCATAGCCCAGCAGGCCAGCGGCGGCGCTCTTGTTCATGGCGTAATGTCTCCGGTTCTAGTC
GCAAGTATTCTACTTTATGCGACTAAAACACGCGACAAGAAAACGCCAGGAAAAGGGCAGGGCGGCAGCC
TGTCGCGTAACTTAGGACTTGTGCGACATGTCGTTTTCAGAAGACGGCTGCACTGAACGTCAGAAGCCGA
CTGCACTATAGCAGCGGAGGGGTTGGATCAAAGTACTTTGATCCCGAGGGGAACCCTGTGGTTGGCATGC
ACATACAAATGGACGAACGGATAAACCTTTTCACGCCCTTTTAAATATCCGTTATTCTAATAAACGCTCT
TTTCTCTTAGGTTTACCCGCCAATATATCCTGTCAAACACTGATAGTTTAAACTGAAGGCGGGAAACGAC
AATCTGATCCAAGCTCAAGCTAAGCTTGAGCTCTGCATGCCTGCAGGTCCCCAGATTAGCCTTTTCAATT
TCAGAAAGAATGCTAACCCACAGATGGTTAGAGAGGCTTACGCAGCAGGTCTCATCAAGACGATCTACCC
GAGCAATAATCTCCAGGAAATCAAATACCTTCCCAAGAAGGTTAAAGATGCAGTCAAAAGATTCAGGACT
AACTGCATCAAGAACACAGAGAAAGATATATTTCTCAAGATCAGAAGTACTATTCCAGTATGGACGATTC
AAGGCTTGCTTCACAAACCAAGGCAAGTAATAGAGATTGGAGTCTCTAAAAAGGTAGTTCCCACTGAATC
AAAGGCCATGGAGTCAAAGATTCAAATAGAGGACCTAACAGAACTCGCCGTAAAGACTGGCGAACAGTTC
ATACAGAGTCTCTTACGACTCAATGACAAGAAGAAAATCTTCGTCAACATGGTGGAGCACGACACACTTG
TCTACTCCAAAAATATCAAAGATACAGTCTCAGAAGACCAAAGGGCAATTGAGACTTTTCAACAAAGGGT
AATATCCGGAAACCTCCTCGGATTCCATTGCCCAGCTATCTGTCACTTTATTGTGAAGATAGTGGAAAAG
GAAGGTGGCTCCTACAAATGCCATCATTGCGATAAAGGAAAGGCCATCGTTGAAGATGCCTCTGCCGACA
GTGGTCCCAAAGATGGACCCCCACCCACGAGGAGCATCGTGGAAAAAGAAGACGTTCCAACCACGTCTTC
AAAGCAAGTGGATTGATGTGATATCTCCACTGACGTAAGGGATGACGCACAATCCCACTATCCTTCGCAA
GACCCTTCCTCTATATAAGGAAGTTCATTTCATTTGGAGAGAACACGGGGGACTCTAGAGGATCCAAGGA

GATATAAGCCGCCACCATGGATTACAAGGACGATGATGACAAGGACTACAAGGACGATGACGATAAGGAC
TATAAGGATGACGATGACAAGGATTACGCCGAGGATGGATGATACTAGCGGACAAACTTTCGTTTCTGTG
AACCTTTCTGACGAAAGCAACACGGCAAGCACTAAAGTTGAAAACGTAAAATCGGAAGCGGATCGCTTGG
AGTTTTTACGTAAAATGAATCCCTTTATTGTTGACGCTCTGGTGCGGAAAACCAATTATCAGGGTGCTCG
CTTTCGCGCAAGAATAATAGGAGTGTGCGTGGATTGTGGTAGAAAACACGACAAGGCGCTCAAGACTGAA
CGTAAGTGTAAGGTCAACAACACGCAATCTCAGAACGAGGTGGCGCATGCGGCGATGCACGATCCCGTTA
AGTATTTGAACAAAAGAAAGGCTAGAGCCTTTTCTAACGCAGAGATGTTTGCGATTGAACTGGTTTCGTA
CACCAAGGAAAGGCAATTGGCGGTCGATTTAGCCGCTGAAAGGGAGAAGACGAGACTGGCTCGTAGACAC
CCAATACGTTCTCCGGAAGAAACTCCGGAACATTATAAATTCGGTATGACTGCTAAGGCAATGTTACCGG
ACATCAACGCCGTAGACGTTGGTGATAACGAGGAAACTTCGTCGGAGTACCCAGTGAGTCTGAGTGTTTC
TGGCGGAGTTCTCCGTGAACACCACTTCATCTGACCTAATGATATCCCGCGGCCATGCTAGAGTCCGCAA
AAATCACCAGTCTCTCTCTACAAATCTATCTCTCTCTATTTTTCTCCAGAATAATGTGTGAGTAGTTCCC
AGATAAGGGAATTAGGGTTCTTATAGGGTTTCGCTCATGTGTTGAGCATATAAGAAACCCTTAGTATGTA
TTTGTATTTGTAAAATACTTCTATCAATAAAATTTCTAATTCCTAAAACCAAAATCCAGTGACCTGCAGG
CATGCGACGTCG

Sequence of pH7LIC3.1.1-p23M7 (35S- p23M7)

GGGTACCCTCGAATTATCATACATGAGAATTAAGGGAGTCACGTTATGACCCCGCCGATGACGCGGGACA
AGCCGTTTTACGTTTGGAACTGACAGAACCGCAACGTTGAAGGAGCCACTGAGCCGCGGGTTTCTGGAGT
TTAATGAGCTAAGCACATACGTCAGAAACCATTATTGCGCGTTCAAAAGTCGCCTAAGGTCACTATCAGC
TAGCAAATATTTCTTGTCAAAAATGCTCCACTGACGTTCCATAAATTCCCCTCGGTATCCAATTAGAGTC
TCATATTCACTCTCAACTCGAATCCCCCCTATGAAAAAGCCTGAACTCACCGCGACGTCTGTCGAGAAGT
TTCTGATCGAAAAGTTCGACAGCGTCTCCGACCTGATGCAGCTCTCGGAGGGCGAAGAATCTCGTGCTTT
CAGCTTCGATGTAGGAGGGCGTGGATATGTCCTGCGGGTAAATAGCTGCGCCGATGGTTTCTACAAAGAT
CGTTATGTTTATCGGCACTTTGCATCGGCCGCGCTCCCGATTCCGGAAGTGCTTGACATTGGGGAGTTCA
GCGAGAGCCTGACCTATTGCATCTCCCGCCGTGCACAGGGTGTCACGTTGCAAGACCTGCCTGAAACCGA
ACTGCCCGCTGTTCTTCAGCCGGTCGCGGAGGCTATGGATGCTATCGCTGCGGCCGATCTTAGCCAGACG
AGCGGGTTCGGCCCATTCGGACCGCAAGGAATCGGTCAATACACTACATGGCGTGATTTCATATGCGCGA
TTGCTGATCCCCATGTGTATCACTGGCAAACTGTGATGGACGACACCGTCAGTGCGTCCGTCGCGCAGGC
TCTCGATGAGCTGATGCTTTGGGCGAGGACTGCCCCGAAGTCCGGCACTCGTGCACGCGGATTCGGCTCC
AACAATGTCCTGACGGACAATGGCCGCATAACAGCGGTCATTGACTGGAGCGAGGCGATGTTCGGGGATT
CCCAATACGAGGTCGCCAACATCTTCTTCTGGAGGCCGTGGTTGGCTTGTATGGAGCAGCAGACGCGCTA
CTTCGAGCGGAGGCATCCGGAGCTTGCAGGATCGCCAAGGCTCCGGGCGTATATGCTCCGCATTGGTCTT
GACCAACTCTATCAGAGCTTGGTTGACGGCAATTTCGATGATGCAGCTTGGGCGCAGGGTCGATGCGACG
CAATCGTCCGATCCGGAGCCGGGACTGTCGGGCGTACACAAATCGCCCGCAGAAGCGCGGCCGTCTGGAC
CGATGGCTGTGTAGAAGTACTCGCCGATAGTGGAAACCGACGCCCCAGCACTCGTCCGAGGGCAAAGGAA
TAGAGTAGATGCCGACCGAACAAGAGCTGATTTCGAGAACGCCTCAGCCAGCAACTCGCGCGAGCCTAGC
AAGGCAAATGCGAGAGAACGGCCTTACGCTTGGTGGCACAGTTCTCGTCCACAGTTCGCTAAGCTCGCTC
GGCTGGGTCGCGGGAGGGCCGGTCGCAGTGATTCAGGAATTAATTCCCTAGAGTCAAGCAGATCGTTCAA
ACATTTGGCAATAAAGTTTCTTAAGATTGAATCCTGTTGCCGGTCTTGCGATGATTATCATATAATTTCT
GTTGAATTACGTTAAGCATGTAATAATTAACATGTAATGCATGACGTTATTTATGAGATGGGTTTTTATG
ATTAGAGTCCCGCAATTATACATTTAATACGCGATAGAAAACAAAATATAGCGCGCAAACTAGGATAAAT
TATCGCGCGCGGTGTCATCTATGTTACTAGATCGACCGGCATGCAAGCTGATAATTCAATTCGGCGTTAA
TTCAGTACATTAAAAACGTCCGCAATGTGTTATTAAGTTGTCTAAGCGTCAATTTGTTTACACCACAATA

TATCCTGCCACCAGCCAGCCAACAGCTCCCCGACCGGCAGCTCGGCACAAAATCACCACTCGATACAGGC
AGCCCATCAGTCCGGGACGGCGTCAGCGGGAGAGCCGTTGTAAGGCGGCAGACTTTGCTCATGTTACCGA
TGCTATTCGGAAGAACGGCAACTAAGCTGCCGGGTTTGAAACACGGATGATCTCGCGGAGGGTAGCATGT
TGATTGTAACGATGACAGAGCGTTGCTGCCTGTGATCAATTCGGGCACGAACCCAGTGGACATAAGCCTC
GTTCGGTTCGTAAGCTGTAATGCAAGTAGCGTAACTGCCGTCACGCAACTGGTCCAGAACCTTGACCGAA
CGCAGCGGTGGTAACGGCGCAGTGGCGGTTTTCATGGCTTCTTGTTATGACATGTTTTTTTGGGGTACAG
TCTATGCCTCGGGCATCCAAGCAGCAAGCGCGTTACGCCGTGGGTCGATGTTTGATGTTATGGAGCAGCA
ACGATGTTACGCAGCAGGGCAGTCGCCCTAAAACAAAGTTAAACATCATGGGGGAAGCGGTGATCGCCGA
AGTATCGACTCAACTATCAGAGGTAGTTGGCGTCATCGAGCGCCATCTCGAACCGACGTTGCTGGCCGTA
CATTTGTACGGCTCCGCAGTGGATGGCGGCCTGAAGCCACACAGTGATATTGATTTGCTGGTTACGGTGA
CCGTAAGGCTTGATGAAACAACGCGGCGAGCTTTGATCAACGACCTTTTGGAAACTTCGGCTTCCCCTGG
AGAGAGCGAGATTCTCCGCGCTGTAGAAGTCACCATTGTTGTGCACGACGACATCATTCCGTGGCGTTAT
CCAGCTAAGCGCGAACTGCAATTTGGAGAATGGCAGCGCAATGACATTCTTGCAGGTATCTTCGAGCCAG
CCACGATCGACATTGATCTGGCTATCTTGCTGACAAAAGCAAGAGAACATAGCGTTGCCTTGGTAGGTCC
AGCGGCGGAGGAACTCTTTGATCCGGTTCCTGAACAGGATCTATTTGAGGCGCTAAATGAAACCTTAACG
CTATGGAACTCGCCGCCCGACTGGGCTGGCGATGAGCGAAATGTAGTGCTTACGTTGTCCCGCATTTGGT
ACAGCGCAGTAACCGGCAAAATCGCGCCGAAGGATGTCGCTGCCGACTGGGCAATGGAGCGCCTGCCGGC
CCAGTATCAGCCCGTCATACTTGAAGCTAGACAGGCTTATCTTGGACAAGAAGAAGATCGCTTGGCCTCG
CGCGCAGATCAGTTGGAAGAATTTGTCCACTACGTGAAAGGCGAGATCACCAAGGTAGTCGGCAAATAAT
GTCTAGCTAGAAATTCGTTCAAGCCGACGCCGCTTCGCCGGCGTTAACTCAAGCGATTAGATGCACTAAG
CACATAATTGCTCACAGCCAAACTATCAGGTCAAGTCTGCTTTTATTATTTTTAAGCGTGCATAATAAGC
CCTACACAAATTGGGAGATATATCATGCATGACCAAAATCCCTTAACGTGAGTTTTCGTTCCACTGAGCG
TCAGACCCCGTAGAAAAGATCAAAGGATCTTCTTGAGATCCTTTTTTTCTGCGCGTAATCTGCTGCTTGC
AAACAAAAAAACCACCGCTACCAGCGGTGGTTTGTTTGCCGGATCAAGAGCTACCAACTCTTTTTCCGAA
GGTAACTGGCTTCAGCAGAGCGCAGATACCAAATACTGTCCTTCTAGTGTAGCCGTAGTTAGGCCACCAC
TTCAAGAACTCTGTAGCACCGCCTACATACCTCGCTCTGCTAATCCTGTTACCAGTGGCTGCTGCCAGTG
GCGATAAGTCGTGTCTTACCGGGTTGGACTCAAGACGATAGTTACCGGATAAGGCGCAGCGGTCGGGCTG
AACGGGGGGTTCGTGCACACAGCCCAGCTTGGAGCGAACGACCTACACCGAACTGAGATACCTACAGCGT
GAGCTATGAGAAAGCGCCACGCTTCCCGAAGGGAGAAAGGCGGACAGGTATCCGGTAAGCGGCAGGGTCG
GAACAGGAGAGCGCACGAGGGAGCTTCCAGGGGGAAACGCCTGGTATCTTTATAGTCCTGTCGGGTTTCG
CCACCTCTGACTTGAGCGTCGATTTTTGTGATGCTCGTCAGGGGGGCGGAGCCTATGGAAAAACGCCAGC
AACGCGGCCTTTTTACGGTTCCTGGCCTTTTGCTGGCCTTTTGCTCACATGTTCTTTCCTGCGTTATCCC
CTGATTCTGTGGATAACCGTATTACCGCCTTTGAGTGAGCTGATACCGCTCGCCGCAGCCGAACGACCGA
GCGCAGCGAGTCAGTGAGCGAGGAAGCGGAAGAGCGCCTGATGCGGTATTTTCTCCTTACGCATCTGTGC
GGTATTTCACACCGCATATGGTGCACTCTCAGTACAATCTGCTCTGATGCCGCATAGTTAAGCCAGTATA
CACTCCGCTATCGCTACGTGACTGGGTCATGGCTGCGCCCCGACACCCGCCAACACCCGCTGACGCGCCC
TGACGGGCTTGTCTGCTCCCGGCATCCGCTTACAGACAAGCTGTGACCGTCTCCGGGAGCTGCATGTGTC
AGAGGTTTTCACCGTCATCACCGAAACGCGCGAGGCAGGGTGCCTTGATGTGGGCGCCGGCGGTCGAGTG
GCGACGGCGCGGCTTGTCCGCGCCCTGGTAGATTGCCTGGCCGTAGGCCAGCCATTTTTGAGCGGCCAGC
GGCCGCGATAGGCCGACGCGAAGCGGCGGGGCGTAGGGAGCGCAGCGACCGAAGGGTAGGCGCTTTTTGC
AGCTCTTCGGCTGTGCGCTGGCCAGACAGTTATGCACAGGCCAGGCGGGTTTTAAGAGTTTTAATAAGTT
TTAAAGAGTTTTAGGCGGAAAAATCGCCTTTTTTCTCTTTTATATCAGTCACTTACATGTGTGACCGGTT
CCCAATGTACGGCTTTGGGTTCCCAATGTACGGGTTCCGGTTCCCAATGTACGGCTTTGGGTTCCCAATG
TACGTGCTATCCACAGGAAAGAGACCTTTTCGACCTTTTTCCCCTGCTAGGGCAATTTGCCCTAGCATCT

GCTCCGTACATTAGGAACCGGCGGATGCTTCGCCCTCGATCAGGTTGCGGTAGCGCATGACTAGGATCGG
GCCAGCCTGCCCCGCCTCCTCCTTCAAATCGTACTCCGGCAGGTCATTTGACCCGATCAGCTTGCGCACG
GTGAAACAGAACTTCTTGAACTCTCCGGCGCTGCCACTGCGTTCGTAGATCGTCTTGAACAACCATCTGG
CTTCTGCCTTGCCTGCGGCGCGGCGTGCCAGGCGGTAGAGAAAACGGCCGATGCCGGGATCGATCAAAAA
GTAATCGGGGTGAACCGTCAGCACGTCCGGGTTCTTGCCTTCTGTGATCTCGCGGTACATCCAATCAGCT
AGCTCGATCTCGATGTACTCCGGCCGCCCGGTTTCGCTCTTTACGATCTTGTAGCGGCTAATCAAGGCTT
CACCCTCGGATACCGTCACCAGGCGGCCGTTCTTGGCCTTCTTCGTACGCTGCATGGCAACGTGCGTGGT
GTTTAACCGAATGCAGGTTTCTACCAGGTCGTCTTTCTGCTTTCCGCCATCGGCTCGCCGGCAGAACTTG
AGTACGTCCGCAACGTGTGGACGGAACACGCGGCCGGGCTTGTCTCCCTTCCCTTCCCGGTATCGGTTCA
TGGATTCGGTTAGATGGGAAACCGCCATCAGTACCAGGTCGTAATCCCACACACTGGCCATGCCGGCCGG
CCCTGCGGAAACCTCTACGTGCCCGTCTGGAAGCTCGTAGCGGATCACCTCGCCAGCTCGTCGGTCACGC
TTCGACAGACGGAAAACGGCCACGTCCATGATGCTGCGACTATCGCGGGTGCCCACGTCATAGAGCATCG
GAACGAAAAAATCTGGTTGCTCGTCGCCCTTGGGCGGCTTCCTAATCGACGGCGCACCGGCTGCCGGCGG
TTGCCGGGATTCTTTGCGGATTCGATCAGCGGCCGCTTGCCACGATTCACCGGGGCGTGCTTCTGCCTCG
ATGCGTTGCCGCTGGGCGGCCTGCGCGGCCTTCAACTTCTCCACCAGGTCATCACCCAGCGCCGCGCCGA
TTTGTACCGGGCCGGATGGTTTGCGACCGTCACGCCGATTCCTCGGGCTTGGGGGTTCCAGTGCCATTGC
AGGGCCGGCAGACAACCCAGCCGCTTACGCCTGGCCAACCGCCCGTTCCTCCACACATGGGGCATTCCAC
GGCGTCGGTGCCTGGTTGTTCTTGATTTTCCATGCCGCCTCCTTTAGCCGCTAAAATTCATCTACTCATT
TATTCATTTGCTCATTTACTCTGGTAGCTGCGCGATGTATTCAGATAGCAGCTCGGTAATGGTCTTGCCT
TGGCGTACCGCGTACATCTTCAGCTTGGTGTGATCCTCCGCCGGCAACTGAAAGTTGACCCGCTTCATGG
CTGGCGTGTCTGCCAGGCTGGCCAACGTTGCAGCCTTGCTGCTGCGTGCGCTCGGACGGCCGGCACTTAG
CGTGTTTGTGCTTTTGCTCATTTTCTCTTTACCTCATTAACTCAAATGAGTTTTGATTTAATTTCAGCGG
CCAGCGCCTGGACCTCGCGGGCAGCGTCGCCCTCGGGTTCTGATTCAAGAACGGTTGTGCCGGCGGCGGC
AGTGCCTGGGTAGCTCACGCGCTGCGTGATACGGGACTCAAGAATGGGCAGCTCGTACCCGGCCAGCGCC
TCGGCAACCTCACCGCCGATGCGCGTGCCTTTGATCGCCCGCGACACGACAAAGGCCGCTTGTAGCCTTC
CATCCGTGACCTCAATGCGCTGCTTAACCAGCTCCACCAGGTCGGCGGTGGCCCATATGTCGTAAGGGCT
TGGCTGCACCGGAATCAGCACGAAGTCGGCTGCCTTGATCGCGGACACAGCCAAGTCCGCCGCCTGGGGC
GCTCCGTCGATCACTACGAAGTCGCGCCGGCCGATGGCCTTCACGTCGCGGTCAATCGTCGGGCGGTCGA
TGCCGACAACGGTTAGCGGTTGATCTTCCCGCACGGCCGCCCAATCGCGGGCACTGCCCTGGGGATCGGA
ATCGACTAACAGAACATCGGCCCCGGCGAGTTGCAGGGCGCGGGCTAGATGGGTTGCGATGGTCGTCTTG
CCTGACCCGCCTTTCTGGTTAAGTACAGCGATAACCTTCATGCGTTCCCCTTGCGTATTTGTTTATTTAC
TCATCGCATCATATACGCAGCGACCGCATGACGCAAGCTGTTTTACTCAAATACACATCACCTTTTTAGA
CGGCGGCGCTCGGTTTCTTCAGCGGCCAAGCTGGCCGGCCAGGCCGCCAGCTTGGCATCAGACAAACCGG
CCAGGATTTCATGCAGCCGCACGGTTGAGACGTGCGCGGGCGGCTCGAACACGTACCCGGCCGCGATCAT
CTCCGCCTCGATCTCTTCGGTAATGAAAAACGGTTCGTCCTGGCCGTCCTGGTGCGGTTTCATGCTTGTT
CCTCTTGGCGTTCATTCTCGGCGGCCGCCAGGGCGTCGGCCTCGGTCAATGCGTCCTCACGGAAGGCACC
GCGCCGCCTGGCCTCGGTGGGCGTCACTTCCTCGCTGCGCTCAAGTGCGCGGTACAGGGTCGAGCGATGC
ACGCCAAGCAGTGCAGCCGCCTCTTTCACGGTGCGGCCTTCCTGGTCGATCAGCTCGCGGGCGTGCGCGA
TCTGTGCCGGGGTGAGGGTAGGGCGGGGGCCAAACTTCACGCCTCGGGCCTTGGCGGCCTCGCGCCCGCT
CCGGGTGCGGTCGATGATTAGGGAACGCTCGAACTCGGCAATGCCGGCGAACACGGTCAACACCATGCGG
CCGGCCGGCGTGGTGGTGTCGGCCCACGGCTCTGCCAGGCTACGCAGGCCCGCGCCGGCCTCCTGGATGC
GCTCGGCAATGTCCAGTAGGTCGCGGGTGCTGCGGGCCAGGCGGTCTAGCCTGGTCACTGTCACAACGTC
GCCAGGGCGTAGGTGGTCAAGCATCCTGGCCAGCTCCGGGCGGTCGCGCCTGGTGCCGGTGATCTTCTCG
GAAAACAGCTTGGTGCAGCCGGCCGCGTGCAGTTCGGCCCGTTGGTTGGTCAAGTCCTGGTCGTCGGTGC

TGACGCGGGCATAGCCCAGCAGGCCAGCGGCGGCGCTCTTGTTCATGGCGTAATGTCTCCGGTTCTAGTC
GCAAGTATTCTACTTTATGCGACTAAAACACGCGACAAGAAAACGCCAGGAAAAGGGCAGGGCGGCAGCC
TGTCGCGTAACTTAGGACTTGTGCGACATGTCGTTTTCAGAAGACGGCTGCACTGAACGTCAGAAGCCGA
CTGCACTATAGCAGCGGAGGGGTTGGATCAAAGTACTTTGATCCCGAGGGGAACCCTGTGGTTGGCATGC
ACATACAAATGGACGAACGGATAAACCTTTTCACGCCCTTTTAAATATCCGTTATTCTAATAAACGCTCT
TTTCTCTTAGGTTTACCCGCCAATATATCCTGTCAAACACTGATAGTTTAAACTGAAGGCGGGAAACGAC
AATCTGATCCAAGCTCAAGCTAAGCTTGAGCTCTGCATGCCTGCAGGTCCCCAGATTAGCCTTTTCAATT
TCAGAAAGAATGCTAACCCACAGATGGTTAGAGAGGCTTACGCAGCAGGTCTCATCAAGACGATCTACCC
GAGCAATAATCTCCAGGAAATCAAATACCTTCCCAAGAAGGTTAAAGATGCAGTCAAAAGATTCAGGACT
AACTGCATCAAGAACACAGAGAAAGATATATTTCTCAAGATCAGAAGTACTATTCCAGTATGGACGATTC
AAGGCTTGCTTCACAAACCAAGGCAAGTAATAGAGATTGGAGTCTCTAAAAAGGTAGTTCCCACTGAATC
AAAGGCCATGGAGTCAAAGATTCAAATAGAGGACCTAACAGAACTCGCCGTAAAGACTGGCGAACAGTTC
ATACAGAGTCTCTTACGACTCAATGACAAGAAGAAAATCTTCGTCAACATGGTGGAGCACGACACACTTG
TCTACTCCAAAAATATCAAAGATACAGTCTCAGAAGACCAAAGGGCAATTGAGACTTTTCAACAAAGGGT
AATATCCGGAAACCTCCTCGGATTCCATTGCCCAGCTATCTGTCACTTTATTGTGAAGATAGTGGAAAAG
GAAGGTGGCTCCTACAAATGCCATCATTGCGATAAAGGAAAGGCCATCGTTGAAGATGCCTCTGCCGACA
GTGGTCCCAAAGATGGACCCCCACCCACGAGGAGCATCGTGGAAAAAGAAGACGTTCCAACCACGTCTTC
AAAGCAAGTGGATTGATGTGATATCTCCACTGACGTAAGGGATGACGCACAATCCCACTATCCTTCGCAA
GACCCTTCCTCTATATAAGGAAGTTCATTTCATTTGGAGAGAACACGGGGGACTCTAGAGGATCCAAGGA
GATATAAGCCGCCACCATGGATTACAAGGACGATGATGACAAGGACTACAAGGACGATGACGATAAGGAC
TATAAGGATGACGATGACAAGGATTACGCCGAGGATGGATGATACTAGCGGACAAACTTTCGTTTCTGTG
AACCTTTCTGACGAAAGCAACACGGCAAGCACTAAAGTTGAAAACGTAAAATCGGAAGCGGATCGCTTGG
AGTTTTTACGTAAAATGAATCCCTTTATTGTTGACGCTCTGGTGCGGAAAACCAATTATCAGGGTGCTCG
CTTTCGCGCAAGAATAATAGGAGTGTGCGTGGATTGTGGTAGAAAACACGACAAGGCGCTCAAGACTGAA
CGTAAGTGTAAGGTCAACAACACGCAATCTCAGAACGAGGTGGCGCATATGTTGATGCACGATGCGGTTA
AGTATTTGAACAAAAGAAAGGCTAGAGCCTTTTCTAACGCAGAGATGTTTGCGATTGAACTGGTTTCGTA
CACCAAGGAAAGGCAATTGGCGGTCGATTTAGCCGCTGAAAGGGAGAAGACGAGACTGGCTCGTAGACAC
CCAATACGTTCTCCGGAAGAAACTCCGGAACATTATAAATTCGGTATGACTGCTAAGGCAATGTTACCGG
ACATCAACGCCGTAGACGTTGGTGATAACGAGGAAACTTCGTCGGAGTACCCAGTGAGTCTGAGTGTTTC
TGGCGGAGTTCTCCGTGAACACCACTTCATCTGACCTAATGATATCCCGCGGCCATGCTAGAGTCCGCAA
AAATCACCAGTCTCTCTCTACAAATCTATCTCTCTCTATTTTTCTCCAGAATAATGTGTGAGTAGTTCCC
AGATAAGGGAATTAGGGTTCTTATAGGGTTTCGCTCATGTGTTGAGCATATAAGAAACCCTTAGTATGTA
TTTGTATTTGTAAAATACTTCTATCAATAAAATTTCTAATTCCTAAAACCAAAATCCAGTGACCTGCAGG
CATGCGACGTCG

Sequence of pH7LIC3.1.1-p23M8 (35S- p23M8)

GGGTACCCTCGAATTATCATACATGAGAATTAAGGGAGTCACGTTATGACCCCGCCGATGACGCGGGACA
AGCCGTTTTACGTTTGGAACTGACAGAACCGCAACGTTGAAGGAGCCACTGAGCCGCGGGTTTCTGGAGT
TTAATGAGCTAAGCACATACGTCAGAAACCATTATTGCGCGTTCAAAAGTCGCCTAAGGTCACTATCAGC
TAGCAAATATTTCTTGTCAAAAATGCTCCACTGACGTTCCATAAATTCCCCTCGGTATCCAATTAGAGTC
TCATATTCACTCTCAACTCGAATCCCCCTATGAAAAAGCCTGAACTCACCGCGACGTCTGTCGAGAAGT
TTCTGATCGAAAAGTTCGACAGCGTCTCCGACCTGATGCAGCTCTCGGAGGGCGAAGAATCTCGTGCTTT
CAGCTTCGATGTAGGAGGGCGTGGATATGTCCTGCGGGTAAATAGCTGCGCCGATGGTTTCTACAAAGAT
CGTTATGTTTATCGGCACTTTGCATCGGCCGCGCTCCCGATTCCGGAAGTGCTTGACATTGGGGAGTTCA

GCGAGAGCCTGACCTATTGCATCTCCCGCCGTGCACAGGGTGTCACGTTGCAAGACCTGCCTGAAACCGA
ACTGCCCGCTGTTCTTCAGCCGGTCGCGGAGGCTATGGATGCTATCGCTGCGGCCGATCTTAGCCAGACG
AGCGGGTTCGGCCCATTCGGACCGCAAGGAATCGGTCAATACACTACATGGCGTGATTTCATATGCGCGA
TTGCTGATCCCCATGTGTATCACTGGCAAACTGTGATGGACGACACCGTCAGTGCGTCCGTCGCGCAGGC
TCTCGATGAGCTGATGCTTTGGGCGAGGACTGCCCCGAAGTCCGGCACTCGTGCACGCGGATTCGGCTCC
AACAATGTCCTGACGGACAATGGCCGCATAACAGCGGTCATTGACTGGAGCGAGGCGATGTTCGGGGATT
CCCAATACGAGGTCGCCAACATCTTCTTCTGGAGGCCGTGGTTGGCTTGTATGGAGCAGCAGACGCGCTA
CTTCGAGCGGAGGCATCCGGAGCTTGCAGGATCGCCAAGGCTCCGGGCGTATATGCTCCGCATTGGTCTT
GACCAACTCTATCAGAGCTTGGTTGACGGCAATTTCGATGATGCAGCTTGGGCGCAGGGTCGATGCGACG
CAATCGTCCGATCCGGAGCCGGGACTGTCGGGCGTACACAAATCGCCCGCAGAAGCGCGGCCGTCTGGAC
CGATGGCTGTGTAGAAGTACTCGCCGATAGTGGAAACCGACGCCCCAGCACTCGTCCGAGGGCAAAGGAA
TAGAGTAGATGCCGACCGAACAAGAGCTGATTTCGAGAACGCCTCAGCCAGCAACTCGCGCGAGCCTAGC
AAGGCAAATGCGAGAGAACGGCCTTACGCTTGGTGGCACAGTTCTCGTCCACAGTTCGCTAAGCTCGCTC
GGCTGGGTCGCGGGAGGGCCGGTCGCAGTGATTCAGGAATTAATTCCCTAGAGTCAAGCAGATCGTTCAA
ACATTTGGCAATAAAGTTTCTTAAGATTGAATCCTGTTGCCGGTCTTGCGATGATTATCATATAATTTCT
GTTGAATTACGTTAAGCATGTAATAATTAACATGTAATGCATGACGTTATTTATGAGATGGGTTTTTATG
ATTAGAGTCCCGCAATTATACATTTAATACGCGATAGAAAACAAAATATAGCGCGCAAACTAGGATAAAT
TATCGCGCGCGGTGTCATCTATGTTACTAGATCGACCGGCATGCAAGCTGATAATTCAATTCGGCGTTAA
TTCAGTACATTAAAAACGTCCGCAATGTGTTATTAAGTTGTCTAAGCGTCAATTTGTTTACACCACAATA
TATCCTGCCACCAGCCAGCCAACAGCTCCCCGACCGGCAGCTCGGCACAAAATCACCACTCGATACAGGC
AGCCCATCAGTCCGGGACGGCGTCAGCGGGAGAGCCGTTGTAAGGCGGCAGACTTTGCTCATGTTACCGA
TGCTATTCGGAAGAACGGCAACTAAGCTGCCGGGTTTGAAACACGGATGATCTCGCGGAGGGTAGCATGT
TGATTGTAACGATGACAGAGCGTTGCTGCCTGTGATCAATTCGGGCACGAACCCAGTGGACATAAGCCTC
GTTCGGTTCGTAAGCTGTAATGCAAGTAGCGTAACTGCCGTCACGCAACTGGTCCAGAACCTTGACCGAA
CGCAGCGGTGGTAACGGCGCAGTGGCGGTTTTCATGGCTTCTTGTTATGACATGTTTTTTTGGGGTACAG
TCTATGCCTCGGGCATCCAAGCAGCAAGCGCGTTACGCCGTGGGTCGATGTTTGATGTTATGGAGCAGCA
ACGATGTTACGCAGCAGGGCAGTCGCCCTAAAACAAAGTTAAACATCATGGGGGAAGCGGTGATCGCCGA
AGTATCGACTCAACTATCAGAGGTAGTTGGCGTCATCGAGCGCCATCTCGAACCGACGTTGCTGGCCGTA
CATTTGTACGGCTCCGCAGTGGATGGCGGCCTGAAGCCACACAGTGATATTGATTTGCTGGTTACGGTGA
CCGTAAGGCTTGATGAAACAACGCGGCGAGCTTTGATCAACGACCTTTTGGAAACTTCGGCTTCCCCTGG
AGAGAGCGAGATTCTCCGCGCTGTAGAAGTCACCATTGTTGTGCACGACGACATCATTCCGTGGCGTTAT
CCAGCTAAGCGCGAACTGCAATTTGGAGAATGGCAGCGCAATGACATTCTTGCAGGTATCTTCGAGCCAG
CCACGATCGACATTGATCTGGCTATCTTGCTGACAAAAGCAAGAGAACATAGCGTTGCCTTGGTAGGTCC
AGCGGCGGAGGAACTCTTTGATCCGGTTCCTGAACAGGATCTATTTGAGGCGCTAAATGAAACCTTAACG
CTATGGAACTCGCCGCCCGACTGGGCTGGCGATGAGCGAAATGTAGTGCTTACGTTGTCCCGCATTTGGT
ACAGCGCAGTAACCGGCAAAATCGCGCCGAAGGATGTCGCTGCCGACTGGGCAATGGAGCGCCTGCCGGC
CCAGTATCAGCCCGTCATACTTGAAGCTAGACAGGCTTATCTTGGACAAGAAGAAGATCGCTTGGCCTCG
CGCGCAGATCAGTTGGAAGAATTTGTCCACTACGTGAAAGGCGAGATCACCAAGGTAGTCGGCAAATAAT
GTCTAGCTAGAAATTCGTTCAAGCCGACGCCGCTTCGCCGGCGTTAACTCAAGCGATTAGATGCACTAAG
CACATAATTGCTCACAGCCAAACTATCAGGTCAAGTCTGCTTTTATTATTTTTAAGCGTGCATAATAAGC
CCTACACAAATTGGGAGATATATCATGCATGACCAAAATCCCTTAACGTGAGTTTTCGTTCCACTGAGCG
TCAGACCCCGTAGAAAAGATCAAAGGATCTTCTTGAGATCCTTTTTTTCTGCGCGTAATCTGCTGCTTGC
AAACAAAAAAACCACCGCTACCAGCGGTGGTTTGTTTGCCGGATCAAGAGCTACCAACTCTTTTTCCGAA
GGTAACTGGCTTCAGCAGAGCGCAGATACCAAATACTGTCCTTCTAGTGTAGCCGTAGTTAGGCCACCAC

TTCAAGAACTCTGTAGCACCGCCTACATACCTCGCTCTGCTAATCCTGTTACCAGTGGCTGCTGCCAGTG
GCGATAAGTCGTGTCTTACCGGGTTGGACTCAAGACGATAGTTACCGGATAAGGCGCAGCGGTCGGGCTG
AACGGGGGGTTCGTGCACACAGCCCAGCTTGGAGCGAACGACCTACACCGAACTGAGATACCTACAGCGT
GAGCTATGAGAAAGCGCCACGCTTCCCGAAGGGAGAAAGGCGGACAGGTATCCGGTAAGCGGCAGGGTCG
GAACAGGAGAGCGCACGAGGGAGCTTCCAGGGGGAAACGCCTGGTATCTTTATAGTCCTGTCGGGTTTCG
CCACCTCTGACTTGAGCGTCGATTTTTGTGATGCTCGTCAGGGGGGCGGAGCCTATGGAAAAACGCCAGC
AACGCGGCCTTTTTACGGTTCCTGGCCTTTTGCTGGCCTTTTGCTCACATGTTCTTTCCTGCGTTATCCC
CTGATTCTGTGGATAACCGTATTACCGCCTTTGAGTGAGCTGATACCGCTCGCCGCAGCCGAACGACCGA
GCGCAGCGAGTCAGTGAGCGAGGAAGCGGAAGAGCGCCTGATGCGGTATTTTCTCCTTACGCATCTGTGC
GGTATTTCACACCGCATATGGTGCACTCTCAGTACAATCTGCTCTGATGCCGCATAGTTAAGCCAGTATA
CACTCCGCTATCGCTACGTGACTGGGTCATGGCTGCGCCCCGACACCCGCCAACACCCGCTGACGCGCCC
TGACGGGCTTGTCTGCTCCCGGCATCCGCTTACAGACAAGCTGTGACCGTCTCCGGGAGCTGCATGTGTC
AGAGGTTTTCACCGTCATCACCGAAACGCGCGAGGCAGGGTGCCTTGATGTGGGCGCCGGCGGTCGAGTG
GCGACGGCGCGGCTTGTCCGCGCCCTGGTAGATTGCCTGGCCGTAGGCCAGCCATTTTTGAGCGGCCAGC
GGCCGCGATAGGCCGACGCGAAGCGGCGGGGCGTAGGGAGCGCAGCGACCGAAGGGTAGGCGCTTTTTGC
AGCTCTTCGGCTGTGCGCTGGCCAGACAGTTATGCACAGGCCAGGCGGGTTTTAAGAGTTTTAATAAGTT
TTAAAGAGTTTTAGGCGGAAAAATCGCCTTTTTTCTCTTTTATATCAGTCACTTACATGTGTGACCGGTT
CCCAATGTACGGCTTTGGGTTCCCAATGTACGGGTTCCGGTTCCCAATGTACGGCTTTGGGTTCCCAATG
TACGTGCTATCCACAGGAAAGAGACCTTTTCGACCTTTTTCCCCTGCTAGGGCAATTTGCCCTAGCATCT
GCTCCGTACATTAGGAACCGGCGGATGCTTCGCCCTCGATCAGGTTGCGGTAGCGCATGACTAGGATCGG
GCCAGCCTGCCCCGCCTCCTCCTTCAAATCGTACTCCGGCAGGTCATTTGACCCGATCAGCTTGCGCACG
GTGAAACAGAACTTCTTGAACTCTCCGGCGCTGCCACTGCGTTCGTAGATCGTCTTGAACAACCATCTGG
CTTCTGCCTTGCCTGCGGCGCGGCGTGCCAGGCGGTAGAGAAAACGGCCGATGCCGGGATCGATCAAAAA
GTAATCGGGGTGAACCGTCAGCACGTCCGGGTTCTTGCCTTCTGTGATCTCGCGGTACATCCAATCAGCT
AGCTCGATCTCGATGTACTCCGGCCGCCCGGTTTCGCTCTTTACGATCTTGTAGCGGCTAATCAAGGCTT
CACCCTCGGATACCGTCACCAGGCGGCCGTTCTTGGCCTTCTTCGTACGCTGCATGGCAACGTGCGTGGT
GTTTAACCGAATGCAGGTTTCTACCAGGTCGTCTTTCTGCTTTCCGCCATCGGCTCGCCGGCAGAACTTG
AGTACGTCCGCAACGTGTGGACGGAACACGCGGCCGGGCTTGTCTCCCTTCCCTTCCCGGTATCGGTTCA
TGGATTCGGTTAGATGGGAAACCGCCATCAGTACCAGGTCGTAATCCCACACACTGGCCATGCCGGCCGG
CCCTGCGGAAACCTCTACGTGCCCGTCTGGAAGCTCGTAGCGGATCACCTCGCCAGCTCGTCGGTCACGC
TTCGACAGACGGAAAACGGCCACGTCCATGATGCTGCGACTATCGCGGGTGCCCACGTCATAGAGCATCG
GAACGAAAAAATCTGGTTGCTCGTCGCCCTTGGGCGGCTTCCTAATCGACGGCGCACCGGCTGCCGGCGG
TTGCCGGGATTCTTTGCGGATTCGATCAGCGGCCGCTTGCCACGATTCACCGGGGCGTGCTTCTGCCTCG
ATGCGTTGCCGCTGGGCGGCCTGCGCGGCCTTCAACTTCTCCACCAGGTCATCACCCAGCGCCGCGCCGA
TTTGTACCGGGCCGGATGGTTTGCGACCGTCACGCCGATTCCTCGGGCTTGGGGGTTCCAGTGCCATTGC
AGGGCCGGCAGACAACCCAGCCGCTTACGCCTGGCCAACCGCCCGTTCCTCCACACATGGGGCATTCCAC
GGCGTCGGTGCCTGGTTGTTCTTGATTTTCCATGCCGCCTCCTTTAGCCGCTAAAATTCATCTACTCATT
TATTCATTTGCTCATTTACTCTGGTAGCTGCGCGATGTATTCAGATAGCAGCTCGGTAATGGTCTTGCCT
TGGCGTACCGCGTACATCTTCAGCTTGGTGTGATCCTCCGCCGGCAACTGAAAGTTGACCCGCTTCATGG
CTGGCGTGTCTGCCAGGCTGGCCAACGTTGCAGCCTTGCTGCTGCGTGCGCTCGGACGGCCGGCACTTAG
CGTGTTTGTGCTTTTGCTCATTTTCTCTTTACCTCATTAACTCAAATGAGTTTTGATTTAATTTCAGCGG
CCAGCGCCTGGACCTCGCGGGCAGCGTCGCCCTCGGGTTCTGATTCAAGAACGGTTGTGCCGGCGGCGGC
AGTGCCTGGGTAGCTCACGCGCTGCGTGATACGGGACTCAAGAATGGGCAGCTCGTACCCGGCCAGCGCC
TCGGCAACCTCACCGCCGATGCGCGTGCCTTTGATCGCCCGCGACACGACAAAGGCCGCTTGTAGCCTTC

CATCCGTGACCTCAATGCGCTGCTTAACCAGCTCCACCAGGTCGGCGGTGGCCCATATGTCGTAAGGGCT
TGGCTGCACCGGAATCAGCACGAAGTCGGCTGCCTTGATCGCGGACACAGCCAAGTCCGCCGCCTGGGGC
GCTCCGTCGATCACTACGAAGTCGCGCCGGCCGATGGCCTTCACGTCGCGGTCAATCGTCGGGCGGTCGA
TGCCGACAACGGTTAGCGGTTGATCTTCCCGCACGGCCGCCCAATCGCGGGCACTGCCCTGGGGATCGGA
ATCGACTAACAGAACATCGGCCCCGGCGAGTTGCAGGGCGCGGGCTAGATGGGTTGCGATGGTCGTCTTG
CCTGACCCGCCTTTCTGGTTAAGTACAGCGATAACCTTCATGCGTTCCCCTTGCGTATTTGTTTATTTAC
TCATCGCATCATATACGCAGCGACCGCATGACGCAAGCTGTTTTACTCAAATACACATCACCTTTTTAGA
CGGCGGCGCTCGGTTTCTTCAGCGGCCAAGCTGGCCGGCCAGGCCGCCAGCTTGGCATCAGACAAACCGG
CCAGGATTTCATGCAGCCGCACGGTTGAGACGTGCGCGGGCGGCTCGAACACGTACCCGGCCGCGATCAT
CTCCGCCTCGATCTCTTCGGTAATGAAAAACGGTTCGTCCTGGCCGTCCTGGTGCGGTTTCATGCTTGTT
CCTCTTGGCGTTCATTCTCGGCGGCCGCCAGGGCGTCGGCCTCGGTCAATGCGTCCTCACGGAAGGCACC
GCGCCGCCTGGCCTCGGTGGGCGTCACTTCCTCGCTGCGCTCAAGTGCGCGGTACAGGGTCGAGCGATGC
ACGCCAAGCAGTGCAGCCGCCTCTTTCACGGTGCGGCCTTCCTGGTCGATCAGCTCGCGGGCGTGCGCGA
TCTGTGCCGGGGTGAGGGTAGGGCGGGGGCCAAACTTCACGCCTCGGGCCTTGGCGGCCTCGCGCCCGCT
CCGGGTGCGGTCGATGATTAGGGAACGCTCGAACTCGGCAATGCCGGCGAACACGGTCAACACCATGCGG
CCGGCCGGCGTGGTGGTGTCGGCCCACGGCTCTGCCAGGCTACGCAGGCCCGCGCCGGCCTCCTGGATGC
GCTCGGCAATGTCCAGTAGGTCGCGGGTGCTGCGGGCCAGGCGGTCTAGCCTGGTCACTGTCACAACGTC
GCCAGGGCGTAGGTGGTCAAGCATCCTGGCCAGCTCCGGGCGGTCGCGCCTGGTGCCGGTGATCTTCTCG
GAAAACAGCTTGGTGCAGCCGGCCGCGTGCAGTTCGGCCCGTTGGTTGGTCAAGTCCTGGTCGTCGGTGC
TGACGCGGGCATAGCCCAGCAGGCCAGCGGCGGCGCTCTTGTTCATGGCGTAATGTCTCCGGTTCTAGTC
GCAAGTATTCTACTTTATGCGACTAAAACACGCGACAAGAAAACGCCAGGAAAAGGGCAGGGCGGCAGCC
TGTCGCGTAACTTAGGACTTGTGCGACATGTCGTTTTCAGAAGACGGCTGCACTGAACGTCAGAAGCCGA
CTGCACTATAGCAGCGGAGGGGTTGGATCAAAGTACTTTGATCCCGAGGGGAACCCTGTGGTTGGCATGC
ACATACAAATGGACGAACGGATAAACCTTTTCACGCCCTTTTAAATATCCGTTATTCTAATAAACGCTCT
TTTCTCTTAGGTTTACCCGCCAATATATCCTGTCAAACACTGATAGTTTAAACTGAAGGCGGGAAACGAC
AATCTGATCCAAGCTCAAGCTAAGCTTGAGCTCTGCATGCCTGCAGGTCCCCAGATTAGCCTTTTCAATT
TCAGAAAGAATGCTAACCCACAGATGGTTAGAGAGGCTTACGCAGCAGGTCTCATCAAGACGATCTACCC
GAGCAATAATCTCCAGGAAATCAAATACCTTCCCAAGAAGGTTAAAGATGCAGTCAAAAGATTCAGGACT
AACTGCATCAAGAACACAGAGAAAGATATATTTCTCAAGATCAGAAGTACTATTCCAGTATGGACGATTC
AAGGCTTGCTTCACAAACCAAGGCAAGTAATAGAGATTGGAGTCTCTAAAAAGGTAGTTCCCACTGAATC
AAAGGCCATGGAGTCAAAGATTCAAATAGAGGACCTAACAGAACTCGCCGTAAAGACTGGCGAACAGTTC
ATACAGAGTCTCTTACGACTCAATGACAAGAAGAAAATCTTCGTCAACATGGTGGAGCACGACACACTTG
TCTACTCCAAAAATATCAAAGATACAGTCTCAGAAGACCAAAGGGCAATTGAGACTTTTCAACAAAGGGT
AATATCCGGAAACCTCCTCGGATTCCATTGCCCAGCTATCTGTCACTTTATTGTGAAGATAGTGGAAAAG
GAAGGTGGCTCCTACAAATGCCATCATTGCGATAAAGGAAAGGCCATCGTTGAAGATGCCTCTGCCGACA
GTGGTCCCAAAGATGGACCCCCACCCACGAGGAGCATCGTGGAAAAAGAAGACGTTCCAACCACGTCTTC
AAAGCAAGTGGATTGATGTGATATCTCCACTGACGTAAGGGATGACGCACAATCCCACTATCCTTCGCAA
GACCCTTCCTCTATATAAGGAAGTTCATTTCATTTGGAGAGAACACGGGGGACTCTAGAGGATCCAAGGA
GATATAAGCCGCCACCATGGATTACAAGGACGATGATGACAAGGACTACAAGGACGATGACGATAAGGAC
TATAAGGATGACGATGACAAGGATTACGCCGAGGATGGATGATACTAGCGGACAAACTTTCGTTTCTGTG
AACCTTTCTGACGAAAGCAACACGGCAAGCACTAAAGTTGAAAACGTAAAATCGGAAGCGGATCGCTTGG
AGTTTTTACGTAAAATGAATCCCTTTATTGTTGACGCTCTGGTGCGGAAAACCAATTATCAGGGTGCTCG
CTTTCGCGCAAGAATAATAGGAGTGTGCGTGGATTGTGGTAGAAAACACGACAAGGCGCTCAAGACTGAA
CGTAAGTGTAAGGTCAACAACACGCAATCTCAGAACGAGGTGGCGCATATGTTGATGCACGATCCCGTTA

AGGCGGCGAACAAAAGAAAGGCTAGAGCCTTTTCTAACGCAGAGATGTTTGCGATTGAACTGGTTTCGTA
CACCAAGGAAAGGCAATTGGCGGTCGATTTAGCCGCTGAAAGGGAGAAGACGAGACTGGCTCGTAGACAC
CCAATACGTTCTCCGGAAGAAACTCCGGAACATTATAAATTCGGTATGACTGCTAAGGCAATGTTACCGG
ACATCAACGCCGTAGACGTTGGTGATAACGAGGAAACTTCGTCGGAGTACCCAGTGAGTCTGAGTGTTTC
TGGCGGAGTTCTCCGTGAACACCACTTCATCTGACCTAATGATATCCCGCGGCCATGCTAGAGTCCGCAA
AAATCACCAGTCTCTCTCTACAAATCTATCTCTCTCTATTTTTCTCCAGAATAATGTGTGAGTAGTTCCC
AGATAAGGGAATTAGGGTTCTTATAGGGTTTCGCTCATGTGTTGAGCATATAAGAAACCCTTAGTATGTA
TTTGTATTTGTAAAATACTTCTATCAATAAAATTTCTAATTCCTAAAACCAAAATCCAGTGACCTGCAGG
CATGCGACGTCG

Sequence of pH7LIC3.1.1-p23M9 (35S- p23M9)

GGGTACCCTCGAATTATCATACATGAGAATTAAGGGAGTCACGTTATGACCCCGCCGATGACGCGGGACA
AGCCGTTTTACGTTTGGAACTGACAGAACCGCAACGTTGAAGGAGCCACTGAGCCGCGGGTTTCTGGAGT
TTAATGAGCTAAGCACATACGTCAGAAACCATTATTGCGCGTTCAAAAGTCGCCTAAGGTCACTATCAGC
TAGCAAATATTTCTTGTCAAAAATGCTCCACTGACGTTCCATAAATTCCCCTCGGTATCCAATTAGAGTC
TCATATTCACTCTCAACTCGAATCCCCCCTATGAAAAAGCCTGAACTCACCGCGACGTCTGTCGAGAAGT
TTCTGATCGAAAAGTTCGACAGCGTCTCCGACCTGATGCAGCTCTCGGAGGGCGAAGAATCTCGTGCTTT
CAGCTTCGATGTAGGAGGGCGTGGATATGTCCTGCGGGTAAATAGCTGCGCCGATGGTTTCTACAAAGAT
CGTTATGTTTATCGGCACTTTGCATCGGCCGCGCTCCCGATTCCGGAAGTGCTTGACATTGGGGAGTTCA
GCGAGAGCCTGACCTATTGCATCTCCCGCCGTGCACAGGGTGTCACGTTGCAAGACCTGCCTGAAACCGA
ACTGCCCGCTGTTCTTCAGCCGGTCGCGGAGGCTATGGATGCTATCGCTGCGGCCGATCTTAGCCAGACG
AGCGGGTTCGGCCCATTCGGACCGCAAGGAATCGGTCAATACACTACATGGCGTGATTTCATATGCGCGA
TTGCTGATCCCCATGTGTATCACTGGCAAACTGTGATGGACGACACCGTCAGTGCGTCCGTCGCGCAGGC
TCTCGATGAGCTGATGCTTTGGGCGAGGACTGCCCCGAAGTCCGGCACTCGTGCACGCGGATTCGGCTCC
AACAATGTCCTGACGGACAATGGCCGCATAACAGCGGTCATTGACTGGAGCGAGGCGATGTTCGGGGATT
CCCAATACGAGGTCGCCAACATCTTCTTCTGGAGGCCGTGGTTGGCTTGTATGGAGCAGCAGACGCGCTA
CTTCGAGCGGAGGCATCCGGAGCTTGCAGGATCGCCAAGGCTCCGGGCGTATATGCTCCGCATTGGTCTT
GACCAACTCTATCAGAGCTTGGTTGACGGCAATTTCGATGATGCAGCTTGGGCGCAGGGTCGATGCGACG
CAATCGTCCGATCCGGAGCCGGGACTGTCGGGCGTACACAAATCGCCCGCAGAAGCGCGGCCGTCTGGAC
CGATGGCTGTGTAGAAGTACTCGCCGATAGTGGAAACCGACGCCCCAGCACTCGTCCGAGGGCAAAGGAA
TAGAGTAGATGCCGACCGAACAAGAGCTGATTTCGAGAACGCCTCAGCCAGCAACTCGCGCGAGCCTAGC
AAGGCAAATGCGAGAGAACGGCCTTACGCTTGGTGGCACAGTTCTCGTCCACAGTTCGCTAAGCTCGCTC
GGCTGGGTCGCGGGAGGGCCGGTCGCAGTGATTCAGGAATTAATTCCCTAGAGTCAAGCAGATCGTTCAA
ACATTTGGCAATAAAGTTTCTTAAGATTGAATCCTGTTGCCGGTCTTGCGATGATTATCATATAATTTCT
GTTGAATTACGTTAAGCATGTAATAATTAACATGTAATGCATGACGTTATTTATGAGATGGGTTTTTATG
ATTAGAGTCCCGCAATTATACATTTAATACGCGATAGAAAACAAAATATAGCGCGCAAACTAGGATAAAT
TATCGCGCGCGGTGTCATCTATGTTACTAGATCGACCGGCATGCAAGCTGATAATTCAATTCGGCGTTAA
TTCAGTACATTAAAAACGTCCGCAATGTGTTATTAAGTTGTCTAAGCGTCAATTTGTTTACACCACAATA
TATCCTGCCACCAGCCAGCCAACAGCTCCCCGACCGGCAGCTCGGCACAAAATCACCACTCGATACAGGC
AGCCCATCAGTCCGGGACGGCGTCAGCGGGAGAGCCGTTGTAAGGCGGCAGACTTTGCTCATGTTACCGA
TGCTATTCGGAAGAACGGCAACTAAGCTGCCGGGTTTGAAACACGGATGATCTCGCGGAGGGTAGCATGT
TGATTGTAACGATGACAGAGCGTTGCTGCCTGTGATCAATTCGGGCACGAACCCAGTGGACATAAGCCTC
GTTCGGTTCGTAAGCTGTAATGCAAGTAGCGTAACTGCCGTCACGCAACTGGTCCAGAACCTTGACCGAA
CGCAGCGGTGGTAACGGCGCAGTGGCGGTTTTCATGGCTTCTTGTTATGACATGTTTTTTTGGGGTACAG

TCTATGCCTCGGGCATCCAAGCAGCAAGCGCGTTACGCCGTGGGTCGATGTTTGATGTTATGGAGCAGCA
ACGATGTTACGCAGCAGGGCAGTCGCCCTAAAACAAAGTTAAACATCATGGGGGAAGCGGTGATCGCCGA
AGTATCGACTCAACTATCAGAGGTAGTTGGCGTCATCGAGCGCCATCTCGAACCGACGTTGCTGGCCGTA
CATTTGTACGGCTCCGCAGTGGATGGCGGCCTGAAGCCACACAGTGATATTGATTTGCTGGTTACGGTGA
CCGTAAGGCTTGATGAAACAACGCGGCGAGCTTTGATCAACGACCTTTTGGAAACTTCGGCTTCCCCTGG
AGAGAGCGAGATTCTCCGCGCTGTAGAAGTCACCATTGTTGTGCACGACGACATCATTCCGTGGCGTTAT
CCAGCTAAGCGCGAACTGCAATTTGGAGAATGGCAGCGCAATGACATTCTTGCAGGTATCTTCGAGCCAG
CCACGATCGACATTGATCTGGCTATCTTGCTGACAAAAGCAAGAGAACATAGCGTTGCCTTGGTAGGTCC
AGCGGCGGAGGAACTCTTTGATCCGGTTCCTGAACAGGATCTATTTGAGGCGCTAAATGAAACCTTAACG
CTATGGAACTCGCCGCCCGACTGGGCTGGCGATGAGCGAAATGTAGTGCTTACGTTGTCCCGCATTTGGT
ACAGCGCAGTAACCGGCAAAATCGCGCCGAAGGATGTCGCTGCCGACTGGGCAATGGAGCGCCTGCCGGC
CCAGTATCAGCCCGTCATACTTGAAGCTAGACAGGCTTATCTTGGACAAGAAGAAGATCGCTTGGCCTCG
CGCGCAGATCAGTTGGAAGAATTTGTCCACTACGTGAAAGGCGAGATCACCAAGGTAGTCGGCAAATAAT
GTCTAGCTAGAAATTCGTTCAAGCCGACGCCGCTTCGCCGGCGTTAACTCAAGCGATTAGATGCACTAAG
CACATAATTGCTCACAGCCAAACTATCAGGTCAAGTCTGCTTTTATTATTTTTAAGCGTGCATAATAAGC
CCTACACAAATTGGGAGATATATCATGCATGACCAAAATCCCTTAACGTGAGTTTTCGTTCCACTGAGCG
TCAGACCCCGTAGAAAAGATCAAAGGATCTTCTTGAGATCCTTTTTTTCTGCGCGTAATCTGCTGCTTGC
AAACAAAAAAACCACCGCTACCAGCGGTGGTTTGTTTGCCGGATCAAGAGCTACCAACTCTTTTTCCGAA
GGTAACTGGCTTCAGCAGAGCGCAGATACCAAATACTGTCCTTCTAGTGTAGCCGTAGTTAGGCCACCAC
TTCAAGAACTCTGTAGCACCGCCTACATACCTCGCTCTGCTAATCCTGTTACCAGTGGCTGCTGCCAGTG
GCGATAAGTCGTGTCTTACCGGGTTGGACTCAAGACGATAGTTACCGGATAAGGCGCAGCGGTCGGGCTG
AACGGGGGGTTCGTGCACACAGCCCAGCTTGGAGCGAACGACCTACACCGAACTGAGATACCTACAGCGT
GAGCTATGAGAAAGCGCCACGCTTCCCGAAGGGAGAAAGGCGGACAGGTATCCGGTAAGCGGCAGGGTCG
GAACAGGAGAGCGCACGAGGGAGCTTCCAGGGGGAAACGCCTGGTATCTTTATAGTCCTGTCGGGTTTCG
CCACCTCTGACTTGAGCGTCGATTTTTGTGATGCTCGTCAGGGGGGCGGAGCCTATGGAAAAACGCCAGC
AACGCGGCCTTTTTACGGTTCCTGGCCTTTTGCTGGCCTTTTGCTCACATGTTCTTTCCTGCGTTATCCC
CTGATTCTGTGGATAACCGTATTACCGCCTTTGAGTGAGCTGATACCGCTCGCCGCAGCCGAACGACCGA
GCGCAGCGAGTCAGTGAGCGAGGAAGCGGAAGAGCGCCTGATGCGGTATTTTCTCCTTACGCATCTGTGC
GGTATTTCACACCGCATATGGTGCACTCTCAGTACAATCTGCTCTGATGCCGCATAGTTAAGCCAGTATA
CACTCCGCTATCGCTACGTGACTGGGTCATGGCTGCGCCCCGACACCCGCCAACACCCGCTGACGCGCCC
TGACGGGCTTGTCTGCTCCCGGCATCCGCTTACAGACAAGCTGTGACCGTCTCCGGGAGCTGCATGTGTC
AGAGGTTTTCACCGTCATCACCGAAACGCGCGAGGCAGGGTGCCTTGATGTGGGCGCCGGCGGTCGAGTG
GCGACGGCGCGGCTTGTCCGCGCCCTGGTAGATTGCCTGGCCGTAGGCCAGCCATTTTTGAGCGGCCAGC
GGCCGCGATAGGCCGACGCGAAGCGGCGGGGCGTAGGGAGCGCAGCGACCGAAGGGTAGGCGCTTTTTGC
AGCTCTTCGGCTGTGCGCTGGCCAGACAGTTATGCACAGGCCAGGCGGGTTTTAAGAGTTTTAATAAGTT
TTAAAGAGTTTTAGGCGGAAAAATCGCCTTTTTTCTCTTTTATATCAGTCACTTACATGTGTGACCGGTT
CCCAATGTACGGCTTTGGGTTCCCAATGTACGGGTTCCGGTTCCCAATGTACGGCTTTGGGTTCCCAATG
TACGTGCTATCCACAGGAAAGAGACCTTTTCGACCTTTTTCCCCTGCTAGGGCAATTTGCCCTAGCATCT
GCTCCGTACATTAGGAACCGGCGGATGCTTCGCCCTCGATCAGGTTGCGGTAGCGCATGACTAGGATCGG
GCCAGCCTGCCCCGCCTCCTCCTTCAAATCGTACTCCGGCAGGTCATTTGACCCGATCAGCTTGCGCACG
GTGAAACAGAACTTCTTGAACTCTCCGGCGCTGCCACTGCGTTCGTAGATCGTCTTGAACAACCATCTGG
CTTCTGCCTTGCCTGCGGCGCGGCGTGCCAGGCGGTAGAGAAAACGGCCGATGCCGGGATCGATCAAAAA
GTAATCGGGGTGAACCGTCAGCACGTCCGGGTTCTTGCCTTCTGTGATCTCGCGGTACATCCAATCAGCT
AGCTCGATCTCGATGTACTCCGGCCGCCCGGTTTCGCTCTTTACGATCTTGTAGCGGCTAATCAAGGCTT

CACCCTCGGATACCGTCACCAGGCGGCCGTTCTTGGCCTTCTTCGTACGCTGCATGGCAACGTGCGTGGT
GTTTAACCGAATGCAGGTTTCTACCAGGTCGTCTTTCTGCTTTCCGCCATCGGCTCGCCGGCAGAACTTG
AGTACGTCCGCAACGTGTGGACGGAACACGCGGCCGGGCTTGTCTCCCTTCCCTTCCCGGTATCGGTTCA
TGGATTCGGTTAGATGGGAAACCGCCATCAGTACCAGGTCGTAATCCCACACACTGGCCATGCCGGCCGG
CCCTGCGGAAACCTCTACGTGCCCGTCTGGAAGCTCGTAGCGGATCACCTCGCCAGCTCGTCGGTCACGC
TTCGACAGACGGAAAACGGCCACGTCCATGATGCTGCGACTATCGCGGGTGCCCACGTCATAGAGCATCG
GAACGAAAAAATCTGGTTGCTCGTCGCCCTTGGGCGGCTTCCTAATCGACGGCGCACCGGCTGCCGGCGG
TTGCCGGGATTCTTTGCGGATTCGATCAGCGGCCGCTTGCCACGATTCACCGGGGCGTGCTTCTGCCTCG
ATGCGTTGCCGCTGGGCGGCCTGCGCGGCCTTCAACTTCTCCACCAGGTCATCACCCAGCGCCGCGCCGA
TTTGTACCGGGCCGGATGGTTTGCGACCGTCACGCCGATTCCTCGGGCTTGGGGGTTCCAGTGCCATTGC
AGGGCCGGCAGACAACCCAGCCGCTTACGCCTGGCCAACCGCCCGTTCCTCCACACATGGGGCATTCCAC
GGCGTCGGTGCCTGGTTGTTCTTGATTTTCCATGCCGCCTCCTTTAGCCGCTAAAATTCATCTACTCATT
TATTCATTTGCTCATTTACTCTGGTAGCTGCGCGATGTATTCAGATAGCAGCTCGGTAATGGTCTTGCCT
TGGCGTACCGCGTACATCTTCAGCTTGGTGTGATCCTCCGCCGGCAACTGAAAGTTGACCCGCTTCATGG
CTGGCGTGTCTGCCAGGCTGGCCAACGTTGCAGCCTTGCTGCTGCGTGCGCTCGGACGGCCGGCACTTAG
CGTGTTTGTGCTTTTGCTCATTTTCTCTTTACCTCATTAACTCAAATGAGTTTTGATTTAATTTCAGCGG
CCAGCGCCTGGACCTCGCGGGCAGCGTCGCCCTCGGGTTCTGATTCAAGAACGGTTGTGCCGGCGGCGGC
AGTGCCTGGGTAGCTCACGCGCTGCGTGATACGGGACTCAAGAATGGGCAGCTCGTACCCGGCCAGCGCC
TCGGCAACCTCACCGCCGATGCGCGTGCCTTTGATCGCCCGCGACACGACAAAGGCCGCTTGTAGCCTTC
CATCCGTGACCTCAATGCGCTGCTTAACCAGCTCCACCAGGTCGGCGGTGGCCCATATGTCGTAAGGGCT
TGGCTGCACCGGAATCAGCACGAAGTCGGCTGCCTTGATCGCGGACACAGCCAAGTCCGCCGCCTGGGGC
GCTCCGTCGATCACTACGAAGTCGCGCCGGCCGATGGCCTTCACGTCGCGGTCAATCGTCGGGCGGTCGA
TGCCGACAACGGTTAGCGGTTGATCTTCCCGCACGGCCGCCCAATCGCGGGCACTGCCCTGGGGATCGGA
ATCGACTAACAGAACATCGGCCCCGGCGAGTTGCAGGGCGCGGGCTAGATGGGTTGCGATGGTCGTCTTG
CCTGACCCGCCTTTCTGGTTAAGTACAGCGATAACCTTCATGCGTTCCCCTTGCGTATTTGTTTATTTAC
TCATCGCATCATATACGCAGCGACCGCATGACGCAAGCTGTTTTACTCAAATACACATCACCTTTTTAGA
CGGCGGCGCTCGGTTTCTTCAGCGGCCAAGCTGGCCGGCCAGGCCGCCAGCTTGGCATCAGACAAACCGG
CCAGGATTTCATGCAGCCGCACGGTTGAGACGTGCGCGGGCGGCTCGAACACGTACCCGGCCGCGATCAT
CTCCGCCTCGATCTCTTCGGTAATGAAAAACGGTTCGTCCTGGCCGTCCTGGTGCGGTTTCATGCTTGTT
CCTCTTGGCGTTCATTCTCGGCGGCCGCCAGGGCGTCGGCCTCGGTCAATGCGTCCTCACGGAAGGCACC
GCGCCGCCTGGCCTCGGTGGGCGTCACTTCCTCGCTGCGCTCAAGTGCGCGGTACAGGGTCGAGCGATGC
ACGCCAAGCAGTGCAGCCGCCTCTTTCACGGTGCGGCCTTCCTGGTCGATCAGCTCGCGGGCGTGCGCGA
TCTGTGCCGGGGTGAGGGTAGGGCGGGGGCCAAACTTCACGCCTCGGGCCTTGGCGGCCTCGCGCCCGCT
CCGGGTGCGGTCGATGATTAGGGAACGCTCGAACTCGGCAATGCCGGCGAACACGGTCAACACCATGCGG
CCGGCCGGCGTGGTGGTGTCGGCCCACGGCTCTGCCAGGCTACGCAGGCCCGCGCCGGCCTCCTGGATGC
GCTCGGCAATGTCCAGTAGGTCGCGGGTGCTGCGGGCCAGGCGGTCTAGCCTGGTCACTGTCACAACGTC
GCCAGGGCGTAGGTGGTCAAGCATCCTGGCCAGCTCCGGGCGGTCGCGCCTGGTGCCGGTGATCTTCTCG
GAAAACAGCTTGGTGCAGCCGGCCGCGTGCAGTTCGGCCCGTTGGTTGGTCAAGTCCTGGTCGTCGGTGC
TGACGCGGGCATAGCCCAGCAGGCCAGCGGCGGCGCTCTTGTTCATGGCGTAATGTCTCCGGTTCTAGTC
GCAAGTATTCTACTTTATGCGACTAAAACACGCGACAAGAAAACGCCAGGAAAAGGGCAGGGCGGCAGCC
TGTCGCGTAACTTAGGACTTGTGCGACATGTCGTTTTCAGAAGACGGCTGCACTGAACGTCAGAAGCCGA
CTGCACTATAGCAGCGGAGGGGTTGGATCAAAGTACTTTGATCCCGAGGGGAACCCTGTGGTTGGCATGC
ACATACAAATGGACGAACGGATAAACCTTTTCACGCCCTTTTAAATATCCGTTATTCTAATAAACGCTCT
TTTCTCTTAGGTTTACCCGCCAATATATCCTGTCAAACACTGATAGTTTAAACTGAAGGCGGGAAACGAC

AATCTGATCCAAGCTCAAGCTAAGCTTGAGCTCTGCATGCCTGCAGGTCCCCAGATTAGCCTTTTCAATT
TCAGAAAGAATGCTAACCCACAGATGGTTAGAGAGGCTTACGCAGCAGGTCTCATCAAGACGATCTACCC
GAGCAATAATCTCCAGGAAATCAAATACCTTCCCAAGAAGGTTAAAGATGCAGTCAAAAGATTCAGGACT
AACTGCATCAAGAACACAGAGAAAGATATATTTCTCAAGATCAGAAGTACTATTCCAGTATGGACGATTC
AAGGCTTGCTTCACAAACCAAGGCAAGTAATAGAGATTGGAGTCTCTAAAAAGGTAGTTCCCACTGAATC
AAAGGCCATGGAGTCAAAGATTCAAATAGAGGACCTAACAGAACTCGCCGTAAAGACTGGCGAACAGTTC
ATACAGAGTCTCTTACGACTCAATGACAAGAAGAAAATCTTCGTCAACATGGTGGAGCACGACACACTTG
TCTACTCCAAAAATATCAAAGATACAGTCTCAGAAGACCAAAGGGCAATTGAGACTTTTCAACAAAGGGT
AATATCCGGAAACCTCCTCGGATTCCATTGCCCAGCTATCTGTCACTTTATTGTGAAGATAGTGGAAAAG
GAAGGTGGCTCCTACAAATGCCATCATTGCGATAAAGGAAAGGCCATCGTTGAAGATGCCTCTGCCGACA
GTGGTCCCAAAGATGGACCCCCACCCACGAGGAGCATCGTGGAAAAAGAAGACGTTCCAACCACGTCTTC
AAAGCAAGTGGATTGATGTGATATCTCCACTGACGTAAGGGATGACGCACAATCCCACTATCCTTCGCAA
GACCCTTCCTCTATATAAGGAAGTTCATTTCATTTGGAGAGAACACGGGGGACTCTAGAGGATCCAAGGA
GATATAAGCCGCCACCATGGATTACAAGGACGATGATGACAAGGACTACAAGGACGATGACGATAAGGAC
TATAAGGATGACGATGACAAGGATTACGCCGAGGATGGATGATACTAGCGGACAAACTTTCGTTTCTGTG
AACCTTTCTGACGAAAGCAACACGGCAAGCACTAAAGTTGAAAACGTAAAATCGGAAGCGGATCGCTTGG
AGTTTTTACGTAAAATGAATCCCTTTATTGTTGACGCTCTGGTGCGGAAAACCAATTATCAGGGTGCTCG
CTTTCGCGCAAGAATAATAGGAGTGTGCGTGGATTGTGGTAGAAAACACGACAAGGCGCTCAAGACTGAA
CGTAAGTGTAAGGTCAACAACACGCAATCTCAGAACGAGGTGGCGCATATGTTGATGCACGATCCCGTTA
AGTATTTGAACAAAAGAAAGGCTGCGGCCTTTTCTAACGCAGAGATGTTTGCGATTGAACTGGTTTCGTA
CACCAAGGAAAGGCAATTGGCGGTCGATTTAGCCGCTGAAAGGGAGAAGACGAGACTGGCTCGTAGACAC
CCAATACGTTCTCCGGAAGAAACTCCGGAACATTATAAATTCGGTATGACTGCTAAGGCAATGTTACCGG
ACATCAACGCCGTAGACGTTGGTGATAACGAGGAAACTTCGTCGGAGTACCCAGTGAGTCTGAGTGTTTC
TGGCGGAGTTCTCCGTGAACACCACTTCATCTGACCTAATGATATCCCGCGGCCATGCTAGAGTCCGCAA
AAATCACCAGTCTCTCTCTACAAATCTATCTCTCTCTATTTTTCTCCAGAATAATGTGTGAGTAGTTCCC
AGATAAGGGAATTAGGGTTCTTATAGGGTTTCGCTCATGTGTTGAGCATATAAGAAACCCTTAGTATGTA
TTTGTATTTGTAAAATACTTCTATCAATAAAATTTCTAATTCCTAAAACCAAAATCCAGTGACCTGCAGG
CATGCGACGTCG

Sequence of pH7LIC3.1.1-p23M10 (35S- p23M10)

GGGTACCCTCGAATTATCATACATGAGAATTAAGGGAGTCACGTTATGACCCCGCCGATGACGCGGGACA
AGCCGTTTTACGTTTGGAACTGACAGAACCGCAACGTTGAAGGAGCCACTGAGCCGCGGGTTTCTGGAGT
TTAATGAGCTAAGCACATACGTCAGAAACCATTATTGCGCGTTCAAAAGTCGCCTAAGGTCACTATCAGC
TAGCAAATATTTCTTGTCAAAAATGCTCCACTGACGTTCCATAAATTCCCCTCGGTATCCAATTAGAGTC
TCATATTCACTCTCAACTCGAATCCCCCCTATGAAAAAGCCTGAACTCACCGCGACGTCTGTCGAGAAGT
TTCTGATCGAAAAGTTCGACAGCGTCTCCGACCTGATGCAGCTCTCGGAGGGCGAAGAATCTCGTGCTTT
CAGCTTCGATGTAGGAGGGCGTGGATATGTCCTGCGGGTAAATAGCTGCGCCGATGGTTTCTACAAAGAT
CGTTATGTTTATCGGCACTTTGCATCGGCCGCGCTCCCGATTCCGGAAGTGCTTGACATTGGGGAGTTCA
GCGAGAGCCTGACCTATTGCATCTCCCGCCGTGCACAGGGTGTCACGTTGCAAGACCTGCCTGAAACCGA
ACTGCCCGCTGTTCTTCAGCCGGTCGCGGAGGCTATGGATGCTATCGCTGCGGCCGATCTTAGCCAGACG
AGCGGGTTCGGCCCATTCGGACCGCAAGGAATCGGTCAATACACTACATGGCGTGATTTCATATGCGCGA
TTGCTGATCCCCATGTGTATCACTGGCAAACTGTGATGGACGACACCGTCAGTGCGTCCGTCGCGCAGGC
TCTCGATGAGCTGATGCTTTGGGCGAGGACTGCCCCGAAGTCCGGCACTCGTGCACGCGGATTCGGCTCC
AACAATGTCCTGACGGACAATGGCCGCATAACAGCGGTCATTGACTGGAGCGAGGCGATGTTCGGGGATT

CCCAATACGAGGTCGCCAACATCTTCTTCTGGAGGCCGTGGTTGGCTTGTATGGAGCAGCAGACGCGCTA
CTTCGAGCGGAGGCATCCGGAGCTTGCAGGATCGCCAAGGCTCCGGGCGTATATGCTCCGCATTGGTCTT
GACCAACTCTATCAGAGCTTGGTTGACGGCAATTTCGATGATGCAGCTTGGGCGCAGGGTCGATGCGACG
CAATCGTCCGATCCGGAGCCGGGACTGTCGGGCGTACACAAATCGCCCGCAGAAGCGCGGCCGTCTGGAC
CGATGGCTGTGTAGAAGTACTCGCCGATAGTGGAAACCGACGCCCCAGCACTCGTCCGAGGGCAAAGGAA
TAGAGTAGATGCCGACCGAACAAGAGCTGATTTCGAGAACGCCTCAGCCAGCAACTCGCGCGAGCCTAGC
AAGGCAAATGCGAGAGAACGGCCTTACGCTTGGTGGCACAGTTCTCGTCCACAGTTCGCTAAGCTCGCTC
GGCTGGGTCGCGGGAGGGCCGGTCGCAGTGATTCAGGAATTAATTCCCTAGAGTCAAGCAGATCGTTCAA
ACATTTGGCAATAAAGTTTCTTAAGATTGAATCCTGTTGCCGGTCTTGCGATGATTATCATATAATTTCT
GTTGAATTACGTTAAGCATGTAATAATTAACATGTAATGCATGACGTTATTTATGAGATGGGTTTTTATG
ATTAGAGTCCCGCAATTATACATTTAATACGCGATAGAAAACAAAATATAGCGCGCAAACTAGGATAAAT
TATCGCGCGCGGTGTCATCTATGTTACTAGATCGACCGGCATGCAAGCTGATAATTCAATTCGGCGTTAA
TTCAGTACATTAAAAACGTCCGCAATGTGTTATTAAGTTGTCTAAGCGTCAATTTGTTTACACCACAATA
TATCCTGCCACCAGCCAGCCAACAGCTCCCCGACCGGCAGCTCGGCACAAAATCACCACTCGATACAGGC
AGCCCATCAGTCCGGGACGGCGTCAGCGGGAGAGCCGTTGTAAGGCGGCAGACTTTGCTCATGTTACCGA
TGCTATTCGGAAGAACGGCAACTAAGCTGCCGGGTTTGAAACACGGATGATCTCGCGGAGGGTAGCATGT
TGATTGTAACGATGACAGAGCGTTGCTGCCTGTGATCAATTCGGGCACGAACCCAGTGGACATAAGCCTC
GTTCGGTTCGTAAGCTGTAATGCAAGTAGCGTAACTGCCGTCACGCAACTGGTCCAGAACCTTGACCGAA
CGCAGCGGTGGTAACGGCGCAGTGGCGGTTTTCATGGCTTCTTGTTATGACATGTTTTTTTGGGGTACAG
TCTATGCCTCGGGCATCCAAGCAGCAAGCGCGTTACGCCGTGGGTCGATGTTTGATGTTATGGAGCAGCA
ACGATGTTACGCAGCAGGGCAGTCGCCCTAAAACAAAGTTAAACATCATGGGGGAAGCGGTGATCGCCGA
AGTATCGACTCAACTATCAGAGGTAGTTGGCGTCATCGAGCGCCATCTCGAACCGACGTTGCTGGCCGTA
CATTTGTACGGCTCCGCAGTGGATGGCGGCCTGAAGCCACACAGTGATATTGATTTGCTGGTTACGGTGA
CCGTAAGGCTTGATGAAACAACGCGGCGAGCTTTGATCAACGACCTTTTGGAAACTTCGGCTTCCCCTGG
AGAGAGCGAGATTCTCCGCGCTGTAGAAGTCACCATTGTTGTGCACGACGACATCATTCCGTGGCGTTAT
CCAGCTAAGCGCGAACTGCAATTTGGAGAATGGCAGCGCAATGACATTCTTGCAGGTATCTTCGAGCCAG
CCACGATCGACATTGATCTGGCTATCTTGCTGACAAAAGCAAGAGAACATAGCGTTGCCTTGGTAGGTCC
AGCGGCGGAGGAACTCTTTGATCCGGTTCCTGAACAGGATCTATTTGAGGCGCTAAATGAAACCTTAACG
CTATGGAACTCGCCGCCCGACTGGGCTGGCGATGAGCGAAATGTAGTGCTTACGTTGTCCCGCATTTGGT
ACAGCGCAGTAACCGGCAAAATCGCGCCGAAGGATGTCGCTGCCGACTGGGCAATGGAGCGCCTGCCGGC
CCAGTATCAGCCCGTCATACTTGAAGCTAGACAGGCTTATCTTGGACAAGAAGAAGATCGCTTGGCCTCG
CGCGCAGATCAGTTGGAAGAATTTGTCCACTACGTGAAAGGCGAGATCACCAAGGTAGTCGGCAAATAAT
GTCTAGCTAGAAATTCGTTCAAGCCGACGCCGCTTCGCCGGCGTTAACTCAAGCGATTAGATGCACTAAG
CACATAATTGCTCACAGCCAAACTATCAGGTCAAGTCTGCTTTTATTATTTTTAAGCGTGCATAATAAGC
CCTACACAAATTGGGAGATATATCATGCATGACCAAAATCCCTTAACGTGAGTTTTCGTTCCACTGAGCG
TCAGACCCCGTAGAAAAGATCAAAGGATCTTCTTGAGATCCTTTTTTTCTGCGCGTAATCTGCTGCTTGC
AAACAAAAAAACCACCGCTACCAGCGGTGGTTTGTTTGCCGGATCAAGAGCTACCAACTCTTTTTCCGAA
GGTAACTGGCTTCAGCAGAGCGCAGATACCAAATACTGTCCTTCTAGTGTAGCCGTAGTTAGGCCACCAC
TTCAAGAACTCTGTAGCACCGCCTACATACCTCGCTCTGCTAATCCTGTTACCAGTGGCTGCTGCCAGTG
GCGATAAGTCGTGTCTTACCGGGTTGGACTCAAGACGATAGTTACCGGATAAGGCGCAGCGGTCGGGCTG
AACGGGGGGTTCGTGCACACAGCCCAGCTTGGAGCGAACGACCTACACCGAACTGAGATACCTACAGCGT
GAGCTATGAGAAAGCGCCACGCTTCCCGAAGGGAGAAAGGCGGACAGGTATCCGGTAAGCGGCAGGGTCG
GAACAGGAGAGCGCACGAGGGAGCTTCCAGGGGGAAACGCCTGGTATCTTTATAGTCCTGTCGGGTTTCG
CCACCTCTGACTTGAGCGTCGATTTTTGTGATGCTCGTCAGGGGGGCGGAGCCTATGGAAAAACGCCAGC

AACGCGGCCTTTTTACGGTTCCTGGCCTTTTGCTGGCCTTTTGCTCACATGTTCTTTCCTGCGTTATCCC
CTGATTCTGTGGATAACCGTATTACCGCCTTTGAGTGAGCTGATACCGCTCGCCGCAGCCGAACGACCGA
GCGCAGCGAGTCAGTGAGCGAGGAAGCGGAAGAGCGCCTGATGCGGTATTTTCTCCTTACGCATCTGTGC
GGTATTTCACACCGCATATGGTGCACTCTCAGTACAATCTGCTCTGATGCCGCATAGTTAAGCCAGTATA
CACTCCGCTATCGCTACGTGACTGGGTCATGGCTGCGCCCCGACACCCGCCAACACCCGCTGACGCGCCC
TGACGGGCTTGTCTGCTCCCGGCATCCGCTTACAGACAAGCTGTGACCGTCTCCGGGAGCTGCATGTGTC
AGAGGTTTTCACCGTCATCACCGAAACGCGCGAGGCAGGGTGCCTTGATGTGGGCGCCGGCGGTCGAGTG
GCGACGGCGCGGCTTGTCCGCGCCCTGGTAGATTGCCTGGCCGTAGGCCAGCCATTTTTGAGCGGCCAGC
GGCCGCGATAGGCCGACGCGAAGCGGCGGGGCGTAGGGAGCGCAGCGACCGAAGGGTAGGCGCTTTTTGC
AGCTCTTCGGCTGTGCGCTGGCCAGACAGTTATGCACAGGCCAGGCGGGTTTTAAGAGTTTTAATAAGTT
TTAAAGAGTTTTAGGCGGAAAAATCGCCTTTTTTCTCTTTTATATCAGTCACTTACATGTGTGACCGGTT
CCCAATGTACGGCTTTGGGTTCCCAATGTACGGGTTCCGGTTCCCAATGTACGGCTTTGGGTTCCCAATG
TACGTGCTATCCACAGGAAAGAGACCTTTTCGACCTTTTTCCCCTGCTAGGGCAATTTGCCCTAGCATCT
GCTCCGTACATTAGGAACCGGCGGATGCTTCGCCCTCGATCAGGTTGCGGTAGCGCATGACTAGGATCGG
GCCAGCCTGCCCCGCCTCCTCCTTCAAATCGTACTCCGGCAGGTCATTTGACCCGATCAGCTTGCGCACG
GTGAAACAGAACTTCTTGAACTCTCCGGCGCTGCCACTGCGTTCGTAGATCGTCTTGAACAACCATCTGG
CTTCTGCCTTGCCTGCGGCGCGGCGTGCCAGGCGGTAGAGAAAACGGCCGATGCCGGGATCGATCAAAAA
GTAATCGGGGTGAACCGTCAGCACGTCCGGGTTCTTGCCTTCTGTGATCTCGCGGTACATCCAATCAGCT
AGCTCGATCTCGATGTACTCCGGCCGCCCGGTTTCGCTCTTTACGATCTTGTAGCGGCTAATCAAGGCTT
CACCCTCGGATACCGTCACCAGGCGGCCGTTCTTGGCCTTCTTCGTACGCTGCATGGCAACGTGCGTGGT
GTTTAACCGAATGCAGGTTTCTACCAGGTCGTCTTTCTGCTTTCCGCCATCGGCTCGCCGGCAGAACTTG
AGTACGTCCGCAACGTGTGGACGGAACACGCGGCCGGGCTTGTCTCCCTTCCCTTCCCGGTATCGGTTCA
TGGATTCGGTTAGATGGGAAACCGCCATCAGTACCAGGTCGTAATCCCACACACTGGCCATGCCGGCCGG
CCCTGCGGAAACCTCTACGTGCCCGTCTGGAAGCTCGTAGCGGATCACCTCGCCAGCTCGTCGGTCACGC
TTCGACAGACGGAAAACGGCCACGTCCATGATGCTGCGACTATCGCGGGTGCCCACGTCATAGAGCATCG
GAACGAAAAAATCTGGTTGCTCGTCGCCCTTGGGCGGCTTCCTAATCGACGGCGCACCGGCTGCCGGCGG
TTGCCGGGATTCTTTGCGGATTCGATCAGCGGCCGCTTGCCACGATTCACCGGGGCGTGCTTCTGCCTCG
ATGCGTTGCCGCTGGGCGGCCTGCGCGGCCTTCAACTTCTCCACCAGGTCATCACCCAGCGCCGCGCCGA
TTTGTACCGGGCCGGATGGTTTGCGACCGTCACGCCGATTCCTCGGGCTTGGGGGTTCCAGTGCCATTGC
AGGGCCGGCAGACAACCCAGCCGCTTACGCCTGGCCAACCGCCCGTTCCTCCACACATGGGGCATTCCAC
GGCGTCGGTGCCTGGTTGTTCTTGATTTTCCATGCCGCCTCCTTTAGCCGCTAAAATTCATCTACTCATT
TATTCATTTGCTCATTTACTCTGGTAGCTGCGCGATGTATTCAGATAGCAGCTCGGTAATGGTCTTGCCT
TGGCGTACCGCGTACATCTTCAGCTTGGTGTGATCCTCCGCCGGCAACTGAAAGTTGACCCGCTTCATGG
CTGGCGTGTCTGCCAGGCTGGCCAACGTTGCAGCCTTGCTGCTGCGTGCGCTCGGACGGCCGGCACTTAG
CGTGTTTGTGCTTTTGCTCATTTTCTCTTTACCTCATTAACTCAAATGAGTTTTGATTTAATTTCAGCGG
CCAGCGCCTGGACCTCGCGGGCAGCGTCGCCCTCGGGTTCTGATTCAAGAACGGTTGTGCCGGCGGCGGC
AGTGCCTGGGTAGCTCACGCGCTGCGTGATACGGGACTCAAGAATGGGCAGCTCGTACCCGGCCAGCGCC
TCGGCAACCTCACCGCCGATGCGCGTGCCTTTGATCGCCCGCGACACGACAAAGGCCGCTTGTAGCCTTC
CATCCGTGACCTCAATGCGCTGCTTAACCAGCTCCACCAGGTCGGCGGTGGCCCATATGTCGTAAGGGCT
TGGCTGCACCGGAATCAGCACGAAGTCGGCTGCCTTGATCGCGGACACAGCCAAGTCCGCCGCCTGGGGC
GCTCCGTCGATCACTACGAAGTCGCGCCGGCCGATGGCCTTCACGTCGCGGTCAATCGTCGGGCGGTCGA
TGCCGACAACGGTTAGCGGTTGATCTTCCCGCACGGCCGCCCAATCGCGGGCACTGCCCTGGGGATCGGA
ATCGACTAACAGAACATCGGCCCCGGCGAGTTGCAGGGCGCGGGCTAGATGGGTTGCGATGGTCGTCTTG
CCTGACCCGCCTTTCTGGTTAAGTACAGCGATAACCTTCATGCGTTCCCCTTGCGTATTTGTTTATTTAC

TCATCGCATCATATACGCAGCGACCGCATGACGCAAGCTGTTTTACTCAAATACACATCACCTTTTTAGA
CGGCGGCGCTCGGTTTCTTCAGCGGCCAAGCTGGCCGGCCAGGCCGCCAGCTTGGCATCAGACAAACCGG
CCAGGATTTCATGCAGCCGCACGGTTGAGACGTGCGCGGGCGGCTCGAACACGTACCCGGCCGCGATCAT
CTCCGCCTCGATCTCTTCGGTAATGAAAAACGGTTCGTCCTGGCCGTCCTGGTGCGGTTTCATGCTTGTT
CCTCTTGGCGTTCATTCTCGGCGGCCGCCAGGGCGTCGGCCTCGGTCAATGCGTCCTCACGGAAGGCACC
GCGCCGCCTGGCCTCGGTGGGCGTCACTTCCTCGCTGCGCTCAAGTGCGCGGTACAGGGTCGAGCGATGC
ACGCCAAGCAGTGCAGCCGCCTCTTTCACGGTGCGGCCTTCCTGGTCGATCAGCTCGCGGGCGTGCGCGA
TCTGTGCCGGGGTGAGGGTAGGGCGGGGGCCAAACTTCACGCCTCGGGCCTTGGCGGCCTCGCGCCCGCT
CCGGGTGCGGTCGATGATTAGGGAACGCTCGAACTCGGCAATGCCGGCGAACACGGTCAACACCATGCGG
CCGGCCGGCGTGGTGGTGTCGGCCCACGGCTCTGCCAGGCTACGCAGGCCCGCGCCGGCCTCCTGGATGC
GCTCGGCAATGTCCAGTAGGTCGCGGGTGCTGCGGGCCAGGCGGTCTAGCCTGGTCACTGTCACAACGTC
GCCAGGGCGTAGGTGGTCAAGCATCCTGGCCAGCTCCGGGCGGTCGCGCCTGGTGCCGGTGATCTTCTCG
GAAAACAGCTTGGTGCAGCCGGCCGCGTGCAGTTCGGCCCGTTGGTTGGTCAAGTCCTGGTCGTCGGTGC
TGACGCGGGCATAGCCCAGCAGGCCAGCGGCGGCGCTCTTGTTCATGGCGTAATGTCTCCGGTTCTAGTC
GCAAGTATTCTACTTTATGCGACTAAAACACGCGACAAGAAAACGCCAGGAAAAGGGCAGGGCGGCAGCC
TGTCGCGTAACTTAGGACTTGTGCGACATGTCGTTTTCAGAAGACGGCTGCACTGAACGTCAGAAGCCGA
CTGCACTATAGCAGCGGAGGGGTTGGATCAAAGTACTTTGATCCCGAGGGGAACCCTGTGGTTGGCATGC
ACATACAAATGGACGAACGGATAAACCTTTTCACGCCCTTTTAAATATCCGTTATTCTAATAAACGCTCT
TTTCTCTTAGGTTTACCCGCCAATATATCCTGTCAAACACTGATAGTTTAAACTGAAGGCGGGAAACGAC
AATCTGATCCAAGCTCAAGCTAAGCTTGAGCTCTGCATGCCTGCAGGTCCCCAGATTAGCCTTTTCAATT
TCAGAAAGAATGCTAACCCACAGATGGTTAGAGAGGCTTACGCAGCAGGTCTCATCAAGACGATCTACCC
GAGCAATAATCTCCAGGAAATCAAATACCTTCCCAAGAAGGTTAAAGATGCAGTCAAAAGATTCAGGACT
AACTGCATCAAGAACACAGAGAAAGATATATTTCTCAAGATCAGAAGTACTATTCCAGTATGGACGATTC
AAGGCTTGCTTCACAAACCAAGGCAAGTAATAGAGATTGGAGTCTCTAAAAAGGTAGTTCCCACTGAATC
AAAGGCCATGGAGTCAAAGATTCAAATAGAGGACCTAACAGAACTCGCCGTAAAGACTGGCGAACAGTTC
ATACAGAGTCTCTTACGACTCAATGACAAGAAGAAAATCTTCGTCAACATGGTGGAGCACGACACACTTG
TCTACTCCAAAAATATCAAAGATACAGTCTCAGAAGACCAAAGGGCAATTGAGACTTTTCAACAAAGGGT
AATATCCGGAAACCTCCTCGGATTCCATTGCCCAGCTATCTGTCACTTTATTGTGAAGATAGTGGAAAAG
GAAGGTGGCTCCTACAAATGCCATCATTGCGATAAAGGAAAGGCCATCGTTGAAGATGCCTCTGCCGACA
GTGGTCCCAAAGATGGACCCCCACCCACGAGGAGCATCGTGGAAAAAGAAGACGTTCCAACCACGTCTTC
AAAGCAAGTGGATTGATGTGATATCTCCACTGACGTAAGGGATGACGCACAATCCCACTATCCTTCGCAA
GACCCTTCCTCTATATAAGGAAGTTCATTTCATTTGGAGAGAACACGGGGGACTCTAGAGGATCCAAGGA
GATATAAGCCGCCACCATGGATTACAAGGACGATGATGACAAGGACTACAAGGACGATGACGATAAGGAC
TATAAGGATGACGATGACAAGGATTACGCCGAGGATGGATGATACTAGCGGACAAACTTTCGTTTCTGTG
AACCTTTCTGACGAAAGCAACACGGCAAGCACTAAAGTTGAAAACGTAAAATCGGAAGCGGATCGCTTGG
AGTTTTTACGTAAAATGAATCCCTTTATTGTTGACGCTCTGGTGCGGAAAACCAATTATCAGGGTGCTCG
CTTTCGCGCAAGAATAATAGGAGTGTGCGTGGATTGTGGTAGAAAACACGACAAGGCGCTCAAGACTGAA
CGTAAGTGTAAGGTCAACAACACGCAATCTCAGAACGAGGTGGCGCATATGTTGATGCACGATCCCGTTA
AGTATTTGAACAAAAGAAAGGCTAGAGCCGCGTCTAACGCAGAGATGTTTGCGATTGAACTGGTTTCGTA
CACCAAGGAAAGGCAATTGGCGGTCGATTTAGCCGCTGAAAGGGAGAAGACGAGACTGGCTCGTAGACAC
CCAATACGTTCTCCGGAAGAAACTCCGGAACATTATAAATTCGGTATGACTGCTAAGGCAATGTTACCGG
ACATCAACGCCGTAGACGTTGGTGATAACGAGGAAACTTCGTCGGAGTACCCAGTGAGTCTGAGTGTTTC
TGGCGGAGTTCTCCGTGAACACCACTTCATCTGACCTAATGATATCCCGCGGCCATGCTAGAGTCCGCAA
AAATCACCAGTCTCTCTCTACAAATCTATCTCTCTCTATTTTTCTCCAGAATAATGTGTGAGTAGTTCCC

AGATAAGGGAATTAGGGTTCTTATAGGGTTTCGCTCATGTGTTGAGCATATAAGAAACCCTTAGTATGTA
TTTGTATTTGTAAAATACTTCTATCAATAAAATTTCTAATTCCTAAAACCAAAATCCAGTGACCTGCAGG
CATGCGACGTCG

Sequence of pH7LIC3.1.1-p23M11 (35S- p23M11)

GGGTACCCTCGAATTATCATACATGAGAATTAAGGGAGTCACGTTATGACCCCGCCGATGACGCGGGACA
AGCCGTTTTACGTTTGGAACTGACAGAACCGCAACGTTGAAGGAGCCACTGAGCCGCGGGTTTCTGGAGT
TTAATGAGCTAAGCACATACGTCAGAAACCATTATTGCGCGTTCAAAAGTCGCCTAAGGTCACTATCAGC
TAGCAAATATTTCTTGTCAAAAATGCTCCACTGACGTTCCATAAATTCCCCTCGGTATCCAATTAGAGTC
TCATATTCACTCTCAACTCGAATCCCCCCTATGAAAAAGCCTGAACTCACCGCGACGTCTGTCGAGAAGT
TTCTGATCGAAAAGTTCGACAGCGTCTCCGACCTGATGCAGCTCTCGGAGGGCGAAGAATCTCGTGCTTT
CAGCTTCGATGTAGGAGGGCGTGGATATGTCCTGCGGGTAAATAGCTGCGCCGATGGTTTCTACAAAGAT
CGTTATGTTTATCGGCACTTTGCATCGGCCGCGCTCCCGATTCCGGAAGTGCTTGACATTGGGGAGTTCA
GCGAGAGCCTGACCTATTGCATCTCCCGCCGTGCACAGGGTGTCACGTTGCAAGACCTGCCTGAAACCGA
ACTGCCCGCTGTTCTTCAGCCGGTCGCGGAGGCTATGGATGCTATCGCTGCGGCCGATCTTAGCCAGACG
AGCGGGTTCGGCCCATTCGGACCGCAAGGAATCGGTCAATACACTACATGGCGTGATTTCATATGCGCGA
TTGCTGATCCCCATGTGTATCACTGGCAAACTGTGATGGACGACACCGTCAGTGCGTCCGTCGCGCAGGC
TCTCGATGAGCTGATGCTTTGGGCGAGGACTGCCCCGAAGTCCGGCACTCGTGCACGCGGATTCGGCTCC
AACAATGTCCTGACGGACAATGGCCGCATAACAGCGGTCATTGACTGGAGCGAGGCGATGTTCGGGGATT
CCCAATACGAGGTCGCCAACATCTTCTTCTGGAGGCCGTGGTTGGCTTGTATGGAGCAGCAGACGCGCTA
CTTCGAGCGGAGGCATCCGGAGCTTGCAGGATCGCCAAGGCTCCGGGCGTATATGCTCCGCATTGGTCTT
GACCAACTCTATCAGAGCTTGGTTGACGGCAATTTCGATGATGCAGCTTGGGCGCAGGGTCGATGCGACG
CAATCGTCCGATCCGGAGCCGGGACTGTCGGGCGTACACAAATCGCCCGCAGAAGCGCGGCCGTCTGGAC
CGATGGCTGTGTAGAAGTACTCGCCGATAGTGGAAACCGACGCCCCAGCACTCGTCCGAGGGCAAAGGAA
TAGAGTAGATGCCGACCGAACAAGAGCTGATTTCGAGAACGCCTCAGCCAGCAACTCGCGCGAGCCTAGC
AAGGCAAATGCGAGAGAACGGCCTTACGCTTGGTGGCACAGTTCTCGTCCACAGTTCGCTAAGCTCGCTC
GGCTGGGTCGCGGGAGGGCCGGTCGCAGTGATTCAGGAATTAATTCCCTAGAGTCAAGCAGATCGTTCAA
ACATTTGGCAATAAAGTTTCTTAAGATTGAATCCTGTTGCCGGTCTTGCGATGATTATCATATAATTTCT
GTTGAATTACGTTAAGCATGTAATAATTAACATGTAATGCATGACGTTATTTATGAGATGGGTTTTTATG
ATTAGAGTCCCGCAATTATACATTTAATACGCGATAGAAAACAAAATATAGCGCGCAAACTAGGATAAAT
TATCGCGCGCGGTGTCATCTATGTTACTAGATCGACCGGCATGCAAGCTGATAATTCAATTCGGCGTTAA
TTCAGTACATTAAAAACGTCCGCAATGTGTTATTAAGTTGTCTAAGCGTCAATTTGTTTACACCACAATA
TATCCTGCCACCAGCCAGCCAACAGCTCCCCGACCGGCAGCTCGGCACAAAATCACCACTCGATACAGGC
AGCCCATCAGTCCGGGACGGCGTCAGCGGGAGAGCCGTTGTAAGGCGGCAGACTTTGCTCATGTTACCGA
TGCTATTCGGAAGAACGGCAACTAAGCTGCCGGGTTTGAAACACGGATGATCTCGCGGAGGGTAGCATGT
TGATTGTAACGATGACAGAGCGTTGCTGCCTGTGATCAATTCGGGCACGAACCCAGTGGACATAAGCCTC
GTTCGGTTCGTAAGCTGTAATGCAAGTAGCGTAACTGCCGTCACGCAACTGGTCCAGAACCTTGACCGAA
CGCAGCGGTGGTAACGGCGCAGTGGCGGTTTTCATGGCTTCTTGTTATGACATGTTTTTTTGGGGTACAG
TCTATGCCTCGGGCATCCAAGCAGCAAGCGCGTTACGCCGTGGGTCGATGTTTGATGTTATGGAGCAGCA
ACGATGTTACGCAGCAGGGCAGTCGCCCTAAAACAAAGTTAAACATCATGGGGGAAGCGGTGATCGCCGA
AGTATCGACTCAACTATCAGAGGTAGTTGGCGTCATCGAGCGCCATCTCGAACCGACGTTGCTGGCCGTA
CATTTGTACGGCTCCGCAGTGGATGGCGGCCTGAAGCCACACAGTGATATTGATTTGCTGGTTACGGTGA
CCGTAAGGCTTGATGAAACAACGCGGCGAGCTTTGATCAACGACCTTTTGGAAACTTCGGCTTCCCCTGG
AGAGAGCGAGATTCTCCGCGCTGTAGAAGTCACCATTGTTGTGCACGACGACATCATTCCGTGGCGTTAT

CCAGCTAAGCGCGAACTGCAATTTGGAGAATGGCAGCGCAATGACATTCTTGCAGGTATCTTCGAGCCAG
CCACGATCGACATTGATCTGGCTATCTTGCTGACAAAAGCAAGAGAACATAGCGTTGCCTTGGTAGGTCC
AGCGGCGGAGGAACTCTTTGATCCGGTTCCTGAACAGGATCTATTTGAGGCGCTAAATGAAACCTTAACG
CTATGGAACTCGCCGCCCGACTGGGCTGGCGATGAGCGAAATGTAGTGCTTACGTTGTCCCGCATTTGGT
ACAGCGCAGTAACCGGCAAAATCGCGCCGAAGGATGTCGCTGCCGACTGGGCAATGGAGCGCCTGCCGGC
CCAGTATCAGCCCGTCATACTTGAAGCTAGACAGGCTTATCTTGGACAAGAAGAAGATCGCTTGGCCTCG
CGCGCAGATCAGTTGGAAGAATTTGTCCACTACGTGAAAGGCGAGATCACCAAGGTAGTCGGCAAATAAT
GTCTAGCTAGAAATTCGTTCAAGCCGACGCCGCTTCGCCGGCGTTAACTCAAGCGATTAGATGCACTAAG
CACATAATTGCTCACAGCCAAACTATCAGGTCAAGTCTGCTTTTATTATTTTTAAGCGTGCATAATAAGC
CCTACACAAATTGGGAGATATATCATGCATGACCAAAATCCCTTAACGTGAGTTTTCGTTCCACTGAGCG
TCAGACCCCGTAGAAAAGATCAAAGGATCTTCTTGAGATCCTTTTTTTCTGCGCGTAATCTGCTGCTTGC
AAACAAAAAAACCACCGCTACCAGCGGTGGTTTGTTTGCCGGATCAAGAGCTACCAACTCTTTTTCCGAA
GGTAACTGGCTTCAGCAGAGCGCAGATACCAAATACTGTCCTTCTAGTGTAGCCGTAGTTAGGCCACCAC
TTCAAGAACTCTGTAGCACCGCCTACATACCTCGCTCTGCTAATCCTGTTACCAGTGGCTGCTGCCAGTG
GCGATAAGTCGTGTCTTACCGGGTTGGACTCAAGACGATAGTTACCGGATAAGGCGCAGCGGTCGGGCTG
AACGGGGGGTTCGTGCACACAGCCCAGCTTGGAGCGAACGACCTACACCGAACTGAGATACCTACAGCGT
GAGCTATGAGAAAGCGCCACGCTTCCCGAAGGGAGAAAGGCGGACAGGTATCCGGTAAGCGGCAGGGTCG
GAACAGGAGAGCGCACGAGGGAGCTTCCAGGGGGAAACGCCTGGTATCTTTATAGTCCTGTCGGGTTTCG
CCACCTCTGACTTGAGCGTCGATTTTTGTGATGCTCGTCAGGGGGGCGGAGCCTATGGAAAAACGCCAGC
AACGCGGCCTTTTTACGGTTCCTGGCCTTTTGCTGGCCTTTTGCTCACATGTTCTTTCCTGCGTTATCCC
CTGATTCTGTGGATAACCGTATTACCGCCTTTGAGTGAGCTGATACCGCTCGCCGCAGCCGAACGACCGA
GCGCAGCGAGTCAGTGAGCGAGGAAGCGGAAGAGCGCCTGATGCGGTATTTTCTCCTTACGCATCTGTGC
GGTATTTCACACCGCATATGGTGCACTCTCAGTACAATCTGCTCTGATGCCGCATAGTTAAGCCAGTATA
CACTCCGCTATCGCTACGTGACTGGGTCATGGCTGCGCCCCGACACCCGCCAACACCCGCTGACGCGCCC
TGACGGGCTTGTCTGCTCCCGGCATCCGCTTACAGACAAGCTGTGACCGTCTCCGGGAGCTGCATGTGTC
AGAGGTTTTCACCGTCATCACCGAAACGCGCGAGGCAGGGTGCCTTGATGTGGGCGCCGGCGGTCGAGTG
GCGACGGCGCGGCTTGTCCGCGCCCTGGTAGATTGCCTGGCCGTAGGCCAGCCATTTTTGAGCGGCCAGC
GGCCGCGATAGGCCGACGCGAAGCGGCGGGGCGTAGGGAGCGCAGCGACCGAAGGGTAGGCGCTTTTTGC
AGCTCTTCGGCTGTGCGCTGGCCAGACAGTTATGCACAGGCCAGGCGGGTTTTAAGAGTTTTAATAAGTT
TTAAAGAGTTTTAGGCGGAAAAATCGCCTTTTTTCTCTTTTATATCAGTCACTTACATGTGTGACCGGTT
CCCAATGTACGGCTTTGGGTTCCCAATGTACGGGTTCCGGTTCCCAATGTACGGCTTTGGGTTCCCAATG
TACGTGCTATCCACAGGAAAGAGACCTTTTCGACCTTTTTCCCCTGCTAGGGCAATTTGCCCTAGCATCT
GCTCCGTACATTAGGAACCGGCGGATGCTTCGCCCTCGATCAGGTTGCGGTAGCGCATGACTAGGATCGG
GCCAGCCTGCCCCGCCTCCTCCTTCAAATCGTACTCCGGCAGGTCATTTGACCCGATCAGCTTGCGCACG
GTGAAACAGAACTTCTTGAACTCTCCGGCGCTGCCACTGCGTTCGTAGATCGTCTTGAACAACCATCTGG
CTTCTGCCTTGCCTGCGGCGCGGCGTGCCAGGCGGTAGAGAAAACGGCCGATGCCGGGATCGATCAAAAA
GTAATCGGGGTGAACCGTCAGCACGTCCGGGTTCTTGCCTTCTGTGATCTCGCGGTACATCCAATCAGCT
AGCTCGATCTCGATGTACTCCGGCCGCCCGGTTTCGCTCTTTACGATCTTGTAGCGGCTAATCAAGGCTT
CACCCTCGGATACCGTCACCAGGCGGCCGTTCTTGGCCTTCTTCGTACGCTGCATGGCAACGTGCGTGGT
GTTTAACCGAATGCAGGTTTCTACCAGGTCGTCTTTCTGCTTTCCGCCATCGGCTCGCCGGCAGAACTTG
AGTACGTCCGCAACGTGTGGACGGAACACGCGGCCGGGCTTGTCTCCCTTCCCTTCCCGGTATCGGTTCA
TGGATTCGGTTAGATGGGAAACCGCCATCAGTACCAGGTCGTAATCCCACACACTGGCCATGCCGGCCGG
CCCTGCGGAAACCTCTACGTGCCCGTCTGGAAGCTCGTAGCGGATCACCTCGCCAGCTCGTCGGTCACGC
TTCGACAGACGGAAAACGGCCACGTCCATGATGCTGCGACTATCGCGGGTGCCCACGTCATAGAGCATCG

GAACGAAAAAATCTGGTTGCTCGTCGCCCTTGGGCGGCTTCCTAATCGACGGCGCACCGGCTGCCGGCGG
TTGCCGGGATTCTTTGCGGATTCGATCAGCGGCCGCTTGCCACGATTCACCGGGGCGTGCTTCTGCCTCG
ATGCGTTGCCGCTGGGCGGCCTGCGCGGCCTTCAACTTCTCCACCAGGTCATCACCCAGCGCCGCGCCGA
TTTGTACCGGGCCGGATGGTTTGCGACCGTCACGCCGATTCCTCGGGCTTGGGGGTTCCAGTGCCATTGC
AGGGCCGGCAGACAACCCAGCCGCTTACGCCTGGCCAACCGCCCGTTCCTCCACACATGGGGCATTCCAC
GGCGTCGGTGCCTGGTTGTTCTTGATTTTCCATGCCGCCTCCTTTAGCCGCTAAAATTCATCTACTCATT
TATTCATTTGCTCATTTACTCTGGTAGCTGCGCGATGTATTCAGATAGCAGCTCGGTAATGGTCTTGCCT
TGGCGTACCGCGTACATCTTCAGCTTGGTGTGATCCTCCGCCGGCAACTGAAAGTTGACCCGCTTCATGG
CTGGCGTGTCTGCCAGGCTGGCCAACGTTGCAGCCTTGCTGCTGCGTGCGCTCGGACGGCCGGCACTTAG
CGTGTTTGTGCTTTTGCTCATTTTCTCTTTACCTCATTAACTCAAATGAGTTTTGATTTAATTTCAGCGG
CCAGCGCCTGGACCTCGCGGGCAGCGTCGCCCTCGGGTTCTGATTCAAGAACGGTTGTGCCGGCGGCGGC
AGTGCCTGGGTAGCTCACGCGCTGCGTGATACGGGACTCAAGAATGGGCAGCTCGTACCCGGCCAGCGCC
TCGGCAACCTCACCGCCGATGCGCGTGCCTTTGATCGCCCGCGACACGACAAAGGCCGCTTGTAGCCTTC
CATCCGTGACCTCAATGCGCTGCTTAACCAGCTCCACCAGGTCGGCGGTGGCCCATATGTCGTAAGGGCT
TGGCTGCACCGGAATCAGCACGAAGTCGGCTGCCTTGATCGCGGACACAGCCAAGTCCGCCGCCTGGGGC
GCTCCGTCGATCACTACGAAGTCGCGCCGGCCGATGGCCTTCACGTCGCGGTCAATCGTCGGGCGGTCGA
TGCCGACAACGGTTAGCGGTTGATCTTCCCGCACGGCCGCCCAATCGCGGGCACTGCCCTGGGGATCGGA
ATCGACTAACAGAACATCGGCCCCGGCGAGTTGCAGGGCGCGGGCTAGATGGGTTGCGATGGTCGTCTTG
CCTGACCCGCCTTTCTGGTTAAGTACAGCGATAACCTTCATGCGTTCCCCTTGCGTATTTGTTTATTTAC
TCATCGCATCATATACGCAGCGACCGCATGACGCAAGCTGTTTTACTCAAATACACATCACCTTTTTAGA
CGGCGGCGCTCGGTTTCTTCAGCGGCCAAGCTGGCCGGCCAGGCCGCCAGCTTGGCATCAGACAAACCGG
CCAGGATTTCATGCAGCCGCACGGTTGAGACGTGCGCGGGCGGCTCGAACACGTACCCGGCCGCGATCAT
CTCCGCCTCGATCTCTTCGGTAATGAAAAACGGTTCGTCCTGGCCGTCCTGGTGCGGTTTCATGCTTGTT
CCTCTTGGCGTTCATTCTCGGCGGCCGCCAGGGCGTCGGCCTCGGTCAATGCGTCCTCACGGAAGGCACC
GCGCCGCCTGGCCTCGGTGGGCGTCACTTCCTCGCTGCGCTCAAGTGCGCGGTACAGGGTCGAGCGATGC
ACGCCAAGCAGTGCAGCCGCCTCTTTCACGGTGCGGCCTTCCTGGTCGATCAGCTCGCGGGCGTGCGCGA
TCTGTGCCGGGGTGAGGGTAGGGCGGGGGCCAAACTTCACGCCTCGGGCCTTGGCGGCCTCGCGCCCGCT
CCGGGTGCGGTCGATGATTAGGGAACGCTCGAACTCGGCAATGCCGGCGAACACGGTCAACACCATGCGG
CCGGCCGGCGTGGTGGTGTCGGCCCACGGCTCTGCCAGGCTACGCAGGCCCGCGCCGGCCTCCTGGATGC
GCTCGGCAATGTCCAGTAGGTCGCGGGTGCTGCGGGCCAGGCGGTCTAGCCTGGTCACTGTCACAACGTC
GCCAGGGCGTAGGTGGTCAAGCATCCTGGCCAGCTCCGGGCGGTCGCGCCTGGTGCCGGTGATCTTCTCG
GAAAACAGCTTGGTGCAGCCGGCCGCGTGCAGTTCGGCCCGTTGGTTGGTCAAGTCCTGGTCGTCGGTGC
TGACGCGGGCATAGCCCAGCAGGCCAGCGGCGGCGCTCTTGTTCATGGCGTAATGTCTCCGGTTCTAGTC
GCAAGTATTCTACTTTATGCGACTAAAACACGCGACAAGAAAACGCCAGGAAAAGGGCAGGGCGGCAGCC
TGTCGCGTAACTTAGGACTTGTGCGACATGTCGTTTTCAGAAGACGGCTGCACTGAACGTCAGAAGCCGA
CTGCACTATAGCAGCGGAGGGGTTGGATCAAAGTACTTTGATCCCGAGGGGAACCCTGTGGTTGGCATGC
ACATACAAATGGACGAACGGATAAACCTTTTCACGCCCTTTTAAATATCCGTTATTCTAATAAACGCTCT
TTTCTCTTAGGTTTACCCGCCAATATATCCTGTCAAACACTGATAGTTTAAACTGAAGGCGGGAAACGAC
AATCTGATCCAAGCTCAAGCTAAGCTTGAGCTCTGCATGCCTGCAGGTCCCCAGATTAGCCTTTTCAATT
TCAGAAAGAATGCTAACCCACAGATGGTTAGAGAGGCTTACGCAGCAGGTCTCATCAAGACGATCTACCC
GAGCAATAATCTCCAGGAAATCAAATACCTTCCCAAGAAGGTTAAAGATGCAGTCAAAAGATTCAGGACT
AACTGCATCAAGAACACAGAGAAAGATATATTTCTCAAGATCAGAAGTACTATTCCAGTATGGACGATTC
AAGGCTTGCTTCACAAACCAAGGCAAGTAATAGAGATTGGAGTCTCTAAAAAGGTAGTTCCCACTGAATC
AAAGGCCATGGAGTCAAAGATTCAAATAGAGGACCTAACAGAACTCGCCGTAAAGACTGGCGAACAGTTC

ATACAGAGTCTCTTACGACTCAATGACAAGAAGAAAATCTTCGTCAACATGGTGGAGCACGACACACTTG
TCTACTCCAAAAATATCAAAGATACAGTCTCAGAAGACCAAAGGGCAATTGAGACTTTTCAACAAAGGGT
AATATCCGGAAACCTCCTCGGATTCCATTGCCCAGCTATCTGTCACTTTATTGTGAAGATAGTGGAAAAG
GAAGGTGGCTCCTACAAATGCCATCATTGCGATAAAGGAAAGGCCATCGTTGAAGATGCCTCTGCCGACA
GTGGTCCCAAAGATGGACCCCCACCCACGAGGAGCATCGTGGAAAAAGAAGACGTTCCAACCACGTCTTC
AAAGCAAGTGGATTGATGTGATATCTCCACTGACGTAAGGGATGACGCACAATCCCACTATCCTTCGCAA
GACCCTTCCTCTATATAAGGAAGTTCATTTCATTTGGAGAGAACACGGGGGACTCTAGAGGATCCAAGGA
GATATAAGCCGCCACCATGGATTACAAGGACGATGATGACAAGGACTACAAGGACGATGACGATAAGGAC
TATAAGGATGACGATGACAAGGATTACGCCGAGGATGGATGATACTAGCGGACAAACTTTCGTTTCTGTG
AACCTTTCTGACGAAAGCAACACGGCAAGCACTAAAGTTGAAAACGTAAAATCGGAAGCGGATCGCTTGG
AGTTTTTACGTAAAATGAATCCCTTTATTGTTGACGCTCTGGTGCGGAAAACCAATTATCAGGGTGCTCG
CTTTCGCGCAAGAATAATAGGAGTGTGCGTGGATTGTGGTAGAAAACACGACAAGGCGCTCAAGACTGAA
CGTAAGTGTAAGGTCAACAACACGCAATCTCAGAACGAGGTGGCGCATATGTTGATGCACGATCCCGTTA
AGTATTTGAACAAAAGAAAGGCTAGAGCCTTTTCTAACGCAGAGATGTTTGCGGCGGAACTGGTTTCGTA
CACCAAGGAAAGGCAATTGGCGGTCGATTTAGCCGCTGAAAGGGAGAAGACGAGACTGGCTCGTAGACAC
CCAATACGTTCTCCGGAAGAAACTCCGGAACATTATAAATTCGGTATGACTGCTAAGGCAATGTTACCGG
ACATCAACGCCGTAGACGTTGGTGATAACGAGGAAACTTCGTCGGAGTACCCAGTGAGTCTGAGTGTTTC
TGGCGGAGTTCTCCGTGAACACCACTTCATCTGACCTAATGATATCCCGCGGCCATGCTAGAGTCCGCAA
AAATCACCAGTCTCTCTCTACAAATCTATCTCTCTCTATTTTTCTCCAGAATAATGTGTGAGTAGTTCCC
AGATAAGGGAATTAGGGTTCTTATAGGGTTTCGCTCATGTGTTGAGCATATAAGAAACCCTTAGTATGTA
TTTGTATTTGTAAAATACTTCTATCAATAAAATTTCTAATTCCTAAAACCAAAATCCAGTGACCTGCAGG
CATGCGACGTCG

Sequence of pH7LIC3.1.1-p23M12 (35S- p23M12)

GGGTACCCTCGAATTATCATACATGAGAATTAAGGGAGTCACGTTATGACCCCCGCCGATGACGCGGGACA
AGCCGTTTTACGTTTGGAACTGACAGAACCGCAACGTTGAAGGAGCCACTGAGCCGCGGGTTTCTGGAGT
TTAATGAGCTAAGCACATACGTCAGAAACCATTATTGCGCGTTCAAAAGTCGCCTAAGGTCACTATCAGC
TAGCAAATATTTCTTGTCAAAAATGCTCCACTGACGTTCCATAAATTCCCCTCGGTATCCAATTAGAGTC
TCATATTCACTCTCAACTCGAATCCCCCCTATGAAAAAGCCTGAACTCACCGCGACGTCTGTCGAGAAGT
TTCTGATCGAAAAGTTCGACAGCGTCTCCGACCTGATGCAGCTCTCGGAGGGCGAAGAATCTCGTGCTTT
CAGCTTCGATGTAGGAGGGCGTGGATATGTCCTGCGGGTAAATAGCTGCGCCGATGGTTTCTACAAAGAT
CGTTATGTTTATCGGCACTTTGCATCGGCCGCGCTCCCGATTCCGGAAGTGCTTGACATTGGGGAGTTCA
GCGAGAGCCTGACCTATTGCATCTCCCGCCGTGCACAGGGTGTCACGTTGCAAGACCTGCCTGAAACCGA
ACTGCCCGCTGTTCTTCAGCCGGTCGCGGAGGCTATGGATGCTATCGCTGCGGCCGATCTTAGCCAGACG
AGCGGGTTCGGCCCATTCGGACCGCAAGGAATCGGTCAATACACTACATGGCGTGATTTCATATGCGCGA
TTGCTGATCCCCATGTGTATCACTGGCAAACTGTGATGGACGACACCGTCAGTGCGTCCGTCGCGCAGGC
TCTCGATGAGCTGATGCTTTGGGCCGAGGACTGCCCCGAAGTCCGGCACCTCGTGCACGCGGATTCGGCTCC
AACAATGTCCTGACGGACAATGGCCGCATAACAGCGGTCATTGACTGGAGCGAGGCGATGTTCGGGGATT
CCCAATACGAGGTCGCCAACATCTTCTTCTGGAGGCCGTGGTTGGCTTGTATGGAGCAGCAGACGCGCTA
CTTCGAGCGGAGGCATCCGGAGCTTGCAGGATCGCCAAGGCTCCGGGCGTATATGCTCCGCATTGGTCTT
GACCAACTCTATCAGAGCTTGGTTGACGGCAATTTCGATGATGCAGCTTGGGCGCAGGGTCGATGCGACG
CAATCGTCCGATCCGGAGCCGGGACTGTCGGGCGTACACAAATCGCCCGCAGAAGCGCGGCCGTCTGGAC
CGATGGCTGTGTAGAAGTACTCGCCGATAGTGGAAACCGACGCCCCAGCACTCGTCCGAGGGCAAAGGAA
TAGAGTAGATGCCGACCGAACAAGAGCTGATTTCGAGAACGCCTCAGCCAGCAACTCGCGCGAGCCTAGC

AAGGCAAATGCGAGAGAACGGCCTTACGCTTGGTGGCACAGTTCTCGTCCACAGTTCGCTAAGCTCGCTC
GGCTGGGTCGCGGGAGGGCCGGTCGCAGTGATTCAGGAATTAATTCCCTAGAGTCAAGCAGATCGTTCAA
ACATTTGGCAATAAAGTTTCTTAAGATTGAATCCTGTTGCCGGTCTTGCGATGATTATCATATAATTTCT
GTTGAATTACGTTAAGCATGTAATAATTAACATGTAATGCATGACGTTATTTATGAGATGGGTTTTTATG
ATTAGAGTCCCGCAATTATACATTTAATACGCGATAGAAAACAAAATATAGCGCGCAAACTAGGATAAAT
TATCGCGCGCGGTGTCATCTATGTTACTAGATCGACCGGCATGCAAGCTGATAATTCAATTCGGCGTTAA
TTCAGTACATTAAAAACGTCCGCAATGTGTTATTAAGTTGTCTAAGCGTCAATTTGTTTACACCACAATA
TATCCTGCCACCAGCCAGCCAACAGCTCCCCGACCGGCAGCTCGGCACAAAATCACCACTCGATACAGGC
AGCCCATCAGTCCGGGACGGCGTCAGCGGGAGAGCCGTTGTAAGGCGGCAGACTTTGCTCATGTTACCGA
TGCTATTCGGAAGAACGGCAACTAAGCTGCCGGGTTTGAAACACGGATGATCTCGCGGAGGGTAGCATGT
TGATTGTAACGATGACAGAGCGTTGCTGCCTGTGATCAATTCGGGCACGAACCCAGTGGACATAAGCCTC
GTTCGGTTCGTAAGCTGTAATGCAAGTAGCGTAACTGCCGTCACGCAACTGGTCCAGAACCTTGACCGAA
CGCAGCGGTGGTAACGGCGCAGTGGCGGTTTTCATGGCTTCTTGTTATGACATGTTTTTTTGGGGTACAG
TCTATGCCTCGGGCATCCAAGCAGCAAGCGCGTTACGCCGTGGGTCGATGTTTGATGTTATGGAGCAGCA
ACGATGTTACGCAGCAGGGCAGTCGCCCTAAAACAAAGTTAAACATCATGGGGGAAGCGGTGATCGCCGA
AGTATCGACTCAACTATCAGAGGTAGTTGGCGTCATCGAGCGCCATCTCGAACCGACGTTGCTGGCCGTA
CATTTGTACGGCTCCGCAGTGGATGGCGGCCTGAAGCCACACAGTGATATTGATTTGCTGGTTACGGTGA
CCGTAAGGCTTGATGAAACAACGCGGCGAGCTTTGATCAACGACCTTTTGGAAACTTCGGCTTCCCCTGG
AGAGAGCGAGATTCTCCGCGCTGTAGAAGTCACCATTGTTGTGCACGACGACATCATTCCGTGGCGTTAT
CCAGCTAAGCGCGAACTGCAATTTGGAGAATGGCAGCGCAATGACATTCTTGCAGGTATCTTCGAGCCAG
CCACGATCGACATTGATCTGGCTATCTTGCTGACAAAAGCAAGAGAACATAGCGTTGCCTTGGTAGGTCC
AGCGGCGGAGGAACTCTTTGATCCGGTTCCTGAACAGGATCTATTTGAGGCGCTAAATGAAACCTTAACG
CTATGGAACTCGCCGCCCGACTGGGCTGGCGATGAGCGAAATGTAGTGCTTACGTTGTCCCGCATTTGGT
ACAGCGCAGTAACCGGCAAAATCGCGCCGAAGGATGTCGCTGCCGACTGGGCAATGGAGCGCCTGCCGGC
CCAGTATCAGCCCGTCATACTTGAAGCTAGACAGGCTTATCTTGGACAAGAAGAAGATCGCTTGGCCTCG
CGCGCAGATCAGTTGGAAGAATTTGTCCACTACGTGAAAGGCGAGATCACCAAGGTAGTCGGCAAATAAT
GTCTAGCTAGAAATTCGTTCAAGCCGACGCCGCTTCGCCGGCGTTAACTCAAGCGATTAGATGCACTAAG
CACATAATTGCTCACAGCCAAACTATCAGGTCAAGTCTGCTTTTATTATTTTTAAGCGTGCATAATAAGC
CCTACACAAATTGGGAGATATATCATGCATGACCAAAATCCCTTAACGTGAGTTTTCGTTCCACTGAGCG
TCAGACCCCGTAGAAAAGATCAAAGGATCTTCTTGAGATCCTTTTTTTCTGCGCGTAATCTGCTGCTTGC
AAACAAAAAAACCACCGCTACCAGCGGTGGTTTGTTTGCCGGATCAAGAGCTACCAACTCTTTTTCCGAA
GGTAACTGGCTTCAGCAGAGCGCAGATACCAAATACTGTCCTTCTAGTGTAGCCGTAGTTAGGCCACCAC
TTCAAGAACTCTGTAGCACCGCCTACATACCTCGCTCTGCTAATCCTGTTACCAGTGGCTGCTGCCAGTG
GCGATAAGTCGTGTCTTACCGGGTTGGACTCAAGACGATAGTTACCGGATAAGGCGCAGCGGTCGGGCTG
AACGGGGGGTTCGTGCACACAGCCCAGCTTGGAGCGAACGACCTACACCGAACTGAGATACCTACAGCGT
GAGCTATGAGAAAGCGCCACGCTTCCCGAAGGGAGAAAGGCGGACAGGTATCCGGTAAGCGGCAGGGTCG
GAACAGGAGAGCGCACGAGGGAGCTTCCAGGGGGAAACGCCTGGTATCTTTATAGTCCTGTCGGGTTTCG
CCACCTCTGACTTGAGCGTCGATTTTTGTGATGCTCGTCAGGGGGGCGGAGCCTATGGAAAAACGCCAGC
AACGCGGCCTTTTTACGGTTCCTGGCCTTTTGCTGGCCTTTTGCTCACATGTTCTTTCCTGCGTTATCCC
CTGATTCTGTGGATAACCGTATTACCGCCTTTGAGTGAGCTGATACCGCTCGCCGCAGCCGAACGACCGA
GCGCAGCGAGTCAGTGAGCGAGGAAGCGGAAGAGCGCCTGATGCGGTATTTTCTCCTTACGCATCTGTGC
GGTATTTCACACCGCATATGGTGCACTCTCAGTACAATCTGCTCTGATGCCGCATAGTTAAGCCAGTATA
CACTCCGCTATCGCTACGTGACTGGGTCATGGCTGCGCCCCGACACCCGCCAACACCCGCTGACGCGCCC
TGACGGGCTTGTCTGCTCCCGGCATCCGCTTACAGACAAGCTGTGACCGTCTCCGGGAGCTGCATGTGTC

AGAGGTTTTCACCGTCATCACCGAAACGCGCGAGGCAGGGTGCCTTGATGTGGGCGCCGGCGGTCGAGTG
GCGACGGCGCGGCTTGTCCGCGCCCTGGTAGATTGCCTGGCCGTAGGCCAGCCATTTTTGAGCGGCCAGC
GGCCGCGATAGGCCGACGCGAAGCGGCGGGGCGTAGGGAGCGCAGCGACCGAAGGGTAGGCGCTTTTTGC
AGCTCTTCGGCTGTGCGCTGGCCAGACAGTTATGCACAGGCCAGGCGGGTTTTAAGAGTTTTAATAAGTT
TTAAAGAGTTTTAGGCGGAAAAATCGCCTTTTTTCTCTTTTATATCAGTCACTTACATGTGTGACCGGTT
CCCAATGTACGGCTTTGGGTTCCCAATGTACGGGTTCCGGTTCCCAATGTACGGCTTTGGGTTCCCAATG
TACGTGCTATCCACAGGAAAGAGACCTTTTCGACCTTTTTCCCCTGCTAGGGCAATTTGCCCTAGCATCT
GCTCCGTACATTAGGAACCGGCGGATGCTTCGCCCTCGATCAGGTTGCGGTAGCGCATGACTAGGATCGG
GCCAGCCTGCCCCGCCTCCTCCTTCAAATCGTACTCCGGCAGGTCATTTGACCCGATCAGCTTGCGCACG
GTGAAACAGAACTTCTTGAACTCTCCGGCGCTGCCACTGCGTTCGTAGATCGTCTTGAACAACCATCTGG
CTTCTGCCTTGCCTGCGGCGCGGCGTGCCAGGCGGTAGAGAAAACGGCCGATGCCGGGATCGATCAAAAA
GTAATCGGGGTGAACCGTCAGCACGTCCGGGTTCTTGCCTTCTGTGATCTCGCGGTACATCCAATCAGCT
AGCTCGATCTCGATGTACTCCGGCCGCCCGGTTTCGCTCTTTACGATCTTGTAGCGGCTAATCAAGGCTT
CACCCTCGGATACCGTCACCAGGCGGCCGTTCTTGGCCTTCTTCGTACGCTGCATGGCAACGTGCGTGGT
GTTTAACCGAATGCAGGTTTCTACCAGGTCGTCTTTCTGCTTTCCGCCATCGGCTCGCCGGCAGAACTTG
AGTACGTCCGCAACGTGTGGACGGAACACGCGGCCGGGCTTGTCTCCCTTCCCTTCCCGGTATCGGTTCA
TGGATTCGGTTAGATGGGAAACCGCCATCAGTACCAGGTCGTAATCCCACACACTGGCCATGCCGGCCGG
CCCTGCGGAAACCTCTACGTGCCCGTCTGGAAGCTCGTAGCGGATCACCTCGCCAGCTCGTCGGTCACGC
TTCGACAGACGGAAAACGGCCACGTCCATGATGCTGCGACTATCGCGGGTGCCCACGTCATAGAGCATCG
GAACGAAAAAATCTGGTTGCTCGTCGCCCTTGGGCGGCTTCCTAATCGACGGCGCACCGGCTGCCGGCGG
TTGCCGGGATTCTTTGCGGATTCGATCAGCGGCCGCTTGCCACGATTCACCGGGGCGTGCTTCTGCCTCG
ATGCGTTGCCGCTGGGCGGCCTGCGCGGCCTTCAACTTCTCCACCAGGTCATCACCCAGCGCCGCGCCGA
TTTGTACCGGGCCGGATGGTTTGCGACCGTCACGCCGATTCCTCGGGCTTGGGGGTTCCAGTGCCATTGC
AGGGCCGGCAGACAACCCAGCCGCTTACGCCTGGCCAACCGCCCGTTCCTCCACACATGGGGCATTCCAC
GGCGTCGGTGCCTGGTTGTTCTTGATTTTCCATGCCGCCTCCTTTAGCCGCTAAAATTCATCTACTCATT
TATTCATTTGCTCATTTACTCTGGTAGCTGCGCGATGTATTCAGATAGCAGCTCGGTAATGGTCTTGCCT
TGGCGTACCGCGTACATCTTCAGCTTGGTGTGATCCTCCGCCGGCAACTGAAAGTTGACCCGCTTCATGG
CTGGCGTGTCTGCCAGGCTGGCCAACGTTGCAGCCTTGCTGCTGCGTGCGCTCGGACGGCCGGCACTTAG
CGTGTTTGTGCTTTTGCTCATTTTCTCTTTACCTCATTAACTCAAATGAGTTTTGATTTAATTTCAGCGG
CCAGCGCCTGGACCTCGCGGGCAGCGTCGCCCTCGGGTTCTGATTCAAGAACGGTTGTGCCGGCGGCGGC
AGTGCCTGGGTAGCTCACGCGCTGCGTGATACGGGACTCAAGAATGGGCAGCTCGTACCCGGCCAGCGCC
TCGGCAACCTCACCGCCGATGCGCGTGCCTTTGATCGCCCGCGACACGACAAAGGCCGCTTGTAGCCTTC
CATCCGTGACCTCAATGCGCTGCTTAACCAGCTCCACCAGGTCGGCGGTGGCCCATATGTCGTAAGGGCT
TGGCTGCACCGGAATCAGCACGAAGTCGGCTGCCTTGATCGCGGACACAGCCAAGTCCGCCGCCTGGGGC
GCTCCGTCGATCACTACGAAGTCGCGCCGGCCGATGGCCTTCACGTCGCGGTCAATCGTCGGGCGGTCGA
TGCCGACAACGGTTAGCGGTTGATCTTCCCGCACGGCCGCCCAATCGCGGGCACTGCCCTGGGGATCGGA
ATCGACTAACAGAACATCGGCCCCGGCGAGTTGCAGGGCGCGGGCTAGATGGGTTGCGATGGTCGTCTTG
CCTGACCCGCCTTTCTGGTTAAGTACAGCGATAACCTTCATGCGTTCCCCTTGCGTATTTGTTTATTTAC
TCATCGCATCATATACGCAGCGACCGCATGACGCAAGCTGTTTTACTCAAATACACATCACCTTTTTAGA
CGGCGGCGCTCGGTTTCTTCAGCGGCCAAGCTGGCCGGCCAGGCCGCCAGCTTGGCATCAGACAAACCGG
CCAGGATTTCATGCAGCCGCACGGTTGAGACGTGCGCGGGCGGCTCGAACACGTACCCGGCCGCGATCAT
CTCCGCCTCGATCTCTTCGGTAATGAAAAACGGTTCGTCCTGGCCGTCCTGGTGCGGTTTCATGCTTGTT
CCTCTTGGCGTTCATTCTCGGCGGCCGCCAGGGCGTCGGCCTCGGTCAATGCGTCCTCACGGAAGGCACC
GCGCCGCCTGGCCTCGGTGGGCGTCACTTCCTCGCTGCGCTCAAGTGCGCGGTACAGGGTCGAGCGATGC

ACGCCAAGCAGTGCAGCCGCCTCTTTCACGGTGCGGCCTTCCTGGTCGATCAGCTCGCGGGCGTGCGCGA
TCTGTGCCGGGGTGAGGGTAGGGCGGGGGCCAAACTTCACGCCTCGGGCCTTGGCGGCCTCGCGCCCGCT
CCGGGTGCGGTCGATGATTAGGGAACGCTCGAACTCGGCAATGCCGGCGAACACGGTCAACACCATGCGG
CCGGCCGGCGTGGTGGTGTCGGCCCACGGCTCTGCCAGGCTACGCAGGCCCGCGCCGGCCTCCTGGATGC
GCTCGGCAATGTCCAGTAGGTCGCGGGTGCTGCGGGCCAGGCGGTCTAGCCTGGTCACTGTCACAACGTC
GCCAGGGCGTAGGTGGTCAAGCATCCTGGCCAGCTCCGGGCGGTCGCGCCTGGTGCCGGTGATCTTCTCG
GAAAACAGCTTGGTGCAGCCGGCCGCGTGCAGTTCGGCCCGTTGGTTGGTCAAGTCCTGGTCGTCGGTGC
TGACGCGGGCATAGCCCAGCAGGCCAGCGGCGGCGCTCTTGTTCATGGCGTAATGTCTCCGGTTCTAGTC
GCAAGTATTCTACTTTATGCGACTAAAACACGCGACAAGAAAACGCCAGGAAAAGGGCAGGGCGGCAGCC
TGTCGCGTAACTTAGGACTTGTGCGACATGTCGTTTTCAGAAGACGGCTGCACTGAACGTCAGAAGCCGA
CTGCACTATAGCAGCGGAGGGGTTGGATCAAAGTACTTTGATCCCGAGGGGAACCCTGTGGTTGGCATGC
ACATACAAATGGACGAACGGATAAACCTTTTCACGCCCTTTTAAATATCCGTTATTCTAATAAACGCTCT
TTTCTCTTAGGTTTACCCGCCAATATATCCTGTCAAACACTGATAGTTTAAACTGAAGGCGGGAAACGAC
AATCTGATCCAAGCTCAAGCTAAGCTTGAGCTCTGCATGCCTGCAGGTCCCCAGATTAGCCTTTTCAATT
TCAGAAAGAATGCTAACCCACAGATGGTTAGAGAGGCTTACGCAGCAGGTCTCATCAAGACGATCTACCC
GAGCAATAATCTCCAGGAAATCAAATACCTTCCCAAGAAGGTTAAAGATGCAGTCAAAAGATTCAGGACT
AACTGCATCAAGAACACAGAGAAAGATATATTTCTCAAGATCAGAAGTACTATTCCAGTATGGACGATTC
AAGGCTTGCTTCACAAACCAAGGCAAGTAATAGAGATTGGAGTCTCTAAAAAGGTAGTTCCCACTGAATC
AAAGGCCATGGAGTCAAAGATTCAAATAGAGGACCTAACAGAACTCGCCGTAAAGACTGGCGAACAGTTC
ATACAGAGTCTCTTACGACTCAATGACAAGAAGAAAATCTTCGTCAACATGGTGGAGCACGACACACTTG
TCTACTCCAAAAATATCAAAGATACAGTCTCAGAAGACCAAAGGGCAATTGAGACTTTTCAACAAAGGGT
AATATCCGGAAACCTCCTCGGATTCCATTGCCCAGCTATCTGTCACTTTATTGTGAAGATAGTGGAAAAG
GAAGGTGGCTCCTACAAATGCCATCATTGCGATAAAGGAAAGGCCATCGTTGAAGATGCCTCTGCCGACA
GTGGTCCCAAAGATGGACCCCCACCCACGAGGAGCATCGTGGAAAAAGAAGACGTTCCAACCACGTCTTC
AAAGCAAGTGGATTGATGTGATATCTCCACTGACGTAAGGGATGACGCACAATCCCACTATCCTTCGCAA
GACCCTTCCTCTATATAAGGAAGTTCATTTCATTTGGAGAGAACACGGGGGACTCTAGAGGATCCAAGGA
GATATAAGCCGCCACCATGGATTACAAGGACGATGATGACAAGGACTACAAGGACGATGACGATAAGGAC
TATAAGGATGACGATGACAAGGATTACGCCGAGGATGGATGATACTAGCGGACAAACTTTCGTTTCTGTG
AACCTTTCTGACGAAAGCAACACGGCAAGCACTAAAGTTGAAAACGTAAAATCGGAAGCGGATCGCTTGG
AGTTTTTACGTAAAATGAATCCCTTTATTGTTGACGCTCTGGTGCGGAAAAACCAATTATCAGGGTGCTCG
CTTTCGCGCAAGAATAATAGGAGTGTGCGTGGATTGTGGTAGAAAACACGACAAGGCGCTCAAGACTGAA
CGTAAGTGTAAGGTCAACAACACGCAATCTCAGAACGAGGTGGCGCATATGTTGATGCACGATCCCGTTA
AGTATTTGAACAAAAGAAAGGCTAGAGCCTTTTCTAACGCAGAGATGTTTGCGATTGAAGCGGTTTCGTA
CACCAAGGAAAGGCAATTGGCGGTCGATTTAGCCGCTGAAAGGGAGAAGACGAGACTGGCTCGTAGACAC
CCAATACGTTCTCCGGAAGAAACTCCGGAACATTATAAATTCGGTATGACTGCTAAGGCAATGTTACCGG
ACATCAACGCCGTAGACGTTGGTGATAACGAGGAAACTTCGTCGGAGTACCCAGTGAGTCTGAGTGTTTC
TGGCGGAGTTCTCCGTGAACACCACTTCATCTGACCTAATGATATCCCGCGGCCATGCTAGAGTCCGCAA
AAATCACCAGTCTCTCTCTACAAATCTATCTCTCTCTATTTTTCTCCAGAATAATGTGTGAGTAGTTCCC
AGATAAGGGAATTAGGGTTCTTATAGGGTTTCGCTCATGTGTTGAGCATATAAGAAACCCTTAGTATGTA
TTTGTATTTGTAAAATACTTCTATCAATAAAATTTCTAATTCCTAAAACCAAAATCCAGTGACCTGCAGG
CATGCGACGTCG

Sequence of pH7LIC3.1.1-p23M13 (35S- p23M13)

GGGTACCCTCGAATTATCATACATGAGAATTAAGGGAGTCACGTTATGACCCCGCCGATGACGCGGGACA

AGCCGTTTTACGTTTGGAACTGACAGAACCGCAACGTTGAAGGAGCCACTGAGCCGCGGGTTTCTGGAGT
TTAATGAGCTAAGCACATACGTCAGAAACCATTATTGCGCGTTCAAAAGTCGCCTAAGGTCACTATCAGC
TAGCAAATATTTCTTGTCAAAAATGCTCCACTGACGTTCCATAAATTCCCCTCGGTATCCAATTAGAGTC
TCATATTCACTCTCAACTCGAATCCCCCCTATGAAAAAGCCTGAACTCACCGCGACGTCTGTCGAGAAGT
TTCTGATCGAAAAGTTCGACAGCGTCTCCGACCTGATGCAGCTCTCGGAGGGCGAAGAATCTCGTGCTTT
CAGCTTCGATGTAGGAGGGCGTGGATATGTCCTGCGGGTAAATAGCTGCGCCGATGGTTTCTACAAAGAT
CGTTATGTTTATCGGCACTTTGCATCGGCCGCGCTCCCGATTCCGGAAGTGCTTGACATTGGGGAGTTCA
GCGAGAGCCTGACCTATTGCATCTCCCGCCGTGCACAGGGTGTCACGTTGCAAGACCTGCCTGAAACCGA
ACTGCCCGCTGTTCTTCAGCCGGTCGCGGAGGCTATGGATGCTATCGCTGCGGCCGATCTTAGCCAGACG
AGCGGGTTCGGCCCATTCGGACCGCAAGGAATCGGTCAATACACTACATGGCGTGATTTCATATGCGCGA
TTGCTGATCCCCATGTGTATCACTGGCAAACTGTGATGGACGACACCGTCAGTGCGTCCGTCGCGCAGGC
TCTCGATGAGCTGATGCTTTGGGCGAGGACTGCCCCGAAGTCCGGCACTCGTGCACGCGGATTCGGCTCC
AACAATGTCCTGACGGACAATGGCCGCATAACAGCGGTCATTGACTGGAGCGAGGCGATGTTCGGGGATT
CCCAATACGAGGTCGCCAACATCTTCTTCTGGAGGCCGTGGTTGGCTTGTATGGAGCAGCAGACGCGCTA
CTTCGAGCGGAGGCATCCGGAGCTTGCAGGATCGCCAAGGCTCCGGGCGTATATGCTCCGCATTGGTCTT
GACCAACTCTATCAGAGCTTGGTTGACGGCAATTTCGATGATGCAGCTTGGGCGCAGGGTCGATGCGACG
CAATCGTCCGATCCGGAGCCGGGACTGTCGGGCGTACACAAATCGCCCGCAGAAGCGCGGCCGTCTGGAC
CGATGGCTGTGTAGAAGTACTCGCCGATAGTGGAAACCGACGCCCCAGCACTCGTCCGAGGGCAAAGGAA
TAGAGTAGATGCCGACCGAACAAGAGCTGATTTCGAGAACGCCTCAGCCAGCAACTCGCGCGAGCCTAGC
AAGGCAAATGCGAGAGAACGGCCTTACGCTTGGTGGCACAGTTCTCGTCCACAGTTCGCTAAGCTCGCTC
GGCTGGGTCGCGGGAGGGCCGGTCGCAGTGATTCAGGAATTAATTCCCTAGAGTCAAGCAGATCGTTCAA
ACATTTGGCAATAAAGTTTCTTAAGATTGAATCCTGTTGCCGGTCTTGCGATGATTATCATATAATTTCT
GTTGAATTACGTTAAGCATGTAATAATTAACATGTAATGCATGACGTTATTTATGAGATGGGTTTTTATG
ATTAGAGTCCCGCAATTATACATTTAATACGCGATAGAAAACAAAATATAGCGCGCAAACTAGGATAAAT
TATCGCGCGCGGTGTCATCTATGTTACTAGATCGACCGGCATGCAAGCTGATAATTCAATTCGGCGTTAA
TTCAGTACATTAAAAACGTCCGCAATGTGTTATTAAGTTGTCTAAGCGTCAATTTGTTTACACCACAATA
TATCCTGCCACCAGCCAGCCAACAGCTCCCCGACCGGCAGCTCGGCACAAAATCACCACTCGATACAGGC
AGCCCATCAGTCCGGGACGGCGTCAGCGGGAGAGCCGTTGTAAGGCGGCAGACTTTGCTCATGTTACCGA
TGCTATTCGGAAGAACGGCAACTAAGCTGCCGGGTTTGAAACACGGATGATCTCGCGGAGGGTAGCATGT
TGATTGTAACGATGACAGAGCGTTGCTGCCTGTGATCAATTCGGGCACGAACCCAGTGGACATAAGCCTC
GTTCGGTTCGTAAGCTGTAATGCAAGTAGCGTAACTGCCGTCACGCAACTGGTCCAGAACCTTGACCGAA
CGCAGCGGTGGTAACGGCGCAGTGGCGGTTTTCATGGCTTCTTGTTATGACATGTTTTTTTGGGGTACAG
TCTATGCCTCGGGCATCCAAGCAGCAAGCGCGTTACGCCGTGGGTCGATGTTTGATGTTATGGAGCAGCA
ACGATGTTACGCAGCAGGGCAGTCGCCCTAAAACAAAGTTAAACATCATGGGGGAAGCGGTGATCGCCGA
AGTATCGACTCAACTATCAGAGGTAGTTGGCGTCATCGAGCGCCATCTCGAACCGACGTTGCTGGCCGTA
CATTTGTACGGCTCCGCAGTGGATGGCGGCCTGAAGCCACACAGTGATATTGATTTGCTGGTTACGGTGA
CCGTAAGGCTTGATGAAACAACGCGGCGAGCTTTGATCAACGACCTTTTGGAAACTTCGGCTTCCCCTGG
AGAGAGCGAGATTCTCCGCGCTGTAGAAGTCACCATTGTTGTGCACGACGACATCATTCCGTGGCGTTAT
CCAGCTAAGCGCGAACTGCAATTTGGAGAATGGCAGCGCAATGACATTCTTGCAGGTATCTTCGAGCCAG
CCACGATCGACATTGATCTGGCTATCTTGCTGACAAAAGCAAGAGAACATAGCGTTGCCTTGGTAGGTCC
AGCGGCGGAGGAACTCTTTGATCCGGTTCCTGAACAGGATCTATTTGAGGCGCTAAATGAAACCTTAACG
CTATGGAACTCGCCGCCCGACTGGGCTGGCGATGAGCGAAATGTAGTGCTTACGTTGTCCCGCATTTGGT
ACAGCGCAGTAACCGGCAAAATCGCGCCGAAGGATGTCGCTGCCGACTGGGCAATGGAGCGCCTGCCGGC
CCAGTATCAGCCCGTCATACTTGAAGCTAGACAGGCTTATCTTGGACAAGAAGAAGATCGCTTGGCCTCG

CGCGCAGATCAGTTGGAAGAATTTGTCCACTACGTGAAAGGCGAGATCACCAAGGTAGTCGGCAAATAAT
GTCTAGCTAGAAATTCGTTCAAGCCGACGCCGCTTCGCCGGCGTTAACTCAAGCGATTAGATGCACTAAG
CACATAATTGCTCACAGCCAAACTATCAGGTCAAGTCTGCTTTTATTATTTTTAAGCGTGCATAATAAGC
CCTACACAAATTGGGAGATATATCATGCATGACCAAAATCCCTTAACGTGAGTTTTCGTTCCACTGAGCG
TCAGACCCCGTAGAAAAGATCAAAGGATCTTCTTGAGATCCTTTTTTTCTGCGCGTAATCTGCTGCTTGC
AAACAAAAAAACCACCGCTACCAGCGGTGGTTTGTTTGCCGGATCAAGAGCTACCAACTCTTTTTCCGAA
GGTAACTGGCTTCAGCAGAGCGCAGATACCAAATACTGTCCTTCTAGTGTAGCCGTAGTTAGGCCACCAC
TTCAAGAACTCTGTAGCACCGCCTACATACCTCGCTCTGCTAATCCTGTTACCAGTGGCTGCTGCCAGTG
GCGATAAGTCGTGTCTTACCGGGTTGGACTCAAGACGATAGTTACCGGATAAGGCGCAGCGGTCGGGCTG
AACGGGGGGTTCGTGCACACAGCCCAGCTTGGAGCGAACGACCTACACCGAACTGAGATACCTACAGCGT
GAGCTATGAGAAAGCGCCACGCTTCCCGAAGGGAGAAAGGCGGACAGGTATCCGGTAAGCGGCAGGGTCG
GAACAGGAGAGCGCACGAGGGAGCTTCCAGGGGGAAACGCCTGGTATCTTTATAGTCCTGTCGGGTTTCG
CCACCTCTGACTTGAGCGTCGATTTTTGTGATGCTCGTCAGGGGGGCGGAGCCTATGGAAAAACGCCAGC
AACGCGGCCTTTTTACGGTTCCTGGCCTTTTGCTGGCCTTTTGCTCACATGTTCTTTCCTGCGTTATCCC
CTGATTCTGTGGATAACCGTATTACCGCCTTTGAGTGAGCTGATACCGCTCGCCGCAGCCGAACGACCGA
GCGCAGCGAGTCAGTGAGCGAGGAAGCGGAAGAGCGCCTGATGCGGTATTTTCTCCTTACGCATCTGTGC
GGTATTTCACACCGCATATGGTGCACTCTCAGTACAATCTGCTCTGATGCCGCATAGTTAAGCCAGTATA
CACTCCGCTATCGCTACGTGACTGGGTCATGGCTGCGCCCCGACACCCGCCAACACCCGCTGACGCGCCC
TGACGGGCTTGTCTGCTCCCGGCATCCGCTTACAGACAAGCTGTGACCGTCTCCGGGAGCTGCATGTGTC
AGAGGTTTTCACCGTCATCACCGAAACGCGCGAGGCAGGGTGCCTTGATGTGGGCGCCGGCGGTCGAGTG
GCGACGGCGCGGCTTGTCCGCGCCCTGGTAGATTGCCTGGCCGTAGGCCAGCCATTTTTGAGCGGCCAGC
GGCCGCGATAGGCCGACGCGAAGCGGCGGGGCGTAGGGAGCGCAGCGACCGAAGGGTAGGCGCTTTTTGC
AGCTCTTCGGCTGTGCGCTGGCCAGACAGTTATGCACAGGCCAGGCGGGTTTTAAGAGTTTTAATAAGTT
TTAAAGAGTTTTAGGCGGAAAAATCGCCTTTTTTCTCTTTTATATCAGTCACTTACATGTGTGACCGGTT
CCCAATGTACGGCTTTGGGTTCCCAATGTACGGGTTCCGGTTCCCAATGTACGGCTTTGGGTTCCCAATG
TACGTGCTATCCACAGGAAAGAGACCTTTTCGACCTTTTTCCCCTGCTAGGGCAATTTGCCCTAGCATCT
GCTCCGTACATTAGGAACCGGCGGATGCTTCGCCCTCGATCAGGTTGCGGTAGCGCATGACTAGGATCGG
GCCAGCCTGCCCCGCCTCCTCCTTCAAATCGTACTCCGGCAGGTCATTTGACCCGATCAGCTTGCGCACG
GTGAAACAGAACTTCTTGAACTCTCCGGCGCTGCCACTGCGTTCGTAGATCGTCTTGAACAACCATCTGG
CTTCTGCCTTGCCTGCGGCGCGGCGTGCCAGGCGGTAGAGAAAACGGCCGATGCCGGGATCGATCAAAAA
GTAATCGGGGTGAACCGTCAGCACGTCCGGGTTCTTGCCTTCTGTGATCTCGCGGTACATCCAATCAGCT
AGCTCGATCTCGATGTACTCCGGCCGCCCGGTTTCGCTCTTTACGATCTTGTAGCGGCTAATCAAGGCTT
CACCCTCGGATACCGTCACCAGGCGGCCGTTCTTGGCCTTCTTCGTACGCTGCATGGCAACGTGCGTGGT
GTTTAACCGAATGCAGGTTTCTACCAGGTCGTCTTTCTGCTTTCCGCCATCGGCTCGCCGGCAGAACTTG
AGTACGTCCGCAACGTGTGGACGGAACACGCGGCCGGGCTTGTCTCCCTTCCCTTCCCGGTATCGGTTCA
TGGATTCGGTTAGATGGGAAACCGCCATCAGTACCAGGTCGTAATCCCACACACTGGCCATGCCGGCCGG
CCCTGCGGAAACCTCTACGTGCCCGTCTGGAAGCTCGTAGCGGATCACCTCGCCAGCTCGTCGGTCACGC
TTCGACAGACGGAAAACGGCCACGTCCATGATGCTGCGACTATCGCGGGTGCCCACGTCATAGAGCATCG
GAACGAAAAAATCTGGTTGCTCGTCGCCCTTGGGCGGCTTCCTAATCGACGGCGCACCGGCTGCCGGCGG
TTGCCGGGATTCTTTGCGGATTCGATCAGCGGCCGCTTGCCACGATTCACCGGGGCGTGCTTCTGCCTCG
ATGCGTTGCCGCTGGGCGGCCTGCGCGGCCTTCAACTTCTCCACCAGGTCATCACCCAGCGCCGCGCCGA
TTTGTACCGGGCCGGATGGTTTGCGACCGTCACGCCGATTCCTCGGGCTTGGGGGTTCCAGTGCCATTGC
AGGGCCGGCAGACAACCCAGCCGCTTACGCCTGGCCAACCGCCCGTTCCTCCACACATGGGGCATTCCAC
GGCGTCGGTGCCTGGTTGTTCTTGATTTTCCATGCCGCCTCCTTTAGCCGCTAAAATTCATCTACTCATT

TATTCATTTGCTCATTTACTCTGGTAGCTGCGCGATGTATTCAGATAGCAGCTCGGTAATGGTCTTGCCT
TGGCGTACCGCGTACATCTTCAGCTTGGTGTGATCCTCCGCCGGCAACTGAAAGTTGACCCGCTTCATGG
CTGGCGTGTCTGCCAGGCTGGCCAACGTTGCAGCCTTGCTGCTGCGTGCGCTCGGACGGCCGGCACTTAG
CGTGTTTGTGCTTTTGCTCATTTTCTCTTTACCTCATTAACTCAAATGAGTTTTGATTTAATTTCAGCGG
CCAGCGCCTGGACCTCGCGGGCAGCGTCGCCCTCGGGTTCTGATTCAAGAACGGTTGTGCCGGCGGCGGC
AGTGCCTGGGTAGCTCACGCGCTGCGTGATACGGGACTCAAGAATGGGCAGCTCGTACCCGGCCAGCGCC
TCGGCAACCTCACCGCCGATGCGCGTGCCTTTGATCGCCCGCGACACGACAAAGGCCGCTTGTAGCCTTC
CATCCGTGACCTCAATGCGCTGCTTAACCAGCTCCACCAGGTCGGCGGTGGCCCATATGTCGTAAGGGCT
TGGCTGCACCGGAATCAGCACGAAGTCGGCTGCCTTGATCGCGGACACAGCCAAGTCCGCCGCCTGGGGC
GCTCCGTCGATCACTACGAAGTCGCGCCGGCCGATGGCCTTCACGTCGCGGTCAATCGTCGGGCGGTCGA
TGCCGACAACGGTTAGCGGTTGATCTTCCCGCACGGCCGCCCAATCGCGGGCACTGCCCTGGGGATCGGA
ATCGACTAACAGAACATCGGCCCCGGCGAGTTGCAGGGCGCGGGCTAGATGGGTTGCGATGGTCGTCTTG
CCTGACCCGCCTTTCTGGTTAAGTACAGCGATAACCTTCATGCGTTCCCCTTGCGTATTTGTTTATTTAC
TCATCGCATCATATACGCAGCGACCGCATGACGCAAGCTGTTTTACTCAAATACACATCACCTTTTTAGA
CGGCGGCGCTCGGTTTCTTCAGCGGCCAAGCTGGCCGGCCAGGCCGCCAGCTTGGCATCAGACAAACCGG
CCAGGATTTCATGCAGCCGCACGGTTGAGACGTGCGCGGGCGGCTCGAACACGTACCCGGCCGCGATCAT
CTCCGCCTCGATCTCTTCGGTAATGAAAAACGGTTCGTCCTGGCCGTCCTGGTGCGGTTTCATGCTTGTT
CCTCTTGGCGTTCATTCTCGGCGGCCGCCAGGGCGTCGGCCTCGGTCAATGCGTCCTCACGGAAGGCACC
GCGCCGCCTGGCCTCGGTGGGCGTCACTTCCTCGCTGCGCTCAAGTGCGCGGTACAGGGTCGAGCGATGC
ACGCCAAGCAGTGCAGCCGCCTCTTTCACGGTGCGGCCTTCCTGGTCGATCAGCTCGCGGGCGTGCGCGA
TCTGTGCCGGGGTGAGGGTAGGGCGGGGGCCAAACTTCACGCCTCGGGCCTTGGCGGCCTCGCGCCCGCT
CCGGGTGCGGTCGATGATTAGGGAACGCTCGAACTCGGCAATGCCGGCGAACACGGTCAACACCATGCGG
CCGGCCGGCGTGGTGGTGTCGGCCCACGGCTCTGCCAGGCTACGCAGGCCCGCGCCGGCCTCCTGGATGC
GCTCGGCAATGTCCAGTAGGTCGCGGGTGCTGCGGGCCAGGCGGTCTAGCCTGGTCACTGTCACAACGTC
GCCAGGGCGTAGGTGGTCAAGCATCCTGGCCAGCTCCGGGCGGTCGCGCCTGGTGCCGGTGATCTTCTCG
GAAAACAGCTTGGTGCAGCCGGCCGCGTGCAGTTCGGCCCGTTGGTTGGTCAAGTCCTGGTCGTCGGTGC
TGACGCGGGCATAGCCCAGCAGGCCAGCGGCGGCGCTCTTGTTCATGGCGTAATGTCTCCGGTTCTAGTC
GCAAGTATTCTACTTTATGCGACTAAAACACGCGACAAGAAAACGCCAGGAAAAGGGCAGGGCGGCAGCC
TGTCGCGTAACTTAGGACTTGTGCGACATGTCGTTTTCAGAAGACGGCTGCACTGAACGTCAGAAGCCGA
CTGCACTATAGCAGCGGAGGGGTTGGATCAAAGTACTTTGATCCCGAGGGGAACCCTGTGGTTGGCATGC
ACATACAAATGGACGAACGGATAAACCTTTTCACGCCCTTTTAAATATCCGTTATTCTAATAAACGCTCT
TTTCTCTTAGGTTTACCCGCCAATATATCCTGTCAAACACTGATAGTTTAAACTGAAGGCGGGAAACGAC
AATCTGATCCAAGCTCAAGCTAAGCTTGAGCTCTGCATGCCTGCAGGTCCCCAGATTAGCCTTTTCAATT
TCAGAAAGAATGCTAACCCACAGATGGTTAGAGAGGCTTACGCAGCAGGTCTCATCAAGACGATCTACCC
GAGCAATAATCTCCAGGAAATCAAATACCTTCCCAAGAAGGTTAAAGATGCAGTCAAAAGATTCAGGACT
AACTGCATCAAGAACACAGAGAAAGATATATTTCTCAAGATCAGAAGTACTATTCCAGTATGGACGATTC
AAGGCTTGCTTCACAAACCAAGGCAAGTAATAGAGATTGGAGTCTCTAAAAAGGTAGTTCCCACTGAATC
AAAGGCCATGGAGTCAAAGATTCAAATAGAGGACCTAACAGAACTCGCCGTAAAGACTGGCGAACAGTTC
ATACAGAGTCTCTTACGACTCAATGACAAGAAGAAAATCTTCGTCAACATGGTGGAGCACGACACACTTG
TCTACTCCAAAAATATCAAAGATACAGTCTCAGAAGACCAAAGGGCAATTGAGACTTTTCAACAAAGGGT
AATATCCGGAAACCTCCTCGGATTCCATTGCCCAGCTATCTGTCACTTTATTGTGAAGATAGTGGAAAAG
GAAGGTGGCTCCTACAAATGCCATCATTGCGATAAAGGAAAGGCCATCGTTGAAGATGCCTCTGCCGACA
GTGGTCCCAAAGATGGACCCCCACCCACGAGGAGCATCGTGGAAAAAGAAGACGTTCCAACCACGTCTTC
AAAGCAAGTGGATTGATGTGATATCTCCACTGACGTAAGGGATGACGCACAATCCCACTATCCTTCGCAA

GACCCTTCCTCTATATAAGGAAGTTCATTTCATTTGGAGAGAACACGGGGGACTCTAGAGGATCCAAGGA
GATATAAGCCGCCACCATGGATTACAAGGACGATGATGACAAGGACTACAAGGACGATGACGATAAGGAC
TATAAGGATGACGATGACAAGGATTACGCCGAGGATGGATGATACTAGCGGACAAACTTTCGTTTCTGTG
AACCTTTCTGACGAAAGCAACACGGCAAGCACTAAAGTTGAAAACGTAAAATCGGAAGCGGATCGCTTGG
AGTTTTTACGTAAAATGAATCCCTTTATTGTTGACGCTCTGGTGCGGAAAACCAATTATCAGGGTGCTCG
CTTTCGCGCAAGAATAATAGGAGTGTGCGTGGATTGTGGTAGAAAACACGACAAGGCGCTCAAGACTGAA
CGTAAGTGTAAGGTCAACAACACGCAATCTCAGAACGAGGTGGCGCATATGTTGATGCACGATCCCGTTA
AGTATTTGAACAAAAGAAAGGCTAGAGCCTTTTCTAACGCAGAGATGTTTGCGATTGAACTGGTTTCGTA
CGCAGCAGCAAGGCAATTGGCGGTCGATTTAGCCGCTGAAAGGGAGAAGACGAGACTGGCTCGTAGACAC
CCAATACGTTCTCCGGAAGAAACTCCGGAACATTATAAATTCGGTATGACTGCTAAGGCAATGTTACCGG
ACATCAACGCCGTAGACGTTGGTGATAACGAGGAAACTTCGTCGGAGTACCCAGTGAGTCTGAGTGTTTC
TGGCGGAGTTCTCCGTGAACACCACTTCATCTGACCTAATGATATCCCGCGGCCATGCTAGAGTCCGCAA
AAATCACCAGTCTCTCTCTACAAATCTATCTCTCTCTATTTTTCTCCAGAATAATGTGTGAGTAGTTCCC
AGATAAGGGAATTAGGGTTCTTATAGGGTTTCGCTCATGTGTTGAGCATATAAGAAACCCTTAGTATGTA
TTTGTATTTGTAAAATACTTCTATCAATAAAATTTCTAATTCCTAAAACCAAAATCCAGTGACCTGCAGG
CATGCGACGTCG

Sequence of pH7LIC3.1.1-p23M14 (35S- p23M14)

GGGTACCCTCGAATTATCATACATGAGAATTAAGGGAGTCACGTTATGACCCCGCCGATGACGCGGGACA
AGCCGTTTTACGTTTGGAACTGACAGAACCGCAACGTTGAAGGAGCCACTGAGCCGCGGGTTTCTGGAGT
TTAATGAGCTAAGCACATACGTCAGAAACCATTATTGCGCGTTCAAAAGTCGCCTAAGGTCACTATCAGC
TAGCAAATATTTCTTGTCAAAAATGCTCCACTGACGTTCCATAAATTCCCCTCGGTATCCAATTAGAGTC
TCATATTCACTCTCAACTCGAATCCCCCCTATGAAAAAGCCTGAACTCACCGCGACGTCTGTCGAGAAGT
TTCTGATCGAAAAGTTCGACAGCGTCTCCGACCTGATGCAGCTCTCGGAGGGCGAAGAATCTCGTGCTTT
CAGCTTCGATGTAGGAGGGCGTGGATATGTCCTGCGGGTAAATAGCTGCGCCGATGGTTTCTACAAAGAT
CGTTATGTTTATCGGCACTTTGCATCGGCCGCGCTCCCGATTCCGGAAGTGCTTGACATTGGGGAGTTCA
GCGAGAGCCTGACCTATTGCATCTCCCGCCGTGCACAGGGTGTCACGTTGCAAGACCTGCCTGAAACCGA
ACTGCCCGCTGTTCTTCAGCCGGTCGCGGAGGCTATGGATGCTATCGCTGCGGCCGATCTTAGCCAGACG
AGCGGGTTCGGCCCATTCGGACCGCAAGGAATCGGTCAATACACTACATGGCGTGATTTCATATGCGCGA
TTGCTGATCCCCATGTGTATCACTGGCAAACTGTGATGGACGACACCGTCAGTGCGTCCGTCGCGCAGGC
TCTCGATGAGCTGATGCTTTGGGCGAGGACTGCCCCGAAGTCCGGCACTCGTGCACGCGGATTCGGCTCC
AACAATGTCCTGACGGACAATGGCCGCATAACAGCGGTCATTGACTGGAGCGAGGCGATGTTCGGGGATT
CCCAATACGAGGTCGCCAACATCTTCTTCTGGAGGCCGTGGTTGGCTTGTATGGAGCAGCAGACGCGCTA
CTTCGAGCGGAGGCATCCGGAGCTTGCAGGATCGCCAAGGCTCCGGGCGTATATGCTCCGCATTGGTCTT
GACCAACTCTATCAGAGCTTGGTTGACGGCAATTTCGATGATGCAGCTTGGGCGCAGGGTCGATGCGACG
CAATCGTCCGATCCGGAGCCGGGACTGTCGGGCGTACACAAATCGCCCGCAGAAGCGCGGCCGTCTGGAC
CGATGGCTGTGTAGAAGTACTCGCCGATAGTGGAAACCGACGCCCCAGCACTCGTCCGAGGGCAAAGGAA
TAGAGTAGATGCCGACCGAACAAGAGCTGATTTCGAGAACGCCTCAGCCAGCAACTCGCGCGAGCCTAGC
AAGGCAAATGCGAGAGAACGGCCTTACGCTTGGTGGCACAGTTCTCGTCCACAGTTCGCTAAGCTCGCTC
GGCTGGGTCGCGGGAGGGCCGGTCGCAGTGATTCAGGAATTAATTCCCTAGAGTCAAGCAGATCGTTCAA
ACATTTGGCAATAAAGTTTCTTAAGATTGAATCCTGTTGCCGGTCTTGCGATGATTATCATATAATTTCT
GTTGAATTACGTTAAGCATGTAATAATTAACATGTAATGCATGACGTTATTTATGAGATGGGTTTTTATG
ATTAGAGTCCCGCAATTATACATTTAATACGCGATAGAAAACAAAATATAGCGCGCAAACTAGGATAAAT
TATCGCGCGCGGTGTCATCTATGTTACTAGATCGACCGGCATGCAAGCTGATAATTCAATTCGGCGTTAA

TTCAGTACATTAAAAACGTCCGCAATGTGTTATTAAGTTGTCTAAGCGTCAATTTGTTTACACCACAATA
TATCCTGCCACCAGCCAGCCAACAGCTCCCCGACCGGCAGCTCGGCACAAAATCACCACTCGATACAGGC
AGCCCATCAGTCCGGGACGGCGTCAGCGGGAGAGCCGTTGTAAGGCGGCAGACTTTGCTCATGTTACCGA
TGCTATTCGGAAGAACGGCAACTAAGCTGCCGGGTTTGAAACACGGATGATCTCGCGGAGGGTAGCATGT
TGATTGTAACGATGACAGAGCGTTGCTGCCTGTGATCAATTCGGGCACGAACCCAGTGGACATAAGCCTC
GTTCGGTTCGTAAGCTGTAATGCAAGTAGCGTAACTGCCGTCACGCAACTGGTCCAGAACCTTGACCGAA
CGCAGCGGTGGTAACGGCGCAGTGGCGGTTTTCATGGCTTCTTGTTATGACATGTTTTTTTGGGGTACAG
TCTATGCCTCGGGCATCCAAGCAGCAAGCGCGTTACGCCGTGGGTCGATGTTTGATGTTATGGAGCAGCA
ACGATGTTACGCAGCAGGGCAGTCGCCCTAAAACAAAGTTAAACATCATGGGGGAAGCGGTGATCGCCGA
AGTATCGACTCAACTATCAGAGGTAGTTGGCGTCATCGAGCGCCATCTCGAACCGACGTTGCTGGCCGTA
CATTTGTACGGCTCCGCAGTGGATGGCGGCCTGAAGCCACACAGTGATATTGATTTGCTGGTTACGGTGA
CCGTAAGGCTTGATGAAACAACGCGGCGAGCTTTGATCAACGACCTTTTGGAAACTTCGGCTTCCCCTGG
AGAGAGCGAGATTCTCCGCGCTGTAGAAGTCACCATTGTTGTGCACGACGACATCATTCCGTGGCGTTAT
CCAGCTAAGCGCGAACTGCAATTTGGAGAATGGCAGCGCAATGACATTCTTGCAGGTATCTTCGAGCCAG
CCACGATCGACATTGATCTGGCTATCTTGCTGACAAAAGCAAGAGAACATAGCGTTGCCTTGGTAGGTCC
AGCGGCGGAGGAACTCTTTGATCCGGTTCCTGAACAGGATCTATTTGAGGCGCTAAATGAAACCTTAACG
CTATGGAACTCGCCGCCCGACTGGGCTGGCGATGAGCGAAATGTAGTGCTTACGTTGTCCCGCATTTGGT
ACAGCGCAGTAACCGGCAAAATCGCGCCGAAGGATGTCGCTGCCGACTGGGCAATGGAGCGCCTGCCGGC
CCAGTATCAGCCCGTCATACTTGAAGCTAGACAGGCTTATCTTGGACAAGAAGAAGATCGCTTGGCCTCG
CGCGCAGATCAGTTGGAAGAATTTGTCCACTACGTGAAAGGCGAGATCACCAAGGTAGTCGGCAAATAAT
GTCTAGCTAGAAATTCGTTCAAGCCGACGCCGCTTCGCCGGCGTTAACTCAAGCGATTAGATGCACTAAG
CACATAATTGCTCACAGCCAAACTATCAGGTCAAGTCTGCTTTTATTATTTTTAAGCGTGCATAATAAGC
CCTACACAAATTGGGAGATATATCATGCATGACCAAAATCCCTTAACGTGAGTTTTCGTTCCACTGAGCG
TCAGACCCCGTAGAAAAGATCAAAGGATCTTCTTGAGATCCTTTTTTTCTGCGCGTAATCTGCTGCTTGC
AAACAAAAAAACCACCGCTACCAGCGGTGGTTTGTTTGCCGGATCAAGAGCTACCAACTCTTTTTCCGAA
GGTAACTGGCTTCAGCAGAGCGCAGATACCAAATACTGTCCTTCTAGTGTAGCCGTAGTTAGGCCACCAC
TTCAAGAACTCTGTAGCACCGCCTACATACCTCGCTCTGCTAATCCTGTTACCAGTGGCTGCTGCCAGTG
GCGATAAGTCGTGTCTTACCGGGTTGGACTCAAGACGATAGTTACCGGATAAGGCGCAGCGGTCGGGCTG
AACGGGGGGTTCGTGCACACAGCCCAGCTTGGAGCGAACGACCTACACCGAACTGAGATACCTACAGCGT
GAGCTATGAGAAAGCGCCACGCTTCCCGAAGGGAGAAAGGCGGACAGGTATCCGGTAAGCGGCAGGGTCG
GAACAGGAGAGCGCACGAGGGAGCTTCCAGGGGGAAACGCCTGGTATCTTTATAGTCCTGTCGGGTTTCG
CCACCTCTGACTTGAGCGTCGATTTTTGTGATGCTCGTCAGGGGGGCGGAGCCTATGGAAAAACGCCAGC
AACGCGGCCTTTTTACGGTTCCTGGCCTTTTGCTGGCCTTTTGCTCACATGTTCTTTCCTGCGTTATCCC
CTGATTCTGTGGATAACCGTATTACCGCCTTTGAGTGAGCTGATACCGCTCGCCGCAGCCGAACGACCGA
GCGCAGCGAGTCAGTGAGCGAGGAAGCGGAAGAGCGCCTGATGCGGTATTTTCTCCTTACGCATCTGTGC
GGTATTTCACACCGCATATGGTGCACTCTCAGTACAATCTGCTCTGATGCCGCATAGTTAAGCCAGTATA
CACTCCGCTATCGCTACGTGACTGGGTCATGGCTGCGCCCCGACACCCGCCAACACCCGCTGACGCGCCC
TGACGGGCTTGTCTGCTCCCGGCATCCGCTTACAGACAAGCTGTGACCGTCTCCGGGAGCTGCATGTGTC
AGAGGTTTTCACCGTCATCACCGAAACGCGCGAGGCAGGGTGCCTTGATGTGGGCGCCGGCGGTCGAGTG
GCGACGGCGCGGCTTGTCCGCGCCCTGGTAGATTGCCTGGCCGTAGGCCAGCCATTTTTGAGCGGCCAGC
GGCCGCGATAGGCCGACGCGAAGCGGCGGGGCGTAGGGAGCGCAGCGACCGAAGGGTAGGCGCTTTTTGC
AGCTCTTCGGCTGTGCGCTGGCCAGACAGTTATGCACAGGCCAGGCGGGTTTTAAGAGTTTTAATAAGTT
TTAAAGAGTTTTAGGCGGAAAAATCGCCTTTTTTCTCTTTTATATCAGTCACTTACATGTGTGACCGGTT
CCCAATGTACGGCTTTGGGTTCCCAATGTACGGGTTCCGGTTCCCAATGTACGGCTTTGGGTTCCCAATG

TACGTGCTATCCACAGGAAAGAGACCTTTTCGACCTTTTTCCCCTGCTAGGGCAATTTGCCCTAGCATCT
GCTCCGTACATTAGGAACCGGCGGATGCTTCGCCCTCGATCAGGTTGCGGTAGCGCATGACTAGGATCGG
GCCAGCCTGCCCCGCCTCCTCCTTCAAATCGTACTCCGGCAGGTCATTTGACCCGATCAGCTTGCGCACG
GTGAAACAGAACTTCTTGAACTCTCCGGCGCTGCCACTGCGTTCGTAGATCGTCTTGAACAACCATCTGG
CTTCTGCCTTGCCTGCGGCGCGGCGTGCCAGGCGGTAGAGAAAACGGCCGATGCCGGGATCGATCAAAAA
GTAATCGGGGTGAACCGTCAGCACGTCCGGGTTCTTGCCTTCTGTGATCTCGCGGTACATCCAATCAGCT
AGCTCGATCTCGATGTACTCCGGCCGCCCGGTTTCGCTCTTTACGATCTTGTAGCGGCTAATCAAGGCTT
CACCCTCGGATACCGTCACCAGGCGGCCGTTCTTGGCCTTCTTCGTACGCTGCATGGCAACGTGCGTGGT
GTTTAACCGAATGCAGGTTTCTACCAGGTCGTCTTTCTGCTTTCCGCCATCGGCTCGCCGGCAGAACTTG
AGTACGTCCGCAACGTGTGGACGGAACACGCGGCCGGGCTTGTCTCCCTTCCCTTCCCGGTATCGGTTCA
TGGATTCGGTTAGATGGGAAACCGCCATCAGTACCAGGTCGTAATCCCACACACTGGCCATGCCGGCCGG
CCCTGCGGAAACCTCTACGTGCCCGTCTGGAAGCTCGTAGCGGATCACCTCGCCAGCTCGTCGGTCACGC
TTCGACAGACGGAAAACGGCCACGTCCATGATGCTGCGACTATCGCGGGTGCCCACGTCATAGAGCATCG
GAACGAAAAAATCTGGTTGCTCGTCGCCCTTGGGCGGCTTCCTAATCGACGGCGCACCGGCTGCCGGCGG
TTGCCGGGATTCTTTGCGGATTCGATCAGCGGCCGCTTGCCACGATTCACCGGGGCGTGCTTCTGCCTCG
ATGCGTTGCCGCTGGGCGGCCTGCGCGGCCTTCAACTTCTCCACCAGGTCATCACCCAGCGCCGCGCCGA
TTTGTACCGGGCCGGATGGTTTGCGACCGTCACGCCGATTCCTCGGGCTTGGGGGTTCCAGTGCCATTGC
AGGGCCGGCAGACAACCCAGCCGCTTACGCCTGGCCAACCGCCCGTTCCTCCACACATGGGGCATTCCAC
GGCGTCGGTGCCTGGTTGTTCTTGATTTTCCATGCCGCCTCCTTTAGCCGCTAAAATTCATCTACTCATT
TATTCATTTGCTCATTTACTCTGGTAGCTGCGCGATGTATTCAGATAGCAGCTCGGTAATGGTCTTGCCT
TGGCGTACCGCGTACATCTTCAGCTTGGTGTGATCCTCCGCCGGCAACTGAAAGTTGACCCGCTTCATGG
CTGGCGTGTCTGCCAGGCTGGCCAACGTTGCAGCCTTGCTGCTGCGTGCGCTCGGACGGCCGGCACTTAG
CGTGTTTGTGCTTTTGCTCATTTTCTCTTTACCTCATTAACTCAAATGAGTTTTGATTTAATTTCAGCGG
CCAGCGCCTGGACCTCGCGGGCAGCGTCGCCCTCGGGTTCTGATTCAAGAACGGTTGTGCCGGCGGCGGC
AGTGCCTGGGTAGCTCACGCGCTGCGTGATACGGGACTCAAGAATGGGCAGCTCGTACCCGGCCAGCGCC
TCGGCAACCTCACCGCCGATGCGCGTGCCTTTGATCGCCCGCGACACGACAAAGGCCGCTTGTAGCCTTC
CATCCGTGACCTCAATGCGCTGCTTAACCAGCTCCACCAGGTCGGCGGTGGCCCATATGTCGTAAGGGCT
TGGCTGCACCGGAATCAGCACGAAGTCGGCTGCCTTGATCGCGGACACAGCCAAGTCCGCCGCCTGGGGC
GCTCCGTCGATCACTACGAAGTCGCGCCGGCCGATGGCCTTCACGTCGCGGTCAATCGTCGGGCGGTCGA
TGCCGACAACGGTTAGCGGTTGATCTTCCCGCACGGCCGCCCAATCGCGGGCACTGCCCTGGGGATCGGA
ATCGACTAACAGAACATCGGCCCCGGCGAGTTGCAGGGCGCGGGCTAGATGGGTTGCGATGGTCGTCTTG
CCTGACCCGCCTTTCTGGTTAAGTACAGCGATAACCTTCATGCGTTCCCCTTGCGTATTTGTTTATTTAC
TCATCGCATCATATACGCAGCGACCGCATGACGCAAGCTGTTTTACTCAAATACACATCACCTTTTTAGA
CGGCGGCGCTCGGTTTCTTCAGCGGCCAAGCTGGCCGGCCAGGCCGCCAGCTTGGCATCAGACAAACCGG
CCAGGATTTCATGCAGCCGCACGGTTGAGACGTGCGCGGGCGGCTCGAACACGTACCCGGCCGCGATCAT
CTCCGCCTCGATCTCTTCGGTAATGAAAAACGGTTCGTCCTGGCCGTCCTGGTGCGGTTTCATGCTTGTT
CCTCTTGGCGTTCATTCTCGGCGGCCGCCAGGGCGTCGGCCTCGGTCAATGCGTCCTCACGGAAGGCACC
GCGCCGCCTGGCCTCGGTGGGCGTCACTTCCTCGCTGCGCTCAAGTGCGCGGTACAGGGTCGAGCGATGC
ACGCCAAGCAGTGCAGCCGCCTCTTTCACGGTGCGGCCTTCCTGGTCGATCAGCTCGCGGGCGTGCGCGA
TCTGTGCCGGGGTGAGGGTAGGGCGGGGGCCAAACTTCACGCCTCGGGCCTTGGCGGCCTCGCGCCCGCT
CCGGGTGCGGTCGATGATTAGGGAACGCTCGAACTCGGCAATGCCGGCGAACACGGTCAACACCATGCGG
CCGGCCGGCGTGGTGGTGTCGGCCCACGGCTCTGCCAGGCTACGCAGGCCCGCGCCGGCCTCCTGGATGC
GCTCGGCAATGTCCAGTAGGTCGCGGGTGCTGCGGGCCAGGCGGTCTAGCCTGGTCACTGTCACAACGTC
GCCAGGGCGTAGGTGGTCAAGCATCCTGGCCAGCTCCGGGCGGTCGCGCCTGGTGCCGGTGATCTTCTCG

GAAAACAGCTTGGTGCAGCCGGCCGCGTGCAGTTCGGCCCGTTGGTTGGTCAAGTCCTGGTCGTCGGTGC
TGACGCGGGCATAGCCCAGCAGGCCAGCGGCGGCGCTCTTGTTCATGGCGTAATGTCTCCGGTTCTAGTC
GCAAGTATTCTACTTTATGCGACTAAAACACGCGACAAGAAAACGCCAGGAAAAGGGCAGGGCGGCAGCC
TGTCGCGTAACTTAGGACTTGTGCGACATGTCGTTTTCAGAAGACGGCTGCACTGAACGTCAGAAGCCGA
CTGCACTATAGCAGCGGAGGGGTTGGATCAAAGTACTTTGATCCCGAGGGGAACCCTGTGGTTGGCATGC
ACATACAAATGGACGAACGGATAAACCTTTTCACGCCCTTTTAAATATCCGTTATTCTAATAAACGCTCT
TTTCTCTTAGGTTTACCCGCCAATATATCCTGTCAAACACTGATAGTTTAAACTGAAGGCGGGAAACGAC
AATCTGATCCAAGCTCAAGCTAAGCTTGAGCTCTGCATGCCTGCAGGTCCCCAGATTAGCCTTTTCAATT
TCAGAAAGAATGCTAACCCACAGATGGTTAGAGAGGCTTACGCAGCAGGTCTCATCAAGACGATCTACCC
GAGCAATAATCTCCAGGAAATCAAATACCTTCCCAAGAAGGTTAAAGATGCAGTCAAAAGATTCAGGACT
AACTGCATCAAGAACACAGAGAAAGATATATTTCTCAAGATCAGAAGTACTATTCCAGTATGGACGATTC
AAGGCTTGCTTCACAAACCAAGGCAAGTAATAGAGATTGGAGTCTCTAAAAAGGTAGTTCCCACTGAATC
AAAGGCCATGGAGTCAAAGATTCAAATAGAGGACCTAACAGAACTCGCCGTAAAGACTGGCGAACAGTTC
ATACAGAGTCTCTTACGACTCAATGACAAGAAGAAAATCTTCGTCAACATGGTGGAGCACGACACACTTG
TCTACTCCAAAAATATCAAAGATACAGTCTCAGAAGACCAAAGGGCAATTGAGACTTTTCAACAAAGGGT
AATATCCGGAAACCTCCTCGGATTCCATTGCCCAGCTATCTGTCACTTTATTGTGAAGATAGTGGAAAAG
GAAGGTGGCTCCTACAAATGCCATCATTGCGATAAAGGAAAGGCCATCGTTGAAGATGCCTCTGCCGACA
GTGGTCCCAAAGATGGACCCCCACCCACGAGGAGCATCGTGGAAAAAGAAGACGTTCCAACCACGTCTTC
AAAGCAAGTGGATTGATGTGATATCTCCACTGACGTAAGGGATGACGCACAATCCCACTATCCTTCGCAA
GACCCTTCCTCTATATAAGGAAGTTCATTTCATTTGGAGAGAACACGGGGGACTCTAGAGGATCCAAGGA
GATATAAGCCGCCACCATGGATTACAAGGACGATGATGACAAGGACTACAAGGACGATGACGATAAGGAC
TATAAGGATGACGATGACAAGGATTACGCCGAGGATGGATGATACTAGCGGACAAACTTTCGTTTCTGTG
AACCTTTCTGACGAAAGCAACACGGCAAGCACTAAAGTTGAAAACGTAAAATCGGAAGCGGATCGCTTGG
AGTTTTTACGTAAAATGAATCCCTTTATTGTTGACGCTCTGGTGCGGAAAACCAATTATCAGGGTGCTCG
CTTTCGCGCAAGAATAATAGGAGTGTGCGTGGATTGTGGTAGAAAACACGACAAGGCGCTCAAGACTGAA
CGTAAGTGTAAGGTCAACAACACGCAATCTCAGAACGAGGTGGCGCATATGTTGATGCACGATCCCGTTA
AGTATTTGAACAAAAGAAAGGCTAGAGCCTTTTCTAACGCAGAGATGTTTGCGATTGAACTGGTTTCGTA
CACCAAGGAAAGGCAATTGGCGGTCGATTTAGCCGCTGAAGCGGCGAAGACGAGACTGGCTCGTAGACAC
CCAATACGTTCTCCGGAAGAAACTCCGGAACATTATAAATTCGGTATGACTGCTAAGGCAATGTTACCGG
ACATCAACGCCGTAGACGTTGGTGATAACGAGGAAACTTCGTCGGAGTACCCAGTGAGTCTGAGTGTTTC
TGGCGGAGTTCTCCGTGAACACCACTTCATCTGACCTAATGATATCCCGCGGCCATGCTAGAGTCCGCAA
AAATCACCAGTCTCTCTCTACAAATCTATCTCTCTCTATTTTTCTCCAGAATAATGTGTGAGTAGTTCCC
AGATAAGGGAATTAGGGTTCTTATAGGGTTTCGCTCATGTGTTGAGCATATAAGAAACCCTTAGTATGTA
TTTGTATTTGTAAAATACTTCTATCAATAAAATTTCTAATTCCTAAAACCAAAATCCAGTGACCTGCAGG
CATGCGACGTCG

Sequence of pH7LIC3.1.1-p23M15 (35S- p23M15)

GGGTACCCTCGAATTATCATACATGAGAATTAAGGGAGTCACGTTATGACCCCGCCGATGACGCGGGACA
AGCCGTTTTACGTTTGGAACTGACAGAACCGCAACGTTGAAGGAGCCACTGAGCCGCGGGTTTCTGGAGT
TTAATGAGCTAAGCACATACGTCAGAAACCATTATTGCGCGTTCAAAAGTCGCCTAAGGTCACTATCAGC
TAGCAAATATTTCTTGTCAAAAATGCTCCACTGACGTTCCATAAATTCCCCTCGGTATCCAATTAGAGTC
TCATATTCACTCTCAACTCGAATCCCCCCTATGAAAAAGCCTGAACTCACCGCGACGTCTGTCGAGAAGT
TTCTGATCGAAAAGTTCGACAGCGTCTCCGACCTGATGCAGCTCTCGGAGGGCGAAGAATCTCGTGCTTT
CAGCTTCGATGTAGGAGGGCGTGGATATGTCCTGCGGGTAAATAGCTGCGCCGATGGTTTCTACAAAGAT

CGTTATGTTTATCGGCACTTTGCATCGGCCGCGCTCCCGATTCCGGAAGTGCTTGACATTGGGGAGTTCA
GCGAGAGCCTGACCTATTGCATCTCCCGCCGTGCACAGGGTGTCACGTTGCAAGACCTGCCTGAAACCGA
ACTGCCCGCTGTTCTTCAGCCGGTCGCGGAGGCTATGGATGCTATCGCTGCGGCCGATCTTAGCCAGACG
AGCGGGTTCGGCCCATTCGGACCGCAAGGAATCGGTCAATACACTACATGGCGTGATTTCATATGCGCGA
TTGCTGATCCCCATGTGTATCACTGGCAAACTGTGATGGACGACACCGTCAGTGCGTCCGTCGCGCAGGC
TCTCGATGAGCTGATGCTTTGGGCGAGGACTGCCCCGAAGTCCGGCACTCGTGCACGCGGATTCGGCTCC
AACAATGTCCTGACGGACAATGGCCGCATAACAGCGGTCATTGACTGGAGCGAGGCGATGTTCGGGGATT
CCCAATACGAGGTCGCCAACATCTTCTTCTGGAGGCCGTGGTTGGCTTGTATGGAGCAGCAGACGCGCTA
CTTCGAGCGGAGGCATCCGGAGCTTGCAGGATCGCCAAGGCTCCGGGCGTATATGCTCCGCATTGGTCTT
GACCAACTCTATCAGAGCTTGGTTGACGGCAATTTCGATGATGCAGCTTGGGCGCAGGGTCGATGCGACG
CAATCGTCCGATCCGGAGCCGGGACTGTCGGGCGTACACAAATCGCCCGCAGAAGCGCGGCCGTCTGGAC
CGATGGCTGTGTAGAAGTACTCGCCGATAGTGGAAACCGACGCCCCAGCACTCGTCCGAGGGCAAAGGAA
TAGAGTAGATGCCGACCGAACAAGAGCTGATTTCGAGAACGCCTCAGCCAGCAACTCGCGCGAGCCTAGC
AAGGCAAATGCGAGAGAACGGCCTTACGCTTGGTGGCACAGTTCTCGTCCACAGTTCGCTAAGCTCGCTC
GGCTGGGTCGCGGGAGGGCCGGTCGCAGTGATTCAGGAATTAATTCCCTAGAGTCAAGCAGATCGTTCAA
ACATTTGGCAATAAAGTTTCTTAAGATTGAATCCTGTTGCCGGTCTTGCGATGATTATCATATAATTTCT
GTTGAATTACGTTAAGCATGTAATAATTAACATGTAATGCATGACGTTATTTATGAGATGGGTTTTTATG
ATTAGAGTCCCGCAATTATACATTTAATACGCGATAGAAAACAAAATATAGCGCGCAAACTAGGATAAAT
TATCGCGCGCGGTGTCATCTATGTTACTAGATCGACCGGCATGCAAGCTGATAATTCAATTCGGCGTTAA
TTCAGTACATTAAAAACGTCCGCAATGTGTTATTAAGTTGTCTAAGCGTCAATTTGTTTACACCACAATA
TATCCTGCCACCAGCCAGCCAACAGCTCCCCGACCGGCAGCTCGGCACAAAATCACCACTCGATACAGGC
AGCCCATCAGTCCGGGACGGCGTCAGCGGGAGAGCCGTTGTAAGGCGGCAGACTTTGCTCATGTTACCGA
TGCTATTCGGAAGAACGGCAACTAAGCTGCCGGGTTTGAAACACGGATGATCTCGCGGAGGGTAGCATGT
TGATTGTAACGATGACAGAGCGTTGCTGCCTGTGATCAATTCGGGCACGAACCCAGTGGACATAAGCCTC
GTTCGGTTCGTAAGCTGTAATGCAAGTAGCGTAACTGCCGTCACGCAACTGGTCCAGAACCTTGACCGAA
CGCAGCGGTGGTAACGGCGCAGTGGCGGTTTTCATGGCTTCTTGTTATGACATGTTTTTTTGGGGTACAG
TCTATGCCTCGGGCATCCAAGCAGCAAGCGCGTTACGCCGTGGGTCGATGTTTGATGTTATGGAGCAGCA
ACGATGTTACGCAGCAGGGCAGTCGCCCTAAAACAAAGTTAAACATCATGGGGGAAGCGGTGATCGCCGA
AGTATCGACTCAACTATCAGAGGTAGTTGGCGTCATCGAGCGCCATCTCGAACCGACGTTGCTGGCCGTA
CATTTGTACGGCTCCGCAGTGGATGGCGGCCTGAAGCCACACAGTGATATTGATTTGCTGGTTACGGTGA
CCGTAAGGCTTGATGAAACAACGCGGCGAGCTTTGATCAACGACCTTTTGGAAACTTCGGCTTCCCCTGG
AGAGAGCGAGATTCTCCGCGCTGTAGAAGTCACCATTGTTGTGCACGACGACATCATTCCGTGGCGTTAT
CCAGCTAAGCGCGAACTGCAATTTGGAGAATGGCAGCGCAATGACATTCTTGCAGGTATCTTCGAGCCAG
CCACGATCGACATTGATCTGGCTATCTTGCTGACAAAAGCAAGAGAACATAGCGTTGCCTTGGTAGGTCC
AGCGGCGGAGGAACTCTTTGATCCGGTTCCTGAACAGGATCTATTTGAGGCGCTAAATGAAACCTTAACG
CTATGGAACTCGCCGCCCGACTGGGCTGGCGATGAGCGAAATGTAGTGCTTACGTTGTCCCGCATTTGGT
ACAGCGCAGTAACCGGCAAAATCGCGCCGAAGGATGTCGCTGCCGACTGGGCAATGGAGCGCCTGCCGGC
CCAGTATCAGCCCGTCATACTTGAAGCTAGACAGGCTTATCTTGGACAAGAAGAAGATCGCTTGGCCTCG
CGCGCAGATCAGTTGGAAGAATTTGTCCACTACGTGAAAGGCGAGATCACCAAGGTAGTCGGCAAATAAT
GTCTAGCTAGAAATTCGTTCAAGCCGACGCCGCTTCGCCGGCGTTAACTCAAGCGATTAGATGCACTAAG
CACATAATTGCTCACAGCCAAACTATCAGGTCAAGTCTGCTTTTATTATTTTTAAGCGTGCATAATAAGC
CCTACACAAATTGGGAGATATATCATGCATGACCAAAATCCCTTAACGTGAGTTTTCGTTCCACTGAGCG
TCAGACCCCGTAGAAAAGATCAAAGGATCTTCTTGAGATCCTTTTTTTCTGCGCGTAATCTGCTGCTTGC
AAACAAAAAAACCACCGCTACCAGCGGTGGTTTGTTTGCCGGATCAAGAGCTACCAACTCTTTTTCCGAA

GGTAACTGGCTTCAGCAGAGCGCAGATACCAAATACTGTCCTTCTAGTGTAGCCGTAGTTAGGCCACCAC
TTCAAGAACTCTGTAGCACCGCCTACATACCTCGCTCTGCTAATCCTGTTACCAGTGGCTGCTGCCAGTG
GCGATAAGTCGTGTCTTACCGGGTTGGACTCAAGACGATAGTTACCGGATAAGGCGCAGCGGTCGGGCTG
AACGGGGGGTTCGTGCACACAGCCCAGCTTGGAGCGAACGACCTACACCGAACTGAGATACCTACAGCGT
GAGCTATGAGAAAGCGCCACGCTTCCCGAAGGGAGAAAGGCGGACAGGTATCCGGTAAGCGGCAGGGTCG
GAACAGGAGAGCGCACGAGGGAGCTTCCAGGGGGAAACGCCTGGTATCTTTATAGTCCTGTCGGGTTTCG
CCACCTCTGACTTGAGCGTCGATTTTTGTGATGCTCGTCAGGGGGGCGGAGCCTATGGAAAAACGCCAGC
AACGCGGCCTTTTTACGGTTCCTGGCCTTTTGCTGGCCTTTTGCTCACATGTTCTTTCCTGCGTTATCCC
CTGATTCTGTGGATAACCGTATTACCGCCTTTGAGTGAGCTGATACCGCTCGCCGCAGCCGAACGACCGA
GCGCAGCGAGTCAGTGAGCGAGGAAGCGGAAGAGCGCCTGATGCGGTATTTTCTCCTTACGCATCTGTGC
GGTATTTCACACCGCATATGGTGCACTCTCAGTACAATCTGCTCTGATGCCGCATAGTTAAGCCAGTATA
CACTCCGCTATCGCTACGTGACTGGGTCATGGCTGCGCCCCGACACCCGCCAACACCCGCTGACGCGCCC
TGACGGGCTTGTCTGCTCCCGGCATCCGCTTACAGACAAGCTGTGACCGTCTCCGGGAGCTGCATGTGTC
AGAGGTTTTCACCGTCATCACCGAAACGCGCGAGGCAGGGTGCCTTGATGTGGGCGCCGGCGGTCGAGTG
GCGACGGCGCGGCTTGTCCGCGCCCTGGTAGATTGCCTGGCCGTAGGCCAGCCATTTTTGAGCGGCCAGC
GGCCGCGATAGGCCGACGCGAAGCGGCGGGGCGTAGGGAGCGCAGCGACCGAAGGGTAGGCGCTTTTTGC
AGCTCTTCGGCTGTGCGCTGGCCAGACAGTTATGCACAGGCCAGGCGGGTTTTAAGAGTTTTAATAAGTT
TTAAAGAGTTTTAGGCGGAAAAATCGCCTTTTTTCTCTTTTATATCAGTCACTTACATGTGTGACCGGTT
CCCAATGTACGGCTTTGGGTTCCCAATGTACGGGTTCCGGTTCCCAATGTACGGCTTTGGGTTCCCAATG
TACGTGCTATCCACAGGAAAGAGACCTTTTCGACCTTTTTCCCCTGCTAGGGCAATTTGCCCTAGCATCT
GCTCCGTACATTAGGAACCGGCGGATGCTTCGCCCTCGATCAGGTTGCGGTAGCGCATGACTAGGATCGG
GCCAGCCTGCCCCGCCTCCTCCTTCAAATCGTACTCCGGCAGGTCATTTGACCCGATCAGCTTGCGCACG
GTGAAACAGAACTTCTTGAACTCTCCGGCGCTGCCACTGCGTTCGTAGATCGTCTTGAACAACCATCTGG
CTTCTGCCTTGCCTGCGGCGCGGCGTGCCAGGCGGTAGAGAAAACGGCCGATGCCGGGATCGATCAAAAA
GTAATCGGGGTGAACCGTCAGCACGTCCGGGTTCTTGCCTTCTGTGATCTCGCGGTACATCCAATCAGCT
AGCTCGATCTCGATGTACTCCGGCCGCCCGGTTTCGCTCTTTACGATCTTGTAGCGGCTAATCAAGGCTT
CACCCTCGGATACCGTCACCAGGCGGCCGTTCTTGGCCTTCTTCGTACGCTGCATGGCAACGTGCGTGGT
GTTTAACCGAATGCAGGTTTCTACCAGGTCGTCTTTCTGCTTTCCGCCATCGGCTCGCCGGCAGAACTTG
AGTACGTCCGCAACGTGTGGACGGAACACGCGGCCGGGCTTGTCTCCCTTCCCTTCCCGGTATCGGTTCA
TGGATTCGGTTAGATGGGAAACCGCCATCAGTACCAGGTCGTAATCCCACACACTGGCCATGCCGGCCGG
CCCTGCGGAAACCTCTACGTGCCCGTCTGGAAGCTCGTAGCGGATCACCTCGCCAGCTCGTCGGTCACGC
TTCGACAGACGGAAAACGGCCACGTCCATGATGCTGCGACTATCGCGGGTGCCCACGTCATAGAGCATCG
GAACGAAAAAATCTGGTTGCTCGTCGCCCTTGGGCGGCTTCCTAATCGACGGCGCACCGGCTGCCGGCGG
TTGCCGGGATTCTTTGCGGATTCGATCAGCGGCCGCTTGCCACGATTCACCGGGGCGTGCTTCTGCCTCG
ATGCGTTGCCGCTGGGCGGCCTGCGCGGCCTTCAACTTCTCCACCAGGTCATCACCCAGCGCCGCGCCGA
TTTGTACCGGGCCGGATGGTTTGCGACCGTCACGCCGATTCCTCGGGCTTGGGGGTTCCAGTGCCATTGC
AGGGCCGGCAGACAACCCAGCCGCTTACGCCTGGCCAACCGCCCGTTCCTCCACACATGGGGCATTCCAC
GGCGTCGGTGCCTGGTTGTTCTTGATTTTCCATGCCGCCTCCTTTAGCCGCTAAAATTCATCTACTCATT
TATTCATTTGCTCATTTACTCTGGTAGCTGCGCGATGTATTCAGATAGCAGCTCGGTAATGGTCTTGCCT
TGGCGTACCGCGTACATCTTCAGCTTGGTGTGATCCTCCGCCGGCAACTGAAAGTTGACCCGCTTCATGG
CTGGCGTGTCTGCCAGGCTGGCCAACGTTGCAGCCTTGCTGCTGCGTGCGCTCGGACGGCCGGCACTTAG
CGTGTTTGTGCTTTTGCTCATTTTCTCTTTACCTCATTAACTCAAATGAGTTTTGATTTAATTTCAGCGG
CCAGCGCCTGGACCTCGCGGGCAGCGTCGCCCTCGGGTTCTGATTCAAGAACGGTTGTGCCGGCGGCGGC
AGTGCCTGGGTAGCTCACGCGCTGCGTGATACGGGACTCAAGAATGGGCAGCTCGTACCCGGCCAGCGCC

TCGGCAACCTCACCGCCGATGCGCGTGCCTTTGATCGCCCGCGACACGACAAAGGCCGCTTGTAGCCTTC
CATCCGTGACCTCAATGCGCTGCTTAACCAGCTCCACCAGGTCGGCGGTGGCCCATATGTCGTAAGGGCT
TGGCTGCACCGGAATCAGCACGAAGTCGGCTGCCTTGATCGCGGACACAGCCAAGTCCGCCGCCTGGGGC
GCTCCGTCGATCACTACGAAGTCGCGCCGGCCGATGGCCTTCACGTCGCGGTCAATCGTCGGGCGGTCGA
TGCCGACAACGGTTAGCGGTTGATCTTCCCGCACGGCCGCCCAATCGCGGGCACTGCCCTGGGGATCGGA
ATCGACTAACAGAACATCGGCCCCGGCGAGTTGCAGGGCGCGGGCTAGATGGGTTGCGATGGTCGTCTTG
CCTGACCCGCCTTTCTGGTTAAGTACAGCGATAACCTTCATGCGTTCCCCTTGCGTATTTGTTTATTTAC
TCATCGCATCATATACGCAGCGACCGCATGACGCAAGCTGTTTTACTCAAATACACATCACCTTTTTAGA
CGGCGGCGCTCGGTTTCTTCAGCGGCCAAGCTGGCCGGCCAGGCCGCCAGCTTGGCATCAGACAAACCGG
CCAGGATTTCATGCAGCCGCACGGTTGAGACGTGCGCGGGCGGCTCGAACACGTACCCGGCCGCGATCAT
CTCCGCCTCGATCTCTTCGGTAATGAAAAACGGTTCGTCCTGGCCGTCCTGGTGCGGTTTCATGCTTGTT
CCTCTTGGCGTTCATTCTCGGCGGCCGCCAGGGCGTCGGCCTCGGTCAATGCGTCCTCACGGAAGGCACC
GCGCCGCCTGGCCTCGGTGGGCGTCACTTCCTCGCTGCGCTCAAGTGCGCGGTACAGGGTCGAGCGATGC
ACGCCAAGCAGTGCAGCCGCCTCTTTCACGGTGCGGCCTTCCTGGTCGATCAGCTCGCGGGCGTGCGCGA
TCTGTGCCGGGGTGAGGGTAGGGCGGGGGCCAAACTTCACGCCTCGGGCCTTGGCGGCCTCGCGCCCGCT
CCGGGTGCGGTCGATGATTAGGGAACGCTCGAACTCGGCAATGCCGGCGAACACGGTCAACACCATGCGG
CCGGCCGGCGTGGTGGTGTCGGCCCACGGCTCTGCCAGGCTACGCAGGCCCGCGCCGGCCTCCTGGATGC
GCTCGGCAATGTCCAGTAGGTCGCGGGTGCTGCGGGCCAGGCGGTCTAGCCTGGTCACTGTCACAACGTC
GCCAGGGCGTAGGTGGTCAAGCATCCTGGCCAGCTCCGGGCGGTCGCGCCTGGTGCCGGTGATCTTCTCG
GAAAACAGCTTGGTGCAGCCGGCCGCGTGCAGTTCGGCCCGTTGGTTGGTCAAGTCCTGGTCGTCGGTGC
TGACGCGGGCATAGCCCAGCAGGCCAGCGGCGGCGCTCTTGTTCATGGCGTAATGTCTCCGGTTCTAGTC
GCAAGTATTCTACTTTATGCGACTAAAACACGCGACAAGAAAACGCCAGGAAAAGGGCAGGGCGGCAGCC
TGTCGCGTAACTTAGGACTTGTGCGACATGTCGTTTTCAGAAGACGGCTGCACTGAACGTCAGAAGCCGA
CTGCACTATAGCAGCGGAGGGGTTGGATCAAAGTACTTTGATCCCGAGGGGAACCCTGTGGTTGGCATGC
ACATACAAATGGACGAACGGATAAACCTTTTCACGCCCTTTTAAATATCCGTTATTCTAATAAACGCTCT
TTTCTCTTAGGTTTACCCGCCAATATATCCTGTCAAACACTGATAGTTTAAACTGAAGGCGGGAAACGAC
AATCTGATCCAAGCTCAAGCTAAGCTTGAGCTCTGCATGCCTGCAGGTCCCCAGATTAGCCTTTTCAATT
TCAGAAAGAATGCTAACCCACAGATGGTTAGAGAGGCTTACGCAGCAGGTCTCATCAAGACGATCTACCC
GAGCAATAATCTCCAGGAAATCAAATACCTTCCCAAGAAGGTTAAAGATGCAGTCAAAAGATTCAGGACT
AACTGCATCAAGAACACAGAGAAAGATATATTTCTCAAGATCAGAAGTACTATTCCAGTATGGACGATTC
AAGGCTTGCTTCACAAACCAAGGCAAGTAATAGAGATTGGAGTCTCTAAAAAGGTAGTTCCCACTGAATC
AAAGGCCATGGAGTCAAAGATTCAAATAGAGGACCTAACAGAACTCGCCGTAAAGACTGGCGAACAGTTC
ATACAGAGTCTCTTACGACTCAATGACAAGAAGAAAATCTTCGTCAACATGGTGGAGCACGACACACTTG
TCTACTCCAAAAATATCAAAGATACAGTCTCAGAAGACCAAAGGGCAATTGAGACTTTTCAACAAAGGGT
AATATCCGGAAACCTCCTCGGATTCCATTGCCCAGCTATCTGTCACTTTATTGTGAAGATAGTGGAAAAG
GAAGGTGGCTCCTACAAATGCCATCATTGCGATAAAGGAAAGGCCATCGTTGAAGATGCCTCTGCCGACA
GTGGTCCCAAAGATGGACCCCCACCCACGAGGAGCATCGTGGAAAAAGAAGACGTTCCAACCACGTCTTC
AAAGCAAGTGGATTGATGTGATATCTCCACTGACGTAAGGGATGACGCACAATCCCACTATCCTTCGCAA
GACCCTTCCTCTATATAAGGAAGTTCATTTCATTTGGAGAGAACACGGGGGACTCTAGAGGATCCAAGGA
GATATAAGCCGCCACCATGGATTACAAGGACGATGATGACAAGGACTACAAGGACGATGACGATAAGGAC
TATAAGGATGACGATGACAAGGATTACGCCGAGGATGGATGATACTAGCGGACAAACTTTCGTTTCTGTG
AACCTTTCTGACGAAAGCAACACGGCAAGCACTAAAGTTGAAAACGTAAAATCGGAAGCGGATCGCTTGG
AGTTTTTACGTAAAATGAATCCCTTTATTGTTGACGCTCTGGTGCGGAAAACCAATTATCAGGGTGCTCG
CTTTCGCGCAAGAATAATAGGAGTGTGCGTGGATTGTGGTAGAAAACACGACAAGGCGCTCAAGACTGAA

CGTAAGTGTAAGGTCAACAACACGCAATCTCAGAACGAGGTGGCGCATATGTTGATGCACGATCCCGTTA
AGTATTTGAACAAAAGAAAGGCTAGAGCCTTTTCTAACGCAGAGATGTTTGCGATTGAACTGGTTTCGTA
CACCAAGGAAAGGCAATTGGCGGTCGATTTAGCCGCTGAAAGGGAGAAGACGAGAGCGGCTCGTAGACAC
CCAATACGTTCTCCGGAAGAAACTCCGGAACATTATAAATTCGGTATGACTGCTAAGGCAATGTTACCGG
ACATCAACGCCGTAGACGTTGGTGATAACGAGGAAACTTCGTCGGAGTACCCAGTGAGTCTGAGTGTTTC
TGGCGGAGTTCTCCGTGAACACCACTTCATCTGACCTAATGATATCCCGCGGCCATGCTAGAGTCCGCAA
AAATCACCAGTCTCTCTCTACAAATCTATCTCTCTCTATTTTTCTCCAGAATAATGTGTGAGTAGTTCCC
AGATAAGGGAATTAGGGTTCTTATAGGGTTTCGCTCATGTGTTGAGCATATAAGAAACCCTTAGTATGTA
TTTGTATTTGTAAAATACTTCTATCAATAAAATTTCTAATTCCTAAAACCAAAATCCAGTGACCTGCAGG
CATGCGACGTCG

Sequence of pH7LIC3.1.1-p23M16 (35S- p23M16)

GGGTACCCTCGAATTATCATACATGAGAATTAAGGGAGTCACGTTATGACCCCGCCGATGACGCGGGACA
AGCCGTTTTACGTTTGGAACTGACAGAACCGCAACGTTGAAGGAGCCACTGAGCCGCGGGTTTCTGGAGT
TTAATGAGCTAAGCACATACGTCAGAAACCATTATTGCGCGTTCAAAAGTCGCCTAAGGTCACTATCAGC
TAGCAAATATTTCTTGTCAAAAATGCTCCACTGACGTTCCATAAATTCCCCTCGGTATCCAATTAGAGTC
TCATATTCACTCTCAACTCGAATCCCCCCTATGAAAAAGCCTGAACTCACCGCGACGTCTGTCGAGAAGT
TTCTGATCGAAAAGTTCGACAGCGTCTCCGACCTGATGCAGCTCTCGGAGGGCGAAGAATCTCGTGCTTT
CAGCTTCGATGTAGGAGGGCGTGGATATGTCCTGCGGGTAAATAGCTGCGCCGATGGTTTCTACAAAGAT
CGTTATGTTTATCGGCACTTTGCATCGGCCGCGCTCCCGATTCCGGAAGTGCTTGACATTGGGGAGTTCA
GCGAGAGCCTGACCTATTGCATCTCCCGCCGTGCACAGGGTGTCACGTTGCAAGACCTGCCTGAAACCGA
ACTGCCCGCTGTTCTTCAGCCGGTCGCGGAGGCTATGGATGCTATCGCTGCGGCCGATCTTAGCCAGACG
AGCGGGTTCGGCCCATTCGGACCGCAAGGAATCGGTCAATACACTACATGGCGTGATTTCATATGCGCGA
TTGCTGATCCCCATGTGTATCACTGGCAAACTGTGATGGACGACACCGTCAGTGCGTCCGTCGCGCAGGC
TCTCGATGAGCTGATGCTTTGGGCGAGGACTGCCCCGAAGTCCGGCACTCGTGCACGCGGATTCGGCTCC
AACAATGTCCTGACGGACAATGGCCGCATAACAGCGGTCATTGACTGGAGCGAGGCGATGTTCGGGGATT
CCCAATACGAGGTCGCCAACATCTTCTTCTGGAGGCCGTGGTTGGCTTGTATGGAGCAGCAGACGCGCTA
CTTCGAGCGGAGGCATCCGGAGCTTGCAGGATCGCCAAGGCTCCGGGCGTATATGCTCCGCATTGGTCTT
GACCAACTCTATCAGAGCTTGGTTGACGGCAATTTCGATGATGCAGCTTGGGCGCAGGGTCGATGCGACG
CAATCGTCCGATCCGGAGCCGGGACTGTCGGGCGTACACAAATCGCCCGCAGAAGCGCGGCCGTCTGGAC
CGATGGCTGTGTAGAAGTACTCGCCGATAGTGGAAACCGACGCCCCAGCACTCGTCCGAGGGCAAAGGAA
TAGAGTAGATGCCGACCGAACAAGAGCTGATTTCGAGAACGCCTCAGCCAGCAACTCGCGCGAGCCTAGC
AAGGCAAATGCGAGAGAACGGCCTTACGCTTGGTGGCACAGTTCTCGTCCACAGTTCGCTAAGCTCGCTC
GGCTGGGTCGCGGGAGGGCCGGTCGCAGTGATTCAGGAATTAATTCCCTAGAGTCAAGCAGATCGTTCAA
ACATTTGGCAATAAAGTTTCTTAAGATTGAATCCTGTTGCCGGTCTTGCGATGATTATCATATAATTTCT
GTTGAATTACGTTAAGCATGTAATAATTAACATGTAATGCATGACGTTATTTATGAGATGGGTTTTTATG
ATTAGAGTCCCGCAATTATACATTTAATACGCGATAGAAAACAAAATATAGCGCGCAAACTAGGATAAAT
TATCGCGCGCGGTGTCATCTATGTTACTAGATCGACCGGCATGCAAGCTGATAATTCAATTCGGCGTTAA
TTCAGTACATTAAAAACGTCCGCAATGTGTTATTAAGTTGTCTAAGCGTCAATTTGTTTACACCACAATA
TATCCTGCCACCAGCCAGCCAACAGCTCCCCGACCGGCAGCTCGGCACAAAATCACCACTCGATACAGGC
AGCCCATCAGTCCGGGACGGCGTCAGCGGGAGAGCCGTTGTAAGGCGGCAGACTTTGCTCATGTTACCGA
TGCTATTCGGAAGAACGGCAACTAAGCTGCCGGGTTTGAAACACGGATGATCTCGCGGAGGGTAGCATGT
TGATTGTAACGATGACAGAGCGTTGCTGCCTGTGATCAATTCGGGCACGAACCCAGTGGACATAAGCCTC
GTTCGGTTCGTAAGCTGTAATGCAAGTAGCGTAACTGCCGTCACGCAACTGGTCCAGAACCTTGACCGAA

CGCAGCGGTGGTAACGGCGCAGTGGCGGTTTTCATGGCTTCTTGTTATGACATGTTTTTTTGGGGTACAG
TCTATGCCTCGGGCATCCAAGCAGCAAGCGCGTTACGCCGTGGGTCGATGTTTGATGTTATGGAGCAGCA
ACGATGTTACGCAGCAGGGCAGTCGCCCTAAAACAAAGTTAAACATCATGGGGGAAGCGGTGATCGCCGA
AGTATCGACTCAACTATCAGAGGTAGTTGGCGTCATCGAGCGCCATCTCGAACCGACGTTGCTGGCCGTA
CATTTGTACGGCTCCGCAGTGGATGGCGGCCTGAAGCCACACAGTGATATTGATTTGCTGGTTACGGTGA
CCGTAAGGCTTGATGAAACAACGCGGCGAGCTTTGATCAACGACCTTTTGGAAACTTCGGCTTCCCCTGG
AGAGAGCGAGATTCTCCGCGCTGTAGAAGTCACCATTGTTGTGCACGACGACATCATTCCGTGGCGTTAT
CCAGCTAAGCGCGAACTGCAATTTGGAGAATGGCAGCGCAATGACATTCTTGCAGGTATCTTCGAGCCAG
CCACGATCGACATTGATCTGGCTATCTTGCTGACAAAAGCAAGAGAACATAGCGTTGCCTTGGTAGGTCC
AGCGGCGGAGGAACTCTTTGATCCGGTTCCTGAACAGGATCTATTTGAGGCGCTAAATGAAACCTTAACG
CTATGGAACTCGCCGCCCGACTGGGCTGGCGATGAGCGAAATGTAGTGCTTACGTTGTCCCGCATTTGGT
ACAGCGCAGTAACCGGCAAAATCGCGCCGAAGGATGTCGCTGCCGACTGGGCAATGGAGCGCCTGCCGGC
CCAGTATCAGCCCGTCATACTTGAAGCTAGACAGGCTTATCTTGGACAAGAAGAAGATCGCTTGGCCTCG
CGCGCAGATCAGTTGGAAGAATTTGTCCACTACGTGAAAGGCGAGATCACCAAGGTAGTCGGCAAATAAT
GTCTAGCTAGAAATTCGTTCAAGCCGACGCCGCTTCGCCGGCGTTAACTCAAGCGATTAGATGCACTAAG
CACATAATTGCTCACAGCCAAACTATCAGGTCAAGTCTGCTTTTATTATTTTTAAGCGTGCATAATAAGC
CCTACACAAATTGGGAGATATATCATGCATGACCAAAATCCCTTAACGTGAGTTTTCGTTCCACTGAGCG
TCAGACCCCGTAGAAAAGATCAAAGGATCTTCTTGAGATCCTTTTTTTCTGCGCGTAATCTGCTGCTTGC
AAACAAAAAAACCACCGCTACCAGCGGTGGTTTGTTTGCCGGATCAAGAGCTACCAACTCTTTTTCCGAA
GGTAACTGGCTTCAGCAGAGCGCAGATACCAAATACTGTCCTTCTAGTGTAGCCGTAGTTAGGCCACCAC
TTCAAGAACTCTGTAGCACCGCCTACATACCTCGCTCTGCTAATCCTGTTACCAGTGGCTGCTGCCAGTG
GCGATAAGTCGTGTCTTACCGGGTTGGACTCAAGACGATAGTTACCGGATAAGGCGCAGCGGTCGGGCTG
AACGGGGGGTTCGTGCACACAGCCCAGCTTGGAGCGAACGACCTACACCGAACTGAGATACCTACAGCGT
GAGCTATGAGAAAGCGCCACGCTTCCCGAAGGGAGAAAGGCGGACAGGTATCCGGTAAGCGGCAGGGTCG
GAACAGGAGAGCGCACGAGGGAGCTTCCAGGGGGAAACGCCTGGTATCTTTATAGTCCTGTCGGGTTTCG
CCACCTCTGACTTGAGCGTCGATTTTTGTGATGCTCGTCAGGGGGGCGGAGCCTATGGAAAAACGCCAGC
AACGCGGCCTTTTTACGGTTCCTGGCCTTTTGCTGGCCTTTTGCTCACATGTTCTTTCCTGCGTTATCCC
CTGATTCTGTGGATAACCGTATTACCGCCTTTGAGTGAGCTGATACCGCTCGCCGCAGCCGAACGACCGA
GCGCAGCGAGTCAGTGAGCGAGGAAGCGGAAGAGCGCCTGATGCGGTATTTTCTCCTTACGCATCTGTGC
GGTATTTCACACCGCATATGGTGCACTCTCAGTACAATCTGCTCTGATGCCGCATAGTTAAGCCAGTATA
CACTCCGCTATCGCTACGTGACTGGGTCATGGCTGCGCCCCGACACCCGCCAACACCCGCTGACGCGCCC
TGACGGGCTTGTCTGCTCCCGGCATCCGCTTACAGACAAGCTGTGACCGTCTCCGGGAGCTGCATGTGTC
AGAGGTTTTCACCGTCATCACCGAAACGCGCGAGGCAGGGTGCCTTGATGTGGGCGCCGGCGGTCGAGTG
GCGACGGCGCGGCTTGTCCGCGCCCTGGTAGATTGCCTGGCCGTAGGCCAGCCATTTTTGAGCGGCCAGC
GGCCGCGATAGGCCGACGCGAAGCGGCGGGGCGTAGGGAGCGCAGCGACCGAAGGGTAGGCGCTTTTTGC
AGCTCTTCGGCTGTGCGCTGGCCAGACAGTTATGCACAGGCCAGGCGGGTTTTAAGAGTTTTAATAAGTT
TTAAAGAGTTTTAGGCGGAAAAATCGCCTTTTTTCTCTTTTATATCAGTCACTTACATGTGTGACCGGTT
CCCAATGTACGGCTTTGGGTTCCCAATGTACGGGTTCCGGTTCCCAATGTACGGCTTTGGGTTCCCAATG
TACGTGCTATCCACAGGAAAGAGACCTTTTCGACCTTTTTCCCCTGCTAGGGCAATTTGCCCTAGCATCT
GCTCCGTACATTAGGAACCGGCGGATGCTTCGCCCTCGATCAGGTTGCGGTAGCGCATGACTAGGATCGG
GCCAGCCTGCCCCGCCTCCTCCTTCAAATCGTACTCCGGCAGGTCATTTGACCCGATCAGCTTGCGCACG
GTGAAACAGAACTTCTTGAACTCTCCGGCGCTGCCACTGCGTTCGTAGATCGTCTTGAACAACCATCTGG
CTTCTGCCTTGCCTGCGGCGCGGCGTGCCAGGCGGTAGAGAAAACGGCCGATGCCGGGATCGATCAAAAA
GTAATCGGGGTGAACCGTCAGCACGTCCGGGTTCTTGCCTTCTGTGATCTCGCGGTACATCCAATCAGCT

AGCTCGATCTCGATGTACTCCGGCCGCCCGGTTTCGCTCTTTACGATCTTGTAGCGGCTAATCAAGGCTT
CACCCTCGGATACCGTCACCAGGCGGCCGTTCTTGGCCTTCTTCGTACGCTGCATGGCAACGTGCGTGGT
GTTTAACCGAATGCAGGTTTCTACCAGGTCGTCTTTCTGCTTTCCGCCATCGGCTCGCCGGCAGAACTTG
AGTACGTCCGCAACGTGTGGACGGAACACGCGGCCGGGCTTGTCTCCCTTCCCTTCCCGGTATCGGTTCA
TGGATTCGGTTAGATGGGAAACCGCCATCAGTACCAGGTCGTAATCCCACACACTGGCCATGCCGGCCGG
CCCTGCGGAAACCTCTACGTGCCCGTCTGGAAGCTCGTAGCGGATCACCTCGCCAGCTCGTCGGTCACGC
TTCGACAGACGGAAAACGGCCACGTCCATGATGCTGCGACTATCGCGGGTGCCCACGTCATAGAGCATCG
GAACGAAAAAATCTGGTTGCTCGTCGCCCTTGGGCGGCTTCCTAATCGACGGCGCACCGGCTGCCGGCGG
TTGCCGGGATTCTTTGCGGATTCGATCAGCGGCCGCTTGCCACGATTCACCGGGGCGTGCTTCTGCCTCG
ATGCGTTGCCGCTGGGCGGCCTGCGCGGCCTTCAACTTCTCCACCAGGTCATCACCCAGCGCCGCGCCGA
TTTGTACCGGGCCGGATGGTTTGCGACCGTCACGCCGATTCCTCGGGCTTGGGGGTTCCAGTGCCATTGC
AGGGCCGGCAGACAACCCAGCCGCTTACGCCTGGCCAACCGCCCGTTCCTCCACACATGGGGCATTCCAC
GGCGTCGGTGCCTGGTTGTTCTTGATTTTCCATGCCGCCTCCTTTAGCCGCTAAAATTCATCTACTCATT
TATTCATTTGCTCATTTACTCTGGTAGCTGCGCGATGTATTCAGATAGCAGCTCGGTAATGGTCTTGCCT
TGGCGTACCGCGTACATCTTCAGCTTGGTGTGATCCTCCGCCGGCAACTGAAAGTTGACCCGCTTCATGG
CTGGCGTGTCTGCCAGGCTGGCCAACGTTGCAGCCTTGCTGCTGCGTGCGCTCGGACGGCCGGCACTTAG
CGTGTTTGTGCTTTTGCTCATTTTCTCTTTACCTCATTAACTCAAATGAGTTTTGATTTAATTTCAGCGG
CCAGCGCCTGGACCTCGCGGGCAGCGTCGCCCTCGGGTTCTGATTCAAGAACGGTTGTGCCGGCGGCGGC
AGTGCCTGGGTAGCTCACGCGCTGCGTGATACGGGACTCAAGAATGGGCAGCTCGTACCCGGCCAGCGCC
TCGGCAACCTCACCGCCGATGCGCGTGCCTTTGATCGCCCGCGACACGACAAAGGCCGCTTGTAGCCTTC
CATCCGTGACCTCAATGCGCTGCTTAACCAGCTCCACCAGGTCGGCGGTGGCCCATATGTCGTAAGGGCT
TGGCTGCACCGGAATCAGCACGAAGTCGGCTGCCTTGATCGCGGACACAGCCAAGTCCGCCGCCTGGGGC
GCTCCGTCGATCACTACGAAGTCGCGCCGGCCGATGGCCTTCACGTCGCGGTCAATCGTCGGGCGGTCGA
TGCCGACAACGGTTAGCGGTTGATCTTCCCGCACGGCCGCCCAATCGCGGGCACTGCCCTGGGGATCGGA
ATCGACTAACAGAACATCGGCCCCGGCGAGTTGCAGGGCGCGGGCTAGATGGGTTGCGATGGTCGTCTTG
CCTGACCCGCCTTTCTGGTTAAGTACAGCGATAACCTTCATGCGTTCCCCTTGCGTATTTGTTTATTTAC
TCATCGCATCATATACGCAGCGACCGCATGACGCAAGCTGTTTTACTCAAATACACATCACCTTTTTAGA
CGGCGGCGCTCGGTTTCTTCAGCGGCCAAGCTGGCCGGCCAGGCCGCCAGCTTGGCATCAGACAAACCGG
CCAGGATTTCATGCAGCCGCACGGTTGAGACGTGCGCGGGCGGCTCGAACACGTACCCGGCCGCGATCAT
CTCCGCCTCGATCTCTTCGGTAATGAAAAACGGTTCGTCCTGGCCGTCCTGGTGCGGTTTCATGCTTGTT
CCTCTTGGCGTTCATTCTCGGCGGCCGCCAGGGCGTCGGCCTCGGTCAATGCGTCCTCACGGAAGGCACC
GCGCCGCCTGGCCTCGGTGGGCGTCACTTCCTCGCTGCGCTCAAGTGCGCGGTACAGGGTCGAGCGATGC
ACGCCAAGCAGTGCAGCCGCCTCTTTCACGGTGCGGCCTTCCTGGTCGATCAGCTCGCGGGCGTGCGCGA
TCTGTGCCGGGGTGAGGGTAGGGCGGGGGCCAAACTTCACGCCTCGGGCCTTGGCGGCCTCGCGCCCGCT
CCGGGTGCGGTCGATGATTAGGGAACGCTCGAACTCGGCAATGCCGGCGAACACGGTCAACACCATGCGG
CCGGCCGGCGTGGTGGTGTCGGCCCACGGCTCTGCCAGGCTACGCAGGCCCGCGCCGGCCTCCTGGATGC
GCTCGGCAATGTCCAGTAGGTCGCGGGTGCTGCGGGCCAGGCGGTCTAGCCTGGTCACTGTCACAACGTC
GCCAGGGCGTAGGTGGTCAAGCATCCTGGCCAGCTCCGGGCGGTCGCGCCTGGTGCCGGTGATCTTCTCG
GAAAACAGCTTGGTGCAGCCGGCCGCGTGCAGTTCGGCCCGTTGGTTGGTCAAGTCCTGGTCGTCGGTGC
TGACGCGGGCATAGCCCAGCAGGCCAGCGGCGGCGCTCTTGTTCATGGCGTAATGTCTCCGGTTCTAGTC
GCAAGTATTCTACTTTATGCGACTAAAACACGCGACAAGAAAACGCCAGGAAAAGGGCAGGGCGGCAGCC
TGTCGCGTAACTTAGGACTTGTGCGACATGTCGTTTTCAGAAGACGGCTGCACTGAACGTCAGAAGCCGA
CTGCACTATAGCAGCGGAGGGGTTGGATCAAAGTACTTTGATCCCGAGGGGAACCCTGTGGTTGGCATGC
ACATACAAATGGACGAACGGATAAACCTTTTCACGCCCTTTTAAATATCCGTTATTCTAATAAACGCTCT

TTTCTCTTAGGTTTACCCGCCAATATATCCTGTCAAACACTGATAGTTTAAACTGAAGGCGGGAAACGAC
AATCTGATCCAAGCTCAAGCTAAGCTTGAGCTCTGCATGCCTGCAGGTCCCCAGATTAGCCTTTTCAATT
TCAGAAAGAATGCTAACCCACAGATGGTTAGAGAGGCTTACGCAGCAGGTCTCATCAAGACGATCTACCC
GAGCAATAATCTCCAGGAAATCAAATACCTTCCCAAGAAGGTTAAAGATGCAGTCAAAAGATTCAGGACT
AACTGCATCAAGAACACAGAGAAAGATATATTTCTCAAGATCAGAAGTACTATTCCAGTATGGACGATTC
AAGGCTTGCTTCACAAACCAAGGCAAGTAATAGAGATTGGAGTCTCTAAAAAGGTAGTTCCCACTGAATC
AAAGGCCATGGAGTCAAAGATTCAAATAGAGGACCTAACAGAACTCGCCGTAAAGACTGGCGAACAGTTC
ATACAGAGTCTCTTACGACTCAATGACAAGAAGAAAATCTTCGTCAACATGGTGGAGCACGACACACTTG
TCTACTCCAAAAATATCAAAGATACAGTCTCAGAAGACCAAAGGGCAATTGAGACTTTTCAACAAAGGGT
AATATCCGGAAACCTCCTCGGATTCCATTGCCCAGCTATCTGTCACTTTATTGTGAAGATAGTGGAAAAG
GAAGGTGGCTCCTACAAATGCCATCATTGCGATAAAGGAAAGGCCATCGTTGAAGATGCCTCTGCCGACA
GTGGTCCCAAAGATGGACCCCCACCCACGAGGAGCATCGTGGAAAAAGAAGACGTTCCAACCACGTCTTC
AAAGCAAGTGGATTGATGTGATATCTCCACTGACGTAAGGGATGACGCACAATCCCACTATCCTTCGCAA
GACCCTTCCTCTATATAAGGAAGTTCATTTCATTTGGAGAGAACACGGGGGACTCTAGAGGATCCAAGGA
GATATAAGCCGCCACCATGGATTACAAGGACGATGATGACAAGGACTACAAGGACGATGACGATAAGGAC
TATAAGGATGACGATGACAAGGATTACGCCGAGGATGGATGATACTAGCGGACAAACTTTCGTTTCTGTG
AACCTTTCTGACGAAAGCAACACGGCAAGCACTAAAGTTGAAAACGTAAAATCGGAAGCGGATCGCTTGG
AGTTTTTACGTAAAATGAATCCCTTTATTGTTGACGCTCTGGTGCGGAAAACCAATTATCAGGGTGCTCG
CTTTCGCGCAAGAATAATAGGAGTGTGCGTGGATTGTGGTAGAAAACACGACAAGGCGCTCAAGACTGAA
CGTAAGTGTAAGGTCAACAACACGCAATCTCAGAACGAGGTGGCGCATATGTTGATGCACGATCCCGTTA
AGTATTTGAACAAAAGAAAGGCTAGAGCCTTTTCTAACGCAGAGATGTTTGCGATTGAACTGGTTTCGTA
CACCAAGGAAAGGCAATTGGCGGTCGATTTAGCCGCTGAAAGGGAGAAGACGAGACTGGCTCGTAGACAC
CCAATACGTTCTCCGGAAGAAACTCCGGAACATTATAAAGCGGCGATGACTGCTAAGGCAATGTTACCGG
ACATCAACGCCGTAGACGTTGGTGATAACGAGGAAACTTCGTCGGAGTACCCAGTGAGTCTGAGTGTTTC
TGGCGGAGTTCTCCGTGAACACCACTTCATCTGACCTAATGATATCCCGCGGCCATGCTAGAGTCCGCAA
AAATCACCAGTCTCTCTCTACAAATCTATCTCTCTCTATTTTTCTCCAGAATAATGTGTGAGTAGTTCCC
AGATAAGGGAATTAGGGTTCTTATAGGGTTTCGCTCATGTGTTGAGCATATAAGAAACCCTTAGTATGTA
TTTGTATTTGTAAAATACTTCTATCAATAAAATTTCTAATTCCTAAAACCAAAATCCAGTGACCTGCAGG
CATGCGACGTCG

Sequence of pH7LIC3.1.1-p23M17 (35S- p23M17)

GGGTACCCTCGAATTATCATACATGAGAATTAAGGGAGTCACGTTATGACCCCGCCGATGACGCGGGACA
AGCCGTTTTACGTTTGGAACTGACAGAACCGCAACGTTGAAGGAGCCACTGAGCCGCGGGTTTCTGGAGT
TTAATGAGCTAAGCACATACGTCAGAAACCATTATTGCGCGTTCAAAAGTCGCCTAAGGTCACTATCAGC
TAGCAAATATTTCTTGTCAAAAATGCTCCACTGACGTTCCATAAATTCCCCTCGGTATCCAATTAGAGTC
TCATATTCACTCTCAACTCGAATCCCCCCTATGAAAAAGCCTGAACTCACCGCGACGTCTGTCGAGAAGT
TTCTGATCGAAAAGTTCGACAGCGTCTCCGACCTGATGCAGCTCTCGGAGGGCGAAGAATCTCGTGCTTT
CAGCTTCGATGTAGGAGGGCGTGGATATGTCCTGCGGGTAAATAGCTGCGCCGATGGTTTCTACAAAGAT
CGTTATGTTTATCGGCACTTTGCATCGGCCGCGCTCCCGATTCCGGAAGTGCTTGACATTGGGGAGTTCA
GCGAGAGCCTGACCTATTGCATCTCCCGCCGTGCACAGGGTGTCACGTTGCAAGACCTGCCTGAAACCGA
ACTGCCCGCTGTTCTTCAGCCGGTCGCGGAGGCTATGGATGCTATCGCTGCGGCCGATCTTAGCCAGACG
AGCGGGTTCGGCCCATTCGGACCGCAAGGAATCGGTCAATACACTACATGGCGTGATTTCATATGCGCGA
TTGCTGATCCCCATGTGTATCACTGGCAAACTGTGATGGACGACACCGTCAGTGCGTCCGTCGCGCAGGC
TCTCGATGAGCTGATGCTTTGGGCGAGGACTGCCCCGAAGTCCGGCACTCGTGCACGCGGATTCGGCTCC

AACAATGTCCTGACGGACAATGGCCGCATAACAGCGGTCATTGACTGGAGCGAGGCGATGTTCGGGGATT
CCCAATACGAGGTCGCCAACATCTTCTTCTGGAGGCCGTGGTTGGCTTGTATGGAGCAGCAGACGCGCTA
CTTCGAGCGGAGGCATCCGGAGCTTGCAGGATCGCCAAGGCTCCGGGCGTATATGCTCCGCATTGGTCTT
GACCAACTCTATCAGAGCTTGGTTGACGGCAATTTCGATGATGCAGCTTGGGCGCAGGGTCGATGCGACG
CAATCGTCCGATCCGGAGCCGGGACTGTCGGGCGTACACAAATCGCCCGCAGAAGCGCGGCCGTCTGGAC
CGATGGCTGTGTAGAAGTACTCGCCGATAGTGGAAACCGACGCCCCAGCACTCGTCCGAGGGCAAAGGAA
TAGAGTAGATGCCGACCGAACAAGAGCTGATTTCGAGAACGCCTCAGCCAGCAACTCGCGCGAGCCTAGC
AAGGCAAATGCGAGAGAACGGCCTTACGCTTGGTGGCACAGTTCTCGTCCACAGTTCGCTAAGCTCGCTC
GGCTGGGTCGCGGGAGGGCCGGTCGCAGTGATTCAGGAATTAATTCCCTAGAGTCAAGCAGATCGTTCAA
ACATTTGGCAATAAAGTTTCTTAAGATTGAATCCTGTTGCCGGTCTTGCGATGATTATCATATAATTTCT
GTTGAATTACGTTAAGCATGTAATAATTAACATGTAATGCATGACGTTATTTATGAGATGGGTTTTTATG
ATTAGAGTCCCGCAATTATACATTTAATACGCGATAGAAAACAAAATATAGCGCGCAAACTAGGATAAAT
TATCGCGCGCGGTGTCATCTATGTTACTAGATCGACCGGCATGCAAGCTGATAATTCAATTCGGCGTTAA
TTCAGTACATTAAAAACGTCCGCAATGTGTTATTAAGTTGTCTAAGCGTCAATTTGTTTACACCACAATA
TATCCTGCCACCAGCCAGCCAACAGCTCCCCGACCGGCAGCTCGGCACAAAATCACCACTCGATACAGGC
AGCCCATCAGTCCGGGACGGCGTCAGCGGGAGAGCCGTTGTAAGGCGGCAGACTTTGCTCATGTTACCGA
TGCTATTCGGAAGAACGGCAACTAAGCTGCCGGGTTTGAAACACGGATGATCTCGCGGAGGGTAGCATGT
TGATTGTAACGATGACAGAGCGTTGCTGCCTGTGATCAATTCGGGCACGAACCCAGTGGACATAAGCCTC
GTTCGGTTCGTAAGCTGTAATGCAAGTAGCGTAACTGCCGTCACGCAACTGGTCCAGAACCTTGACCGAA
CGCAGCGGTGGTAACGGCGCAGTGGCGGTTTTCATGGCTTCTTGTTATGACATGTTTTTTTGGGGTACAG
TCTATGCCTCGGGCATCCAAGCAGCAAGCGCGTTACGCCGTGGGTCGATGTTTGATGTTATGGAGCAGCA
ACGATGTTACGCAGCAGGGCAGTCGCCCTAAAACAAAGTTAAACATCATGGGGGAAGCGGTGATCGCCGA
AGTATCGACTCAACTATCAGAGGTAGTTGGCGTCATCGAGCGCCATCTCGAACCGACGTTGCTGGCCGTA
CATTTGTACGGCTCCGCAGTGGATGGCGGCCTGAAGCCACACAGTGATATTGATTTGCTGGTTACGGTGA
CCGTAAGGCTTGATGAAACAACGCGGCGAGCTTTGATCAACGACCTTTTGGAAACTTCGGCTTCCCCTGG
AGAGAGCGAGATTCTCCGCGCTGTAGAAGTCACCATTGTTGTGCACGACGACATCATTCCGTGGCGTTAT
CCAGCTAAGCGCGAACTGCAATTTGGAGAATGGCAGCGCAATGACATTCTTGCAGGTATCTTCGAGCCAG
CCACGATCGACATTGATCTGGCTATCTTGCTGACAAAAGCAAGAGAACATAGCGTTGCCTTGGTAGGTCC
AGCGGCGGAGGAACTCTTTGATCCGGTTCCTGAACAGGATCTATTTGAGGCGCTAAATGAAACCTTAACG
CTATGGAACTCGCCGCCCGACTGGGCTGGCGATGAGCGAAATGTAGTGCTTACGTTGTCCCGCATTTGGT
ACAGCGCAGTAACCGGCAAAATCGCGCCGAAGGATGTCGCTGCCGACTGGGCAATGGAGCGCCTGCCGGC
CCAGTATCAGCCCGTCATACTTGAAGCTAGACAGGCTTATCTTGGACAAGAAGAAGATCGCTTGGCCTCG
CGCGCAGATCAGTTGGAAGAATTTGTCCACTACGTGAAAGGCGAGATCACCAAGGTAGTCGGCAAATAAT
GTCTAGCTAGAAATTCGTTCAAGCCGACGCCGCTTCGCCGGCGTTAACTCAAGCGATTAGATGCACTAAG
CACATAATTGCTCACAGCCAAACTATCAGGTCAAGTCTGCTTTTATTATTTTTAAGCGTGCATAATAAGC
CCTACACAAATTGGGAGATATATCATGCATGACCAAAATCCCTTAACGTGAGTTTTCGTTCCACTGAGCG
TCAGACCCCGTAGAAAAGATCAAAGGATCTTCTTGAGATCCTTTTTTTCTGCGCGTAATCTGCTGCTTGC
AAACAAAAAAACCACCGCTACCAGCGGTGGTTTGTTTGCCGGATCAAGAGCTACCAACTCTTTTTCCGAA
GGTAACTGGCTTCAGCAGAGCGCAGATACCAAATACTGTCCTTCTAGTGTAGCCGTAGTTAGGCCACCAC
TTCAAGAACTCTGTAGCACCGCCTACATACCTCGCTCTGCTAATCCTGTTACCAGTGGCTGCTGCCAGTG
GCGATAAGTCGTGTCTTACCGGGTTGGACTCAAGACGATAGTTACCGGATAAGGCGCAGCGGTCGGGCTG
AACGGGGGGTTCGTGCACACAGCCCAGCTTGGAGCGAACGACCTACACCGAACTGAGATACCTACAGCGT
GAGCTATGAGAAAGCGCCACGCTTCCCGAAGGGAGAAAGGCGGACAGGTATCCGGTAAGCGGCAGGGTCG
GAACAGGAGAGCGCACGAGGGAGCTTCCAGGGGGAAACGCCTGGTATCTTTATAGTCCTGTCGGGTTTCG

CCACCTCTGACTTGAGCGTCGATTTTTGTGATGCTCGTCAGGGGGGCGGAGCCTATGGAAAAACGCCAGC
AACGCGGCCTTTTTACGGTTCCTGGCCTTTTGCTGGCCTTTTGCTCACATGTTCTTTCCTGCGTTATCCC
CTGATTCTGTGGATAACCGTATTACCGCCTTTGAGTGAGCTGATACCGCTCGCCGCAGCCGAACGACCGA
GCGCAGCGAGTCAGTGAGCGAGGAAGCGGAAGAGCGCCTGATGCGGTATTTTCTCCTTACGCATCTGTGC
GGTATTTCACACCGCATATGGTGCACTCTCAGTACAATCTGCTCTGATGCCGCATAGTTAAGCCAGTATA
CACTCCGCTATCGCTACGTGACTGGGTCATGGCTGCGCCCCGACACCCGCCAACACCCGCTGACGCGCCC
TGACGGGCTTGTCTGCTCCCGGCATCCGCTTACAGACAAGCTGTGACCGTCTCCGGGAGCTGCATGTGTC
AGAGGTTTTCACCGTCATCACCGAAACGCGCGAGGCAGGGTGCCTTGATGTGGGCGCCGGCGGTCGAGTG
GCGACGGCGCGGCTTGTCCGCGCCCTGGTAGATTGCCTGGCCGTAGGCCAGCCATTTTTGAGCGGCCAGC
GGCCGCGATAGGCCGACGCGAAGCGGCGGGGCGTAGGGAGCGCAGCGACCGAAGGGTAGGCGCTTTTTGC
AGCTCTTCGGCTGTGCGCTGGCCAGACAGTTATGCACAGGCCAGGCGGGTTTTAAGAGTTTTAATAAGTT
TTAAAGAGTTTTAGGCGGAAAAATCGCCTTTTTTCTCTTTTATATCAGTCACTTACATGTGTGACCGGTT
CCCAATGTACGGCTTTGGGTTCCCAATGTACGGGTTCCGGTTCCCAATGTACGGCTTTGGGTTCCCAATG
TACGTGCTATCCACAGGAAAGAGACCTTTTCGACCTTTTTCCCCTGCTAGGGCAATTTGCCCTAGCATCT
GCTCCGTACATTAGGAACCGGCGGATGCTTCGCCCTCGATCAGGTTGCGGTAGCGCATGACTAGGATCGG
GCCAGCCTGCCCCGCCTCCTCCTTCAAATCGTACTCCGGCAGGTCATTTGACCCGATCAGCTTGCGCACG
GTGAAACAGAACTTCTTGAACTCTCCGGCGCTGCCACTGCGTTCGTAGATCGTCTTGAACAACCATCTGG
CTTCTGCCTTGCCTGCGGCGCGGCGTGCCAGGCGGTAGAGAAAACGGCCGATGCCGGGATCGATCAAAAA
GTAATCGGGGTGAACCGTCAGCACGTCCGGGTTCTTGCCTTCTGTGATCTCGCGGTACATCCAATCAGCT
AGCTCGATCTCGATGTACTCCGGCCGCCCGGTTTCGCTCTTTACGATCTTGTAGCGGCTAATCAAGGCTT
CACCCTCGGATACCGTCACCAGGCGGCCGTTCTTGGCCTTCTTCGTACGCTGCATGGCAACGTGCGTGGT
GTTTAACCGAATGCAGGTTTCTACCAGGTCGTCTTTCTGCTTTCCGCCATCGGCTCGCCGGCAGAACTTG
AGTACGTCCGCAACGTGTGGACGGAACACGCGGCCGGGCTTGTCTCCCTTCCCTTCCCGGTATCGGTTCA
TGGATTCGGTTAGATGGGAAACCGCCATCAGTACCAGGTCGTAATCCCACACACTGGCCATGCCGGCCGG
CCCTGCGGAAACCTCTACGTGCCCGTCTGGAAGCTCGTAGCGGATCACCTCGCCAGCTCGTCGGTCACGC
TTCGACAGACGGAAAACGGCCACGTCCATGATGCTGCGACTATCGCGGGTGCCCACGTCATAGAGCATCG
GAACGAAAAAATCTGGTTGCTCGTCGCCCTTGGGCGGCTTCCTAATCGACGGCGCACCGGCTGCCGGCGG
TTGCCGGGATTCTTTGCGGATTCGATCAGCGGCCGCTTGCCACGATTCACCGGGGCGTGCTTCTGCCTCG
ATGCGTTGCCGCTGGGCGGCCTGCGCGGCCTTCAACTTCTCCACCAGGTCATCACCCAGCGCCGCGCCGA
TTTGTACCGGGCCGGATGGTTTGCGACCGTCACGCCGATTCCTCGGGCTTGGGGGTTCCAGTGCCATTGC
AGGGCCGGCAGACAACCCAGCCGCTTACGCCTGGCCAACCGCCCGTTCCTCCACACATGGGGCATTCCAC
GGCGTCGGTGCCTGGTTGTTCTTGATTTTCCATGCCGCCTCCTTTAGCCGCTAAAATTCATCTACTCATT
TATTCATTTGCTCATTTACTCTGGTAGCTGCGCGATGTATTCAGATAGCAGCTCGGTAATGGTCTTGCCT
TGGCGTACCGCGTACATCTTCAGCTTGGTGTGATCCTCCGCCGGCAACTGAAAGTTGACCCGCTTCATGG
CTGGCGTGTCTGCCAGGCTGGCCAACGTTGCAGCCTTGCTGCTGCGTGCGCTCGGACGGCCGGCACTTAG
CGTGTTTGTGCTTTTGCTCATTTTCTCTTTACCTCATTAACTCAAATGAGTTTTGATTTAATTTCAGCGG
CCAGCGCCTGGACCTCGCGGGCAGCGTCGCCCTCGGGTTCTGATTCAAGAACGGTTGTGCCGGCGGCGGC
AGTGCCTGGGTAGCTCACGCGCTGCGTGATACGGGACTCAAGAATGGGCAGCTCGTACCCGGCCAGCGCC
TCGGCAACCTCACCGCCGATGCGCGTGCCTTTGATCGCCCGCGACACGACAAAGGCCGCTTGTAGCCTTC
CATCCGTGACCTCAATGCGCTGCTTAACCAGCTCCACCAGGTCGGCGGTGGCCCATATGTCGTAAGGGCT
TGGCTGCACCGGAATCAGCACGAAGTCGGCTGCCTTGATCGCGGACACAGCCAAGTCCGCCGCCTGGGGC
GCTCCGTCGATCACTACGAAGTCGCGCCGGCCGATGGCCTTCACGTCGCGGTCAATCGTCGGGCGGTCGA
TGCCGACAACGGTTAGCGGTTGATCTTCCCGCACGGCCGCCCAATCGCGGGCACTGCCCTGGGGATCGGA
ATCGACTAACAGAACATCGGCCCCGGCGAGTTGCAGGGCGCGGGCTAGATGGGTTGCGATGGTCGTCTTG

CCTGACCCGCCTTTCTGGTTAAGTACAGCGATAACCTTCATGCGTTCCCCTTGCGTATTTGTTTATTTAC
TCATCGCATCATATACGCAGCGACCGCATGACGCAAGCTGTTTTACTCAAATACACATCACCTTTTTAGA
CGGCGGCGCTCGGTTTCTTCAGCGGCCAAGCTGGCCGGCCAGGCCGCCAGCTTGGCATCAGACAAACCGG
CCAGGATTTCATGCAGCCGCACGGTTGAGACGTGCGCGGGCGGCTCGAACACGTACCCGGCCGCGATCAT
CTCCGCCTCGATCTCTTCGGTAATGAAAAACGGTTCGTCCTGGCCGTCCTGGTGCGGTTTCATGCTTGTT
CCTCTTGGCGTTCATTCTCGGCGGCCGCCAGGGCGTCGGCCTCGGTCAATGCGTCCTCACGGAAGGCACC
GCGCCGCCTGGCCTCGGTGGGCGTCACTTCCTCGCTGCGCTCAAGTGCGCGGTACAGGGTCGAGCGATGC
ACGCCAAGCAGTGCAGCCGCCTCTTTCACGGTGCGGCCTTCCTGGTCGATCAGCTCGCGGGCGTGCGCGA
TCTGTGCCGGGGTGAGGGTAGGGCGGGGGCCAAACTTCACGCCTCGGGCCTTGGCGGCCTCGCGCCCGCT
CCGGGTGCGGTCGATGATTAGGGAACGCTCGAACTCGGCAATGCCGGCGAACACGGTCAACACCATGCGG
CCGGCCGGCGTGGTGGTGTCGGCCCACGGCTCTGCCAGGCTACGCAGGCCCGCGCCGGCCTCCTGGATGC
GCTCGGCAATGTCCAGTAGGTCGCGGGTGCTGCGGGCCAGGCGGTCTAGCCTGGTCACTGTCACAACGTC
GCCAGGGCGTAGGTGGTCAAGCATCCTGGCCAGCTCCGGGCGGTCGCGCCTGGTGCCGGTGATCTTCTCG
GAAAACAGCTTGGTGCAGCCGGCCGCGTGCAGTTCGGCCCGTTGGTTGGTCAAGTCCTGGTCGTCGGTGC
TGACGCGGGCATAGCCCAGCAGGCCAGCGGCGGCGCTCTTGTTCATGGCGTAATGTCTCCGGTTCTAGTC
GCAAGTATTCTACTTTATGCGACTAAAACACGCGACAAGAAAACGCCAGGAAAAGGGCAGGGCGGCAGCC
TGTCGCGTAACTTAGGACTTGTGCGACATGTCGTTTTCAGAAGACGGCTGCACTGAACGTCAGAAGCCGA
CTGCACTATAGCAGCGGAGGGGTTGGATCAAAGTACTTTGATCCCGAGGGGAACCCTGTGGTTGGCATGC
ACATACAAATGGACGAACGGATAAACCTTTTCACGCCCTTTTAAATATCCGTTATTCTAATAAACGCTCT
TTTCTCTTAGGTTTACCCGCCAATATATCCTGTCAAACACTGATAGTTTAAACTGAAGGCGGGAAACGAC
AATCTGATCCAAGCTCAAGCTAAGCTTGAGCTCTGCATGCCTGCAGGTCCCCAGATTAGCCTTTTCAATT
TCAGAAAGAATGCTAACCCACAGATGGTTAGAGAGGCTTACGCAGCAGGTCTCATCAAGACGATCTACCC
GAGCAATAATCTCCAGGAAATCAAATACCTTCCCAAGAAGGTTAAAGATGCAGTCAAAAGATTCAGGACT
AACTGCATCAAGAACACAGAGAAAGATATATTTCTCAAGATCAGAAGTACTATTCCAGTATGGACGATTC
AAGGCTTGCTTCACAAACCAAGGCAAGTAATAGAGATTGGAGTCTCTAAAAAGGTAGTTCCCACTGAATC
AAAGGCCATGGAGTCAAAGATTCAAATAGAGGACCTAACAGAACTCGCCGTAAAGACTGGCGAACAGTTC
ATACAGAGTCTCTTACGACTCAATGACAAGAAGAAAATCTTCGTCAACATGGTGGAGCACGACACACTTG
TCTACTCCAAAAATATCAAAGATACAGTCTCAGAAGACCAAAGGGCAATTGAGACTTTTCAACAAAGGGT
AATATCCGGAAACCTCCTCGGATTCCATTGCCCAGCTATCTGTCACTTTATTGTGAAGATAGTGGAAAAG
GAAGGTGGCTCCTACAAATGCCATCATTGCGATAAAGGAAAGGCCATCGTTGAAGATGCCTCTGCCGACA
GTGGTCCCAAAGATGGACCCCCACCCACGAGGAGCATCGTGGAAAAAGAAGACGTTCCAACCACGTCTTC
AAAGCAAGTGGATTGATGTGATATCTCCACTGACGTAAGGGATGACGCACAATCCCACTATCCTTCGCAA
GACCCTTCCTCTATATAAGGAAGTTCATTTCATTTGGAGAGAACACGGGGGACTCTAGAGGATCCAAGGA
GATATAAGCCGCCACCATGGATTACAAGGACGATGATGACAAGGACTACAAGGACGATGACGATAAGGAC
TATAAGGATGACGATGACAAGGATTACGCCGAGGATGGATGATACTAGCGGACAAACTTTCGTTTCTGTG
AACCTTTCTGACGAAAGCAACACGGCAAGCACTAAAGTTGAAAACGTAAAATCGGAAGCGGATCGCTTGG
AGTTTTTACGTAAAATGAATCCCTTTATTGTTGACGCTCTGGTGCGGAAAACCAATTATCAGGGTGCTCG
CTTTCGCGCAAGAATAATAGGAGTGTGCGTGGATTGTGGTAGAAAACACGACAAGGCGCTCAAGACTGAA
CGTAAGTGTAAGGTCAACAACACGCAATCTCAGAACGAGGTGGCGCATATGTTGATGCACGATCCCGTTA
AGTATTTGAACAAAAGAAAGGCTAGAGCCTTTTCTAACGCAGAGATGTTTGCGATTGAACTGGTTTCGTA
CACCAAGGAAAGGCAATTGGCGGTCGATTTAGCCGCTGAAAGGGAGAAGACGAGACTGGCTCGTAGACAC
CCAATACGTTCTCCGGAAGAAACTCCGGAACATTATAAATTCGGTATGACTGCTAAGGCAATGTTAGCGG
ACATCAACGCCGTAGACGTTGGTGATAACGAGGAAACTTCGTCGGAGTACCCAGTGAGTCTGAGTGTTTC
TGGCGGAGTTCTCCGTGAACACCACTTCATCTGACCTAATGATATCCCGCGGCCATGCTAGAGTCCGCAA

AAATCACCAGTCTCTCTCTACAAATCTATCTCTCTCTATTTTTCTCCAGAATAATGTGTGAGTAGTTCCC
AGATAAGGGAATTAGGGTTCTTATAGGGTTTCGCTCATGTGTTGAGCATATAAGAAACCCTTAGTATGTA
TTTGTATTTGTAAAATACTTCTATCAATAAAATTTCTAATTCCTAAAACCAAAATCCAGTGACCTGCAGG
CATGCGACGTCG

Sequence of pH7LIC3.1.1-p23M18 (35S- p23M18)

GGGTACCCTCGAATTATCATACATGAGAATTAAGGGAGTCACGTTATGACCCCGCCGATGACGCGGGACA
AGCCGTTTTACGTTTGGAACTGACAGAACCGCAACGTTGAAGGAGCCACTGAGCCGCGGGTTTCTGGAGT
TTAATGAGCTAAGCACATACGTCAGAAACCATTATTGCGCGTTCAAAAGTCGCCTAAGGTCACTATCAGC
TAGCAAATATTTCTTGTCAAAAATGCTCCACTGACGTTCCATAAATTCCCCTCGGTATCCAATTAGAGTC
TCATATTCACTCTCAACTCGAATCCCCCCTATGAAAAAGCCTGAACTCACCGCGACGTCTGTCGAGAAGT
TTCTGATCGAAAAGTTCGACAGCGTCTCCGACCTGATGCAGCTCTCGGAGGGCGAAGAATCTCGTGCTTT
CAGCTTCGATGTAGGAGGGCGTGGATATGTCCTGCGGGTAAATAGCTGCGCCGATGGTTTCTACAAAGAT
CGTTATGTTTATCGGCACTTTGCATCGGCCGCGCTCCCGATTCCGGAAGTGCTTGACATTGGGGAGTTCA
GCGAGAGCCTGACCTATTGCATCTCCCGCCGTGCACAGGGTGTCACGTTGCAAGACCTGCCTGAAACCGA
ACTGCCCGCTGTTCTTCAGCCGGTCGCGGAGGCTATGGATGCTATCGCTGCGGCCGATCTTAGCCAGACG
AGCGGGTTCGGCCCATTCGGACCGCAAGGAATCGGTCAATACACTACATGGCGTGATTTCATATGCGCGA
TTGCTGATCCCCATGTGTATCACTGGCAAACTGTGATGGACGACACCGTCAGTGCGTCCGTCGCGCAGGC
TCTCGATGAGCTGATGCTTTGGGCGAGGACTGCCCCGAAGTCCGGCACTCGTGCACGCGGATTCGGCTCC
AACAATGTCCTGACGGACAATGGCCGCATAACAGCGGTCATTGACTGGAGCGAGGCGATGTTCGGGGATT
CCCAATACGAGGTCGCCAACATCTTCTTCTGGAGGCCGTGGTTGGCTTGTATGGAGCAGCAGACGCGCTA
CTTCGAGCGGAGGCATCCGGAGCTTGCAGGATCGCCAAGGCTCCGGGCGTATATGCTCCGCATTGGTCTT
GACCAACTCTATCAGAGCTTGGTTGACGGCAATTTCGATGATGCAGCTTGGGCGCAGGGTCGATGCGACG
CAATCGTCCGATCCGGAGCCGGGACTGTCGGGCGTACACAAATCGCCCGCAGAAGCGCGGCCGTCTGGAC
CGATGGCTGTGTAGAAGTACTCGCCGATAGTGGAAACCGACGCCCCAGCACTCGTCCGAGGGCAAAGGAA
TAGAGTAGATGCCGACCGAACAAGAGCTGATTTCGAGAACGCCTCAGCCAGCAACTCGCGCGAGCCTAGC
AAGGCAAATGCGAGAGAACGGCCTTACGCTTGGTGGCACAGTTCTCGTCCACAGTTCGCTAAGCTCGCTC
GGCTGGGTCGCGGGAGGGCCGGTCGCAGTGATTCAGGAATTAATTCCCTAGAGTCAAGCAGATCGTTCAA
ACATTTGGCAATAAAGTTTCTTAAGATTGAATCCTGTTGCCGGTCTTGCGATGATTATCATATAATTTCT
GTTGAATTACGTTAAGCATGTAATAATTAACATGTAATGCATGACGTTATTTATGAGATGGGTTTTTATG
ATTAGAGTCCCGCAATTATACATTTAATACGCGATAGAAAACAAAATATAGCGCGCAAACTAGGATAAAT
TATCGCGCGCGGTGTCATCTATGTTACTAGATCGACCGGCATGCAAGCTGATAATTCAATTCGGCGTTAA
TTCAGTACATTAAAAACGTCCGCAATGTGTTATTAAGTTGTCTAAGCGTCAATTTGTTTACACCACAATA
TATCCTGCCACCAGCCAGCCAACAGCTCCCCGACCGGCAGCTCGGCACAAAATCACCACTCGATACAGGC
AGCCCATCAGTCCGGGACGGCGTCAGCGGGAGAGCCGTTGTAAGGCGGCAGACTTTGCTCATGTTACCGA
TGCTATTCGGAAGAACGGCAACTAAGCTGCCGGGTTTGAAACACGGATGATCTCGCGGAGGGTAGCATGT
TGATTGTAACGATGACAGAGCGTTGCTGCCTGTGATCAATTCGGGCACGAACCCAGTGGACATAAGCCTC
GTTCGGTTCGTAAGCTGTAATGCAAGTAGCGTAACTGCCGTCACGCAACTGGTCCAGAACCTTGACCGAA
CGCAGCGGTGGTAACGGCGCAGTGGCGGTTTTCATGGCTTCTTGTTATGACATGTTTTTTTGGGGTACAG
TCTATGCCTCGGGCATCCAAGCAGCAAGCGCGTTACGCCGTGGGTCGATGTTTGATGTTATGGAGCAGCA
ACGATGTTACGCAGCAGGGCAGTCGCCCTAAAACAAAGTTAAACATCATGGGGGAAGCGGTGATCGCCGA
AGTATCGACTCAACTATCAGAGGTAGTTGGCGTCATCGAGCGCCATCTCGAACCGACGTTGCTGGCCGTA
CATTTGTACGGCTCCGCAGTGGATGGCGGCCTGAAGCCACACAGTGATATTGATTTGCTGGTTACGGTGA
CCGTAAGGCTTGATGAAACAACGCGGCGAGCTTTGATCAACGACCTTTTGGAAACTTCGGCTTCCCCTGG

AGAGAGCGAGATTCTCCGCGCTGTAGAAGTCACCATTGTTGTGCACGACGACATCATTCCGTGGCGTTAT
CCAGCTAAGCGCGAACTGCAATTTGGAGAATGGCAGCGCAATGACATTCTTGCAGGTATCTTCGAGCCAG
CCACGATCGACATTGATCTGGCTATCTTGCTGACAAAAGCAAGAGAACATAGCGTTGCCTTGGTAGGTCC
AGCGGCGGAGGAACTCTTTGATCCGGTTCCTGAACAGGATCTATTTGAGGCGCTAAATGAAACCTTAACG
CTATGGAACTCGCCGCCCGACTGGGCTGGCGATGAGCGAAATGTAGTGCTTACGTTGTCCCGCATTTGGT
ACAGCGCAGTAACCGGCAAAATCGCGCCGAAGGATGTCGCTGCCGACTGGGCAATGGAGCGCCTGCCGGC
CCAGTATCAGCCCGTCATACTTGAAGCTAGACAGGCTTATCTTGGACAAGAAGAAGATCGCTTGGCCTCG
CGCGCAGATCAGTTGGAAGAATTTGTCCACTACGTGAAAGGCGAGATCACCAAGGTAGTCGGCAAATAAT
GTCTAGCTAGAAATTCGTTCAAGCCGACGCCGCTTCGCCGGCGTTAACTCAAGCGATTAGATGCACTAAG
CACATAATTGCTCACAGCCAAACTATCAGGTCAAGTCTGCTTTTATTATTTTTAAGCGTGCATAATAAGC
CCTACACAAATTGGGAGATATATCATGCATGACCAAAATCCCTTAACGTGAGTTTTCGTTCCACTGAGCG
TCAGACCCCGTAGAAAAGATCAAAGGATCTTCTTGAGATCCTTTTTTTCTGCGCGTAATCTGCTGCTTGC
AAACAAAAAAACCACCGCTACCAGCGGTGGTTTGTTTGCCGGATCAAGAGCTACCAACTCTTTTTCCGAA
GGTAACTGGCTTCAGCAGAGCGCAGATACCAAATACTGTCCTTCTAGTGTAGCCGTAGTTAGGCCACCAC
TTCAAGAACTCTGTAGCACCGCCTACATACCTCGCTCTGCTAATCCTGTTACCAGTGGCTGCTGCCAGTG
GCGATAAGTCGTGTCTTACCGGGTTGGACTCAAGACGATAGTTACCGGATAAGGCGCAGCGGTCGGGCTG
AACGGGGGGTTCGTGCACACAGCCCAGCTTGGAGCGAACGACCTACACCGAACTGAGATACCTACAGCGT
GAGCTATGAGAAAGCGCCACGCTTCCCGAAGGGAGAAAGGCGGACAGGTATCCGGTAAGCGGCAGGGTCG
GAACAGGAGAGCGCACGAGGGAGCTTCCAGGGGGAAACGCCTGGTATCTTTATAGTCCTGTCGGGTTTCG
CCACCTCTGACTTGAGCGTCGATTTTTGTGATGCTCGTCAGGGGGGCGGAGCCTATGGAAAAACGCCAGC
AACGCGGCCTTTTTACGGTTCCTGGCCTTTTGCTGGCCTTTTGCTCACATGTTCTTTCCTGCGTTATCCC
CTGATTCTGTGGATAACCGTATTACCGCCTTTGAGTGAGCTGATACCGCTCGCCGCAGCCGAACGACCGA
GCGCAGCGAGTCAGTGAGCGAGGAAGCGGAAGAGCGCCTGATGCGGTATTTTCTCCTTACGCATCTGTGC
GGTATTTCACACCGCATATGGTGCACTCTCAGTACAATCTGCTCTGATGCCGCATAGTTAAGCCAGTATA
CACTCCGCTATCGCTACGTGACTGGGTCATGGCTGCGCCCCGACACCCGCCAACACCCGCTGACGCGCCC
TGACGGGCTTGTCTGCTCCCGGCATCCGCTTACAGACAAGCTGTGACCGTCTCCGGGAGCTGCATGTGTC
AGAGGTTTTCACCGTCATCACCGAAACGCGCGAGGCAGGGTGCCTTGATGTGGGCGCCGGCGGTCGAGTG
GCGACGGCGCGGCTTGTCCGCGCCCTGGTAGATTGCCTGGCCGTAGGCCAGCCATTTTTGAGCGGCCAGC
GGCCGCGATAGGCCGACGCGAAGCGGCGGGGCGTAGGGAGCGCAGCGACCGAAGGGTAGGCGCTTTTTGC
AGCTCTTCGGCTGTGCGCTGGCCAGACAGTTATGCACAGGCCAGGCGGGTTTTAAGAGTTTTAATAAGTT
TTAAAGAGTTTTAGGCGGAAAAATCGCCTTTTTTCTCTTTTATATCAGTCACTTACATGTGTGACCGGTT
CCCAATGTACGGCTTTGGGTTCCCAATGTACGGGTTCCGGTTCCCAATGTACGGCTTTGGGTTCCCAATG
TACGTGCTATCCACAGGAAAGAGACCTTTTCGACCTTTTTCCCCTGCTAGGGCAATTTGCCCTAGCATCT
GCTCCGTACATTAGGAACCGGCGGATGCTTCGCCCTCGATCAGGTTGCGGTAGCGCATGACTAGGATCGG
GCCAGCCTGCCCCGCCTCCTCCTTCAAATCGTACTCCGGCAGGTCATTTGACCCGATCAGCTTGCGCACG
GTGAAACAGAACTTCTTGAACTCTCCGGCGCTGCCACTGCGTTCGTAGATCGTCTTGAACAACCATCTGG
CTTCTGCCTTGCCTGCGGCGCGGCGTGCCAGGCGGTAGAGAAAACGGCCGATGCCGGGATCGATCAAAAA
GTAATCGGGGTGAACCGTCAGCACGTCCGGGTTCTTGCCTTCTGTGATCTCGCGGTACATCCAATCAGCT
AGCTCGATCTCGATGTACTCCGGCCGCCCGGTTTCGCTCTTTACGATCTTGTAGCGGCTAATCAAGGCTT
CACCCTCGGATACCGTCACCAGGCGGCCGTTCTTGGCCTTCTTCGTACGCTGCATGGCAACGTGCGTGGT
GTTTAACCGAATGCAGGTTTCTACCAGGTCGTCTTTCTGCTTTCCGCCATCGGCTCGCCGGCAGAACTTG
AGTACGTCCGCAACGTGTGGACGGAACACGCGGCCGGGCTTGTCTCCCTTCCCTTCCCGGTATCGGTTCA
TGGATTCGGTTAGATGGGAAACCGCCATCAGTACCAGGTCGTAATCCCACACACTGGCCATGCCGGCCGG
CCCTGCGGAAACCTCTACGTGCCCGTCTGGAAGCTCGTAGCGGATCACCTCGCCAGCTCGTCGGTCACGC

TTCGACAGACGGAAAACGGCCACGTCCATGATGCTGCGACTATCGCGGGTGCCCACGTCATAGAGCATCG
GAACGAAAAAATCTGGTTGCTCGTCGCCCTTGGGCGGCTTCCTAATCGACGGCGCACCGGCTGCCGGCGG
TTGCCGGGATTCTTTGCGGATTCGATCAGCGGCCGCTTGCCACGATTCACCGGGGCGTGCTTCTGCCTCG
ATGCGTTGCCGCTGGGCGGCCTGCGCGGCCTTCAACTTCTCCACCAGGTCATCACCCAGCGCCGCGCCGA
TTTGTACCGGGCCGGATGGTTTGCGACCGTCACGCCGATTCCTCGGGCTTGGGGGTTCCAGTGCCATTGC
AGGGCCGGCAGACAACCCAGCCGCTTACGCCTGGCCAACCGCCCGTTCCTCCACACATGGGGCATTCCAC
GGCGTCGGTGCCTGGTTGTTCTTGATTTTCCATGCCGCCTCCTTTAGCCGCTAAAATTCATCTACTCATT
TATTCATTTGCTCATTTACTCTGGTAGCTGCGCGATGTATTCAGATAGCAGCTCGGTAATGGTCTTGCCT
TGGCGTACCGCGTACATCTTCAGCTTGGTGTGATCCTCCGCCGGCAACTGAAAGTTGACCCGCTTCATGG
CTGGCGTGTCTGCCAGGCTGGCCAACGTTGCAGCCTTGCTGCTGCGTGCGCTCGGACGGCCGGCACTTAG
CGTGTTTGTGCTTTTGCTCATTTTCTCTTTACCTCATTAACTCAAATGAGTTTTGATTTAATTTCAGCGG
CCAGCGCCTGGACCTCGCGGGCAGCGTCGCCCTCGGGTTCTGATTCAAGAACGGTTGTGCCGGCGGCGGC
AGTGCCTGGGTAGCTCACGCGCTGCGTGATACGGGACTCAAGAATGGGCAGCTCGTACCCGGCCAGCGCC
TCGGCAACCTCACCGCCGATGCGCGTGCCTTTGATCGCCCGCGACACGACAAAGGCCGCTTGTAGCCTTC
CATCCGTGACCTCAATGCGCTGCTTAACCAGCTCCACCAGGTCGGCGGTGGCCCATATGTCGTAAGGGCT
TGGCTGCACCGGAATCAGCACGAAGTCGGCTGCCTTGATCGCGGACACAGCCAAGTCCGCCGCCTGGGGC
GCTCCGTCGATCACTACGAAGTCGCGCCGGCCGATGGCCTTCACGTCGCGGTCAATCGTCGGGCGGTCGA
TGCCGACAACGGTTAGCGGTTGATCTTCCCGCACGGCCGCCCAATCGCGGGCACTGCCCTGGGGATCGGA
ATCGACTAACAGAACATCGGCCCCGGCGAGTTGCAGGGCGCGGGCTAGATGGGTTGCGATGGTCGTCTTG
CCTGACCCGCCTTTCTGGTTAAGTACAGCGATAACCTTCATGCGTTCCCCTTGCGTATTTGTTTATTTAC
TCATCGCATCATATACGCAGCGACCGCATGACGCAAGCTGTTTTACTCAAATACACATCACCTTTTTAGA
CGGCGGCGCTCGGTTTCTTCAGCGGCCAAGCTGGCCGGCCAGGCCGCCAGCTTGGCATCAGACAAACCGG
CCAGGATTTCATGCAGCCGCACGGTTGAGACGTGCGCGGGCGGCTCGAACACGTACCCGGCCGCGATCAT
CTCCGCCTCGATCTCTTCGGTAATGAAAAACGGTTCGTCCTGGCCGTCCTGGTGCGGTTTCATGCTTGTT
CCTCTTGGCGTTCATTCTCGGCGGCCGCCAGGGCGTCGGCCTCGGTCAATGCGTCCTCACGGAAGGCACC
GCGCCGCCTGGCCTCGGTGGGCGTCACTTCCTCGCTGCGCTCAAGTGCGCGGTACAGGGTCGAGCGATGC
ACGCCAAGCAGTGCAGCCGCCTCTTTCACGGTGCGGCCTTCCTGGTCGATCAGCTCGCGGGCGTGCGCGA
TCTGTGCCGGGGTGAGGGTAGGGCGGGGGCCAAACTTCACGCCTCGGGCCTTGGCGGCCTCGCGCCCGCT
CCGGGTGCGGTCGATGATTAGGGAACGCTCGAACTCGGCAATGCCGGCGAACACGGTCAACACCATGCGG
CCGGCCGGCGTGGTGGTGTCGGCCCACGGCTCTGCCAGGCTACGCAGGCCCGCGCCGGCCTCCTGGATGC
GCTCGGCAATGTCCAGTAGGTCGCGGGTGCTGCGGGCCAGGCGGTCTAGCCTGGTCACTGTCACAACGTC
GCCAGGGCGTAGGTGGTCAAGCATCCTGGCCAGCTCCGGGCGGTCGCGCCTGGTGCCGGTGATCTTCTCG
GAAAACAGCTTGGTGCAGCCGGCCGCGTGCAGTTCGGCCCGTTGGTTGGTCAAGTCCTGGTCGTCGGTGC
TGACGCGGGCATAGCCCAGCAGGCCAGCGGCGGCGCTCTTGTTCATGGCGTAATGTCTCCGGTTCTAGTC
GCAAGTATTCTACTTTATGCGACTAAAACACGCGACAAGAAAACGCCAGGAAAAGGGCAGGGCGGCAGCC
TGTCGCGTAACTTAGGACTTGTGCGACATGTCGTTTTCAGAAGACGGCTGCACTGAACGTCAGAAGCCGA
CTGCACTATAGCAGCGGAGGGGTTGGATCAAAGTACTTTGATCCCGAGGGGAACCCTGTGGTTGGCATGC
ACATACAAATGGACGAACGGATAAACCTTTTCACGCCCTTTTAAATATCCGTTATTCTAATAAACGCTCT
TTTCTCTTAGGTTTACCCGCCAATATATCCTGTCAAACACTGATAGTTTAAACTGAAGGCGGGAAACGAC
AATCTGATCCAAGCTCAAGCTAAGCTTGAGCTCTGCATGCCTGCAGGTCCCCAGATTAGCCTTTTCAATT
TCAGAAAGAATGCTAACCCACAGATGGTTAGAGAGGCTTACGCAGCAGGTCTCATCAAGACGATCTACCC
GAGCAATAATCTCCAGGAAATCAAATACCTTCCCAAGAAGGTTAAAGATGCAGTCAAAAGATTCAGGACT
AACTGCATCAAGAACACAGAGAAAGATATATTTCTCAAGATCAGAAGTACTATTCCAGTATGGACGATTC
AAGGCTTGCTTCACAAACCAAGGCAAGTAATAGAGATTGGAGTCTCTAAAAAGGTAGTTCCCACTGAATC

AAAGGCCATGGAGTCAAAGATTCAAATAGAGGACCTAACAGAACTCGCCGTAAAGACTGGCGAACAGTTC
ATACAGAGTCTCTTACGACTCAATGACAAGAAGAAAATCTTCGTCAACATGGTGGAGCACGACACACTTG
TCTACTCCAAAAATATCAAAGATACAGTCTCAGAAGACCAAAGGGCAATTGAGACTTTTCAACAAAGGGT
AATATCCGGAAACCTCCTCGGATTCCATTGCCCAGCTATCTGTCACTTTATTGTGAAGATAGTGGAAAAG
GAAGGTGGCTCCTACAAATGCCATCATTGCGATAAAGGAAAGGCCATCGTTGAAGATGCCTCTGCCGACA
GTGGTCCCAAAGATGGACCCCCACCCACGAGGAGCATCGTGGAAAAAGAAGACGTTCCAACCACGTCTTC
AAAGCAAGTGGATTGATGTGATATCTCCACTGACGTAAGGGATGACGCACAATCCCACTATCCTTCGCAA
GACCCTTCCTCTATATAAGGAAGTTCATTTCATTTGGAGAGAACACGGGGGACTCTAGAGGATCCAAGGA
GATATAAGCCGCCACCATGGATTACAAGGACGATGATGACAAGGACTACAAGGACGATGACGATAAGGAC
TATAAGGATGACGATGACAAGGATTACGCCGAGGATGGATGATACTAGCGGACAAACTTTCGTTTCTGTG
AACCTTTCTGACGAAAGCAACACGGCAAGCACTAAAGTTGAAAACGTAAAATCGGAAGCGGATCGCTTGG
AGTTTTTACGTAAAATGAATCCCTTTATTGTTGACGCTCTGGTGCGGAAAACCAATTATCAGGGTGCTCG
CTTTCGCGCAAGAATAATAGGAGTGTGCGTGGATTGTGGTAGAAAACACGACAAGGCGCTCAAGACTGAA
CGTAAGTGTAAGGTCAACAACACGCAATCTCAGAACGAGGTGGCGCATATGTTGATGCACGATCCCGTTA
AGTATTTGAACAAAAGAAAGGCTAGAGCCTTTTCTAACGCAGAGATGTTTGCGATTGAACTGGTTTCGTA
CACCAAGGAAAGGCAATTGGCGGTCGATTTAGCCGCTGAAAGGGAGAAGACGAGACTGGCTCGTAGACAC
CCAATACGTTCTCCGGAAGAAACTCCGGAACATTATAAATTCGGTATGACTGCTAAGGCAATGTTACCGG
ACATCAACGCCGTAGACGCGGGTGATAACGAGGAAACTTCGTCGGAGTACCCAGTGAGTCTGAGTGTTTC
TGGCGGAGTTCTCCGTGAACACCACTTCATCTGACCTAATGATATCCCGCGGCCATGCTAGAGTCCGCAA
AAATCACCAGTCTCTCTCTACAAATCTATCTCTCTCTATTTTTCTCCAGAATAATGTGTGAGTAGTTCCC
AGATAAGGGAATTAGGGTTCTTATAGGGTTTCGCTCATGTGTTGAGCATATAAGAAACCCTTAGTATGTA
TTTGTATTTGTAAAATACTTCTATCAATAAAATTTCTAATTCCTAAAACCAAAATCCAGTGACCTGCAGG
CATGCGACGTCG

Supplemental Data 3. Related sequences of N4p23 subcellular localization expression vector

Sequence of pH7LIC5.1.1 (35S-GFP-CCDB)

GGGTACCCTCGAATTATCATACATGAGAATTAAGGGAGTCACGTTATGACCCCGCCGATGACGCGGGACA
AGCCGTTTTACGTTTGGAACTGACAGAACCGCAACGTTGAAGGAGCCACTGAGCCGCGGGTTTCTGGAGT
TTAATGAGCTAAGCACATACGTCAGAAACCATTATTGCGCGTTCAAAAGTCGCCTAAGGTCACTATCAGC
TAGCAAATATTTCTTGTCAAAAATGCTCCACTGACGTTCCATAAATTCCCCTCGGTATCCAATTAGAGTC
TCATATTCACTCTCAACTCGAATCCCCCCTATGAAAAAGCCTGAACTCACCGCGACGTCTGTCGAGAAGT
TTCTGATCGAAAAGTTCGACAGCGTCTCCGACCTGATGCAGCTCTCGGAGGGCGAAGAATCTCGTGCTTT
CAGCTTCGATGTAGGAGGGCGTGGATATGTCCTGCGGGTAAATAGCTGCGCCGATGGTTTCTACAAAGAT
CGTTATGTTTATCGGCACTTTGCATCGGCCGCGCTCCCGATTCCGGAAGTGCTTGACATTGGGGAGTTCA
GCGAGAGCCTGACCTATTGCATCTCCCGCCGTGCACAGGGTGTCACGTTGCAAGACCTGCCTGAAACCGA
ACTGCCCGCTGTTCTTCAGCCGGTCGCGGAGGCTATGGATGCTATCGCTGCGGCCGATCTTAGCCAGACG
AGCGGGTTCGGCCCATTCGGACCGCAAGGAATCGGTCAATACACTACATGGCGTGATTTCATATGCGCGA
TTGCTGATCCCCATGTGTATCACTGGCAAACTGTGATGGACGACACCGTCAGTGCGTCCGTCGCGCAGGC
TCTCGATGAGCTGATGCTTTGGGCGAGGACTGCCCCGAAGTCCGGCACTCGTGCACGCGGATTCGGCTCC
AACAATGTCCTGACGGACAATGGCCGCATAACAGCGGTCATTGACTGGAGCGAGGCGATGTTCGGGGATT
CCCAATACGAGGTCGCCAACATCTTCTTCTGGAGGCCGTGGTTGGCTTGTATGGAGCAGCAGACGCGCTA
CTTCGAGCGGAGGCATCCGGAGCTTGCAGGATCGCCAAGGCTCCGGGCGTATATGCTCCGCATTGGTCTT
GACCAACTCTATCAGAGCTTGGTTGACGGCAATTTCGATGATGCAGCTTGGGCGCAGGGTCGATGCGACG
CAATCGTCCGATCCGGAGCCGGGACTGTCGGGCGTACACAAATCGCCCGCAGAAGCGCGGCCGTCTGGAC
CGATGGCTGTGTAGAAGTACTCGCCGATAGTGGAAACCGACGCCCCAGCACTCGTCCGAGGGCAAAGGAA
TAGAGTAGATGCCGACCGAACAAGAGCTGATTTCGAGAACGCCTCAGCCAGCAACTCGCGCGAGCCTAGC
AAGGCAAATGCGAGAGAACGGCCTTACGCTTGGTGGCACAGTTCTCGTCCACAGTTCGCTAAGCTCGCTC
GGCTGGGTCGCGGGAGGGCCGGTCGCAGTGATTCAGGAATTAATTCCCTAGAGTCAAGCAGATCGTTCAA
ACATTTGGCAATAAAGTTTCTTAAGATTGAATCCTGTTGCCGGTCTTGCGATGATTATCATATAATTTCT
GTTGAATTACGTTAAGCATGTAATAATTAACATGTAATGCATGACGTTATTTATGAGATGGGTTTTTATG
ATTAGAGTCCCGCAATTATACATTTAATACGCGATAGAAAACAAAATATAGCGCGCAAACTAGGATAAAT
TATCGCGCGCGGTGTCATCTATGTTACTAGATCGACCGGCATGCAAGCTGATAATTCAATTCGGCGTTAA
TTCAGTACATTAAAAACGTCCGCAATGTGTTATTAAGTTGTCTAAGCGTCAATTTGTTTACACCACAATA
TATCCTGCCACCAGCCAGCCAACAGCTCCCCGACCGGCAGCTCGGCACAAAATCACCACTCGATACAGGC
AGCCCATCAGTCCGGGACGGCGTCAGCGGGAGAGCCGTTGTAAGGCGGCAGACTTTGCTCATGTTACCGA
TGCTATTCGGAAGAACGGCAACTAAGCTGCCGGGTTTGAAACACGGATGATCTCGCGGAGGGTAGCATGT
TGATTGTAACGATGACAGAGCGTTGCTGCCTGTGATCAATTCGGGCACGAACCCAGTGGACATAAGCCTC
GTTCGGTTCGTAAGCTGTAATGCAAGTAGCGTAACTGCCGTCACGCAACTGGTCCAGAACCTTGACCGAA
CGCAGCGGTGGTAACGGCGCAGTGGCGGTTTTCATGGCTTCTTGTTATGACATGTTTTTTTGGGGTACAG
TCTATGCCTCGGGCATCCAAGCAGCAAGCGCGTTACGCCGTGGGTCGATGTTTGATGTTATGGAGCAGCA
ACGATGTTACGCAGCAGGGCAGTCGCCCTAAAACAAAGTTAAACATCATGGGGGAAGCGGTGATCGCCGA
AGTATCGACTCAACTATCAGAGGTAGTTGGCGTCATCGAGCGCCATCTCGAACCGACGTTGCTGGCCGTA
CATTTGTACGGCTCCGCAGTGGATGGCGGCCTGAAGCCACACAGTGATATTGATTTGCTGGTTACGGTGA
CCGTAAGGCTTGATGAAACAACGCGGCGAGCTTTGATCAACGACCTTTTGGAAACTTCGGCTTCCCCTGG
AGAGAGCGAGATTCTCCGCGCTGTAGAAGTCACCATTGTTGTGCACGACGACATCATTCCGTGGCGTTAT
CCAGCTAAGCGCGAACTGCAATTTGGAGAATGGCAGCGCAATGACATTCTTGCAGGTATCTTCGAGCCAG

CCACGATCGACATTGATCTGGCTATCTTGCTGACAAAAGCAAGAGAACATAGCGTTGCCTTGGTAGGTCC
AGCGGCGGAGGAACTCTTTGATCCGGTTCCTGAACAGGATCTATTTGAGGCGCTAAATGAAACCTTAACG
CTATGGAACTCGCCGCCCGACTGGGCTGGCGATGAGCGAAATGTAGTGCTTACGTTGTCCCGCATTTGGT
ACAGCGCAGTAACCGGCAAAATCGCGCCGAAGGATGTCGCTGCCGACTGGGCAATGGAGCGCCTGCCGGC
CCAGTATCAGCCCGTCATACTTGAAGCTAGACAGGCTTATCTTGGACAAGAAGAAGATCGCTTGGCCTCG
CGCGCAGATCAGTTGGAAGAATTTGTCCACTACGTGAAAGGCGAGATCACCAAGGTAGTCGGCAAATAAT
GTCTAGCTAGAAATTCGTTCAAGCCGACGCCGCTTCGCCGGCGTTAACTCAAGCGATTAGATGCACTAAG
CACATAATTGCTCACAGCCAAACTATCAGGTCAAGTCTGCTTTTATTATTTTTAAGCGTGCATAATAAGC
CCTACACAAATTGGGAGATATATCATGCATGACCAAAATCCCTTAACGTGAGTTTTCGTTCCACTGAGCG
TCAGACCCCGTAGAAAAGATCAAAGGATCTTCTTGAGATCCTTTTTTTCTGCGCGTAATCTGCTGCTTGC
AAACAAAAAAACCACCGCTACCAGCGGTGGTTTGTTTGCCGGATCAAGAGCTACCAACTCTTTTTCCGAA
GGTAACTGGCTTCAGCAGAGCGCAGATACCAAATACTGTCCTTCTAGTGTAGCCGTAGTTAGGCCACCAC
TTCAAGAACTCTGTAGCACCGCCTACATACCTCGCTCTGCTAATCCTGTTACCAGTGGCTGCTGCCAGTG
GCGATAAGTCGTGTCTTACCGGGTTGGACTCAAGACGATAGTTACCGGATAAGGCGCAGCGGTCGGGCTG
AACGGGGGGTTCGTGCACACAGCCCAGCTTGGAGCGAACGACCTACACCGAACTGAGATACCTACAGCGT
GAGCTATGAGAAAGCGCCACGCTTCCCGAAGGGAGAAAGGCGGACAGGTATCCGGTAAGCGGCAGGGTCG
GAACAGGAGAGCGCACGAGGGAGCTTCCAGGGGGAAACGCCTGGTATCTTTATAGTCCTGTCGGGTTTCG
CCACCTCTGACTTGAGCGTCGATTTTTGTGATGCTCGTCAGGGGGGCGGAGCCTATGGAAAAACGCCAGC
AACGCGGCCTTTTTACGGTTCCTGGCCTTTTGCTGGCCTTTTGCTCACATGTTCTTTCCTGCGTTATCCC
CTGATTCTGTGGATAACCGTATTACCGCCTTTGAGTGAGCTGATACCGCTCGCCGCAGCCGAACGACCGA
GCGCAGCGAGTCAGTGAGCGAGGAAGCGGAAGAGCGCCTGATGCGGTATTTTCTCCTTACGCATCTGTGC
GGTATTTCACACCGCATATGGTGCACTCTCAGTACAATCTGCTCTGATGCCGCATAGTTAAGCCAGTATA
CACTCCGCTATCGCTACGTGACTGGGTCATGGCTGCGCCCCGACACCCGCCAACACCCGCTGACGCGCCC
TGACGGGCTTGTCTGCTCCCGGCATCCGCTTACAGACAAGCTGTGACCGTCTCCGGGAGCTGCATGTGTC
AGAGGTTTTCACCGTCATCACCGAAACGCGCGAGGCAGGGTGCCTTGATGTGGGCGCCGGCGGTCGAGTG
GCGACGGCGCGGCTTGTCCGCGCCCTGGTAGATTGCCTGGCCGTAGGCCAGCCATTTTTGAGCGGCCAGC
GGCCGCGATAGGCCGACGCGAAGCGGCGGGGCGTAGGGAGCGCAGCGACCGAAGGGTAGGCGCTTTTTGC
AGCTCTTCGGCTGTGCGCTGGCCAGACAGTTATGCACAGGCCAGGCGGGTTTTAAGAGTTTTAATAAGTT
TTAAAGAGTTTTAGGCGGAAAAATCGCCTTTTTTCTCTTTTATATCAGTCACTTACATGTGTGACCGGTT
CCCAATGTACGGCTTTGGGTTCCCAATGTACGGGTTCCGGTTCCCAATGTACGGCTTTGGGTTCCCAATG
TACGTGCTATCCACAGGAAAGAGACCTTTTCGACCTTTTTCCCCTGCTAGGGCAATTTGCCCTAGCATCT
GCTCCGTACATTAGGAACCGGCGGATGCTTCGCCCTCGATCAGGTTGCGGTAGCGCATGACTAGGATCGG
GCCAGCCTGCCCCGCCTCCTCCTTCAAATCGTACTCCGGCAGGTCATTTGACCCGATCAGCTTGCGCACG
GTGAAACAGAACTTCTTGAACTCTCCGGCGCTGCCACTGCGTTCGTAGATCGTCTTGAACAACCATCTGG
CTTCTGCCTTGCCTGCGGCGCGGCGTGCCAGGCGGTAGAGAAAACGGCCGATGCCGGGATCGATCAAAAA
GTAATCGGGGTGAACCGTCAGCACGTCCGGGTTCTTGCCTTCTGTGATCTCGCGGTACATCCAATCAGCT
AGCTCGATCTCGATGTACTCCGGCCGCCCGGTTTCGCTCTTTACGATCTTGTAGCGGCTAATCAAGGCTT
CACCCTCGGATACCGTCACCAGGCGGCCGTTCTTGGCCTTCTTCGTACGCTGCATGGCAACGTGCGTGGT
GTTTAACCGAATGCAGGTTTCTACCAGGTCGTCTTTCTGCTTTCCGCCATCGGCTCGCCGGCAGAACTTG
AGTACGTCCGCAACGTGTGGACGGAACACGCGGCCGGGCTTGTCTCCCTTCCCTTCCCGGTATCGGTTCA
TGGATTCGGTTAGATGGGAAACCGCCATCAGTACCAGGTCGTAATCCCACACACTGGCCATGCCGGCCGG
CCCTGCGGAAACCTCTACGTGCCCGTCTGGAAGCTCGTAGCGGATCACCTCGCCAGCTCGTCGGTCACGC
TTCGACAGACGGAAAACGGCCACGTCCATGATGCTGCGACTATCGCGGGTGCCCACGTCATAGAGCATCG
GAACGAAAAAATCTGGTTGCTCGTCGCCCTTGGGCGGCTTCCTAATCGACGGCGCACCGGCTGCCGGCGG

TTGCCGGGATTCTTTGCGGATTCGATCAGCGGCCGCTTGCCACGATTCACCGGGGCGTGCTTCTGCCTCG
ATGCGTTGCCGCTGGGCGGCCTGCGCGGCCTTCAACTTCTCCACCAGGTCATCACCCAGCGCCGCGCCGA
TTTGTACCGGGCCGGATGGTTTGCGACCGTCACGCCGATTCCTCGGGCTTGGGGGTTCCAGTGCCATTGC
AGGGCCGGCAGACAACCCAGCCGCTTACGCCTGGCCAACCGCCCGTTCCTCCACACATGGGGCATTCCAC
GGCGTCGGTGCCTGGTTGTTCTTGATTTTCCATGCCGCCTCCTTTAGCCGCTAAAATTCATCTACTCATT
TATTCATTTGCTCATTTACTCTGGTAGCTGCGCGATGTATTCAGATAGCAGCTCGGTAATGGTCTTGCCT
TGGCGTACCGCGTACATCTTCAGCTTGGTGTGATCCTCCGCCGGCAACTGAAAGTTGACCCGCTTCATGG
CTGGCGTGTCTGCCAGGCTGGCCAACGTTGCAGCCTTGCTGCTGCGTGCGCTCGGACGGCCGGCACTTAG
CGTGTTTGTGCTTTTGCTCATTTTCTCTTTACCTCATTAACTCAAATGAGTTTTGATTTAATTTCAGCGG
CCAGCGCCTGGACCTCGCGGGCAGCGTCGCCCTCGGGTTCTGATTCAAGAACGGTTGTGCCGGCGGCGGC
AGTGCCTGGGTAGCTCACGCGCTGCGTGATACGGGACTCAAGAATGGGCAGCTCGTACCCGGCCAGCGCC
TCGGCAACCTCACCGCCGATGCGCGTGCCTTTGATCGCCCGCGACACGACAAAGGCCGCTTGTAGCCTTC
CATCCGTGACCTCAATGCGCTGCTTAACCAGCTCCACCAGGTCGGCGGTGGCCCATATGTCGTAAGGGCT
TGGCTGCACCGGAATCAGCACGAAGTCGGCTGCCTTGATCGCGGACACAGCCAAGTCCGCCGCCTGGGGC
GCTCCGTCGATCACTACGAAGTCGCGCCGGCCGATGGCCTTCACGTCGCGGTCAATCGTCGGGCGGTCGA
TGCCGACAACGGTTAGCGGTTGATCTTCCCGCACGGCCGCCCAATCGCGGGCACTGCCCTGGGGATCGGA
ATCGACTAACAGAACATCGGCCCCGGCGAGTTGCAGGGCGCGGGCTAGATGGGTTGCGATGGTCGTCTTG
CCTGACCCGCCTTTCTGGTTAAGTACAGCGATAACCTTCATGCGTTCCCCTTGCGTATTTGTTTATTTAC
TCATCGCATCATATACGCAGCGACCGCATGACGCAAGCTGTTTTACTCAAATACACATCACCTTTTTAGA
CGGCGGCGCTCGGTTTCTTCAGCGGCCAAGCTGGCCGGCCAGGCCGCCAGCTTGGCATCAGACAAACCGG
CCAGGATTTCATGCAGCCGCACGGTTGAGACGTGCGCGGGCGGCTCGAACACGTACCCGGCCGCGATCAT
CTCCGCCTCGATCTCTTCGGTAATGAAAAACGGTTCGTCCTGGCCGTCCTGGTGCGGTTTCATGCTTGTT
CCTCTTGGCGTTCATTCTCGGCGGCCGCCAGGGCGTCGGCCTCGGTCAATGCGTCCTCACGGAAGGCACC
GCGCCGCCTGGCCTCGGTGGGCGTCACTTCCTCGCTGCGCTCAAGTGCGCGGTACAGGGTCGAGCGATGC
ACGCCAAGCAGTGCAGCCGCCTCTTTCACGGTGCGGCCTTCCTGGTCGATCAGCTCGCGGGCGTGCGCGA
TCTGTGCCGGGGTGAGGGTAGGGCGGGGGCCAAACTTCACGCCTCGGGCCTTGGCGGCCTCGCGCCCGCT
CCGGGTGCGGTCGATGATTAGGGAACGCTCGAACTCGGCAATGCCGGCGAACACGGTCAACACCATGCGG
CCGGCCGGCGTGGTGGTGTCGGCCCACGGCTCTGCCAGGCTACGCAGGCCCGCGCCGGCCTCCTGGATGC
GCTCGGCAATGTCCAGTAGGTCGCGGGTGCTGCGGGCCAGGCGGTCTAGCCTGGTCACTGTCACAACGTC
GCCAGGGCGTAGGTGGTCAAGCATCCTGGCCAGCTCCGGGCGGTCGCGCCTGGTGCCGGTGATCTTCTCG
GAAAACAGCTTGGTGCAGCCGGCCGCGTGCAGTTCGGCCCGTTGGTTGGTCAAGTCCTGGTCGTCGGTGC
TGACGCGGGCATAGCCCAGCAGGCCAGCGGCGGCGCTCTTGTTCATGGCGTAATGTCTCCGGTTCTAGTC
GCAAGTATTCTACTTTATGCGACTAAAACACGCGACAAGAAAACGCCAGGAAAAGGGCAGGGCGGCAGCC
TGTCGCGTAACTTAGGACTTGTGCGACATGTCGTTTTCAGAAGACGGCTGCACTGAACGTCAGAAGCCGA
CTGCACTATAGCAGCGGAGGGGTTGGATCAAAGTACTTTGATCCCGAGGGGAACCCTGTGGTTGGCATGC
ACATACAAATGGACGAACGGATAAACCTTTTCACGCCCTTTTAAATATCCGTTATTCTAATAAACGCTCT
TTTCTCTTAGGTTTACCCGCCAATATATCCTGTCAAACACTGATAGTTTAAACTGAAGGCGGGAAACGAC
AATCTGATCCAAGCTCAAGCTAAGCTTGAGCTCTGCATGCCTGCAGGTCCCCAGATTAGCCTTTTCAATT
TCAGAAAGAATGCTAACCCACAGATGGTTAGAGAGGCTTACGCAGCAGGTCTCATCAAGACGATCTACCC
GAGCAATAATCTCCAGGAAATCAAATACCTTCCCAAGAAGGTTAAAGATGCAGTCAAAAGATTCAGGACT
AACTGCATCAAGAACACAGAGAAAGATATATTTCTCAAGATCAGAAGTACTATTCCAGTATGGACGATTC
AAGGCTTGCTTCACAAACCAAGGCAAGTAATAGAGATTGGAGTCTCTAAAAAGGTAGTTCCCACTGAATC
AAAGGCCATGGAGTCAAAGATTCAAATAGAGGACCTAACAGAACTCGCCGTAAAGACTGGCGAACAGTTC
ATACAGAGTCTCTTACGACTCAATGACAAGAAGAAAATCTTCGTCAACATGGTGGAGCACGACACACTTG

TCTACTCCAAAAATATCAAAGATACAGTCTCAGAAGACCAAAGGGCAATTGAGACTTTTCAACAAAGGGT
AATATCCGGAAACCTCCTCGGATTCCATTGCCCAGCTATCTGTCACTTTATTGTGAAGATAGTGGAAAAG
GAAGGTGGCTCCTACAAATGCCATCATTGCGATAAAGGAAAGGCCATCGTTGAAGATGCCTCTGCCGACA
GTGGTCCCAAAGATGGACCCCCACCCACGAGGAGCATCGTGGAAAAAGAAGACGTTCCAACCACGTCTTC
AAAGCAAGTGGATTGATGTGATATCTCCACTGACGTAAGGGATGACGCACAATCCCACTATCCTTCGCAA
GACCCTTCCTCTATATAAGGAAGTTCATTTCATTTGGAGAGAACACGGGGGACTCTAGAGGATCCAAGGA
GATATAAGCCGCCACCATGGTGAGCAAGGGCGAGGAGCTGTTCACCGGGGTGGTGCCCATCCTGGTCGAG
CTGGACGGCGACGTAAACGGCCACAAGTTCAGCGTGTCCGGCGAGGGCGAGGGCGATGCCACCTACGGCA
AGCTGACCCTGAAGTTCATCTGCACCACCGGCAAGCTGCCCGTGCCCTGGCCCACCCTCGTGACCACCCT
GACCTACGGCGTGCAGTGCTTCAGCCGCTACCCCGACCACATGAAGCAGCACGACTTCTTCAAGTCCGCC
ATGCCCGAAGGCTACGTCCAGGAGCGCACCATCTTCTTCAAGGACGACGGCAACTACAAGACCCGCGCCG
AGGTGAAGTTCGAGGGCGACACCCTGGTGAACCGCATCGAGCTGAAGGGCATCGACTTCAAGGAGGACGG
CAACATCCTGGGGCACAAGCTGGAGTACAACTACAACAGCCACAACGTCTATATCATGGCCGACAAGCAG
AAGAACGGCATCAAGGTGAACTTCAAGATCCGCCACAACATCGAGGACGGCAGCGTGCAGCTCGCCGACC
ACTACCAGCAGAACACCCCCATCGGCGACGGCCCCGTGCTGCTGCCCGACAACCACTACCTGAGCACCCA
GTCCGCCCTGAGCAAAGACCCCAACGAGAAGCGCGATCACATGGTCCTGCTGGAGTTCGTGACCGCCGCC
GGGATCACTCTCGGCATGGACGAGCTGTACAAGGATTACGCCGAGGCCTGTCGACCTGCAGACTGGCTGT
GTATAAGGGAGCCTGACATTTATATTCCCCAGAACATCAGGTTAATGGCGTTTTTGATGTCATTTTCGCG
GTGGCTGAGATCAGCCACTTCTTCCCCGATAACGGAGACCGGCACACTGGCCATATCGGTGGTCATCATG
CGCCAGCTTTCATCCCCGATATGCACCACCGGGTAAAGTTCACGGGAGACTTTATCTGACAGCAGACGTG
CACTGGCCAGGGGGATCACCATCCGTCGCCCGGGCGTGTCAATAATATCACTCTGTACATCCACAAACAG
ACGATAACGGCTCTCTCTTTTATAGGTGTAAACCTTAAACTGCATTTCACCAGCCCCTGTTCTCGTCAGC
AAAAGAGCCGTTCATTTCAATAAACCGGGCGACCTCAGCCATCCCTTCCTGATTTTCCGCTTTCCAGCGT
TCGGCACGCAGACGACGGGCTTCATTCTGCATGGTTGTGCTTACCAGACCGGAGATATTGACATCATATA
TGCCTTGAGCAACTGATAGCTGTCGCTGTCAACTGTCACTGTAATACGCTGCTTCATAGCATACCTCTTT
TTGACATACTTCGGGTATACATATCAGTATATATTCTTATACCGCAAAAATCAGCGCGCAAATACGCATA
CTGTTATCTGGCTTTTAGTAAGCCGGATCCACGCGTTTACGCCCCGCCCTGCCACTCATCGCAGTACTGT
TGTAATTCATTAAGCATTCTGCCGACATGGAAGCCATCACAGACGGCATGATGAACCTGAATCGCCAGCG
GCATCAGCACCTTGTCGCCTTGCGTATAATATTTGCCCATGGTGAAAACGGGGGCGAAGAAGTTGTCCAT
ATTGGCCACGTTTAAATCAAAACTGGTGAAACTCACCCAGGGATTGGCTGAGACGAAAAACATATTCTCA
ATAAACCCTTTAGGGAAATAGGCCAGGTTTTCACCGTAACACGCCACATCTTGCGAATATATGTGTAGAA
ACTGCCGGAAATCGTCGTGGTATTCACTCCAGAGCGATGAAAACGTTTCAGTTTGCTCATGGAAAACGGT
GTAACAAGGGTGAACACTATCCCATATCACCAGCTCACCGTCTTTCATTGCCATACGGAATTCCGGATGA
GCATTCATCAGGCGGGCAAGAATGTGAATAAAGGCCGGATAAAACTTGTGCTTATTTTTCTTTACGGTCT
TTAAAAAGGCCGTAATATCCAGCTGAACGGTCTGGTTATAGGTACATTGAGCAACTGACTGAAATGCCTC
AAAATGTTCTTTACGATGCCATTGGGATATATCAACGGTGGTATATCCAGTGATTTTTTTCTCCATTTTA
GCTTCCTTAGCTCCTGAAAATCTCGCCGGATCCTAACTCAAAATCCACACATTATACGAGCCGGAAGCAT
AAAGTGTAAAGCCTGGGGTGCCTAATGCGGCCGCAGGCCTCTTCCCTAATGATATCCCGCGGCCATGCTA
GAGTCCGCAAAAATCACCAGTCTCTCTCTACAAATCTATCTCTCTCTATTTTTCTCCAGAATAATGTGTG
AGTAGTTCCCAGATAAGGGAATTAGGGTTCTTATAGGGTTTCGCTCATGTGTTGAGCATATAAGAAACCC
TTAGTATGTATTTGTATTTGTAAAATACTTCTATCAATAAAATTTCTAATTCCTAAAACCAAAATCCAGT
GACCTGCAGGCATGCGACGTCG

Sequence of pH7LIC5.1.1-C2b (35S-GFP-C2b)

GGGTACCCTCGAATTATCATACATGAGAATTAAGGGAGTCACGTTATGACCCCGCCGATGACGCGGGACA
AGCCGTTTTACGTTTGGAACTGACAGAACCGCAACGTTGAAGGAGCCACTGAGCCGCGGGTTTCTGGAGT
TTAATGAGCTAAGCACATACGTCAGAAACCATTATTGCGCGTTCAAAAGTCGCCTAAGGTCACTATCAGC
TAGCAAATATTTCTTGTCAAAAATGCTCCACTGACGTTCCATAAATTCCCCTCGGTATCCAATTAGAGTC
TCATATTCACTCTCAACTCGAATCCCCCCTATGAAAAAGCCTGAACTCACCGCGACGTCTGTCGAGAAGT
TTCTGATCGAAAAGTTCGACAGCGTCTCCGACCTGATGCAGCTCTCGGAGGGCGAAGAATCTCGTGCTTT
CAGCTTCGATGTAGGAGGGCGTGGATATGTCCTGCGGGTAAATAGCTGCGCCGATGGTTTCTACAAAGAT
CGTTATGTTTATCGGCACTTTGCATCGGCCGCGCTCCCGATTCCGGAAGTGCTTGACATTGGGGAGTTCA
GCGAGAGCCTGACCTATTGCATCTCCCGCCGTGCACAGGGTGTCACGTTGCAAGACCTGCCTGAAACCGA
ACTGCCCGCTGTTCTTCAGCCGGTCGCGGAGGCTATGGATGCTATCGCTGCGGCCGATCTTAGCCAGACG
AGCGGGTTCGGCCCATTCGGACCGCAAGGAATCGGTCAATACACTACATGGCGTGATTTCATATGCGCGA
TTGCTGATCCCCATGTGTATCACTGGCAAACTGTGATGGACGACACCGTCAGTGCGTCCGTCGCGCAGGC
TCTCGATGAGCTGATGCTTTGGGCGAGGACTGCCCCGAAGTCCGGCACTCGTGCACGCGGATTCGGCTCC
AACAATGTCCTGACGGACAATGGCCGCATAACAGCGGTCATTGACTGGAGCGAGGCGATGTTCGGGGATT
CCCAATACGAGGTCGCCAACATCTTCTTCTGGAGGCCGTGGTTGGCTTGTATGGAGCAGCAGACGCGCTA
CTTCGAGCGGAGGCATCCGGAGCTTGCAGGATCGCCAAGGCTCCGGGCGTATATGCTCCGCATTGGTCTT
GACCAACTCTATCAGAGCTTGGTTGACGGCAATTTCGATGATGCAGCTTGGGCGCAGGGTCGATGCGACG
CAATCGTCCGATCCGGAGCCGGGACTGTCGGGCGTACACAAATCGCCCGCAGAAGCGCGGCCGTCTGGAC
CGATGGCTGTGTAGAAGTACTCGCCGATAGTGGAAACCGACGCCCCAGCACTCGTCCGAGGGCAAAGGAA
TAGAGTAGATGCCGACCGAACAAGAGCTGATTTCGAGAACGCCTCAGCCAGCAACTCGCGCGAGCCTAGC
AAGGCAAATGCGAGAGAACGGCCTTACGCTTGGTGGCACAGTTCTCGTCCACAGTTCGCTAAGCTCGCTC
GGCTGGGTCGCGGGAGGGCCGGTCGCAGTGATTCAGGAATTAATTCCCTAGAGTCAAGCAGATCGTTCAA
ACATTTGGCAATAAAGTTTCTTAAGATTGAATCCTGTTGCCGGTCTTGCGATGATTATCATATAATTTCT
GTTGAATTACGTTAAGCATGTAATAATTAACATGTAATGCATGACGTTATTTATGAGATGGGTTTTTATG
ATTAGAGTCCCGCAATTATACATTTAATACGCGATAGAAAACAAAATATAGCGCGCAAACTAGGATAAAT
TATCGCGCGCGGTGTCATCTATGTTACTAGATCGACCGGCATGCAAGCTGATAATTCAATTCGGCGTTAA
TTCAGTACATTAAAAACGTCCGCAATGTGTTATTAAGTTGTCTAAGCGTCAATTTGTTTACACCACAATA
TATCCTGCCACCAGCCAGCCAACAGCTCCCCGACCGGCAGCTCGGCACAAAATCACCACTCGATACAGGC
AGCCCATCAGTCCGGGACGGCGTCAGCGGGAGAGCCGTTGTAAGGCGGCAGACTTTGCTCATGTTACCGA
TGCTATTCGGAAGAACGGCAACTAAGCTGCCGGGTTTGAAACACGGATGATCTCGCGGAGGGTAGCATGT
TGATTGTAACGATGACAGAGCGTTGCTGCCTGTGATCAATTCGGGCACGAACCCAGTGGACATAAGCCTC
GTTCGGTTCGTAAGCTGTAATGCAAGTAGCGTAACTGCCGTCACGCAACTGGTCCAGAACCTTGACCGAA
CGCAGCGGTGGTAACGGCGCAGTGGCGGTTTTCATGGCTTCTTGTTATGACATGTTTTTTTGGGGTACAG
TCTATGCCTCGGGCATCCAAGCAGCAAGCGCGTTACGCCGTGGGTCGATGTTTGATGTTATGGAGCAGCA
ACGATGTTACGCAGCAGGGCAGTCGCCCTAAAACAAAGTTAAACATCATGGGGGAAGCGGTGATCGCCGA
AGTATCGACTCAACTATCAGAGGTAGTTGGCGTCATCGAGCGCCATCTCGAACCGACGTTGCTGGCCGTA
CATTTGTACGGCTCCGCAGTGGATGGCGGCCTGAAGCCACACAGTGATATTGATTTGCTGGTTACGGTGA
CCGTAAGGCTTGATGAAACAACGCGGCGAGCTTTGATCAACGACCTTTTGGAAACTTCGGCTTCCCCTGG
AGAGAGCGAGATTCTCCGCGCTGTAGAAGTCACCATTGTTGTGCACGACGACATCATTCCGTGGCGTTAT
CCAGCTAAGCGCGAACTGCAATTTGGAGAATGGCAGCGCAATGACATTCTTGCAGGTATCTTCGAGCCAG
CCACGATCGACATTGATCTGGCTATCTTGCTGACAAAAGCAAGAGAACATAGCGTTGCCTTGGTAGGTCC
AGCGGCGGAGGAACTCTTTGATCCGGTTCCTGAACAGGATCTATTTGAGGCGCTAAATGAAACCTTAACG
CTATGGAACTCGCCGCCCGACTGGGCTGGCGATGAGCGAAATGTAGTGCTTACGTTGTCCCGCATTTGGT
ACAGCGCAGTAACCGGCAAAATCGCGCCGAAGGATGTCGCTGCCGACTGGGCAATGGAGCGCCTGCCGGC

CCAGTATCAGCCCGTCATACTTGAAGCTAGACAGGCTTATCTTGGACAAGAAGAAGATCGCTTGGCCTCG
CGCGCAGATCAGTTGGAAGAATTTGTCCACTACGTGAAAGGCGAGATCACCAAGGTAGTCGGCAAATAAT
GTCTAGCTAGAAATTCGTTCAAGCCGACGCCGCTTCGCCGGCGTTAACTCAAGCGATTAGATGCACTAAG
CACATAATTGCTCACAGCCAAACTATCAGGTCAAGTCTGCTTTTATTATTTTTAAGCGTGCATAATAAGC
CCTACACAAATTGGGAGATATATCATGCATGACCAAAATCCCTTAACGTGAGTTTTCGTTCCACTGAGCG
TCAGACCCCGTAGAAAAGATCAAAGGATCTTCTTGAGATCCTTTTTTTCTGCGCGTAATCTGCTGCTTGC
AAACAAAAAAACCACCGCTACCAGCGGTGGTTTGTTTGCCGGATCAAGAGCTACCAACTCTTTTTCCGAA
GGTAACTGGCTTCAGCAGAGCGCAGATACCAAATACTGTCCTTCTAGTGTAGCCGTAGTTAGGCCACCAC
TTCAAGAACTCTGTAGCACCGCCTACATACCTCGCTCTGCTAATCCTGTTACCAGTGGCTGCTGCCAGTG
GCGATAAGTCGTGTCTTACCGGGTTGGACTCAAGACGATAGTTACCGGATAAGGCGCAGCGGTCGGGCTG
AACGGGGGGTTCGTGCACACAGCCCAGCTTGGAGCGAACGACCTACACCGAACTGAGATACCTACAGCGT
GAGCTATGAGAAAGCGCCACGCTTCCCGAAGGGAGAAAGGCGGACAGGTATCCGGTAAGCGGCAGGGTCG
GAACAGGAGAGCGCACGAGGGAGCTTCCAGGGGGAAACGCCTGGTATCTTTATAGTCCTGTCGGGTTTCG
CCACCTCTGACTTGAGCGTCGATTTTTGTGATGCTCGTCAGGGGGGCGGAGCCTATGGAAAAACGCCAGC
AACGCGGCCTTTTTACGGTTCCTGGCCTTTTGCTGGCCTTTTGCTCACATGTTCTTTCCTGCGTTATCCC
CTGATTCTGTGGATAACCGTATTACCGCCTTTGAGTGAGCTGATACCGCTCGCCGCAGCCGAACGACCGA
GCGCAGCGAGTCAGTGAGCGAGGAAGCGGAAGAGCGCCTGATGCGGTATTTTCTCCTTACGCATCTGTGC
GGTATTTCACACCGCATATGGTGCACTCTCAGTACAATCTGCTCTGATGCCGCATAGTTAAGCCAGTATA
CACTCCGCTATCGCTACGTGACTGGGTCATGGCTGCGCCCCGACACCCGCCAACACCCGCTGACGCGCCC
TGACGGGCTTGTCTGCTCCCGGCATCCGCTTACAGACAAGCTGTGACCGTCTCCGGGAGCTGCATGTGTC
AGAGGTTTTCACCGTCATCACCGAAACGCGCGAGGCAGGGTGCCTTGATGTGGGCGCCGGCGGTCGAGTG
GCGACGGCGCGGCTTGTCCGCGCCCTGGTAGATTGCCTGGCCGTAGGCCAGCCATTTTTGAGCGGCCAGC
GGCCGCGATAGGCCGACGCGAAGCGGCGGGGCGTAGGGAGCGCAGCGACCGAAGGGTAGGCGCTTTTTGC
AGCTCTTCGGCTGTGCGCTGGCCAGACAGTTATGCACAGGCCAGGCGGGTTTTAAGAGTTTTAATAAGTT
TTAAAGAGTTTTAGGCGGAAAAATCGCCTTTTTTCTCTTTTATATCAGTCACTTACATGTGTGACCGGTT
CCCAATGTACGGCTTTGGGTTCCCAATGTACGGGTTCCGGTTCCCAATGTACGGCTTTGGGTTCCCAATG
TACGTGCTATCCACAGGAAAGAGACCTTTTCGACCTTTTTCCCCTGCTAGGGCAATTTGCCCTAGCATCT
GCTCCGTACATTAGGAACCGGCGGATGCTTCGCCCTCGATCAGGTTGCGGTAGCGCATGACTAGGATCGG
GCCAGCCTGCCCCGCCTCCTCCTTCAAATCGTACTCCGGCAGGTCATTTGACCCGATCAGCTTGCGCACG
GTGAAACAGAACTTCTTGAACTCTCCGGCGCTGCCACTGCGTTCGTAGATCGTCTTGAACAACCATCTGG
CTTCTGCCTTGCCTGCGGCGCGGCGTGCCAGGCGGTAGAGAAAACGGCCGATGCCGGGATCGATCAAAAA
GTAATCGGGGTGAACCGTCAGCACGTCCGGGTTCTTGCCTTCTGTGATCTCGCGGTACATCCAATCAGCT
AGCTCGATCTCGATGTACTCCGGCCGCCCGGTTTCGCTCTTTACGATCTTGTAGCGGCTAATCAAGGCTT
CACCCTCGGATACCGTCACCAGGCGGCCGTTCTTGGCCTTCTTCGTACGCTGCATGGCAACGTGCGTGGT
GTTTAACCGAATGCAGGTTTCTACCAGGTCGTCTTTCTGCTTTCCGCCATCGGCTCGCCGGCAGAACTTG
AGTACGTCCGCAACGTGTGGACGGAACACGCGGCCGGGCTTGTCTCCCTTCCCTTCCCGGTATCGGTTCA
TGGATTCGGTTAGATGGGAAACCGCCATCAGTACCAGGTCGTAATCCCACACACTGGCCATGCCGGCCGG
CCCTGCGGAAACCTCTACGTGCCCGTCTGGAAGCTCGTAGCGGATCACCTCGCCAGCTCGTCGGTCACGC
TTCGACAGACGGAAAACGGCCACGTCCATGATGCTGCGACTATCGCGGGTGCCCACGTCATAGAGCATCG
GAACGAAAAAATCTGGTTGCTCGTCGCCCTTGGGCGGCTTCCTAATCGACGGCGCACCGGCTGCCGGCGG
TTGCCGGGATTCTTTGCGGATTCGATCAGCGGCCGCTTGCCACGATTCACCGGGGCGTGCTTCTGCCTCG
ATGCGTTGCCGCTGGGCGGCCTGCGCGGCCTTCAACTTCTCCACCAGGTCATCACCCAGCGCCGCGCCGA
TTTGTACCGGGCCGGATGGTTTGCGACCGTCACGCCGATTCCTCGGGCTTGGGGGTTCCAGTGCCATTGC
AGGGCCGGCAGACAACCCAGCCGCTTACGCCTGGCCAACCGCCCGTTCCTCCACACATGGGGCATTCCAC

GGCGTCGGTGCCTGGTTGTTCTTGATTTTCCATGCCGCCTCCTTTAGCCGCTAAAATTCATCTACTCATT
TATTCATTTGCTCATTTACTCTGGTAGCTGCGCGATGTATTCAGATAGCAGCTCGGTAATGGTCTTGCCT
TGGCGTACCGCGTACATCTTCAGCTTGGTGTGATCCTCCGCCGGCAACTGAAAGTTGACCCGCTTCATGG
CTGGCGTGTCTGCCAGGCTGGCCAACGTTGCAGCCTTGCTGCTGCGTGCGCTCGGACGGCCGGCACTTAG
CGTGTTTGTGCTTTTGCTCATTTTCTCTTTACCTCATTAACTCAAATGAGTTTTGATTTAATTTCAGCGG
CCAGCGCCTGGACCTCGCGGGCAGCGTCGCCCTCGGGTTCTGATTCAAGAACGGTTGTGCCGGCGGCGGC
AGTGCCTGGGTAGCTCACGCGCTGCGTGATACGGGACTCAAGAATGGGCAGCTCGTACCCGGCCAGCGCC
TCGGCAACCTCACCGCCGATGCGCGTGCCTTTGATCGCCCGCGACACGACAAAGGCCGCTTGTAGCCTTC
CATCCGTGACCTCAATGCGCTGCTTAACCAGCTCCACCAGGTCGGCGGTGGCCCATATGTCGTAAGGGCT
TGGCTGCACCGGAATCAGCACGAAGTCGGCTGCCTTGATCGCGGACACAGCCAAGTCCGCCGCCTGGGGC
GCTCCGTCGATCACTACGAAGTCGCGCCGGCCGATGGCCTTCACGTCGCGGTCAATCGTCGGGCGGTCGA
TGCCGACAACGGTTAGCGGTTGATCTTCCCGCACGGCCGCCCAATCGCGGGCACTGCCCTGGGGATCGGA
ATCGACTAACAGAACATCGGCCCCGGCGAGTTGCAGGGCGCGGGCTAGATGGGTTGCGATGGTCGTCTTG
CCTGACCCGCCTTTCTGGTTAAGTACAGCGATAACCTTCATGCGTTCCCCTTGCGTATTTGTTTATTTAC
TCATCGCATCATATACGCAGCGACCGCATGACGCAAGCTGTTTTACTCAAATACACATCACCTTTTTAGA
CGGCGGCGCTCGGTTTCTTCAGCGGCCAAGCTGGCCGGCCAGGCCGCCAGCTTGGCATCAGACAAACCGG
CCAGGATTTCATGCAGCCGCACGGTTGAGACGTGCGCGGGCGGCTCGAACACGTACCCGGCCGCGATCAT
CTCCGCCTCGATCTCTTCGGTAATGAAAAACGGTTCGTCCTGGCCGTCCTGGTGCGGTTTCATGCTTGTT
CCTCTTGGCGTTCATTCTCGGCGGCCGCCAGGGCGTCGGCCTCGGTCAATGCGTCCTCACGGAAGGCACC
GCGCCGCCTGGCCTCGGTGGGCGTCACTTCCTCGCTGCGCTCAAGTGCGCGGTACAGGGTCGAGCGATGC
ACGCCAAGCAGTGCAGCCGCCTCTTTCACGGTGCGGCCTTCCTGGTCGATCAGCTCGCGGGCGTGCGCGA
TCTGTGCCGGGGTGAGGGTAGGGCGGGGGCCAAACTTCACGCCTCGGGCCTTGGCGGCCTCGCGCCCGCT
CCGGGTGCGGTCGATGATTAGGGAACGCTCGAACTCGGCAATGCCGGCGAACACGGTCAACACCATGCGG
CCGGCCGGCGTGGTGGTGTCGGCCCACGGCTCTGCCAGGCTACGCAGGCCCGCGCCGGCCTCCTGGATGC
GCTCGGCAATGTCCAGTAGGTCGCGGGTGCTGCGGGCCAGGCGGTCTAGCCTGGTCACTGTCACAACGTC
GCCAGGGCGTAGGTGGTCAAGCATCCTGGCCAGCTCCGGGCGGTCGCGCCTGGTGCCGGTGATCTTCTCG
GAAAACAGCTTGGTGCAGCCGGCCGCGTGCAGTTCGGCCCGTTGGTTGGTCAAGTCCTGGTCGTCGGTGC
TGACGCGGGCATAGCCCAGCAGGCCAGCGGCGGCGCTCTTGTTCATGGCGTAATGTCTCCGGTTCTAGTC
GCAAGTATTCTACTTTATGCGACTAAAACACGCGACAAGAAAACGCCAGGAAAAGGGCAGGGCGGCAGCC
TGTCGCGTAACTTAGGACTTGTGCGACATGTCGTTTTCAGAAGACGGCTGCACTGAACGTCAGAAGCCGA
CTGCACTATAGCAGCGGAGGGGTTGGATCAAAGTACTTTGATCCCGAGGGGAACCCTGTGGTTGGCATGC
ACATACAAATGGACGAACGGATAAACCTTTTCACGCCCTTTTAAATATCCGTTATTCTAATAAACGCTCT
TTTCTCTTAGGTTTACCCGCCAATATATCCTGTCAAACACTGATAGTTTAAACTGAAGGCGGGAAACGAC
AATCTGATCCAAGCTCAAGCTAAGCTTGAGCTCTGCATGCCTGCAGGTCCCCAGATTAGCCTTTTCAATT
TCAGAAAGAATGCTAACCCACAGATGGTTAGAGAGGCTTACGCAGCAGGTCTCATCAAGACGATCTACCC
GAGCAATAATCTCCAGGAAATCAAATACCTTCCCAAGAAGGTTAAAGATGCAGTCAAAAGATTCAGGACT
AACTGCATCAAGAACACAGAGAAAGATATATTTCTCAAGATCAGAAGTACTATTCCAGTATGGACGATTC
AAGGCTTGCTTCACAAACCAAGGCAAGTAATAGAGATTGGAGTCTCTAAAAAGGTAGTTCCCACTGAATC
AAAGGCCATGGAGTCAAAGATTCAAATAGAGGACCTAACAGAACTCGCCGTAAAGACTGGCGAACAGTTC
ATACAGAGTCTCTTACGACTCAATGACAAGAAGAAAATCTTCGTCAACATGGTGGAGCACGACACACTTG
TCTACTCCAAAAATATCAAAGATACAGTCTCAGAAGACCAAAGGGCAATTGAGACTTTTCAACAAAGGGT
AATATCCGGAAACCTCCTCGGATTCCATTGCCCAGCTATCTGTCACTTTATTGTGAAGATAGTGGAAAAG
GAAGGTGGCTCCTACAAATGCCATCATTGCGATAAAGGAAAGGCCATCGTTGAAGATGCCTCTGCCGACA
GTGGTCCCAAAGATGGACCCCCACCCACGAGGAGCATCGTGGAAAAAGAAGACGTTCCAACCACGTCTTC

AAAGCAAGTGGATTGATGTGATATCTCCACTGACGTAAGGGATGACGCACAATCCCACTATCCTTCGCAA
GACCCTTCCTCTATATAAGGAAGTTCATTTCATTTGGAGAGAACACGGGGGACTCTAGAGGATCCAAGGA
GATATAAGCCGCCACCATGGTGAGCAAGGGCGAGGAGCTGTTCACCGGGGTGGTGCCCATCCTGGTCGAG
CTGGACGGCGACGTAAACGGCCACAAGTTCAGCGTGTCCGGCGAGGGCGAGGGCGATGCCACCTACGGCA
AGCTGACCCTGAAGTTCATCTGCACCACCGGCAAGCTGCCCGTGCCCTGGCCCACCCTCGTGACCACCCT
GACCTACGGCGTGCAGTGCTTCAGCCGCTACCCCGACCACATGAAGCAGCACGACTTCTTCAAGTCCGCC
ATGCCCGAAGGCTACGTCCAGGAGCGCACCATCTTCTTCAAGGACGACGGCAACTACAAGACCCGCGCCG
AGGTGAAGTTCGAGGGCGACACCCTGGTGAACCGCATCGAGCTGAAGGGCATCGACTTCAAGGAGGACGG
CAACATCCTGGGGCACAAGCTGGAGTACAACTACAACAGCCACAACGTCTATATCATGGCCGACAAGCAG
AAGAACGGCATCAAGGTGAACTTCAAGATCCGCCACAACATCGAGGACGGCAGCGTGCAGCTCGCCGACC
ACTACCAGCAGAACACCCCCATCGGCGACGGCCCCGTGCTGCTGCCCGACAACCACTACCTGAGCACCCA
GTCCGCCCTGAGCAAAGACCCCAACGAGAAGCGCGATCACATGGTCCTGCTGGAGTTCGTGACCGCCGCC
GGGATCACTCTCGGCATGGACGAGCTGTACAAGGATTACGCCGAGGTCATGGATGTGTTGACAGTAGTGG
TGTCGACCGCCGACCTCCACTTAGCCAATTTGCAGGAGGTGAAACGTCGAAGACGAAGGTCTCACGTCAG
AAACCGGCGAGCGAGGGGTTACAAAAGTCCCAGCGAGAGAGCGCGATCTATAGCGAGACTTTTCCAGATG
TTACCATTCCACGGAGTAGATCCCGTGGATTGGTTTCCTGATGTCGTTCGCTCTCCGTCCGTTACCAGCC
TTGTTTCTTATGAATCTTTTGATGATACTGATTGGTTTGCTGGTAACGAATGGGCCGAAGGGTCGTTTTG
ACCTCTTCCCTAATGATATCCCGCGGCCATGCTAGAGTCCGCAAAAATCACCAGTCTCTCTCTACAAATC
TATCTCTCTCTATTTTTCTCCAGAATAATGTGTGAGTAGTTCCCAGATAAGGGAATTAGGGTTCTTATAG
GGTTTCGCTCATGTGTTGAGCATATAAGAAACCCTTAGTATGTATTTGTATTTGTAAAATACTTCTATCA
ATAAAATTTCTAATTCCTAAAACCAAAATCCAGTGACCTGCAGGCATGCGACGTCG

Sequence of pH7LIC5.1.1-N4p23 (35S-GFP-N4p23)

GGGTACCCTCGAATTATCATACATGAGAATTAAGGGAGTCACGTTATGACCCCCGCCGATGACGCGGGACA
AGCCGTTTTACGTTTGGAACTGACAGAACCGCAACGTTGAAGGAGCCACTGAGCCGCGGGTTTCTGGAGT
TTAATGAGCTAAGCACATACGTCAGAAACCATTATTGCGCGTTCAAAAGTCGCCTAAGGTCACTATCAGC
TAGCAAATATTTCTTGTCAAAAATGCTCCACTGACGTTCCATAAATTCCCCTCGGTATCCAATTAGAGTC
TCATATTCACTCTCAACTCGAATCCCCCCTATGAAAAAGCCTGAACTCACCGCGACGTCTGTCGAGAAGT
TTCTGATCGAAAAGTTCGACAGCGTCTCCGACCTGATGCAGCTCTCGGAGGGCGAAGAATCTCGTGCTTT
CAGCTTCGATGTAGGAGGGCGTGGATATGTCCTGCGGGTAAATAGCTGCGCCGATGGTTTCTACAAAGAT
CGTTATGTTTATCGGCACTTTGCATCGGCCGCGCTCCCGATTCCGGAAGTGCTTGACATTGGGGAGTTCA
GCGAGAGCCTGACCTATTGCATCTCCCGCCGTGCACAGGGTGTCACGTTGCAAGACCTGCCTGAAACCGA
ACTGCCCGCTGTTCTTCAGCCGGTCGCGGAGGCTATGGATGCTATCGCTGCGGCCGATCTTAGCCAGACG
AGCGGGTTCGGCCCATTCGGACCGCAAGGAATCGGTCAATACACTACATGGCGTGATTTCATATGCGCGA
TTGCTGATCCCCATGTGTATCACTGGCAAACTGTGATGGACGACACCGTCAGTGCGTCCGTCGCGCAGGC
TCTCGATGAGCTGATGCTTTGGGCCGAGGACTGCCCCGAAGTCCGGCACCTCGTGCACGCGGATTCGGCTCC
AACAATGTCCTGACGGACAATGGCCGCATAACAGCGGTCATTGACTGGAGCGAGGCGATGTTCGGGGATT
CCCAATACGAGGTCGCCAACATCTTCTTCTGGAGGCCGTGGTTGGCTTGTATGGAGCAGCAGACGCGCTA
CTTCGAGCGGAGGCATCCGGAGCTTGCAGGATCGCCAAGGCTCCGGGCGTATATGCTCCGCATTGGTCTT
GACCAACTCTATCAGAGCTTGGTTGACGGCAATTTCGATGATGCAGCTTGGGCGCAGGGTCGATGCGACG
CAATCGTCCGATCCGGAGCCGGGACTGTCGGGCGTACACAAATCGCCCGCAGAAGCGCGGCCGTCTGGAC
CGATGGCTGTGTAGAAGTACTCGCCGATAGTGGAAACCGACGCCCCAGCACTCGTCCGAGGGCAAAGGAA
TAGAGTAGATGCCGACCGAACAAGAGCTGATTTCGAGAACGCCTCAGCCAGCAACTCGCGCGAGCCTAGC
AAGGCAAATGCGAGAGAACGGCCTTACGCTTGGTGGCACAGTTCTCGTCCACAGTTCGCTAAGCTCGCTC

GGCTGGGTCGCGGGAGGGCCGGTCGCAGTGATTCAGGAATTAATTCCCTAGAGTCAAGCAGATCGTTCAA
ACATTTGGCAATAAAGTTTCTTAAGATTGAATCCTGTTGCCGGTCTTGCGATGATTATCATATAATTTCT
GTTGAATTACGTTAAGCATGTAATAATTAACATGTAATGCATGACGTTATTTATGAGATGGGTTTTTATG
ATTAGAGTCCCGCAATTATACATTTAATACGCGATAGAAAACAAAATATAGCGCGCAAACTAGGATAAAT
TATCGCGCGCGGTGTCATCTATGTTACTAGATCGACCGGCATGCAAGCTGATAATTCAATTCGGCGTTAA
TTCAGTACATTAAAAACGTCCGCAATGTGTTATTAAGTTGTCTAAGCGTCAATTTGTTTACACCACAATA
TATCCTGCCACCAGCCAGCCAACAGCTCCCCGACCGGCAGCTCGGCACAAAATCACCACTCGATACAGGC
AGCCCATCAGTCCGGGACGGCGTCAGCGGGAGAGCCGTTGTAAGGCGGCAGACTTTGCTCATGTTACCGA
TGCTATTCGGAAGAACGGCAACTAAGCTGCCGGGTTTGAAACACGGATGATCTCGCGGAGGGTAGCATGT
TGATTGTAACGATGACAGAGCGTTGCTGCCTGTGATCAATTCGGGCACGAACCCAGTGGACATAAGCCTC
GTTCGGTTCGTAAGCTGTAATGCAAGTAGCGTAACTGCCGTCACGCAACTGGTCCAGAACCTTGACCGAA
CGCAGCGGTGGTAACGGCGCAGTGGCGGTTTTCATGGCTTCTTGTTATGACATGTTTTTTTGGGGTACAG
TCTATGCCTCGGGCATCCAAGCAGCAAGCGCGTTACGCCGTGGGTCGATGTTTGATGTTATGGAGCAGCA
ACGATGTTACGCAGCAGGGCAGTCGCCCTAAAACAAAGTTAAACATCATGGGGGAAGCGGTGATCGCCGA
AGTATCGACTCAACTATCAGAGGTAGTTGGCGTCATCGAGCGCCATCTCGAACCGACGTTGCTGGCCGTA
CATTTGTACGGCTCCGCAGTGGATGGCGGCCTGAAGCCACACAGTGATATTGATTTGCTGGTTACGGTGA
CCGTAAGGCTTGATGAAACAACGCGGCGAGCTTTGATCAACGACCTTTTGGAAACTTCGGCTTCCCCTGG
AGAGAGCGAGATTCTCCGCGCTGTAGAAGTCACCATTGTTGTGCACGACGACATCATTCCGTGGCGTTAT
CCAGCTAAGCGCGAACTGCAATTTGGAGAATGGCAGCGCAATGACATTCTTGCAGGTATCTTCGAGCCAG
CCACGATCGACATTGATCTGGCTATCTTGCTGACAAAAGCAAGAGAACATAGCGTTGCCTTGGTAGGTCC
AGCGGCGGAGGAACTCTTTGATCCGGTTCCTGAACAGGATCTATTTGAGGCGCTAAATGAAACCTTAACG
CTATGGAACTCGCCGCCCGACTGGGCTGGCGATGAGCGAAATGTAGTGCTTACGTTGTCCCGCATTTGGT
ACAGCGCAGTAACCGGCAAAATCGCGCCGAAGGATGTCGCTGCCGACTGGGCAATGGAGCGCCTGCCGGC
CCAGTATCAGCCCGTCATACTTGAAGCTAGACAGGCTTATCTTGGACAAGAAGAAGATCGCTTGGCCTCG
CGCGCAGATCAGTTGGAAGAATTTGTCCACTACGTGAAAGGCGAGATCACCAAGGTAGTCGGCAAATAAT
GTCTAGCTAGAAATTCGTTCAAGCCGACGCCGCTTCGCCGGCGTTAACTCAAGCGATTAGATGCACTAAG
CACATAATTGCTCACAGCCAAACTATCAGGTCAAGTCTGCTTTTATTATTTTTAAGCGTGCATAATAAGC
CCTACACAAATTGGGAGATATATCATGCATGACCAAAATCCCTTAACGTGAGTTTTCGTTCCACTGAGCG
TCAGACCCCGTAGAAAAGATCAAAGGATCTTCTTGAGATCCTTTTTTTCTGCGCGTAATCTGCTGCTTGC
AAACAAAAAAACCACCGCTACCAGCGGTGGTTTGTTTGCCGGATCAAGAGCTACCAACTCTTTTTCCGAA
GGTAACTGGCTTCAGCAGAGCGCAGATACCAAATACTGTCCTTCTAGTGTAGCCGTAGTTAGGCCACCAC
TTCAAGAACTCTGTAGCACCGCCTACATACCTCGCTCTGCTAATCCTGTTACCAGTGGCTGCTGCCAGTG
GCGATAAGTCGTGTCTTACCGGGTTGGACTCAAGACGATAGTTACCGGATAAGGCGCAGCGGTCGGGCTG
AACGGGGGGTTCGTGCACACAGCCCAGCTTGGAGCGAACGACCTACACCGAACTGAGATACCTACAGCGT
GAGCTATGAGAAAGCGCCACGCTTCCCGAAGGGAGAAAGGCGGACAGGTATCCGGTAAGCGGCAGGGTCG
GAACAGGAGAGCGCACGAGGGAGCTTCCAGGGGGAAACGCCTGGTATCTTTATAGTCCTGTCGGGTTTCG
CCACCTCTGACTTGAGCGTCGATTTTTGTGATGCTCGTCAGGGGGGCGGAGCCTATGGAAAAACGCCAGC
AACGCGGCCTTTTTACGGTTCCTGGCCTTTTGCTGGCCTTTTGCTCACATGTTCTTTCCTGCGTTATCCC
CTGATTCTGTGGATAACCGTATTACCGCCTTTGAGTGAGCTGATACCGCTCGCCGCAGCCGAACGACCGA
GCGCAGCGAGTCAGTGAGCGAGGAAGCGGAAGAGCGCCTGATGCGGTATTTTCTCCTTACGCATCTGTGC
GGTATTTCACACCGCATATGGTGCACTCTCAGTACAATCTGCTCTGATGCCGCATAGTTAAGCCAGTATA
CACTCCGCTATCGCTACGTGACTGGGTCATGGCTGCGCCCCGACACCCGCCAACACCCGCTGACGCGCCC
TGACGGGCTTGTCTGCTCCCGGCATCCGCTTACAGACAAGCTGTGACCGTCTCCGGGAGCTGCATGTGTC
AGAGGTTTTCACCGTCATCACCGAAACGCGCGAGGCAGGGTGCCTTGATGTGGGCGCCGGCGGTCGAGTG

GCGACGGCGCGGCTTGTCCGCGCCCTGGTAGATTGCCTGGCCGTAGGCCAGCCATTTTTGAGCGGCCAGC
GGCCGCGATAGGCCGACGCGAAGCGGCGGGGCGTAGGGAGCGCAGCGACCGAAGGGTAGGCGCTTTTTGC
AGCTCTTCGGCTGTGCGCTGGCCAGACAGTTATGCACAGGCCAGGCGGGTTTTAAGAGTTTTAATAAGTT
TTAAAGAGTTTTAGGCGGAAAAATCGCCTTTTTTCTCTTTTATATCAGTCACTTACATGTGTGACCGGTT
CCCAATGTACGGCTTTGGGTTCCCAATGTACGGGTTCCGGTTCCCAATGTACGGCTTTGGGTTCCCAATG
TACGTGCTATCCACAGGAAAGAGACCTTTTCGACCTTTTTCCCCTGCTAGGGCAATTTGCCCTAGCATCT
GCTCCGTACATTAGGAACCGGCGGATGCTTCGCCCTCGATCAGGTTGCGGTAGCGCATGACTAGGATCGG
GCCAGCCTGCCCCGCCTCCTCCTTCAAATCGTACTCCGGCAGGTCATTTGACCCGATCAGCTTGCGCACG
GTGAAACAGAACTTCTTGAACTCTCCGGCGCTGCCACTGCGTTCGTAGATCGTCTTGAACAACCATCTGG
CTTCTGCCTTGCCTGCGGCGCGGCGTGCCAGGCGGTAGAGAAAACGGCCGATGCCGGGATCGATCAAAAA
GTAATCGGGGTGAACCGTCAGCACGTCCGGGTTCTTGCCTTCTGTGATCTCGCGGTACATCCAATCAGCT
AGCTCGATCTCGATGTACTCCGGCCGCCCGGTTTCGCTCTTTACGATCTTGTAGCGGCTAATCAAGGCTT
CACCCTCGGATACCGTCACCAGGCGGCCGTTCTTGGCCTTCTTCGTACGCTGCATGGCAACGTGCGTGGT
GTTTAACCGAATGCAGGTTTCTACCAGGTCGTCTTTCTGCTTTCCGCCATCGGCTCGCCGGCAGAACTTG
AGTACGTCCGCAACGTGTGGACGGAACACGCGGCCGGGCTTGTCTCCCTTCCCTTCCCGGTATCGGTTCA
TGGATTCGGTTAGATGGGAAACCGCCATCAGTACCAGGTCGTAATCCCACACACTGGCCATGCCGGCCGG
CCCTGCGGAAACCTCTACGTGCCCGTCTGGAAGCTCGTAGCGGATCACCTCGCCAGCTCGTCGGTCACGC
TTCGACAGACGGAAAACGGCCACGTCCATGATGCTGCGACTATCGCGGGTGCCCACGTCATAGAGCATCG
GAACGAAAAAATCTGGTTGCTCGTCGCCCTTGGGCGGCTTCCTAATCGACGGCGCACCGGCTGCCGGCGG
TTGCCGGGATTCTTTGCGGATTCGATCAGCGGCCGCTTGCCACGATTCACCGGGGCGTGCTTCTGCCTCG
ATGCGTTGCCGCTGGGCGGCCTGCGCGGCCTTCAACTTCTCCACCAGGTCATCACCCAGCGCCGCGCCGA
TTTGTACCGGGCCGGATGGTTTGCGACCGTCACGCCGATTCCTCGGGCTTGGGGGTTCCAGTGCCATTGC
AGGGCCGGCAGACAACCCAGCCGCTTACGCCTGGCCAACCGCCCGTTCCTCCACACATGGGGCATTCCAC
GGCGTCGGTGCCTGGTTGTTCTTGATTTTCCATGCCGCCTCCTTTAGCCGCTAAAATTCATCTACTCATT
TATTCATTTGCTCATTTACTCTGGTAGCTGCGCGATGTATTCAGATAGCAGCTCGGTAATGGTCTTGCCT
TGGCGTACCGCGTACATCTTCAGCTTGGTGTGATCCTCCGCCGGCAACTGAAAGTTGACCCGCTTCATGG
CTGGCGTGTCTGCCAGGCTGGCCAACGTTGCAGCCTTGCTGCTGCGTGCGCTCGGACGGCCGGCACTTAG
CGTGTTTGTGCTTTTGCTCATTTTCTCTTTACCTCATTAACTCAAATGAGTTTTGATTTAATTTCAGCGG
CCAGCGCCTGGACCTCGCGGGCAGCGTCGCCCTCGGGTTCTGATTCAAGAACGGTTGTGCCGGCGGCGGC
AGTGCCTGGGTAGCTCACGCGCTGCGTGATACGGGACTCAAGAATGGGCAGCTCGTACCCGGCCAGCGCC
TCGGCAACCTCACCGCCGATGCGCGTGCCTTTGATCGCCCGCGACACGACAAAGGCCGCTTGTAGCCTTC
CATCCGTGACCTCAATGCGCTGCTTAACCAGCTCCACCAGGTCGGCGGTGGCCCATATGTCGTAAGGGCT
TGGCTGCACCGGAATCAGCACGAAGTCGGCTGCCTTGATCGCGGACACAGCCAAGTCCGCCGCCTGGGGC
GCTCCGTCGATCACTACGAAGTCGCGCCGGCCGATGGCCTTCACGTCGCGGTCAATCGTCGGGCGGTCGA
TGCCGACAACGGTTAGCGGTTGATCTTCCCGCACGGCCGCCCAATCGCGGGCACTGCCCTGGGGATCGGA
ATCGACTAACAGAACATCGGCCCCGGCGAGTTGCAGGGCGCGGGCTAGATGGGTTGCGATGGTCGTCTTG
CCTGACCCGCCTTTCTGGTTAAGTACAGCGATAACCTTCATGCGTTCCCCTTGCGTATTTGTTTATTTAC
TCATCGCATCATATACGCAGCGACCGCATGACGCAAGCTGTTTTACTCAAATACACATCACCTTTTTAGA
CGGCGGCGCTCGGTTTCTTCAGCGGCCAAGCTGGCCGGCCAGGCCGCCAGCTTGGCATCAGACAAACCGG
CCAGGATTTCATGCAGCCGCACGGTTGAGACGTGCGCGGGCGGCTCGAACACGTACCCGGCCGCGATCAT
CTCCGCCTCGATCTCTTCGGTAATGAAAAACGGTTCGTCCTGGCCGTCCTGGTGCGGTTTCATGCTTGTT
CCTCTTGGCGTTCATTCTCGGCGGCCGCCAGGGCGTCGGCCTCGGTCAATGCGTCCTCACGGAAGGCACC
GCGCCGCCTGGCCTCGGTGGGCGTCACTTCCTCGCTGCGCTCAAGTGCGCGGTACAGGGTCGAGCGATGC
ACGCCAAGCAGTGCAGCCGCCTCTTTCACGGTGCGGCCTTCCTGGTCGATCAGCTCGCGGGCGTGCGCGA

TCTGTGCCGGGGTGAGGGTAGGGCGGGGGCCAAACTTCACGCCTCGGGCCTTGGCGGCCTCGCGCCCGCT
CCGGGTGCGGTCGATGATTAGGGAACGCTCGAACTCGGCAATGCCGGCGAACACGGTCAACACCATGCGG
CCGGCCGGCGTGGTGGTGTCGGCCCACGGCTCTGCCAGGCTACGCAGGCCCGCGCCGGCCTCCTGGATGC
GCTCGGCAATGTCCAGTAGGTCGCGGGTGCTGCGGGCCAGGCGGTCTAGCCTGGTCACTGTCACAACGTC
GCCAGGGCGTAGGTGGTCAAGCATCCTGGCCAGCTCCGGGCGGTCGCGCCTGGTGCCGGTGATCTTCTCG
GAAAACAGCTTGGTGCAGCCGGCCGCGTGCAGTTCGGCCCGTTGGTTGGTCAAGTCCTGGTCGTCGGTGC
TGACGCGGGCATAGCCCAGCAGGCCAGCGGCGGCGCTCTTGTTCATGGCGTAATGTCTCCGGTTCTAGTC
GCAAGTATTCTACTTTATGCGACTAAAACACGCGACAAGAAAACGCCAGGAAAAGGGCAGGGCGGCAGCC
TGTCGCGTAACTTAGGACTTGTGCGACATGTCGTTTTCAGAAGACGGCTGCACTGAACGTCAGAAGCCGA
CTGCACTATAGCAGCGGAGGGGTTGGATCAAAGTACTTTGATCCCGAGGGGAACCCTGTGGTTGGCATGC
ACATACAAATGGACGAACGGATAAACCTTTTCACGCCCTTTTAAATATCCGTTATTCTAATAAACGCTCT
TTTCTCTTAGGTTTACCCGCCAATATATCCTGTCAAACACTGATAGTTTAAACTGAAGGCGGGAAACGAC
AATCTGATCCAAGCTCAAGCTAAGCTTGAGCTCTGCATGCCTGCAGGTCCCCAGATTAGCCTTTTCAATT
TCAGAAAGAATGCTAACCCACAGATGGTTAGAGAGGCTTACGCAGCAGGTCTCATCAAGACGATCTACCC
GAGCAATAATCTCCAGGAAATCAAATACCTTCCCAAGAAGGTTAAAGATGCAGTCAAAAGATTCAGGACT
AACTGCATCAAGAACACAGAGAAAGATATATTTCTCAAGATCAGAAGTACTATTCCAGTATGGACGATTC
AAGGCTTGCTTCACAAACCAAGGCAAGTAATAGAGATTGGAGTCTCTAAAAAGGTAGTTCCCACTGAATC
AAAGGCCATGGAGTCAAAGATTCAAATAGAGGACCTAACAGAACTCGCCGTAAAGACTGGCGAACAGTTC
ATACAGAGTCTCTTACGACTCAATGACAAGAAGAAAATCTTCGTCAACATGGTGGAGCACGACACACTTG
TCTACTCCAAAAATATCAAAGATACAGTCTCAGAAGACCAAAGGGCAATTGAGACTTTTCAACAAAGGGT
AATATCCGGAAACCTCCTCGGATTCCATTGCCCAGCTATCTGTCACTTTATTGTGAAGATAGTGGAAAAG
GAAGGTGGCTCCTACAAATGCCATCATTGCGATAAAGGAAAGGCCATCGTTGAAGATGCCTCTGCCGACA
GTGGTCCCAAAGATGGACCCCCACCCACGAGGAGCATCGTGGAAAAAGAAGACGTTCCAACCACGTCTTC
AAAGCAAGTGGATTGATGTGATATCTCCACTGACGTAAGGGATGACGCACAATCCCACTATCCTTCGCAA
GACCCTTCCTCTATATAAGGAAGTTCATTTCATTTGGAGAGAACACGGGGGACTCTAGAGGATCCAAGGA
GATATAAGCCGCCACCATGGTGAGCAAGGGCGAGGAGCTGTTCACCGGGGTGGTGCCCATCCTGGTCGAG
CTGGACGGCGACGTAAACGGCCACAAGTTCAGCGTGTCCGGCGAGGGCGAGGGCGATGCCACCTACGGCA
AGCTGACCCTGAAGTTCATCTGCACCACCGGCAAGCTGCCCGTGCCCTGGCCCACCCTCGTGACCACCCT
GACCTACGGCGTGCAGTGCTTCAGCCGCTACCCCGACCACATGAAGCAGCACGACTTCTTCAAGTCCGCC
ATGCCCGAAGGCTACGTCCAGGAGCGCACCATCTTCTTCAAGGACGACGGCAACTACAAGACCCGCGCCG
AGGTGAAGTTCGAGGGCGACACCCTGGTGAACCGCATCGAGCTGAAGGGCATCGACTTCAAGGAGGACGG
CAACATCCTGGGGCACAAGCTGGAGTACAACTACAACAGCCACAACGTCTATATCATGGCCGACAAGCAG
AAGAACGGCATCAAGGTGAACTTCAAGATCCGCCACAACATCGAGGACGGCAGCGTGCAGCTCGCCGACC
ACTACCAGCAGAACACCCCCATCGGCGACGGCCCCGTGCTGCTGCCCGACAACCACTACCTGAGCACCCA
GTCCGCCCTGAGCAAAGACCCCAACGAGAAGCGCGATCACATGGTCCTGCTGGAGTTCGTGACCGCCGCC
GGGATCACTCTCGGCATGGACGAGCTGTACAAGGATTACGCCGAGGTCATGGATGATACTAGCGGACAAA
CTTTCGTTTCTGTGAACCTTTCTGACGAAAGCAACACGGCAAGCACTAAAGTTGAAAACGTAAAATCGGA
AGCGGATCGCTTGGAGTTTTTACGTAAAATGAATCCCTTTATTGTTGACGCTCTGGTGCGGAAAACCAAT
TATCAGGGTGCTCGCTTTCGCGCAAGAATAATAGGAGTGTGCGTGGATTGTGGTAGAAAACACGACAAGG
CGCTCAAGACTGAACGTAAGTGTAAGGTCAACAACACGCAATCTCAGAACGAGGTGGCGCATATGTTGAT
GCACGATCCCGTTAAGTATTTGAACAAAAGAAAGGCTAGAGCCTTTTCTAACGCAGAGATGTTTGCGATT
GAACTGGTTTCGTACACCAAGGAAAGGCAATTGGCGGTCGATTTAGCCGCTGAAAGGGAGAAGACGAGAC
TGGCTCGTAGACACCCAATACGTTCTCCGGAAGAAACTCCGGAACATTATAAATTCGGTATGACTGCTAA
GGCAATGTTACCGGACATCAACGCCGTAGACGTTGGTGATAACGAGGAAACTTCGTCGGAGTACCCAGTG

AGTCTGAGTGTTTCTGGCGGAGTTCTCCGTGAACACCACTTCATCTGACCTCTTCCCTAATGATATCCCG
CGGCCATGCTAGAGTCCGCAAAAATCACCAGTCTCTCTCTACAAATCTATCTCTCTCTATTTTTCTCCAG
AATAATGTGTGAGTAGTTCCCAGATAAGGGAATTAGGGTTCTTATAGGGTTTCGCTCATGTGTTGAGCAT
ATAAGAAACCCTTAGTATGTATTTGTATTTGTAAAATACTTCTATCAATAAAATTTCTAATTCCTAAAAC
CAAAATCCAGTGACCTGCAGGCATGCGACGTCG

Sequence of pH7LIC5.1.1-N4T36 (35S-GFP-N4T36)

GGGTACCCTCGAATTATCATACATGAGAATTAAGGGAGTCACGTTATGACCCCGCCGATGACGCGGGACA
AGCCGTTTTACGTTTGGAACTGACAGAACCGCAACGTTGAAGGAGCCACTGAGCCGCGGGTTTCTGGAGT
TTAATGAGCTAAGCACATACGTCAGAAACCATTATTGCGCGTTCAAAAGTCGCCTAAGGTCACTATCAGC
TAGCAAATATTTCTTGTCAAAAATGCTCCACTGACGTTCCATAAATTCCCCTCGGTATCCAATTAGAGTC
TCATATTCACTCTCAACTCGAATCCCCCCTATGAAAAAGCCTGAACTCACCGCGACGTCTGTCGAGAAGT
TTCTGATCGAAAAGTTCGACAGCGTCTCCGACCTGATGCAGCTCTCGGAGGGCGAAGAATCTCGTGCTTT
CAGCTTCGATGTAGGAGGGCGTGGATATGTCCTGCGGGTAAATAGCTGCGCCGATGGTTTCTACAAAGAT
CGTTATGTTTATCGGCACTTTGCATCGGCCGCGCTCCCGATTCCGGAAGTGCTTGACATTGGGGAGTTCA
GCGAGAGCCTGACCTATTGCATCTCCCGCCGTGCACAGGGTGTCACGTTGCAAGACCTGCCTGAAACCGA
ACTGCCCGCTGTTCTTCAGCCGGTCGCGGAGGCTATGGATGCTATCGCTGCGGCCGATCTTAGCCAGACG
AGCGGGTTCGGCCCATTCGGACCGCAAGGAATCGGTCAATACACTACATGGCGTGATTTCATATGCGCGA
TTGCTGATCCCCATGTGTATCACTGGCAAACTGTGATGGACGACACCGTCAGTGCGTCCGTCGCGCAGGC
TCTCGATGAGCTGATGCTTTGGGCGAGGACTGCCCCGAAGTCCGGCACTCGTGCACGCGGATTCGGCTCC
AACAATGTCCTGACGGACAATGGCCGCATAACAGCGGTCATTGACTGGAGCGAGGCGATGTTCGGGGATT
CCCAATACGAGGTCGCCAACATCTTCTTCTGGAGGCCGTGGTTGGCTTGTATGGAGCAGCAGACGCGCTA
CTTCGAGCGGAGGCATCCGGAGCTTGCAGGATCGCCAAGGCTCCGGGCGTATATGCTCCGCATTGGTCTT
GACCAACTCTATCAGAGCTTGGTTGACGGCAATTTCGATGATGCAGCTTGGGCGCAGGGTCGATGCGACG
CAATCGTCCGATCCGGAGCCGGGACTGTCGGGCGTACACAAATCGCCCGCAGAAGCGCGGCCGTCTGGAC
CGATGGCTGTGTAGAAGTACTCGCCGATAGTGGAAACCGACGCCCCAGCACTCGTCCGAGGGCAAAGGAA
TAGAGTAGATGCCGACCGAACAAGAGCTGATTTCGAGAACGCCTCAGCCAGCAACTCGCGCGAGCCTAGC
AAGGCAAATGCGAGAGAACGGCCTTACGCTTGGTGGCACAGTTCTCGTCCACAGTTCGCTAAGCTCGCTC
GGCTGGGTCGCGGGAGGGCCGGTCGCAGTGATTCAGGAATTAATTCCCTAGAGTCAAGCAGATCGTTCAA
ACATTTGGCAATAAAGTTTCTTAAGATTGAATCCTGTTGCCGGTCTTGCGATGATTATCATATAATTTCT
GTTGAATTACGTTAAGCATGTAATAATTAACATGTAATGCATGACGTTATTTATGAGATGGGTTTTTATG
ATTAGAGTCCCGCAATTATACATTTAATACGCGATAGAAAACAAAATATAGCGCGCAAACTAGGATAAAT
TATCGCGCGCGGTGTCATCTATGTTACTAGATCGACCGGCATGCAAGCTGATAATTCAATTCGGCGTTAA
TTCAGTACATTAAAAACGTCCGCAATGTGTTATTAAGTTGTCTAAGCGTCAATTTGTTTACACCACAATA
TATCCTGCCACCAGCCAGCCAACAGCTCCCCGACCGGCAGCTCGGCACAAAATCACCACTCGATACAGGC
AGCCCATCAGTCCGGGACGGCGTCAGCGGGAGAGCCGTTGTAAGGCGGCAGACTTTGCTCATGTTACCGA
TGCTATTCGGAAGAACGGCAACTAAGCTGCCGGGTTTGAAACACGGATGATCTCGCGGAGGGTAGCATGT
TGATTGTAACGATGACAGAGCGTTGCTGCCTGTGATCAATTCGGGCACGAACCCAGTGGACATAAGCCTC
GTTCGGTTCGTAAGCTGTAATGCAAGTAGCGTAACTGCCGTCACGCAACTGGTCCAGAACCTTGACCGAA
CGCAGCGGTGGTAACGGCGCAGTGGCGGTTTTCATGGCTTCTTGTTATGACATGTTTTTTTGGGGTACAG
TCTATGCCTCGGGCATCCAAGCAGCAAGCGCGTTACGCCGTGGGTCGATGTTTGATGTTATGGAGCAGCA
ACGATGTTACGCAGCAGGGCAGTCGCCCTAAAACAAAGTTAAACATCATGGGGGAAGCGGTGATCGCCGA
AGTATCGACTCAACTATCAGAGGTAGTTGGCGTCATCGAGCGCCATCTCGAACCGACGTTGCTGGCCGTA
CATTTGTACGGCTCCGCAGTGGATGGCGGCCTGAAGCCACACAGTGATATTGATTTGCTGGTTACGGTGA

CCGTAAGGCTTGATGAAACAACGCGGCGAGCTTTGATCAACGACCTTTTGGAAACTTCGGCTTCCCCTGG
AGAGAGCGAGATTCTCCGCGCTGTAGAAGTCACCATTGTTGTGCACGACGACATCATTCCGTGGCGTTAT
CCAGCTAAGCGCGAACTGCAATTTGGAGAATGGCAGCGCAATGACATTCTTGCAGGTATCTTCGAGCCAG
CCACGATCGACATTGATCTGGCTATCTTGCTGACAAAAGCAAGAGAACATAGCGTTGCCTTGGTAGGTCC
AGCGGCGGAGGAACTCTTTGATCCGGTTCCTGAACAGGATCTATTTGAGGCGCTAAATGAAACCTTAACG
CTATGGAACTCGCCGCCCGACTGGGCTGGCGATGAGCGAAATGTAGTGCTTACGTTGTCCCGCATTTGGT
ACAGCGCAGTAACCGGCAAAATCGCGCCGAAGGATGTCGCTGCCGACTGGGCAATGGAGCGCCTGCCGGC
CCAGTATCAGCCCGTCATACTTGAAGCTAGACAGGCTTATCTTGGACAAGAAGAAGATCGCTTGGCCTCG
CGCGCAGATCAGTTGGAAGAATTTGTCCACTACGTGAAAGGCGAGATCACCAAGGTAGTCGGCAAATAAT
GTCTAGCTAGAAATTCGTTCAAGCCGACGCCGCTTCGCCGGCGTTAACTCAAGCGATTAGATGCACTAAG
CACATAATTGCTCACAGCCAAACTATCAGGTCAAGTCTGCTTTTATTATTTTTAAGCGTGCATAATAAGC
CCTACACAAATTGGGAGATATATCATGCATGACCAAAATCCCTTAACGTGAGTTTTCGTTCCACTGAGCG
TCAGACCCCGTAGAAAAGATCAAAGGATCTTCTTGAGATCCTTTTTTTCTGCGCGTAATCTGCTGCTTGC
AAACAAAAAAACCACCGCTACCAGCGGTGGTTTGTTTGCCGGATCAAGAGCTACCAACTCTTTTTCCGAA
GGTAACTGGCTTCAGCAGAGCGCAGATACCAAATACTGTCCTTCTAGTGTAGCCGTAGTTAGGCCACCAC
TTCAAGAACTCTGTAGCACCGCCTACATACCTCGCTCTGCTAATCCTGTTACCAGTGGCTGCTGCCAGTG
GCGATAAGTCGTGTCTTACCGGGTTGGACTCAAGACGATAGTTACCGGATAAGGCGCAGCGGTCGGGCTG
AACGGGGGGTTCGTGCACACAGCCCAGCTTGGAGCGAACGACCTACACCGAACTGAGATACCTACAGCGT
GAGCTATGAGAAAGCGCCACGCTTCCCGAAGGGAGAAAGGCGGACAGGTATCCGGTAAGCGGCAGGGTCG
GAACAGGAGAGCGCACGAGGGAGCTTCCAGGGGGAAACGCCTGGTATCTTTATAGTCCTGTCGGGTTTCG
CCACCTCTGACTTGAGCGTCGATTTTTGTGATGCTCGTCAGGGGGGCGGAGCCTATGGAAAAACGCCAGC
AACGCGGCCTTTTTACGGTTCCTGGCCTTTTGCTGGCCTTTTGCTCACATGTTCTTTCCTGCGTTATCCC
CTGATTCTGTGGATAACCGTATTACCGCCTTTGAGTGAGCTGATACCGCTCGCCGCAGCCGAACGACCGA
GCGCAGCGAGTCAGTGAGCGAGGAAGCGGAAGAGCGCCTGATGCGGTATTTTCTCCTTACGCATCTGTGC
GGTATTTCACACCGCATATGGTGCACTCTCAGTACAATCTGCTCTGATGCCGCATAGTTAAGCCAGTATA
CACTCCGCTATCGCTACGTGACTGGGTCATGGCTGCGCCCCGACACCCGCCAACACCCGCTGACGCGCCC
TGACGGGCTTGTCTGCTCCCGGCATCCGCTTACAGACAAGCTGTGACCGTCTCCGGGAGCTGCATGTGTC
AGAGGTTTTCACCGTCATCACCGAAACGCGCGAGGCAGGGTGCCTTGATGTGGGCGCCGGCGGTCGAGTG
GCGACGGCGCGGCTTGTCCGCGCCCTGGTAGATTGCCTGGCCGTAGGCCAGCCATTTTTGAGCGGCCAGC
GGCCGCGATAGGCCGACGCGAAGCGGCGGGGCGTAGGGAGCGCAGCGACCGAAGGGTAGGCGCTTTTTGC
AGCTCTTCGGCTGTGCGCTGGCCAGACAGTTATGCACAGGCCAGGCGGGTTTTAAGAGTTTTAATAAGTT
TTAAAGAGTTTTAGGCGGAAAAATCGCCTTTTTTCTCTTTTATATCAGTCACTTACATGTGTGACCGGTT
CCCAATGTACGGCTTTGGGTTCCCAATGTACGGGTTCCGGTTCCCAATGTACGGCTTTGGGTTCCCAATG
TACGTGCTATCCACAGGAAAGAGACCTTTTCGACCTTTTTCCCCTGCTAGGGCAATTTGCCCTAGCATCT
GCTCCGTACATTAGGAACCGGCGGATGCTTCGCCCTCGATCAGGTTGCGGTAGCGCATGACTAGGATCGG
GCCAGCCTGCCCCGCCTCCTCCTTCAAATCGTACTCCGGCAGGTCATTTGACCCGATCAGCTTGCGCACG
GTGAAACAGAACTTCTTGAACTCTCCGGCGCTGCCACTGCGTTCGTAGATCGTCTTGAACAACCATCTGG
CTTCTGCCTTGCCTGCGGCGCGGCGTGCCAGGCGGTAGAGAAAACGGCCGATGCCGGGATCGATCAAAAA
GTAATCGGGGTGAACCGTCAGCACGTCCGGGTTCTTGCCTTCTGTGATCTCGCGGTACATCCAATCAGCT
AGCTCGATCTCGATGTACTCCGGCCGCCCGGTTTCGCTCTTTACGATCTTGTAGCGGCTAATCAAGGCTT
CACCCTCGGATACCGTCACCAGGCGGCCGTTCTTGGCCTTCTTCGTACGCTGCATGGCAACGTGCGTGGT
GTTTAACCGAATGCAGGTTTCTACCAGGTCGTCTTTCTGCTTTCCGCCATCGGCTCGCCGGCAGAACTTG
AGTACGTCCGCAACGTGTGGACGGAACACGCGGCCGGGCTTGTCTCCCTTCCCTTCCCGGTATCGGTTCA
TGGATTCGGTTAGATGGGAAACCGCCATCAGTACCAGGTCGTAATCCCACACACTGGCCATGCCGGCCGG

CCCTGCGGAAACCTCTACGTGCCCGTCTGGAAGCTCGTAGCGGATCACCTCGCCAGCTCGTCGGTCACGC
TTCGACAGACGGAAAACGGCCACGTCCATGATGCTGCGACTATCGCGGGTGCCCACGTCATAGAGCATCG
GAACGAAAAAATCTGGTTGCTCGTCGCCCTTGGGCGGCTTCCTAATCGACGGCGCACCGGCTGCCGGCGG
TTGCCGGGATTCTTTGCGGATTCGATCAGCGGCCGCTTGCCACGATTCACCGGGGCGTGCTTCTGCCTCG
ATGCGTTGCCGCTGGGCGGCCTGCGCGGCCTTCAACTTCTCCACCAGGTCATCACCCAGCGCCGCGCCGA
TTTGTACCGGGCCGGATGGTTTGCGACCGTCACGCCGATTCCTCGGGCTTGGGGGTTCCAGTGCCATTGC
AGGGCCGGCAGACAACCCAGCCGCTTACGCCTGGCCAACCGCCCGTTCCTCCACACATGGGGCATTCCAC
GGCGTCGGTGCCTGGTTGTTCTTGATTTTCCATGCCGCCTCCTTTAGCCGCTAAAATTCATCTACTCATT
TATTCATTTGCTCATTTACTCTGGTAGCTGCGCGATGTATTCAGATAGCAGCTCGGTAATGGTCTTGCCT
TGGCGTACCGCGTACATCTTCAGCTTGGTGTGATCCTCCGCCGGCAACTGAAAGTTGACCCGCTTCATGG
CTGGCGTGTCTGCCAGGCTGGCCAACGTTGCAGCCTTGCTGCTGCGTGCGCTCGGACGGCCGGCACTTAG
CGTGTTTGTGCTTTTGCTCATTTTCTCTTTACCTCATTAACTCAAATGAGTTTTGATTTAATTTCAGCGG
CCAGCGCCTGGACCTCGCGGGCAGCGTCGCCCTCGGGTTCTGATTCAAGAACGGTTGTGCCGGCGGCGGC
AGTGCCTGGGTAGCTCACGCGCTGCGTGATACGGGACTCAAGAATGGGCAGCTCGTACCCGGCCAGCGCC
TCGGCAACCTCACCGCCGATGCGCGTGCCTTTGATCGCCCGCGACACGACAAAGGCCGCTTGTAGCCTTC
CATCCGTGACCTCAATGCGCTGCTTAACCAGCTCCACCAGGTCGGCGGTGGCCCATATGTCGTAAGGGCT
TGGCTGCACCGGAATCAGCACGAAGTCGGCTGCCTTGATCGCGGACACAGCCAAGTCCGCCGCCTGGGGC
GCTCCGTCGATCACTACGAAGTCGCGCCGGCCGATGGCCTTCACGTCGCGGTCAATCGTCGGGCGGTCGA
TGCCGACAACGGTTAGCGGTTGATCTTCCCGCACGGCCGCCCAATCGCGGGCACTGCCCTGGGGATCGGA
ATCGACTAACAGAACATCGGCCCCGGCGAGTTGCAGGGCGCGGGCTAGATGGGTTGCGATGGTCGTCTTG
CCTGACCCGCCTTTCTGGTTAAGTACAGCGATAACCTTCATGCGTTCCCCTTGCGTATTTGTTTATTTAC
TCATCGCATCATATACGCAGCGACCGCATGACGCAAGCTGTTTTACTCAAATACACATCACCTTTTTAGA
CGGCGGCGCTCGGTTTCTTCAGCGGCCAAGCTGGCCGGCCAGGCCGCCAGCTTGGCATCAGACAAACCGG
CCAGGATTTCATGCAGCCGCACGGTTGAGACGTGCGCGGGCGGCTCGAACACGTACCCGGCCGCGATCAT
CTCCGCCTCGATCTCTTCGGTAATGAAAAACGGTTCGTCCTGGCCGTCCTGGTGCGGTTTCATGCTTGTT
CCTCTTGGCGTTCATTCTCGGCGGCCGCCAGGGCGTCGGCCTCGGTCAATGCGTCCTCACGGAAGGCACC
GCGCCGCCTGGCCTCGGTGGGCGTCACTTCCTCGCTGCGCTCAAGTGCGCGGTACAGGGTCGAGCGATGC
ACGCCAAGCAGTGCAGCCGCCTCTTTCACGGTGCGGCCTTCCTGGTCGATCAGCTCGCGGGCGTGCGCGA
TCTGTGCCGGGGTGAGGGTAGGGCGGGGGCCAAACTTCACGCCTCGGGCCTTGGCGGCCTCGCGCCCGCT
CCGGGTGCGGTCGATGATTAGGGAACGCTCGAACTCGGCAATGCCGGCGAACACGGTCAACACCATGCGG
CCGGCCGGCGTGGTGGTGTCGGCCCACGGCTCTGCCAGGCTACGCAGGCCCGCGCCGGCCTCCTGGATGC
GCTCGGCAATGTCCAGTAGGTCGCGGGTGCTGCGGGCCAGGCGGTCTAGCCTGGTCACTGTCACAACGTC
GCCAGGGCGTAGGTGGTCAAGCATCCTGGCCAGCTCCGGGCGGTCGCGCCTGGTGCCGGTGATCTTCTCG
GAAAACAGCTTGGTGCAGCCGGCCGCGTGCAGTTCGGCCCGTTGGTTGGTCAAGTCCTGGTCGTCGGTGC
TGACGCGGGCATAGCCCAGCAGGCCAGCGGCGGCGCTCTTGTTCATGGCGTAATGTCTCCGGTTCTAGTC
GCAAGTATTCTACTTTATGCGACTAAAACACGCGACAAGAAAACGCCAGGAAAAGGGCAGGGCGGCAGCC
TGTCGCGTAACTTAGGACTTGTGCGACATGTCGTTTTCAGAAGACGGCTGCACTGAACGTCAGAAGCCGA
CTGCACTATAGCAGCGGAGGGGTTGGATCAAAGTACTTTGATCCCGAGGGGAACCCTGTGGTTGGCATGC
ACATACAAATGGACGAACGGATAAACCTTTTCACGCCCTTTTAAATATCCGTTATTCTAATAAACGCTCT
TTTCTCTTAGGTTTACCCGCCAATATATCCTGTCAAACACTGATAGTTTAAACTGAAGGCGGGAAACGAC
AATCTGATCCAAGCTCAAGCTAAGCTTGAGCTCTGCATGCCTGCAGGTCCCCAGATTAGCCTTTTCAATT
TCAGAAAGAATGCTAACCCACAGATGGTTAGAGAGGCTTACGCAGCAGGTCTCATCAAGACGATCTACCC
GAGCAATAATCTCCAGGAAATCAAATACCTTCCCAAGAAGGTTAAAGATGCAGTCAAAAGATTCAGGACT
AACTGCATCAAGAACACAGAGAAAGATATATTTCTCAAGATCAGAAGTACTATTCCAGTATGGACGATTC

AAGGCTTGCTTCACAAACCAAGGCAAGTAATAGAGATTGGAGTCTCTAAAAAGGTAGTTCCCACTGAATC
AAAGGCCATGGAGTCAAAGATTCAAATAGAGGACCTAACAGAACTCGCCGTAAAGACTGGCGAACAGTTC
ATACAGAGTCTCTTACGACTCAATGACAAGAAGAAAATCTTCGTCAACATGGTGGAGCACGACACACTTG
TCTACTCCAAAAATATCAAAGATACAGTCTCAGAAGACCAAAGGGCAATTGAGACTTTTCAACAAAGGGT
AATATCCGGAAACCTCCTCGGATTCCATTGCCCAGCTATCTGTCACTTTATTGTGAAGATAGTGGAAAAG
GAAGGTGGCTCCTACAAATGCCATCATTGCGATAAAGGAAAGGCCATCGTTGAAGATGCCTCTGCCGACA
GTGGTCCCAAAGATGGACCCCCACCCACGAGGAGCATCGTGGAAAAAGAAGACGTTCCAACCACGTCTTC
AAAGCAAGTGGATTGATGTGATATCTCCACTGACGTAAGGGATGACGCACAATCCCACTATCCTTCGCAA
GACCCTTCCTCTATATAAGGAAGTTCATTTCATTTGGAGAGAACACGGGGGACTCTAGAGGATCCAAGGA
GATATAAGCCGCCACCATGGTGAGCAAGGGCGAGGAGCTGTTCACCGGGGTGGTGCCCATCCTGGTCGAG
CTGGACGGCGACGTAAACGGCCACAAGTTCAGCGTGTCCGGCGAGGGCGAGGGCGATGCCACCTACGGCA
AGCTGACCCTGAAGTTCATCTGCACCACCGGCAAGCTGCCCGTGCCCTGGCCCACCCTCGTGACCACCCT
GACCTACGGCGTGCAGTGCTTCAGCCGCTACCCCGACCACATGAAGCAGCACGACTTCTTCAAGTCCGCC
ATGCCCGAAGGCTACGTCCAGGAGCGCACCATCTTCTTCAAGGACGACGGCAACTACAAGACCCGCGCCG
AGGTGAAGTTCGAGGGCGACACCCTGGTGAACCGCATCGAGCTGAAGGGCATCGACTTCAAGGAGGACGG
CAACATCCTGGGGCACAAGCTGGAGTACAACTACAACAGCCACAACGTCTATATCATGGCCGACAAGCAG
AAGAACGGCATCAAGGTGAACTTCAAGATCCGCCACAACATCGAGGACGGCAGCGTGCAGCTCGCCGACC
ACTACCAGCAGAACACCCCCATCGGCGACGGCCCCGTGCTGCTGCCCGACAACCACTACCTGAGCACCCA
GTCCGCCCTGAGCAAAGACCCCAACGAGAAGCGCGATCACATGGTCCTGCTGGAGTTCGTGACCGCCGCC
GGGATCACTCTCGGCATGGACGAGCTGTACAAGGATTACGCCGAGGTCATGGATAATACTAGCGGACAAA
CTTTCGTTTCTGTGAACCTTTCTGACGAAAGCAACACAGCGACCACTGACGTCAAACCCGTGAGTTCGGA
AGCGGATCGCTTGGATTTTTTACAGAAAATGAATCCCATTATTATCGATGCTTTGATACGGAAGAATAGT
TATCAGGGCGCTCGCTTTCGCGCGAGAATAATAGGAGTGTGCGTGGATTGCGGTAGAAAACACGATAAGG
GGTTGAAGACTGAACGTAAGTGTAAGGTCAACAATACGCAGTCTCAGAACGAGGTGGCGCATATGTTAAT
GCACGACCCCGTTAAGTATTTAAACAAAAGAAAAGCTAGAGCCTTTTCTAATGCGGAGATATTTGCGATT
GATTTGGTTATGTACACCAAGGAAAGGCAATTGGCTATTGATTTGGCCGCTGAAAGGGAGAAAACGAGAC
TGGCTCGTAGACACCCGATGCGTTCTCCGGAAGAAACTCCGGAATATTATAAATTCGGTAGGACTGCTAA
AGCAATGTTACCGGACATCAACGCCGTAGACGTTGGTGATAACGAGGAAACTTCGTCGGAGTATCCAGTG
AGTCTGAGTGTTTCTGGCGGAGTTCTCCGTGAACACCACTTCATCTGACCTCTTCCCTAATGATATCCCG
CGGCCATGCTAGAGTCCGCAAAAATCACCAGTCTCTCTCTACAAATCTATCTCTCTCTATTTTTCTCCAG
AATAATGTGTGAGTAGTTCCCAGATAAGGGAATTAGGGTTCTTATAGGGTTTCGCTCATGTGTTGAGCAT
ATAAGAAACCCTTAGTATGTATTTGTATTTGTAAAATACTTCTATCAATAAAATTTCTAATTCCTAAAAC
CAAAATCCAGTGACCTGCAGGCATGCGACGTCG

Sequence of pH7LIC6.0 (35S-GFP-EV)

GGGTACCCTCGAATTATCATACATGAGAATTAAGGGAGTCACGTTATGACCCCGCCGATGACGCGGGACA
AGCCGTTTTACGTTTGGAACTGACAGAACCGCAACGTTGAAGGAGCCACTGAGCCGCGGGTTTCTGGAGT
TTAATGAGCTAAGCACATACGTCAGAAACCATTATTGCGCGTTCAAAAGTCGCCTAAGGTCACTATCAGC
TAGCAAATATTTCTTGTCAAAAATGCTCCACTGACGTTCCATAAATTCCCCTCGGTATCCAATTAGAGTC
TCATATTCACTCTCAACTCGAATCCCCCCTATGAAAAAGCCTGAACTCACCGCGACGTCTGTCGAGAAGT
TTCTGATCGAAAAGTTCGACAGCGTCTCCGACCTGATGCAGCTCTCGGAGGGCGAAGAATCTCGTGCTTT
CAGCTTCGATGTAGGAGGGCGTGGATATGTCCTGCGGGTAAATAGCTGCGCCGATGGTTTCTACAAAGAT
CGTTATGTTTATCGGCACTTTGCATCGGCCGCGCTCCCGATTCCGGAAGTGCTTGACATTGGGGAGTTCA
GCGAGAGCCTGACCTATTGCATCTCCCGCCGTGCACAGGGTGTCACGTTGCAAGACCTGCCTGAAACCGA

ACTGCCCGCTGTTCTTCAGCCGGTCGCGGAGGCTATGGATGCTATCGCTGCGGCCGATCTTAGCCAGACG
AGCGGGTTCGGCCCATTCGGACCGCAAGGAATCGGTCAATACACTACATGGCGTGATTTCATATGCGCGA
TTGCTGATCCCCATGTGTATCACTGGCAAACTGTGATGGACGACACCGTCAGTGCGTCCGTCGCGCAGGC
TCTCGATGAGCTGATGCTTTGGGCGAGGACTGCCCCGAAGTCCGGCACTCGTGCACGCGGATTCGGCTCC
AACAATGTCCTGACGGACAATGGCCGCATAACAGCGGTCATTGACTGGAGCGAGGCGATGTTCGGGGATT
CCCAATACGAGGTCGCCAACATCTTCTTCTGGAGGCCGTGGTTGGCTTGTATGGAGCAGCAGACGCGCTA
CTTCGAGCGGAGGCATCCGGAGCTTGCAGGATCGCCAAGGCTCCGGGCGTATATGCTCCGCATTGGTCTT
GACCAACTCTATCAGAGCTTGGTTGACGGCAATTTCGATGATGCAGCTTGGGCGCAGGGTCGATGCGACG
CAATCGTCCGATCCGGAGCCGGGACTGTCGGGCGTACACAAATCGCCCGCAGAAGCGCGGCCGTCTGGAC
CGATGGCTGTGTAGAAGTACTCGCCGATAGTGGAAACCGACGCCCCAGCACTCGTCCGAGGGCAAAGGAA
TAGAGTAGATGCCGACCGAACAAGAGCTGATTTCGAGAACGCCTCAGCCAGCAACTCGCGCGAGCCTAGC
AAGGCAAATGCGAGAGAACGGCCTTACGCTTGGTGGCACAGTTCTCGTCCACAGTTCGCTAAGCTCGCTC
GGCTGGGTCGCGGGAGGGCCGGTCGCAGTGATTCAGGAATTAATTCCCTAGAGTCAAGCAGATCGTTCAA
ACATTTGGCAATAAAGTTTCTTAAGATTGAATCCTGTTGCCGGTCTTGCGATGATTATCATATAATTTCT
GTTGAATTACGTTAAGCATGTAATAATTAACATGTAATGCATGACGTTATTTATGAGATGGGTTTTTATG
ATTAGAGTCCCGCAATTATACATTTAATACGCGATAGAAAACAAAATATAGCGCGCAAACTAGGATAAAT
TATCGCGCGCGGTGTCATCTATGTTACTAGATCGACCGGCATGCAAGCTGATAATTCAATTCGGCGTTAA
TTCAGTACATTAAAAACGTCCGCAATGTGTTATTAAGTTGTCTAAGCGTCAATTTGTTTACACCACAATA
TATCCTGCCACCAGCCAGCCAACAGCTCCCCGACCGGCAGCTCGGCACAAAATCACCACTCGATACAGGC
AGCCCATCAGTCCGGGACGGCGTCAGCGGGAGAGCCGTTGTAAGGCGGCAGACTTTGCTCATGTTACCGA
TGCTATTCGGAAGAACGGCAACTAAGCTGCCGGGTTTGAAACACGGATGATCTCGCGGAGGGTAGCATGT
TGATTGTAACGATGACAGAGCGTTGCTGCCTGTGATCAATTCGGGCACGAACCCAGTGGACATAAGCCTC
GTTCGGTTCGTAAGCTGTAATGCAAGTAGCGTAACTGCCGTCACGCAACTGGTCCAGAACCTTGACCGAA
CGCAGCGGTGGTAACGGCGCAGTGGCGGTTTTCATGGCTTCTTGTTATGACATGTTTTTTTGGGGTACAG
TCTATGCCTCGGGCATCCAAGCAGCAAGCGCGTTACGCCGTGGGTCGATGTTTGATGTTATGGAGCAGCA
ACGATGTTACGCAGCAGGGCAGTCGCCCTAAAACAAAGTTAAACATCATGGGGGAAGCGGTGATCGCCGA
AGTATCGACTCAACTATCAGAGGTAGTTGGCGTCATCGAGCGCCATCTCGAACCGACGTTGCTGGCCGTA
CATTTGTACGGCTCCGCAGTGGATGGCGGCCTGAAGCCACACAGTGATATTGATTTGCTGGTTACGGTGA
CCGTAAGGCTTGATGAAACAACGCGGCGAGCTTTGATCAACGACCTTTTGGAAACTTCGGCTTCCCCTGG
AGAGAGCGAGATTCTCCGCGCTGTAGAAGTCACCATTGTTGTGCACGACGACATCATTCCGTGGCGTTAT
CCAGCTAAGCGCGAACTGCAATTTGGAGAATGGCAGCGCAATGACATTCTTGCAGGTATCTTCGAGCCAG
CCACGATCGACATTGATCTGGCTATCTTGCTGACAAAAGCAAGAGAACATAGCGTTGCCTTGGTAGGTCC
AGCGGCGGAGGAACTCTTTGATCCGGTTCCTGAACAGGATCTATTTGAGGCGCTAAATGAAACCTTAACG
CTATGGAACTCGCCGCCCGACTGGGCTGGCGATGAGCGAAATGTAGTGCTTACGTTGTCCCGCATTTGGT
ACAGCGCAGTAACCGGCAAAATCGCGCCGAAGGATGTCGCTGCCGACTGGGCAATGGAGCGCCTGCCGGC
CCAGTATCAGCCCGTCATACTTGAAGCTAGACAGGCTTATCTTGGACAAGAAGAAGATCGCTTGGCCTCG
CGCGCAGATCAGTTGGAAGAATTTGTCCACTACGTGAAAGGCGAGATCACCAAGGTAGTCGGCAAATAAT
GTCTAGCTAGAAATTCGTTCAAGCCGACGCCGCTTCGCCGGCGTTAACTCAAGCGATTAGATGCACTAAG
CACATAATTGCTCACAGCCAAACTATCAGGTCAAGTCTGCTTTTATTATTTTTAAGCGTGCATAATAAGC
CCTACACAAATTGGGAGATATATCATGCATGACCAAAATCCCTTAACGTGAGTTTTCGTTCCACTGAGCG
TCAGACCCCGTAGAAAAGATCAAAGGATCTTCTTGAGATCCTTTTTTTCTGCGCGTAATCTGCTGCTTGC
AAACAAAAAAACCACCGCTACCAGCGGTGGTTTGTTTGCCGGATCAAGAGCTACCAACTCTTTTTCCGAA
GGTAACTGGCTTCAGCAGAGCGCAGATACCAAATACTGTCCTTCTAGTGTAGCCGTAGTTAGGCCACCAC
TTCAAGAACTCTGTAGCACCGCCTACATACCTCGCTCTGCTAATCCTGTTACCAGTGGCTGCTGCCAGTG

GCGATAAGTCGTGTCTTACCGGGTTGGACTCAAGACGATAGTTACCGGATAAGGCGCAGCGGTCGGGCTG
AACGGGGGGTTCGTGCACACAGCCCAGCTTGGAGCGAACGACCTACACCGAACTGAGATACCTACAGCGT
GAGCTATGAGAAAGCGCCACGCTTCCCGAAGGGAGAAAGGCGGACAGGTATCCGGTAAGCGGCAGGGTCG
GAACAGGAGAGCGCACGAGGGAGCTTCCAGGGGGAAACGCCTGGTATCTTTATAGTCCTGTCGGGTTTCG
CCACCTCTGACTTGAGCGTCGATTTTTGTGATGCTCGTCAGGGGGGCGGAGCCTATGGAAAAACGCCAGC
AACGCGGCCTTTTTACGGTTCCTGGCCTTTTGCTGGCCTTTTGCTCACATGTTCTTTCCTGCGTTATCCC
CTGATTCTGTGGATAACCGTATTACCGCCTTTGAGTGAGCTGATACCGCTCGCCGCAGCCGAACGACCGA
GCGCAGCGAGTCAGTGAGCGAGGAAGCGGAAGAGCGCCTGATGCGGTATTTTCTCCTTACGCATCTGTGC
GGTATTTCACACCGCATATGGTGCACTCTCAGTACAATCTGCTCTGATGCCGCATAGTTAAGCCAGTATA
CACTCCGCTATCGCTACGTGACTGGGTCATGGCTGCGCCCCGACACCCGCCAACACCCGCTGACGCGCCC
TGACGGGCTTGTCTGCTCCCGGCATCCGCTTACAGACAAGCTGTGACCGTCTCCGGGAGCTGCATGTGTC
AGAGGTTTTCACCGTCATCACCGAAACGCGCGAGGCAGGGTGCCTTGATGTGGGCGCCGGCGGTCGAGTG
GCGACGGCGCGGCTTGTCCGCGCCCTGGTAGATTGCCTGGCCGTAGGCCAGCCATTTTTGAGCGGCCAGC
GGCCGCGATAGGCCGACGCGAAGCGGCGGGGCGTAGGGAGCGCAGCGACCGAAGGGTAGGCGCTTTTTGC
AGCTCTTCGGCTGTGCGCTGGCCAGACAGTTATGCACAGGCCAGGCGGGTTTTAAGAGTTTTAATAAGTT
TTAAAGAGTTTTAGGCGGAAAAATCGCCTTTTTTCTCTTTTATATCAGTCACTTACATGTGTGACCGGTT
CCCAATGTACGGCTTTGGGTTCCCAATGTACGGGTTCCGGTTCCCAATGTACGGCTTTGGGTTCCCAATG
TACGTGCTATCCACAGGAAAGAGACCTTTTCGACCTTTTTCCCCTGCTAGGGCAATTTGCCCTAGCATCT
GCTCCGTACATTAGGAACCGGCGGATGCTTCGCCCTCGATCAGGTTGCGGTAGCGCATGACTAGGATCGG
GCCAGCCTGCCCCGCCTCCTCCTTCAAATCGTACTCCGGCAGGTCATTTGACCCGATCAGCTTGCGCACG
GTGAAACAGAACTTCTTGAACTCTCCGGCGCTGCCACTGCGTTCGTAGATCGTCTTGAACAACCATCTGG
CTTCTGCCTTGCCTGCGGCGCGGCGTGCCAGGCGGTAGAGAAAACGGCCGATGCCGGGATCGATCAAAAA
GTAATCGGGGTGAACCGTCAGCACGTCCGGGTTCTTGCCTTCTGTGATCTCGCGGTACATCCAATCAGCT
AGCTCGATCTCGATGTACTCCGGCCGCCCGGTTTCGCTCTTTACGATCTTGTAGCGGCTAATCAAGGCTT
CACCCTCGGATACCGTCACCAGGCGGCCGTTCTTGGCCTTCTTCGTACGCTGCATGGCAACGTGCGTGGT
GTTTAACCGAATGCAGGTTTCTACCAGGTCGTCTTTCTGCTTTCCGCCATCGGCTCGCCGGCAGAACTTG
AGTACGTCCGCAACGTGTGGACGGAACACGCGGCCGGGCTTGTCTCCCTTCCCTTCCCGGTATCGGTTCA
TGGATTCGGTTAGATGGGAAACCGCCATCAGTACCAGGTCGTAATCCCACACACTGGCCATGCCGGCCGG
CCCTGCGGAAACCTCTACGTGCCCGTCTGGAAGCTCGTAGCGGATCACCTCGCCAGCTCGTCGGTCACGC
TTCGACAGACGGAAAACGGCCACGTCCATGATGCTGCGACTATCGCGGGTGCCCACGTCATAGAGCATCG
GAACGAAAAAATCTGGTTGCTCGTCGCCCTTGGGCGGCTTCCTAATCGACGGCGCACCGGCTGCCGGCGG
TTGCCGGGATTCTTTGCGGATTCGATCAGCGGCCGCTTGCCACGATTCACCGGGGCGTGCTTCTGCCTCG
ATGCGTTGCCGCTGGGCGGCCTGCGCGGCCTTCAACTTCTCCACCAGGTCATCACCCAGCGCCGCGCCGA
TTTGTACCGGGCCGGATGGTTTGCGACCGTCACGCCGATTCCTCGGGCTTGGGGGTTCCAGTGCCATTGC
AGGGCCGGCAGACAACCCAGCCGCTTACGCCTGGCCAACCGCCCGTTCCTCCACACATGGGGCATTCCAC
GGCGTCGGTGCCTGGTTGTTCTTGATTTTCCATGCCGCCTCCTTTAGCCGCTAAAATTCATCTACTCATT
TATTCATTTGCTCATTTACTCTGGTAGCTGCGCGATGTATTCAGATAGCAGCTCGGTAATGGTCTTGCCT
TGGCGTACCGCGTACATCTTCAGCTTGGTGTGATCCTCCGCCGGCAACTGAAAGTTGACCCGCTTCATGG
CTGGCGTGTCTGCCAGGCTGGCCAACGTTGCAGCCTTGCTGCTGCGTGCGCTCGGACGGCCGGCACTTAG
CGTGTTTGTGCTTTTGCTCATTTTCTCTTTACCTCATTAACTCAAATGAGTTTTGATTTAATTTCAGCGG
CCAGCGCCTGGACCTCGCGGGCAGCGTCGCCCTCGGGTTCTGATTCAAGAACGGTTGTGCCGGCGGCGGC
AGTGCCTGGGTAGCTCACGCGCTGCGTGATACGGGACTCAAGAATGGGCAGCTCGTACCCGGCCAGCGCC
TCGGCAACCTCACCGCCGATGCGCGTGCCTTTGATCGCCCGCGACACGACAAAGGCCGCTTGTAGCCTTC
CATCCGTGACCTCAATGCGCTGCTTAACCAGCTCCACCAGGTCGGCGGTGGCCCATATGTCGTAAGGGCT

TGGCTGCACCGGAATCAGCACGAAGTCGGCTGCCTTGATCGCGGACACAGCCAAGTCCGCCGCCTGGGGC
GCTCCGTCGATCACTACGAAGTCGCGCCGGCCGATGGCCTTCACGTCGCGGTCAATCGTCGGGCGGTCGA
TGCCGACAACGGTTAGCGGTTGATCTTCCCGCACGGCCGCCCAATCGCGGGCACTGCCCTGGGGATCGGA
ATCGACTAACAGAACATCGGCCCCGGCGAGTTGCAGGGCGCGGGCTAGATGGGTTGCGATGGTCGTCTTG
CCTGACCCGCCTTTCTGGTTAAGTACAGCGATAACCTTCATGCGTTCCCCTTGCGTATTTGTTTATTTAC
TCATCGCATCATATACGCAGCGACCGCATGACGCAAGCTGTTTTACTCAAATACACATCACCTTTTTAGA
CGGCGGCGCTCGGTTTCTTCAGCGGCCAAGCTGGCCGGCCAGGCCGCCAGCTTGGCATCAGACAAACCGG
CCAGGATTTCATGCAGCCGCACGGTTGAGACGTGCGCGGGCGGCTCGAACACGTACCCGGCCGCGATCAT
CTCCGCCTCGATCTCTTCGGTAATGAAAAACGGTTCGTCCTGGCCGTCCTGGTGCGGTTTCATGCTTGTT
CCTCTTGGCGTTCATTCTCGGCGGCCGCCAGGGCGTCGGCCTCGGTCAATGCGTCCTCACGGAAGGCACC
GCGCCGCCTGGCCTCGGTGGGCGTCACTTCCTCGCTGCGCTCAAGTGCGCGGTACAGGGTCGAGCGATGC
ACGCCAAGCAGTGCAGCCGCCTCTTTCACGGTGCGGCCTTCCTGGTCGATCAGCTCGCGGGCGTGCGCGA
TCTGTGCCGGGGTGAGGGTAGGGCGGGGGCCAAACTTCACGCCTCGGGCCTTGGCGGCCTCGCGCCCGCT
CCGGGTGCGGTCGATGATTAGGGAACGCTCGAACTCGGCAATGCCGGCGAACACGGTCAACACCATGCGG
CCGGCCGGCGTGGTGGTGTCGGCCCACGGCTCTGCCAGGCTACGCAGGCCCGCGCCGGCCTCCTGGATGC
GCTCGGCAATGTCCAGTAGGTCGCGGGTGCTGCGGGCCAGGCGGTCTAGCCTGGTCACTGTCACAACGTC
GCCAGGGCGTAGGTGGTCAAGCATCCTGGCCAGCTCCGGGCGGTCGCGCCTGGTGCCGGTGATCTTCTCG
GAAAACAGCTTGGTGCAGCCGGCCGCGTGCAGTTCGGCCCGTTGGTTGGTCAAGTCCTGGTCGTCGGTGC
TGACGCGGGCATAGCCCAGCAGGCCAGCGGCGGCGCTCTTGTTCATGGCGTAATGTCTCCGGTTCTAGTC
GCAAGTATTCTACTTTATGCGACTAAAACACGCGACAAGAAAACGCCAGGAAAAGGGCAGGGCGGCAGCC
TGTCGCGTAACTTAGGACTTGTGCGACATGTCGTTTTCAGAAGACGGCTGCACTGAACGTCAGAAGCCGA
CTGCACTATAGCAGCGGAGGGGTTGGATCAAAGTACTTTGATCCCGAGGGGAACCCTGTGGTTGGCATGC
ACATACAAATGGACGAACGGATAAACCTTTTCACGCCCTTTTAAATATCCGTTATTCTAATAAACGCTCT
TTTCTCTTAGGTTTACCCGCCAATATATCCTGTCAAACACTGATAGTTTAAACTGAAGGCGGGAAACGAC
AATCTGATCCAAGCTCAAGCTAAGCTTGAGCTCTGCATGCCTGCAGGTCCCCAGATTAGCCTTTTCAATT
TCAGAAAGAATGCTAACCCACAGATGGTTAGAGAGGCTTACGCAGCAGGTCTCATCAAGACGATCTACCC
GAGCAATAATCTCCAGGAAATCAAATACCTTCCCAAGAAGGTTAAAGATGCAGTCAAAAGATTCAGGACT
AACTGCATCAAGAACACAGAGAAAGATATATTTCTCAAGATCAGAAGTACTATTCCAGTATGGACGATTC
AAGGCTTGCTTCACAAACCAAGGCAAGTAATAGAGATTGGAGTCTCTAAAAAGGTAGTTCCCACTGAATC
AAAGGCCATGGAGTCAAAGATTCAAATAGAGGACCTAACAGAACTCGCCGTAAAGACTGGCGAACAGTTC
ATACAGAGTCTCTTACGACTCAATGACAAGAAGAAAATCTTCGTCAACATGGTGGAGCACGACACACTTG
TCTACTCCAAAAATATCAAAGATACAGTCTCAGAAGACCAAAGGGCAATTGAGACTTTTCAACAAAGGGT
AATATCCGGAAACCTCCTCGGATTCCATTGCCCAGCTATCTGTCACTTTATTGTGAAGATAGTGGAAAAG
GAAGGTGGCTCCTACAAATGCCATCATTGCGATAAAGGAAAGGCCATCGTTGAAGATGCCTCTGCCGACA
GTGGTCCCAAAGATGGACCCCCACCCACGAGGAGCATCGTGGAAAAAGAAGACGTTCCAACCACGTCTTC
AAAGCAAGTGGATTGATGTGATATCTCCACTGACGTAAGGGATGACGCACAATCCCACTATCCTTCGCAA
GACCCTTCCTCTATATAAGGAAGTTCATTTCATTTGGAGAGAACACGGGGGACTCTAGAGGATCCAAGGA
GATATAACATTACGCCGAGGCCTCTTCCCTATATGGTGAGCAAGGGCGAGGAGCTGTTCACCGGGGTGGT
GCCCATCCTGGTCGAGCTGGACGGCGACGTAAACGGCCACAAGTTCAGCGTGTCCGGCGAGGGCGAGGGC
GATGCCACCTACGGCAAGCTGACCCTGAAGTTCATCTGCACCACCGGCAAGCTGCCCGTGCCCTGGCCCA
CCCTCGTGACCACCCTGACCTACGGCGTGCAGTGCTTCAGCCGCTACCCCGACCACATGAAGCAGCACGA
CTTCTTCAAGTCCGCCATGCCCGAAGGCTACGTCCAGGAGCGCACCATCTTCTTCAAGGACGACGGCAAC
TACAAGACCCGCGCCGAGGTGAAGTTCGAGGGCGACACCCTGGTGAACCGCATCGAGCTGAAGGGCATCG
ACTTCAAGGAGGACGGCAACATCCTGGGGCACAAGCTGGAGTACAACTACAACAGCCACAACGTCTATAT

CATGGCCGACAAGCAGAAGAACGGCATCAAGGTGAACTTCAAGATCCGCCACAACATCGAGGACGGCAGC
GTGCAGCTCGCCGACCACTACCAGCAGAACACCCCCATCGGCGACGGCCCCGTGCTGCTGCCCGACAACC
ACTACCTGAGCACCCAGTCCGCCCTGAGCAAAGACCCCAACGAGAAGCGCGATCACATGGTCCTGCTGGA
GTTCGTGACCGCCGCCGGGATCACTCTCGGCATGGACGAGCTGTACAAGTAATGATATCCCGCGGCCATG
CTAGAGTCCGCAAAAATCACCAGTCTCTCTCTACAAATCTATCTCTCTCTATTTTTCTCCAGAATAATGT
GTGAGTAGTTCCCAGATAAGGGAATTAGGGTTCTTATAGGGTTTCGCTCATGTGTTGAGCATATAAGAAA
CCCTTAGTATGTATTTGTATTTGTAAAATACTTCTATCAATAAAATTTCTAATTCCTAAAACCAAAATCC
AGTGACCTGCAGGCATGCGACGTCG

Supplemental Data 4. Amino acid sequence alignment, classification, and phylogenetic tree analysis

Supplemental Data 4-1 : p23 amino acid sequences

>AJ297704.1
tfvsvnlsdesntastrvenvkseadrleflrkmnpliidalvrktnyqgarfrariigvcvdcg
rkhdkalkterkckvnntqsqnevahmlmhdpvkylnkrkarafsnaemfaielvlytkekqlav
dlaaerektrlarrhpmrspeetpehykfgmtasamlpelnavgvgdnedtsseysvslsvsggv
l

>DQ831487.1
tastevktvssevdrleflrkmnpfiidalirktsyqgarfrariigvcvdcgrkhdkglknerk
ckvnntqsqnevahmlmhdpvkylnkrkarafsnaemfaidlvmytkerqlavdlaaerektrla
rrhpmrspeetpehykfgmtakaispdinavdvgdnedtsseyp

>DQ831488.1
tastavrtvsseadrleflrkmnpfiidalvrktnyqgarfrakiigvcvdcgrkhdkglkterk
ckvnntqsqnevahmlmhdpvkylnkgkarafsnaemfaidlvmytketqlavdlaaerektrla
rrhpmrspeetpefykfgitakamlpninavdvgdnedtsseyp

>DQ831493.1
tastrvenvkseadrleflrkmnpfiidalvrktnyqgarfrariigvcvdcgrkhdkalkterk
ckvnntqsqnevahmlmhdpvkylnkrkarafsnaetfaielvlytkekqlavdlaaerektrla
rrhpmrspeetpehykfgmtakamlpefnavgvgdnedtsseyp

>DQ831495.1
tastkvetvkseadrleflrkmnpfiidalvrktnyqgarfrariigvcvdcgrkhdkgfkterk
ckvnntqsqnevahmlmhdpvkylnkrkarafsnaetfaidlvlytkekqlavdlaaerektrla
rrhpmrspeetpehykfgmtakamlpefnavgvgdnedtsseyp

>DQ831496.1
tastkvenvkseadrleflrkmnpfvidalvrktnyqgarfrariigvcvdcgrkhdralkterk
ckvnntqsqnevahmlmhdpvrylnkrkarafsnaetfaielvlytkerqlavdlaaerektrla
rrhpmrspeetpeyykfgmtakamlpdinavdvgdneetsseyp

>AJ298843.1
mndtsgqtfvsvnlsdesntastrvenvkseadrleflrkmnpliidalvrktnyqgarfrarii
gvcvdcgrkhdkalkterkckvnntqsqnevahmlmhdpvkylnkrkarafsnaemfaielvlyt
kekqlavdlaaerektrlarrhpmrspeetpehykfgmtasamlpelnavgvgdnedtsseypvs
lsvsggvlrehhfi

>U42006.1
mdntsgqtfisvnlsdesntasteikavsseadrleflrkmnpfiidalirknnyqgarfrarii
gvcvdcgrkhdkasrterkckvnntlvqnevahmlmhdpvkylnkrkarafsnaemfaidlvmht
kerqlavdlaaerektrlarrhpmrspeetpehykfgttakamlpdinavdvgdnedtsseypvs
lsvsggvlrehhfi

>U42017.1
mdntsgqtfisvnlsdesntastevktvsseadrleflrkmnpfiidalirktsyqgarfrarii
gvcvdcgrkhdkglkterkckvnntqsqnevahmlmhdpvkylnkrkarafsnaemfaidlvmht
kerqlavglaaerektrlarrhpmrspeetpehykfgmtakatlpdinaidvgdnedasseypvs
lsvsggvlrehhfi

>U42018.1
mdntsgqtfisvnlsdksntastevktvsseadrleflrkmnpfvidalirktsyqgarfrarii
gvcvdcgrkhekgsrterkckvnntqsqnevahmlmhdpvkylskrkarafsnaemfaidlvmyt
kerqlavdlaaerektrlarkhpmrspeetpehykfgmtakamlpdinvvnvgdnedtsseypvs
lsvssgvlrehhli
>U42019.1
mdntsgqtfvsvnlsdesntastkvenvkseadrleflrkmnpfiidalvrktnyqgarfrarii
gvcvdcgrkhdkalkterkckvnntrsqnevahmlmhdpvkylnkrkarafsnaemfaielvlyt
kerqlavdlaaerektrlarrhpirspeetpehykfgmtakamlpdinavdvgdneetsseypvs
lsvsggvlrehhfi
>U42020.1
mdntsgqtfvsvnlsdesntastkvenvkseadrleflrkmnpviidalvrktnyqgarfrarii
gvcvdcgrkhdkalkterkckvnntqsqnevahmlmhdpvkyltkrkarafsnaemfaielvlyt
kerqlavdlaaerektrlarrhpmrspeetpehykfgmtakamlpdinavdvgdseetsseypvs
lsvsggvlrehhfi
>U42021.1
mdntsgqtfvsvnlsdesntastkvenvkseadrldflrkmnpfivdalvrktnyqgarfrarii
gvcvdcgrkhdkalkterkckvnntqsqnevahmlmhdpvkylnkrkarafsnaemfaielvlyt
kerqlavdlaaerektrlarrhpirspeetpehykfgmtakamlpdinavdvgdneetsseypvs
lsvsggvlrehhfi
>U42022.1
mdnpsgqtfisvnlsdesntastetkavsseadrleflrkmnpfiidalirknsyqgarfrarii
gvcvdcgrkhdkasrterkckvnntqsqnevahmlmhdpvkylnkrkarafsnaemfaidlvmht
kerqlavdlaaerektrlarrhpmrspeetpehykfgmtakamlpninavdvgdnedtsseypvs
lsvsggvlrehhli
>U42023.1
mdntsgqtfisvnlsdesntastevktvtseadrleflrkmnpftidalirktsyqgarfrarii
gvcvdcgrkhdkglkterkckvnntqsqnevahmlmhdpvkylnkrkarafsnaemfaidlvmht
kerqlavglaaerektrlarrhpmrspeetpehykfgmtakamlpdinaigvgdnedtsseypvs
lsvsggvlrehhfi
>U42024.1
mdntsgqtfvsvnlsdesntastkvenvkseadrleflrkmnpfivdalvrktnyqgarfrarii
gvcvdcgrkhdkalkterkckvnntqsqnevahmlmhdpvkylnkrkarafsnaemsaielvsyt
kerqlavdlaaerektrlarrhpirspeetpdyykfgmtakamlpdinavdvgdneetsseypvs
lsvsggvlrehhfi
>EU262682.1
desntastavrtvsseadrleflrkmnpfiidalvrktnyqgarfrakiigvcvdcgrkhdkglk
terkckvnntqsqnevahmlmhdpvkylnkgkarafsnaemfaidlvmytkekqlavnlaaerek
trlarrhpmrspeetpefykfgitakamlpninavdvgdnedtsseypvslsvsd
>EU262683.1
desntastavrtvsseadrleflrkmnpfiidalvrktnyqgarfrakiigvcadcgrkhdkglk
terkckvnntqsqnevahmlmhdpvkylnkgkarafsnaemfaidlvmytkekqlavnlaaerek
trlarrhpmrspeetpefykfgitakamlpninavdvgdnedtsseypvslsvsd
>EU262685.1

desntasteikavsseadrleflrkmnpfiidalirknsyqgarfrariigvcvdcgrkhdkasr
terkckvnntqsqnevahmlmhdpvkylnkrkarafsnaemfaidlvmhtkerqlavdlaaerek
trlarrhpmrspeetpehykfgmtakamlpdinavdvgdnedtsseypvslsvsg
>EU262686.1
desntasteikavsseadrleflrkmnpfiidalvrktnyqgarfrakiigvcvdcgrkhdkglk
terkckvnntqsqnevahmlmhdpvkylnkgkarafsnaemfaidlvmytkekqlavnlaaerek
trlarrhpmrspeetpefykfgttakamlpninavdvgdnedtsseypvslsvsd
>EU262688.1
desntasteikavsseadrleflrkmnpfiidalirknsyqgarfrariigvcvdcgrkhdkasr
terkckvnntqsqnevahmlmhdpveylnkrkarafsnaeifaidlvmhtkerqlavdlaaerek
trlarrhpmrspeetpehykfgmtakamlpdinavdvgdnedtsseypvslsvsd
>EU262689.1
desntastavrtvsseadrleflrkmnpfiidalvrktnyqgarfrakiigvcvdcgrkhdkglk
terkckvnntqsqnevahmlmhdpvkylnkgkarafsnaemfaidlvmytkekqlavnlaaerek
trlarrhpmrspeetpefykfgttakamlpninavdvgdnedtsseypvslsvsd
>EU483657.1
gqtfvsvnlsdesntasteikavsseadrleflrkmnpfiidalirknsyqgarfrariigvcvd
cgrkhdkasrterkckvnntqsqnevahmlmhdpvkylskrkarafsnaemfaidlvmhtkerql
avdlaaerektrlarrhpmrspeetpehykfgmtakamlpdinavdvgdnedtsseypvslsvsg
gvlrehhfi
>EU487601.1
gqtfvsvnlsdesntasteikavsseadrleflrkmnpfiidalirknsyqgarfrariigvcvd
cgrkhdkasrterkckvnntqsqnevahmlmhdpvkylnkgkarafsnaemfaidlvmhtkerql
avdlaaerektrlarrhpmrspeetpehykfgmtakamlpdinavdvgdnedtsseypvslsvsg
gvlrehhfi
>EU487603.1
gqtfvsvnlsdesntastkvenvkseadrleflrkmnpfivdalvrktnyqgarfrariigvcvd
cgrkhdkalkterkckvnntqsqnevahmlmhdpvkylnkrkarafsnaemfaielvfytkerql
avdlaaerektrlarrhpirspeetpehykfgmtakamlpdinavdvgdneetsseypvslsvsg
gvlrehhfi
>FJ860136.1
mddtsgqtfisvnlsdesntastevktvsleadrleflrkmnpfiidalirktnyqgarfrarii
gvcvdcgrkhdkasrterkckvnntqsqaevahmlmhdpvkylnkrkarafsnaemfaidlvmht
kerqlavdlaaerektrlarrhpmrspeetpehykfgitakamlpdidaidvgdnedtsseypvs
lsvsggvlrehhfil
>FJ860138.1
mddtsgqtfisvnlsdesntastevktvsleadrleflrkmnpfiidalirktnyqgarfrarii
gvcvdcgrkhdkasrterkckvnntqsqaevahmlmhdpvkylnkrkarafsnaemfaidlvmht
kerqlavdlaaerektrlarrhpmrspeetpehykfgitakamlpdidaidvgdnedtsseypvs
lsvsggvsrehhfi
>FJ860140.1
mddtsgqtfisvnlsdesntastevktvsleadrleflrkmnpfiidalirktnyqgarfrarii
gvcvdcgrkhdkasrterkckvnntqsqaevahmlmhdpvkylnkrkarafsnaemfaidlvmht
kerqlavdlaaerektrlarrhpmrspeetpehykfgitakamlpdinaidvgdnedtsseypvs

lsvsggvsrehhfi
>FJ860142.1
mddtsgqtfvsvnlsdesntastavrtvsseadrleflrkmnpfiidalvrktnyqgarfrarii
gvcvdcgrkhdkglkterkckvnntqsqnevahmlmhdpvrylnkgkarafsnaemfaidlvmyt
kekqlavnlaaerektrlarrhpmrspeetpehykfgitakamlpninavdvgdnedtsseypvs
lsvsdgvlrehhfi
>FJ860143.1
mddisgqtfvsvnlsdesntastrvenvnseadrleflrkmnpliidalvrktnyqgarfrarii
gvcvdcgrkhdkalkterkckvnntqsqnevahmlmhdpvkylnkrkarafsnaemfaielvlyt
kerqlavdlaaerektrlarrhpirspeetpehykfgmtakamlpdinavdvgdneetsseypvs
lsvsggvlrehhfi
>FJ860144.1
mddtigqtfvsvnlsdesntastrvenvnseadrleflrkmnpliidalvrktnyqgarfrarii
gvcvdcgrkhdkalkterkckvnntqsqnevahmlmhdpvkylnkrkarafsnaemfaielvlyt
kerqlavdlaaerektrlarrhpirspeetpehykfgmtakamlpdinavdvgdneetsseypvs
lsvsggvlrehhfi
>FJ860145.1
mddtsgqtfisvnlsdesntasteiktvsseadrleflrkmnpfiidalirknsyqgarfrarii
gvcvdcgrkhdkasrterkckvnntqsqaevahmlmhdpvkylnkrkarafsnaemfaidlvmht
kerqlavdlaaerektrlarrhpmrspeetpehykfgitakamlpdinaidvgdnedtsseypvs
lsvsggvlrehhfi
>FJ860146.1
mddtsgqtfvsvnlsdesntastrvenvkseadrleflrkmnpliidalvrktnyqgarfrarii
gvcvdcgrkhdkalkterkckvnntqsqnevahmlmhdpvkylnkrkarafsnaemfaielvlyt
kerqlavdlaaerektrlarrhpirspeetpehykfgmtakamlpdinavdvgdneetsseypvs
lsvsggvlrehhfi
>GQ475044.1
mdntsgqtfisvnlsdesntasteiktvsseadrleflrkmnpfiidalirknsyqgarfrarii
gvcvdcgrkhdkasrterkckvnntqsqnevahmlmhdpvkylnkrkarafsnaemfaidlvmht
kerqlavdlaaerektrlarrhpmrspeetpehykfgmtakamlpdinavdvgdnedtsseypvs
lsvsggvlrehhfi
>GQ475045.1
mdntsgqtfvsvnlsdesntasteikavsseadrleflrkmnpfiidalirknsyqgarfrarii
gvcvdcgrkhdkasrterkckvnntqsqnevahmlmhdpvkylnkrkarafsnaetfaidlvmht
kerqlavdlaaerektrlarrhpmrspeetpehykfgmtakamlpdinavdvgdnedtsseypvs
lsvsggvlrehhfi
>GQ475046.1
mdntsgqtfisvnlsdesnpasteiktvsseadrleflrkmnpfiidalirknsyqgarfrarii
gvcvdcgrkhdkasrterkcrvnntqsqnevahmlmhdpvkylnkrkarafsnaemfaidlvvht
kerqlavdlaaerektrlarrhpmrspeetpehykfgmtakamlpdinavdvgdnedtsseypvs
lsvsggvlrehhfi
>GQ475047.1
mdntsgttfisvnlsdesntasteiktvsseadrleflrkmnpfiidalirknsyqgarfrarii
gvcvdcgrkhdkasrterkckvnntqsqnevahmlmhdpvkylnkgkarafsnaemfaidlvmht

kerrlavdlaaerektrlarrhpmrspeetpehykfgmtakamlpdinavdvgdnedtsseypvs
lsvsggvlrehhfi

>GQ475048.1

mdntsgqtfisvnlsdesntasteikavsseadrleflrkmnpfiidalirknsyqgarfrarii
gvcvdcgrkhdkasrterkckvnntqsqnevahmlmhdpveylnkrkarafsnaemfaidlvmht
kerqlavdlaaerektrlarrhpmrspeetpehykfgmtakamlpdinavdvgdnedtsseypvs
lsvsggvlrehhfi

>GQ475049.1

mdntsgqtfvsvnlsdesntastavrtvsseadrleflrkmnpfiidalirktnyqgarfrakii
gvcvdcgrkhdkglkterkckvnntqsqnevahmlmhdpvkylnkgkarafsnaemfaidlvmyt
kekqlavnlaaerektrlarrhpmrspeetpefykfgitakamlpninavdvgdnedtsseypvs
lsvsdgvlrehhfi

>GQ475051.1

mdntsgqtfvsvnlsdegntastavrtvsseadrleflrkmnpfiidalvrktnyqgarfrakii
gvcvdcgrkhdkelkterkckvnntqsqnevahmlmhdpvkylnkgkarafsnaemfaidlvmyt
kekqlavnlaaerektrlarrhpmrspeetpefykfgitakamlpninavdvgdnedtsseypvs
lsvsdgvlrehhfi

>GQ475052.1

mdntsgqtfvsvnlsdesntastavrtvsseadrleflrkmnpfiidalvrktnyqgarfrakii
gvcvdcgrkhdkglkterkckvnntqsqnevahmlmhdpvkylnkgkarafsnaemfaidlvmyt
kekqlavnlaaerektrlarrhpmrspeetpefykfgitakamlpninavdvgdnedtsseypvs
lsvsdgvlrehhfi

>GQ338645.1

tfvsvnlsdesntastevenvkseadrleflrkmnpfiidalvrktnyqgarfrariigvcvdcg
rkhdkalkterkckvnntqsqnevahmlmhdpvkylnkrkarafsnaetfaielvlytkekqlav
dlaaerektrlarrhpmrspeetpehykfgmtakamlpdfnavdvgdnedtsseypvslsvsggv
l

>GQ338646.1

tfvsvnlsdesntastrvenvkseadrleflrkmnpfiidalvrktnyqgarfrariigvcvdcg
rkhdkalkterkckvnntqsqnevahmlmhdpvkylnkrkarafsnaetfaielvlytkekqlav
dlaaerektrlarrhpmrspeetpehykfgmtakamlpefnavgvgdsedtsseypvslsvsggv
l

>GQ338647.1

tfisvnlsdesntasteiktvsseadrleflrkmnpfiidalirknsyqgarfrariigvcvdcg
rkhdkasrterkckvnntqsqnevahmlmhdpvkylnkrkarafsnaemfaidlvmhtkerqlav
dlaaerektrlarrhpmrspeetpehykfgmtakamlpdinavdvgdnedtsseypvslsvsggv
l

>GQ338648.1

tfvsvnlsdesntastkvenvkseadrleflrkmnpviidalvrktnyqgarfrariigvcvdcg
rkhdkalkterkckvnntqsqnevahmlmhdpvkylnkrkarafsnaemfaielvlytkerqlav
dlaaerektrlarrhpmrspeetpehykfgmtakamlpdinaadvddneetsseypvslsvsggv
l

>GQ338650.1

tfisvnlsdesntastevktvsseadrleflrkmnpfiidalirktsyqgarfrariigvcvdcg

rkhdkglkterkckvnntqsqnevahmlmhdpvkylskgkarafsnaekfaidlvmytkekqlav
nltaerektrlarrhpmrspeetpehykfgmtakamlpdinavvvgdnedtsseypvslsisdgv
l
>GQ338651.1
tfvsvnlsdesntastrvenvkpeadrleflrkmnpfiidalvrktnyqgarfrariigvcvdcg
rkhdkalkterkckvnntqsqnevahmlmhdpvkylnkrkarafsnaemfaielvsytkekqlav
dlaaerektrlarrhpmrspeetpefykfgmtakamlpelnavgvgdnedtsseypvslsvsggv
l
>GQ338652.1
tfisvnlsdesntasteiktvsseadrleflrkmnpfvidalirktsyqgarfrariigvcvdcg
rkhdkgsrterkckvnntqsqnevahmlmhdpvkylskrkarafsnaemfaidlvmytkerqlav
dlaaerektrlarkhpmrspeetpeyykfgmtakamlpdinvvgvgdnedtsseypvslsvsggv
l
>GQ338653.1
tfisvnlsdesntasteikavsseadrleflrkmnpfiidalirknsyqgarfrariigvcvdcg
rkhdkasrterkckvnntqsqnevahmlmhdpvkylnkrkarafsnaemfaidlvmhtkerqlav
dlaaerektrlarrhpmrspeetpehykfgmtakamlpdinavdvgdnedtsseypvslsvsggv
l
>GQ338654.1
tfisvnlsdesntasteiktvsseadrleflrkmnpfiidalirknsyqgarfrariigvcvdcg
rkhdkasrterkckvnntqsqnevahmlmhdpvkylnkgkarafsnaemfaidlvmhtkerqlav
dlaakrektrlarrhpmrspeetpehykfgmtakamlpdinavdvgdnedtsseypvslnvsggv
l
>GQ338656.1
tfvsvtlsdesntastrvenvkpeadrleflrkmnpfiidalvrktnyqgarfrakiigvcvdcg
rkhdkalkterkckvnntqsqnevahmlmhdpvkylnkrkarafsnaetfaielvlytkekqlav
dlaaerektrlarrhpmrspeetpehykfgmtakamlpelnavgvgdnedtsseypvslsvsggv
l
>GQ338657.1
tfvsvnlsdesntastavrtvsseadrleflrkmnpfiidalvrktnyqgarfrakiigvcvdcg
rkhdkglkterkckvnntqsqnevahmlmhdpvkylnkgkarafsnaemfaidlvmytkekqlai
nlaaerektrlarrhpmrspeetpefykfgitakamlpninavdvgdnedtsseypvslsvsdgv
l
>GQ338658.1
tfisvnlsdesntastevktvsseadrleflrkmnpfiidalirktsyqgarfrariigvcvdcg
rkhdkgsrterkckvnntqsqnevahmlmhdpvkylnkrkarafsnaemfaidlvmhtkerqlav
dlaaerektrlarkhpmrspeetpehykfgmtakamlpdinavdvgdnedtsseypvslsvsggv
l
>GQ338659.1
tfvsvnlsdesntastevenvnseadrleflrkmnpfiidalvrktnyqgarfrariigvcvdcg
rkhdkalrterkckvnntqsqnevahmlmhdpvkylnkrkarafsnaemfaielvsytkekqlav
dlaaerektrlarrhpmrspeetpefykfgmtakamlpdinavgvgdnedtsseypvslsvsggv
l
>HQ329231.1

mdntsgqtfisvslsdesntastevktvsseadrleflrkmnpfvidalirktsyqgarfrarii
gvcvdcgrkhdkgsrterkckvnntqsqnevahmlmhdpvkylskrkarafsnaemfaidlvmyt
kerqlavdlaaerektrlarkhpmrspeetpehykfgmtakamlpdinvvnvgdnedtsseypvs
lslrggvlrehhfil
>HQ329232.1
mdntsgqtfisvnlsdesntastevktvsseadrleflrkmnpfiidalirktsyqgarfrarii
gvcvdcgrkhdkgsrterkckvnntqsqnevahmlmhdpvkylskrkarafsnaemfaidlvmyt
kerqlavdlaaerektrlarkhpmrspeetpehykfgmtakamlpdvnvvdvgdnedtsseypvs
lsisggvlrehhfil
>HQ329233.1
mdntsgqtfisvnlsdesntastevktvsseadrleflrkmnpfvidalirktsyqgarfrarii
gvcvdcgrkhdkgsrterkckvnntqsqnevahmlmhdpvkylskrkarafsnaemfaidlvmyt
kerqlavdlaaerektrlarkhpmrspeetpehykfgmtakamlpdinvvnvgdnedtsseypvs
lsvsggvlrehhfil
>HQ329234.1
mdntsgqtfisvnlsdesntastevktvgseadrleflrkmnpfvidalirktsyqgarfrarii
gvcvdcgrkhdkatrterkckvnntqsqaevahmlmhdpvkyltkrkarafsnaemfaidlvmht
kerqlavnlaaerektrlarrhpmrspeetpehykfgmtakamlpdinaidvgdnedtsseypvs
lsisggvlrehhfil
>HQ329235.1
mdntsgqtfisvnlsdesntastevktvgseadrleflrkmnpfiidtlirktsyqgarfrarii
gvcvdcgrkhdkatrterkckvnntqsqaevahmlmhdpvkylikrkarafsnaemfaidlvmht
kerqlavnlaaerektrlarrhpmrspeetpehykfgmtakamlpdinaidvgdnedtsseypvs
lsisggvlrehhfil
>HQ329236.1
mdntsgqtfisvnlsdesntastevktvgseadrleflrkmnpfiidalirktsyqgarfrarii
gvcvdcgrkhdkatrterkckvnntqsqaevahmlmhdpvkylikrkarafsnaemfaidlvmht
kerqlavdlaaerektrlarrhpmrspeetpehykfgmtakamlpdinaidvgdnedtsseypvs
lsisggvlrehhfil
>HQ329237.1
mdntsgqtfisvnlsdesntastevktvsseadrleflrkmnpfvidalirktsyqgarfrarii
gvcvdcgrkhdkgsrterkckvnntqsqnevahmlmhdpvkylskrkarafsnaemfaidlvmyt
kerqlavdlaaerektrlarkhpmrspeetpeyykfgmtakamlpdinvvnvgdnedtsseypvs
lsvsggvlrehhfil
>HQ329238.1
mdntsgqtfisvnlsdesntastevktvsseadrleflrkmnpfiidalirknsyqgarfrarii
gvcvdcgrkhdkglkterkckvnntqsqnevahmlmhdpvkylnkgkarpfsnaemfaidlvmht
kerqlavnlaaerektrlarrhpmrspeetpeyykfgmtakamlpdinainvgdnedtsseypvs
lsvsggvlrehhfil
>HQ329239.1
mdntsgqtfisvnlsdesntastevktvsseadrleflrkmnpfvidalirktsyqgarfrarii
gvcvdcgrkhekgsrterkckvnntqsqnevahmlmhdpvkylskrkarafsnaemfaidlvmyt
kerqlavdlaaerektrlarkhpmrspeetpeyykfgmtakavlpdinvvnvgdnedtsseypvk
ssvsggvlrehhfil

>HQ329240.1
mdntsgqtfisvnlsdesntastevktvgseadrleflrkmnpfiidalirktsyqgarfrarii
gvcvdcgrkhdkatrterkckvnntqsqaevahmlmhdpvkylikrkarafsnaemfaidlvmht
kerqlavnlaaerektrlarrhpmrspeetpehykfgmtakamlpdinaidvgdnedtsseypvs
lsisggvlrehhfil
>HQ329241.1
mdntsgqtfisvnlsdesntastevktvgseadrleflrkmnpfvidalirktsyqgarfrarii
gvcvdcgrkhdkatrterkckvnntqsqaevahmlmhdpvkylikrkarafsnaemfaidlvmht
kerqlavnlaaerektrlarrhpmrspeetpehyefgmtakamlpdvnaidvgdnedtsseypvs
lsisggvlrehhfil
>HQ329242.1
mdntsgqtfisvnlsdesntastevktvsseadrleflrkmnpftidalirktsyqgarfrarii
gvcvdcgrkhdkvsrterkckvnntqsqnevahmlmhdpvkylnkrkarafsnaemfaidlvmht
kekqlavdlaaerektrlarrhpmrspeetpeyykfgmtakamlpdinaidvgdnedtsseypvs
lsvsggvlrehhfil
>HQ329243.1
mdntsgqtfisvnlsdesntastevktvsseadrleflrkmnpfvidalirktsyqgarfrarii
gvcvdcgrkhdkglrterkckvnntqsqnevahmlmhdpvkylskrkarafsnaemfaidlvmyt
kerqlavdlaaerektrlarkhpmrspeetpehykfgmtakamlpdinvvnvgdnedtsseypvs
lnvsggvlrehhfil
>HQ329244.1
mdntsgqtfisvnlsdeshtastevktvsseadrleflrkmnpfiidalirktsyqgarfrarii
gvcvdcgrkhdkgsrterkckvnntqsqnevahmlmhdpvkylskrkarafsnaemfaidlvmyt
kerqlavdlaaerektrlarkhpmrspeetpehykfgmtakamlpdvnvvdvgdnedtsseypvs
lsisggvlrehhfil
>HQ329245.1
mdntsgqtfisvnlsdesntastevktvsseadrleflrkmnpfiidalirktsyqgarfrarii
gvcvdcgrkhdkasrterkckvnntqsqnevahmlmhdpvkylnkrkarafsnaemfaidlvmht
kekqlavdlaaerektrlarrhpmrspeetpeyykfgmtakamlpdinaidvgdnedtsseypvs
lsvsggvlrehhfil
>HQ329246.1
mdntsgqtfisvnlsdesntastevktvssevdrleflrkmnpftidalirkpsyqgarfrarii
gvcvdcgrkhdkasrterkckvnntqsqnevahmlmhdpvkylnkrkarafsnaemfaidlvmht
kekqlavdlaaerektrlarrhpmrspeetpeyykfgmtakamlpdinaidvgdnedtsseypvs
lsvsggvlrehhfil
>AJ579762.1
mddtigqtfvsvnlsdesntastkvenvkseadrleflrkinpfivdalvrktnyqgarfrarii
gvcvdcgrkhdkalkterkckvnntqsqnevahmlmhdpvkylnkrkarafsnaemfaielvlyt
kerqlavdlaaerektrlarrhpirspeetpehykfgmtakamlpdinavdvgdneetsseypvs
lsvsggvlrehhfi
>AJ579764.1
mdntsgqtfvsvnlsdesntastkvenvkseadrleflrkmnpfivdalvrktnyqgarfrarii
gvcvdcgrkhdkalkterkckvnntqsqnevahmlmhdpvkylnkrkarafsnaemfaielvsyt
kerqlavdlaaerektrlarrhpirspeetpehykfgmtakamlpdinavdvgdneetsseypvs

lsvsggvlrehhfi
>AJ579766.1
mddtsgqtfvsvnlsdesntastkvenvkseadrleflrkmnpfivdalvrktnyqgarfrarii
gvcvdcgrkhdkalkterkckvnntqsqnevahmlmhdpvkylnkrkarafsnaemfaielvlyt
kerqlavdlaaerektrlarrhpirspeetpehykfgmtakamlpdtnavdvgdneetsseypvs
lsvsggvlrehhfi
>AJ579767.1
mndtsgqtfvsvnlsdesntastevktvsseadrleflrkmnpftidalirkasyqgarfrarii
gvcvdcgrkhdkglkterkckvnntqsqnevahmlmhdpvkylnkrkarafsnaemfaielvlht
kerqlavdlaaerektrlarrhpmrspeetpehykfgttakamlpdvnavdvgdneetsseypvs
lsvsggvlrehhfi
>AJ579768.1
mddtsgqtfvsvnlsdesntastrvenvkseadrleflrkmnpfiidalvrktnyqgarfrarii
gvcvdcgrkhdkalkterkckvnntqsqnevahmlmhdpvrylnkrkarafsnaetfaielvlyt
kekqlavdlaaerekarlarrhpmrspeetpehykfgmtakamlpefnavgvgdnedtsseypvs
lsvsggvlrehhfi
>AJ579769.1
mddtsgqtfisvdlsdesntastevktvsleadrleflrkmnpftidalirktnyqgarfrarii
gvcvdcgrkhnkglrterkckvnntqsqnevahmlmhnpveylnkgkarafsnaemfaidlvmyt
kekqsavnlaaerektrlarrhpmrspeetpehykfgmtakatlpdinavdvgdnedtsseypvs
lsvsngvlrehhfi
>AJ579770.1
mdstsgqtfvsvnlsdesntastavrtvsseadrleflrkmnpfvidalvrktsyqgarfrakii
gvcvdcgrkhdkglkterkckvnntqsqnevahmlmhdpvkylnkrkarafsnaemfaidlvmyt
kekqlavnlaaerektrlarrhpmrspeetpehykfgmtakamlpdinavdvgdnedtsseypvs
lsvsdgvlrehhfi
>AJ579771.1
mdstsgqtfvsvnlsdesntastavrtvsseadrleflrkmnpfiidalvrktnyqgarfrakii
gvcvdcgrkhdkglkterkckvnntqsqnevahmlmhdpvkylnkgkarafsnaemfaidlvmyt
kekqlavnlaaerektrlarrhpmrspeetpefykfgitakamlpninavdvgdnedtsseypvs
lsvsdgvlrehhfi
>AJ579772.1
mdntsgqtfvsvnlsdesntattdvkpvsseadrldflqkmnpiiidalirknsyqgarfrarii
gvcvdcgrkhdkglkterkckvnntqsqnevahmlmhdpvkylnkrkarafsnaeifaidlvmyt
kerqlaidlaaerektrlarrhpmrspeetpeyykfgrtakamlpdinavdvgdneetsseypvs
lsvsggvlrehhfi
>AJ579773.1
mddtsgqtfisvnlsdesntastevktvsleadrleflrkmnpfiidalirktsyqgarfrarii
gvcvdcgrkhdkasrterkckvnntqsqnevahmlmhdpvkylnkrearafsnaemfaidlvmht
kerqlavdlaaerektrlarrhpmrspeeapehykfgmtakamlpninaidvgdnedtsseypvs
lsvsggvlrehhfi
>AJ579774.1
mgdtsgqtfisvnlsdesntasteikavsseadrleflrkmnpfiidalirknsyqgarfragii
gvcvdcgrkhdkasrterkckvnntqsqnevahmlmhdpviylnkrkarafsnaemfaidlvmht

kerqlavdlaaerektrlarrhpmrspeetpehykfgmtakamlpdinavdvgdnedtsseypvs
lsvsggvlrehhfi
>AJ579776.1
mddtsgqtfisvnlsdesntasteiktvsseadrleflrkmnpfiidalirknsyqgarfrarii
gvcvdcgrkhdkasrterkckvnntqsqnevahmlmhdpvkylnkrkarafsnaemfaidlvmyt
kerqlavdlaaerektrlarrhpmrspeetpehykfgmtakamlpdinavdvgdnedtsseypvs
lsvsggvlrehhfi
>AJ579777.1
mddtsgqtfisvnlsdesntasteiktvsseadrleflrkmnpfiidalirknsyqgarfrarii
gvcvdcgrkhdkasrterkckvnntqsqnevahmlmhdpvkylnkrkarafsnaemfaidlvmht
kerqlavdlaaerektrlarrhpmrspeetpehykfgmtakamlpdinavdvgdnedtsseypvs
lsvsggvlrehhfi
>JN384022.1
mddtsgqtfisvnlsdesntasteikavsseadrleflrkmnpfiidalirknsyqgarfrarii
gvcvdcgrkhdkasrterkckvnntqsqnevahmlmhdpvkylnkrkarafsnaemfaidlvmht
kerqlavdlaaerektrlarrhpmrspeetpehykfgmtakamlpdinavdvgdnedtsseypvs
lsvsggvlrehhfil
>JN384024.1
mddtsgqtfvsvnlsdesntastkvenvkseadrleflrkmnpfivdalvrktnyqgarfrarii
gvcvdcgrkhdkalkterkckvnntqsqnevahmlmhdpvkylnkrkarafsnaemfaielvsyt
kerqlavdlaaerektrlarrhpirspeetpehykfgmtakamlpdinavdvgdneetsseypvs
lsvsggvlrehhfil
>JQ039913.1
mdntsgqtfvsvnlsdesntastkvenvnseadrleflrkmnpviidalvrktnyqgarfrarii
gvcvdcgrkhdkalkterkckvnntqsqnevahmlmhdpvkylnkrkarafsnaemfaielvlyt
kerqlavdlaaerektrlarrhpmrspeetpehykfgmtakamlpdinaadvgdseetsseypvs
lsvsggvlrehhfi
>JQ819943.1
mndtsgqtfvsvnlsdesntaisrvenvksetdrleflrkmnpliidalvrktnyqgarfrarii
gvcvdcgrkhdkalkterkckvnntqsqnevahmlmhdpvkylnkrkarafsnaemfaielvlyt
kekqlavdlaaerektrlarrhpmrspeetpehykfgmtakamlpelnavgvgdnedtsseypvi
lsvsggvlre
>JQ819944.1
mddasgqtfisvnlsdesntastevktvsleadrleflrkmnpfiidtlirktnyqgarfrarii
gvcvdcgrkhdkasrterkckvnntqsqaevahmlmhdpvkylnkrkarafsnaemfaidlvmht
kerqlavdlaaerektrlarrhpmrspeetpehykfgitakamlpdinaidvgdnedtsseypvf
lsvsggvlrk
>JQ819945.1
mndtsgqtfvsvnlsdesntaisrvenvkseadrleflrkmnpliidalvrktnyqgarfrarii
gvcvdcgrkhdkalkterkckvnntqsqnevahmlmhdpvkylnkrkarafsnaemfaielvlyt
kekqlavdlaaerektrlarrhpmrspeetpehykfgmtakamlpelnavgvgdnedtsseypvs
lsvsggvlre
>JQ819946.1
mndtsgqtfvsvnlsdesntaisrvenvkseadrleflrkmnpliidalvrktnyqgarfrarii

gvcvdcgrkhdkalkterkckvnntqsqnevahmlmhdpvkylnkrkarafsnaemfaielvlyt
kekqlavdlaaerektrlarrhpmrspeetpehykfgmtakailpelnavgvgdnedtsseypvs
lsvsggvlre
>JQ819947.1
mndtsgqifvsvnlsdesntaisrvenvkseadrleflrkmnpliidalvrktnyqgarfrarii
gvcvdcgrkhdkalkterkckvnntqsqnevahmlmhdpvkylnkrkarafsnaemfaielvlyt
kekqlavdlaaerektrlarrhpmrspeetpehykfgmttkamlpelnavgvgdnedtsseypvs
lsvsggvlre
>JQ819948.1
mndtsgqtfvsvnlsdesntaisrvenvkseadrleflrkmnpliidalvrktnyqgarfrarii
gvcvdcgrkhdkalkterkckvnntqsqnevahmlmhdpvkylnkrkarafsnaemfaielvlyt
kekqlavdlaaerektrlarrhpmrspeeapehykfgmtakamlpelnavgvgdnedtssefpif
lsvsggvlre
>JQ819952.1
mndtsgqtfvsvnlsdesntaisrvknvkseadrleflrkmnpliidalvgktnyqgarfrarii
gvcvdcgrkhdkavrterkckvnntqsqnevahmlmhdpvnylnkrkarafsnaemfaielflyt
kekqlavdlaaerektrlarrhpmrspeetpehykfgmtakailpelnavgvgdnedtssdfpvf
lsvsggvlre
>JQ819954.1
mndtsgqtfvsvnlsdesntaisrvenvkseadrleflrkmnpliidalvrktnyqgarfrarii
gvcvdcgrkhdkalkterkckvnntqsqnevahmlmhdpvkylnkrkarafsnaemfaielvlyt
kekqlavdlaaerektrlarrhpmrspeetpehykfgitakamlpdinaidvgdnedtsseypvs
lsvsggvlre
>JQ819955.1
mddtsgqtfisvnlsdesntastevktvsleadrleflrkmnpftidalirktnyqgarfrarii
gvcvdcgrkhdkasrterkckvnntqsqaevahmlmhdpvkylnkrkarafsnaemfaidlvmht
kerqlavdlaaerektrlarrhpmrspeetpehykfgitakamlpdinaidvgdnedtsseypvs
lsasggvlre
>JQ819960.1
mddtsgqtfisvnlsdesntastevktvsleadrleflrkmnpftidalirktnyqgarfrarii
gvcvdcgrkhdkasrterkckvnntqsqaevahmlmhdpvkylnkrkarafsnaemfaidlvmht
kerqlavdlaaerektrlarrhpmrspeetpehykfgitakamlpdinaidvgdnedtsseypvs
lsvsggvlre
>JQ819963.1
mvdtsgqtfisvnlsdesntastevktvsleadrleflrkmnpfnidalirktnyqgarfrarii
gvcvdcgrkhdkasrterkckvnntqsqaevahmlmhdpvkylnkrkarafsnaemfaidlvmht
kerqlavdlaaerektrlarrhpmrspeetpehykfgitakamlpdinaidvgdnedtsseypvs
lsvsggvlre
>JQ819964.1
mddtsgqtfisvnlsdesntastevktvsleadrlddlrkmnpfiidalirktnyqgarfrarii
gvcvdcgrkhdkasrterkckvnntqsqaevahmlmhdpvkylnkrkarafsnaemfaidlvmht
kerqlavdlaaereetrlarrhpmrspeetpehykfgitakamlpdinaidvgdnedtsseypvs
lsvsggvlre
>JQ819965.1

mddtsgqtfisvnlsdesntastevktvsleadrleflrkmnpftidalirktnyqgarfrarii
gvcvdcgrkhdkasrterkckvnntqsqaevahmlmhdpvkylnkrkarafsnaemfaidlvmht
kerqlavdlaaerektrlarrhpmrspeetpehykfgitakamlpdinaidvgdnedtsseypvs
lsvpggvlre
>JQ819967.1
mddtsgqtfisvnlsdesntastevktvsleadrleflrkmnpftidalirktnyqgahfrarii
gvcvdcgrkhdkasrterkckvnntqsqaevahmlmhdpvkylnkrkarafsnaemfaidlvmht
kerqlavdlaaerektrlarrhpmrspeetpehykfgitakamlpginaidvgdnedtsseypvs
lsvsggvlre
>JQ819968.1
mddtsgqtfisvnlsdesntastevktvsleadrleflrkmnpfiidalirktnyqgarfrarii
gvcvdcgrkhdkasrterkckvsntqsqaevahmlmhdpvkylnkrkarafsnaerfaidlvmht
kerqlavdlaaerektrlarrqpmrspeetpehykfgitakamlpdinaidvgdnedtsseypvs
lsvsggvlre
>JQ819969.1
mddtsgqtfisvnlsdenntastevktvsleadrleflrkmnpfiidalirktnyqgarfrarii
gvcvdcgrkhdkasrterkckvnntqsqaevahmlmhdpvkylnkrkarafsnaemfaidlvmht
kerqlavdlaaerektrlarrhpmrspeetpehykfgitakamlpdinaidvgdnedtsseypvs
lsvsggvlre
>JQ819971.1
mddtsgqtfisvnlsdesntastevktvsleadrleflrkmnpfiidalirktnyqgarfrarii
gvcvdcgrkhdkasrterkckvnntqsqaevahmlmhdpakylnkrkarafsnaemfaidlvmht
kerqlavdlaaerektrlarrhpmrspeetpehykfgitakamlpdinaidvgdnedtsseypvs
lsvsggvlre
>JQ819973.1
mddtsgqtfisvnlsdesntastevktvsleadrleflrkmnpftidalirktnyqgarfrarii
gvcvdcgrkhdkasrterkckvnntqsqaevahmlvhdpvkylnkrkarafsnaemfaidlvmht
kerqlavdlaaerektrlarrhpmrspeetpehykfgitakamlpdinaidvgdnedtsseypvs
lsvsggvlre
>JQ819974.1
mddtsgqtfisvnlsdesntastevktvsldadrleflrkmnpfiidalirktnyqgarfragii
gvcvdcgrkhdkasrterkckvnntqsqaevahmlmhdpvkylnkrkarafsnaemfaidlvmht
kerqlavdlaaerektrlarrhpmrspeetpehykfgitakamlpdinaidvgdnedtsseypvs
lsvsggvlre
>JQ819976.1
mddtsgqtfisvnlsdesntastevktvsleadrleflrkmnpfiinalirktnyqgarfrarii
gvcvdcgrkhdkasrterkckvnntqsqaevahmlmhdpvkylnkrkarafsnaemfaidlvmht
kerqlavdlaaerektrlarrhpmrspeetpehykfgitakamlpdinaidvgdnedtsseypvs
lsvsggvlre
>JQ819979.1
mddtsgqtfisvnlsdesntastevktvsleadrleflrkmnpftidalirktnyqgarfrarii
gvcvdcgrkhdkasrterkckvnntqsqaevahmlmhdpvkylnkrkarafsnaemfaidlvmht
kerqlavdlaaerektrlarrhpmrspeetpehyefgitakamlpdinaidvgdnedtsseypvs
lsvsggvlre

>JQ819981.1
mddtsgqtfisvnlsdesntastevktvsleadrleflrkmnpftidalirktnyqgarfrarii
gvcvdcgrkhdkasrterkckvnntqsqaevahmlmhdpvkylnkrkarafsnaemfaidlvmht
kerqlavdlaaerektrlarrhpmrspeetpehykfgitakamlpdinaidvgdnedtsseypvs
lsvsggvvre
>JQ819983.1
mddtsgqtfisvnlsdesntastevktvsleadrleflrkmnpfiidalirktnyqgarfrarii
gvcvdcgrkhdkasrterkckvnntqsqaevahmlmhdpvrylnkrktrafsnaemfaidlvmht
kerqlavdlaaerektrlarrhpmrspeetpehykfgitakamlpdinaidvgdnedtssgypvs
lsvsggvlre
>JQ819987.1
mddtsgqtfisvnlsdesntastevktvsleadrleflrkmnpftidalirktnyqgarfrarii
gvcvdcgrkhdkasrterkckvnntqsqaevahmlmhdpvkylnkrkarafsnagmfaidlvmht
kerqlavdlaaerektrlarrhpmrspeetpehykfgitakamlpdinaidvgdnedtsseypvs
lsvsggvlre
>JQ819988.1
mddtsgqtfisvnlsdesntastevktvsleadrleflrkmnpfiidalirktnyqgarfrarii
gvcvdcgrkhdkasrterkckvnntqsqaevahmlmhdpvkylnkrkarafsnaemfaidlvmht
kerqlavdlaaerektrlarrhpmrspeetpehykfgitakamlpdinaidvgdnedtsseypvs
lsvscgvlre
>JQ819991.1
mddtsgqtfisvnlsdesntastevktvsleadrleflrkmnpftidalirktncqgarfrarii
gvcvdcgrkhdkasrterkckvnntqsqaevahmlmhdpvkylnkrkarafsnaemfaidlvmht
kerqlavdlaaerektrlarrhpmrspeetpehykfgitakamlpdinaidvgdnedtsseypvs
lsvsggvsre
>JQ819996.1
mddtsgqtftsvnlsdesntastevktvsleadrleflrkmnpfiidalirktnyqgarfrarii
gvcvdcgrkhdkasrterkckvnntqsqaevahmlmhdpvkylnkrkarafsnaemfaidlvmht
kerqlavdlaaerektrlarrhpmrspeetpehykfgitakamlpdinaidvgdnedtsseylvs
lsvsggvlre
>JQ819997.1
mddtsgqtftsvnlsdesntastevktvsleadrleflrkmnpfiidalirktnyqgarfrarii
gvcvdcgrkhdkasrterkckvnntqsqaevahmlmhdpvkylnkrkarafsnaemfaidlvmht
kerqlavdlaaerektrlarrhpmrspeetpehykfgitakamlpdinaidvgdnedtsseypvs
lsvsggvlre
>JQ820000.1
mddtsgqtftsvnlsdesntasteaktvsleadrleflrkmnpfiidalirktnyqgarfrarii
gvcvdcgrkhdkasrterkckvnntqsqaevahmlmhdpvkylnkrkarafsnaemfaidlvmht
kerqlavdlaaerektrlarrhpmrspeetpehykfgitakamlpdinaidvgdnedtsseypvs
lsvsggvlre
>JQ820001.1
mddtsgqtfisvnlsdesntastevktvsleadrleflrkmnpfiidalirktnyqgarfrarii
gvcvdcgrkhdkasrterkckvnntqsqaevahmlmhdpvkylnkrkarafsnaemfaidlvmht
kerqlavdlaaerektrlarrhpmrspeetpehykfgitakamlpdinaidvgdnedtsseypvs

lsvsggvlre
>JQ820003.1
mddtsgqtfisvnlsdesntastevktvsleadrleflqkmnpftidalirktnyqgarfrarii
gvcvdcgrkhdkasrterkckvnntqsqaevahmlmhdpvkylnkrkarafsnaemfaidlvmht
kerqlavdlaaerektrlarrhpmrspeetpehykfgitakamlpdinaidvgdnedtsseypvs
lsvsggvlre
>JQ820007.1
mddtsgqtfisvnlpdesntastevktvsleadrleflrkmnpfiidalirktnyqgarfrarii
gvcvdcgrkhdkasrterkckvnntqsqaevahmlmhdpvkylnkrkarafsnaemfaidlvmht
kerqlavdlaaerektrlarrhpmrspeetpehykfgitakamlpdinaidvgdnedtsseypvs
lsvsggvlre
>JQ820008.1
mddtsgqtfisvnlsdesntastevktvsleadrleflrkmnpfiidalirktnyqgarfrarii
gvcvdcgrkhdkasrterkckvnntqsqaevahmlvhdpvkylnkrkarafsnaemfaidlvmht
kerqlavdlaaerektrlarrhpmrspeetpehykfgitakamlpdinaidvgdnedtsseypvs
lsvsggvlre
>JQ820010.1
mgdtsgqtfisvnlpdesntastevktvsleadrweflrkmnpfiidalirktnyqgarfrarii
gvcvdcgrkhdkasrtepkckvnntqsqaevahmlmhdpvkylnkrkarafsnaemfaidlvmht
kerqlavdlaaerektrlarrhpmrspeetrehykfgitakpmlpdinaidvgdnedtsseypvs
lsvsggvlre
>JQ820013.1
mddtsgqtfisvnlsdesntastevktvsleadrleflrkmnpfidalirktnyqgarfrariig
vcvdcgrkhdkasrterkckvnntqsqaevahmlmhdpvkylnkrkarafsnaemfaidlvmhtk
erqlavdlaaerektrlarrhpmrspeetpehykfgitanamlpdinavdvgdnedtsseypvsl
svsggvlre
>KC202886.1
mdstsgqtfvsvnlspesntastavrtvsseadrleflrkmnpfiidalvrktnyqgarfrakii
gvcvdcgrkhdkglkterkckvnntqsqnevahmlmhdpvkylnkgkarafsnaemfaidlvmyt
kekqlavnlaaerektrlarrhpmrspeetpefykfgitakamlpninavdvgdnedtsseypvs
lsvsdgvlrehhfil
>KC202887.1
mnpfvidalirktsyqgarfrariigvcvdcgrkhekgsrterkckvnntqsqnevahmlmhdpv
kylskrkarafsnaemfaidlvmytkerqlavdlaaerektrlarkhpmrspeetpehykfgmta
kamlpdinivdvgettktlfgdgvslsvsggvlrehhfil
>KC202888.1
mddtsgqtfisvnlsdesntastevktvsseaerleflrkmnpfvidalirktsyqgarfrarii
gvcvdcgrkhekgsrterkckvnntqsqnevahmlmhdpvkylskrkarafsnaeiaidlvmytk
erqlavdlaaerektrlalntqcvlrkklrnikfgmtakamlpdinivdvgdnedtsseyrkses
fwrvs
>KC202889.1
mddtsgqkgtpfrfcepfvtastevktvsseyrtvsseadtlgiltkneplyhrrfgtentvsrv
knslsrknnrsmrrlgrkhdkglkterkckvnntqsqnevahmlmhdpvkylnkgkarafsnaem
faidlvmytkekqlavnlaaerektrlarrhpmrspeetpefykfgitakamlpninavdvgdne

dtsseteesev
>KC202908.1
mdntsgqtfisvnlsdesntastevktvsseadrleflrkmnpfvidalirktsyqgarfrarii
gvcvdcsrkhdkgsrterkckvnntqsqnevahmlmhdpvkylskrkarafsnaemfaidlvmyt
kerqlavdlaaerektrlarkhpmrspeetpehykfgmtakamlpdinvvdvgdnedtsseypvs
lsisggvlrehhfirsnh
>KC562141.1
mdytsgqtfvsvnlsdesntastevktvsseadrleflqkmnpfvidalirktsyqgarfrarii
gvcvdcgrkhnkglkterkckvnntqsqnevahmlmhdpvkylnegkarafsnaemfaidlvmyt
kerqlavnltaerektrlarrhpmrspeetpehykfgmtakvmlpdidavdvddnedtlsecpvn
lsvsggvlrehhfi
>KC562142.1
mddtsgqtfvsvnlsdesntastevktvsleadrleflrkmnpfiidalirktsyqgarfrarii
gvcvdcgrkhdkasrterkckvnntqsqnevahmlmhdpvkylnkrkarafsnaemfaidlvmht
kerqlavdlaaerektrlarrhpmrspeetpehykfgmtakamlpdtnaidvgdnedtsseypvs
lsvsggvfrehhfi
>KC562143.1
mdntsgqtfvsvnlsdesntastevktvsleadrleflrkmnpliidalirktsyqgarfrarii
gvcvdcgrkhdeasrterkckvnntqsqnevahmlmhdpvkylnkrkarafsnaemfaidlvmht
kerqlavdlaaerektrlarrhpmrspeetpehykfgmaakamlpdtnaidvgdnedtsseypvs
lsvsggvlrehhfi
>KC562144.1
mdytsgqtfvsvnlsdesntastevktvnleadrleflrkmnpfiidalirktsyqgarfrarii
gvcvdcgrkhdkasrterkckvnntqsqnevahmlmhdpvkylnkrkarafsnaemfaidlvmht
kerqlavdlaaerektrlarrhpmrspeetpehykfgmtakamlpdtnaidvgdnedtsseypvs
lsvsggvfrehhfi
>KC562145.1
mddtsgqtfvsvnlsdesntastevktvsseadrleflqkmnpfvidalirktsyqgarfrarii
gvcvdcgrkhnkglkterkckvnntqsqnevahmlmhdpvkylnegkarafsnaemfaidlvmyt
kerqlavnltaerektrlarrhpmrspeetpehykfgmtakvmlpdidavdvddnedtlsecpvn
lsvsggvlrehhli
>KC562146.1
mdytsgqtfvsvnlsdesntastevktvsseadrleflqkmnplvidalirktsyqgarfrarii
gvcvdcgrkhnkglkterkckvnntqsqnevahmlmhdpvkylnegkarafsnaemfaidlvmyt
kekqlavnltaerektrlarrhpmrspeetpehykfgmtakamlpgidvvdvddnedtlseypvn
lsvsggvlrehhfi
>KC577594.1
mdytsgqtfisvnlsdesntasteikavsseadrleflrkmnpfiidalirknnyqgarfrarii
gvcvdcgrkhdkasrterkckvnntqsqnevahmlmhdpvkylnkrkarafsnaemfaidlvmlt
kerqlavdlaaerektrlarrhpmrspeetpehykfgmtakamlpdtnavdvgdnedtsseypvs
lsvsggvlrehhfi
>AY652901.1
mdstsgqtfvsvnlsdesntastavrtvsseadrleflrkmnpfvidalvrktsyqgarfrakii
gvcvdcgrkhdkglkterkckvnntqsqnevaymlmhdpvkylnkgkarafsnaemfaidlvmyt

kekqlavslaaerektrlarrhpmrspeetpehykfgmtakamlpdtnavdvgdnedtsseypvs
lsvsdgvlrehhfi

>AY652902.1
mddtsgqtfvsvnlsdesntastrvenvkseadrleflrkmnpfiidalvrktnyqgarfrarii
gvcvdcgrkhdkalkterkckvnntqsqnevahmlmhdpvkylnkrkarafsnaetfaielvlyt
kekqlavdlaaerekarlarrhpmrspeetpehykfgmtakamlpefnavgvgdiedtsseypvs
lsvsggvlrehhfi

>AY652904.1
mdstngqtfvsvnlsdesntastavrtvsseadrleflrkmnpfiidalirktsyqgarfrarii
gvcvdcgrkhdkglrterkckvnntrsqnevahmlmhdpvkylnegkarafsnaemfaidlvmyt
kekqladdlaaerektrlarrhpmrspeetpehykfgmtakamipdidvvyvgdnedtsseypvs
lgvsdgvlrehhfv

>AY652906.1
mddtngqtfvsvnlsdesntastkvenvkseadrleflrkmnpfivdalvrktnyqgarfrarii
gvcvdcgrkhdkalkterkckvnntqsqnevahmlmhdpvkylnkrkarafsnaemfaielvsyt
kerqlavdlaaerektrlarrhtirspeetpehykfgmtakamlpninavdvgdneetsseypvs
lsvsggvlrehhfi

>KF724132.1
mddtsgqtfvsvnlsdesntastkvenvkseadrleflrkmnpfivdalvrktnyqgarfrarii
gvcvdcgrkhdkalkterkckvnntqsqnevahmlmhdpvkylnkrkarafsnaemfaielvfyt
kerqlavdlaaerektrlarrhpirspeetpehykfgmtakamlpdinavdvgdneetsseypvs
lsvsggvlrehhfi

>KF724135.1
mddtsgqtfvsvnlsdesntastkvenvkseadrleflrkmnpfivdalvrktnyqgarfrarii
gvcvdcgrkhdkalkterkckvnntqsqnevahmlmhdpvkylnkrkarafsnaemfaielvlyt
kerqlavdlaaerektrlarrhpirspeetpehykfgmtakamlpdinavdvgdneetsseypvs
lsvsggvlrehhfi

>KF724136.1
mddtsgqtfvsvnlsdesntastkvenvkseadrleflrkmnpfivdalvrktnyqgarfrarii
gvcvdcgrkhdkalkterkckvnntqsqnevahmlmhdpvkylnkrkarafsnaemfaielvlyt
kerqlavdlaaerektrlasrhpirspeetpehykfgmtakamlpdinavdvgdneetsseypvs
lsvsggvlrehhfi

>KF724137.1
mddtsgqtfvsvnlsdesntastkvenvkseadrleflrkmnpfivdalvrktnyqgarfrarii
gvcvdcgrkhekalkterkckvnntqsqnevahmlmhdpvkylnkrkarafsnaemfaielvfyt
kerqlavdlaaerektrlarrhpirspeetpehykfgmtakamlpdinavdvgdneetsseypvs
lsvsggvlrehhfi

>KF724141.1
mddtsgqtfvsvnlsdesntastkvenvkseadrldflrkmnpfivdalvrktnyqgarfrarii
gvcvdcgrkhdkalkterkckvnntqsqnevahmlmhdpvkylnkrkarafsnaemfaielvlyt
kerqlavdlaaerektrlarrhpirspeetpehykfgmtakamlpdinavdvgdneetsseypvs
lsvsggvlrehhfi

>KF724146.1
mddtsgqtfisvnlsdesntastevktvsseadrleflrkmnpfvidalirktsyqgarfrarii

gvcvdcgrkhekgsrterkckvnntqsqnevahmlmhdpvkylskrkarafsnaemfaidlvmyt
kerqlavdlaaerektrlarkhpmrspeetpehykfgmtakamlpdinvvnvgdnedtsseypvs
lsvsggvlrehhfi
>KC774010.1
mddtsgqtfisvnlsdesntasteikavsseadrleflrkmnpfiidalirknsyqgarfrarii
gvcvdcgrkhdkasrterkckfnntqsqnevahmlmhdpvkylnkrkarafsnaemfaidlvmht
kerqlavdlaaerektrlarrhpmrspeetpehykfgmtakamlpdinavdvgdnedtsseypvs
lsvsggvlrehhfi
>KC774011.1
mddtsgqtfvsvnlsdesntastkvenvkseadrldflrkmnpfivdalvrktnyqgarfrarii
gvcvdcgrkhdkalkterkckvyntqsqnevahmlmhdpvkylnkrkarafsnaemfaielvlyt
kerqlavdlaaerektrlarrhpirspeetpehykfgmtakamlpdinavdvgdneetsseypvs
lsvsggvlrehhfi
>KC774012.1
mddtsgqtfisvnlsdesntasteikavsseadrleflrkmnpliidalirknsyqgarfrarii
gvcvdcgrkhdkasrterkckvnntqsqnevahmlmhdpvkylnkrkarafsnaemfaidlvmht
kerqlavdlaaerektrlarrhpmrspeetpehykfgmtakamlpdinavdvgdnedtsseypvs
lsvsggvlrehhfi
>KC774013.1
mddtsgqtfvsvnlsdesntastkvenvkseadrldflrkmnpfivdalvrktnyqgarfrarii
gvcvdcgrkhdkalkterkckvnntqsqnevahmlmhdpvkylnkrkarafsnaemfaielvlyt
kerqlavdlaaerektrlarrhpirspeetpehykfgmtakamlpdinavdvgdneetsseypvs
lsvsggvlrehhfi
>KC774014.1
mddtsgqtfisvnlsdesntasteikavsseadrleflrkmnpfiidalirknsyqgarfrarii
gvcvdcgrkhdkasrterkckvnntqsqnevahmlmhdpvkylnkrkarafsnaemfaidlvmht
kerqlavdlaaerektrlarrhpmrspeetpehykfgmtakamlpdinavdvgdnedtsseypvs
lsvsggvlrehhfi
>KC774015.1
mddtsgqtfisvnlsdesntasteikavsseadrleflrkmnpfiidalirknsyqgarfrarii
gvcvdcgrkhdkasrterkckvnntqsqnevahmlmhdpvkylnkgkarafsnaemfaidlvmht
kerqlavdlaaerektrlarrhpmrspeetpehykfgmtakamlpdinavdvgdnedtsseypvs
lsisggvlrehhfi
>KC774016.1
mdntsgqtfvsvnlsdesntattdvkpvsseadrldflqkmdpiiidalirknsyqgarfrarii
gvcvdcgrkhdkglkterkckvnntqsqnevahmlmhdpvkylnkrkarafanaeifaidlvmyt
kerqlavdlaaerektrlarrhpmrspeetpehykfgrtakamlpdvnavdvgdneetsseypvs
lsvsggvlrehhfi
>KC774017.1
mddtsgqtfvsvnlsdesntastkvenvkseadrleflrkmnpfivdalvrktnyqgarfrarii
gvcvdcgrkhdkalkterkckvnntqsqnevahmlmhdpvkylnkrkarafsnaemfaielvsyt
kerqlavdlaaerektrlarrhpirspeetpehykfgmtakamlpdinavdvgdneetsseypvs
lsvsggvlrehhfi
>KF913715.1

mdytsgqtfvsvnlsdesntastevktvsseadrleflqkmnplvidalirktsyqgarfrarii
gvcvdcgrkhnkglktereckvnntqsqnevahmlmhdpvkylnegkarafsnaemfaidlvmyt
kekqlavnltaerektrlarrhpmrspeetpehykfgmtakamlpgidvvdvddnedtlseypvn
lsvsggvlrehhfi
>KF913716.1
mdntsgqtfisvnlsdesntasteikavsseadrleflrkmnpfiidalirknnyqgarfrarii
gvcvdcgrkhdkasrterkckvnntqsqnevahmlmhdpvkylnkrkarafsnaemfaidlvmlt
kerqlavdlaaerektrlarrhpmrspeetpehykfgmtakamlpdtnavdvgdnedtsseypvs
lsvsggvlrehhfi
>KF913717.1
mdntsgqtfisvnlsdesntastevetvsleadrleflrkmnpfiidalirrtsyqgarfrarii
gvcvdcgrkhdeasrterkckvnntqsqnevahmlmhdpvkylnkrkarafsnaemfaidlvmht
kerqlavdlaaerektrlarrhpmrspeetpehykfgmaakamlpdtnaidvgdnegtsseypvs
lsvsggvlrehhfi
>KF913718.1
mdytsgqtfnsvnlsdesntastevktvsleadrleflrkmnsfiidalirktsyqgarfrarii
gvcvdcgrkhdkasrterkckvnntqsqnevahmlmhdpvkylnkrkarafsnaemfaidlvmht
kerqlavdlaaerektrlarrhpmrspeetpehykfgmtakamlpdtnaidvgdiedtsseypvs
lsvsggvlrehhfi
>AY750732.1
mddtsgqtfisvnlsdesntastevktvsseadrleflrkmnpfiidalirktsyqgarfrarii
gvcvdcgrkhdkalrterkckvnntqsqnevahmlmhdpveylnkrkarafskaemfaidlvmht
kerqlavdlaaerektrlarrhpmrspeetpehykfgmtakamlpdinaidvgdnedtsseypvs
lsvsggvlrehhfil
>AY750733.1
mddtsgqtfisvnlsdesntasteikavsseadrleflrkmnpftidalirknsyqgarfrarii
gvcvdcgrkhdkasrterkckvtntqsqnevahmlmhdpvkylnerkarafsnaemfaidlvmht
kerqlavdlaaerektrlarrhpmrspeetpehykfgmtakamlpdinavdvgdnedtsseypvs
lsvsggvlrehhfil
>AY750735.1
mddtsgqtfisvnlsdesntastevktvsseadrleflrkmnpfiidalirktsyqgarfrarii
gvcvdcgrkhdkgsrterkckvnntqsqnevahmlmhdpvkylnkrkarafsnaemfaidlvmht
kekqlavdlaaerektrlarrhpmrspeetpeyykfgmtakamlpdinaidvgdnedtssdypvs
lsvsggvlrehhfil
>AY750736.1
mndtsgqtfvsvnlsdesntastrvenvkseadrldflrkmnpliidalvrktnyqgarfrarii
gvcvdcgrkhdkalkterkckvnntqsqnevahmlmhdpvkylnkrkarafsnaemfaielvlyt
kekqlavdlaaerektrlarrhpmrspeetpehykfgmtakvmlpelnavgvgdnedtsseypvs
lsvsggvlrehhfil
>AY750737.1
mdstsgqtfvsvnlsdesntastavrtvsseadrleflrkmnpfiidalvrktnyqgarfrakii
gvcvdcgrkhdkglkterkckvnntqsqnevahmlmhdpveylnkgkarafsnaemfaidlvmyt
kekqlavnlaaerektslarrhpmrspeetpefykfgitakamlpninavdvgdnedtsseypvs
lsvsdgvlrehhfil

>AY750738.1
mddtsgqtfisvnlsdesntasteikavsseadrleflrkmnpfiidalirknsyqgarfrerii
gvcvdcgrkhdkasrterkckvnntqsqnevahmlmhdpviylnkrkarafsnaemfaidlvmht
kerqlavdlaaerektrlarrhpmrspeetpehykfgmtakamlpdinavdvgdnedtsseypvs
lsvsggvlrehhfil
>AY750739.1
mddtsgqtfisvnlsdesntastevktvsseadrleflrkmnpfiidalirktsyqgarfrarii
gvcvdcgrkhdkglkterkckvnntqsqnevahmlmhdpvkylsegkarafsnaemfaidlvmyt
kekqlavnltaerektrlarrhpmrspeetpehykfgmtakamlpdinavvvgdnedtsseypvs
lsisdgvlrehhfil
>AY750740.1
mddtsgqtfisvnlsdesntastevktvsseaerleflrkmnpfvidalirktsyqgarfrarii
gvcvdcgrkhekgsrterkckvnntqsqnevahmlmhdpvkylskrkarafsnaemfaidlvmyt
kerqlavdlaaerektrlarkhpmrspeetpehykfgmtakamlpdinivdvgdnedtsseypvs
lsvsggvlrehhfil
>AY750741.1
mddtsgqtfvsvnlsdesntastavrtvsseadrleflrkmnpfildalirknnyqgarfrarii
gvcvdcgrkhdkasrterkckvnntqsqnevahmlmhdpvkylnkrkarafsnaemfaidlvmht
kerqlavdlaaerektrlarrhpmrspeetpehykfgitakamlpdinavdvgdnedtsseypvs
lsvsdgvlrehhfil
>AY750742.1
mddtsgqtfisvnlsdesntasteikavsseadrleflrkmnpfiidalirknnyqgarfrarii
gvcvdcgrkhdkasrterkckvnntqsqnevahmlmhdpvkylnkrkarafsnaemfaidlvmht
kerqlavdlaaerektrlarrhpmrspeetpehykfgmtakamlpdinavdvgdnedtsseypvs
lsvsggvlrehhfil
>AY750743.1
mdstsgqtfvsvnlsdesntastavetvnseadrleflrkmnpfiidalvrktnyqgarfrakii
gvcvdcgrkhdkglkterkckvnntqsqnevahmlmhdpveylnkgkarafskaemfaidlvmyt
kekqlavnlaaerektrlarrhpmrspeetpehykfgmtakamlpdinavdvgdnedtsseypvs
lsvsdgvlrehhfil
>AY750744.1
mddtsgqtfisvnlsdesntasteikavsseadrleflrkmnpftidalirknsyqgarfrarii
gvcvdcgrkhdkasrterkckvnntqsqnevahmlmhdpvkylnerkarafsnaemfaidlvmht
kerqlavdlaaerektrlarrhpmrspeetpehykfgmtakamlpninavdvgdnedtsseypvs
lsvsdgvlrehhfil
>AY750745.1
mdstsgqtfisvnlsdesntastevktvsseadrleflrkmnpfvidalirktsyqgarfrarii
gvcvdcgrkhekgsrterkckvnntqsqnevahmlmhdpvkylskrkarafsnaemfaidlvmyt
kerqlavdlaaerektrlarkhpmrspeetpehykfgmtakamlpdinvvnvgdnedtsseypvs
lsvsggvlrehhfil
>AY750746.1
mddtsgqtfisvnlsdesntastevktvsseadrleflrkmnpfiidalirktsyqgarfrarii
gvcvdcgrkhdkgsrterkckvnntqsqnevahmlmhdpvkylnkrkarafsnaemfaidlvmht
kekqlavdlaaerektrlarrhpmrspeetpeyykfgmtakamlpdinaidvgdnedtsseypvs

lsvsggvlrehhfil
>AY750747.1
mndtsgqtfvsvnlsdesntastrvenvkseadrldflrkmnpliidalvrktnyqgarfrarii
gvcvdcgrkhdkalkterkckvnntqsqnevahmlmhdpvkylnkrkarafsnaemfaielvlyt
kekqlavelaaerektrlarrhpmrspeetpehykfgmtakvmlpelnavgvgdnedtsseypvs
lsvsggvlrehhfil
>AY750748.1
mdstsgqtfvsvnlsdesntastavrtvsseadrleflrkmnpfvidalvrktsyqgarfrakii
gvcvdcgrkhdkglkterkckvnntqsqnevahmlmhdpvkylnkrkarafsnaemfaidlvmyt
kekqlavnlaaerektrlarrhpmrspeetpehykfgmtakamlpdinavdvgdnedtsseypvs
lsvsdgvlrehhfil
>AY750749.1
mdstsgqtfvsvnlsdesntastavrtvsseadrleflrkmnpfiidalvrktnyqgarfrakii
gvcvdcgrkhdkglkterkckvnntqsqnevahmlmhdpveylnkgkarafsnaemfaidlvmyt
kekqlavnlaaerektrlarrhpmrspeetpefykfgitakamlpninavdvgdnedtsseypvs
lsvsdgvlrehhfil
>AY750750.1
mddtsgqtfisvnlsdesntasteikavsseadrleflrkmnpfiidaltrknsyqgarfrarii
gvcvdcgrkhdkasrterkckvnntqsqnevahmlmhdpviylnkrkarafsnaemfaidlvmht
kerqlavdlaaerektrlarrhpmrspeetpehykfgmtakamlpdinavdvgdnedtsseypvs
lsvsggvlrehhfil
>AY750751.1
mdstsgqtfvsvnlsdesntastavrtvsseadrleflrkmnpfiidalvrktnyqgarfrakii
gvcvdcgrkhdkglkterkckvnntqsqnevahmlmhdpvkylnkgkarafsnaemfaidlvmyt
kekqlavnlaaerektrlarrhpmrspeetpefykfgitakamlpninavdvgdnedtsseypvs
lsvsdgvlrehhfil
>KJ094313.1
mdntsgqtfvsvnlsdesntastevktvssetdrleflqkmnpfvidalirktsyqgarfrarii
gvcvdcgrkhnkglkterkckvnntqsqnevahmlmhdpvkylnegkarafsnaemfaidlvmyt
kerqlavnltaerektrlarrhpmrspeetpehykfgmtakamlpdvdvvnvddnedtlseypvn
lsvsggvlrehhfi
>AY962348.1
mddtsgqtlisvnlsdesntastevktvsseaerleflrkmnpfvidalirktsyqgarfrarii
gvcvdcgrkhekgsrterkckvnntqsqnevahmlmhdpveylskrkarafsnaemfaidlvmyt
kerqlavdlaaerektrlarkhpmrspeetpehykfgmtakamlpdinivdvgdnedtsseypvs
lrvsggvlrehhfil
>AY962349.1
mddtsgqtfvsvnlsdesntastrvenvkseadrleflrkmnpliidalvrktnyqgarfrarii
gvcvdcgrkhdkalkterkckvnntqsqnevahmlmhdpvkylnkrkarafsnaemfaielvlyt
kekqlavdlaaerektrlarrhpmrspeetpehykfgltakamlpelnavgvgdnedtsseypvs
lsvsggvlrehhfil
>AY962350.1
mddtsgqtfisvnlsdesntastevktvsseadrleflrkmnpfiidalirktsyqgarfrarii
gvcvdcgrkhekgsrterkckvnntqsqnevahmlmhdpveylskrkarafsnaemfaidlvmyt

kerqlavdlaaerektrlarkhpmrspeetpehykfgmtakamlpdvnvvdvgdnedtsseypvs
lrvsggvlrehhfil
>AY962351.1
mddtsgqtfvsmnlsdesntastavrtvsseadrleflrkmnpfiidalvrktnyqgarfrakii
gvcvdcgrkhdkglkterkckvnntqsqnevahmlmhdpvkylnkgkarafsnaemfaidlvmyt
kekqlavnlaaerektrlarrhpmrspeetpefykfgitakamlpninavdvgdnedtsseypvs
lsvsggvlrehhfil
>AY962355.1
mddtsgqtlisvnlsdesntastevktvsseaerleflrkmnpfvidalirktsyqgarfrarii
gvcvgcgrkhekgsrterkckvnntqsqnevahmlmhdpveylskrkarafsnaemfaidlvmyt
kerqlavdlaaerektrlarrhpmrspeetpehykfgltakamlpelnavgvgdnedtsseypvs
lsvsggvlrehhfil
>AY962356.1
mddtsgqtfisvnlsdesntastevktvsseadrleflrkmnpfvidalirktsyqgarfrarii
gvcvdcggkhekgsrterkckvnntqsqnevahmlmhdpvkylnkrkarafsnaemfaielvlyt
kerqlavdlaaerektrlarkhpmrspeetpehykfgmtakamlpdvnvvdvgdnedtsseypvs
lsisggvlrehhfil
>AY962357.1
mddtsgqtfvsvnlsdesntastrvenvkseadrleflrkmnpliidalarktnyqgarfrarii
gvcvdcgkkhdkalkterkckvnntqsqnevahmlmhdpvkylnkrkarafsnakmfaielvlyt
kekqlavdlaaerektrlarrhpmrspeetpehykfgltakamlpelnavgvgdnedtsseypvs
lsvsggvlrehhfil
>AY962358.1
mddtsgqtfvsvnlsdesntastrvenvkseadrleflrkmnpliidalvrktnyqgarfrarii
gvcvdcgkkhdkalkterkckvnntqsqnevahmlmhdpvkylnkrkarafsnakmfaielvlyt
kekqlavdlaaerektrlarkhpmrspeetpehykfgmtakamlpdinivdvgdnentsseypvs
lrvsggvlrehhfil
>AY962359.1
mddtsgqtfvsvnlsdesntastavrtvsseadrleflrkmnpfiidalvrktnyqgarfrakii
gvcvdcgrkhdkglkterkckvnntqsqnevahmlmhdpvkylnkgkarafsnaemfaidlvmyt
kekqlavnlaaerektrlarrhpmrspeetpefykfgitakamlpninavdvgdnedtsseypvs
lsvsdgvlrehhfil
>AY962360.1
mddtsgqtfvsvnlsdesntastrvenvkseadrleflrkmnpliidalvrktnyqgarfrakii
gvcvdcgkkhdkalkterkckvnntqsqnevahmlmhdpvkylnkrkarafsnakmfaielvlyt
kekqlavdlaaerektrlarrhpmrspeetpehykfgltakamlpelnavgvgdnkdtsseypvs
lsvsggvlrehhfil
>AY962361.1
mddtsgqtfvsmnlsdesntastavrtvsseadrleflrkmnpfiidalvrktnyqgarfrakii
gvcvdcgrkhdkglkterkckvnntqsqnevahmlmhdpvkylnkgkarafsnaemfaidlvmyt
kekqlavnlaaerektrlarrhpmrspeetpefykfgitakamlpninavdvgdnedtsseypvs
lsvsdgvlrehhfil
>AY962362.1
mddtsgqtfvsvnlsdesntastrvenvkseadrleflrkmnpliidalvrktnyqgarfrarii

gvcvdcgrkhdkalkterkckvnntqsqnevahmlmhdpvkylnkrkarafsnakmfaielvlyt
kekqlavdlaaerektrlarrhpmrspeetpehykfgltakamlpelnavgvgdnedtsseypvs
lsvsggvlrehhfil
>AY962363.1
mddtsgqtlisvnlsdesntastevktvsseaerleflrkmnpfvidalirktsyqgarfrarii
gvcvdcgkkhekgsrterkckvnntqsqnevahmlmhdpveylskrkarafsnaemfaidlvmyt
kerqlavdlaaerektrlarkhpmrspeetpehykfgmtskamlpdinivdvgdnedtsseypvs
lrvsggvlrehhfil
>AY962365.1
mddtsgqtfvsvnlsdesntastkvenvkseadrleflrkmnplvidalvrktnyqgarfrarii
gvcvdcgkkhdkalkterkckvnntqsqnevahmlmhdpvkylnkrkarafsnakmfaielglyt
kekqlavdlaaerektrlarkhpmrspeetpehykfgltakailpdlnavgvgdnedtsseypvs
lsvsggvlrehhfil
>AY962368.1
mddtsgqtlisvnlsdesntastevktvsseaerleflrkmnpfvidalirktsyqgarfrarii
gvcvdcgkkhekgsrterkckvnntqsqnevahmlmhdpveylskrkarafsnaemfaidlvmyt
kerqlavdlaaerektrlarkhpmrspeetpehykfgmtakamlpdvnvvdvgdnedtsseypvs
lsisggvlrehhfil
>AY962369.1
mddtsgqtlisvnlsdesntastevktvsseaerleflrkmnpfvidalirktsyqgarfrarii
gvcvdcgkkhekgsrterkckvnntqsqnevahmlmhdpveylskrkarafsnaemfaidlvmyt
kerqlavdlaaerektrlarkhpmrspeetpehykfgmtakamlpdinivdvgdnentsseypvs
lrvsggvlrehhfil
>AY962370.1
mddtsgqtlisvnlsdesntastevktvsseaerleflrkmnpfvidalirktsyqgarfrarit
gvcvdcgkkhekgsrterkckvnntqsqnevahmlmhdpveylskrkarafsnaemfaidlvmyt
kerqlavdlaaerektrlarkhpmrspeetpehykfgmtakamlpdinivdvgdnedtsseypvs
lrvsggvlrehhfil
>AY962373.1
mddtsgqtfvsvnlsdesntastrvenvkseadrleflrkmnpliidalvrktnyqgarfrarii
gvcvdcgkkhdkalkterkckvnntqsqnevahmlmhdpvkylnkrkarafsnakmfaielvlyt
kekqlavdlaaerektrlarkhpmrspeetpehykfgltakamlpelnavgvgdnedtsseypvs
lsvsggvlrehhfil
>AY962374.1
mddtsgqtlisvnlsdesntastevktvsseaerleflrkmnpfvidalirktsyqgarfrarii
gvcvdcgkkhekgsrterkckvnntqsqnevahmlmhdpveylskrkarafsnaemfaidlvmyt
kerqlavdlaaerektrlarkhpmrspeetpehykfgmtakamlpginivdvgdnedtsseypvs
lrvsggvlrehhfil
>AY962375.1
mddtsgqtfvsvnlsdesntastrvenvkseadrleflrkmnpliidalvrktnyqgarfrarii
gvcvdcgrkhdkalkterkckvnntqsqnevahmlmhdpvkylnkrkarafsnakmfaielvlyt
kekqlavdlaaerektrlarrhpmrspeetpehykfgltakamlpelnavgvgdnedtsseypvs
lsvsggalrehhfil
>AY962376.1

mddtsgqtfvsvnlsdesntastrvenvkseadrleflrkmnpliidalvrktnyqgarfrarii
gvcvdcgkkhdkalkterkckvnntqsqnevahmlmhdpvkylnkrkarafsnakmfaielvlyt
kekqlavdlaaerektrlarrhpmrspeetpehykfgltakamlpelnavgvgdnedtsseypvs
lsvsggvlrehhfil

>AY962377.1
mddtsgqtlisvnlsdesntastevktvsseaerleflrkmnpfvvdalirktsyqgarfrarii
gvcvdcgkkhekgsrterkckvnntqsqnevahmlmhdpveylskrkarafsnaemfaidlvmyt
kerqlavdlaaerektrlarkhpmrspeetpehykfgmtakamlpdinivdvgdnedtsseypvs
lrvsggvlrehhfil

>AY962378.1
mddtsgqtlisvnlsdesntastevktvsseaerleflrkmnpfvvdalirktsyqgarfrarii
gvcvdcgkkhekgsrterkckvnntqsqnevahmlmhdpveylskrkarafsnaemfaidlvmyt
kesqlavdlaaerektrlarkhpmrspeetpehykfgmtakamlpdinivdvgdnedtsseypvs
lrvsggvlrehhfil

>AY962380.1
mddtsgqtfvsvnlsdesntastrvenvkseadrleflrkmnpliidalvrktnyqgarfrarii
gvcvdcdkkhdkalkterkckvnntqsqnevahmlmhdpvkylnkrkarafsnakmfaielvlyt
kekqlavdlaaerektrlarrhpmrspeetpehykfgmtakamlpdinivdvgdnedtsseypvs
lrvsggvlrehhfil

>AY962382.1
mddtsgqtfisvnlsdesntastrvenakseadrleflrkmnpliidalvrktnyqgarfrarii
gvcvdcgkkhdkalkterkckvnntqsqnevahmlmhdpvkylnkrkarafsnakmfaielvlyt
kekqlavdlaaerektrlarrhpmrspeetpehykfgltakailpelnavgvgdnedtsseypvs
lsvsggvlrehhfil

>AY962383.1
mddtsgqtlisvnlsdesdtastevktvsseaerleflrkmnlfvidalirktsyqgarfrarii
gvcvdcgkkhekgsrterkckvnntqsqnevahmlmhdpveylskrkarafsnaemfaidlvmyt
kerqlvvdlaaerektrlarkhpmrspeetpehykfgmtakamlpdinivdvgdnedtsseypvs
lrvsggvlrehhfil

>AY962385.1
mddtsgqtlisvnlsdesntastevktvsseaerleflrkmnpfvidalirktsyqgarfrarii
gvcvdcgkkhekgsrterkckvnntqsqnevahmlmhdpveylskrkarafsnaemfaidlvmyt
kerqlavdlaaerektrlarkhsmrspeetpehykfgmtakamlpdinivdvgdnentsseypvs
lrvsggvlrehhfil

>AY962386.1
mddtsgqtfvsvnlsdesntastrvenvkseadrleflrkmnpliidalvrktnyqgarfrarii
gvcvdcgkkhdkalkterkckvnntqsqnevahmlmhdpvkylnkrkarafsnagmfaielvlht
kekqlavdlaaerektrlarrhpmrspeetpehykfgltakailpelnavgvgdnedtsseypvs
lsvsggvlrehhfil

>AY962388.1
mddtsgqtfvsvnlsdesntastrvenvkseadrleflrkmnpliidalvrktnyqgarfrarii
gvcvdcgkkhdkalkterkykvnntqsqnevahmlmhdpvkylnkrkarafsnaemfaielvlyt
kekqlavdlaaerektrlarkhpmrspeetpehykfgltakailpelnavgvgdnentsseypvs
lsvsggvlrehhfil

>KP284579.1
mddtsgqtfvsvnlsdesntastkvenvkseadrleflrkmnpfivdalvrktnyqgarfrarii
gvcvdcgrkhdkalkterkckvnntqsqnevahmlmhdpvkylnkrkarafsnaemfaielvlyt
kerqlavdlaaerektrlarrhpirspeetpehykfgmtakamlpdinavdvgdneetsseypvs
lsvsggvlrehhfi
>KP284580.1
mddtsgqtfvsvnlsdesntaisrvenvkseadrleflrkmnpfiidalirktnyqgarfrarii
gvcvdcgrkhdkapkterkckvnntqsqnevahmlmhdpvkylnkrkarafsnaetfaielvmht
kekqlavdlaaerektrlarrhpmrspeetpehykfgitakamlpdinaidvgdnedtsseypvs
lsvssgvlrehhfi
>KP284581.1
mddtsgqtfisvnlsdesntastevktvsseadrleflrkmnpfvidalirktnyqgarfrarii
gvcvdcgrkhekgsrterkckvnntqsqnevahmlmhdpvkylskrkarafsnaemfaidlvmyt
kerqlavdlaaerektrlarkhpmrspeetpehykfgmtakamlpdvnvvdvgdnedtsseypvs
lsisggvlrehhfi
>DQ443744.1
tfvsvdfsdesntastrvenvnseadrleflrkmnpliidalvrktnyqgarfrariigvcvdcg
rkhdkalkterkckvnntqsqnevahmlmhdpvkylkkrkarafsnaemfaielvlytkekqlav
dlaaerektrlarrhpmrspeetpeyykfgmtakamlpelnavdvgdneetsseypvslsfagvv
l
>DQ443745.1
tfvsvnlsdesntastkvenvkseadrleflrkmnpfiidalvrktnyqgarfrariigvcvdcg
rkhdkalkterkckvnntqsqnevahmlmhdpvkylnkrkarafsnaemfaielvlhtkerqlav
dlaaerektrlarrhpmrspeetpehykfgmtakailpdinaidvgdneetsseypvslsvsggv
l
>DQ443746.1
tfvsvnlsdesntastavrtvsseadrleflrkmnpfiidalvrktnyqgarfrakiigvcvdcg
rkhdkglkterkckvnntqsqnevahmlmhdpvkylnkgkarafsnaemfaidlvmytkerqlav
nlaaerektrlarrhpmrspeetpeyykfgitakamlpdinavdvgdneetsseypvslsvsggv
l
>DQ443747.1
tfvsvnlsdesntastavrtvsseadrleflqkmnpfiidalvrktnyqgarfrakiigvcvdcg
rkhdkglkterkckvnntqsqnevahmlmhdpvkylnkgkarafsnaemfaidlvmytkekqlav
nlaaerektrlarrhpmrspeetpefykfgitakamlpninavdvgdnedtsseypvslsvsdgv
l
>DQ443748.1
tfvsvnlsdesntastavrtvsseadrleflrkmnpfiidalvrktnyqgarfrakiigvcvdcg
rkhdkglkterkckvnntqsqnevahmlmhdpvkylnkgkarafsnaemfaidlvmytkekqlav
nlaaerektrlarrhpmrspeetpefykfgitakamlpninavdvgdnedtssecpvslivsdgv
l
>DQ443749.1
tfvsvnlsdesntasiavrtvsseadrleflrkmnpfiidalvrktnyqgarfrakiigvcvdcg
rkhdkglkterkckvnntqsqnevahmlmhdpvkylnkgkarafsnaemfaidlvmytkekqlav
dlaaerektrlarrhpmrspeetpefykfgitakamlpninavdvgdnedtsseypvslsvsdgv

l
>DQ443750.1
tfvsvnlsdesntastavrtvsseadrleflrkmnpfiidalvrktnyqgarfrakiigvcvdcg
rkhdkglkterkckvnntqsqnevahmlmhdpvkylnkgkarafsnaemfaidlvmytkerqlav
nlaaerektrlarrhpmrspeetpefykfgitakamlpninavdvgdnedtsseypvslsvsdgv
l
>DQ443751.1
tfvsvnlsdesntastavrtvsseadrleflrkmnpfiidalvrktnyqgarfrakiigvcvdcg
rkhdkglkterkckvnntqsqnevahmlmhdpvkylnkgkarafsnaemfaidlvmytkekqlav
nlaaerektrlarrhpmrspeetpefykfgitakamlpninavdvgdnedtsseypvslsvsdgv
l
>KP268290.1
mddtsgqtfvsvnlsdesntastevktvsleadrleflrkmnpftidalirkttyqgarfrarim
gvcvdcgrkhdkasrterkckvnntqsqnevahmlmhdpvkylkerkarafsnaemfaidlvmht
kerqlavdlaaerertrlarrhpmrspeetpehykfgmtakamlpdinaidvgdn
>KP268297.1
mddtsgqtfvsvnlsdesntastevktvsleadrleflrkmnpftidalirkttyqgarfrarim
gvcvdcgrkhdkasraerkckvnntqsqnevahmlmhdpvkylkerkarafsnaemfaidlvmht
kerqlavdlaaerertrlarrhpmrspeetpehykfgmtakamlpdinaidvgdn
>KP268298.1
mdstsgqtfvsvnlsdesntastavrtvsseadrleflrkmnpfvidalvrktsyqgarfrakii
gvcvdcgrkhekgsrterkckvnntqsqnevahmlmhdpvkylskrkarafsnaemfaidlvmyt
kerqlavdlaaerektrlarkhpmrspeetpehykfgmtakamlpdinavdvgdn
>KP268300.1
mdstsgqtfisvnlsdesntastevktvsseaerleflrkmnpfvidaliretsyqgarfrarii
gvcvdcgrkhekgsrterkckvnntqsqnevahmlmhdpvkylskrkarafsntemfaidlvmyt
kerqlavdlaaerektrlarkhpmrspeetpehykfgmtakamlpdinivdvgdn
>KP268301.1
mdstsgqtfvsvnlsdesntastavrtvsseadrleflrkmnpfvidalvrktsyqgarfrakii
gvcvdcgrkhdkglkterkckvnntqsqnevahmlmhdpvkylnkrkarafsnaemfaidlvmyt
kekqlavnlaaerektrlarrhpmrspeetpehykfgmtakamlpdinavdvgdn
>KP268302.1
mdstsgqtfvsvnlsdesntastavrtvsseadrleflrkmnpfvidalvrktsyqgarfrakii
gvcvdcgrkhdkglkterkckvnntqsqnevahmlmhdpvkylnkrkarafsnaemfaidlvmyt
kerqlavdlaaerektrlarkhpmrspeetpehykfgmtakamlpdinivdvgdn
>KP268305.1
mddtsgqtfisvnlsdesntastevktvsseaerleflrkmnpfvidalirktsyqgarfrarii
gvcvdcgrkhekgsrterkckvnntqsqnevahmlmhdpvkylskrkarafsnaemfaidlvmyt
kerqlavdlaaerektrlarkhpmrspeetpehykfgmtakamlpdinivdvgdn
>KP268310.1
mdstsgqtfvsvnlsdesntastavrtvsseadrleflrkmnpfvidalvrktsyqgarfrakii
gvcvdcgrkhdkglkterkckvnntqsqnevahmlmhdpvkylnkrkarafsnaemfaidlvmyt
kekqlavnlaaerektrlarrhpmrfpeetpehykfgmtakamlpdinavdvgdn
>KP268313.1

mddtsgqtfisvnlsdesntasteikavsseadrleflrkmnpfiidalirknnyqgarfrvrii
gvcvdcgrkhdkasrterkckvnntqsqnevahmlmhdpvkylnkrkarafsnaemfaidlvmht
kerqlavdlaaerektrlarrhpmrspeetpehykfgmtakamlpdinavdvgdn
>KP268315.1
mddtsgqtfisvnlsdesntasteikavsseadrleflrkmnpfiidalirknnyqgarfrarii
gvcvdcgrkhdkasrterkckvnntqsqnevahmlmhdpvkylnkrkarafsnaemfaidlamht
kerqlavdlaaerektrlarrhpmrspeetpehykfgmtakamlpdinavdvgdn
>KP268318.1
mddtsgqtfisvnlsdesntasteikavsseadrleflrkmnpfiidalirknnyqgarfrarii
gvcvdcgrkhdkasrterkckvnntqsqnevahmlmhdpvkylnkrkarafsnaemfaidlvmht
kerqlavdlaaerektrlarrhpmrspeetpehykfgmtakamlpdinavdvgdn
>KP268320.1
mddtsgqtfisvnlsdesntasteieavsseadrleflrkmnpfiidalirknnyqgarfrarii
gvcvdcgrkhdkasrterkckvnntqsqnevahmlmhdpvkylnkrkarafsnaemfaidlvmht
kerqlavdlaaerektrlarrhpmrspeetpehykfgmtakamlpdinavdvgdn
>KP268323.1
mddtsgqtfisvnlsdesntasteikavsseadrleflrkmnpfiidalirknnyqgarfrarii
gvcvdcgrkhdkasrterkckvnntqsqnevahmlmhdpvkylnkrkarafsnaemfaidlvmht
kerqlavdlaaerektrlarrhpmrspeetpehykfgmtakamlpdinavdvgdnrwr
>KP268335.1
mddtsgqtfvsvnlsdesntastevktvsleadrleflrkmnpfiidalirkttyqgarfrarii
gvcvdcgrkhdkasrterkckvnntqsqnevahmlmhnpveylkkrkarafsnaemfaidlvmht
kerqlavdlaaerertrlarrhpmrspeetpehykfgmtakamlpdinaidvgdn
>KP268336.1
mddtsgqtfvsvnlsdesntastevktvsleadrleflrkmnpfiidalirkttyqgarfrarii
gvcvdcgrkhdkasrterkckvnntqsqnevahmlmhnpveylkkrkarafsnaemfaidlvmht
kerqlavdlaaererarlarrhpmrspeetpehykfgmtakamlpdinaidvgdn
>KP268343.1
mddtsgqtfvsvnlsdesntastevktvsleadrleflrkmnpfiidalirkttyqgarfrarii
gvcvdcgrkhdkasrterkckvnntqsqnevahmlmhnpveylkkrkarafsnaemfaidlvtht
kerqlavdlaaerertrlarrhpmrspeetpehykfgmtakamlpdinaidvgdn
>KP268346.1
mddtsgqtfvsvslsdesntastevktvsleadrleflrkmnpfiidalirkttyqgarfrarii
gvcvdcgrkhdkasrterkckvnntqsqnevahmlmhnpveylkkrkarafsnaemfaidlvmht
kerqlavdlaaerertrlarrhpmrspeetpehykfgmtakamlpdinaidvgdn
>KP268349.1
mddtsgqtfvsvnlsdesntastevktvsleadrleflrkmnpfiidalirkttyqgarfrarii
gvcvdcgrkhdkasrterkckvnntqsqnevahmlmhnpveylkkrkarafsnaemfaidlvmht
kerqlavdlaaerertrlarrhpmrspeetpehykfgmtakamlpdtnaidvgdn
>KP268355.1
mddtsgqtfvsvnlsdesntattdvkpvsseadrldflqkmnpiiidalirknsyqgarfrarii
gvcvdcgrkhdkglkterkckvnntqsqnevahmlmhdpvkylnkrkarafsnaeifaidlvmyt
kerqlaidlaaerektrlarrhpmrspeetpehykfgmtakamlpdinaidvgdn
>KP268357.1

mddtsgqtfvsvnlsdesntattdvkpvsseadrldflqkmnpiiidalirknsyqgarfrarii
gvcvdcgrkhdkglkterkckvnntqsqnevahmlmhdpvkylnkrkarafsnveifaidlvmyt
kerqlaidsaaerektrlarrhpmrspeetpghykfgrtakamlpdvnavdvgdn
>KP268359.1
mddtsgqtfvsvnlsdesntastevktvsleadrleflrkmnpfiidalirkttyqgarfrarii
gvcvdcgrkhdkasrterkckvnntqsqnevahmlmhdpveylkkrkarafsnaemfaidlvmht
kerqlavdsaaerertrlarrhpmrspeetpehykfgmtakamlpdinaidvgdn
>KP268361.1
mdnasgqtfvsvnlsdesntattdvkpvsseadrldflqkmnpiiidalirknsyqgarfrarii
gvcvdcgrkhdkglkterkckvnntqsqnevahmlmhdpvkylnkrkarafsnaeifaidlvmyt
kerqlaidlaaerektrlarrhpmrspeetpehykfgrtakamlpdvnavdvgdn
>KP268364.1
mddtsgqtfvsvnlsdesntastevktvsleadrleflrkmnpfiidalirkttyqgarfrarii
gvcvdcgrkhdkasrterkckvnntqsqnevahmlmhdpveylkkrkarafsnaemfaidlvmht
kerqlavdlaaerertrlarrhpmrspeetpehykfgmtakamlpdinaidvgdn
>KP268368.1
idstsgqtfvsvnlsdesntastavrtvsseadrleflrkmnpfvidalvrktsyqgarfrakii
gvcvdcgrkhdkglkterkckvnntqsqnevahmlmhdpvkylnkrkarafsnaemfaidlvmyt
kekqlavnlaaerektrlarrhpmrspeetpehykfgmtakamlpdinavdvgdn
>KP268371.1
mndtsgqtfisvnlsdesntastevktvsseadrleflrkmnpfiidalirktnyqgarfrarii
gvcvdcgrkhdkglkterkckvnntqsqnevahmlmhdpvkylskrkarafsnaemfaidlvmyt
kerqlavdlaaerektrlarkhpmrspeetpehykfgmtakamlpdvnvvdvgdn
>KP268372.1
mdstsgqtfvsvnlsdesntastavrtvsseadrleflrkmnpfvidalvrktsyqgarfrakii
gvcvdcgrkhdkglkterkckvnntqsqnevahmlmhdpvkylnkrkarafsnaemfaidlvmyt
kekqlavdlaaerektrlarrhpmrspeetpehykfgmtakamlpdinavdvgdn
>KP268376.1
mddtsgqtfvsvnlsdesntastevktvsleadrleflrkmnpftinalirkttyqgarfrarii
gvcvdcgrkhdkasrterkckvnntqsqnevahmlmhdpvkylkerkarafsnaemfaidlvmht
kerqlavdlaaererarlarrhpmrspeetpehykfgvtakamlpdinaidvgdn
>KP268377.1
mddtsgqtfvsvnlsdesntastevktvsleadrleflrkmnpftinaliwkttyqgarfrarii
gvcvdcgrkhdkasrterkckvnntqsqnevahmlmhdpvkylkerkarafsnaemfaidlvmht
kerqlavdlsaererarlarrhpmrspeetpehykfgvtakamlpdinaidvgdn
>KP268383.1
mddtsgqtfisvnlsdesntastevktvsseaerleflrkmnpfvidalirktsyqgarfrarii
gvcvdcgrkhekglrterkckvnntqsqnevahmlmrdpvkylskrkarafsnaemfaidlvmyt
kerqlavdlaaerektrlarkhpmrspeetpehykfgmtakamspdinivdvgdn
>KP268384.1
mddtsgqtfisvnlsdesntastevktvsseaerleflrkmnpfvidalirktsyqgarfrarii
gvcvdcgrkhekglrterkckvnntqsqnevahmlmrdpvkylskrkarafsnaemfaidlvmyt
kerqlavdlaaerektrlarkhpmrspeetpehykfgmtakamspdinivdvgdd
>KP268385.1

mddtsgqtfisvnlsdesntastevktvsseaerleflrkmnpfvidalirktsyqgarfrarii
gvcvdcgrkhekglrterkckvnntqsqnevahmlmrdpvkylskrkarafsnaemfaidlvmyt
kerqlavdlaaerektrlarkhpmrspeetpehykfgmtakamlpdinivdvgdn

>KP268386.1

mddtsgqtfisvnlsdesntastevktvsseaerleflrkmnpfvidalirktsyqgtrfrarii
gvcvdcgrkhekglrterkckvnntqsqnevahmlmrdpvkylskrkarafsnaemfaidlvmyt
kerqlavdlaaerektrlarkhpmrspeetpehykfgmtakamlpdinivdvgdn

>KP268391.1

mddtsgqtlisvnlsdesntastevktvsseaerleflrkmnpfvidalirktsyqgarfrarii
gvcvdcgkkhekgsrterkckvnntqsqnevahmlmhdpveylskrkarafsnaemfaidlvmyt
kerqlavdlaaerektrlarkhpmrspeetpehykfgmtakamlpdinivdvgdnedtsseypvs
lrvsggvlrehhfil

>KP268393.1

mndtsgqtfisvnlsdesntastevktvsseadrleflrkmnpfiidalirktsyqgarfrarii
gvcvdcgrkhdkgsrterkckvnntqsqnevahmlmhdpvkylskrkarafsnaemfaidlvmyt
kerqlavdlaaerektrlarkhpmrspeetpehykfgmtakamlpdvnvvdvgdn

>KP268396.1

Mddtsgqtfisvnlsdesntastevktvsseadrleflrkmnpfvidalirktsyqgarfrarii
gvcvdcgrkhekgsrterkckvnntqsqnevahmlmhdpvkylskrkarafsnaemlaidlvmyt
kerqlavdlaaerektrlarkhpmrspeetpehykfgmtakamlpdvnvvdvgdn

Supplemental Data 4-2 : classification of p23 amino acid sequences

>Cluster 0

0 174aa, >DQ831487.1... at 93.68%
1 209aa, >U42017.1... at 93.78%
2 209aa, >U42018.1... at 96.65%
3 209aa, >U42022.1... at 93.30%
4 209aa, >U42023.1... at 93.78%
5 185aa, >EU262685.1... at 94.05%
6 204aa, >EU483657.1... at 94.61%
7 204aa, >EU487601.1... at 93.63%
8 209aa, >FJ860145.1... at 93.30%
9 209aa, >GQ475044.1... at 95.22%
10 209aa, >GQ475045.1... at 93.78%
11 209aa, >GQ475046.1... at 93.78%
12 209aa, >GQ475047.1... at 93.78%
13 209aa, >GQ475048.1... at 94.26%
14 196aa, >GQ338647.1... at 94.90%
15 196aa, >GQ338650.1... at 93.37%
16 196aa, >GQ338652.1... at 97.45%
17 196aa, >GQ338653.1... at 94.39%
18 196aa, >GQ338654.1... at 93.37%
19 196aa, >GQ338658.1... at 96.94%
20 210aa, >HQ329231.1... at 97.14%
21 210aa, >HQ329232.1... at 98.10%
22 210aa, >HQ329233.1... at 98.10%
23 210aa, >HQ329234.1... at 94.29%
24 210aa, >HQ329235.1... at 93.33%
25 210aa, >HQ329236.1... at 94.29%
26 210aa, >HQ329237.1... at 97.62%
27 210aa, >HQ329239.1... at 95.71%
28 210aa, >HQ329240.1... at 93.81%
29 210aa, >HQ329241.1... at 93.33%
30 210aa, >HQ329242.1... at 94.29%
31 210aa, >HQ329243.1... at 97.14%
32 210aa, >HQ329244.1... at 97.62%
33 210aa, >HQ329245.1... at 94.29%
34 210aa, >HQ329246.1... at 93.33%
35 209aa, >AJ579773.1... at 93.30%
36 209aa, >AJ579776.1... at 95.22%
37 209aa, >AJ579777.1... at 94.74%
38 210aa, >JN384022.1... at 93.81%
39 205aa, >JQ819955.1... at 93.17%
40 205aa, >JQ819960.1... at 93.17%
41 205aa, >JQ820001.1... at 93.17%

42 204aa, >JQ820013.1... at 93.63%

43 213aa, >KC202908.1... *

44 209aa, >KC562142.1... at 93.30%

45 209aa, >KC577594.1... at 93.30%

46 209aa, >KF724146.1... at 97.61%

47 209aa, >KC774010.1... at 93.78%

48 209aa, >KC774012.1... at 93.78%

49 209aa, >KC774014.1... at 94.26%

50 209aa, >KC774015.1... at 94.26%

51 209aa, >KF913716.1... at 93.78%

52 210aa, >AY750732.1... at 93.33%

53 210aa, >AY750735.1... at 93.81%

54 210aa, >AY750740.1... at 96.67%

55 210aa, >AY750742.1... at 93.33%

56 210aa, >AY750745.1... at 97.14%

57 210aa, >AY750746.1... at 94.29%

58 210aa, >AY962348.1... at 95.24%

59 210aa, >AY962350.1... at 95.71%

60 210aa, >AY962356.1... at 95.71%

61 210aa, >AY962363.1... at 94.29%

62 210aa, >AY962368.1... at 95.71%

63 210aa, >AY962369.1... at 94.29%

64 210aa, >AY962370.1... at 94.29%

65 210aa, >AY962374.1... at 94.29%

66 210aa, >AY962377.1... at 94.29%

67 210aa, >AY962378.1... at 93.81%

68 210aa, >AY962383.1... at 93.33%

69 210aa, >AY962385.1... at 93.81%

70 209aa, >KP284581.1... at 97.61%

71 185aa, >KP268298.1... at 95.14%

72 185aa, >KP268300.1... at 96.22%

73 185aa, >KP268302.1... at 94.05%

74 185aa, >KP268305.1... at 97.30%

75 185aa, >KP268318.1... at 93.51%

76 185aa, >KP268371.1... at 95.68%

77 185aa, >KP268383.1... at 95.68%

78 185aa, >KP268384.1... at 95.14%

79 185aa, >KP268385.1... at 96.22%

80 185aa, >KP268386.1... at 95.68%

81 210aa, >KP268391.1... at 94.76%

82 185aa, >KP268393.1... at 97.30%

83 185aa, >KP268396.1... at 97.30%

>Cluster 1

0 209aa, >U42006.1... at 94.74%

1	185aa, >EU262688.1... at 93.51%
2	210aa, >FJ860136.1... *
3	209aa, >FJ860138.1... at 99.52%
4	209aa, >FJ860140.1... at 99.04%
5	209aa, >AJ579774.1... at 94.26%
6	205aa, >JQ819944.1... at 97.56%
7	205aa, >JQ819963.1... at 98.54%
8	205aa, >JQ819964.1... at 98.05%
9	205aa, >JQ819965.1... at 98.54%
10	205aa, >JQ819967.1... at 98.05%
11	205aa, >JQ819968.1... at 98.05%
12	205aa, >JQ819969.1... at 99.02%
13	205aa, >JQ819971.1... at 99.02%
14	205aa, >JQ819973.1... at 98.54%
15	205aa, >JQ819974.1... at 98.54%
16	205aa, >JQ819976.1... at 99.02%
17	205aa, >JQ819979.1... at 98.54%
18	205aa, >JQ819981.1... at 98.54%
19	205aa, >JQ819983.1... at 98.05%
20	205aa, >JQ819987.1... at 98.54%
21	205aa, >JQ819988.1... at 99.02%
22	205aa, >JQ819991.1... at 98.05%
23	205aa, >JQ819996.1... at 98.54%
24	205aa, >JQ819997.1... at 99.02%
25	205aa, >JQ820000.1... at 98.54%
26	205aa, >JQ820003.1... at 98.54%
27	205aa, >JQ820007.1... at 99.02%
28	205aa, >JQ820008.1... at 99.02%
29	205aa, >JQ820010.1... at 96.59%
30	209aa, >KC562143.1... at 95.22%
31	209aa, >KC562144.1... at 95.69%
32	209aa, >KF913717.1... at 94.74%
33	209aa, >KF913718.1... at 95.69%
34	210aa, >AY750733.1... at 94.29%
35	210aa, >AY750738.1... at 94.76%
36	210aa, >AY750741.1... at 95.24%
37	210aa, >AY750744.1... at 93.81%
38	210aa, >AY750750.1... at 94.76%
39	185aa, >KP268290.1... at 94.59%
40	185aa, >KP268297.1... at 94.05%
41	185aa, >KP268313.1... at 95.14%
42	185aa, >KP268315.1... at 95.14%
43	185aa, >KP268320.1... at 95.14%
44	188aa, >KP268323.1... at 94.15%

45 185aa, >KP268335.1... at 95.14%
46 185aa, >KP268336.1... at 94.59%
47 185aa, >KP268343.1... at 94.59%
48 185aa, >KP268346.1... at 94.59%
49 185aa, >KP268349.1... at 94.59%
50 185aa, >KP268359.1... at 95.14%
51 185aa, >KP268364.1... at 95.68%
52 185aa, >KP268376.1... at 94.05%
>Cluster 2
0 210aa, >HQ329238.1... *
1 210aa, >AY750739.1... at 93.81%
2 196aa, >DQ443746.1... at 93.37%
>Cluster 3
0 196aa, >AJ297704.1... at 93.88%
1 174aa, >DQ831493.1... at 94.25%
2 174aa, >DQ831496.1... at 95.40%
3 209aa, >AJ298843.1... at 94.26%
4 209aa, >U42019.1... at 98.09%
5 209aa, >U42020.1... at 96.65%
6 209aa, >U42021.1... at 98.56%
7 209aa, >U42024.1... at 98.09%
8 204aa, >EU487603.1... at 99.51%
9 209aa, >FJ860143.1... at 97.13%
10 209aa, >FJ860144.1... at 97.13%
11 209aa, >FJ860146.1... at 98.09%
12 196aa, >GQ338645.1... at 95.92%
13 196aa, >GQ338646.1... at 94.39%
14 196aa, >GQ338648.1... at 96.94%
15 196aa, >GQ338651.1... at 94.90%
16 196aa, >GQ338656.1... at 93.37%
17 196aa, >GQ338659.1... at 95.41%
18 209aa, >AJ579762.1... at 98.56%
19 209aa, >AJ579764.1... at 99.52%
20 209aa, >AJ579766.1... at 99.04%
21 209aa, >AJ579768.1... at 94.26%
22 210aa, >JN384024.1... *
23 209aa, >JQ039913.1... at 96.17%
24 205aa, >JQ819945.1... at 93.66%
25 205aa, >JQ819946.1... at 93.17%
26 205aa, >JQ819954.1... at 94.15%
27 209aa, >AY652902.1... at 94.26%
28 209aa, >AY652906.1... at 98.56%
29 209aa, >KF724132.1... at 99.52%
30 209aa, >KF724135.1... at 99.52%

31 209aa, >KF724136.1... at 99.04%
32 209aa, >KF724137.1... at 99.04%
33 209aa, >KF724141.1... at 99.04%
34 209aa, >KC774011.1... at 98.56%
35 209aa, >KC774013.1... at 99.04%
36 209aa, >KC774017.1... at 100.00%
37 210aa, >AY750736.1... at 93.81%
38 210aa, >AY750747.1... at 93.33%
39 210aa, >AY962349.1... at 94.76%
40 210aa, >AY962357.1... at 93.33%
41 210aa, >AY962358.1... at 94.29%
42 210aa, >AY962362.1... at 94.29%
43 210aa, >AY962373.1... at 93.33%
44 210aa, >AY962375.1... at 93.81%
45 210aa, >AY962376.1... at 93.81%
46 210aa, >AY962380.1... at 94.29%
47 209aa, >KP284579.1... at 99.52%
48 196aa, >DQ443745.1... at 96.94%
>Cluster 4
0 174aa, >DQ831488.1... at 98.85%
1 185aa, >EU262682.1... at 99.46%
2 185aa, >EU262683.1... at 98.92%
3 185aa, >EU262686.1... at 96.76%
4 185aa, >EU262689.1... at 98.92%
5 209aa, >FJ860142.1... at 97.61%
6 209aa, >GQ475049.1... at 98.56%
7 209aa, >GQ475051.1... at 98.09%
8 209aa, >GQ475052.1... at 99.04%
9 196aa, >GQ338657.1... at 98.98%
10 209aa, >AJ579770.1... at 96.65%
11 209aa, >AJ579771.1... at 99.52%
12 210aa, >KC202886.1... *
13 209aa, >AY652901.1... at 95.69%
14 210aa, >AY750737.1... at 98.57%
15 210aa, >AY750743.1... at 96.19%
16 210aa, >AY750748.1... at 96.67%
17 210aa, >AY750749.1... at 99.05%
18 210aa, >AY750751.1... at 99.52%
19 210aa, >AY962351.1... at 98.10%
20 210aa, >AY962359.1... at 99.05%
21 210aa, >AY962361.1... at 98.57%
22 196aa, >DQ443747.1... at 98.98%
23 196aa, >DQ443748.1... at 98.47%
24 196aa, >DQ443749.1... at 98.47%

25 196aa, >DQ443750.1... at 98.98%
26 196aa, >DQ443751.1... at 99.49%
27 185aa, >KP268301.1... at 96.22%
28 185aa, >KP268310.1... at 95.68%
29 185aa, >KP268368.1... at 95.68%
30 185aa, >KP268372.1... at 95.68%
>Cluster 5
0 210aa, >AY962355.1... *
>Cluster 6
0 205aa, >JQ819943.1... at 95.12%
1 205aa, >JQ819947.1... at 95.12%
2 205aa, >JQ819948.1... at 94.15%
3 210aa, >AY962360.1... *
4 210aa, >AY962365.1... at 96.19%
5 210aa, >AY962382.1... at 97.62%
6 210aa, >AY962386.1... at 97.62%
7 210aa, >AY962388.1... at 96.67%
>Cluster 7
0 209aa, >AJ579767.1... *
>Cluster 8
0 209aa, >AJ579769.1... *
>Cluster 9
0 209aa, >AJ579772.1... *
1 209aa, >KC774016.1... at 97.61%
2 185aa, >KP268355.1... at 97.84%
3 185aa, >KP268357.1... at 96.76%
4 185aa, >KP268361.1... at 98.38%
>Cluster 10
0 209aa, >KC562141.1... *
1 209aa, >KC562145.1... at 99.04%
2 209aa, >KC562146.1... at 97.13%
3 209aa, >KF913715.1... at 96.65%
4 209aa, >KJ094313.1... at 96.65%
>Cluster 11
0 209aa, >AY652904.1... *
>Cluster 12
0 209aa, >KP284580.1... *
>Cluster 13
0 206aa, >KC202889.1... *
>Cluster 14
0 205aa, >JQ819952.1... *
>Cluster 15
0 200aa, >KC202888.1... *
>Cluster 16

0	196aa, >DQ443744.1... *
>Cluster 17
0	185aa, >KP268377.1... *
>Cluster 18
0	174aa, >DQ831495.1... *
>Cluster 19
0	170aa, >KC202887.1... *

Supplemental Data 4-3 : The phylogenetic tree of p23 sequences

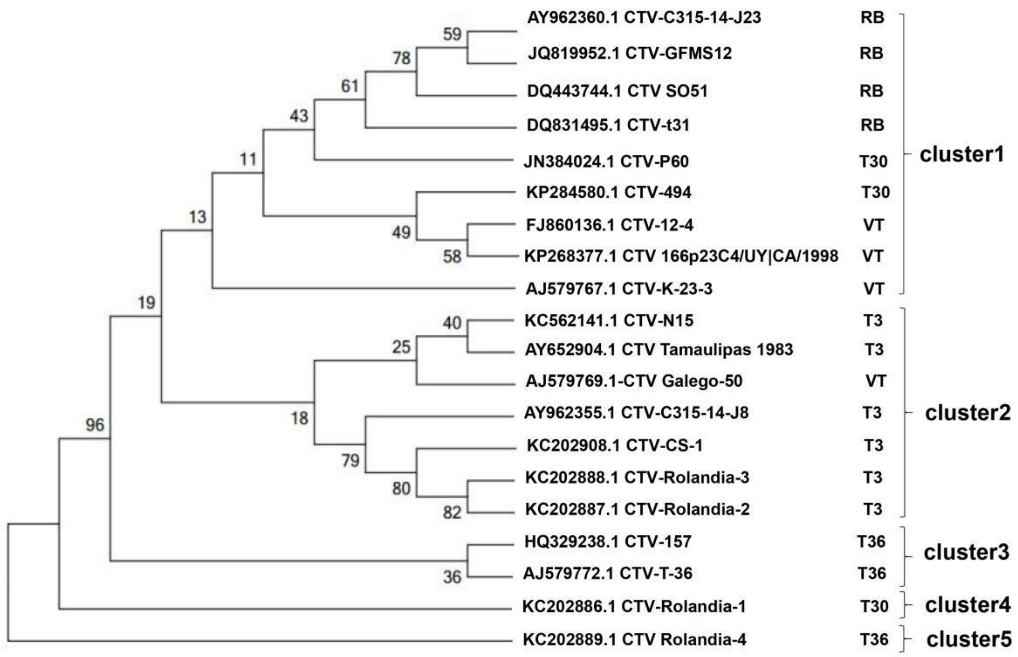

Supplemental Data 4-4 : p23 amino acid sequence alignment

```
                                                                          Section 1
              (1) 1        10        20        30        40            54
AJ579767.1    (1) MNDTSGQT-------FVSVNLSDESNTASTEVKTVSSEADRLEFLRKMNPFTID
AY962360.1    (1) MDDTSGQT-------FVSVNLSDESNTASTRVENVKSEADRLEFLRKMNPLIID
JQ819952.1    (1) MNDTSGQT-------FVSVNLSDESNTAISRVKNVKSEADRLEFLRKMNPLIID
DQ443744.1    (1) -------T-------FVSVDFSDESNTASTRVENVNSEADRLEFLRKMNPLIID
DQ831495.1    (1) --------------------------TASTKVETVKSEADRLEFLRKMNPFIID
JN384024.1    (1) MDDTSGQT-------FVSVNLSDESNTASTKVENVKSEADRLEFLRKMNPFIVD
KP284580.1    (1) MDDTSGQT-------FVSVNLSDESNTAISRVENVKSEADRLEFLRKMNPFIID
AJ579772.1    (1) MDNTSGQT-------FVSVNLSDESNTATTDVKPVSSEADRLDFLQKMNPIIID
HQ329238.1    (1) MDNTSGQT-------FISVNLSDESNTASTEVKTVSSEADRLEFLRKMNPFIID
AY652904.1    (1) MDSTNGQT-------FVSVNLSDESNTASTAVRTVSSEADRLEFLRKMNPFIID
KC202886.1    (1) MDSTSGQT-------FVSVNLSPESNTASTAVRTVSSEADRLEFLRKMNPFIID
KC202889.1    (1) MDDTSGQKGTPFRFCEPFVTASTEVKTVSSEYRTVSSEADTLGILTKNEPLYHR
AJ579769.1    (1) MDDTSGQT-------FISVDLSDESNTASTEVKTVSLEADRLEFLRKMNPFTID
AY962355.1    (1) MDDTSGQT-------LISVNLSDESNTASTEVKTVSSEAERLEFLRKMNPFVID
KC202888.1    (1) MDDTSGQT-------FISVNLSDESNTASTEVKTVSSEAERLEFLRKMNPFVID
KC202887.1    (1) -----------------------------------------------MNPFVID
KC202908.1    (1) MDNTSGQT-------FISVNLSDESNTASTEVKTVSSEADRLEFLRKMNPFVID
FJ860136.1    (1) MDDTSGQT-------FISVNLSDESNTASTEVKTVSLEADRLEFLRKMNPFIID
KP268377.1    (1) MDDTSGQT-------FVSVNLSDESNTASTEVKTVSLEADRLEFLRKMNPFTIN
KC562141.1    (1) MDYTSGQT-------FVSVNLSDESNTASTEVKTVSSEADRLEFLQKMNPFVID

                                                                          Section 2
             (55) 55   60        70        80        90                108
AJ579767.1   (48) ALIRKASYQGARFRARIIG--VCVDCGRKHDKGLKTERKCKVNNTQSQNEVAHM
AY962360.1   (48) ALVRKTNYQGARFRAKIIG--VCVDCGKKHDKALKTERKCKVNNTQSQNEVAHM
JQ819952.1   (48) ALVGKTNYQGARFRARIIG--VCVDCGRKHDKAVRTERKCKVNNTQSQNEVAHM
DQ443744.1   (41) ALVRKTNYQGARFRARIIG--VCVDCGRKHDKALKTERKCKVNNTQSQNEVAHM
DQ831495.1   (29) ALVRKTNYQGARFRARIIG--VCVDCGRKHDKGFKTERKCKVNNTQSQNEVAHM
JN384024.1   (48) ALVRKTNYQGARFRARIIG--VCVDCGRKHDKALKTERKCKVNNTQSQNEVAHM
KP284580.1   (48) ALIRKTNYQGARFRARIIG--VCVDCGRKHDKAPKTERKCKVNNTQSQNEVAHM
AJ579772.1   (48) ALIRKNSYQGARFRARIIG--VCVDCGRKHDKGLKTERKCKVNNTQSQNEVAHM
HQ329238.1   (48) ALIRKNSYQGARFRARIIG--VCVDCGRKHDKGLKTERKCKVNNTQSQNEVAHM
AY652904.1   (48) ALIRKTSYQGARFRARIIG--VCVDCGRKHDKGLRTERKCKVNNTRSQNEVAHM
KC202886.1   (48) ALVRKTNYQGARFRAKIIG--VCVDCGRKHDKGLKTERKCKVNNTQSQNEVAHM
KC202889.1   (55) RFGTENTVSRVKNSLSRKNNRSMRRLGRKHDKGLKTERKCKVNNTQSQNEVAHM
AJ579769.1   (48) ALIRKTNYQGARFRARIIG--VCVDCGRKHNKGLRTERKCKVNNTQSQNEVAHM
AY962355.1   (48) ALIRKTSYQGARFRARIIG--VCVGCGRKHEKGSRTERKCKVNNTQSQNEVAHM
KC202888.1   (48) ALIRKTSYQGARFRARIIG--VCVDCGRKHEKGSRTERKCKVNNTQSQNEVAHM
KC202887.1    (8) ALIRKTSYQGARFRARIIG--VCVDCGRKHEKGSRTERKCKVNNTQSQNEVAHM
KC202908.1   (48) ALIRKTSYQGARFRARIIG--VCVDCSRKHDKGSRTERKCKVNNTQSQNEVAHM
FJ860136.1   (48) ALIRKTNYQGARFRARIIG--VCVDCGRKHDKASRTERKCKVNNTQSQAEVAHM
KP268377.1   (48) ALIWKTTYQGARFRARIIG--VCVDCGRKHDKASRTERKCKVNNTQSQNEVAHM
KC562141.1   (48) ALIRKTSYQGARFRARIIG--VCVDCGRKHNKGLKTERKCKVNNTQSQNEVAHM
```

Section 3

```
              (109) 109      120       130       140       150         162
AJ579767.1 (100) LMHDPVKYLNKRKARAFSNAEMFAIELVLHTKERQLAVDLAAEREKTRLARRHP
AY962360.1 (100) LMHDPVKYLNKRKARAFSNAKMFAIELVLYTKEKQLAVDLAAEREKTRLARRHP
JQ819952.1 (100) LMHDPVNYLNKRKARAFSNAEMFAIELFLYTKEKQLAVDLAAEREKTRLARRHP
DQ443744.1  (93) LMHDPVKYLKKRKARAFSNAEMFAIELVLYTKEKQLAVDLAAEREKTRLARRHP
DQ831495.1  (81) LMHDPVKYLNKRKARAFSNAETFAIDLVLYTKEKQLAVDLAAEREKTRLARRHP
JN384024.1 (100) LMHDPVKYLNKRKARAFSNAEMFAIELVSYTKERQLAVDLAAEREKTRLARRHP
KP284580.1 (100) LMHDPVKYLNKRKARAFSNAETFAIELVMHTKEKQLAVDLAAEREKTRLARRHP
AJ579772.1 (100) LMHDPVKYLNKRKARAFSNAEIFAIDLVMYTKERQLAIDLAAEREKTRLARRHP
HQ329238.1 (100) LMHDPVKYLNKGKARPFSNAEMFAIDLVMHTKERQLAVNLAAEREKTRLARRHP
AY652904.1 (100) LMHDPVKYLNEGKARAFSNAEMFAIDLVMYTKEKQLADDLAAEREKTRLARRHP
KC202886.1 (100) LMHDPVKYLNKGKARAFSNAEMFAIDLVMYTKEKQLAVNLAAEREKTRLARRHP
KC202889.1 (109) LMHDPVKYLNKGKARAFSNAEMFAIDLVMYTKEKQLAVNLAAEREKTRLARRHP
AJ579769.1 (100) LMHNPVEYLNKGKARAFSNAEMFAIDLVMYTKEKQSAVNLAAEREKTRLARRHP
AY962355.1 (100) LMHDPVEYLSKRKARAFSNAEMFAIDLVMYTKERQLAVDLAAEREKTRLARRHP
KC202888.1 (100) LMHDPVKYLSKRKARAFSNAEI-AIDLVMYTKERQLAVDLAAEREKTRLALNTQ
KC202887.1  (60) LMHDPVKYLSKRKARAFSNAEMFAIDLVMYTKERQLAVDLAAEREKTRLARKHP
KC202908.1 (100) LMHDPVKYLSKRKARAFSNAEMFAIDLVMYTKERQLAVDLAAEREKTRLARKHP
FJ860136.1 (100) LMHDPVKYLNKRKARAFSNAEMFAIDLVMHTKERQLAVDLAAEREKTRLARRHP
KP268377.1 (100) LMHDPVKYLKERKARAFSNAEMFAIDLVMHTKERQLAVDLSAERERARLARRHP
KC562141.1 (100) LMHDPVKYLNEGKARAFSNAEMFAIDLVMYTKERQLAVNLTAEREKTRLARRHP
```

Section 4

```
              (163) 163    170       180       190       200            216
AJ579767.1 (154) MRSPEETPEHYKFGTTAKAMLPDVNAVDVGDNEETSSEYPVSLSVSGGVLREHH
AY962360.1 (154) MRSPEETPEHYKFGLTAKAMLPELNAVGVGDNKDTSSEYPVSLSVSGGVLREHH
JQ819952.1 (154) MRSPEETPEHYKFGMTAKAILPELNAVGVGDNEDTSSDFPVFLSVSGGVLRE--
DQ443744.1 (147) MRSPEETPEYYKFGMTAKAMLPELNAVDVGDNEETSSEYPVSLSFAGVVL----
DQ831495.1 (135) MRSPEETPEHYKFGMTAKAMLPEFNAVGVGDNEDTSSEYP--------------
JN384024.1 (154) IRSPEETPEHYKFGMTAKAMLPDINAVDVGDNEETSSEYPVSLSVSGGVLREHH
KP284580.1 (154) MRSPEETPEHYKFGITAKAMLPDINAIDVGDNEDTSSEYPVSLSVSSGVLREHH
AJ579772.1 (154) MRSPEETPEYYKFGRTAKAMLPDINAVDVGDNEETSSEYPVSLSVSGGVLREHH
HQ329238.1 (154) MRSPEETPEYYKFGMTAKAMLPDINAINVGDNEDTSSEYPVSLSVSGGVLREHH
AY652904.1 (154) MRSPEETPEHYKFGMTAKAMIPDIDVVYVGDNEDTSSEYPVSLGVSDGVLREHH
KC202886.1 (154) MRSPEETPEFYKFGITAKAMLPNINAVDVGDNEDTSSEYPVSLSVSDGVLREHH
KC202889.1 (163) MRSPEETPEFYKFGITAKAMLPNINAVDVGDNEDTSSETEESEV----------
AJ579769.1 (154) MRSPEETPEHYKFGMTAKATLPDINAVDVGDNEDTSSEYPVSLSVSNGVLREHH
AY962355.1 (154) MRSPEETPEHYKFGLTAKAMLPELNAVGVGDNEDTSSEYPVSLSVSGGVLREHH
KC202888.1 (153) CVLR-KKLRNIKFGMTAKAMLPDINIVDVGDNEDTSSEYRKSESFWRVS-----
KC202887.1 (114) MRSPEETPEHYKFGMTAKAMLPDINIVDVGETTKTLFGDGVSLSVSGGVLREHH
KC202908.1 (154) MRSPEETPEHYKFGMTAKAMLPDINVVDVGDNEDTSSEYPVSLSISGGVLREHH
FJ860136.1 (154) MRSPEETPEHYKFGITAKAMLPDIDAIDVGDNEDTSSEYPVSLSVSGGVLREHH
KP268377.1 (154) MRSPEETPEHYKFGVTAKAMLPDINAIDVGDN----------------------
KC562141.1 (154) MRSPEETPEHYKFGMTAKVMLPDIDAVDVDDNEDTLSECPVNLSVSGGVLREHH
```

	(217)	217 222
AJ579767.1	(208)	FI----
AY962360.1	(208)	FIL---
JQ819952.1	(206)	------
DQ443744.1	(197)	------
DQ831495.1	(175)	------
JN384024.1	(208)	FIL---
KP284580.1	(208)	FI----
AJ579772.1	(208)	FI----
HQ329238.1	(208)	FIL---
AY652904.1	(208)	FV----
KC202886.1	(208)	FIL---
KC202889.1	(207)	------
AJ579769.1	(208)	FI----
AY962355.1	(208)	FIL---
KC202888.1	(201)	------
KC202887.1	(168)	FIL---
KC202908.1	(208)	FIRSNH
FJ860136.1	(208)	FIL---
KP268377.1	(186)	------
KC562141.1	(208)	FI----
