# Supplementary material for: Identification of Key Residues Required for RNA Silencing Suppressor Activity of p23 Protein from a Mild Strain of Citrus Tristeza Virus
Source: Viruses. 2019 Aug 25;11(9):782. doi: 10.3390/v11090782 (PMC6784005; doi:10.3390/v11090782)
Supplement: Supplementary file 1 [file viruses-11-00782-s001.zip › Supplementary Figure.pdf]

Supplemental Figure

Figure S1：Subcellular localization of EV and C2b

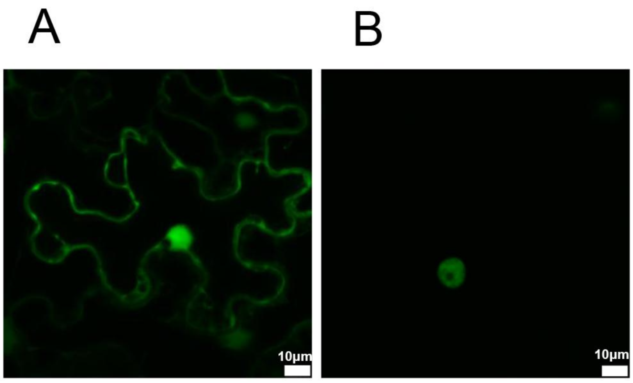

Figure S1. Subcellular localization of EV and C2b. (A) GFP-EV subcellular localization information. (B) GFP-C2b subcellular localization information.
