# Supplementary material for: Identification of Key Residues Required for RNA Silencing Suppressor Activity of p23 Protein from a Mild Strain of Citrus Tristeza Virus
Source: Viruses. 2019 Aug 25;11(9):782. doi: 10.3390/v11090782 (PMC6784005; doi:10.3390/v11090782)
Supplement: Supplementary file 1 [file viruses-11-00782-s001.zip › Supplementary Tables.pdf]

Supplemental Table

Table S1：CTV-N4 cloning primers

Primer ID	Start on N4	End on N4	Sequence
CTV-fragment1F	21	42	CCGTACCTCCGGAGCTCGATCCGGA
CTV-fragment1R	2965	2991	AATCTGAAGGGTCTAGGCGAATCGGTA
CTV-fragment2F	2935	2958	TTCAGCACCGCGTCTTTACCATTG
CTV-fragment2R	4361	4384	CTGAATGCCCACAAATAAATCGCG
CTV-fragment3F	4258	4279	GAAAGGTAGGCAGAACGCAGAA
CTV-fragment3R	6012	6034	CCTTCGATATACTAAGACACGCC
CTV-fragment4F	5935	5959	GGACGAGTCGCTGCTTAGTGACACA
CTV-fragment4R	8931	8954	TGCACCTTACCAAACGGACCTTCT
CTV-fragment5F	8881	8905	AGCGGTGCCTGTTGTGAACACAGTA
CTV-fragment5R	11938	11962	CTAACAGGCTTAGTCGTGCACCGAT
CTV-fragment6F	11901	11926	GCCTTTGCCGGACTGATTATCATTGT
CTV-fragment6R	14971	14994	AGTTGCGATACAGTCGCAAAGCCA
CTV-fragment7F	14939	14964	TAGACGTCAGTTCATGGGACGTCACA
CTV-fragment7R	17504	17523	TCATTCGCCGTGCCACCACT
CTV-fragment8F	17476	17500	CTTTTTGCGCATCGTTATCATTCCT
CTV-fragment8R	19163	19189	GGTTCACGCATACGTTAAGCCTCACTT

Table S2：CTV RNA silencing suppressor vector construction primers

Primer ID	Sequence
PH7LIC3.1.1-CP-N4-F	TGACAAGGATTACGCCGAGGTCATGGACGACGAAACAAAGAAATTG
PH7 LIC3.1.1-CP-N4-R	GGATATCATTAGGGAAGAGGTTAACGTGTGTTAAATTTCCCA
PH7 LIC3.1.1-p20-N4-F	TGACAAGGATTACGCCGAGGTCATGCGAGCTTACTTTAGTGTTAAC
PH7 LIC3.1.1-p20-N4-R	GGATATCATTAGGGAAGAGGTCACACGCAAGACGGAGAAACTAAA
PH7 LIC3.1.1-p23-N4-F	TGACAAGGATTACGCCGAGGTCATGGATGATACTAGCGGACAAACT
PH7 LIC3.1.1-p23-N4-R	GGATATCATTAGGGAAGAGGTCAGATGAAGTGGTGTTCACGGAGA

Table S3： p23 mutant vector construction primers

Primer ID	Sequence
PH7LIC3.1.1-p23 M1-R	CGCCGCTCTACCACAATCCACGC
PH7LIC3.1.1-p23 M1-F	GATTGTGGTAGAGCGGCGGACAAGGCGCTCAAGA
PH7LIC3.1.1-p23 M2-R	CGCAGTCTTGAGCGCCTTG
PH7LIC3.1.1-p23 M2-F	AAGGCGCTCAAGACTGCGCGTAAGTGTAAGGTCAAC
PH7LIC3.1.1-p23 M3-R	CGCCGCGTTGACCTTACACTTACG
PH7LIC3.1.1-p23 M3-F	TGTAAGGTCAACGCGGCGCAATCTCAGAACGAGG
PH7LIC3.1.1-p23 M4-R	CGCAGATTGCGTGTTGTTGACC
PH7LIC3.1.1-p23 M4-F	AACAACACGCAATCTGCGAACGAGGTGGCGCAT
PH7LIC3.1.1-p23 M5-R	CGCCGCGTTCTGAGATTGCGTG
PH7LIC3.1.1-p23 M5-F	CAATCTCAGAACGCGGCGGCGCATATGTTGATGCA
PH7LIC3.1.1-p23 M6-R	CGCCGCATGCGCCACCTCGTT
PH7LIC3.1.1-p23 M6-F	GAGGTGGCGCATGCGGCGATGCACGATCCCGTTA
PH7LIC3.1.1-p23 M7-R	CGCATCGTGCATCAACAT
PH7LIC3.1.1-p23 M7-F	ATGTTGATGCACGATGCGGTTAAGTATTTGAACA
PH7LIC3.1.1-p23 M8-R	CGCCGCCTTAACGGGATCGTGC
PH7LIC3.1.1-p23 M8-F	GATCCCGTTAAGGCGGCGAACAAAAGAAAGGC
PH7LIC3.1.1-p23 M9-R	CGCAGCCTTTCTTTTGTTC
PH7LIC3.1.1-p23 M9-F	AACAAAAGAAAGGCTGCGGCCTTTTCTAACGCAG
PH7LIC3.1.1-p23 M10-R	CGCGGCTCTAGCCTTTCTT
PH7LIC3.1.1-p23 M10-F	AGAAAGGCTAGAGCCGCGTCTAACGCAGAGATGT
PH7LIC3.1.1-p23 M11-R	CGCCGCAAACATCTCTGCGT
PH7LIC3.1.1-p23 M11-F	GCAGAGATGTTTGCGGCGGAACTGGTTTCGTA
PH7LIC3.1.1-p23 M12-R	CGCTTCAATCGCAAACATC
PH7LIC3.1.1-p23 M12-F	ATGTTTGCGATTGAAGCGGTTTCGTACACCAAG
PH7LIC3.1.1-p23 M13-R	TGCTGCTGCGTACGAAACCAGTTCA
PH7LIC3.1.1-p23 M13-F	TTTCGTACGCAGCAGCAAGGCAATTGGCGGTCGATTT
PH7LIC3.1.1-p23 M14-R	CGCCGCTTCAGCGGCTAAATC
PH7LIC3.1.1-p23 M14-F	TAGCCGCTGAAGCGGCGAAGACGAGACTGGCT
PH7LIC3.1.1-p23 M15-R	CGCTCTCGTCTTCTCCCTTTC
PH7LIC3.1.1-p23 M15-F	GAGAAGACGAGAGCGGCTCGTAGACACCCAATA
PH7LIC3.1.1-p23 M16-R	CTTAGCAGTCATCGCCGCTTTATAATGTTCCGG
PH7LIC3.1.1-p23 M16-F	GCGGCGATGACTGCTAAGGCA
PH7LIC3.1.1-p23 M17-R	CGCTAACATTGCCTTAGCA
PH7LIC3.1.1-p23 M17-F	GCTAAGGCAATGTTAGCGGACATCAACGCCGTAGA
PH7LIC3.1.1-p23 M18-R	CGCGTCTACGGCGTTGATG
PH7LIC3.1.1-p23 M18-F	AACGCCGTAGACGCGGGTGATAACGAGGAAA
PH7LIC3.1.1-p23- M-F	GACAAGGATTACGCCGAGGTCATGGATGATACTAGCGGAC
PH7LIC3.1.1-p23-M-R	ATATCATTAGGGAAGAGGTCAGATGAAGTGGTGTTCAC

Table S4： Northern Blot probe sequences (GFP probes include all probes except the miR156)

Oligo ID	Sequence
G-1	AGTAAAGGAGAAGAACTTTTCACTGGAGTTGTCCCAATTC
G-2	GTCCCAATTCTTGTTGAATTAGATGGTGATGTTAATGGGT
G-3	GTTAATGGGTACAAATTTTCTGTCAGTGGAGAGGGTGAAG
G-4	GAGGGTGAAGGTGATGCAACATACGGAAAACTTACCCTTA
G-5	CTTACCCTTAAATTTATTTGCACTACTGGAAAACTACCTG
G-6	AAACTACCTGTTCCATGGCCAACACTTGTCACTACTTTCT
F-1	TGGTGTTCAATGCTTTTCAAGATACCCAGATCATATGAAG
F-2	TCATATGAAGCGGCACGACTTCTTCAAGAGCGCCATGCCT
F-3	CGCCATGCCTGAGGGATACGTGCAGGAGAGGACCATCTTC
F-4	GACCATCTTCTTCAAGGACGACGGGAACTACAAGACACGT
F-5	CAAGACACGTGCTGAAGTCAAGTTTGAGGGAGACACCCTC
F-6	AGACACCCTCGTCAACAGGATCGAGCTTAAGGGAATCGAT
F-7	GGGAATCGATTTCAAGGAGGACGGAAACATCCTCGGCCAC
F-8	CCTCGGCCACAAGTTGGAATACAACTACAACTCCCACAAC
P-1	GGCCGACAAGCAAAAGAACGGCATCAAAGCCAACTTCAAG
P-2	CAACTTCAAGACCCGCCACAACATCGAAGACGGCGGCGTG
P-3	CGGCGGCGTGCAACTCGCTGATCATTATCAACAAAATACT
P-4	ACAAAATACTCCAATTGGCGATGGCCCTGTCCTTTTACCA
P-5	CCTTTTACCAGACAACCATTACCTGTCCACACAATCTGCC
P-6	ACAATCTGCCCTTTCGAAAGATCCCAACGAAAAGAGAGAC
P-7	AAAGAGAGACCACATGGTCCTTCTTGAGTTTGTAACAGCT
P-8	TGTAACAGCTGCTGGGATTACACATGGCATGGATGAACTA
P-9	ACATGGCATGGATGAACTATACAAACATGATGAGCTTTGA
MIR156	GTGCTCACTCTCTTCTGTCA

Table S5：CTV RNA silencing suppressor Subcellular localization primers

Primer ID	Sequence
pH7LIC5.1.1-C2B-F	TACAAGGATTACGCCGAGGTCATGGATGTGTTGACAGTAGTGG
pH7LIC5.1.1-C2B-C	GGATATCATTAGGGAAGAGGTCAAAACGACCCTTCGGCCC
pH7LIC5.1.1- p23-N4-F	TACAAGGATTACGCCGAGGTCATGGATGATACTAGCGGACAAA
pH7LIC5.1.1 -p23-N4-C	GGATATCATTAGGGAAGAGGTCAGATGAAGTGGTGTTCAC
pH7LIC5.1.1-t36-p23-F	TACAAGGATTACGCCGAGGTCATGGATAATACTAGCGGACAAA
pH7LIC5.1.1-t36-p23-C	GGATATCATTAGGGAAGAGGTCAGATGAAGTGGTGTTCAC
